# Sexual and reproductive health risk behaviours: higher education students’ perceptions

**DOI:** 10.1590/0034-7167-2021-0712

**Published:** 2022-07-29

**Authors:** Maria José de Oliveira Santos, Manuela Maria da Conceição Ferreira, Elisabete Maria Soares Ferreira

**Affiliations:** I Universidade de Trás-os-Montes e Alto Douro. Vila Real, Portugal; II Instituto Politécnico de Viseu. Viseu, Portugal; III Universidade do Porto. Porto, Portugal

**Keywords:** Risk Behaviours, Students, Condoms, Sexual and Reproductive Health, Unprotected Sex, Conductas de Riesgo para la Salud, Estudiantes, Condones, Salud Sexual y Reproductiva, Sexo inseguro, Comportamentos de Risco, Estudantes, Preservativos, Saúde Sexual e Reprodutiva, Sexo sem Proteção

## Abstract

**Objective:**

to understand higher education students’ perceptions of sexual and reproductive health risk behaviours.

**Methods:**

a descriptive study following a qualitative approach was conducted, using Pender’s Health Promotion Model as a theoretical and methodological framework. A thematic analysis of the data obtained from different focus groups was performed.

**Results:**

participants consider that factors such as communication with their sexual partner, the ability to negotiate and a positive attitude regarding condoms are positive aspects that will encourage consistent use of condom. The embarrassment felt at the time of purchase, the reduction of sexual pleasure and the growing stability of the relationship are usually seen as barriers.

**Final considerations:**

the study was crucial to identify some strategies that will be considered in further health promotion programmes, namely peer education, and will help promote personal and social skills and the (re)organisation of healthcare services.

## RESUMO

**Objetivo:**

conhecer as perceções dos estudantes do ensino superior sobre comportamentos de risco sexual e reprodutivo.

**Métodos:**

estudo descritivo de abordagem qualitativa, usando como referencial teórico-metodológico o Modelo de Promoção da Saúde de Pender. Foi realizada uma análise temática dos dados obtidos através dos grupos focais.

**Resultados:**

os participantes consideram que fatores como a comunicação com o parceiro sexual, a capacidade de negociação e uma atitude positiva face ao uso do preservativo poderão constituir benefícios para uma utilização consistente do preservativo. Já o embaraço para comprar o preservativo, a alegada redução do prazer e a estabilidade da relação amorosa são entendidos como barreiras a esse uso consistente.

**Considerações finais:**

foram identificadas estratégias a considerar na criação de programas de promoção da saúde sexual, nomeadamente a educação pelos pares para promover competências pessoais e sociais e a (re)organização dos serviços de saúde.

## RESUMEN

**Objetivo:**

conocer las percepciones de los estudiantes de educación superior sobre las conductas de riesgo en materia de salud sexual y reproductiva.

**Métodos:**

estudio descriptivo con un enfoque cualitativo utilizando el Modelo de Promoción de la Salud de Pender como marco teórico y metodológico. Se realizó un análisis temático de los datos obtenidos en los grupos focales.

**Resultados:**

una buena comunicación con la pareja sexual, la capacidad de negociación y una actitud positiva hacia el preservativo son aspectos benéficos para un uso regular del preservativo. La vergüenza que mucha gente todavía siente cuando va a comprar condones, la reducción del placer sexual y la estabilidad de la relación amorosa pueden actuar como barreras.

**Consideraciones Finales:**

se identificaron las estrategias a tener en cuenta en el diseño de los programas de promoción de la salud sexual, como la educación por pares, para promover las habilidades personales y sociales y la (re)organización de los servicios sanitarios.

## INTRODUCTION

The promotion of young people’s health should consider their specific needs, particularly those related to key issues, like sexual and reproductive health (SRH). Nowadays, it is commonly accepted that universities are capable of playing a key role in the promoting of their students’ health, creating solid learning, providing them with healthier experiences and promoting healthcare and well-being ^(1)^. In Western society, youth is marked by a great range of normative events. The transition to higher education is clearly one of those moments, since it involves the achievement of two developmental tasks: the construction of one’s autonomy and intimacy. When young people decide to initiate their sexual life, they are usually struggling with several other massive changes that are occurring in their lives, and the exercise of sexuality will have an impact on their reproductive process and biopsychosocial health^(2)^. Although there is a widespread conviction that most harmful behaviours are acquired before young people reach adolescence, several studies have showed that getting admitted to a higher education institution may increase SRH related risks. These risks are mainly caused by the inconsistent use of condom, and by alcohol and substance abuse that is often associated with casual sex and random partners. These behaviours are considered almost normative nowadays, especially when academic nightlife and parties are involved^(3-8)^. This sort of behaviour may affect negatively young people’s SRH, since it may cause unwanted pregnancy, sexually transmitted infections (STI)^(9)^ and sexual violence^(10)^. The lack of information, or the fact that it is often acquired from unreliable sources, overconfidence or a certain sense of invulnerability, and the existence of social and family taboos related to sexuality may lead young people to unwittingly use contraceptive methods, and they will not feel confident enough to ask their partners to use a condom or to seek SRH services. Those are behaviours that are becoming increasingly common among this particular age group^(11-12)^.

Risk behaviours and lifestyles are currently two important determinants of young people’s health. That way, health promotion programmes must include not only educational interventions, but also the development of personal and social skills, as well as accessible, appropriate and integrated health services that really meet young people’s needs^(13)^. From this perspective, understanding the sexual behaviours of higher education students is an absolute necessity from the epidemiological point of view, but knowing the perceptions, the rationality of choices and the factors that may increase or reduce sexual risk is also crucial to define successful intervention strategies meant to promote the adoption of safer sexual behaviours.

Different models emerged from the concern to implement strategies carried out to promote healthy behaviours. Pender’s Health Promotion Model (HPM) ^(14)^ is one of those health promotion models. It focuses on nursing intervention and follows different behavioural models that seek to understand changes in health-related behaviours. This model describes the multidimensional nature of persons as they interact with others and with the environment in their search for health and well-being^(15)^, and includes four fundamental concepts: health in its various dimensions, the environment (physical, social, and cultural), the persons in all their biopsychosocial complexity and in their capacity to make decisions, and nursing which is regarded as an agent of health promotion^(14)^. The HPM has been used by several researchers to study behaviours across several health areas, including the sexual behaviour of people with HIV/AIDS^(16-17)^. However, we found no theoretical references of its use in the promotion of SRH as part of the nursing practice in Portugal. In this context, we believe that the use of the theoretical support offered by the HPM is of great importance to understand the complexity of the determinants of SRH behaviours among higher education students and to define a SRH promotion programme that will be capable of meeting their specific needs.

## OBJECTIVE

To understand higher education students’ perceptions of sexual and reproductive risk behaviours.

## METHODS

### Ethical Aspects

The informed consent, produced according to the Helsinki Declaration guidelines (1975), was signed by all participants. The participants’ participation in the background questionnaire was voluntary and their anonymity was ensured. The study was approved by the National Data Protection Committee and the Ethics Committee of the University. To make sure that the respondents’ anonymity is preserved, their statements were identified with the letter “P” (P1 - for the participants in the first focus group (FG) and P2 - for the participants in the second FG), followed by a randomly assigned Arabic numeral.

### Theoretical and methodological frame of reference

This study was part of a broader research, a quantitative approach carried out to anlyse SRH behaviours among higher education students, with a particular focus on sexual risk behaviours and that helped raise several questions^(18)^. The aim of this study was to seek a deeper understanding of the behaviours related to the promotion of SRH, by studying the interrelationships that exist between different individual and contextual factors related to risk and protection to which young adults are exposed when they initiate their sexual activity. Evidence gathered in the course of this study demonstrated that the HPM is appropriate^(14)^.

The HPM focuses on three components that are independent but interconnected nonetheless: 1) Individual characteristics and experiences - that include prior behaviour that needs to be changed and personal factors (biological, psychological, sociocultural); 2) Feelings and knowledge about behaviours that need to be changed and one wishes to achieve - that include feelings about health behaviour, perceived benefits and barriers to action, perceived self-efficacy, interpersonal and situational influences; 3) Whitin behavioural outcome there is a commitment to follow a certain plan of action, competing demands and preferences, which are the alternative behaviours over which people have low or high control and that will help people achieve the health behaviour they truly desire.

### Type of study

A descriptive study following a qualitative approach was conducted, since the main objective was to understand the perceptions, opinions and feelings that the participants have regarding the topic under study.

### Methodological Procedures

Data was collected using the FG (Focus Group) technique and a semi-structured interview was conducted to guide the discussion. This technique allows a strong interaction between the groups as they question and discuss complex and private issues, such as sexual behaviours^(19)^. The 4 essential steps in conducting focus groups suggested in the literature^(19-20)^, planning, recruiting, moderation and data analysis and reporting, were dully considered.

### Study settings

The focus groups took place in one of the university meeting rooms. We made sure that the room was comfortable, allowed for some privacy and was capable of providing a circular organization that would favour the group dynamics. This dynamic atmosphere was the result of the presence and of the interaction developed between the moderator and the participants, and each session took on average two hours. Before conducting the FG, there was an individual presentation and an icebreaker activity. There was also a brief description of the FG methodology that was important to clarify the role played by the moderator and the observer. This moment was used to recall the objectives of the study and to stress how important it was for everyone to take active part in the discussion.

### Source of data

Invitations to participate in the study were sent by email to the addresses provided by students who were interested in taking part in this project. Twenty-three students agreed to participate. Those students were informed via telephone about the objectives of the study, the confidential and voluntary nature of participation, the location and the procedures that would be followed. 21 students responded to the invitation on the prescribed date. Two mix-gender groups were formed-the first group consisted of 10 students (7 girls and 3 boys) and the second group consisted of 11 students (9 girls and 2 boys)- to ensure some sort of group heterogeneity achieved through gender representativity and the scientific field of studies represented.

### Data Collection and Organization

At the end of the sessions, a questionnaire was administered to collect information on the participants’ sociodemographic background (gender, age, origin) and on their sexual and reproductive behaviours (whether or not they have already had sexual intercourse, use contraceptive methods, used a condom during their last sexual intercourse, or if they usually participate in health check sessions). The sessions were recorded, after the participants’ prior consent. Once the FG were completed, the observations on the group dynamics were recorded and the information collected was transcribed and would become the corpus of the study.

### Data analysis

The data analysis process requires a theme/category-based content analysis^(21-22)^. After the transcription, the text was subjected to pre-analysis, through the so-called “floating” reading process, which allowed the identification of a set of words, themes or concepts using a clipping process. Those units were classified and grouped according to the semantics in the themes and categories identified beforehand. After that, data inference and interpretation procedures were carried out.

The themes and categories identified based on the HPM components were as follows: 1) Individual characteristics and experiences - in this theme the mobilization of the participants’ responses led to the definition of two categories: “individual characteristics” and “risky sexual behaviour”; 2) Knowledge and feelings about specific behaviours - in this theme the analysis of the participants’ words allowed the definition of six categories: “feelings experienced,” “perceived benefits,” “perceived barriers,” “self-efficacy,” “interpersonal influences” (family, peers and health professionals) and “ situational influences” (alcohol and drug use associated with sexual intercourse); 3) Safe sexual behaviours: a process under construction - in this theme the participants suggested some strategies that will be included in a programme to promote safe sexual behaviours among higher education students. The analysis of the participants’ answers allowed the identification of two categories: “educational strategies” and “SRH health surveillance”.

## RESULTS

Most of the 21 participants were female (16 girls and 5 boys), with a mean age of 21 years, who were all sexually active and had their first sexual intercourse, on average, at the age of 17. They all stated that they have used some kind of birth control method, such as condoms or dual contraceptive method (pill/condom), but only half of them reported using a condom in their last sexual intercourse. Fourteen participants reported using SRH surveillance. Four out of the seven who did not use that sort of surveillance were male students. The results will be presented in accordance with the major themes and categories defined a priori, using the HPM.

### Individual background and experiences

In the individual background category related to the adoption of safe sexual behaviour, personality traits were mentioned more often than any other.

*Strong personality is very important indeed.* (P2.3.)

*Awareness, responsibility.* (P1.6)

Risky sexual behaviour is considered a frequent conduct and is mostly associated to male individuals. Students point out unsafe sexual behaviours, like sex without condom and occasional partners, usually associated with alcohol consumption.

*I think that the main risk behaviours are really unprotected sex, with several partners.* (P1.5)

*Because I know that many of my friends here at the university are capable of meeting some girl today, have a few drinks and then wake up next to her in the morning...Can you believe that?...and what about the condom?* (P1.4)

### Feelings and knowledge of safe sexual behaviour

The interpretative analysis of the data showed that, when they talked about their feelings, it became obvious that students’ fears and worries condition the experience of a full sexuality. The fear of unwanted pregnancy was the most prominent.

*Getting pregnant at our age… I think this is one of the biggest worries…and diseases too.* (P1.7)

*Diseases…that’s what scares me the most. Every time we hear those numbers…70% people infected with HPV.* (P1.1)

Paradoxically, some students are convinced that young people are careless and negligent, not only regarding the prevention of sexual risk behaviours, but also regarding health surveillance.

*It’s a laissez-faire attitude, after all I’ve never caught anything.* (P1.4)

*I think it’s pure laziness.* (P1.3)

Although the participants agree that the use of condoms is very important, some of them showed a striking lack of knowledge on how to use it. Their responses demonstrate that even when they mean to do so, they are not always able to use the condom.

*Even though we have that conviction, always use condoms, things are different in real-life situations.* (P2.1)

*We do know that we are being irresponsible* [...] *but in the heat of the moment, sometimes we don’t make the right decision.* (P2.1)

The analysis of the participants’ responses shows that a positive attitude towards condoms stands out as one of the perceived benefits of safe sexual behaviours, as condoms are regarded as a way to prevent unwanted pregnancies and because they are the only contraceptive method that protects people from STI.

*We have to use it… in my case, I know people who have already caught diseases, here at school* [...]. (P1.1)

*We might not get pregnant but diseases exist.* (P2.9)

Similarly, they seem to understand that the communication with their sexual partner is of the utmost importance. It is crucial to negotiate the use of condoms, and to know about their past sexual conduct.

*I think that communication should always be present and so should prevention.* (P2.4)

*In my case, either you use the condom or nothing happens.* (P1.5)

[...] *I was the first to put all my doubts on the table, since he had already had other relationships and other sexual encounters and I cleared everything up. I think this is essential.* (P2.3)

Most of the participants also believe that they have the right to ask their partner about their sexual history, and they feel that they should know if they had ever been tested for HIV. They also mention that they would not mind taking the test.

Negative beliefs and the embarrassment they feel when they have to buy condoms were perceived as barriers to the adoption of safe sexual behaviours. These beliefs are mostly found among boys.

*Condom always reduces sexual pleasure.* (P1.5)

*Condom decreases sensitivity, kills the spontaneity of the moment, and can be a barrier between the couple.* (P2.4)

Most of the participants, also consider that the embarrassment they sometimes feel when they have to go and buy condoms can represent a barrier to the adoption of safe sexual behaviours. This situation is pointed out by both genders. Two students claim that:

[...] *people still look sideways, I bought condoms once and the desk woman was staring at me, like: a girl …buying condoms!* (P1.2)

*Even if I had the chance and even if I felt confident enough to talk about it, I could never ask for condoms or buy condoms.* (P2.3)

The status of the relationship is often referred to justify abandonment of condom use: young people argue that the stability of the relationship and the trust they put in their sexual partner make condoms less indispensable.

*Has she ever asked me not to use condom? It’s happened... but with me it’s different... there’s already a basis of mutual trust.* (P1.4)

*I have heard many colleagues saying that after a year of dating, using a condom shows that we don’t trust our partner.* (P2.8)

However, there are students who disagree with this logic of protection based on how much we trust our partner, claiming that:

*‘I don’t agree at all* [...], *using a condom is not a matter of trust it is a matter of health.* (P2.6)

Students also refer some barriers to accessing SRH care, namely issues that have to do with their anonymity and confidentiality, since they live in a small town where everyone knows each other.

*In general, young people don’t seek those services, they are ashamed, they feel intimidated.* (P1.3)

*We turn to those services and everybody knows that we have been there… because of this, because of that.* (P1.10)

[...] *in my neighbourhood, the nurses and doctors know my mother, it’s complicated.* (P1.3)

As for interpersonal influences and considering the different agents of socialisation, friends and peers have proved to be a massive influence, particularly when it comes to share information about sex and sexuality.

*My friends and colleagues were my first source of information. It might not have been the most reliable source information, but it was always the first.* (P2.4)

Participants also recognise the dual influence of the group, mentioning that:

*‘If it is a good group, it can help… a group where all differences are accepted.’*(P2.8)

*Peer pressure is often associated with excessive alcohol consumption, which is increasingly frequent at university parties.* (P2.4)

Although everyone seems to agree that family is the best emotional space to achieve the development of healthy attitudes and behaviours, most of the participants stated that they don’t talk to their parents about sex-related issues.

*I don’t talk to my parents… my parents are not prepared to address these issues.* (P1.9)

Students, and girls in particular, consider that health professionals are a credible and trustworthy source of information on sexuality issues and highlight the chance they are given to speak openly and share doubts, as they recognize that there is a lot they don’t know, especially about STI.

*To clear doubts and talk about important things, we trust health professionals* [...] *it’s something I do with the nurse…it’s with her that I can clarify any doubt and talk about everything.* (P2.2)

*AIDS is the “bogeyman”, but there are other sexually transmitted diseases which young people don’t know about and that I don’t know about either* [...]. (P1.4)

When they are asked to point out negative situational influences, students often refer to the consumption of psychoactive substances (alcohol and drugs) that will often lead to unsafe sexual relations with occasional partners, especially during nightlife experiences and academic parties.

*Sometimes, it’s not just alcohol, you mix a lot of things.* (P1.5)

*In those moments, a person under the influence will forget responsibilities* [...], *and in many of those sexual intercourses no barrier will be used, neither pill nor condom. (P2.4)*

*During our most popular college party* [Queima das Fitas], *there is a lot occasional sex in the toilets, or even in public places.* (P1.3)

### Safe sexual behaviours: an ongoing process

Students were asked to come up with strategies to improve adherence to safe sexual behaviours, but it was very hard for them to come up with valid and viable suggestions.

As educational strategies they mainly suggest the implementation of more interactive approaches, focus groups, group reflection, debate and even role-playing. They also suggest that young people should be placed in situations where they would have to face their fears. The use of “shocking messages/images” would be part of such strategy.

*Issues need to be addressed as they are…bluntly. We cannot be afraid to scare people.* (P2.1)

*Life stories, narratives, debates. I think that’s what captivates us the most and draws our attention. I think those stories scare us to the bone and that makes us think. We may or may not change our behaviour* [...], *but we will think about that.* (P2.2)

They also consider that the (re)organisation of SRH services is essential so they can meet the specific needs of young adults, and that they should include planned consultations.

*We attend planned medical appointments when we are younger, those scheduled appointments should be available when we are older, whether we are planning on starting our sex life or not, because seeking healthcare services isn’t important to most of us.* (P1.3)

They also stress the importance of the compulsory STI partner notification that would include HIV/AIDS.

*I also think so, and for example, getting tested for HIV/AIDS should be compulsory. In terms of public health, it should be compulsory. I know it tinkers with ethical issues, with issues related to individual freedom, but it is a situation that concerns everyone.* (P2.4)

## DISCUSSION

The data obtained in the FG allowed a better understanding of how students perceive issues related to SRH, particularly the factors that may have a greater influence on sexual risk behaviours. Personality traits are quite likely, according to the participants’ words, to influence sexual risk behaviour, and those responses are in line with some studies that have demonstrated how important one’s personality traits are, especially the search for sexual sensations, to the existence of sexual risk behaviours^(22)^. As for the feelings, the students are afraid about unwanted pregnancy and possible diseases, but, apparently, many of them seem to show little concern with issues like the prevention of sexual risk behaviours or, most of all, health surveillance. These results are in line with those found in several studies already conducted that show that the fear of getting pregnant is still the primary motivating factor for the use of condoms. The risks associated with STI are still undervalued^(23-25)^. This apparent carelessness may contribute to perpetuate the high rates of STI infection among young adults^(9)^.

Although there is a positive attitude about condoms among students, some of them show low self-efficacy to use them consistently. The literature supports that a high level of self-efficacy optimises condom use and the ability to negotiate safer sex^(26)^, and a more favourable attitude towards condoms is predictive of a more consistent use^(27)^.

Positive attitude towards condoms and the ability to communicate and negotiate condom use were identified as perceived benefits. In this regard, some authors^(28)^, state that the romantic ideals associated with the spontaneity of the intercourse, and the difficulty in confessing that there were other partners are elements that will have a great impact on the students’ attitude towards condom use. Knowledge of the partner’s sexual history was also perceived as a benefit; however, discussing the partner’s sexual past may not result in safe sexual practices. Some studies have revealed that many sexual partners lie about their sexual past, and that there are high rates of unrevealed casual sexual involvement (26.1%), even in serious relationships^(28)^.

According to other studies, the romantic relationship status can also determine sexual risk^(23,29)^, since, even though students realize the importance of consistent condom use, exceptions are allowed based on how strongly they trust their partner’s sexual past, and, in longer relationships, condom is often replaced by oral contraceptives. Trust can contribute to develop a sense of invulnerability, since each partner is “unique” and the relationship between both is accompanied by emotions, feelings and mutual concerns, which go beyond individual protection issues^(30)^. Other barriers to consistent condom use are the belief that condoms interfere with sexual pleasure and the embarrassment associated with their purchase. The valorisation of pleasure over prevention has been pointed as a factor that compromises condom use, since the condom is often associated with decreased sexual pleasure and has little to do with people’s erotic imaginary^(31)^. Boys don’t seek SRH services as much as they should because they believe that those services are essentially oriented to girls. In this regard, there is a growing concern to make sure that SRH consultations are prepared to address the specific needs of both boys and girls^(30-32)^.

As for interpersonal influences, young people are particularly sensitive to the influences of friends and peers and to the rules shared by the group^(33-34)^. This data is in line with the results found in other studies that stress that peers are the main source of information regarding sex and sexuality, in about 60% of the cases^(27)^. Some studies have also documented that excessive drinking may be associated with peer influence, and that the association of alcohol with sexual intercourse can increase unsafe sexual behaviours^(34)^, a finding that was also confirmed in this study. Health professionals play a massive role in the dissemination of credible information on safe sexual behaviours. This opinion is especially prevalent among girls.

When asked to express their opinion about possible situational influences, students point out academic parties, discos and bars. In their opinions, those are environments where contacts with occasional partners are easily established and where there is excessive consumption of alcohol and drugs that will often lead to risky sexual practices. From this perspective, there is evidence of a growing phenomenon among college students, the so-called hookups culture, a practice that encourages brief and casual sexual encounters between individuals who are not in a committed relationship^(5,7)^. A review of the literature suggests that sexual encounters of this type are frequent: for instance, 60% to 80% of North American college students state that they have already experienced this sort of sexual practices^(36)^. These practices may carry an increased risk for STI, since in many situations condoms are not used and individuals are more likely to have multiple sexual partners^(5,7,36)^.

Excessive consumption of psychoactive substances among young adults is associated with social drinking and peer pressure. Several authors have claimed that casual sex is a common behaviour among college students and is often related to alcohol consumption^(4,6,37)^. In most university parties, the phenomenon called “binge drinking”, defined as a episodic and excessive consumption of alcohol over a short period of time, usually in a group context, is becoming increasingly popular^(6-7)^. According to the National Survey - 2016/17, that focused on consumption of psychoactive substances among the Portuguese population^(38)^, binge drinking is practiced by 11.8% of young people who are between 15 and 24 years old. The consumption of psychoactive substances associated with sexual intercourse can be problematic, since it can cloud one’s judgement, and will directly affect the ability to practice safe sex and refuse to have unwanted sexual intercourse^(39)^. Alcohol is also used as an emotional stimulant, reducing inhibitions and increasing confidence for new social contacts, which will make sex with occasional partners much easier^(7,36)^.

Stressing the importance that individuals should be able to decide about their health, students were encouraged to discuss and suggest strategies to promote safe sexual behaviours. They failed to point long term strategies. For the most part, they suggested change in educational approaches. They stated that these should be more interactive and favour a more cultural approach, that there should be more debates, discussion and reflection groups and that strategies or activities where students should have to confront their fears should be implemented. Some authors justify the students’ immature discourse where sexual risks are devalued by the way information is transmitted in prevention campaigns, namely in information campaigns on HIV/AIDS that use a theoretical approach based on the biomedical and rationalist model^(39)^. Although students make little use of SRH services, they value regular health surveillance and contact with health professionals, especially when they respect the idiosyncrasies of young people.

### Limitations of the study

The students’ perceptions of sexual risk behaviour may be associated to social desirability bias, due to the sensitive nature of the subject under study. The use of the HPM may also constitute a limitation, not only because the model is complex, but also because it assumes that sexual behaviour is always a planned behaviour, forgetting that in young people it is often impulsive and determined by emotional and affective factors.

### Contributions to nursing

Data analysis and the reflections carried out on the topic under study suggest that the promotion of SRH among higher education students will consider two different levels of intervention: the (re) organisation of existing healthcare services, namely of those located on the University campus, so they can meet their users’ specific needs and the design and implementation of educational programmes that will help fill the gaps identified. At the organisational level, SRH services should provide SRH counselling and surveillance consultations, and raise the awareness of sexually active young people about the importance of STI screening. At the educational level, the active participation of students in the design of sex education programmes should be considered, programmes that will include more dynamic and reflective strategies to promote personal and psychosocial skills and to raise young people’s awareness about the importance of consistent condom use.

## FINAL CONSIDERATIONS

The constructs that are part of the HPM were crucial to identify important aspects that nurses will have to consider when they design SRH promotion programmes for higher education students that will involve sex education and the organization of healthcare services. The analysis of the responses provided allowed us to understand some of the motivations underlying the students’ sexual risk options and to conclude that they are a group greatly exposed to sexual risk. The consistent use of condoms is the health-promoting behaviour that should be consolidated and that should be supported by a more regular SRH surveillance. A consistent condom use should be emphasised, as young people find too many reasons to neglect its use: they don’t use it because they trust their partner or because they believe it will reduce their sexual pleasure. Alcohol and drug consumption and the way it promotes sexual intercourse during university parties also justifies inconsistent condom use and the existence of casual sex partners. That way, in addition to providing information on the risks of unprotected sex and to implementing global sexual risk mitigation strategies during college parties, nurses should also invest in peer education in order to promote personal and social skills (self-efficacy, assertive communication, condom negotiation) that will prevent unsafe sex situations, in an attitude of social responsibility and citizenship that is necessary for health reasons.

## SUPPLEMENTARY MATERIAL

Santos MJ. Sexual and Reproductive Health of Higher Education Students - Contribution to the development of intervention programs [Dissertation on the Internet]. Porto (Portugal): Universidade do Porto, Instituto de Ciências Biomédicas Abel Salazar; 2017. 386 p. Available from: https://hdl.handle.net/10216/105488

0034-7167-reben-75-06-e20210712-sup01
