# Supplementary material for: Sexual and reproductive health risk behaviours: higher education students’ perceptions
Source: Rev Bras Enferm. 2022 Jul 29;75(6):e20210712. doi: 10.1590/0034-7167-2021-0712 (PMC9749776; doi:10.1590/0034-7167-2021-0712)
Supplement: 0034-7167-reben-75-06-e20210712-sup01 [file 0034-7167-reben-75-06-e20210712-sup01.pdf]

U.PORTO

DOUTORAMENTO

CIÊNCIAS DE ENFERMAGEM

Saúde Sexual e Reprodutiva de Estudantes do Ensino Superior

Contributo para o desenvolvimento de programas de intervenção

Maria José de Oliveira Santos

D

2017

MARIA JOSÉ DE OLIVEIRA SANTOS

SAÚDE SEXUAL E REPRODUTIVA EM ESTUDANTES DO ENSINO SUPERIOR: CONTRIBUTO PARA O DESENVOLVIMENTO DE PROGRAMAS DE INTERVENÇÃO

Tese de candidatura ao grau de Doutor em Ciências de Enfermagem, submetida ao Instituto de Ciências Biomédicas de Abel Salazar da Universidade do Porto.

Orientador - Doutora Manuela Maria da Conceição Ferreira
Professora Coordenadora
Escola Superior de Saúde de Viseu do Instituto Politécnico de Viseu.

Coorientador - Doutora Elisabete Maria Soares Ferreira
Professora Auxiliar
Faculdade de Psicologia e de Ciências da Educação da Universidade do Porto.

Dedicatória

Aos meus "Amores", Vasco, Beatriz, Francisco e Luís

AGRADECIMENTOS

Findo este percurso que permitiu a elaboração deste trabalho de investigação, espero conseguir agradecer a todos aqueles que de alguma forma permitiram concretizar este imenso desafio de crescimento pessoal e profissional.

À Professora Doutora Manuela Ferreira, o meu reconhecimento pela orientação científica, pelas aprendizagens, reflexões, pragmatismo e constante motivação, mas sobretudo pela confiança que depositou sempre no nosso trabalho.

À Professora Doutora Elisabete Ferreira, pelas suas pequenas grandes sugestões, que se revelaram uma mais-valia na definição do pecurso de investigação. Pela motivação, positivismo e grande entusiasmo que colocou sempre na relevância destes resultados para os estudantes universitários.

Duas orientadoras, dois olhares e duas perspetivas que me fizeram ver a investigação com outro olhar mais integrador.

Ao Professor Doutor João Duarte, pela valiosa colaboração na análise estatística, mas sobretudo pela generosidade com que partilhou o seu saber e simpatia com que sempre nos acolheu.

Aos estudantes que participaram nesta investigação, pela sua generosa colaboração, sem a qual este estudo não teria sido possível. Espero conseguir passar para a realidade as Vossas vivências e preocupações.

À direção da Escola Superior de Enfermagem da Universidade de Trás-os-Montes e Alto Douro, pelas facilidades concedidas.

Aos amigos e colegas do departamento, Carmo, Anabela, Filomena, Fátima, Carlos e Castro, pelo incentivo e imenso apoio na gestão das atividades académicas, particularmente importantes na fase final do trabalho.

À amiga Carmo, por ter procurado proporcionar condições indispensáveis à conclusão deste trabalho de invetigação, mas sobretudo pelo incentivo e pela amizade com que sempre nos distinguiu.

À amiga Anabela, pelo olhar atento, incentivo, valiosa troca de ideias e permanente disponibilidade que espero conseguir retribuir um dia.

À Teresa Carvalho, pela disponibilidade e preciosa colaboração na revisão e formatação final da tese.

À amiga Sofia Dias, pela disponibilidade, incentivo e competente contributo na revisão do manuscrito final.

Aos meus amigos, além-mundo académico, pela compreensão nas ausências, mas qualidade do tempo partilhado. Ficam na minha memória os muitos abraços apertados…

À minha família, em particular à minha mãe, à Maria e ao Mário, o meu porto seguro, pelo seu infinito apoio e amor incondicional. Ao meu pai (*in memoriam*), que me ensinou que só o conhecimento nos traz a verdadeira liberdade.

Aos meus filhos, Vasco, Beatriz e Francisco, vocês são a razão do meu encanto pela vida, fazendo-me acreditar todos os dias, que tudo vale a pena… Desculpem-me as ausências e as impaciências e fica a promessa de um regresso por inteiro.

Ao Luís, pelo amor, incentivo, reflexões e partilha, mas sobretudo por acreditar, muitas vezes mais do que eu, que é possível concretizar o impossível.

A todos os que de alguma forma contribuíram para a concretização deste trabalho, e que eu não tive oportunidade de mencionar, o meu sincero OBRIGADA.

PUBLICAÇÕES CIENTÍFICAS ASSOCIADAS AO PERCURSO DE INVESTIGAÇÃO

Artigos em revistas internacionais

- Santos, M.J., Ferreira, E., Duarte, J., & Ferreira, M. (2017). Risk factors that influence sexual and reproductive health in Portuguese university students. *Internacional Nursing Review*, (aprovado para publicação, Article ID: INR 12387). doi: 10.1111/inr.12387

- Santos, M.J., Ferreira, E., Duarte, J., & Ferreira, M. (2016). Adaptação portuguesa e validação da versão reduzida da Condom Use Self-efficacy Scale. *Revista Internacional de Andrologia*, 15 (1), 23-30. doi:10.1016./j.androl.2016.06.002

- Santos, M.J., Ferreira, E., & Ferreira, M. (2016). Knowledge of and attitudes toward sexual and reproductive health among college students. *Atención Primaria, 48* (Esp Cong 1), 188-194.

Artigos em revistas nacionais

- Santos, M.J., Ferreira, E., & Ferreira, M. (2015). Barreiras no acesso dos jovens adultos aos cuidados de saúde sexual e reprodutiva. *Revista da Associação Portuguesa de Enfermeiros Obstetras, 15* (6), 4-8.

Resumos de comunicações em encontros científicos

- Santos, M.J., Ferreira, E., & Ferreira, M. (2016). Contraception in college students: Practices and challenges. *Atención Primaria, 48* (Espec Cong 1), 117-118.

- Santos, M.J., Ferreira, E., & Ferreira, M. (2014). Adaptação portuguesa e validação da versão reduzida da Condom Use Self-efficacy Scale (CUSES) em estudantes do ensino superior. *Revista de Enfermagem Referência, 4* (suplemento AO 2), 90.

- Santos, M.J., & Ferreira, E. (2014). Acesso aos serviços de saúde sexual e reprodutiva pelos estudantes do ensino superior. *Revista de Enfermagem Referência, 4* (suplemento AO 2), 644.

- Santos, M.J., Ferreira, E., & Ferreira, M. (2014). Study of factors determining condom use in college students. *Atención Primaria, 46* (Espec Cong 1), 29.

- Santos, M.J., Ferreira, E., & Ferreira, M. (2013). Portuguese adaptations of the sexual sensation seeking scale whiten college students. Symposium: Psychosocial determinants of health. *Atención Primaria, 45* (Espec Cong 1), 59-131, 81.

RESUMO

Introdução: Os estudos sobre os comportamentos sexuais e reprodutivos em jovens adultos sugerem que há um número significativo de jovens que mantém comportamentos sexuais de risco. Estes podem resultar numa gravidez não planeada ou num aumento da exposição ao risco de infeções sexualmente transmissíveis (IST), pelo que se devem considerar estratégias de educação para a saúde, mas também a implementação de intervenções que visem a promoção de competências pessoais e sociais no âmbito destes grupos. Os profissionais de enfermagem, pelo perfil de competências que detêm, podem contribuir de forma significativa para promover a saúde nesta área.

Objetivo: O presente trabalho teve como objetivo geral contribuir para a compreensão dos comportamentos de saúde sexual e reprodutiva (SSR) de estudantes do ensino superior, visando traçar linhas de orientação e estruturar estratégias para o desenvolvimento de programas de promoção da SSR nesta população.

Metodologia: Procedeu-se a um estudo quantitativo, transversal, observacional e descritivo-correlacional. A população-alvo foi constituída por 1946 estudantes (64% raparigas e 36% rapazes), com idade média de 21 anos, de uma universidade do norte de Portugal. Os dados foram obtidos pela aplicação de um protocolo de investigação, constituído por escalas (Inventário de Conhecimento Sobre SSR - ICSSR, Escala de Atitude de SSR - EASSR, Escala de Autoeficácia para Usar o Preservativo - CUSES; Escala de Busca de Sensações Sexuais - SSSS e Escala de Satisfação com o Suporte Social - ESSS), e questões de caracterização individual e dos comportamentos sexuais e reprodutivos. O estudo quantitativo foi triangulado com um estudo qualitativo que utilizou a técnica de grupos de discussão focalizada para aprofundar a análise e compreensão das motivações subjacentes a algumas das opções assumidas pelos estudantes.

Resultados: A maioria dos participantes (76,9%) é sexualmente ativa, tem um conhecimento mediano e uma atitude SSR favorável. O método contracetivo mais utilizado pelas raparigas é a pílula (52,9%) e por rapazes o preservativo (32,1%). Os estudantes estão expostos a riscos sexuais e reprodutivos, por não usarem consistentemente o preservativo (60,5%), por terem relações sexuais com parceiros ocasionais (32,0%), dos quais desconhecem o passado sexual e o nível de risco, e pela combinação de relações sexuais com álcool (33,0%) ou drogas (9,7%). Os rapazes e os estudantes mais velhos são os que estão mais expostos ao risco. Os estudantes que namoram abandonam o preservativo, com base numa forte confiança no parceiro, apesar de cerca de um quarto (26,1%) indicar ter tido relações sexuais fora da relação de namoro. Os estudantes que fazem escolhas mais acertadas de contraceção e os que se protegem mais do risco de IST têm uma atitude SSR mais favorável, dão uma maior importância aos pares, têm uma maior autoeficácia para o uso do preservativo e uma menor procura de sensações sexuais. Existe uma subutilização dos serviços de SSR e pouca adesão a comportamentos de vigilância de saúde, particularmente por parte dos rapazes. Os preditores do uso consistente do preservativo, foram estudados através do Modelo de Promoção de Saúde de Pender, tendo-se verificado uma forte influência dos benefícios percebidos, dos sentimentos positivos e das influências interpessoais no compromisso com o uso do preservativo,

que por sua vez é a variável que melhor prediz o comportamento de usar o preservativo ($\beta=0{,}580$, $p<0{,}001$). A análise dos discursos gerados nos grupos focais, confirmaram as grandes tendências observadas no estudo quantitativo. Uma maior preocupação e responsabilidade por parte das raparigas, uma grande preocupação com a gravidez e uma reduzida perceção de vulnerabilidade face às IST. Os estudantes têm um discurso favorável à utilização do preservativo, admitem usá-lo, mas têm muitas exceções, o que os deixa expostos ao risco.

Conclusões: Com base nos resultados, podemos inferir que os estudantes do ensino superior têm práticas contracetivas suscetíveis de serem melhoradas e são um grupo com alguma exposição ao risco para IST. A utilização consistente e correta do preservativo é o comportamento promotor de saúde que importa consolidar, associado ao evitamento de situações de maior exposição ao risco e a uma vigilância de SSR mais regular. Os resultados sugerem como estratégias de intervenção, a adequação dos serviços de saúde às necessidades específicas dos estudantes e o delineamento de programas educacionais para colmatar as lacunas identificadas.

A adoção de comportamentos salutogénicos é um desafio que se tem colocado sistematicamente aos profissionais de saúde, pois muitas vezes são identificados os determinantes desses comportamentos, mas a fraca compreensão das suas relações e interações compromete o resultado final pretendido. Neste contexto, o Modelo de Promoção da Saúde de Pender, ajudou a compreender essa complexidade, pelo que foi usado como referencial organizativo de medidas que consideramos pertinentes para promover a mudança de comportamentos sexuais de risco, quer usando estratégias educacionais, quer organizacionais, otimizando os recursos disponíveis ao nível dos cuidados de saúde.

Palavras-chave: Comportamentos sexuais de risco, estudantes ensino superior, enfermagem, preditores, saúde sexual e reprodutiva.

ABSTRACT

Introduction: Studies on sexual and reproductive behaviours in young adults suggest that there is a significant number that maintains risky health behaviours that might result in unintended pregnancy or increased exposure to the risk of sexually transmitted infections (STI). Therefore, health education strategies and interventions should be implemented to promote personal and social skills. Considering the nurses' skill profile, they can contribute significantly to health promotion in this area.

Objective: The main objective of this study was to contribute to the understanding of sexual and reproductive health (SRH) behaviours in college student, in order to design guidelines for the development of SRH programs in this population.

Methodology: A quantitative, cross-sectional, observational and descriptive-correlational study was carried out. The target population consisted of 1946 students (64% women and 36% men), with a mean age of 21 years, from a university in the north of Portugal. The data were collected through the application of a questionnaire protocol, consisting of scales (SRH Knowledge Inventory, SRH Atitude Scale, Condom Use Self-efficacy Scale - CUSES, Sexual Sensation Seeking Scale - SSSS and Social Support Scale), and issues of individual and sexual and reproductive behaviours characterization. The quantitative study was triangulated with a qualitative study assessed with the focus group technique, used to deepen the analysis and understanding of the motivations underlying some of the options taken by students.

Results: The majority of the participants (76.9%) are sexually active, have a medium knowledge and a favorable SRH attitude. The contraceptive method most used by women is the pill (52.9%) and by men the condom (32.1%). Students are exposed to sexual and reproductive risks because they do not use condoms consistently (60.5%), because they have sex with occasional partners (32.0%), of whom they are unaware of sexual past and level of risk, and by combining sexual intercourse with alcohol (33.0%) or drugs (9.7%). Male and older students are the ones most at risk. Students involved in a committed relationship drop out of the condom use, based on a strong trust in the partner, although about a quarter (26.1%) indicate they have had sex outside the dating relationship. Students who make better contraception choices and those who have a more protective behaviour have a more favorable SRH attitude, gave greater importance to peers, have a greater self-efficacy for condom use, and a lower demand for sexual sensation seeking. There is underutilization of SRH services and little adherence to health care surveillance behaviours, particularly by young men. The predictors of consistent condom use were studied through the Pender's Health Promotion Model. It was observed a strong influence of perceived benefits, positive feelings and interpersonal influences on the commitment to condom use. The commitment is a variable that best predicts the behaviour itself of using the condom ($\beta=0.580$, $p<0.001$). The analysis of the discourses generated in the focus groups, confirmed the main trends observed in the quantitative study. A higher concern and responsibility of women, associated to a strong preoccupation with the pregnancy and a reduced perception of vulnerability to the STI. Students have a condom-friendly discourse, admit to using it, but report many exceptions, resulting in higher risk exposure.

Conclusions: Based on the results of the present study, we can infer that college students have contraceptive practices that can be improved and are a group with some risk exposure to STI. Consistent use of condom is a health promoting behaviour that must be consolidated, associated to the avoidance of risk exposure situations and more regular SRH surveillance. The results point to intervention strategies based on the adequacy of the health services to the specific needs of college students and the design of educational programs to fulfill the identified gaps.

The adoption of salutogenic behaviours is a challenge that has been systematically posed to health professionals, once the determinants of these behaviours are often identified, but poor understanding of their relationships and interactions compromises the understanding of the final result. In this context, the Nola Pender' Health Promotion Model allowed us to understand this complexity and was used as an organizational reference for measures that are considered relevant to promote changes in sexual risk behaviours, either through educational and organizational strategies, by optimizing the health care services.

Keywords: Sexual risky behaviours, college students, nursing, predictors, sexual and reproductive health.

ÍNDICE GERAL

ABSTRACT XI
ÍNDICE DE TABELAS XV
ÍNDICE DE FIGURAS XIX
ABREVIATURAS E SIGLAS XXI

INTRODUÇÃO 1

CAPÍTULO 1 – ENQUADRAMENTO CONCEPTUAL 9
1.1. Políticas de desenvolvimento da saúde sexual e reprodutiva em Portugal 11
1.2. Promoção da saúde sexual e reprodutiva 20
1.2.1. Promoção da saúde 20
1.2.2. Promoção e educação para a saúde 23
1.2.3. Modelos de mudança de comportamento 25
1.3. Saúde sexual e reprodutiva em estudantes do ensino superior 35
1.3.1. Os estudantes do ensino superior 35
1.3.2. Comportamentos de saúde sexual e reprodutiva 37
1.3.3. Determinantes dos comportamentos de risco e protetores para a saúde sexual e reprodutiva dos jovens 44
1.4. Saúde sexual e reprodutiva como foco de atenção em enfermagem 51

CAPÍTULO 2 – METODOLOGIA GERAL 57
2.1. Objetivos do estudo 59
2.2. Desenho do estudo 59
2.3. Estudo quantitativo 61
2.3.1. Tipo de estudo 61
2.3.2. População e amostra 61
2.3.3. Procedimentos de recolha de dados 65
2.3.4. Instrumentos de recolha de dados 65
2.3.5. Adaptação, construção e validação de instrumentos 68
2.3.6. Análise estatística 75
2.4. Estudo qualitativo 76
2.4.1. Percurso metodológico - Grupos de discussão focalizada 77
2.4.2. Planeamento e procedimentos de recolha de dados 79
2.4.3. Instrumentos de recolha de dados 81
2.4.4. Participantes no estudo 81
2.4.5. Análise interpretativa de dados 83
2.5. Considerações éticas 84

CAPÍTULO 3 – ESTUDOS EMPÍRICOS METODOLÓGICOS 87
3.1. Artigo 1 - Adaptação portuguesa e validação da versão reduzida da *Condom Use Self-efficacy Scale* 89

3.2. Artigo 2 - Adaptação portuguesa e validação da *Sexual Sensation Seeking Scale* para estudantes do ensino superior ... 105
3.3. Artigo 3 - Construção e validação da Escala de Atitudes de Saúde Sexual e Reprodutiva (EASSR) para estudantes do ensino superior ... 123

CAPÍTULO 4 – ESTUDOS EMPÍRICOS EM SAÚDE SEXUAL E REPRODUTIVA ... 143
4.1. Artigo1 - Knowledge of and attitudes on sexual and reproductive health among college students ... 145
4.2. Artigo 2 - Comportamentos contracetivos de estudantes do ensino superior ... 159
4.3. Artigo 3 - Risk and protective factors for college students, sexual and reproductive health ... 183
4.4. Artigo 4 - Barreiras no acesso dos jovens adultos aos cuidados de saúde sexual e reprodutiva ... 201
4.5. Artigo 5 - Uso do preservativo em estudantes do ensino superior. Aplicação do Modelo de Promoção da Saúde de Pender ... 215
4.6. Artigo 6 - *"Usar o preservativo não é questão de confiança é uma questão de saúde"* **Discussão focalizada com estudantes de ensino superior sobre comportamentos de saúde sexual e reprodutiva** ... 239

CAPÍTULO 5 – SÍNTESE INTEGRATIVA E PRINCIPAIS CONCLUSÕES ... 273

CAPÍTULO 6 – LINHAS ORIENTADORAS DE PROMOÇÃO DA SAÚDE SEXUAL E REPRODUTIVA PARA ESTUDANTES DO ENSINO SUPERIOR ... 281

REFERÊNCIAS BIBLIOGRÁFICAS ... 293

APÊNDICES ... 313

Apêndice A – Protocolo de investigação ... 315
Apêndice B – Autorização dos autores para utilização das escalas ... 329
Apêndice C – Informação transmitida aos participantes convidados a integrar os grupos focais sobre os procedimentos e condições de recolha de dados ... 333
Apêndice D – Questões orientadoras dos grupos de discussão focalizada ... 337
Apêndice E – Questionário de caracterização dos participantes dos grupos focais ... 345
Apêndice F – Autorização da Comissão Nacional de Proteção de Dados (Nº 7409/2013) ... 349
Apêndice G – Parecer da Comissão de Ética da Universidade (Parecer nº 2/2012) ... 355
Apêndice H – Consentimento informado dos participantes dos grupos focais ... 359

ÍNDICE DE TABELAS

Capítulo 2 – Metodologia Geral

Tabela 1. Estatísticas relativas à idade dos participantes 62

Tabela 2. Caracterização sociodemográfica e académica da amostra em função do género 63

Tabela 3. Valores de consistência interna de cada um dos fatores, e correlação *item total corrigidas* para a Escala de Satisfação com o Suporte Social (ESSS) 73

Tabela 4. Estrutura fatorial da Escala de Satisfação com o Suporte Social (ESSS), e valores de consistência interna por fator e para o total da escala 75

Tabela 5. Distribuição das características sociodemográficas dos participantes 82

Tabela 6. Comportamentos sexuais e reprodutivos dos participantes. 83

Tabela 7. Componentes do Modelo de Promoção da Saúde de Pender, adaptado de *Health Promotion in Nursing Pratice* (Pender et al., 2011), utilizado na análise dos grupos focais. 84

Capítulo 3 – Estudos Empíricos Metodológicos

Artigo 1

Tabela 1. Estrutura fatorial da versão portuguesa da versão reduzida da *Condom Use Self-efficacy Scale* (CUSES - RP) 97

Tabela 2. Análise da fiabilidade (valores de alfa de *Cronbach*, médias das correlações inter-itens e amplitude das correlações item-total corrigidas) para a versão reduzida portuguesa da *Condom Use Self-efficacy Scale* (CUSES - RP). 98

Tabela 3. Correlação de *Pearson* entre os diferentes fatores que compõem a versão reduzida portuguesa da *Condom Use Self-efficacy Scale* (CUSES-RP). 99

Tabela 4. Fiabilidade compósita, variância extraída média e validade discriminante da versão reduzida portuguesa da *Condom Use Self-efficacy Scale* (CUSES-RP). 100

Artigo 2

Tabela 1. Constituição dos fatores, níveis de saturação e comunalidades da *Sexual Sensation Seeking Scale* (SSSS-P). 114

Tabela 2. Resultados descritivos dos itens e correlação item total da *Sexual Sensation Seeking Scale* (SSSS-P) 115

Tabela 3. Correlação de *Pearson* entre os diferentes fatores que compõem a versão portuguesa da *Sexual Sensation Seeking Scale* (SSSS-P) 116

Tabela 4. Fiabilidade compósita, variância extraída média e validade discriminante da versão portuguesa da *Sexual Sensation Seeking Scale* (SSSS-P) 117

Artigo 3

Tabela 1. Constituição dos fatores/dimensões, níveis de saturação e consistência interna das dimensões e total da Escala de Atitudes SSR (EASSR).. 133

Tabela 2. Comparação dos resultados dos índices de qualidade de ajustamento dos cinco modelos analisados 135

Tabela 3. Fiabilidade compósita, variância extraída média e validade discriminante da Escala de Atitudes de SSR (EASSR) 136

Tabela 4. Correlação de *Pearson* entre os diferentes fatores que compõem a Escala de Atitude de SSR (EASSR)... 136

Capítulo 4 – Estudos Empíricos sobre Saúde Sexual e Reprodutiva

Artigo 1

Table 1. The influence of demographics and academic factors on sexual and reproductive health knowledge and attitudes. The results are presented as the mean (standard deviation).. 152

Table 2. The relationship between sexual and reproductive health behaviors and knowledge and attitudes. The results are presented as the mean (standard deviation).................. 154

Artigo 2

Tabela 1. Caracterização das práticas contracetivas de estudantes do ensino superior 169

Tabela 2. Influência dos fatores ambientais e individuais nas escolhas contracetivas dos estudantes do ensino superior 171

Tabela 3. Influência dos fatores cognitivos e psicossociais nas escolhas contracetivas dos estudantes do ensino superior 173

Artigo 3

Table 1. The influence of individual and environmental factors on the risk-taking behaviours of having sex without using a condom, having sex under the influence of alcohol or drugs and having had sex with casual partners over the last 12 months. Results are presented in percentage in rows pertaining to each of the risk behaviours 191

Table 2. The influence of cognitive and psychosocial factors on the risk-taking behaviours of having unprotected sex without a condom, having sex under the influence of alcohol or drugs, having had sex with casual partners over the last 12 months. Results are presented in mean (standard deviation) 193

Table 3. Logistic regression models obtained for cognitive and psychosocial factors predict risk-taking behaviours of having sex without using a condom, having sex under the influence of alcohol or drugs and having had sex with casual partners over the last 12 months 193

Artigo 5

Tabela 1. Variáveis preditoras do uso do preservativo, adaptadas do modelo HPM de Pender 223

Tabela 2. Influência de características individuais e experiências no comportamento promotor da saúde - uso do preservativo 226

Tabela 3. Influência dos constructos do HPM relacionados com o compromisso com o plano de ação e o comportamento promotor de saúde uso de preservativo 228

Tabela 4. Coeficientes do modelo final refinado para predizer a adoção do comportamento promotor de saúde de usar preservativo (variável dependente), do compromisso com o plano de ação (variável mediadora) e os constructos do HPM (variáveis independentes) 229

ÍNDICE DE FIGURAS

Capítulo 1 – Enquadramento Conceptual

Figura 1. Componentes do Modelo de Promoção da Saúde de Pender 32

Figura 2. Modelo explicativo dos fatores psicossociais que afetam os comportamentos sexuais de risco 46

Capítulo 2 – Metodologia Geral

Figura 3. Desenho geral do estudo 60

Capítulo 3 – Estudos Empíricos Metodológicos

Artigo 1

Figura 1. Pesos fatoriais, fiabilidade individual e correlações entre fatores da CUSES-RP do modelo com índices de modificação (1a) e modelo de segunda ordem da CUSES-RP (1b) 100

Artigo 2

Figura 1. Pesos fatoriais, fiabilidade individual e correlações entre fatores da SSSS-P do modelo inicial (1a) e modelo final (1b) 117

Artigo 3

Figura 1. Pesos fatoriais, fiabilidade individual e correlações entre os fatores da EASSR do modelo inicial (1a) e modelo de 2.ª ordem final (1b) 138

Capítulo 4 – Estudos Empíricos em Saúde Sexual e Reprodutiva

Artigo 5

Figura 1. Modelo de Promoção da Saúde (HPM) 222

Figura 2. Modelo final refinado da associação entre adoção do comportamento promotor de saúde de usar preservativo, do compromisso com o plano de ação e os constructos do HPM, para o género feminino 231

Figura 3. Modelo final refinado da associação entre adoção do comportamento promotor de saúde de usar preservativo, do compromisso com o plano de ação e os constructos do HPM, para o género masculino 231

Figura 4. Modelo final refinado da associação entre adoção do comportamento promotor de saúde de usar preservativo, do compromisso com o plano de ação e os constructos do HPM, para o total da amostra estudada 232

Capítulo 6 – Linhas orientadoras de promoção da saúde sexual e reprodutiva para estudantes do ensino superior

Figura 1. Síntese dos resultados do estudo relativos aos constructos do HPM de Pender, instrumentos utilizados para os avaliar e medidas propostas para melhorar o comportamento promotor de saúde dos estudantes 285

ABREVIATURAS E SIGLAS

AFC - Análise Fatorial Confirmatória

AFE - Análise Fatorial Exploratória

AMOS - *Analysis of Moment Structures*

APF - Associação para o Planeamento da Família

CAJ - Centro de Atendimento de Jovens

CDC - Centers for Disease Control and Prevention

CE - Contraceção de Emergência

CFI - *Comparative Fit Index*

CUSES - *Condom Use Self-efficacy Scale*

CUSES-R - *Condom Use Self-efficacy Scale*, versão reduzida

CUSES-RP- *Condom Use Self-efficacy Scale*, versão reduzida portuguesa

DGS - Direção-Geral da Saúde

DIP - Doença Inflamatória Pélvica

DIU - Dispositivo Intra-Uterino

EASSR - Escala de Atitudes de Saúde Sexual e Reprodutiva

ECDC - European Centre for Disease Prevention and Control

EESMO - Enfermeiro Especialista em Saúde Materna e Obstétrica

EpS - Educação para a Saúde

FC - Fiabilidade Compósita

FIPF - Federação Internacional do Planeamento Familiar

FNUAP - Fundo das Nações Unidas Para a População

GFI - *Goodness-of-fit Index*

GTES - Grupo de Trabalho de Educação Sexual

HBM - *Health Belief Model*

HBSC - Health Behaviour in School-Aged Children

HPM - *Health Promotion Model*

HPV - *Human Papiloma Virus*

IBM - *Information, Motivation, and Behavioral Skills Model*

ICEC - International Consortium for Emergency Contraception

ICSSR - Inventário de Conhecimentos em Saúde Sexual e Reprodutiva

IMC - Índice de Massa Corporal IMC - Índice de Massa Corporal

IPJ - Instituto Português da Juventude

IPPF - International Planned Parenthood Federation

IST - Infeções Sexualmente Transmissíveis

IVG - Interrupção Voluntária da Gravidez

KMO - *Kaiser-Meyer-Olkin*

KR - *Coeficiente de Kuder-Richardson*

KU - Valores de Achatamento

MCS - Modelo de Crenças da Saúde

MRSEA - *Root Mean Square Error of Approximation*

nº - número

ODM - Objetivos para o Desenvolvimento do Milénio

OE - Ordem dos Enfermeiros

OMS - Organização Mundial da Saúde

ONU - Organização das Nações Unidas

p. - página

PF - Peso Fatorial

RCN - Royal College of Nursing

RMR - *Root Mean Square Residual*

RMSEA - *Root Mean Square Error of Approximation*

SIDA - Síndrome da Imunodeficiência Adquirida

SK - Valores de Assimetria

SNS - Serviço Nacional de Saúde

SPC - Sociedade Portuguesa de Contraceção

SPG - Sociedade Portuguesa de Ginecologia

SPSS - *Statistical Package for the Social Science*

SRH - *Sexual and Reproductive Health*

SRHS - *Sexual and Reproductive Health Scale*

SRMSR - *Standardized Root Mean Square Residual*

SSR - Saúde Sexual e Reprodutiva

SSSS - Sexual Sensation Seeking Scale

SSSS-P - *Sexual Sensation Seeking Scale,* versão Portuguesa

STI - *Sexually Transmitted Infections*

TPB - Theory of Planned Behavior

TRA - *Theory of Reasoned Action*

UE - União Europeia

UNAIDS - Joint United Nations Programme on HIV/AIDS

UNESCO - United Nations Educational, Scientific and Cultural Organization

UNFPA - United Nations Population Fund

UNICEF - United Nations Children’s Fund

VEM - Variância Extraída Média

VIH - Vírus da Imunodeficiência Humana

WHO - World Health Organization

INTRODUÇÃO

As pessoas jovens representam o futuro de um país, daí a importância de assegurar que se possam tornar cidadãos saudáveis e ativos na sociedade onde se inserem (Associação para o Planeamento da Família [APF], 2010). A sexualidade é um aspeto central do ser humano ao longo da vida e engloba sexo, identidades de género e papéis, orientação sexual, erotismo, prazer, intimidade e reprodução. A forma como os jovens abordam e expressam a sua sexualidade e relacionamentos, assim como as escolhas que fazem em termos de saúde sexual e reprodutiva (SSR), têm um impacto considerável no seu futuro e nas suas vidas. A promoção da saúde dos jovens deve ter como pressuposto básico dar resposta às suas necessidades específicas, particularmente no que diz respeito a questões fundamentais como a SSR, construindo sociedades mais saudáveis e melhorando a sua qualidade de vida. Se se concordar que “a promoção da saúde juvenil pode constituir um investimento significativo em termos de ganhos de saúde no plano individual e coletivo, contribuindo para o desenvolvimento e para o bem-estar das populações” (Direção-Geral da Saúde [DGS], 2006, p.6), então investir na saúde desta população assume extrema importância.

Em Portugal, desde os anos 1970 que se tem procurado investir nas questões específicas da saúde dos jovens, iniciaram-se múltiplas experiências em promoção de saúde, em atendimento, investigação, formação, planeamento e organização de cuidados de saúde adaptados a este grupo etário (APF, 2010). A enquadrar este movimento foi criada, no âmbito da saúde reprodutiva, legislação específica para a prestação de cuidados a adolescentes e jovens. Não obstante, a melhoria significativa dos indicadores relativos à SSR, o comportamento sexual dos jovens continua a ser uma das preocupações dos serviços de saúde pública nacionais e internacionais, pela associação a consequências negativas na sua saúde e bem-estar (DGS, 2006). Acresce o facto dos estudantes do ensino superior serem habitualmente esquecidos nos diagnósticos e intervenções de saúde pública, em virtude do entendimento de que este grupo, comparativamente com os adolescentes, apresenta maior conhecimento e maior capacidade para tomar decisões relacionadas com a sexualidade (Matos, Reis, Ramiro & Equipa Aventura Social, 2011).

As universidades e outras instituições do ensino superior constituem grandes organizações em que se ensina e se aprende e onde há um forte processo de socialização, pelo que têm um grande potencial para afetar significativamente e de modo positivo as vidas e saúde dos seus membros (Rocha, 2008). As Universidades Promotoras de Saúde podem ter um papel decisivo na promoção e proteção da saúde dos estudantes e outros membros da comunidade universitária, pelo contributo que dão para a criação de ambientes favoráveis de vida e de aprendizagem. Isso significa que, para além de se focarem na divulgação do conhecimento e competências profissionais, devem promover a participação ativa dos

estudantes num processo social organizado, atendendo aos seus problemas e necessidades efetivas. Nesta perspetiva, o programa das Universidades Saudáveis pretende desenvolver nos jovens a capacidade para tomar decisões sobre a sua saúde, e procurar que o ambiente em que se desenvolvem seja favorável, contribuindo para minimizar os riscos que a maioria dos jovens enfrenta e que podem ter repercussões na sua vida atual e futura (Lara Flores, Saldaña Balmori, Férnandez Vera & Delgadillo Gutiérrez, 2015).

Na universidade os estudantes ficam, em muitos casos, mais independentes e livres da supervisão direta dos pais e pela primeira vez enfrentam maiores pressões sociais e académicas num ambiente em que se questionam sobre os valores, as crenças e objetivos com que cresceram e foram educados (Rocha, 2008). A frequência da universidade corresponde, igualmente, a uma idade em que aqueles que ainda não iniciaram a sua vida sexual provavelmente o farão. A decisão de iniciar relações sexuais acontece paralelamente a várias modificações na sua vida, sendo que a vivência da sexualidade tem implicações a nível biológico e psicossocial (Claxton & van Dulman, 2015). Embora exista a convicção de que a maioria dos comportamentos nocivos para a saúde, sejam adquiridos até à adolescência, a evidência sugere que muitos destes comportamentos são adquiridos já na idade adulta e no contexto universitário, decorrente das modificações relacionadas com fatores sociais e ambientais, que potenciam a adoção de comportamentos de risco (Precioso, 2004). Alguns dos estudos sobre os comportamentos sexuais e reprodutivos em jovens universitários portugueses referem que um número significativo mantém comportamentos sexuais de risco (Abreu, 2010; Cunha-Oliveira, Cunha-Oliveira, Pita & Massano-Cardoso, 2013; Matos et al., 2011; Oliveira, 2014; Pacheco, 2012), os quais podem ter reflexos negativos na sua SSR, pelo que um modelo de educação que vise a promoção de comportamentos preventivos ao nivel da SSR, deverá ser assegurado para além da adolescência, estendendo-se também aos jovens adultos (Matos et al., 2011).

A vulnerabilidade dos jovens nas questões de SSR relaciona-se com idade em que ocorrem as primeiras experiências sexuais, a maior suscetibilidade biológica para a infeção e as barreiras no acesso dos jovens aos serviços de saúde (Falcão Junior et al., 2009; Workowski & Bolan, 2015). Também no contexto académico, têm vindo a ser identificados comportamentos considerados de risco para a SSR dos estudantes universitários, designadamente o uso inconsistente do preservativo (Cunha-Oliveira et al., 2009; Gomes & Nunes, 2011; Kann et al., 2014; Reis, Ramiro, Matos & Diniz, 2013), relações amorosas de curta duração, prática de relações sexuais desprotegidas com parceiros ocasionais e a associação frequente com álcool e/ou drogas (Adefuye, Abiona, Balogun & Lukobo-Durrell, 2009; Burnett et al., 2014; Reis et al., 2013; Uecker, 2015; Wicki, Kuntsche & Gmel, 2010) e

ausência de uma vigilância de saúde regular (Vilar & Ferreira, 2010). Os principais relatórios de vigilância epidemiológica a nivel internacional e europeu (European Centre for Disease Prevention and Control [ECDC] & WHO Regional Office for Europe, 2014), apontam para um aumento significativo das taxas de prevalência das IST entre os jovens adultos (CDC, 2014; ECDC & WHO Regional Office for Europe, 2014) e de gravidez indesejada (WHO, 2015). Apesar da infeção por VIH/SIDA ser a mais severa, muitas outras IST, tais como a *Chlamydia*, o Vírus Papiloma Humano (HPV - da sigla inglesa *Human Papiloma Vírus*), a sífilis e a gonorreia podem testar associadas a inúmeras complicações reprodutivas, a curto e a longo prazo e, em particular, na fertilidade (Shepherd et al., 2010).

A falta de informação ou a sua obtenção por fontes pouco credíveis (Sant'Anna, Carvalho, Bastos & Coates, 2008; UNESCO, 2010), o excesso de confiança em relação à invulnerabilidade, bem como tabus sociais e familiares sobre a sexualidade podem ser responsáveis pela utilização de métodos contracetivos sem aconselhamento (APF, 2010), insegurança para solicitar ao parceiro o uso de métodos contracetivos e preventivos e a baixa adesão aos serviços de SSR (Granja, 2009; Martins, Nunes, Muñoz-Silva & Sánchez-García, 2008; Vilar, 2008). O acesso a informação de qualidade, a educação e serviços de SSR abrangentes podem levar a um aumento da prática de sexo seguro e à utilização mais responsável de contracetivos. Daqui resulta a necessidade de intervir na prevenção de comportamentos sexuais de risco e de reconhecer a sexualidade e a saúde reprodutiva como um campo de estudo e de intervenção nas universidades (Matos et al., 2011; Mello, Moysés e Moysès, 2009; Rocha, 2008).

Os comportamentos de risco e o estilo de vida são, atualmente, dois elementos determinantes na saúde dos jovens, pelo que, no âmbito da educação para a saúde (EpS), para além da informação genérica disponibilizada, deve considerar-se a implementação de intervenções que visem a promoção de conhecimento, de atitudes mais positivas e de competências pessoais e sociais mais favoráveis. Adicionalmente, deve investir-se na criação de serviços apropriados para jovens, acessíveis e integrados numa resposta de saúde e educação abrangente e de acordo com as suas necessidades. Identificar fatores de risco e de proteção da SSR dos jovens é uma estratégia importante para o desenvolvimento de intervenções eficazes para mudar comportamentos sexuais de risco (Mmari & Sabherwal, 2013) e promover uma maior consciencialização e participação dos jovens nos programas de promoção da SSR (Silva & Silva, 2011).

Um largo corpo de investigação sobre os determinantes do comportamento sexual e reprodutivo aponta para o facto de este ser influenciado por múltiplos fatores, entre os quais, fatores individuais, cognitivos (conhecimento) e psicossociais (atitudes, normas subjetivas ou sociais, autoeficácia, suporte social, disponibilidade de serviços e cuidados de saúde,

entre outros) (Kirby Coyle, Alton, Rolleri & Robin, 2011), e cuja totalidade de interações ainda não é totalmente conhecida. Esta complexidade torna-se ainda maior dado o contexto de mudança que define o desenvolvimento psicossocial dos jovens adultos integrados no ambiente académico. Estes fatores são particularmente importantes porque, contrariamente aos fatores sociodemográficos - idade, o género, o estatuto socioeconómico, grau de escolaridade ou religião - são potencialmente modificáveis e têm um forte impacto na tomada de decisão relacionada com os comportamentos sexuais seguros (Kirby et al., 2011; Lazarus et al., 2010). Contudo, o estudo dos fatores sociodemográficos revela-se particularmente útil para segmentar as populações e direcionar as intervenções em saúde (Silva & Brito, 2014).

Da preocupação em operacionalizar estratégias de promoção de comportamentos saudáveis foram surgindo diferentes modelos, dos quais destacamos o modelo de mudança de comportamentos, denominados cognitivo-comportamentais, e o Modelo de Promoção da Saúde de Pender (*Health Promotion Model* – HPM). Destacamos o primeiro pela sua importância na compreensão da mudança de comportamentos, incluindo os comportamentos de risco sexual, e o segundo por se situar dentro dos modelos de promoção da saúde que centra o seu foco de atenção na intervenção de enfermagem, através de uma aproximação aos modelos comportamentais.

A dificuldade em integrar num só modelo a ação (e interação) de todos os fatores envolvidos tem sido um dos desafios da conceptualização e investigação do comportamento sexual de risco (Kirby et al., 2011). No presente trabalho procuramos incorporar os conceitos mais relevantes das diferentes teorias e modelos que procuram compreender a mudança de comportamentos de saúde, conceitos também incluídos no HPM e outros relevantes na área da sexualidade, como a busca de sensações sexuais, uma vez que os fatores considerados nos quadros teóricos individuais podem não ser suficientes para explicar a complexidade do comportamento sexual. Vários investigadores têm incentivado o uso dos modelos de forma complementar e a inclusão de variáveis adicionais para melhor prever os comportamentos de saúde (Conner & Armitage, 1998; Glanz & Bishop, 2010). Conhecer os fatores de risco e proteção permitirá a elaboração de programas de intervenção, promovendo em simultâneo os fatores de proteção e a própria saúde dos jovens.

A promoção da saúde em geral e a EpS, em particular, representam atividades nas quais o enfermeiro assume um papel relevante, garantindo sempre a participação ativa dos cidadãos. Educar as pessoas para a saúde é criar condições para que adquiram informação e competências necessárias para elas fazerem escolhas saudáveis e modificarem os comportamentos de risco. Neste sentido, a EpS pode potenciar a mudança de comportamentos e deve ser encarada como um ato de cidadania organizada e planeada por

profissionais de saúde (Simões, Nogueira, Lopes, Santos & Peres, 2011). Esta visão requer que os profissionais de enfermagem sejam capazes de ajudar os indivíduos a alcançar o seu potencial físico, mental e social, fazendo-o dentro do contexto desafiador do ambiente em que vivem (Royal College of Nursing [RCN], 2014). Os cuidados de enfermagem são essenciais ao longo do ciclo vital dos indivíduos, com ênfase particular nos processos adaptativos, de crescimento e desenvolvimento, do qual faz parte a transição para a idade adulta e a vivência positiva da sexualidade. Compete ao enfermeiro identificar situações de saúde da população, criar e aproveitar oportunidades para promover estilos de vida saudáveis, disponibilizando informação geradora de aprendizagens e de novas capacidades e valorizar os recursos individuais (Graça, 2015; OE, 2000; Rodrigues, Pereira & Barroso, 2005). No âmbito da promoção da SSR, a cooperação e a complementaridade dos serviços e dos profissionais de saúde, bem como a harmonização de estratégias, são de extrema importância. O enfermeiro, pelo perfil de competências que detém, deve ser capaz de dar reposta às necessidades de saúde nesta área e de atuar em complementaridade com outros profissionais, visando a mudança de comportamentos de risco sexual. Neste contexto, propomo-nos estudar os determinantes da SSR em estudantes do ensino superior e contribuir para o desenvolvimento de estratégias de promoção da SSR.

Estruturalmente, o presente trabalho encontra-se organizado em seis capítulos, para além da introdução. O primeiro designado de enquadramento conceptual é constituído por quatro subcapítulos, onde procuramos fundamentar, rever e sistematizar os conceitos mais relevantes nesta área do conhecimento, que versa a SSR dos estudantes do ensino superior. Inicialmente, apresenta-se uma perspetiva global do desenvolvimento das políticas de SSR em Portugal, como reflexo das mudanças a nivel internacional, em particular no que respeita à população juvenil. Segue-se uma abordagem dos principais conceitos de promoção e EpS, dando destaque a alguns dos desafios atuais em saúde e refletindo sobre o papel das universidades como potenciais contextos de promoção da saúde, para posteriormente apresentar alguns dos modelos de promoção de saúde que visam a mudança de comportamento de risco, em particular os que se apresentam com maior relevância para a intervenção de enfermagem. De seguida faz-se uma caracterização dos comportamentos de SSR dos estudantes, para identificar alguns dos determinantes do comportamento de risco sexual relevantes na adultez emergente. Finalmente, procede-se a uma abordagem da SSR como um foco de intervenção em enfermagem.

No segundo capítulo é explicitada a metodologia geral utilizada no presente estudo, que se configura como um estudo de natureza mista, uma vez que compreende uma abordagem quantitativa e qualitativa. No estudo quantitativo, descreve-se a forma como os diferentes estudos se complementam entre si e apresentam-se as opções metodológicas: os objetivos

gerais do estudo, o desenho do estudo, a caracterização da amostra e os instrumentos de recolha de dados com as considerações sobre o seu processo de construção ou adaptação para a população portuguesa e a análise das características psicométricas e a descrição da análise estatística. No estudo qualitativo descreve-se o percurso metodológico, processo de planeamento e instrumentos de recolha de dados, e procede-se à caracterização dos participantes e dos procedimentos de análise interpretativa dos dados, finalizando com as considerações éticas inerentes ao processo de investigação.

O terceiro capítulo, estudos empíricos metodológicos, integra três dos estudos referentes à análise psicométrica dos instrumentos utilizados na investigação. Por sua vez, o quarto capítulo, designado de estudos empíricos em SSR, inclui cinco estudos de natureza quantitativa, e um último de natureza qualitativa, onde foi usado como procedimento metodológico a técnica dos grupos focais (*Focus Groups*).

Os artigos que integram este capítulo foram estruturados de forma independente mas complementar, levando em consideração não só os procedimentos adequados para a realização de um trabalho de natureza científica, mas também as normas de organização da revista onde se publicaram, ou se pretende publicar. Os três primeiros artigos que integram os estudos metodológicos são referentes ao processo de adaptação e validação para a população portuguesa da versão reduzida da Escala de Autoeficácia para Usar o Preservativo (CUSES) e da Escala de Busca de Sensações Sexuais (SSSS) e construção e validação da Escala de Atitudes de Saúde Sexual e Reprodutiva (EASSR), para estudantes do ensino superior. No capítulo referente aos estudos empíricos em SSR, no primeiro estudo analisamos a relação entre o conhecimento, as atitudes e os comportamentos sexuais e reprodutivos dos estudantes. No segundo estudo, descrevemos as práticas contracetivas dos estudantes e identificamos fatores que influenciam os comportamentos de contraceção, atendendo às questões de género. No terceiro identificamos fatores preditores dos comportamentos de risco para a SSR. No quarto estudo procedemos à caracterização dos comportamentos sexuais e reprodutivos dos estudantes e das barreiras no acesso aos serviços de SSR. No quinto estudo, procuramos identificar os fatores preditores do uso do preservativo. Finalmente, no sexto estudo, procuramos conhecer as representações e perceções dos estudantes sobre os comportamentos sexuais de risco e de proteção, tendo por base o referencial teórico o HPM de Pender.

No quinto capítulo apresenta-se uma síntese integrativa das principais conclusões, mencionando as suas implicações para a prática de enfermagem, que constituiu a base empírica para a conceção do sexto e último capítulo, onde se apontam algumas linhas de orientação para o desenvolvimento de programas de promoção da SSR para estudantes do ensino superior.

CAPÍTULO 1

ENQUADRAMENTO CONCEPTUAL

1.1. Políticas de desenvolvimento da saúde sexual e reprodutiva em Portugal

Na maioria dos países, a SSR emergiu como uma área prioritária a considerar nas políticas e programas de saúde, o que revela uma preocupação com os potenciais problemas de saúde que podem afetar, direta ou indiretamente, o bem-estar dos indivíduos e das comunidades, pois implicam elevados custos ao nível do capital humano e da produtividade (Fundo das Nações Unidas Para a População [FNUAPA], 2005). O entendimento sobre os conceitos de saúde reprodutiva e saúde sexual são relativamente recentes e têm conhecido transformações substanciais ao longo do tempo, em resultado de um conjunto de fatores socioculturais, políticos e médico-científicos, que permitiram reconhecer que os indivíduos têm o direito a desfrutar da sexualidade de uma forma livre e esclarecida. A queda generalizada da fecundidade no mundo facilitou a mudança de paradigma do planeamento familiar para os direitos sexuais e reprodutivos. Estes passam a ser considerados direitos individuais, independentemente da idade, género, orientação sexual, religião ou etnia e abrangem a sexualidade e a fecundidade, o direito à informação e o acesso aos serviços de saúde, através de uma abordagem interdisciplinar e não apenas biomédica (Vilar, 2009).

Na abordagem das políticas de SSR em Portugal, devemos considerar diferentes fases que refletiram alguns dos factos mais relevantes a nível nacional e internacional, que permitiram o desenvolvimento das políticas de saúde sexual e reprodutiva, em particular nas questões relacionadas com a juventude.

As mudanças no contexto histórico, social e cultural que atravessaram todo o século XIX e XX influenciaram claramente a saúde reprodutiva em Portugal. À problemática levantada por Malthus[1] relativa à explosão demográfica veio juntar-se, no século XIX, um conjunto de fatores sociais, tais como o desenvolvimento da sociedade industrial, a entrada da mulher no mundo do trabalho e o avanço do conhecimento médico, aumentando a esperança de vida, que vieram ao encontro de necessidades individuais. A diminuição significativa das taxas de mortalidade infantil e o aumento da esperança média de vida conduziram a um aumento populacional que criou a necessidade de implementar políticas de controlo da natalidade (Manuel, 2007).

No início do século XX, em diversos países da Europa e nos Estados Unidos da América, multiplicaram-se atividades pioneiras no domínio da maternidade livremente assumida e do controlo da natalidade "*birth control*". A partir dos anos 20, foi fundada a Liga Internacional

[1] Thomas Malthus (1766-1834), economista e pastor da igreja Anglicana, preocupado com as reservas do mundo, apresentou no seu livro "Ensaio sobre o Princípio da População" (1798), uma posição pessimista, argumentando que a população tendia sempre a crescer mais rapidamente do que os meios de subsistência, pelo que o desenvolvimento e o progresso seriam utópicos, longe do alcance da maioria da população. Malthus apresentava uma posição conservadora relativamente às políticas de natalidade, não separando a atividade sexual da reprodução. Opunha-se à utilização de métodos contracetivos e ao aborto, apontando apenas como meios aceitáveis de limitação da natalidade o aumento da idade para o casamento e o celibato (Alves, 2002). Foi Francis Place quem considerou, em 1822, que a resposta aos problemas populacionais residia no uso de métodos de contraceção artificial.

com o objetivo de difundir este movimento e promover a realização de Conferências Internacionais da População. Nos anos 30 e 40, a teoria do "*birth control*", dá lugar às ideologias neomalthusiana[2] e pronatalistas. Depois da contraceção e regulação da natalidade, chega-se, nos anos 30, ao conceito mais amplo de planeamento familiar, que antecedeu o conceito de saúde reprodutiva (Gomes, 1988). Esta designação encerra dois significados destintos, por um lado, a separação do sexo da reprodução pelo uso de métodos contracetivos e, por outro lado, um esforço no controlo do crescimento populacional, como medida de combate ao subdesenvolvimento social e económico (Manuel, 2007).

Nas décadas de 1950-1960, observou-se o maior crescimento demográfico de toda a história da humanidade, difundindo-se o medo da "explosão populacional". Os formuladores de políticas, influenciados pelo pensamento neomalthusiano, passaram a defender a disseminação de métodos contracetivos. Nas décadas seguintes, o pensamento neomalthusiano tornou-se mais moderado, na medida em que a fecundidade diminuía no mundo e, mesmo contrariando os interesses individuais, procurava-se obter uma limitação global da população. Quando as pesquisas demográficas passaram a mostrar que o número desejado de filhos, por parte das mulheres e casais, era menor que a fecundidade observada, passou a dar-se mais ênfase às necessidades não atendidas de contraceção. A introdução da contraceção hormonal oral na década de 1960 representa uma revolução, antropológica, afectiva e sexual. Pela primeira vez na história da humanidade, o prazer sexual é dissociado da reprodução, e onde a mulher pode assumir a responsabilidade por não querer engravidar. No entanto a sua aceitação é reticente não só pela população em geral como até mesmo pelos ginecologistas. Potugal era ainda um país moralmente conservador nas questões da sexualidade, o que levantou um profundo debate ético entre a classe médica e a igreja, até porque a igreja teimava em não tomar uma posição oficial face à aceitação da contraceção (Silva, 2013).

Foi neste ambiente de alto crescimento demográfico e sob a influência do pensamento neomalthusiano que ocorreram as primeiras Conferências Internacionais de População e Desenvolvimento organizadas pela Organização das Nações Unidas (ONU), que permitiram uma transformação profunda no debate populacional, ao dar prioridade às questões dos direitos humanos, bem-estar social, igualdade de géneros e, em especial, à saúde e aos direitos reprodutivos (Corrêa, Alves & Jannuzi, 2006).

Mas foi apenas na segunda metade do século XX que a saúde e o bem-estar passaram a ser considerados direitos do indivíduo, aceitando-se que um elevado número de filhos

[2] Uma diferença fundamental entre Malthus e o neomalthusianismo é que este último aceita os métodos contracetivos como forma de limitação da fecundidade.

poderia ser prejudicial para a saúde da mãe e dos recém-nascidos, o que resultou na afirmação da necessidade de espaçamento entre as gravidezes, aceitando-se os métodos contracetivos não só como medida de controlo da natalidade, mas também como forma efetiva de planear os nascimentos de acordo com os desejos de cada família. Assim, em 1952, foi fundada, na conferência de Bombaim, a Federação Internacional do Planeamento Familiar (da sigla inglesa International Planned Parenthood Federation [IPPF]), proclamando-se que o conhecimento do planeamento familiar é um direito humano fundamental (Manuel, 2007). Mas só em 1966 este direito veio a ter reconhecimento oficial na Assembleia Geral das Nações Unidas, e apenas em 1968 a Organização Mundial da Saúde (OMS) reconhece o planeamento familiar como componente fundamental dos serviços de saúde (Gomes, 1988). Apesar dos avanços realizados a nivel mundial, em Portugal, nos anos 60, o planeamento familiar era ainda considerado subversivo e semiclandestino, aceitando-se os métodos naturais propostos pela igreja católica, o coito interrompido e as irrigações vaginais, apesar de a pílula e o preservativo já serem comercializados em Portugal desde 1962, com indicações terapêuticas. A partir dos anos 70, o direito de escolha e a liberdade de decisão nos assuntos sexuais e reprodutivos tornam-se aspetos inegociáveis nas agendas políticas dos diferentes países, ganhando relevância as questões relativas à saúde integral da mulher e à SSR, incluindo o acesso à contraceção e ao aborto seguro (Corrêa et al., 2006).

O início do planeamento familiar em Portugal tem como referência a criação da APF, em 1967, que permitiu que um conjunto de profissionais de saúde, dedicados à prestação de cuidados da família, iniciasse o debate sobre a necessidade de modernização e implementação do planeamento familiar em Portugal. Mas, apenas em 1974, após a Revolução de 25 de Abril, um texto oficial do Programa de Ação do Ministério dos Assuntos Sociais, faz a primeira referência expressa à necessidade de se informar os cidadãos sobre o planeamento familiar. A elevada taxa de mortalidade infantil e o número de mortes que resultavam de complicações por aborto clandestino terão justificado as medidas que se seguiram. Em 1976, o Dr. Albino Aroso, então secretário de estado da saúde e considerado o pai do planeamento familiar em Portugal, determinou a criação de consultas de planeamento familiar em todos os centros de saúde do país, integradas nos serviços de saúde materna e infantil. Nesta perspetiva, a constituição portuguesa é uma das raras no mundo a consignar, já em 1976, o direito dos cidadãos ao planeamento familiar (artigo 67º), apontando como deveres do Estado, assegurar "o direito ao planeamento familiar, promovendo a informação e o acesso aos métodos e aos meios que o assegurem" (p.11) e de "organizar as estruturas jurídicas e técnicas que permitam o exercício de uma paternidade consciente" (p.11). O processo de implementação das consultas de

planeamento familiar foi lento e só no final dos anos 70 estas consultas estavam disponíveis nos centros de saúde. O controlo da conceção iniciou um percurso mais consistente a partir de 1974 e, apesar das assimetrias existentes, a história da contraceção em Portugal revela algum sucesso. No início da década de 1980, encontrava-se em funcionamento cerca de 400 consultas de planeamento familiar nos centros de saúde e hospitais, com os cuidados a serem prestados por enfermeiros, no âmbito das consultas com o médico de família, ou ainda em consultas especializadas. Apesar dos avanços legislativos, o planeamento familiar continuava a ser desconhecido para uma grande parte da população, e as políticas de saúde reprodutiva centraram-se quase exclusivamente na promoção do acesso à contraceção e educação contracetiva das mulheres (Vilar, 2009).

A década de 1980 foi o momento de viragem nas questões específicas da saúde dos adolescentes e jovens. Iniciaram-se múltiplas experiências de promoção de saúde, atendimento, investigação, formação e planeamento e organização de cuidados de saúde reprodutiva adaptados a este grupo etário. Em termos legislativos, foram marcos essenciais na afirmação da SSR dos jovens em Portugal, a lei nº 3/84, de 24 de março, que estabelece o direito e à educação sexual e define as formas de acesso ao planeamento familiar, a lei nº 4/84, de 5 de abril, que regula a proteção e defesa da maternidade e paternidade, a lei nº 6/84, de 11 de maio, que estabelece a exclusão de ilicitude de alguns casos de interrupção voluntária da gravidez e a portaria nº 52/85, de 26 de janeiro, que regula o funcionamento das consultas de planeamento familiar e centros de atendimento a jovens. Estas leis vieram garantir o desenvolvimento das consultas de planeamento familiar e dos Centros de Atendimento de Jovens (CAJ), assegurando o acesso sem restrições às consultas de planeamento familiar a todos os indivíduos e, em particular, a todos os jovens em idade fértil.

Foi também no início dos anos 80 do século XX que a OMS começou a utilizar o conceito de saúde sexual, transpondo o tradicional conceito de saúde para a área da vivência da sexualidade e integrando um conjunto de preocupações e de projetos aí centrados (Vilar, 1986). Na construção destes novos conceitos, a pandemia do VIH/SIDA teve um papel importante, uma vez que houve necessidade de equacionar a saúde reprodutiva não apenas centrado na saúde materna, mas também nas questões da saúde sexual, com ênfase particular na prevenção do VIH/SIDA e nos comportamentos sexuais de risco. O combate à infeção pelo VIH/SIDA foi assumido como um objetivo prioritário, implementando-se inúmeras campanhas de prevenção da infeção e promoção do uso do preservativo. Esta nova abordagem permitiu a definição de políticas públicas de saúde focadas na diversidade de comportamentos sexuais (Vilar, 2003). Os comportamentos sexuais passaram a ser considerados uma questão de urgência social e epidemiológica, como um fator que pode

influenciar diretamente a saúde dos indivíduos e determinar as políticas de saúde pública (Ferreira & Cabral, 2010; Nodin, 2001).

A década de 1980 centra-se, sobretudo, na generalização do acesso à contraceção, nos debates sobre a sexualidade juvenil e das políticas de educação sexual, tendo como dinamizadores o parlamento e as organizações não-governamentais que se posicionam de forma mais liberal ou mais conservadora sobre as políticas relacionadas com a sexualidade juvenil. Por sua vez, o Ministério da Educação começa a ter um papel central na educação sexual, promovendo, no contexto da EpS, ações e programas de educação sexual (Vilar, 2009). Embora com dezenas de anos de atraso em relação à legislação dos restantes países europeus, Portugal passa a dispor de instrumentos legais para que a parentalidade não seja fruto do acaso, mas uma escolha livre, consciente e responsável (Gomes, 1988).

Nos finais da década de 1990, deu-se a fase de institucionalização com a convergência de três processos: a organização do Serviço Nacional de Saúde (SNS), os debates parlamentares sobre planeamento familiar, educação sexual e aborto e o debate promovido pela APF sobre o acesso dos jovens à contraceção (Vilar, 2009). Dois acontecimentos a nível internacional tiveram reflexos significativos na evolução das políticas de SSR neste período: a aprovação da Carta dos Direitos Sexuais e Reprodutivos pela *Internacional Planned Parenthood Federation* (IPPF) e a consagração dos direitos reprodutivos com delineamento de políticas de saúde a nível mundial, resultado da Conferência Internacional sobre População e Desenvolvimento, realizada no Cairo em 1994 e, posteriormente, a Conferência Internacional Sobre a Mulher, realizada em Pequim, em 1995. A Conferência do Cairo foi um dos marcos mais significativos na redefinição de políticas de SSR, ao denunciar práticas coercivas e ao iniciar a discussões sobre temas polémicos, como o aborto e a esterilização forçada, e ao considerar a relação entre população e desenvolvimento. Observou-se, desde então, uma mudança na abordagem nos temas da demografia, com as perspetivas de controlo populacional a perder força em favor do planeamento familiar, pautado pelos direitos reprodutivos, como uma das ferramentas fundamentais de promoção da SSR, suportados por políticas sociais e económicas para reforçar os direitos das mulheres (Santos, Leão, Araújo & Ferreira, 2011, citando a ONU, 2004). O Programa de Ação do Cairo apontou, ainda, para a necessidade de se abordar a SSR dos jovens de uma forma integral, centrada na pessoa, com o objetivo de reduzir a gravidez adolescente e os comportamentos de riscos associados às infeções sexualmente transmissíveis (IST) e de promover a igualdade de género na definição de políticas de SSR.

Foi no decurso destas conferências internacionais que o conceito de saúde reprodutiva (SR), foi ampliado e redefinido como sendo

> … um estado de completo bem-estar físico, mental e social, e não apenas a ausência de doença ou incapacidade, em tudo o que respeita ao sistema reprodutivo e às suas funções e processos. A saúde reprodutiva implica, por consequência, que as pessoas possam ter uma vida sexual satisfatória em segurança e que tenham a capacidade de se reproduzir e decidir livremente sobre se, quando e com que frequência o querem fazer. Esta última condição pressupõe o direito de homens e mulheres serem informados e terem acesso a métodos de planeamento familiar de sua escolha, que sejam seguros, eficazes e aceitáveis, assim como a outros métodos que escolham de regulação da fecundidade que não sejam contra a lei e ainda o direito de acesso a serviços de saúde adequados, que permitam às mulheres terem uma gravidez e um parto em segurança e proporcionem aos casais as melhores oportunidades de terem crianças saudáveis. Em conformidade com esta definição, os cuidados de Saúde Reprodutiva são considerados como a constelação de métodos, técnicas e serviços que contribuem para a saúde reprodutiva e o bem-estar, pela prevenção e resolução dos problemas de Saúde Reprodutiva. Também incluem a saúde sexual, cujo propósito é a melhoria da vida e das relações interpessoais, e não o mero aconselhamento e os cuidados relacionados com a procriação e as infeções sexualmente transmissíveis. (ONU, 1995, par. 7.2)

Desta forma, a saúde reprodutiva constitui-se como parte integrante do conceito universal de saúde da OMS, e implícito na sua definição está o direito dos indivíduos à informação, o acesso aos métodos contracetivos e ao planeamento familiar eficazes, seguros e financeiramente compatíveis com a sua situação económica.

Mais tarde, resultado de um Convénio Internacional de Saúde Sexual, promovido pela OMS em 2002 surge a contribuição para uma definição autónoma da saúde sexual (WHO, 2006), mais abrangente e positiva. Esta definição reforça a noção que a saúde sexual deve pressupor, tanto quanto possível, a existência de "experiências prazerosas e sexo seguro, livre de coerção, discriminação e violência. Para se alcançar e manter a saúde sexual, os direitos sexuais de todas as pessoas devem ser respeitados, protegidos e satisfeitos" (WHO, 2006, p.5), não se restringindo apenas à prevenção de disfunções ou IST. O conceito de saúde sexual é parte integrante do conceito de saúde reprodutiva, que visa potenciar a vida, as relações pessoais e o bem-estar geral, dado que, nas sociedades contemporâneas, a sexualidade não está limitada à reprodução (WHO, 2011). A necessidade de redefinição independente destes dois conceitos - saúde sexual e saúde reprodutiva - justifica-se em virtude dos desafios que ambas as áreas colocam no domínio da saúde pública, nomeadamente a prevenção da gravidez na adolescência e das IST. Desta forma, os conceitos de saúde sexual e de saúde reprodutiva tornam-se mais abrangentes, ultrapassando a dimensão médica e preventiva a que tradicionalmente as questões de saúde aparecem associadas. Passam a considerar os comportamentos individuais, as relações de intimidade, a proteção da saúde física e mental, os direitos individuais e sociais e o direito à informação e acesso aos serviços e cuidados de saúde (Vilar, 2009). No mesmo sentido, o conceito de SSR é também amplamente utilizado, uma vez que os dois conceitos se complementam numa abordagem integrada da sexualidade humana (Nodin, 2001).

Outras iniciativas de dimensão mundial reforçaram a necessidade de manter o desenvolvimento de políticas de promoção da SSR, nomeadamente através de programas mais abrangentes englobando a saúde, a educação bem como a política, o setor económico, assim como o enquadramento jurídico. Dando continuidade à Conferência do Cairo, quatro anos depois, numa sessão especial da Assembleia Geral das Nações Unidas (CIPID +5, Nova Iorque, 1999), uma das metas definidas foi a promoção do acesso universal à saúde reprodutiva até 2015. A nível internacional destacam-se, ainda, por terem reflexos na saúde reprodutiva, os Objetivos para o Desenvolvimento do Milénio (ODM), que foram apresentados em 2000 pela ONU e subscritos por 189 estados membros, incluindo Portugal, assumindo o compromisso de dar cumprimento a oito grandes objetivos, visando o desenvolvimento humano a nível global e onde a SSR desempenha um papel fundamental. Entre os ODM a atingir até 2015 encontrava-se a redução da mortalidade infantil (ODM 4) e a melhoria da saúde materna (ODM 5). No quadro deste último objetivo:

> ...os países comprometem-se a investir: 1) na diminuição da mortalidade materna, melhorando o acesso aos cuidados pré-natais e aumentando a proporção de partos assistidos por profissionais de saúde; 2) na diminuição da taxa de gravidez na adolescência; 3) no aumento da prevalência contraceptiva, reduzindo a proporção de mulheres sem contracepção, ou necessidades contraceptivas não satisfeitas. (DGS, 2010, p.1)

Beneficiando da conjuntura internacional, no final dos anos 90 é aprovada em Portugal a lei nº 120/99, de 11 de agosto, que vem a ser regulamentada pelo decreto-lei nº 259/2000, de 17 de outubro, integrando já o novo conceito de SSR, que veio reforçar as garantias do direito dos indivíduos à saúde reprodutiva, abordando temas como a promoção da educação sexual e a saúde reprodutiva, a prevenção de IST, métodos contracetivos, planeamento familiar e interrupção voluntária da gravidez (IVG). A lei nº 12/2001, de 29 de maio, vem, deste modo, regulamentar a distribuição gratuita de contracetivos, incluindo a contraceção de emergência, em todas as unidades de saúde da rede de cuidados primários e hospitalares, autorizando a sua venda sem necessidade de receita médica, e a lei nº 90/2001, de 20 de agosto, define as medidas de apoio às mães e pais estudantes (Vilar, 2008).

Entre 2000 e 2002, assiste-se a um desenvolvimento da educação sexual nas escolas, impulsionado pelo decreto-lei nº 259/2000, de 17 de outubro, que estabeleceu que cada escola deveria integrar a educação sexual no seu projeto educativo. Esta norma veio contemplar a implementação de programas de promoção da educação sexual na escola, adequados aos diferentes grupos etários, com envolvimento da comunidade educativa, serviços de saúde da área, associações de estudantes e de pais/encarregados de educação. Preconiza, ainda, a prevenção das IST, considerando a importância da utilização

do preservativo na prevenção das mesmas e a disponibilização do preservativo através de meios mecânicos a todos os estabelecimentos de ensino secundário e superior, por decisão dos conselhos diretivos, ouvidas as associações de pais e estudantes. O decreto-lei nº 259/2000, de 17 de outubro, prevê também o acesso dos jovens a consultas de planeamento familiar, quer nos centros de saúde, quer a nível hospitalar, mesmo que fora da área de residência, bem como a criação de consultas de planeamento familiar integradas nos serviços de saúde, nos estabelecimentos de ensino superior, com o objetivo de aconselhar e dar apoio técnico na utilização dos métodos contracetivos.

A publicação do documento “Educação Sexual em Meio Escolar - Linhas Orientadoras”, editado conjuntamente pelos Ministérios da Educação e da Saúde, foi também crucial, pois apresentou, pela primeira vez, os valores essenciais que deviam orientar a educação sexual escolar em termos de política educativa, bem como os seus objetivos, domínios do conhecimento, das atitudes e das competências, potenciando a efetiva dinamização da educação sexual em meio escolar (Ramiro, Reis, Matos, Diniz & Simões, 2011). Ainda no âmbito da educação sexual, seguiram-se outros os marcos legislativos importantes, como foi a publicação do despacho nº 19737/2005, de 13 de setembro, que determinou a criação de uma comissão independente (Grupo de Trabalho em Educação Sexual - GTES) encarregada de avaliar os conteúdos dos programas de educação sexual e propor os parâmetros gerais de educação sexual em meio escolar, numa perspetiva de promoção da saúde dos jovens. No seu relatório final, apresentado em 2007, este grupo de trabalho recomendou que a abordagem da saúde sexual considerasse o entendimento da sexualidade como uma das componentes da pessoa, no contexto de um projeto de vida que engloba valores e uma dimensão ética, a compreensão dos aspetos relacionados com as principais IST (incluindo o VIH/SIDA), a maternidade na adolescência, a IVG e o uso de métodos contracetivos, em particular o preservativo para a prevenção da gravidez e das IST (Ramiro et al., 2011). Porém, só com a lei nº 60/2009, de 6 de agosto, a educação sexual nas escolas foi tornada obrigatória, ao estabelecer o regime da educação sexual em meio escolar, em todos os níveis de ensino, com exceção do ensino pré-escolar. A portaria nº 196-A/2010, de 4 de abril, procede à sua regulamentação, estabelecendo a obrigatoriedade de educação sexual em todas as instituições de ensino básico e secundário, público e privado, a nível nacional, numa aceção verdadeiramente democrática (Matos, Reis, Ramiro, Pais-Ribeiro & Leal, 2014). A lei prevê, ainda, que a educação sexual tenha o acompanhamento de profissionais de saúde e a possibilidade de serem estabelecidas parceria com entidades credenciadas na área da EpS/educação sexual para o desenvolvimento de projetos específicos (lei nº 60/2009, de 6 de agosto), nomeadamente através das unidades de saúde pública locais. Um estudo de Matos et al. (2014) que

procurou avaliar o processo de implementação da educação sexual em meio escolar, revelou que a implementação da legislação está a realizar-se de uma forma boa/muito boa; no entanto, regista-se um défice na uniformização de procedimentos na implementação da lei e dirigentes e professores sublinham o enorme esforço que tem sido feito para cumprir as orientações constantes da lei, questionando a capacidade das escolas para dar continuidade a esse processo em circunstâncias em que, politicamente, há fortes limitações orçamentais. O processo de implementação da educação sexual nas escolas deve ser continuamente monitorizado, uma vez que é reconhecida a sua importância, pois a educação sexual é fundamental no controlo da fertilidade e na prevenção das IST, com consequências positivas na sexualidade, gravidez, infertilidade, vigilância pré-concepcional e pré-natal, qualidade e sobrevivência das crianças (DGS, 2008). As dificuldades na implementação da educação sexual nas escolas já começam a ser visíveis, uma vez que, em 2015, apenas 67,4% dos jovens refere ter acesso a educação sexual/informação sobre contraceção e prevenção de IST (Matos, Simões, Camacho, Reis & Equipa Aventura Social, 2015).

Mais recentemente, realidades emergentes como, por exemplo, a democratização do uso da internet e as redes sociais, levantam novos desafios e preocupações nesta área. As novas tecnologias de informação criam um ambiente extremamente apelativo e com novas oportunidades de vivência da sexualidade, mas também com novos riscos que não devem ser negligenciados (APF, 2015).

Em síntese, o desenvolvimento de políticas de SSR em Portugal foi marcado, na segunda metade do século XX, por algum sucesso, decorrente dos intensos debates morais e políticos: a promoção da contraceção e planeamento familiar nas décadas de 1960 e 1970, a legislação sobre sexualidade juvenil e o acesso dos jovens às consultas de planeamento familiar, nos anos de 1980 e seguintes, a formalização da contraceção de emergência e o aborto legal e seguro em circunstâncias específicas, que percorre todo este período e, mais recentemente, a implementação da educação sexual nas escolas. Nesta perspetiva, Romoaldo (2001), afirma que todo o quadro legislativo que surgiu nas décadas de 1980 e 1990 foi de extrema importância para o desenvolvimento das políticas de SSR em Portugal, permitindo que, no início deste século, estivessem reunidas as condições para uma efetiva aplicação das orientações constantes da legislação. Diversas questões de SSR, como a sexualidade juvenil, a educação sexual, a contraceção e a IVG a pedido da mulher, foram alvo de novos enquadramentos legais e novas políticas no contexto da educação, juventude e saúde, e foi neste confronto ideológico e político que se foram produzindo e implementando, ou não, as políticas de SSR. Os resultados alcançados nestas quatro décadas são claramente positivos por via dos direitos alcançados, pelos ganhos de saúde,

autonomia e de empoderamento pessoal das mulheres, sobretudo através do acesso à contraceção e às consultas de planeamento familiar como instrumento de controlo da fecundidade e, por conseguinte, de controlo do próprio percurso de vida (Vilar, 2009).

É incontestável que, nos últimos anos, Portugal registou melhorias significativas dos indicadores relativos à SSR, nomeadamente no aumento do uso dos métodos contracetivos, na diminuição da gravidez na adolescência e na melhoria dos indicadores de saúde materna e infantil. Contudo, as modificações sociais que se têm registado nos últimos anos, a par das dificuldades económicas e da necessidade de contenção das despesas no SNS, colocam desafios acrescidos que devem ser equacionados de forma a não comprometer os ganhos em saúde já alcançados (DGS, 2010). São as leis e políticas em vigor que protegem os indivíduos mais vulneráveis, como os jovens, garantindo o direito à informação e serviços de saúde, e que são capazes de garantir cuidados e serviços de saúde de qualidade, acessíveis, de uma forma equitativa a todos os cidadãos. Pelo exposto, é nosso entendimento que a evolução das políticas de SSR só será possível se existir a crença fundamental na sociedade de que a SSR é parte integrante da saúde e bem-estar e um direito de todos os indivíduos (WHO, 2011).

1.2. Promoção da saúde sexual e reprodutiva

1.2.1. Promoção da saúde

O conceito de saúde tem evoluído aos longos dos tempos, estando definido desde 1948 pela OMS, como sendo um estado de "completo bem-estar físico, mental e social e não a mera ausência de doença" (WHO, 1948). Este conceito caracteriza-se por uma grande amplitude, incluindo a componente física, mental e social, e positividade - a doença é o aspeto negativo da saúde. Esta nova abordagem conceptual reflete uma mudança de paradigma, que reformula a tradicional posição do modelo biomédico e concebe a saúde como um complexo equilíbrio dinâmico entre múltiplos fatores (Rodrigues et al., 2005). Os mesmos autores consideram que

> Ao descentrar a sua atenção na doença e patologia e ao focar o indivíduo na sua dimensão total e integradora, introduz-se uma reflexão crítica de natureza epistemológica, abrindo caminho de novas perspectivas de abordagem, passando do primado das drogas e tratamentos para a prioridade da prevenção da doença e da promoção da saúde. (p.12)

Na conferência internacional sobre cuidados de saúde primários (OMS, 1978), na qual se tornou pública a Declaração de Alma Ata**,** a saúde foi considerada não uma finalidade em si mesma mas "um recurso que deve estar ao alcance de todos para o desenvolvimento progressivo das comunidades". As atuais políticas de saúde têm também subjacente aos

cuidados de saúde o conceito de promoção da saúde, adotado pela Carta de Ottawa (OMS, 1986), onde se afirma que a promoção da saúde é o

> …processo que assegura às pessoas os meios para terem maior controlo sobre o seu nível de saúde e serem capazes de o melhorar. Para alcançar um completo bem-estar físico, mental e social o indivíduo ou o grupo deve ser capaz de determinar e atingir as suas próprias aspirações, satisfazer as suas necessidades e influenciar o ambiente. A saúde é considerada como um " recurso para a vida, não um objetivo de vida". (p.2)

Na promoção da saúde devem ser usadas diferentes estratégias para desenvolver as capacidades individuais, alterar as circunstâncias sociais e o comportamento de saúde das pessoas (OE, 2000). Na formulação de estratégias, são considerados três elementos fundamentais: estilo de vida, ambiente e participação a nível político e profissional. Por sua vez, as estratégias incluem políticas de saúde, ambiente sustentável, (re)organização dos serviços de saúde, competências pessoais e sociais, construção de parcerias e participação comunitária (Loureiro & Miranda, 2010). Os seus valores principais são a democracia, a sustentabilidade, a equidade e a capacitação (*self-empowerment*), responsabilizando os indivíduos e grupos pela promoção de atitudes e comportamentos saudáveis e pela satisfação das suas próprias necessidades. Os programas de EpS, influenciados pela teoria do *empowerment*[3], promovem os estilos de vida saudáveis e a prevenção da doença, numa perspetiva de capacitação e de aprendizagem, dando poder e intervenção aos indivíduos envolvidos (Rodrigues et al., 2005). Torna-se, por isso, essencial garantir que todos tenham condições facilitadoras de acesso aos cuidados de saúde, à informação ou a ambientes que os capacitem para vidas mais saudáveis (OMS, 2006). Uma abordagem centrada na promoção da saúde, altera a perceção do conceito de saúde e realça a importância da informação e das decisões políticas alicerçadas na realidade local e necessidades individuais (Loureiro & Miranda, 2010).

Diversas conferências internacionais vieram reafirmar a pertinência e o sentido das estratégias postuladas pela Carta de Ottawa (1986), e que permitiram continuar o debate em torno dos marcos conceptuais da promoção da saúde, EpS, participação comunitária, ambientes saudáveis, programas de ação que foram acompanhando as transformações sociais e o pensamento axiológico em torno do conceito de saúde, educação e existência humana. De uma forma geral, as políticas governamentais dos países, incluindo Portugal, têm sido norteadas pelos valores e princípios decorrentes destas conferências.

Em 2000, a Assembleia Geral das Nações Unidas adoptou a Declaração do Milénio, assumindo a corresponsabilidade dos Estados na defesa dos princípios da dignidade

[3] O conceito de *empowerment* na promoção da saúde é definido como a educação sobre problemas de saúde, colocando os interesses e as necessidades dos aprendentes no centro do processo, como sujeitos ativos e participantes em todas as fases (Rodrigues et al., 2005, p.96)

humana, igualdade e equidade, centrando a sua atenção nas populações mais vulneráveis, em particular as crianças e os jovens (Loureiro & Miranda, 2010). Visando complementar os valores, os princípios e estratégias de intervenção estabelecidas na Carta de Otawa, a Carta de Bangkok (OMS, 2005) reiterou a importância da promoção da saúde num mundo globalizado. Na promoção da saúde, surge a noção da "saúde como um recurso" e um "empreendimento coletivo". Torna-se fundamental o estabelecimento de parcerias funcionais para a promoção da saúde, que incluam o setor público e privado e diversos grupos da sociedade civil, para além daqueles já tradicionalmente envolvidos na intervenção em saúde, de modo a criar massa crítica para a promoção da saúde em diferentes locais (Administração Regional de Saúde [ARS], 2015). Deste modo, reconhece-se a importância dos determinantes culturais, dos estilos de vida saudáveis, das redes de cidades saudáveis e das escolas promotoras de saúde. Torna-se, portanto, essencial garantir que todos tenham condições facilitadoras de acesso aos cuidados de saúde, à informação, ou a ambientes que capacitem os indivíduos para uma vida mais saudável (OMS, 2006). Assim, do entendimento de que a saúde é criada e vivida pelas populações em todos os contextos da vida quotidiana - nos locais onde se aprende, se trabalha, se brinca e se cresce (Carta de Otawa, OMS, 1986), também as instituições do ensino superior podem ser excelentes contextos de promoção da saúde, com melhorias do bem-estar da população universitária.

A primeira conferência internacional sobre a promoção de saúde nas universidades foi organizada, em 1996, pela Universidade de Lancaster (Reino Unido) em colaboração com a Agência Regional para a Europa da OMS (Tsouros, Dowding, Thompson & Dooris, 1998). Seguiram-se diversos congressos internacionais[4], que foram reiterando a importância das universidades enquanto contextos promotores da saúde no âmbito do movimento das Universidades Saudáveis. O número de instituições de ensino superior que aderiu ao movimento das Universidades Promotoras de Saúde não tem parado de crescer, criando redes de trabalho nacionais e internacionais, entendidas como um mecanismo potenciador do comprometimento com este projeto e promovendo a solidariedade e apoio mútuo (Tsouros et al., 1998). Independentemente das diferentes posições estabelecidas ao longo destes encontros e assumidas por diferentes organizações, é consensual que uma universidade saudável considere a promoção da saúde como seu eixo principal e deve

[4] **2003** - Foi realizado o I Congresso na América Latina (Universidade Pontifícia Católica do Chile, em colaboração com a Universidade de Alberta), onde foi instituído o programa Universidades promotoras de saúde.
2005 - Foi realizado o II Congresso, de Universidades promotoras de Saúde das Américas, onde se definiu um conjunto de orientações que integram a Carta de Edmonton.
2006 - Foi publicado o *Guía para Universidades Saludables y otras Instituciones de Educación Superior* (Lange & Vio, 2006), pela Universidade Pontifícia Católica do Chile, conjuntamente com a Organização Pan-Americana da Saúde (OPS) e a OMS. Este documento estabelece as diretrizes para a implementação de atividades de promoção da saúde nas Universidades dentro do movimento universidade saudável.
2007- Foi realizado o III Congresso Internacional das Universidades Promotoras de Saúde, na Universidade Autónoma da Cidade Juárez Chihuahua, onde se propõe a criação da Rede Iberoamericana de Universidades Promotoras de Saúde.
2009 - Foi realizado o IV Congresso na Universidade Pública de Navarra em Espanha.

melhorar o estilo de vida dos indivíduos, capacitando-os para uma vivência saudável, não só no presente mas também como futuros cidadãos (Silva & Brito, 2014). Algumas instituições de ensino superior portuguesas têm tentado aderir a este movimento, apresentando um conjunto diverso de iniciativas que visam contribuir para a promoção da saúde e o bem-estar da comunidade académica, procurando desenvolver uma rede de trabalho nacional e de colaborar ativamente com a congénere europeia que integram (Soares, Pereira & Canavarro, 2015).

De acordo com Mello, Moysés e Moysès (2009), as universidades têm um amplo potencial para proteger a saúde e promover o bem-estar dos estudantes, em três grandes áreas: i) criando ambientes de trabalho, aprendizagens e vivências mais saudáveis; ii) ampliando a importância da saúde, da promoção da saúde e da saúde pública no ensino e na investigação; iii) desenvolvendo parcerias para a promoção da saúde e atuação comunitária. Neste sentido, nas universidades não se pode apenas ensinar, é necessário desenvolver o espírito crítico perante as situações, implantar mudanças construtivas, promovendo o desenvolvimento integral dos jovens, nomeadamente ao nível do seu bem-estar e saúde, incluindo a saúde sexual. No contexto do desenvolvimento sustentável e do aumento significativo dos comportamentos de risco para a saúde dos estudantes do ensino superior, que tem vindo a ser reportado em alguns estudos (Matos et al., 2011; Pedroso & Brito, 2014), reafirma-se a importância de envolver as universidades em projetos que visam a integração da saúde na sua missão, cultura, estruturas e modos de funcionamento, que se configuram como Universidades Promotoras de Saúde (Rocha, 2008).

Uma Universidade Promotora de Saúde promove a responsabilidade individual e comunitária, apoia o desenvolvimento de estilos de vida saudáveis, auxilia os jovens a atingir o seu potencial físico, psicológico e social, fornecendo aos estudantes o conhecimento e as competências necessárias para tomar decisões saudáveis acerca da sua saúde. Esta cultura favorecedora da saúde e bem-estar dentro das instituições deve estar clara nos planos estratégicos das instituições do ensino superior e ser sustentável no tempo (Silva & Brito, 2014).

1.2.2. Promoção e educação para a saúde

A melhoria dos determinantes da saúde depende, em grande parte, de boas políticas de educação e do nível educacional dos indivíduos e das comunidades (Loureiro & Miranda, 2010), por isso, a EpS tem vindo a ser reconhecida como parte integrante dos esforços para prevenção da doença e promoção da saúde. De facto, no novo paradigma de saúde preconiza-se a utilização do processo educação-aprendizagem, envolvendo e capacitando

os indivíduos nos processos de planificação e tomada de decisão, com vista a alcançar "saúde para todos" (Rodrigues et al., 2005).

Das diferentes conceptualizações de EpS, um dos conceitos com melhor aceitação é o de Tones e Tilford (1994):

> A educação para a saúde é toda a atividade intencional conducente a aprendizagens relacionadas com saúde e doença..., produzindo mudanças no conhecimento e compreensão e nas formas de pensar. Pode influenciar ou clarificar valores, pode proporcionar mudanças de convicções e atitudes; pode facilitar a aquisição de competências; pode ainda conduzir a mudanças de comportamentos e de estilos de vida. (p.11)

Rodrigues et al. (2005) sublinham como foco da EpS as atitudes e os comportamentos de saúde, o que pressupõe que, para além da consciência individual sobre a importância da saúde e do conhecimento em saúde, será necessário uma mudança das atitudes, das crenças e dos comportamentos em relação aos problemas de saúde. Neste sentido, a EpS deve centrar-se nas disposições e capacidades individuais e grupais, oferecendo conhecimento, influenciando modos de pensar, clarificando valores, mudando atitudes e crenças, facilitando a aquisição de competências e conduzindo a mudanças comportamentais. A EpS equacionada deste modo, apresenta-se como um processo algo complexo, exigindo profissionais qualificados, capazes de adotar posições transdisciplinares na conceção e implementação de intervenções educativas que facilitem o processo de tomada de decisão e de mudança comportamental. Os enfermeiros, reconhecidos legalmente como "a comunidade profissional e científica de maior relevância no funcionamento do sistema de saúde e na garantia do acesso a cuidados de saúde de qualidade…" (decreto-lei nº 104/98, de 21 de abril), exercem a sua atividade em diferentes contextos em complementaridade com outros profissionais de saúde e educação, sendo os elementos que melhor conhecem as pessoas, encontrando-se numa situação privilegiada para promover comportamentos de saúde e estilos de vida saudáveis.

No processo de definição de políticas de EpS é essencial o estudo de comportamentos de saúde e os fatores que os influenciam e que podem ser vistos como resultado da interação de fatores biológicos, psicológicos e sociais e a forma como influenciam a saúde (Rodrigues et al., 2005). Quando se abordam questões relacionadas com comportamentos de saúde, aparece estreitamente ligado o conceito de "risco", que está quase sempre presente na comunicação em saúde, considerando as condições onde se encontram os indivíduos perante determinadas ameaças à sua saúde. Atualmente, as principais causas de morbilidade e mortalidade dos jovens decorrem de comportamentos de risco que prejudicam a saúde, pelo que a promoção da saúde nesta faixa etária deve privilegiar o ensino e a orientação, a fim de evitar atividades de risco e comportamentos prejudiciais à saúde (DGS,

2006). A EpS dá aos jovens uma oportunidade para adotarem estilos de vida saudáveis, capazes de lhes trazer benefícios ao longo de toda a sua vida (Conselho de Enfermagem [CE], 2009). Informar sobre riscos é um processo que exige conhecimentos científicos para ajudar a equacionar a melhor forma de produção e difusão das mensagens e gerar diferentes respostas (aceitação, interesse, medo, negação). Muitas destas respostas em relação à perceção de risco e formas de responder às mensagens são explicadas através de modelos e teorias de saúde, que serão abordadas posteriormente no presente trabalho.

A EpS constitui-se como um instrumento de promoção da saúde e um recurso importante para os profissionais de saúde em diferentes contextos de intervenção (Graça, 2015), que deve ser considerado também na educação sexual de jovens adultos no contexto universitário, tal como preconizado no documento de Orientação Técnica Internacional sobre Educação em Sexualidade, desenvolvido pela UNESCO (2010). No documento anterior preconiza-se não só uma abordagem positiva da sexualidade, mas também que educação para a sexualidade deva aumentar o conhecimento e a compreensão sobre a sexualidade, explicar e esclarecer sentimentos, valores e atitudes, desenvolver ou reforçar as competências e promover e manter comportamentos de baixo risco. Para concretizar estes objetivos, este organismo estabeleceu seis conceitos-chave para os programas e projetos de educação para a sexualidade:" relacionamentos; valores, atitudes e competências; cultura, sociedade e direitos humanos; desenvolvimento humano; comportamento sexual e de SSR" (UNESCO, 2010, p.7).

1.2.3. Modelos de mudança de comportamento

Têm sido usados muitos modelos teóricos para prever a mudança dos comportamentos relacionados com a saúde, e devem ser entendidos como referenciais conceptuais no delineamento de programas de intervenção para promoção de comportamentos de saúde. A utilização de modelos e teorias no campo da promoção da saúde pode facilitar na compreensão dos determinantes dos problemas de saúde e orientar nas soluções que respondem às necessidades e interesses dos indivíduos (Glanz & Bishop, 2010; Vitor, Lopes & Ximenes, 2005). Os profissionais de enfermagem devem fundamentar as suas intervenções em pressupostos teóricos, uma vez que as teorias podem orientar a prática clínica e permitir uma visão mais abrangente dos fenómenos e da reflexão sobre os mesmos (Rodrigues et al., 2005).

A impossibilidade de controlar as consequências dos comportamentos sexuais de risco, como as IST e o VIH/SIDA, tem contribuído para que diversos organismos tenham investido na informação e ações preventivas de forma a persuadir os indivíduos a adotar

comportamentos sexuais seguros, sobretudo os indivíduos jovens, dado que é nesta idade se adquirem a grande maioria das infeções No entanto, frequentemente, os resultados das investigações desenvolvidas evidenciaram que a educação, exclusivamente, não é suficiente para operar a mudança de comportamento (UNESCO, 2010). De acordo com Oliveira (2014), apesar de existir muita formação na área da sexualidade, a adesão aos dos jovens aos programas educativos é baixa, provavelmente resultado de ações de formação pouco estruturadas, teoricamente arbitrárias e sem avaliação de resultados.

O facto de considerar a mudança comportamental como o meio mais efetivo no controlo das IST levou ao desenvolvimento de vários modelos cognitivo-comportamentais e intervenções psicossociais (Dias, 2007), que se focam em estratégias mais práticas e no desenvolvimento de competências. Os resultados de uma meta-análise realizada por Noar (2008), evidenciam que as intervenções comportamentais se têm revelado eficazes na redução do sexo desprotegido e no aumento do uso do preservativo, com resultados menos consistentes no que refere à redução do número de parceiros sexuais. Procurando operacionalizar estratégias de promoção e EpS, foram surgindo diferentes modelos, dos quais destacamos: os modelos de mudança comportamental e o HPM de Nola Pender. Os primeiros, pela sua importância na explicação da mudança de comportamentos, que têm sido amplamente utilizados na análise e compreensão do comportamento sexual e as suas componentes que têm sido aplicadas a vários programas de intervenção, considerados eficazes na promoção de comportamentos sexuais saudáveis e na prevenção do VIH/SIDA. O segundo, por se encontrar dentro dos modelos de promoção da saúde que centra o seu foco de atenção na intervenção de enfermagem.

Modelos Cognitivo-comportamentais

Os modelos cognitivo-comportamentais procuram explicar o desempenho de comportamentos preventivos a partir de constructos como a perceção de suscetibilidade, as intenções comportamentais e a perceção de controlo. Os modelos de cognição social fornecem uma compreensão parcial dos processos cognitivos envolvidos no processo de decisão comportamental (Straub, 2005). O comportamento é resultado de um processo complexo que comporta as atitudes, a análise de custo-benefício e o juízo quanto aos resultados e à eficácia. Embora a ênfase seja colocada nos modelos cognitivos, os processos sociais e ambientais devem também ser considerados, quer na forma de normas sociais e suporte social, nas barreiras à mudança e quer nos processos de controlo sobre o comportamento (Glanz & Bishop, 2010). São vários os modelos cognitivo-comportamentais que têm vindo a ser utilizados na compreensão dos determinantes dos comportamentos sexuais de risco, em particular no que diz respeito à transmissão da infeção pelo VIH/SIDA e outras IST, e dos quais destacamos os mais referenciados na literatura.

O *Modelo de Crenças da Saúde* - (*Health Belief Model*, Rosenstock - HBM, 1974) analisa os fatores preditivos e percursores dos comportamentos de saúde, sugerindo que os comportamentos resultam de uma ponderação racional dos potenciais custos e benefícios de um dado comportamento. O HBM postula que os indivíduos são mais propensos à mudança de comportamento quando percebem que podem estar vulneráveis à doença (perceção de suscetibilidade), que as consequências da doença podem ser graves (perceção de severidade), que há benefícios em adotar comportamentos preventivos (perceção de benefício), e que a mudança comportamental gera benefícios que superam os inconvenientes da adoção de determinada prática (perceção de barreiras). Embora na literatura haja muitas contradições à volta do modelo HBM, ele tem sido utilizado para predizer uma grande variedade de comportamentos ligados à saúde, incluindo os comportamentos sexuais. Alguns autores mencionam que o modelo tem tido um nível de sucesso moderado ao prever o uso do preservativo (Reid & Aiken, 2011), outros indicam que aceitar praticar sexo seguro, utilizar contraceção, ser vacinado contra o HPV, ou realizar rastreio do cancro do colo do útero, pode estar relacionados com as crenças de saúde (Ferreira, Ferreira, Ferreira, Andrade & Duarte, 2014; Mortensen, 2010; Secginili & Nahcivan, 2006).

A *Teoria da Autoeficácia* (Bandura, 1977) baseia-se na autorregulação do comportamento individual. Esta capacidade de se autorregular, acompanhada da convicção de que o indivíduo é capaz de realizar um dado comportamento, aumenta a possibilidade de que ele mantenha ou modifique o seu comportamento. Desta forma, a autoeficácia tem um importante papel na forma como aprendemos e mantemos novos comportamentos. Não obstante, a importância do conceito central desta teoria *per si*, a sua integração em muitas outras teorias, tem permitido que o seu contributo seja muito maior, como é o caso da Teoria Sociocognitiva. Na área da sexualidade, os estudos desenvolvidos nos últimos anos têm-se debruçado em particular sobre a autoeficácia para o uso do preservativo. Diversos estudos realizados com jovens têm permitido concluir que elevados níveis de autoeficácia estão relacionados com o uso efetivo do preservativo e capacidade de negociar sexo seguro (Artistico, Oliver, Dowd, Rothenberg & Khalil, 2014; Asante & Doku, 2010; Casey,Timmermann, Allen, Krahn & Turkiewicz, 2009), sendo que uma autoeficácia limitada na negociação do preservativo é considerada um fator de risco comportamental para o sexo seguro (French & Holland, 2013). Contudo os níveis de autoeficácia pessoal podem ser condicionados por diversas influências situacionais, nomeadamente o estatuto da relação da relação amorosa, pois o medo da confrontação, da rejeição do parceiro ou o embaraço podem colocar em causa a autoeficácia pessoal (Alvarez, 2005).

A *Teoria Sociocognitiva* (Bandura, 1977, 1986), sugere que o comportamento é resultado da interação entre o ambiente social, os fatores pessoais e o comportamento propriamente dito na determinação e previsão do comportamento futuro. Este modelo tem sido aplicado a uma série de domínios da saúde, nomeadamente em programas/intervenções que visam a promoção de comportamentos sexuais seguros, incluindo a prevenção do VIH/SIDA (Bandura, 1992; Wang et al., 2007). Três fatores deste modelo são particularmente importantes para programas de intervenção bem-sucedidos: i) perceção de autoeficácia, traduzida pela capacidade do indivíduo em acreditar ser capaz de executar um comportamento (ser capaz de negociar o preservativo com o companheiro); ii) normas percebidas, traduzidas pela aceitação dos amigos acerca de comportamentos que reduzem o risco sexual; iii) e as habilidades sociais, traduzidas na capacidade de responder de forma assertiva ou negociar comportamentos de risco sexual.

A *Teoria da Ação Racional* (*Theory of Reasoned Action* - TRA; Azjen & Fishbein, 1985) foi elaborada para explicar comportamentos na área da saúde e tem na sua intenção o maior preditor do comportamento. As intenções comportamentais são determinadas por dois processos cognitivos, as atitudes face a um comportamento (expectativa individual face às consequências de dado comportamento e o valor das mesmas para o indivíduo) e as normas subjetivas (influência social da família, dos amigos ou grupo de pares), e que se tem revelado um preditor importante, provavelmente devido à influência das normas sociais sobre a atividade sexual em muitas populações de risco, incluindo os jovens (Straub, 2005). A adaptação deste modelo aos comportamentos sexuais de risco tem sido feita com algum sucesso em programas de intervenção que visam a promoção de comportamentos sexuais seguros mas não tem permitido a compreensão dos motivos que desencadeiam os comportamentos sexuais de risco (Breakwell, Millward & Fife-Schaw, 1994). Diversos investigadores têm verificado de forma consistente, que indivíduos com atitudes mais favoráveis ao uso do preservativo, assim como aqueles cujos pares apoiam o uso do preservativo, têm mais probabilidade de terem sexo protegido (Bermúdez et al., 2012; Kirby et al., 2011).

A *Teoria do Comportamento Planeado* (*Theory of Planned Behavior* - TPB; Ajzen, 1991) foi desenvolvida a partir da TRA (Ajzen & Fishbein, 1985) com a intenção de prever e explicar o comportamento humano em contextos particulares e tem sido amplamente utilizada na análise de fatores que predizem os comportamentos de saúde. Para além do papel das normas subjetivas e das atitudes, a TPB enfatiza a importância do controlo comportamental percebido - perceção que um indivíduo tem de poder executar um comportamento desejado. A TPB tem sido utilizada para estudar diversos comportamentos sexuais, incluindo a intenção dos estudantes de usarem preservativo (Asare, 2015; Carmack & Lewis-Moss,

2009; Protogerou, Fisher, Wild & Aaro, 2013). Os resultados desses trabalhos confirmam que a TPB pode ser usada para orientar os programas de promoção do uso de preservativos entre estudantes, incluindo estudantes universitários.

A *Teoria das Fases de Mudança* (*Stages of Change Model*, Prochaska, Diclement & Norcross, 1992), onde a mudança de comportamento é conceptualizada num processo gradual que inclui cinco fases: pré-contemplação (o indivíduo não tem intenção de mudar, pelo que precisa de ter consciencialização do problema), contemplação (o indivíduo reconhece a necessidade de mudar), a decisão (prevê o momento de mudar), a ação (implementar planos específicos), e a manutenção (continua com ações positivas e mantém o comportamento ao longo do tempo). Apesar de suscitar algumas críticas, sobretudo pela fraca valorização dos fatores estruturais e ambientais, este modelo tem vindo a ser aplicado nas práticas sexuais de risco relacionadas com VIH/SIDA, com algum sucesso.

Apesar das contribuições das diferentes teorias da mudança comportamental para a redução do risco associado às IST e VIH/SIDA, persiste a necessidade do desenvolvimento de modelos que contemplem a complexidade do comportamento sexual. Cada modelo parece ser preditor de diferentes aspetos do comportamento e os modelos desenvolvidos especificamente para o comportamento sexual (como os dois abaixo mencionados), parecem ter vantagens na compreensão dos comportamentos sexuais de risco, em relação aos modelos genéricos para a saúde (Carvalho & Baptista, 2006).

O *Modelo de Redução do Risco do VIH/Sida* (*AIDS Risk Reduction Model*, Catania, Kegeles & Coates, 1990), integra elementos dos diferentes modelos para explicar a mudança comportamental, relacionada especificamente com os comportamentos sexuais de risco decorrentes da transmissão pelo VIH/SIDA. Este modelo propõe a mudança de comportamento em três fases: i) reconhecer que o comportamento o coloca em risco de infeção (noção de susceptibilidade individual); ii) responsabilizar-se pelo comportamento de risco e ter o compromisso de o mudar (a tomada de decisão resulta da ponderação do custo benefício e da autoeficácia para do uso do preservativo); iii) procurar estratégias para a redução de comportamentos de risco e adotar comportamentos preventivos.

O *Modelo das Competências de Informação-Motivação-Comportamento* (*Information, Motivation, and Behavioral Skills Model* – IBM, Fisher & Fisher 1992), procura compreender os comportamentos sexuais de risco para o VIH/SIDA, em função do conhecimento que os indivíduos têm sobre as formas de prevenção, a motivação para alterar os seus comportamentos sexuais de risco (que incluem as atitudes preventivas como, por exemplo, o uso do preservativo, normas sociais e a intenção de usar o comportamento preventivo) e as competências comportamentais para adotar comportamentos sexuais seguros. A

utilização do IBM tem sido defendida no desenvolvimento de intervenções sobre o VIH/SIDA e outras IST que impliquem o conhecimento como uma competência para comunicar acerca do preservativo e para negociar a sua utilização. O conhecimento, por si só, nem sempre provoca mudança de comportamento, mas tem sido demonstrada uma associação positiva com as variáveis de mudança de comportamento. Vários estudos confirmam as associações hipotéticas entre motivação, competências comportamentais e comportamento de risco; no entanto, o papel preditivo da informação tem-se revelado menos consistente, com estudos que encontraram associações positivas, negativas e nulas entre a informação, as competências e o comportamento (Fisher, Fisher & Shuper, 2009). Mesmo com estas inconsistências, os construtos que integram o modelo tem demonstrado elevada capacidade preditiva da mudança de comportamento, em particular no que respeita à utilização do preservativo (Reis et al., 2013; Walsh, Senn, Scott-Sheldon, Vanable & Carey, 2011).

Modelo de Promoção da Saúde de Nola Pender

O Modelo de Promoção da Saúde (*Health Promotion Model - HPM, Pender, 1996*) foi desenvolvido na década de 1980 do século XX, e integra o grupo dos modelos e teorias de enfermagem que podem ser aplicados no estudo dos diversos domínios dos comportamentos para desenvolver cuidados de enfermagem promotores de saúde. O modelo surgiu como proposta de ligação da enfermagem com as ciências comportamentais, procurando identificar fatores que influenciam comportamentos saudáveis, além de ser um guia para explorar o complexo processo biopsicossocial que motiva os indivíduos a optar por comportamentos promotores de saúde (Sakraida, 2004). Na construção do modelo, a autora utilizou como base teórica dois modelos da psicologia, a Teoria Cognitiva Social (Bandura, 1977), sobretudo o construto de autoeficácia, e a Teoria de Avaliação de Expectativas, centrada na importância da motivação (Feather, 1988).

Este modelo apresenta, ainda, quatro conceitos fundamentais para a enfermagem: i) a **saúde**, na sua dimensão individual, familiar e comunitária, coloca a ênfase na melhoria do bem-estar, no desenvolvimento de capacidades e não na mera ausência de doença. Numa perspetiva holística, a saúde deve ser estudada durante todo o processo de desenvolvimento do ser humano, tendo em consideração a idade, raça e cultura; ii) o **ambiente**, definido como o contexto social, cultural e físico, no qual o indivíduo se desenvolve, considerando que esta relação vai proporcionar um ambiente saudável; iii) a **pessoa**, entendida em toda a sua complexidade biopsicossocial como sendo capaz de tomar decisões, resolver problemas, numa relação recíproca com o meio ambiente. Nesta perspetiva, as características individuais, bem como experiências de vida, moldam os comportamentos de saúde; iv) **enfermagem**, que está relacionada com as estratégias e

intervenções que o enfermeiro deve aplicar para criar condições favoráveis aos comportamentos de promoção da saúde (Pender, Murdaugh & Parsons, 2011).

Para melhor compreensão do modelo em questão, apresenta-se a sua representação gráfica (Figura 1), onde é possível verificar-se a forma como cada variável se relaciona com as restantes varáveis do modelo. O HPM pode ser usado para planear, implementar e avaliar ações de promoção da saúde, pelo estudo das interrelações de três componentes principais que, embora independentes, se interligam entre si: i) as características e experiências individuais; ii) os sentimentos e conhecimentos sobre o comportamento; iii) e o comportamento de promoção da saúde. A primeira componente, “características individuais e experiências”, compreende o comportamento anterior, ou seja, o comportamento que precisa ser mudado e os fatores pessoais que se dividem em fatores biológicos (idade, género), psicológicos (autoestima, competência pessoal, estado de saúde percebido) e socioculturais (educação, nível socioeconómico). A segunda componente, “ Conhecimento e sentimentos sobre o comportamento" que se pretende alcançar, inclui as variáveis que são consideradas como o núcleo central do modelo, uma vez que podem ser potencialmente modificadas através de intervenções. Estas variáveis incluem os benefícios percebidos à ação, que são representações mentais positivas que fortalecem as consequências de adotar um comportamento de saúde, barreiras percebidas à ação, que são as perceções negativas sobre um comportamento ou dificuldades, a autoeficácia percebida, que se relaciona com as capacidades pessoais de organizar e executar ações e os sentimentos positivos ou negativos subjetivos relacionados com o comportamento. Integra, ainda, as influências interpessoais que incluem normas, apoio social e modelagem, sendo consideradas como recurso, a família, o(a) companheiro(a), os pares, os profissionais de saúde e as influências situacionais relacionadas com o ambiente onde a pessoa está inserida e que podem facilitar, ou impedir, determinados comportamentos de saúde. A terceira componente, “o resultado do comportamento”, ao equacionar o compromisso com um plano de ação que possibilita a pessoa manter-se no comportamento de promoção de saúde esperado, reforçado pelas intervenções de enfermagem e pelas exigências imediatas e preferências (comportamentos alternativos sobre o qual as pessoas têm um controlo reduzido, no caso das exigências ou um grande controlo no caso das preferências), permite concretizar o comportamento de promoção de saúde que representa o resultado da ação orientado para a obtenção de resultados de saúde (Mccullagh, 2009; Pender et al., 2011; Sakraida, 2004; Vítor et al., 2005).

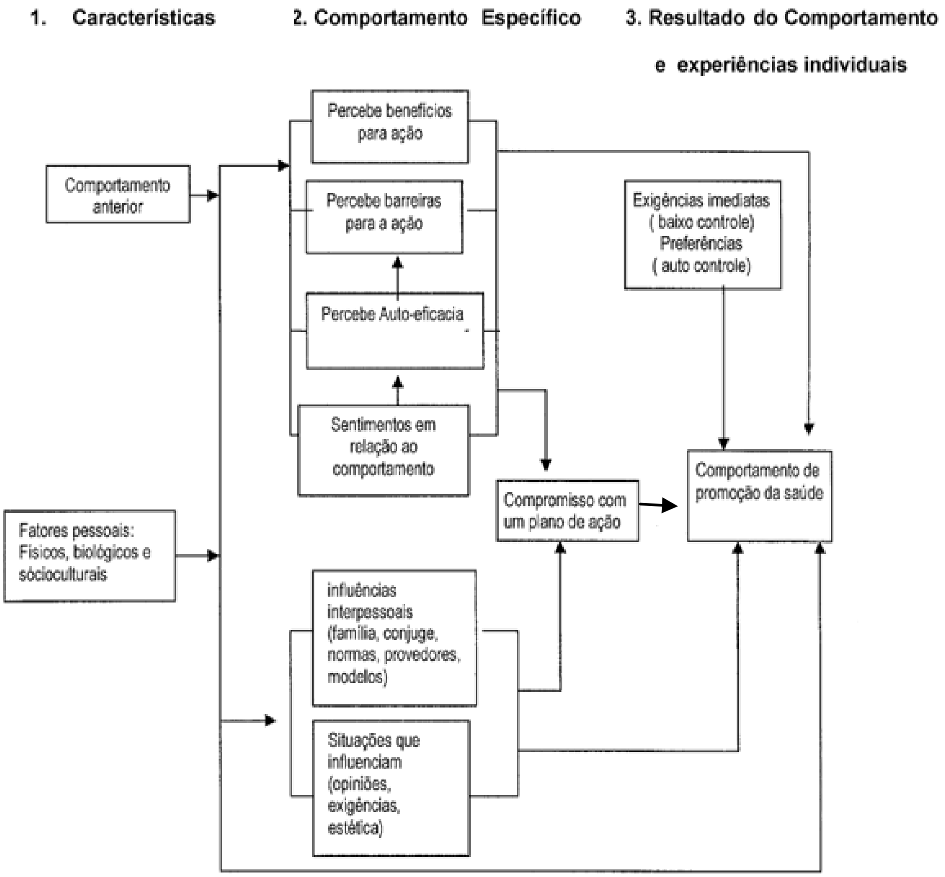

Figura 1. Componentes do Modelo de Promoção da Saúde de Pender (adaptado de *Health Promotion in Nursing Practice*, Pender et al., 2011).

Da análise das variáveis do HPM, é possível verificar algumas aproximações aos conceitos avançados nos modelos cognitivo-comportamentais referidos anteriormente, do qual emergem alguns pressupostos teóricos de relevância para o exercício profissional dos enfermeiros quando se pretende implementar intervenções de promoção da saúde (Pender et al., 2011), e que podem também ser usados na promoção da saúde sexual e reprodutiva dos jovens adultos:

- *Características individuais e experiências anteriores:* as características individuais e o comportamento anterior influenciam as crenças individuais e o seu envolvimento no comportamento de promoção da saúde. O comportamento anterior pode influenciar, direta ou indiretamente, a probabilidade da pessoa para se envolver nos comportamentos promotores de saúde, o que pode ser explicado pela formação do hábito.

- *Benefícios e barreiras percebidas*: as pessoas comprometem-se mais facilmente com comportamentos dos quais prevêem benefícios, e as barreiras percebidas podem restringir o comprometimento com a ação.

- *A autoeficácia percebida*: a autoeficácia aumenta a probabilidade de compromisso com um plano de ação, diminui a perceção de barreiras e aumenta a probabilidade de o indivíduo adotar comportamento saudáveis. A autoeficácia é um construto relevante na educação em saúde, uma vez que defende um dos princípios mais importantes na promoção da saúde, que é: o processo de capacitar os indivíduos para aumentar a perceção de controlo que pode melhorar a sua saúde.

- *Sentimentos positivos e negativos*: o afeto positivo em relação a um comportamento resulta em maior autoeficácia percebida que, por sua vez, aumenta as representações positivas sobre o mesmo, reforçando a probabilidade de compromisso com um comportamento de promoção da saúde e de implementação do mesmo. Os afetos positivos também podem ser traduzidos pelas atitudes positivas, conceitos centrais do HBM. Duas das dimensões do HBM, suscetibilidade e crença na gravidade das consequências, podem ser interpretadas como sentimentos negativos.

- *Influências interpessoais*: as pessoas comprometem-se e adotam comportamentos de promoção da saúde mais facilmente se observarem esse comportamento nas pessoas que lhe são próximas e significativas (normas subjetivas ou sociais). A família, o grupo de pares, os profissionais de saúde os contextos podem influenciar positiva ou negativamente a adoção de comportamentos de promoção de saúde, em particular pela mudança de atitude face ao comportamento de risco (Pender, et al., 2011; Sakraida, 2004).

- *Influências situacionais*: compreendem o ambiente físico e interpessoal para criar incentivos à adoção de comportamentos de promoção da saúde. O ambiente deve ser compreendido como resultado das relações entre os indivíduos e o seu acesso a recursos de saúde, sociais e económicos, na medida em que esta relação proporciona um ambiente saudável.

- **Compromisso com o plano de ações e exigências imediatas e preferências**: quanto maior for o compromisso da pessoa com o plano de ação específico, maior será a probabilidade de realizar o comportamento promotor da saúde e de o manter ao longo do tempo. Em sentido oposto atuam as exigências de competição, sobre as quais a pessoa tem pouco controlo, pois diminui a probabilidade do compromisso com o plano de ação resultar no comportamento promotor de saúde. O mesmo se passa quando outras ações são mais atrativas e, portanto, preferidas à adoção do comportamento desejado.

Em termos conceptuais, o HPM fornece um método para avaliar comportamentos promotores de saúde, tendo em consideração não só as experiências anteriores e características individuais, a perceção de autoeficácia, a perceção de barreiras e benefícios, as influências interpessoais e situacionais, mas também contempla a natureza holística das

pessoas em interação com o meio onde se inserem enquanto procuram saúde, direcionando a ação dos enfermeiros para desenvolver intervenções de enfermagem promotoras de saúde, no âmbito da promoção da saúde ao longo do ciclo de vida (Mccullagh, 2009; Pender et al., 2011). Os mesmos autores acrescentam, ainda, que a literacia em saúde é uma estratégia que deve ser sempre considerada quando procuramos promover na saúde, pois permite que as pessoas assumam a responsabilidade e o controlo sobre os determinantes pessoais, sociais e ambientais da sua saúde. Nesta perspetiva, as intervenções de enfermagem serão essencialmente do tipo educar, ensinar, instruir e treinar competências para comportamentos sexuais seguros. O planeamento e a implementação das intervenções serão adaptados às necessidades e características específicas da pessoa ou do grupo, aos objetivos que se pretende atingir, sem descurar o papel central do compromisso da pessoa com o plano de ação.

Este modelo tem sido utilizado por diversos investigadores para estudar comportamentos promotores da saúde em diversas áreas da saúde e bem-estar, tais como o exercício físico, alimentação saudável, controlo da hipertensão, autocuidado e comportamento sexuais de pessoas com VIH/SIDA (Dehdari, Rahimi, Aryaeian & Gohari, 2014; Marziale, Zapparoli, Felli & Anabuki 2010; Melo et al., 2016; Pender & Yang, 2002; Santos, Barlem, Silva, Cestari & Lunardi, 2008; Santos, Soares & Berardinelli, 2013), no entanto, não encontramos referenciais teóricos da utilização deste modelo na área da promoção da SSR, na prática de enfermagem em Portugal. Neste contexto, consideramos importante a utilização do suporte teórico do HPM de Pender, pois permite perceber a complexidade dos fenómenos do comportamento de saúde, no sentido de delinear estratégias de intervenção em enfermagem, cujo objetivo é a promoção da SSR e a mudança comportamental.

Apesar das contribuições conceptuais dos modelos de mudança de comportamento e do HPM para compreender a complexidade dos comportamentos de saúde, alguns autores apontam-lhes limitações. Relativamente aos modelos cognitivo-comportamentais, deve ter-se presente que os comportamentos não dependem exclusivamente das experiências individuais, mas também das relações interpessoais, sociais, culturais e ambientais (Glanz & Bishop, 2010). Na sua complexidade, o comportamento sexual não depende exclusivamente de comportamentos individuais determinados por decisões racionais conscientes, orientadas pelo desejo de se preservar a saúde, mas também de determinantes sociais e culturais. Estes modelos pressupõem também que o comportamento sexual é planeado, esquecendo que, de facto, ele é frequentemente impulsivo e, muitas vezes, motivado por fatores emocionais e afetivos, em particular nas faixas etárias mais jovens (Dias, 2009).

No que respeita ao HPM, as principais limitações estão relacionadas com a complexidade do modelo e o grande número de variáveis que têm de ser medidas para testar o modelo

completo, pelo que diversos estudos se limitam a testar somente um número limitado de construtos como preditores de alguns comportamentos. Apesar das limitações, algumas das variáveis que integram o modelo, tais como a autoeficácia, as barreiras percebidas e as influências interpessoais, são recorrentemente apontados como preditores consistentes dos comportamentos de saúde (Pender et al., 2011). Reconhecendo o contributo de cada um dos modelos, para o diagnóstico dos fatores relacionados com os comportamentos sexuais de risco, diversos autores têm advogado a necessidade de os modelos serem considerados não de forma isolada, mas como complementares, considerando nesta complementaridade não só o nivel individual, mas também os determinantes contextuais e socioculturais (Conner & Armitage, 1998; Porter, 2002).

1.3. Saúde sexual e reprodutiva em estudantes do ensino superior

1.3.1. Os estudantes do ensino superior

A palavra "juventude" tem assumido diferentes significados, de acordo com o contexto histórico, social, económico e cultural vigente. O sentido mais comummente encontrado é aquele que a define como uma fase de transição entre a adolescência e a vida adulta (Silva & Silva, 2008). Segundo a WHO (2002) e a UNFPA (2005), a juventude é uma categoria sociológica que representa um momento de preparação dos indivíduos para assumirem o papel de adultos na sociedade abrange o período dos 15 aos 24 anos de idade, e considera como jovens as pessoas entre os 10 e 25 anos. As alterações nos padrões sociais e culturais dos países industrializados levam a que os marcos que tradicionalmente definiam o início da idade adulta - casamento e parentalidade - sejam adiados. Tendo em conta estas mudanças, Arnett (1998, 2004) propôs a delimitação de uma nova fase no ciclo de vida dos indivíduos, a qual designou por *adultez emergente ("emerging adulthood")*. O autor situa esta fase entre os 18 e 25 anos e caracteriza-a como sendo a idade da exploração da identidade, na medida em que, ao longo deste período, existe a exploração de diversas possibilidades, principalmente na vida profissional e afetiva. Neurocientistas do desenvolvimento procuram explicar esta fase "adultos emergentes", justificando que o cérebro humano, ao contrário do que se pensava, continua a desenvolver mecanismos de controlo cognitivo ao longo da adolescência e no início da vida adulta, consistente com as alterações anatómicas no córtex pré-frontal dorso-lateral, com fortes implicações no raciocínio e tomada de decisão. Embora a rede de controlo cognitivo esteja fortemente implicada no raciocínio e na tomada de decisão, depende também da rede de suporte sócio-emocional, onde os contextos assumem um papel relevante (Steinberg, 2008).

Também a sociedade portuguesa parece manifestar características que denunciam a presença desta fase desenvolvimental, dado que, nas últimas décadas, se tem assistido ao prolongamento dos percursos académicos, com adiamento dos processos de transição para a vida profissional, assim como a um aumento progressivo nas idades do casamento ou união de facto, do nascimento do primeiro filho, um fenómeno que se tem verificado principalmente em jovens com formação universitária (Mendonça, Andrade & Fontaine, 2009). A grande adesão ao ensino superior alterou de forma significativa os percursos e projetos dos jovens portugueses, constituindo-se como um contexto que proporcionou o adiamento da transição para a vida adulta, ampliou as expectativas em relação ao futuro e causou uma maior diversificação do perfil social dos jovens (Guerreiro & Abrantes, 2004). O ensino superior em Portugal sofreu, nos últimos 30 anos, profundas alterações na sua estrutura e configuração. Atualmente, Portugal tem uma proporção de estudantes no ensino superior semelhante à de muitos países da União Europeia (UE), observando-se um crescimento do número de estudantes no ensino superior, que no ano de 2016 se situa nos 356.399, dos quais 166 177 rapazes e 190 282 raparigas, (Pordata, 2016).

Na sociedade ocidental, a juventude é marcada por acontecimentos normativos, tais como a transição para o ensino superior, que pressupõe a realização de duas tarefas nucleares deste período desenvolvimental – construção da autonomia e da intimidade (Faria, 2008). Provavelmente, pela primeira vez o jovem será responsável por si próprio e vai desenvolver relações íntimas com os seus pares, quer de carácter amoroso quer de amizade, integradas numa rede social complexa e contextos de grande diversidade social (Cavanhaug, 2005). O jovem adulto encontra-se numa fase de conquista da autonomia psicológica e emocional. Neste período, é essencial o jovem ser capaz de construir e manter relações íntimas, criando laços afetivos promotores de segurança, assumir diversas responsabilidades e tornar-se autónomo, ao mesmo tempo que se afasta do ciclo familiar e de amigos (Soares, Pereira & Canavarro, 2015). Todavia, muitos jovens, quando ingressam no ensino superior, não apresentam a maturidade psicoemocional, autonomia e confiança necessárias para assumir escolhas e gerir interações socialmente ajustadas (Matos et al., 2011). Este período nem sempre é um processo bem-sucedido, podendo constituir, para alguns estudantes menos resilientes, momentos de isolamento social, desinteresse e até mesmo de alterações psico-emocionais e afetivas, com repercussões significativas na sua saúde (Freitas, Martins & Vasconcelos, 2003). Adicionalmente, muitos jovens iniciam a vida académica longe do seu meio de origem, o que implica uma modificação no padrão de procura dos serviços de saúde, ao mesmo tempo que adquirem novos hábitos e comportamentos relacionados com o contexto académico, os quais podem originar problemas de saúde.

A vigilância de saúde dos jovens está preconizada pelos serviços de saúde no Programa Nacional de Saúde Infantil e Juvenil (DGS, 2013) apenas até aos 18 anos, sendo que a partir desta idade fica à responsabilidade de cada indivíduo, de acordo com as suas preocupações, necessidade de cuidados ou integrada na vigilância de saúde do adulto. Neste sentido, Prazeres et al. (2006) consideram que é um imperativo adequar, de forma mais efetiva, a abordagem às necessidades de saúde deste grupo etário, uma vez que, não pertencendo ao mundo infantil, ainda não alcançaram em pleno o estatuto de adultos. Acrescentam, ainda, que mais do que lidar com algumas particularidades biológicas ou psicológicas dos indivíduos, o que está em causa é apoiar, educar, proteger e prevenir eventuais repercussões negativas para a saúde causadas pelos novos desafios de carácter académico, laboral e de socialização que encaram, resultado das vivências que o processo de aquisição plena da cidadania vai exigindo. No mesmo sentido, nas conclusões do relatório do estudo de âmbito nacional sobre SSR em estudantes universitários (Matos et al., 2011), os autores mencionam que, embora a educação sexual nas escolas tenha vindo a cumprir o seu papel, existe efetivamente uma percentagem significativa de jovens com potencial risco sexual e reprodutivo a frequentar as universidades portuguesas.

1.3.2. Comportamentos de saúde sexual e reprodutiva

O grupo dos jovens adultos, onde se incluem os estudantes do ensino superior, tem sido considerado um grupo prioritário nas questões relacionadas com a SSR (WHO, 2010), dado que vários estudos têm demonstrado que este se encontra em situação de particular risco para aquisição de IST e do VIH/SIDA, gravidez não desejada e IVG, resultado do tipo de comportamentos sexuais que adotam (Kann et al., 2014; Trieu, Bralton & Marshak, 2011). A vulnerabilidade dos jovens nas questões de SSR relaciona-se também com a idade das primeiras experiências sexuais, a maior suscetibilidade biológica para a infeção e as barreiras no acesso dos jovens aos serviços de saúde (Falcão Júnior et al., 2009; Workowski & Bolan, 2015). Nesta perspetiva, o conhecimento dos comportamentos sexuais dos jovens adultos constitui uma necessidade, do ponto de vista epidemiológico, à qual se associa a necessidade de conhecer a racionalidade das escolhas, os fatores de riscos e de prevenção, dado que as questões de SSR não podem ser entendidas de uma forma isolada mas inserida no contexto das relações individuais e sociais (Ferreira & Cabral, 2010).

No que diz respeito aos comportamentos sexuais e reprodutivos, têm-se constatado diferenças entre os géneros nos marcadores temporais (idade da primeira relação sexual e frequência das relações sexuais) e nos aspetos relacionais, que dizem respeito ao estatuto afetivo do parceiro e aos motivos que os jovens apontam para o início da atividade sexual (Ferreira & Cabral, 2010), factos que se têm observado tanto na Europa como em Portugal

(Currie et al., 2012). No estudo HBSC/SSREU realizado em Portugal (Matos et al., 2011), dos 3278 estudantes universitários, a maioria já tinha iniciado relações sexuais e a primeira relação sexual ocorreu aos 16 anos ou mais, com os rapazes a iniciarem relações sexuais mais cedo do que as raparigas. O motivo mais apontado para o início das relações sexuais foi uma decisão por consentimento mútuo, em particular no caso das raparigas, referindo os que iniciaram as relações sexuais por acaso ou porque tomaram a iniciativa. A maioria dos jovens refere, ainda, ter um relacionamento do tipo afetivo e consideram que os sentimentos, a comunicação e o prazer sexual são muito importantes numa relação, embora os rapazes mais velhos atribuam sempre maior importância à vertente erótico-hedonista da relação. De facto, os resultados de diversos estudos revelam diferenças de género no que respeita às atitudes sexuais, demonstrando que os rapazes, contrariamente às raparigas, manifestam atitudes mais permissivas para com o sexo ocasional e sem compromisso, aceitando a diversidade de parceiros e uma maior aceitação do risco sexual. Por sua vez, as raparigas apresentam maior preocupação preventiva, evidenciando atitudes mais positivas face ao planeamento familiar e à educação sexual (Antunes, 2007; Janeiro, Oliveira, Rodrigues, Macieiras & Rocha, 2013; Matos et al., 2011).

Comportamentos de contraceção e infeções sexualmente transmissíveis

A contraceção e a prevenção de IST constituem duas importantes vertentes da saúde reprodutiva e a sua interação é inequívoca, pelo que a realização da sua abordagem conjunta junto dos jovens, representa mais uma oportunidade para informação, educação e prevenção da SSR. A prática de sexo seguro implica não só o conhecimento dos métodos contracetivos, mas também das estratégias de redução da incidência das IST (Neves, 2013).

Dados do último estudo de âmbito nacional de avaliação das práticas contracetivas das mulheres portuguesas (Sociedade Portuguesa de Ginecologia [SPG] & Sociedade Portuguesa de Contraceção [SPC], 2015), revelam que 97% das jovens entre os 20-29 anos de idade utilizam contraceção. A pílula continua a ser o método mais utilizado (57%), seguida do preservativo quando considerado isoladamente (14,2%). A utilização do duplo método (pílula e preservativo) tem maior expressão nos grupos etários dos 15 aos 19 anos e dos 20 aos 29 anos (24%). Situando-nos nos comportamentos de SSR dos estudantes universitários, quer o estudo de âmbito nacional (Matos et al., 2011), quer os estudos parcelares (Cunha-Oliveira et al., 2009; Gomes & Nunes, 2011; Ribeiro & Fernandes, 2009), revelam que o padrão de utilização dos métodos contracetivos é similar. Os jovens sexualmente ativos utilizam frequentemente os métodos contracetivos, sendo os métodos mais utilizados a pílula e o preservativo, este último mais utilizado pelos rapazes. Embora exista uma maior tendência para o uso de métodos hormonais menos dependentes da

utilizadora, como o anel vaginal, o adesivo e o implante (SPG & SPC, 2015), entre estudantes universitários a utilização destes métodos é ainda pouco comum (Matos et al., 2011). Procurando ultrapassar esta dificuldade, a lista nacional de contracetivos disponibilizados de forma gratuita pelo Serviço Nacional de Saúde (SNS) tem sido progressivamente alargada, oferecendo uma diversidade de métodos que permita uma escolha adaptada a um maior número de utentes e garanta a liberdade de escolha e uma maior adesão ao método (DGS, 2015). Neste contexto, é importante o aconselhamento dos jovens sobre as diferentes opções contracetivas para que possam tomar decisões mais informadas, uma vez que a idade, por si só, não constitui contraindicação à utilização de qualquer método e a maioria deles pode ser usada sem restrições nesta faixa etária (Barros & Neves, 2013).

Relativamente à pílula contracetiva, mantêm-se os problemas de má utilização, pois 22% das mulheres admite esquecer-se de a tomar em todos os ciclos ou mais de uma vez por mês, problema mais grave nas utilizadoras com idade inferior a 29 anos, onde atinge os 40% (SPG & SPC, 2015). O mesmo relatório menciona que uma elevada percentagem (88%) das mulheres sexualmente ativas conhece a pílula de emergência e 17% afirma já a ter utilizado. No que diz respeito aos estudantes universitários, o estudo nacional refere percentagens menores de utilização da contraceção de emergência pelos estudantes universitários, situando-se esta nos 2,6% (Matos et al., 2011). Será importante consciencializar as utilizadoras para o facto de que as falhas atribuídas à pílula decorrem maioritariamente de uma má utilização e que comportam um risco acrescido para a gravidez não desejada (SPG & SPC, 2015). Portugal, tem a oitava maior taxa de gravidez na adolescência da UE, sendo que, em 2011, o número de nascimentos nesta faixa etária (10 e 19 anos) corresponde a 3,8% do total de nascimentos (Eurostat, 2014). Dada a natureza esporádica e, por vezes, não planeada do comportamento sexual dos jovens, o aconselhamento e a disponibilização da contraceção de emergência deve também fazer parte de uma orientação dos jovens e deve ser sempre acompanhada da mensagem de que a eficácia contracetiva desta é inferior à de qualquer um dos métodos de contraceção regular (Santos & Figueiredo, 2015). Alguns estudos mostram que os jovens não compram contracetivos por timidez, por receio dos potenciais efeitos colaterais (aumento de peso, aumento do risco de cancro) e por desconhecimento sobre os métodos contracetivos mais adequados às suas necessidades e ao modo como os obter. As questões sociais e de comportamento são fatores relevantes na escolha do método contracetivo e incluem aspetos como os padrões de relações – muitas vezes esporádicas - necessidade de esconder a prática de relações sexuais da família, a acessibilidade e a disponibilidade financeira (APF, 2010). Para além de se assegurar a disponibilidade dos contracetivos, é essencial reforçar a

educação contracetiva dos jovens no sentido de potenciar a sua capacidade de escolha, investindo também na formação específica dos profissionais de saúde, uma vez que diversos estudos comprovam que um bom aconselhamento contracetivo melhora a adesão e diminui a taxa de descontinuação do método contracetivo (Alli, Maharaj & Vawda, 2013; DGS, 2010; Geary, Gómez-Olivé, Kahn, Tollman & Norris, 2014).

Verifica-se um consenso generalizado de que o uso correto e consistente de métodos contracetivos, particularmente de métodos de barreira, tais como o preservativo masculino e feminino, é fundamental na prevenção das IST e da gravidez indesejada (CDC, 2014; Joint United Nations Programme on HIV/AIDS [UNAIDS], 2013). As Sociedades Portuguesas de Ginecologia, Contraceção e de Medicina da Reprodução, recomendam aos jovens a utilização de métodos contracetivos eficazes de forma correta e consistente, sempre em associação com o preservativo para uma prevenção simultânea da gravidez não planeada e das IST (Pacheco et al., 2011). No entanto, a utilização do preservativo pelos jovens nem sempre é consistente, existindo uma tendência para abandonar o uso do preservativo quando têm um parceiro regular (Cunha-Oliveira et al., 2009; Gomes & Nunes, 2011; Hickey & Cleveland, 2013; Kann et al., 2014). Também no contexto académico, têm vindo a ser identificados outros comportamentos de risco para a SSR dos estudantes universitários, designadamente a prática de relações sexuais desprotegidas com parceiros ocasionais e com outras pessoas para além do parceiro habitual (Certain, Harahan, Saewyc & Fleming, 2009; Kuperberg & Padgett, 2015; Pacheco, 2012; Reis et al., 2013), a utilização de substâncias psicoativas, sobretudo álcool, associado às relações sexuais, considerado quase normativo no contexto da noite e das festas académicas (Burnett et al., 2014; Eaton et al., 2010; Wicki et al., 2010). O consumo crescente de álcool pelos jovens universitários tem-se revelado uma preocupação em diversos países, em virtude da sua associação a comportamentos potencialmente prejudiciais à saúde, incluindo as relações sexuais sem proteção (Pedrosa, Camacho, Passos & Oliveira, 2011; Rodrigues, Salvador, Lourenço & Santos, 2014; Wicki et al., 2010). Destaca-se, em particular, o fenómeno denominado de “binge drinking”, que pode ser definido como um consumo esporádico e excessivo de álcool, num curto espaço de tempo e normalmente em contexto de grupo (Wechsler, Kuh & Davenport, 2009). Dados de alguns dos estudos nacionais em estudantes universitários revelam que cerca de um terço dos estudantes teve relações sexuais associadas ao consumo de álcool, em particular os rapazes, mais velhos e com relacionamentos mais recentes (Pacheco 2012; Reis et al., 2011). Esta preocupação com a associação entre o não uso do preservativo e o consumo de bebidas alcoólicas tem sido identificada desde a adolescência. Face aos resultados do último relatório sobre a saúde dos adolescentes (Matos et al., 2014), os autores sugerem que a prevenção deste comportamento deverá ser

centrada nesse grupo etário mais jovem. O álcool é usado como desinibidor emocional, reduzindo as inibições e aumentando a confiança para novos contactos sociais, o que pode facilitar o sexo com parceiros ocasionais (Fielder & Carey, 2010). Contudo, o consumo de substâncias psicoativas está associado a alterações de pensamento, com consequências diretas na capacidade dos jovens para usar corretamente e negociar o uso do preservativo (Uecker, 2015; Wilton, Palma & Maramba, 2014).

As IST continuam a ser um problema de saúde pública com grande significado em diversos países, com elevadas taxas de prevalência na população mais jovem (CDC, 2014). A prevalência destas infeções mantém-se elevada nos países em vias de desenvolvimento, mas também nos países desenvolvidos, estimando-se em cerca de 200 milhões de novos casos/ano, a incidência de apenas quatro dessas infeções em mulheres em idade reprodutiva: *Chlamydia*, sífilis, gonorreia e tricomoniose. O facto do número de casos de IST estar a aumentar nos jovens adultos, pode refletir uma melhoria dos sistemas de vigilância epidemiológica e dos meios de diagnóstico (Singh, Darroch & Ashford, 2014), mas também um aumento dos comportamentos sexuais de risco (Logan Koo, Kilmer, Blayney & Lewis, 2015; Sheperd et al., 2010).

Dados dos relatórios de vigilância epidemiológica dos EUA e da UE demonstram que se têm mantido, nos últimos anos, alguns comportamentos de risco, com reflexos negativos na SSR dos jovens (entre os 10-24 anos), nomeadamente a gravidez não desejada, VIH/SIDA e outras IST (Kann et al., 2014), encontrando-se entre as mais prevalentes nesta faixa etária a *Chlamydia*, gonorreia, sífilis e o HPV (CDC, 2014). A infeção pelo VIH/SIDA é uma das IST mais comuns e que, ao longo de três décadas, causou a morte de cerca de 35 milhões de pessoas em todo o mundo e continua a afetar, anualmente, cerca de 300 milhões de indivíduos (UNAIDS, 2013). A doença, outrora considerada uma ameaça de morte, tornou-se, hoje, uma doença crónica, graças aos avanços terapêuticos. Contudo a epidemia não está resolvida e a Europa é o único continente onde o número de novas infeções continua a aumentar. Os últimos dados divulgados pelo Departamento de Doenças Infeciosas do Instituto Nacional de Saúde Dr. Ricardo Jorge e Unidade de Referência de Vigilância Epidemiológica (Martins & Shivaji, 2014), confirmam a tendência, uma vez que, do total de 47390 casos de VIH/SIDA notificados até 2013, as taxas mais elevadas (11,2% e 18,8%) situam-se em indivíduos com idades compreendidas entre 20-24 anos e 25 aos 29 anos, respetivamente. Na maioria dos casos (61,1%), a infeção foi adquirida por via sexual. É importante consciencializar os indivíduos da importância do diagnóstico precoce do VIH/SIDA, que não é exclusivo dos grupos de risco, mas de todos os indivíduos com comportamentos de risco, dado que o tratamento antirretroviral potencia a sobrevida dos indivíduos infetados (Oliveira, 2014). Relativamente às restantes IST, os dados

epidemiológicos não se encontram sistematizados, mas Portugal segue a tendência dos países da UE, onde a infeção por Chlamydia é das mais frequentes, com cerca de 350 mil casos notificados em 2011, mais de três quartos diagnosticados em jovens com menos de 25 anos (ECDC & WHO Regional Office for Europe, 2014). Embora as taxas de notificação da gonorreia e sífilis variem bastante de país para país, desde 2010 têm-se observado um aumento nos países da UE, situando-se as taxas de prevalência da gonorreia nos 10,4 por 100 mil casos e as taxas de sífilis nos 4,5 por 100 mil casos. A classe etária dos 25 aos 34 anos é a mais afetada, com 32% do total de casos de gonorreia, seguindo-se a classe etária dos 20-24 anos com 28%. Da mesma forma, a prevalência da sífilis é maior na classe etária dos 25-34 anos (35% do total de casos reportados), (ECDC & WHO Regional Office for Europe, 2014). Relativamente à infeção pelo HPV, Pista e colaboradores (2011) determinaram uma prevalência de 28,8% nas mulheres entre os 18-24 anos, sendo que em 68% dos casos estava presente um tipo de HPV com potencial oncogénico (HPV 16). O HPV é atualmente uma causa bem estabelecida de cancro cervical, sendo responsável por 70% dos casos. Há crescente evidência de que o HPV é também um fator de risco para outros tipos de cancro a nível anogenital (ânus, vulva, vagina e pénis) e da cabeça e pescoço. Em 2012, a nível mundial, 527 624 mulheres foram diagnosticadas com cancro cervical e 265 653 morreram da doença (Bruni et al., 2015).

Estudos epidemiológicos demonstram uma forte associação entre o cancro do colo do útero e os padrões de comportamento sexual (Koshiol et al., 2008), uma vez que a relação sexual é a principal via de transmissão da infeção genital por HPV (Bruni et al., 2015) e a infeção com estirpes oncogénicas do HPV representa o principal factor de risco para o seu desenvolvimento (Kurtinaitiene, Drasutiene, Apeikiene & Ragauskyte, 2007; Oh, Lim, Yun, Lee & Shin, 2010). Torna-se fundamental fazer a EpS e a sensibilização dos jovens para as medidas de prevenção que passam essencialmente pela utilização do preservativo e pela vacinação das raparigas (CDC, 2014). Alguns autores vieram recentemente confirmar que intervenções na área da educação aumentam os conhecimentos e a consciência sobre os riscos associados à infeção pelo HPV e influenciam positivamente as atitudes e os comportamentos face às medidas de prevenção (Höglund, Gottvall, Tydén, Hoglund & Larsson, 2009), nomeadamente a aceitabilidade da citologia vaginal e a adesão à vacinação (Bruni et al., 2015; Gerend & Shepherd, 2011). Portugal, introduziu a vacina no seu plano nacional de vacinação para raparigas adolescentes com 13 anos em 2007, e as taxas de cobertura vacinal situam-se nos 84% (ECDC, 2012).

A introdução de medidas preventivas para as IST reveste-se de particular importância na medida em que os jovens são identificados dentro dos grupos populacionais de risco (APF, 2015), dada a sua vulnerabilidade biológica, psíquica e social. Estas infeções têm

implicações graves na saúde reprodutiva a curto e a longo prazo, exigindo tratamentos rápidos e modificações no comportamento sexual dado que provocam lesões irreversíveis, como a dor pélvica crónica, infertilidade e gravidez ectópica na mulher e, em caso de gravidez, podem causar problemas na saúde da criança (Haggerty et al., 2010; WHO, 2011). Neste contexto, a APF (2015) recomenda como medidas de controlo das IST: i) a melhoria dos sistemas de monitorização e colheita de dados, que permita o conhecimento real da situação das IST em Portugal, quer do ponto de vista clínico e laboratorial, mas também sociodemográfico e comportamental; ii) aumentar a acessibilidade aos serviços de saúde e incluir o rastreio das IST mais prevalentes (*Chlamydia*, sífilis, gonorreia e VIH) como norma, nas consultas periódicas de saúde, inclusive nas consultas de jovens sexualmente ativos; iii) a comparticipação dos exames médicos de rastreio, incluindo os testes anuais de pesquisa de *Chlamydia* em jovens até aos 25 anos, repetindo os testes três meses após o tratamento, uma vez que as taxas de reinfeção são frequentes, e nos grupos de risco realizar também o rastreio de VIH e sífilis (Mansouri & Santos, 2014); iv) a notificação e tratamentos dos parceiros sexuais; v) a elaboração de programas de saúde focados nos grupos vulneráveis, incluindo os jovens com comportamentos de risco para a aquisição de IST e VIH/SIDA; vi) incluir a temática das IST, das formas de transmissão, da prevenção e da história natural das doenças nas aulas de educação sexual.

Acesso aos serviços e cuidados de saúde sexual e reprodutiva

A existência de serviços de SSR apropriados, economicamente acessíveis e integrados, é considerada uma estratégia fundamental na promoção da saúde global dos jovens (Silva & Meneses, 2010; Vilar & Ferreira, 2010). Em Portugal, a legislação e o quadro normativo que rege o SNS contemplam alguns aspetos fundamentais no que diz respeito à saúde reprodutiva, nomeadamente o acesso dos jovens ao PF, contraceção e IVG (APF, 2015). Por sua vez, a DGS reconhece que os cuidados nesta área devem disponibilizar um conjunto diversificado de serviços, técnicas e métodos que contribuam para a saúde e o bem-estar reprodutivo dos indivíduos, através da prevenção e resolução de problemas, dando respostas adequadas às suas necessidades específicas (DGS, 2008). Contudo, continuam a existir barreiras e assimetrias no acesso aos serviços e cuidados de saúde reprodutiva, sobretudo pelos jovens, conforme documentado pelos dados do mais recente estudo nacional das práticas contracetivas (SPG & SPC, 2015), onde se constatou que 40% das mulheres com vida sexual ativa e a usar contraceção não frequentou no último ano a consulta de PF, sendo que as percentagens são ainda mais elevadas nas idades mais jovens (90% adolescentes; 50% entre os 20 e 29 anos).

A investigação desenvolvida no âmbito do "Projeto SAFE: uma parceria Europeia para promover a Saúde e Direitos Sexuais e Reprodutivos de Jovens", identificou como barreiras

no acesso dos jovens aos serviços de SSR, a insuficiência de serviços específicos para jovens, a rigidez de horários, a localização inadequada, mas também aspetos administrativos, financeiros, sociais, culturais e mesmo religiosos (APF, 2010). A falta de informação e os receios sobre a confidencialidade são também fatores que contribuem para o adiamento da procura de informação sobre contraceção, muitas vezes por vários meses, após o início das relações sexuais. Este direito e a forma como o devem assegurar tem que lhes ser transmitido pois muitos não o conhecem, um desconhecimento que continua a representar uma importante barreira no acesso a estes serviços (Ott, Sucato & Committee on Adolescence, 2014).

O acesso universal a consultas e métodos contracetivos constitui uma forma privilegiada de diminuir a gravidez indesejada e as IST (DGS, 2015) e a informação sobre a prevenção de comportamentos de risco para as IST, associada ao aconselhamento contracetivo pelos profissionais de saúde, são a chave para que os jovens possam fazer escolhas apropriadas (Alli et al., 2013; APF, 2015). A disponibilização destes serviços no *campus* universitário pode ajudar no esclarecimento e treino de competências relacionadas com a sexualidade e melhorar a adesão à contraceção e vigilância de SSR.

1.3.3. Determinantes dos comportamentos de risco e protetores para a saúde sexual e reprodutiva dos jovens

Os problemas de saúde relacionados com os estilos de vida e os comportamentos adquiriram uma especial relevância, reduzida que foi a magnitude das infeções contraídas de forma passiva. As questões de saúde devem ser abordadas numa perspetiva alargada, multidisciplinar, respeitando o conceito holístico de saúde ao longo do ciclo de vida, integrando a promoção da saúde nas suas vertentes de educação, prevenção e proteção, tendo em conta os múltiplos determinantes do comportamento de saúde (Rodrigues et al., 2005). As estratégias de promoção da saúde devem contemplar os múltiplos fatores determinantes da saúde, dado que é importante haver compreensão das circunstâncias em que as pessoas vivem e das escolhas que fazem para se tornarem mais saudáveis (OE, 2000).

A OMS (WHO, 1998) definiu como determinantes de saúde o conjunto de fatores pessoais, sociais, económicos e ambientais que, atuando em interação, influenciam ou afetam a saúde dos indivíduos, das famílias ou das comunidades. Todos os determinantes mencionados influenciam, num ou noutro sentido, o estado de saúde individual, familiar ou comunitária. Embora seja compreensível o sentido dessa influência, tem sido mais difícil ponderar o peso específico de cada um deles. De entre os determinantes da saúde, os

estilos de vida saudáveis ocupam um lugar de destaque pela aparente facilidade que apresentam na obtenção de mais ganhos em saúde (DGS, 2014).

Tendo em conta a temática em estudo, alguns determinantes a considerar podem ser organizados nas seguintes categorias: biológicos/individuais (e.g. idade, sexo); ambientais (ambiente social, suporte social) comportamentais (comportamento sexual, uso de contraceção, vigilância de saúde); e acesso aos serviços de saúde (educação, existência de serviços de SSR específicos para jovens, acessibilidade, serviços sociais).

No delineamento de programas de promoção da SSR dos jovens adultos, os profissionais de saúde devem considerar "fatores de risco e de proteção", caracterizados como fatores que estimulam ou desencorajam a adoção de comportamentos de risco e que a maior parte dos programas visa modificar. A presença ou ausência de fatores de risco pode ajudara a identificar quais os jovens em maior risco de ter comportamentos sexuais de risco (Kirby & Lepore, 2007; Mmari & Sabherwal, 2013). Os fatores biológicos/individuais e ambientais, tais como a idade, o género, o nivel educacional, o estatuto socioeconómico, o suporte social e até mesmo a religião, embora relacionados com os comportamentos, não são passíveis de ser alterados através de programas de promoção da saúde, mas são importantes para identificar os grupos em maior risco e direcionar as estratégias de intervenção de uma forma mais universal ou seletiva (Silva & Brito, 2014).

Alguns estudos (Kirby & Lepore, 2007; Kirby et al., 2011) têm vindo a identificar os fatores cognitivos e psicossociais – o conhecimento, a perceção de risco, as atitudes, as normas subjetivas, a autoeficácia e a intenção comportamental - como particularmente importantes, não só porque influenciam a tomada de decisão relacionada com os comportamentos sexuais, mas sobretudo porque são potencialmente modificáveis pela implementação de programas de promoção de comportamentos sexuais seguros. Embora não haja evidência de que todos estes fatores afetam o comportamento, alguns deles atuam sobre outros e indiretamente podem determinar o comportamento. Um modelo explicativo (Figura 2) é proposto por Kirby et al. (2011), onde demonstram como esses fatores podem estar interrelacionados e determinar os resultados em saúde, nomeadamente diminuir as taxas de gravidez indesejada e de transmissão de IST. Por exemplo, é comum acreditar que as intenções de realizar um comportamento, permite mais facilmente a sua concretização e, por sua vez, a atitude, a perceção de normas e a autoeficácia podem afetar as intenções comportamentais. Além disso, outros fatores, como o conhecimento, podem afetar as atitudes, perceções de normas e de autoeficácia.

No presente capítulo iremos fazer uma breve abordagem aos principais fatores que, de acordo com a evidência científica (Kirby et al., 2011; Mmari & Sabherwal, 2013, podem ser determinantes dos comportamentos de risco ou de proteção sexual em jovens adultos.

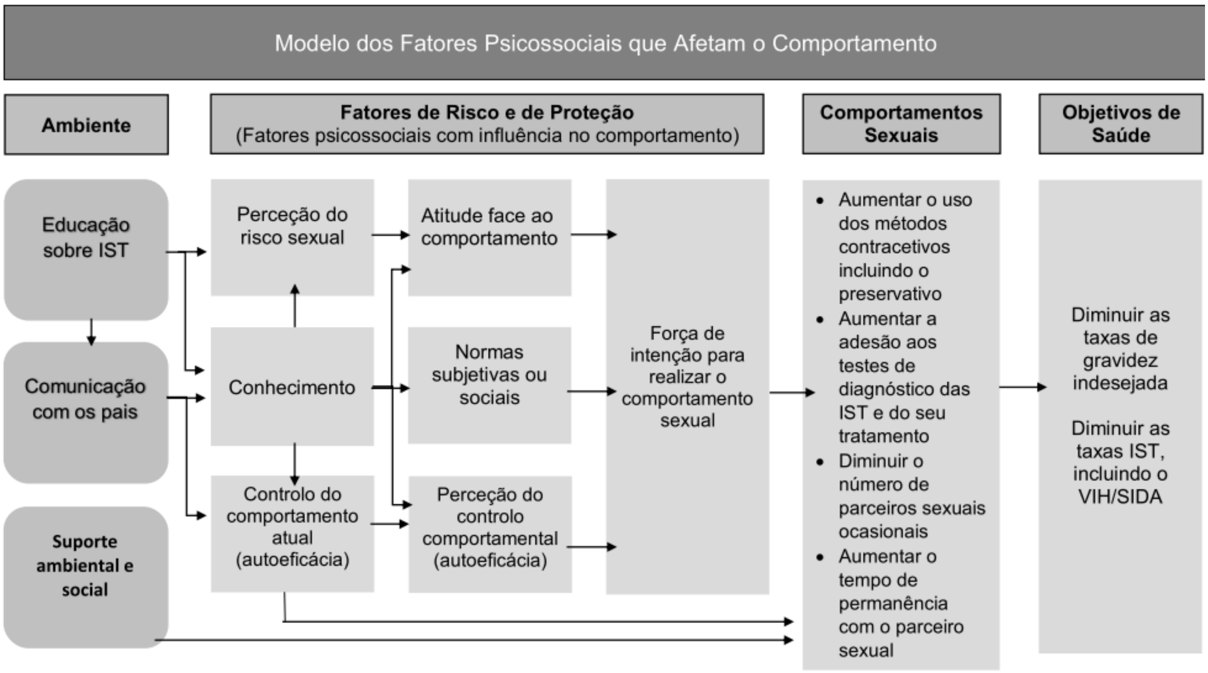

Figura 2. Modelo explicativo dos fatores psicossociais que afetam os comportamentos sexuais de risco (adaptado de Kirby et al., 2011)

Fatores individuais (idade e género)

O início da atividade sexual é um acontecimento normativo no ciclo de vida dos indivíduos, e na sociedade atual acontece frequentemente durante a juventude. Dados recentes sugerem que mais de metade dos estudantes universitários quando ingressa na universidade já é sexualmente ativa e teve a sua primeira relação sexual, em média, cerca dos 17 anos (Matos et al., 2011; Oliveira, 2014; Pacheco, 2012). As raparigas têm uma idade de iniciação sexual mais tardia que os rapazes, demonstrando uma atitude mais responsável e idealista da sexualidade (Antunes, 2007). A idade determina maior suscetibilidade biológica às IST, sobretudo no sexo feminino, resultado da maior sensibilidade do epitélio vaginal às infeções em idades precoces (Yi, Shannon, Prodger, McKinnon & Kaul, 2013). De acordo com Shneyderman e Schwartz (2012), as relações sexuais precoces aumentam o risco de contrair uma IST, dado que os indivíduos que iniciam a atividade sexual mais cedo referiram mais frequentemente que já tiveram uma IST.

O género é também um dos fatores a considerar no estudo dos comportamentos sexuais, uma vez que os jovens do sexo masculino têm sido sistematicamente identificados como tendo um risco acrescido, não só resultado dos processos de socialização e

desenvolvimento psicossocial, mas também da menor tendência para a adoção de comportamentos protetores de saúde e de procura de cuidados de saúde (Castro, 2017; Cragg, Steenbeek, Asbridge, Andreou & Langille, 2016). Contudo, embora as taxas de prevalência de IST, incluindo o VIH/SIDA, sejam elevadas entre adolescentes e jovens adultos (15-24 anos), elas são significativamente mais elevadas nas raparigas, decorrente da maior suscetibilidade biológica e vulnerabilidade social (Dellar, Dlamini & Karim, 2015; Workowski & Bolan, 2015). Esta preocupação tem despoletado alguma investigação no sentido de perceber o que potencia o risco biológico nas raparigas e que não pode ser justificado apenas pelo comportamento sexual. Relativamente ao VIH/SIDA, os estudos destacam o tempo de exposição da mucosa cervico-vaginal ao VIH (o sémen pode permanecer no trato genital feminino até três dias após o coito) (Kigozi et al., 2009), os níveis relativamente altos de ativação das células imunes no trato genital feminino, o aumento da expressão de corecetores do VIH em células cervicais em comparação com células do prepúcio (Yi et al., 2013), a superfície da mucosa vaginal mais propensa a adquirir micro-lesões durante o ato sexual (Stanley, 2009), assim como a existência de outras infeções vaginais (Cohen, 2004).

Fatores ambientais (suporte social)

Dentro dos fatores ambientais, podemos considerar a perceção de suporte social que parece ser importante para o desenvolvimento psicológico e adaptação dos indivíduos ao meio em que se relacionam com os outros. O suporte social tem sido alvo de múltiplas conceptualizações, desde aspetos mais operacionais, como o número de amigos, frequência e tipo de contactos que estabelecem numa rede de apoio social, até aspetos mais subjetivos relacionados com a perceção do tipo de apoio e satisfação com o apoio que o indivíduo recebe (Marôco, Campos, Vinagre & Pais-Ribeiro, 2014). A satisfação com o suporte social parece influenciar positivamente o bem-estar emocional dos jovens adultos, uma vez que este se percebe como mais disponível, amado, valorizado e reconhecido (Pinheiro & Ferreira, 2005; Ratelle, Simard & Guay, 2013). As qualidades das relações que se estabelecem, nomeadamente nas relações amorosas, também podem ser afetadas pelo suporte social, uma vez que são influenciadas pela rede social dos jovens, sobretudo a família e o grupo de pares (Sprecher, 2011). O suporte social representa, assim, um constructo complexo e multidimensional, que se relaciona com a saúde e bem-estar, permitindo perceber como os indivíduos enfrentam as situações de adversidade no decurso do seu desenvolvimento, incluindo os comportamentos de risco (Freitas & Mota, 2015; Marôco et al., 2014). Relativamente à influência do suporte social na compreensão dos comportamentos sexuais de risco, Kalina, Lukacs, Kriaucioniene e Orosova (2014), observaram que o suporte social estava associado a menores taxas de comportamentos

sexuais de risco, uma vez que os estudantes universitários com maior perceção de suporte social relataram um menor envolvimento com múltiplos parceiros sexuais, relações sexuais associadas a álcool e drogas e uso inconsistente do preservativo.

Fatores cognitivos

O conhecimento, embora por si só não determine o comportamento, é um pré-requisito importante porque fornece a fundamentação para muitos valores, atitudes, perceções de normas e competências que, por sua vez, permitem a concretização do comportamento (Kabwama & Berg-Beckhoff, 2015; Kirby et al., 2011). Contudo, um considerável número de estudos aponta para um desfasamento entre cognições, informação e comportamento, o que explica que pessoas com um nível adequado de informação sobre sexualidade nem sempre adotem comportamentos seguros (Kirby et al., 2011; Reis et al., 2013). Procurando perceber a relevância científica atribuída ao do conhecimento sobre SSR, Cassidy, Curran, Steenbeek & Langille (2015), realizaram uma revisão sistemática da literatura que incluiu 91 estudos na sua maioria de desenho transversal, publicados em língua inglesa entre 2000 e 2014. Os estudos investigaram o conhecimento dos estudantes universitários sobre VIH/SIDA (n=35), IST (n=23), HPV (n=9) e contraceção (n=24). A revisão destaca lacunas na literatura e nos resultados relacionados com a dominância da investigação dos estudos realizados nos EUA, e diferenças na conceção dos estudos e dos instrumentos de medição, o que dificulta a comparação de resultados.

Perceção de risco

A perceção do risco individual de contrair uma doença é fundamental para compreender as motivações que reduzem ou aumentam os comportamentos de risco. Alguns dos modelos cognitivo-comportamentais já descritos, consideram o risco percebido como um importante preditor do comportamento de risco. E embora o risco percebido não seja, por si só, suficiente para motivar uma mudança de atitude ou de comportamento, é visto como um fator necessário para operar a mudança comportamental (Asare, 2015; Kirby et al., 2011). Independentemente da perceção de invulnerabilidade ser adaptativa ou biologicamente determinada, as alterações na perceção de invulnerabilidade dos jovens face à gravidez indesejada e IST, pode reduzir os riscos associados aos comportamentos sexuais, uma vez que permite, por um lado, reconhecer as situações de risco e, por outro, fazer uma avaliação pessoal desse risco. Vários estudos e diferentes tipos de evidências demonstraram que os programas podem aumentar a perceção dos riscos e diminuir a possibilidade de relações sexuais desprotegidas (Kirby et al., 2011).

Atitudes

As atitudes podem explicar e prever os comportamentos em vários domínios e são uma componente importante que determina os comportamentos individuais de risco relacionados com a saúde (Albarracín, Johnson & Zanna, 2014; Pickens, 2011), revelando-se também como um dos preditores mais consistentes dos comportamentos de risco sexual (Ajzen & Fishbein, 2005; Albarracín, Johnson, Fishbein & Muellerleile, 2001; Alvarez, 2005), em particular na utilização do preservativo (Asare, 2015; Kang & Moneyham, 2007; Reis et al., 2013). Nas questões da sexualidade, as atitudes não são inteiramente determinadas pelo pensamento racional, sendo afetadas por sentimentos como o amor, a atração sexual, o medo, a insegurança e a invulnerabilidade. No entanto, as pessoas que têm uma "atitude negativa ou menos favorável" em relação a alguns comportamentos de risco, são menos propensas a envolver-se nesse comportamento.

Uma revisão integrativa da literatura de 14 estudos investigou a relação entre atitudes, valores e crenças e o comportamento sexual (Kirby & Lepore, 2007). Em 10 dos 14 estudos analisados, verificou-se que as atitudes estavam significativamente relacionadas com a iniciação sexual. Quarenta e seis estudos mediram o impacto de atitudes sobre uso do preservativo e contracetivos nas relações sexuais atuais e, destes, 30 estudos evidenciaram que as atitudes estavam relacionadas com o uso real do preservativo e anticoncecionais. Esta ausência de significado estatístico pode estar relacionada com limitações metodológicas ou refletir que as atitudes favoráveis ao uso do preservativo nem sempre estão relacionadas com o seu uso efetivo por parte dos jovens.

Normas subjetivas

Vários estudos têm demonstrado que as perceções que os jovens têm das normas dos seus pares sobre a atividade sexual afetam o seu comportamento sexual e contracetivo. Este facto resulta não só do desejo de estar em conformidade com normas sociais (normas de comportamento aceitável), mas também porque estão explicitamente incentivados a fazê-lo. As normas sociais são criadas pelo comportamento real e crenças sobre o que esse comportamento deve ser, no entanto, também reconhece que as perceções das normas sociais das pessoas podem diferir das normas sociais reais, o que é visível quando as pessoas muitas vezes exageram a extensão com que os outros se envolvem em comportamentos de risco (Kirby et al., 2011). No grupo dos jovens adultos, as abordagens para melhorar os comportamentos de promoção da saúde devem concentrar-se nos grupos de pares, uma vez que os valores, as atitudes, crenças e comportamentos dos colegas/ amigos influenciam o seu estilo de vida (Black, Schmiege & Bull, 2013). As atitudes dos pares ou a perceção de que estes valorizam e utilizam o preservativo também constituem,

para os estudantes universitários, um preditor poderoso da sua utilização, em particular a perceção de que este comportamento é aprovado pelo parceiro sexual (Asare, 2015). No entanto, para a população em geral, estas normas influenciam mais o uso do preservativo com parceiros casuais ou secundários do que com parceiros primários (Alvarez, 2005).

Uma revisão de 15 estudos que mediram o impacto das perceções de normas de pares sobre o comportamento sexual ou perceções dos pares sobre o uso do preservativo, 12 estudos encontraram uma relação significativa entre as perceções de normas ou comportamento dos pares com o uso efetivo do preservativo (Kirby & Lepore, 2007).

Autoeficácia

A autoeficácia é o julgamento ou a confiança das pessoas na sua capacidade de realizar bem determinadas habilidades ou comportamentos. Pessoas com alta autoeficácia têm maior confiança na sua capacidade executar com sucesso determinados comportamentos e alcançar um objetivo (Bandura, 2006). Vários estudos têm demonstrado que a capacidade dos jovens para evitar atividades sexuais que comportam risco está relacionada com a autoeficácia para recusar o sexo ou a usar preservativo ou outro método contracetivo, na medida em que a convicção de realizar com sucesso o comportamento necessário mostrou-se um indicador importante da probabilidade do indivíduo adotar o comportamento de proteção (Artistico et al., 2014; Asante & Daku, 2010; French & Holland, 2013). No que se refere à utilização do preservativo, a sua caracterização multidimensional permite incluir vários aspetos neste conceito, nomeadamente a obtenção, a capacidade de negociação e a utilização, componentes importantes da autoeficácia para usar o preservativo (Brafford & Beck, 1991).

Uma revisão de diversos estudos, onde foi avaliada a relação entre fatores de risco e de proteção do comportamento sexual de jovens, conclui que dos 14 estudos que avaliaram a relação entre a autoeficácia para usar o preservativo e o seu uso real, 13 encontraram relações significativas (Kirby & Lepore, 2007). Alguns autores mencionam ainda que níveis elevados de autoeficácia permitem não só aumentar o uso do preservativo, mas também ultrapassar barreiras na sua aquisição e uma utilização mais correta, com diminuição do risco de IST e gravidez indesejada (Artistico et al., 2014; French & Holland, 2013).

Busca de sensações sexuais

Mais recentemente, alguns investigadores têm também estudado a influência dos traços de personalidade na adoção de comportamentos de risco, que apontam a personalidade com disposição para a busca de sensações sexuais, como importante na compreensão dos comportamentos de risco sexual (Pechorro et al., 2016; Sales et al., 2013; Teva, Bermúdez & Buela-Casal, 2010; Voisin King, Schneider, Diclemente & Tan, 2012). Os estudos têm

revelado que os indivíduos com níveis superiores de busca de sensações sexuais apresentam uma atitude de maior permissividade sexual (Gaither & Sellbom, 2003; Hendershot, Stoner, George & Norris, 2007), maior consumo de álcool ou drogas associado a relações sexuais e maior risco de transmissão de IST (Gullette & Lyons, 2006).

1.4. Saúde sexual e reprodutiva como foco de atenção em enfermagem

A saúde SSR é uma área fundamental dos cuidados de saúde e deve estar disponível a todos os indivíduos numa resposta abrangente e integradora, visando alcançar elevados níveis de saúde e bem-estar, na interdependência de três pilares fundamentais, a saber: i) o acesso à informação sobre a sexualidade, o conhecimento sobre os riscos que as pessoas enfrentam e as consequências adversas de alguns tipos de atividade sexual; ii) o acesso a serviços e cuidados de SSR de qualidade, num ambiente que afirme e promova a saúde sexual; iii) a construção e implementação de politicas de promoção da SSR à escala nacional, atendendo às desigualdades e vulnerabilidades da população (RCN, 2001; WHO, 2011).

A promoção da SSR dos jovens numa perspectiva educacional deve contemplar o desenvolvimento de uma sexualidade esclarecida e saudável, onde a responsabilidade individual e a tomada de decisão assumem um papel determinante na garantia de comportamentos sexuais positivos para a saúde e para a qualidade de vida dos jovens (Dias, 2009). Para uma vivência saudável da sexualidade, é importante que os jovens estejam informados e conscientes acerca de todos os aspetos relacionados, nomeadamente os afetivos, biológicos, as consequências da atividade sexual e as principais medidas de proteção. Promover a SSR implica valorizar as suas diferentes dimensões: ética, afetiva e relacional, o que não minimiza a expressão da sexualidade, assim como a motivação para a proteção face a situações de risco sexual não reprime os afetos nem o erotismo (Sampaio, 2006).

Face ao processo de liberalização das normas sociais relativas à sexualidade, os enfermeiros desempenham um papel crucial na educação sexual dos jovens, não só no sentido de potenciar a sua capacidade de escolha e melhorar a qualidade do uso dos contracetivos, mas também alertando para a importância do sexo seguro como forma de minimizar o risco associado às IST e a importância da vigilância regular da SSR (Barros & Neves, 2013). Os enfermeiros devem entender a educação sexual na sua complexidade que inclui as dimensões, biológica, psicológica e sociocultural, integrando nos cuidados de enfermagem o domínio cognitivo (informação), um domínio afetivo (sentimentos, valores e atitudes) e um domínio comportamental (comunicação, tomada de decisões e outras

competências pessoais relevantes). A EpS, nesta área, deverá pressupor ainda uma comunicação eficaz entre o enfermeiro e o/a jovem, sustentada em bases científicas e legais sólidas, numa relação baseada na confiança e no respeito para que se crie um ambiente de segurança, intimidade e privacidade, evitando juízos de valor e discriminações e salvaguardando sempre a qualidade na prestação dos cuidados de enfermagem e todas as orientações éticas e deontologias inerentes à profissão, tal como preconizado no Regulamento do Exercício Profissional dos Enfermeiros (OE, 1996). O contexto e o ambiente de privacidade propício permitirão o desenvolvimento da consulta de enfermagem, onde as mensagens devem ser claras e objetivas, a informação fornecida fundamentada e discutida, e a escuta ativa pode ser uma ferramenta fundamental (Carteiro & Nené, 2015).

No exercido das suas competências, os enfermeiros em complementaridade com a equipa médica, integram os serviços de planeamento familiar, uma componente fundamental da prestação de cuidados em saúde reprodutiva. Ao nível dos cuidados de saúde primários quer os enfermeiros generalistas nas unidades de saúde familiar ou de cuidados de saúde personalizados, quer os enfermeiros especialistas em saúde materna e obstetrícia (EESMO) integrados nesta unidades ou nas unidades de cuidados na comunidade, desenvolvem diversas atividades que permitem assegurar as necessidades de cuidados nesta área. De salientar que, em particular, o EESMO tem o seu campo de intervenção bem definido no regulamento de competências do EESMO (Regulamento n.º 127/2011, de 18 de fevereiro), onde se prevê de forma clara que este assuma responsabilidades de intervenção na área da sexualidade e do planeamento familiar. O regulamento supracitado explicita que são competências específicas do EESMO a intervenção na área da SSR, nomeadamente ao nível da "assistência à mulher em idade fértil, atuando no ambiente em que vivem e se desenvolvem no sentido de promover a SSR e prevenir os processos de doença" (p.8662). Assim o EESMO pelos conhecimentos técnicos e científicos especializados e pela relação de proximidade que estabelece com os indivíduos/ família ao longo do ciclo vida reprodutiva, constitui-se como uma mais-valia no aconselhamento, diagnóstico precoce e prevenção do risco e de complicações no âmbito da saúde sexual e planeamento familiar.

Na faixa etária dos adultos jovens, dos objetivos propostos pela DGS (2008) para o planeamento familiar, entendemos como essenciais: i) promover a vivência da sexualidade de forma saudável e segura; ii) regular a fecundidade segundo o desejo do casal; iii) preparar para a maternidade e a paternidade responsáveis; iii) reduzir a incidência das ITS e as suas consequências, designadamente, a infertilidade; iv) melhorar a saúde e o bem-estar dos indivíduos.

A consulta de enfermagem de SSR representa ainda uma oportunidade para: i) avaliar os antecedentes pessoais e familiares, incluindo a história sexual e reprodutiva e parâmetros

de saúde, estando recomendado nesta faixa etária apenas a avaliação da tensão arterial e o índice de massa corporal (IMC); ii) promover uma atitude positiva face à sexualidade, numa visão integrada da sexualidade e reprodução; iii) reforçar o conhecimento sobre a anatomofisiologia da reprodução, métodos contracetivos, sinais e sintomas de IST e medidas de prevenção; iv) promover uma vivência saudável e segura da sexualidade; com sensibilização para comportamentos protetores, tais como, a limitação do número de parceiros sexuais e conhecimento da história sexual do parceiro, utilização correta e consistente do preservativo, uma vez que apenas o preservativo ou a dupla proteção são eficazes na prevenção da gravidez e das IST e a vacinação pré-exposição no caso do HPV; v) fornecer métodos contracetivos eficazes, seguros e gratuitos e informação adequada sobre os mesmos; vi) sensibilizar para a importância da vigilância regular da SSR, com recomendação do autoexame da mama e do testículo, rastreio do cancro da mama e do colo do útero com realização de citologia vaginal; vii) nos jovens sexualmente ativos, encaminhar para o médico para avaliar a necessidade de rastreio de IST mais prevalentes nesta faixa etária, uma vez que alguns estudos epidemiológicos recentes têm apontado como recomendação a realização de testes anuais de pesquisa de *Chlamydia* e *Neisseria Gonorrhoeae* em jovens sexualmente ativos até aos 25 anos (Mansouri & Santos, 2014); viii) promover a adoção de estilos de vida saudáveis.

Refletindo sobre os estereótipos de género que, nesta área da saúde, ocupam a máxima expressão, é visível que os serviços e cuidados de SSR estão essencialmente vocacionados para o género feminino. A este propósito, Prazeres (2003) menciona que, "Tradicionalmente não existem estruturas de saúde pública orientadas para as necessidades reprodutivas dos homens, nomeadamente para aspectos de prevenção de situações que envolvem risco" (p.55). Neste sentido, deve ser considerada uma adequação da estrutura organizativa dos cuidados de SSR, orientada para as questões de género.

O aconselhamento contracetivo dos jovens

No âmbito da promoção da SSR, o bom aconselhamento contracetivo e a escolha do método contracetivo garantem uma maior adesão e continuidade à utilização de contraceção. Assim, a disponibilização de métodos contracetivos que respondam às necessidades específicas de cada utente na sua diversidade e características individuais constitui um importante mecanismo de melhoria dos cuidados de SSR prestados (DGS, 2015). O aconselhamento contracetivo pode ser realizado por profissionais de saúde (médico ou enfermeiro) e pressupõe uma abordagem centrada na pessoa, envolvendo-a no processo de decisão, que de acordo com a DGS (2010), deve ter em linha de conta as seguintes orientações: i) prestar informação clara sobre os métodos contracetivos disponíveis, sobre os efeitos secundários e a forma correta de utilização; ii) saber ouvir e

esclarecer dúvidas; iii) encontrar e trabalhar no aconselhamento a motivação para iniciar e/ou realizar contraceção correta e consistentemente; iv) assegurar a disponibilidade de meios contracetivos; v) e manter o investimento na formação dos profissionais de saúde.

Desta forma, o aconselhamento contracetivo deve ser integrado no âmbito da promoção de um estilo de vida saudável, sendo a escolha contracetiva realizada de forma multidisciplinar, em resultado de uma avaliação adequada ao estado de saúde e tendo em conta as recomendações presentes nos critérios médicos de elegibilidade para o uso de contracetivos (WHO, 2004). A intervenção deve estar orientada simultaneamente para a promoção da saúde sexual, prevenção de IST e tratamento dos problemas ginecológicos/andrológicos comuns nesta faixa etária (Ott et al., 2014). Atendendo às características dos jovens adultos, idealmente o método contracetivo deverá ser seguro, eficaz, reversível, pouco dispendioso, discreto e com poucos efeitos colaterais (Barros & Neves, 2013). Nesta escolha é recomendada a utilização de uma abordagem "em camadas", começando por ponderar os métodos mais eficazes, uma eficácia que é calculada em função de dois termos distintos - o "uso perfeito" e o "uso típico" (Ott et al., 2014).

Uma vez que a idade, por si só, não constitui contraindicação à utilização de qualquer método contracetivo e a maioria dos métodos pode ser usada pelos jovens sem restrições, entre eles, pode contar-se com a contraceção hormonal combinada (oral, transdérmica, anel vaginal), de elevada eficácia contracetiva e com benefícios não contracetivos como a regularização dos ciclos menstruais, a diminuição da dismenorreia, melhoria da acne e prevenção dos quistos funcionais do ovário. No entanto, a sua utilização deve ser sempre complementada com o uso do preservativo masculino ou feminino, uma vez que nenhum destes contracetivos protege de IST. Nas raparigas mais novas ou com maior IMC, é dada preferência às pílulas com menores níveis de estrogénios por terem menor impacto sobre a densidade mineral óssea. O implante contracetivo tem uma elevada eficácia, associada à comodidade posológica, pelo que representa uma alternativa viável, particularmente em adolescentes que pretendem uma contraceção eficaz de longa duração ou perante a dificuldade na utilização de outros métodos (Ott et al., 2014). Dada a elevada eficácia, dos denominados métodos reversíveis de longa duração, onde se incluem os implantes contracetivos, anel vaginal e a contraceção intrauterina, tem-se observado uma mudança de paradigma estendendo as indicações para estes métodos a mulheres de todas as idades, inclusive às adolescentes (DGS, 2015).

O aconselhamento e disponibilização da contraceção de emergência devem fazer parte de uma orientação antecipatória e ser sempre acompanhada da mensagem de que, embora este método não tenha contraindicações a sua eficácia contracetiva é inferior à de qualquer

um dos métodos de contraceção regular (Barros & Neves, 2013; International Consortium for Emergency Contraception [ICEC], 2015). Mas, a necessidade desta contraceção não deve deixar de constituir uma oportunidade para aconselhar a jovem a iniciar uma contraceção regular, sendo que o início de um contracetivo deve ser imediato à contraceção de emergência (DGS, 2015).

Quando usados de forma correta e consistente, os preservativos são um método seguro para prevenir uma gravidez indesejada. É um método de barreira local, não hormonal e na maioria dos casos não existe qualquer efeito secundário para a sua utilização. O uso do preservativo não necessita de qualquer preparação sendo essencial para uma contraceção eficaz em relações inesperadas. Tem, além disso, a grande vantagem acrescida da prevenção das infeções de transmissão sexual, podendo ser utilizado conjuntamente com qualquer outro método (DGS, 2015). Os métodos naturais não são recomendados nos adolescentes, pela sua baixa eficácia, reduzida taxa de continuação do método, agravada nestas idades pelas irregularidades do ciclo menstrual e baixa previsibilidade da ovulação nos primeiros anos após a menarca (Pacheco et al., 2011). Antes de iniciarem a contraceção, os jovens devem recorrer aos serviços de saúde para atendimento especializado e a avaliação da tensão arterial e do IMC, sem necessidade de exames complementares de diagnóstico (Neto, Bombas, Arriaga, Almeida & Moleiro, 2012).

A posterior manutenção da vigilância em consultas de acompanhamento é imprescindível para maximizar a adesão aos métodos de contraceção, promover e reforçar a tomada de decisão (o uso, efeitos adversos e complicações) e implementação de outras medidas protetoras, como a imunização para HPV e/ou despiste periódico de comportamentos de risco e IST, dada a natureza intrínseca dos jovens para se exporem ao risco. O momento e a frequência da reavaliação dependem do método contracetivo utilizado e de outras necessidades de saúde do jovem adulto (Santos & Figueiredo, 2015). Assim, a educação contracetiva dos jovens pelos profissionais de enfermagem revela-se particularmente importante, podendo potenciar a sua capacidade de escolha e melhorar a qualidade do uso dos contracetivos, nomeadamente a sua efetividade e consistência de utilização.

Considerando a abordagem holística que caracteriza os cuidados de enfermagem, para além do aconselhamento contracetivo deve ser realizado também o aconselhamento sobre prevenção de comportamentos de risco para aquisição de IST, recomendado a todos os jovens sexualmente ativos, e em particular em jovens com história clinica de IST no passado ou com múltiplos parceiros sexuais. O aconselhamento individual, a entrevista motivacional e as mensagens de prevenção de riscos mesmo que breves, têm-se revelado eficazes na prevenção de IST (Workowski & Bolan, 2015).

CAPÍTULO 2

METODOLOGIA GERAL

No âmbito da revisão da literatura realizada no decurso deste trabalho, os estudos sobre os comportamentos sexuais e reprodutivos em jovens adultos evidenciam que os jovens do ensino superior mantêm comportamentos de risco sexual, que resultam da interligação complexa entre diversos fatores de âmbito individual, cognitivo, psicossocial e mesmo contextuais. Esta investigação assume-se como um contributo para aprofundar o conhecimento e a compreensão sobre os comportamentos de SSR entre os estudantes do ensino superior, o que permitirá traçar linhas de orientação e estruturar estratégias para o desenvolvimento de programas de promoção da SSR nesta população, e onde os enfermeiros integrados na equipa multidisciplinar, pela especificidade da sua formação, devem assumir um papel relevante.

2.1. Objetivos do estudo

Os objetivos gerais da presente investigação são:

i) Analisar a relação entre o conhecimento, as atitudes e os comportamentos de SSR de estudantes do ensino superior;

ii) Caracterizar as práticas contracetivas dos estudantes do ensino superior e identificar fatores relacionados com as escolhas contracetivas, atendendo ao género;

iii) Identificar fatores relacionados com a proteção e o risco sexual e reprodutivo em estudantes do ensino superior.

iv) Identificar as barreiras que os estudantes do ensino superior encontram no acesso aos serviços e cuidados de SSR;

v) Identificar fatores preditores do uso do preservativo em estudantes do ensino superior;

vi) Compreender as representações e perceções que medeiam a adoção de comportamentos de risco sexual e reprodutivo, explorando o papel das barreiras e dos benefícios, da autoeficácia, dos sentimentos e das influências interpessoais e situacionais;

vii) Proceder à adaptação cultural e validação de instrumentos psicométricos, com relevância para o estudo da área da sexualidade e saúde reprodutiva nesta população.

2.2. Desenho geral do estudo

No quadro conceptual procurou-se enquadrar a temática e apresentar uma visão geral dos múltiplos fatores e contextos que determinam os comportamentos sexuais e reprodutivos dos estudantes do ensino superior, e que serviu de eixo norteador os objetivos gerais do estudo. Em termos metodológicos, o presente estudo configura-se como um estudo de

natureza mista, uma vez que compreende uma abordagem quantitativa e qualitativa, e cujas etapas de desenvolvimento se encontram sistematizadas na Figura 3. A opção pela triangulação de metodologias, com combinação de métodos, teve como propósito clarificar e compreender alguns dos resultados obtidos na primeira fase do estudo, uma vez que a triangulação dos métodos é, habitualmente, usada no estudo de conceitos complexos (Fortin, Côté & Filion, 2009), nos quais podemos incluir o estudo dos comprtamentos sexuais e reprodutivos.

No delineamento de programas de intervenção para promoção de comportamentos sexuais seguros é essencial o rigor da avaliação dos comportamentos sexuais e das estratégias a utilizar, pelo que se revela importante a utilização de instrumentos adequados aos objetivos e população a estudar. Neste sentido, numa primeira fase procedemos a uma investigação dos instrumentos já existentes, que permitissem medir as variáveis que pretendíamos estudar, dos quais, dois foram validados e adaptados para a população em estudo (CUSES-RP e SSSS-P) e à construção de outros (ICSSR e EASSSR), quando os consideramos que os existentes não se adequavam aos objetivos do estudo. Do estudo quantitativo resultaram três artigos metodológicos e cinco artigos sobre questões relacionadas com a SSR dos estudantes do ensino superior.

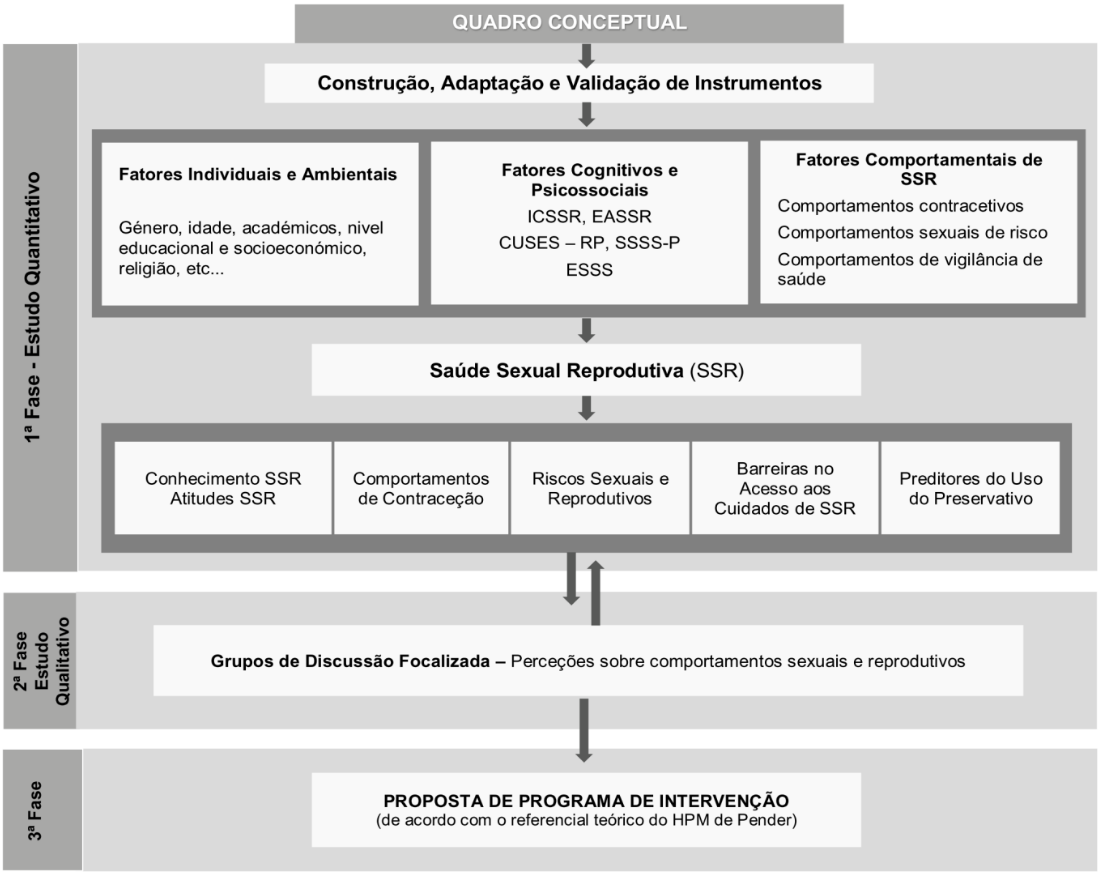

Figura 3. Desenho geral do estudo

Numa segunda fase, procedemos à realização de dois grupos focais, objetivando compreender algum dos resultados obtidos na primeira fase do estudo e incrementar a consistência dos mesmos. Finalmente, na terceira fase, decorrente da triangulação dos resultados dos dois estudos, apresenta-se a proposta de um programa de intervenção, onde o HPM de Pender foi usado como referencial organizativo das medidas avançadas para promover comportamentos sexuais e seguros.

2.3. Estudo quantitativo

Neste capítulo apresenta-se a informação geral sobre os procedimentos metodológicos que suportaram o estudo quantitativo, no que concerne ao tipo de estudo, amostragem realizada, instrumentos de colheita de dados e processo de aplicação, análise estatística e considerações éticas. A descrição dos objetivos específicos, das variáveis e do papel que desempenham na investigação, será apresentada em cada um dos artigos que compõem o capítulo dos estudos empíricos.

2.3.1. Tipo de estudo

O presente estudo é de natureza transversal, observacional, e do tipo descritivo-correlacional, com uma componente analítica. A opção por este tipo de estudo, relaciona-se com o facto de entendermos como metodologicamente mais adequado ao estudo das relações entre as varáveis que consideramos pertinentes estudar.

Nos estudos observacionais (não experimentais), o investigador estuda, observa e regista o fenómeno em estudo e os seus atributos, e a forma como este se relaciona com outras condições/atributos sem ter qualquer intervenção. Por sua vez, os estudos transversais analisam a relação entre o fenómeno em estudo e/ou outra condição de interesse e outras características da população num determinado tempo e lugar (Porta, 2008).

Apesar de reconhecermos inúmeras vantagens aos estudos longitudinais de carácter experimental, nomeadamente na demonstração da relação causa-efeito, a opção pelo desenho transversal reflete as condições de realização e as limitações temporais às quais esta investigação esteve sujeita, bem como as limitações metodológicas, logísticas e económicas.

2.3.2. População e amostra

Para realizar o estudo quantitativo utilizou-se uma amostra não probabilística de conveniência, constituída por 1 946 estudantes (cerca de 30% da população dos estudantes

de 1.º ciclo e mestrado integrado), num total de 1 946 estudantes que frequentavam cursos das cinco escolas da universidade onde decorreu o estudo, no ano letivo 2012/2013. A técnica de escolha da amostra foi a amostragem por grupos - *Cluster sampling* - onde o grupo ou a unidade de amostra foi a turma. As turmas foram agrupadas com base na representação das áreas científicas e os diferentes anos do curso.

O cálculo do tamanho amostral teve em consideração os pressupostos de Gil (1999) e Richardson (1999), sendo que para populações finitas, com um nível de confiança de 95%, a amostra deveria ser constituída no mínimo por 904 estudantes, considerando um universo populacional de aproximadamente 6 500 estudantes. Foram considerados como critérios de inclusão os estudantes dos cursos de 1.º ciclo e mestrado integrado das cinco escolas da universidade, com idade igual ou superior a 18 anos e inferior a 30 anos, e que aceitaram participar voluntariamente ao estudo, depois de conhecerem os objetivos do mesmo.

Caracterização da amostra

Dos estudantes que participaram no estudo, 1 246 (64%) eram do género feminino e 700 (36%) do género masculino. A média de idades dos participantes era 21 anos (20,74±2,32), sendo que os rapazes eram em média mais velhos (21,21±2,63) do que as raparigas (20,74±2,08), com diferenças estatisticamente significativas entre os grupos (p≤0,001). Os coeficientes de variação indicam uma dispersão baixa face às idades médias encontradas (Tabela 1).

Tabela 1.
Estatísticas relativas à idade dos participantes

Variáveis	N	Mínimo	Máximo	Média	DP	Teste t	IC 95%	
							Inferior	Superior
Masculino	700	18	29	21,21	2,63	6,776 p≤ 0,001	21,01	21,41
Feminino	1246	18	29	20,48	2,08		20,36	20,59
**Total**	1946	18	29	20,74	2,32		20,64	20,84

Do agrupamento da idade em classes, 31,8% dos participantes apresentava idade inferior ou igual a 19 anos, 61,0% entre 20 e 24 anos e 7,1% com idade superior ou igual a 25 anos. A maioria dos participantes era de nacionalidade portuguesa (97,3%) e solteira (97,6%). Cerca de 40% dos estudantes referem ter crescido na cidade, 38% na aldeia e 21,9% numa vila. Para efeitos de análise de dados, a origem de uma cidade ou vila foi agrupada em "meio urbano" e de uma aldeia em "meio rural".

Na distribuição dos estudantes por área científica de formação, observou-se que a maioria pertencia às quatro escolas de natureza universitária (29,2% à Escola das Ciências da Vida, 21,6% à Escola das Ciências Agrárias e Veterinárias, 21,2% Escola das Ciências Humanas e Sociais, 15,4% à Escola das Ciências e Tecnologias) e 12,5% à Escola de Enfermagem, esta última de natureza politécnica. Para efeitos de análise de dados, considerou-se os

estudantes que frequentavam cursos da área das "Ciências da Vida e Saúde" e os estudantes que frequentavam cursos da área das "Ciências Humanas e Sociais, e Tecnologias", na medida em que os cursos que integram a primeira têm nos conteúdos programáticos várias matérias relacionadas com a reprodução humana e a saúde.

A distribuição por ano de curso foi bastante homogénea (28,1% dos estudantes são do 1.º ano, 31% do 2.º ano, 32,9% do 3º ano), com exceção para o 4.º ano, que inclui os estudantes da licenciatura em Enfermagem e mestrado integrado em Medicina Veterinária (4,7%) e 5.º ano, que inclui apenas os estudantes do mestrado integrado da Medicina Veterinária (3,3%). Relativamente à importância da religião, metade dos estudantes (50,8%) refere que a religião é muito importante ou importante na sua vida.

No que se refere à profissão dos pais, e tendo por base a classificação nacional de profissões, no caso dos pais predominam as profissões pouco diferenciadas, relacionadas com serviços e vendedores (20,5%), operários e artífices e trabalhadores similares (15,2%), e as profissões mais diferenciadas, no caso das mães, predominam as especialistas das profissões intelectuais e científicas (23,5%) e as profissões relacionadas com serviços e vendedores (23,5%). Existe, ainda, uma percentagem de estudantes que refere que os pais se encontram desempregados (3% dos pais e 2,6% das mães). O rendimento familiar é baixo, sendo que 57% dos estudantes refere um rendimento familiar inferior ou igual a dois salários mínimos. O nível de escolaridade dos pais é também, em média, baixo, cerca de metade das mães (54,5%) e dos pais (61,6%) têm apenas nove anos de escolaridade.

Tabela 2.
Caracterização sociodemográfica e académica da amostra em função do género

**Variáveis**	**Masculino** n=700	**Feminino** n=1246	**Total** n=1946
	**nº %**	**nº %**	**nº %**
**Classe etária**			
Menor ou igual a 19	195 (27,9)	424 (34,0)	620 (31,8)
20- 24 anos	424 (60,7)	763 (61,3)	1187 (61,0)
Igual ou superior 25	80 (11,4)	59 (4,7)	139 (7,1)
Nacionalidade			
Portuguesa	673 (96,1)	1215 (97,5)	1888 (97,1)
Brasileira	10 (1,0)	12 (1,0)	22 (1,1)
Espanhola	3 (0,4)	4 (0,3)	7 (0,4)
Outra	14 (2,5)	15 (1,2)	29 (1,5)
Ano de curso			
1.º Ano	210 (30,0)	337 (27,0)	547 (28,1)
2.º Ano	215 (30,7)	388 (31,1)	603 (31,0)
3.ª Ano	240 (34,3)	400 (32,1)	640 (32,9)
4.ª Ano	20 (2,9)	72 (5,8)	92 (4,7)
5.º Ano	15 (2,1)	49 (3,9)	64 (3,3)
Escola			
Escola das Ciências da Vida	150 (21,4)	270 (21,7)	569 (29,2)
Escola das Ciências Agrárias e Veterinárias	217 (31,0)	353 (28,3)	420 (21,6)
Escola das Ciências Humanas e Sociais	199 (28,4)	101 (8,1)	413 (21,2)
Escola das Ciências e Tecnologias	84 (12,0)	329 (26,4)	300 (15,4)
Escola de Enfermagem	50 (7,1)	193 (15,5)	243 (12,5)

Tabela 2.
Caracterização sociodemográfica e académica da amostra em função do género (continuação)

Zona habitacional onde cresceu			
Aldeia	246 (35,1)	496 (39,8	740 (38,0)
Vila	166 (23,7)	261 (20,9)	427(21,9)
Cidade	288 (41,1)	489.(39,2)	767 (39,9)
Estado civil			
Solteiros	677 (96,7)	1223 (98,2)	1900 (97,6)
Casados	10 (1,4)	14 (1,1)	24 (1,2)
União facto	9 (1,3)	8 (0,6)	17 (0,9)
Divorciados	4 (0,6)	1 (0,1)	5 (0,3)
Importância religião			
Muito Importante ou Importante	309 (44,1)	1223 (54,6)	489 (50,8)
Pouco ou nada importante	298 (42,6)	14 (37,9)	770 (39,6)
Sem religião	93 (13,3)	8 (7,5)	187 (9,6)
Escolaridade mãe			
Ensino básico	338 (48,3)	723 (58,0)	1061 (54,5)
Ensino secundário	188 (26,9)	300 (24,1)	488 (25,1)
Ensino superior	174 (24,9)	223 (17,9)	397 (20,4)
Escolaridade pai			
Ensino básico	400 (57,6)	799 (64,9)	1199 (61,6)
Ensino secundário	172 (24,8)	278 (22,6)	450 (23,1)
Ensino superior	122 (17.6)	154 (12,5)	276 (14,2)
Profissão da mãe			
Quadro superior da administração pública, dirigentes e quadros superiores	29 (5,1)	26 (2,7)	55 (3,6)
Especialista das profissões intelectuais e científicas	147 (25,7)	210 (22,1)	357 (23,5)
Técnicas e profissionais de nível intermédio	21 (3,7)	51 (5,4)	72 (3,7)
Pessoal administrativo e similares	54 (9,4)	76 (8,0)	130 (8,5)
Pessoal dos serviços e vendedoras	117 (20,5)	241 (25,4)	358 (23,5)
Agricultora e trabalhadores qualificados	6 (1,0)	14 (1,5)	20 (1,3)
Operária, artífices e trabalhadores similares	34 (5,9)	73 (7,7)	107 (7,0)
Operadora de instalações e máquinas	-	5 (0,5)	5 (0,3)
Trabalhadora não qualificada	71 (12,4)	150 (15,8)	221 (14,5)
Doméstica	72 (12,6)	78 (8,2)	150 (9,9)
Reformada	6 (1,0)	1 (0,1)	7 (0,5)
Desempregada	15 (2,6)	25 (2,6)	40 (2,6)
Profissão do pai			
Forças Armadas	6 (2,0)	3 (0,7)	9 (1,3)
Quadro superior da administração pública, dirigente e quadro superior	41 (13,9)	46 (11,2)	87 (12,3)
Especialista das profissões intelectuais e científicas	34 (11,6)	54 (13,1)	88 (12,5)
Técnico e profissionais de nível intermédio	32 (10,9	36 (8,7)	68 (9,6)
Pessoal administrativo e similares	3 (0,1)	5 (1,2)	8 (1,1)
Pessoal dos serviços e vendedores	55 (18,7)	90 (21,8)	145 (20,5)
Agricultor e trabalhadores qualificados	10 (3,4)	23 (5,6)	33 (4,7)
Operário, artífice e trabalhadores similares	38 (12,9)	69 (16,7)	107 (15,2)
Operador de instalações e máquinas	12 (4,1)	21 (5,1)	33 (4,7)
Trabalhador não qualificado	38 (12,9)	42 (10,0)	80 (11,3)
Reformado	14 (4,8)	13 (3,2)	27 (3,8)
Desempregado	10 (3,4)	11 (2,7)	21 (3,0)
Rendimento familiar			
Menos de 2 salários mínimos	133 (19,0)	241 (19,3)	374 (19,2)
2 Salários mínimos	218 (31,1)	518 (41,6)	736 (37,8)
4 Salários mínimos	211 (30,1)	337 (27,0)	548 (28,2)
Mais de 4 salários mínimos	138 (19,7)	150 (12,0)	288 (14,8)

2.3.3. Procedimentos de recolha de dados

Após aprovação do estudo de investigação pela Comissão de Ética da universidade, foi solicitada autorização aos Presidentes das cinco escolas que a compõem (Escola das Ciências da Vida, Ciências Agrárias e Veterinárias, Ciências Humanas e Sociais, Ciências e Tecnologias e Enfermagem), para aplicação do protocolo de investigação *(Apêndice A)*. Obtida a autorização destes, foram contactados os diretores dos diferentes cursos e docentes e foram agendadas as datas de aplicação dos instrumentos de colheita de dados. Os dados do estudo quantitativo foram recolhidos entre fevereiro e maio de 2013.

A recolha de dados foi realizada em sala de aula, através de um questionário de autopreenchimento, em papel, cujo preenchimento demorou, em média, 50 minutos, sendo aplicado pelo investigador ou por um colaborador devidamente preparado para o efeito. Antes do início do preenchimento do questionário, foi realizada a leitura do texto de apresentação e instruções de preenchimento que se encontravam na folha de rosto do mesmo. Foram descritos os objetivos gerais do estudo e explicada a natureza facultativa da participação, com possibilidade do participante poder desistir a qualquer momento, bem como do carácter anónimo confidencial do tratamento da informação

Foram considerados inválidos 23 questionários, por apresentarem incongruências que colocavam em questão a qualidade da informação recolhida, ou em virtude do não preenchimento das escalas consideradas como importantes para o estudo. Alguns dos questionários foram considerados válidos, embora apresentassem algumas das variáveis com respostas omissas. Essas omissões nunca atingiram os 20% recomendados como limite para se proceder à análise. Dada a dimensão da amostra, não é previsível que as respostas omissas possam enviesar os resultados finais (Pestana & Gageiro, 2014).

2.3.4. Instrumentos de recolha de dados

O método de recolha de dados a utilizar é determinado pela natureza do problema de investigação, pelas variáveis em estudo e pelas estratégias de análise estatística a realizar (Fortin et al., 2009). Foi com base nestes pressupostos que decidimos efetuar a recolha de informação, tendo como suporte um protocolo de investigação elaborado e selecionado com base na pesquisa bibliográfica sobre a temática em estudo. Na seleção dos diferentes instrumentos que integram o protocolo de investigação, tivemos em consideração alguns aspetos fundamentais, nomeadamente os objetivos delineados, metodologia de investigação e os instrumentos já existentes, que permitissem medir as variáveis e os constructos que pretendíamos estudar. Privilegiamos os instrumentos de avaliação com melhores características discriminantes, cuja utilização estivesse mais difundida na investigação e que

fossem adaptados para a população portuguesa, uma vez que a construção de instrumentos é uma prática morosa e de elevado rigor. Vários autores (Borsa, Damásio & Bandeira, 2012) sugerem a utilização de instrumentos já validados numa determinada língua, sendo recomendada a adaptação desse instrumento para a cultura e a língua onde se deseja estudar o fenómeno, uma vez que se trata de uma mesma medida, que permite avaliar o constructo a partir de uma mesma perspetiva teórica e metodológica. Entende-se que a utilização de instrumentos adaptados em diferentes amostras e contextos permite uma maior capacidade de generalização e investigação de diferenças interculturais (Arafat, Chowdhury, Qusar & Hafez, 2016; Hambleton, 2005).

Em conformidade com a natureza do fenómeno que se pretende estudar e os objetivos do estudo, o protocolo de investigação incluiu diversos instrumentos de colheita de dados e escalas *(Apêndice B)*. A relevância de cada uma das dimensões avaliadas e respetivas variáveis, bem como a descrição das questões usadas, encontram-se apresentadas nos diversos trabalhos que compõem o capítulo dos estudos empíricos, pelo que não vão ser alvo de uma descrição exaustiva neste capítulo. Apresenta-se de seguida uma sistematização dos instrumentos utilizados e a natureza da informação avaliada:

1. Questionário de caracterização dos estudantes, construído especificamente para o estudo. Com este instrumento pretendeu-se obter informação relativa a variáveis:

- *Sociodemográficas*: género, idade, nacionalidade, estado civil, zona habitacional onde cresceu, profissão dos pais, escolaridade dos pais, rendimento médio familiar e importância da religião;
- *Académicas:* ano de curso, curso que frequentava;
- *Fontes de informação sobre SSR*: médicos, enfermeiros, professores, amigos, pai, mãe, outro familiar, CAJ, internet, meios de comunicação social, livros e revistas científicas, outro;
- *Comportamentos sexuais*: já teve relações sexuais, idade da 1ª relação sexual; método contracetivo utilizado, orientação sexual, no último ano teve relações sexuais, relações sexuais aconteceram no contexto de uma relação amorosa, duração da relação amorosa;
- *Comportamentos e atitudes face à contraceção*: utiliza contraceção, qual o método contracetivo, se utiliza a pílula alguma vez se esqueceu de tomar, o que fez em caso de esquecimento, onde obteve informação sobre o método contracetivo que costuma usar, onde adquire o método contracetivo habitualmente, já utilizou contraceção de emergência, quantas vezes, qual o motivo para ter de usar contraceção de emergência, no momento de aquisição foi-lhe dada alguma informação e que tipo de informação;

- *Comportamentos de risco para a SSR*: utilizou sempre o preservativo nas relações sexuais no último ano, qual o motivo para não ter usado de forma consistente, já teve relações sexuais associadas ao consumo de álcool, drogas e quantas vezes, já teve relações sexuais com parceiros ocasionais e quantas vezes, já teve alguma IST, qual, onde recorreu para fazer tratamento, informou o parceiro sexual que estava com uma IST, se não, porquê;
- *Comportamento de vigilância de SSR:* já esteve alguma vez grávida, ou a companheira, quantas vezes, o que aconteceu com a gravidez, foi vacinada contra o HPV, faz citologia vaginal, faz autoexame da mama, faz autoexame do testículo, já fez o teste VIH/SIDA;
- *Acesso aos serviços de SSR:* já recorreu aos serviços de SSR, onde recorreu, qual o motivo, teve dificuldades no acesso, tem conhecimento dos serviços de SSR da universidade, na sua opinião o que poderia mudar para os serviços irem ao encontro das necessidades dos jovens;
- *Comportamentos psicossociais relativos à utilização do preservativo:* permitiu avaliar a perceção de benefícios e barreiras, perceção de sentimentos, influências interpessoais e situacionais e o compromisso com o plano de ação.

2. Instrumentos psicométricos:

- *Inventário de conhecimentos sobre SSR (ICSSR)*, foi construído especificamente para o estudo, constituído por 44 questões;
- *Escala de Atitudes face à SSR* (EASSR), foi construída especificamente para o estudo; inicialmente constituída por 38 questões, após validação ficou com 20 questões;
- *Escala de Autoeficácia para o Uso do Preservativo*: adaptada da *Condom Use Self-efficacy Scale* (CUSES) de Brafford e Beck (1991), versão reduzida de Brien, Thombs, Mahoney e Wallnau (1994), constituída por 15 questões. Após o processo de tradução, adaptação cultural e validação, a escala manteve o mesmo número de questões;
- *Escala de Busca de Sensações Sexuais*, adaptada da "*Sexual Sensation Seeking Scale*" (SSSS) de Kalichman e Rompa (1995), constituída por 11 questões, que no presente estudo, após o processo de tradução, adaptação cultural e validação, ficou composta por nove questões;
- *Escala de Satisfação com o Suporte Social (ESSS)*, versão portuguesa de Ribeiro (1999), constituída inicialmente por 15 questões que, no presente trabalho e depois do processo de validação, ficou constituída por 12 questões, as mesmas propostas pelo autor num estudo mais recente de validação da mesma escala (Marôco et al., 2014).

2.3.5. Adaptação, construção e validação de instrumentos

Neste capítulo são apresentados os critérios gerais utilizados na análise psicométrica das diferentes escalas, assim como os resultados da análise das qualidades psicométricas dos vários instrumentos que permitiram operacionalizar os constructos teóricos em estudo. Utilizamos instrumentos já validados para a população portuguesa (Escala de Satisfação com o Suporte Social) mas também procedemos à adaptação de instrumentos não validados para a população portuguesa (*Sexual Sensation Seeking Scale* [SSSS] *e a Condom Use Self-efficacy Scale* [CUSES]) e à construção de instrumentos, quando consideramos que os existentes não se adequavam à população em estudo (Inventário de Conhecimento sobre SSR e Escala de Atitudes de SSR).

Relativamente aos dois instrumentos que pretendíamos validar para a população portuguesa, foi realizado um contacto prévio com os autores através de correio eletrônico a solicitar autorização da sua tradução e validação de acordo com a realidade portuguesa, e estes autorizaram a sua adaptação *(Apêndice B)*. As duas escalas foram traduzidas, baseando-se em orientações de normas estabelecidas previamente (Beaton, Bombardier, Guillemin & Ferraz, 2000; WHO, 2011) que recomendam tradução, retrotradução, revisão por painel de especialistas e adaptação cultural.

Tradução inicial: foi realizada a tradução da escala por dois tradutores de inglês, conhecedores da finalidade do estudo, de inglês para português. *Revisão por um painel de especialistas:* foi realizada a revisão por um painel de especialistas na área da SSR para avaliar a equivalência conceptual. As versões (original e tradução) foram comparadas e as discrepâncias corrigidas dando origem a uma versão de consenso. *Retrotradução:* a versão de consenso das duas escalas foi novamente traduzida para o idioma original por dois tradutores ingleses, desconhecedores da versão original. As diferentes versões (original, tradução e retroversão) foram comparadas e a versão pré-final foi avaliada por especialistas na área temática (dois docentes especialistas em saúde materna e obstetrícia, um enfermeiro especialista em saúde materna e obstetrícia e um revisor de inglês) e um psicólogo relativamente à equivalência semântica, idiomática e conceptual. No que respeita ao pré-teste dos instrumentos, as versões finais foram testadas numa população de 173 estudantes, de duas instituições de ensino superior do norte dos pais, de modo a avaliar a sua aplicabilidade, selecionar e ajustar os itens do questionário e as instruções de resposta.

Passamos a descrever, em linhas gerais, o processo de avaliação das qualidades psicométricas dos vários instrumentos utilizados no estudo qualitativo, começando por apresentar os critérios gerais que estiveram subjacentes a toda a análise e que são

apresentados de forma sumária nos trabalhos que compõem o capítulo referente aos estudos empíricos.

Critérios de análise psicométrica dos instrumentos

No estudo das qualidades psicométricas dos instrumentos, foi realizada a análise da fidelidade, avaliada pela consistência interna (índice alfa de *Cronbach*) e validade, avaliada pela análise fatorial exploratória (AFE) e análise fatorial confirmatória (AFC).

A fidelidade é a capacidade que uma escala tem de obter os mesmos resultados se for aplicada às mesmas pessoas em condições semelhantes e expressa-se pelo coeficiente de correlação, pois mede a consistência entre dois resultados independentes (Ribeiro, 2010). De forma genérica, podemos considerar que a fiabilidade avalia a estabilidade temporal e a consistência interna ou homogeneidade dos itens, sendo que quanto mais elevadas forem as covariâncias (ou correlações entre os itens) maior é a homogeneidade dos itens e maior é a consistência com que medem a mesma dimensão ou constructo teórico Esta medida é particularmente importante em relação às escalas de itens múltiplos, o procedimento mais utilizado para a sua avaliação é o cálculo do alfa de *Cronbach* (α).Valores de alfa de *Cronbach* superiores a 0,90 são considerados muito bons, valores entre 0,80 e 0,90 são considerados bons, entre 0,70 e 0,80 são razoáveis e 0,70 e 0,60 são fracos (Marôco, 2011). Contudo, valores inferiores a 0,60 podem ser aceitáveis quando as escalas apresentam um número de itens reduzido (Hills & Hills, 2012). Na presente análise, foram considerados os seguintes critérios: i) a consistência interna do fator deve ser igual ou superior a 0,70; ii) a consistência interna do fator não deve aumentar se o item for eliminado; iii) as correlações item-total (correlações entre o item e o valor total do fator), devem apresentar valores superiores 0,40. Na análise das escalas, utilizou-se ainda o coeficiente de correlação de *Pearson* (r) e, embora sejam aceitáveis valores mínimos de correlação a partir de 0,20, quanto mais próximo o seu valor absoluto for de 1, mais forte a relação entre as duas variáveis (Hair, Black, Babin & Anderson, 2006).

Na análise fatorial exploratória (AFE) foi utilizado o método de extração de componentes principais com rotação ortogonal de *varimax.* Para confirmar a adequação da amostra, foram utilizados os critérios de *Kaiser-Meyer-Olkin* (KMO), e o teste de esfericidade de *Bartlett*. O KMO assume valores entre 0 e 1, sendo que quanto mais próximos de 1, melhor a adequação da amostra, seguindo os critérios de classificação definidos por Marôco (2011). O teste de esfericidade de Bartlett testa a hipótese das variáveis não serem correlacionadas na população e valores do teste de esfericidade de Bartlett com níveis de significância $p < 0,05$ indicam que as variáveis estão correlacionadas significativamente (Marôco, 2011). É

recomendado que o valor do teste KMO seja superior a 0,6 e o teste *Bartlett* esteja associado a valor de p significativo (Hill & Hill, 2012).

Para a determinação de fatores e retenção de itens, foram seguidas as recomendações de Worthington e Whittaker (2006), Marôco (2011) e de Brown (2016), utilizando os seguintes critérios: i) critério de Kaiser ou regra do valor próprio (*eigenvalue)*, igual ou superior a 1 (EV≥1); ii) peso fatorial dos itens iguais ou superiores a 0,4; iii) a percentagem da variância explicada pelos fatores retidos deve ser superior a 40%; iv) só devem ser retidos fatores com pelo menos três itens; v) como orientação foram também analisadas as comunalidades, ou seja, a quantidade de variância de cada variável que é explicada pelos fatores retidos. Quanto maior o valor da comunalidade, maior a variância dessa variável, extraída na análise fatorial e mais a variável está associada a determinado fator. Valores acima de 0,50 indicam que uma boa parte da variância dos resultados de cada item é explicada pela solução fatorial. No entanto, a hipótese de eliminação dos itens só deve ser considerada com valores abaixo de 0,40 (Worthington & Whittaker, 2006). O número de sujeitos necessários para realizar a AFE foi também tido em conta para no presente estudo. Uma razão de 10:1 (número de sujeitos por cada item do questionário) é o número recomendado e que gera algum consenso na literatura (Hill & Hill, 2012; Worthington & Whittaker, 2006).

Na análise fatorial confirmatória (AFC), os modelos foram estimados pelo método da máxima verosimilhança e testado pela razão entre o qui-quadrado e os graus de liberdade (χ^2/gl), considerando os seguintes índices de ajustamento e critérios de corte: *Comparative Fit Index* (CFI> 0,90), *Goodness-of-fit Index* (GFI> 0,90), *Standardized Root Mean Square Residual* (SRMSR <0,08), Root Mean Square Residual (tanto melhor quanto mais próximo de zero) e *Root Mean Square Error of Approximation* (RMSEA <0,06). A validade convergente foi avaliada pela variância extraída média (VEM), a discriminante comparando a VEM com o quadrado da correlação de *Pearson* e a fiabilidade do constructo pela fiabilidade compósita (FC). Foram considerados como valores de referência a VEM> 0,5, FC ≥0,7 e existência de validade discriminante quando o quadrado da correlação entre os fatores é menor do que a VEM para cada fator (Marôco, 2011).

Avaliação psicométrica dos instrumentos

De seguida apresenta-se a análise das qualidades psicométricas dos instrumentos utilizados, referindo o seu processo de construção e os resultados da análise da fiabilidade e validade na população em estudo, nomeadamente do Inventário Conhecimento em SSR (ICSSR) e da Escala de Satisfação com o Suporte Social (ESSS). Os resultados referentes à construção e/ou adaptação cultural e validação restantes escalas (Escala de Atitudes de SSR**,** Escala de Autoeficácia para o Uso do Preservativo (CUSES), Escala de Busca de

Sensações Sexuais (SSSS) encontram-se no capitulo III - Estudos empíricos - uma vez que fazem parte dos artigos já publicados, submetidos ou a submeter para publicação.

Inventário de Conhecimento em Saúde Sexual e Reprodutiva

A construção do ICSSR teve por base a revisão da literatura e a opinião de peritos. Fundamenta-se nas recomendações de documentos orientadores a nível nacional e internacional, nomeadamente nas recomendações da DGS e APF, OMS, CDC, inventários e testes de avaliação de conhecimento que constam do *Handbook of Sexuality-Related Measures* (Davis, Yarber, Bauseserman, Schreer & Davis, 1998), nomeadamente o *Contracetive Knowledge Inventory*, o *HIV Prevention Knowdelege Test for Teenagers*, *The sex knowledge and Atitude Test*, e no documento orientador para a elaboração de questões no âmbito da sexualidade, o *Family Planning and Reproductive Health Indicators Database.*

Para construção do inventário procedemos à seleção de um conjunto de itens a incluir em cada uma das quatro áreas temáticas consideradas a partir da revisão da literatura, nomeadamente: informação relacionada com a sexualidade e reprodução, vigilância e cuidados de SSR, contraceção e IST e comportamentos sexuais de risco (com particular ênfase nas infeções mais prevalentes em jovens adultos).

Após a construção e antes da aplicação do pré-teste, foi solicitado a um grupo de peritos (quatro enfermeiros especialistas em saúde materna e obstétrica, dois da prática clínica e dois da docência), que avaliassem da importância teórica, pertinência e clareza dos diferentes itens da versão preliminar do inventário, bem como da sua adequação aos objetivos em estudo. Após análise, foram reformuladas algumas questões e retiradas duas que, de alguma forma, repetiam as anteriores e foi simplificada a linguagem de algumas questões. A versão de consenso do ICSSR ficou composta por 44 questões que integravam quatro áreas temáticas: informação relacionada com sexualidade e reprodução (1, 2, 4, 7, 8, 10, 15 e 41), vigilância e cuidados de SSR (35, 36, 40, e 43), métodos contracetivos (3, 5, 9, 12, 14,17, 20, 21, 24, 27, 28, 30, 31, 32, 37 e 38), IST e comportamento sexuais de risco (6, 11, 13, 16, 18, 19, 22, 23, 25, 26, 29, 33, 34, 39, 42, e 44). As afirmações permitiam três possibilidades de resposta (“verdadeiro”, “falso” e “não sei”), a última opção foi considerada para evitar que os participantes respondessem ao acaso. As afirmações foram pontuadas com um ponto no caso das respostas corretas e zero pontos para as respostas incorretas ou respondidas com “não sei”, uma vez que consideramos que em ambas as situações existe falta de conhecimento. A pontuação total do ICSSR é obtida pela soma de todas as afirmações consideradas corretas, variando entre 0 e 44 pontos, sendo que quanto maior é a pontuação, maior é o nível de conhecimento sobre SSR. Procurando evitar tendências de

resposta, as afirmações foram construídas de forma a representarem conceitos positivos e negativos relacionados com a SSR.

Apesar de o ICSSR ter sido elaborado como um instrumento de três pontos, posteriormente foi transformado num instrumento dicotómico, pelo que consideramos realizar o estudo da homogeneidade dos itens pela determinação do alfa de *Cronbach,* visto este ser considerado a “medida de consistência interna por excelência” (Marôco & Garcia-Marques, 2006, p.72), no entanto, considerando os pressupostos defendidos por alguns autores relativamente aos instrumentos com enunciado dicotómico, para confirmação da consistência interna do inventário, foi avaliado o coeficiente *Kuder-Richardson* (KR20), considerado como um análogo ao coeficiente alfa de *Cronbach,* utilizado como medidas da consistência interna no estudo da fidelidade de escalas dicotómicas. O valor obtido para o ICSSR foi de KR20=0,780 e KR21=0,724, considerados como bons (Marôco & Garcia-Marques, 2006), pelo que a consistência interna do instrumento revelou ser adequada.

Escala de Satisfação com o Suporte Social

A ESSS pretende avaliar o suporte social percebido e o apoio que os respondentes recebem dos amigos, família e comunidade. É constituída por 15 itens de autopreenchimento, numa escala tipo *Likert* com cinco posições (1- Concordo totalmente e 5 – Discordo totalmente), são exceção os itens invertidos (4, 5, 9, 10, 11, 12, 13, 14 e 15). A pontuação total da escala varia entre 15 e 75 pontos e à nota mais alta corresponde uma perceção de maior suporte social. No presente estudo, optamos por inverter a codificação numérica para que a um maior valor numérico correspondesse uma maior concordância (1- Discordo totalmente e 5- Concordo totalmente), à semelhança das restantes escalas utilizadas na presente investigação. Assim, na versão utilizada no presente estudo, a pontuação dos itens 1, 2, 3, 6, 7 e 8 foi revertida.

No processo de validação da ESSS pelo autor (Ribeiro, 1999), numa amostra de estudantes universitários, a escala revelou uma estrutura fatorial composta por quatro fatores que explicam 63,1% da variância total. O primeiro fator foi denominado “satisfação com os amigos” e mede a satisfação com as amizades/amigos e inclui cinco itens (3, 12, 13, 14 e 15). O segundo fator, denominado “intimidade”, mede a perceção da existência de suporte social íntimo e inclui quatro itens (1, 4, 5 e 6). O fator três, denominado “satisfação com a família”, mede a satisfação com o suporte familiar existente, é composto por três itens (9, 10 e 11). Finalmente, o quarto fator, denominado “atividades sociais”, mede a satisfação com as atividades sociais que realiza e é composto por três itens (2, 7 e 8). O valor de consistência interna para a nota global da escala foi adequado (α=0,85). De acordo com o autor (Ribeiro, 2007), esta escala tem sido utilizada em diversos estudos e populações,

onde se tem observado a necessidade de redução de itens, ou os itens apresentam uma distribuição diferente por dimensão, nomeadamente fundindo dimensões (Ferreira, Ribeiro & Guerreiro, 2004; Santos, Pais Ribeiro & Lopes, 2003). Estes ajustamentos podem estar associados à idade, onde a satisfação com a família e amigos pode diferir consideravelmente.

A avaliação das qualidades psicométricas da ESSS no presente estudo foi avaliada pela confiabilidade e validade com recurso à AFE, numa amostra de1933 estudantes do ensino superior (Tabela 3). A consistência interna para a escala global foi calculada pelo coeficiente de alfa de *Cronbach*, tendo-se obtido um valor de 0,868, revelando que todos os itens contribuem para consistência da escala.

Tabela 3.
Valores de consistência interna de cada um dos fatores, com e sem exclusão de itens, bem como a correlação *item total* para cada um dos itens e para o total da Escala de Satisfação com o Suporte Social (ESSS)

Nº Item	Questões	Média	DP	Item total correlação corrigido	Correlação múltipla ao quadrado	Alfa de *Cronbach* se o item for excluído
1	Por vezes sinto-me só no mundo e sem apoio	3,79	1,16	0,516	0,325	0,850
2	Não saio com amigos tantas vezes quantas eu gostaria	3,24	1,25	0,473	0,406	0,852
3	Os amigos não me procuram tantas vezes quantas eu gostaria	3,70	1,06	0,616	0,489	0,844
4	Quando preciso de desabafar com alguém encontro facilmente amigos com quem o fazer.	3,96	1,06	0,497	0,480	0,850
5	Mesmo nas situações mais embaraçosas, se precisar de apoio de emergência tenho várias pessoas a quem posso recorrer	4,05	,95	0,498	0,480	0,851
6	Às vezes sinto falta de alguém verdadeiramente íntimo que me compreenda e com quem possa desabafar sobre coisas íntimas.	3,49	1,29	0,413	0,261	0,857
7	Sinto falta de atividades sociais que me satisfaçam.	3,41	1,15	0,599	0,479	0,845
8	Gostava de participar mais em atividades de organizações (por ex. clubes desportivos, escuteiros, partidos políticos, etc.)	2,99	1,21	0,250	0,191	0,865
9	Estou satisfeito com a forma como me relaciono com a minha família	4,10	0,95	0,507	0,455	0,850
10	Estou satisfeito com a quantidade de tempo que passo com a minha família	3,44	1,13	0,390	0,343	0,856
11	Estou satisfeito com o que faço em conjunto com a minha família	3,79	1,02	0,512	0,531	0,850
12	Estou satisfeito com a quantidade de amigos que tenho	4,11	0,85	0,645	0,556	0,845
13	Estou satisfeito com a quantidade de tempo que passo com os meus amigos	3,74	1,01	0,588	0,545	0,846
14	Estou satisfeito com as atividades e coisas que faço com o meu grupo de amigos	3,95	0,88	0,636	0,577	0,845
15	Estou satisfeito com o tipo de amigos que tenho	4,35	0,75	0,505	0,443	0,851
			Primeira metade		0,780	
	Coeficiente Spilt-half		Segunda metade		0,831	
	Coeficiente alfa Cronbach total				0,868	

Os coeficientes de correlação item total corrigido revelam que o item 8 é o que apresenta menor correlação (r=0,250) e o item 12 a correlação mais elevada (r=0,645), havendo homogeneidade dos itens e sugerindo bons índices de validade interna (poder discriminativo). Dado o reduzido número de itens da subescala a que pertence o item 8 “Gostava de participar mais em atividades de organizações” e visto que a sua remoção não implica um aumento do valor de alfa de *Cronbach*, optou-se por manter a estrutura fatorial proposta pelo autor.

A estrutura fatorial da escala ESSS foi avaliada pela AFE, utilizando o método de extração das componentes principais, seguido da rotação de *varimax*, considerando os critérios gerais de análise mencionados pelo autor da escala original. A medida de adequação da amostra (teste KMO=0,866) indica que a análise de componentes principais pode ser realizada e o teste de esfericidade significativo indica-nos que as variáveis se correlacionam entre si (Marôco, 2011). Os resultados das comunalidades também são aceitáveis (Hair et al., 2006), com valores a variar entre 0,45 para o item 8 e 0,79 para o item 11. A solução inicial apresenta uma estrutura ligeiramente discordante da estrutura original (Ribeiro, 1999), uma vez que, embora o agrupamento dos itens indique uma estrutura de quatro fatores, que justificam no seu conjunto 63,75% da variância total dos resultados, da análise do agrupamento dos itens nas dimensões propostas, observou-se que os itens 1, 2, 3, 6 e 15 saturavam com valores próximos ou mesmo superiores em dimensões diferentes das sugeridas pelo autor. Assim, optamos por proceder a nova análise seguindo os pressupostos descritos no artigo recente de adaptação transcultural da ESSS com estudantes do ensino superior portugueses e brasileiros (Marôco et al., 2014), onde o autor original propõe uma versão modificada da ESSS com 12 itens, mantendo as quatro dimensões. De acordo com os autores (Marôco et al., 2014), a versão modificada revelou adequada confiabilidade, validade fatorial, validade concorrente, divergente e discriminante.

Procedemos a nova AFE, retirando os itens 1, 3 e 13, sugeridos na versão modificada (Marôco et al., 2014). A análise confirmou a estrutura conceptual proposta pelo autor composta por quatro dimensões - satisfação com os amigos (SA) – itens 12, 14 e 15; intimidade (IN) – itens 4, 5 e 6, satisfação com a família (SF) – itens 9, 10 e 11; e atividades sociais (AS) – itens 2, 7 e 8 – que, no seu conjunto, explicam 67,12% da variância total dos resultados (SF - 36,08%, IN - 12,36%, SA - 11,20% e AS - 7,47%) (Tabela 4). Os fatores que melhor explicam o resultado da escala são o da “satisfação com a família” e “intimidade”, que explicam, respetivamente, 36,08% e 12,36% da variância dos resultados.

A análise da consistência interna dos fatores revelou valores adequados para as dimensões “satisfação com a família” e “satisfação com os amigos” (α_{SF}=0,799, α_{SF}=0,756) e valores no limite da confiabilidade adequada para as dimensões intimidade e atividades sociais

(α_{IN}=0,635, α_{AS}=0,624), contudo, estes valores de alfa de Cronbach são aceitáveis, considerando que estas dimensões são constituídas apenas por três itens (Hair et al., 2006).

Quadro 4.
Estrutura fatorial da escala de satisfação com o suporte social (ESSS), e valores de consistência interna por fator e para o total da escala

Nº Item	Itens	Carga Fatorial				H^2
		Satisfação Família	Intimidade	Satisfação Amigos	Atividade Social	
11	Estou satisfeito com o que faço em conjunto com a minha família	0,861				0,796
10	Estou satisfeito com a quantidade de tempo que passo com a minha família	0,776				0,634
9	Estou satisfeito com a forma como me relaciono com a minha família	0,727				0,646
4	Quando preciso de desabafar com alguém encontro facilmente amigos com quem o fazer.		0,807			0,723
5	Mesmo nas situações mais embaraçosas, se precisar de apoio de emergência tenho várias pessoas a quem posso recorrer		0,804			0,716
6	Às vezes sinto falta de alguém verdadeiramente íntimo que me compreenda e com quem possa desabafar sobre coisas íntimas.		0,462			,559
14	Estou satisfeito com as atividades e coisas que faço com o meu grupo de amigos			0,754		0,705
12	Estou satisfeito com a quantidade de amigos que tenho			0,662		0,692
15	Estou satisfeito com o tipo de amigos que tenho.			0,627		0,640
7	Sinto falta de atividades sociais que me satisfaçam.				0,768	0,728
8	Gostava de participar mais em atividades de organizações (ex. clubes desportivos, escuteiros, partidos políticos, etc.).				0,737	,555
2	Não saio com amigos tantas vezes quantas eu gostaria				0,519	0,660
	Medida de adequação da amostra - KMO	0,826				
	Teste de esfericidade de Bartlett	χ^2 =7788,908; p<0,000				
	Valores próprios iniciais	4,330	1,483	1,344	0,996	
	% de variância explicada	36,08%	12,36%	11,20%	7,47%	
	Alfa de Cronbach por dimensão	0,799	0,635	0,756	0,624	
	Alfa Cronbach total	0,814				

H^2 - Comunalidades

Face aos resultados, consideramos que a ESSS, na sua versão modificada aplicada nesta amostra de estudantes do ensino superior, tem propriedades psicométricas adequadas, podendo ser usada na avaliação da satisfação com o suporte social no presente estudo.

2.3.6. Análise estatística

Os dados recolhidos, após codificação e informatização, foram analisados no programa SPSS (versão 22.0). Para efeitos de análise estatística, algumas das variáveis foram agrupadas e recodificadas de forma a permitir uma análise mais adequada.

Na análise descritiva utilizaram-se distribuição de frequências, medidas de tendência central e de dispersão, de acordo com o tipo de variável e a escala de medida. Os pressupostos de normalidade foram avaliados através dos valores de assimetria e achatamento.

Na análise inferencial, as variáveis contínuas foram comparadas pelos testes *t-Student* para amostras independentes ou análise de variância (Anova). Nas situações em que se observaram diferenças, utilizou-se o teste de comparações múltiplas de *Tukey*. As variáveis nominais foram avaliadas pelo teste de *Qui-quadrado* com análise das diferenças de residuais ajustados para localização das células com valores significativos. No estudo da associação entre as variáveis, foi utilizada a correlação de *Pearson*, estabelecendo os seguintes valores de corte: $0,2 \leq r \leq 0,39$ - associação baixa, $0,4 \leq r \leq 0,69$ - associação moderada, $0,7 \leq r \leq 0,89$; associação alta e $0,9 \leq r \leq 1,0$ – associação muito alta (Marôco, 2011).

Na análise multivariada foi realizada regressão logística (método *forword likelihood-ratio*) e a regressão múltipla. Foi ainda utilizada a *Path analysis* (através do programa AMOS, versão 23) para testar o modelo teórico explicativo do comportamento de utilização do preservativo de acordo com as variáveis do HPM. Esta técnica estatística, considerada uma extensão da regressão múltipla, permitiu estudar as relações e interações entre as variáveis pertencentes ao modelo teórico hipotético e incluir todas as variáveis dentro de um mesmo modelo de regressão (Marôco, 2011). A colinearidade entre as variáveis independentes inseridas no modelo foi avaliada pelos valores de inflação da variância (VIF), aceitando-se a ausência de colinearidade quando os valores foram inferiores a 5. Nos índices de ajustamento global do modelo, foram cumpridos os pressupostos de amostragem que recomendam pelo menos 10 participantes por variável (Kline, 2005), e considerados os indicadores de ajustamento já mencionados neste capítulo, relativos à AFC.

Todos os testes utilizados com valor de prova inferior a 0,05 foram considerados como indicando significância estatística.

2.4. Estudo qualitativo

Neste ponto, apresenta-se a abordagem qualitativa na perspetiva de aprofundar a compreensão do fenómeno em estudo e dando conta do percurso metodológico efetuado apresentando os procedimentos de planeamento, a recolha e análise dos dados obtidos nos grupos de discussão focalizada. A descrição do estudo e dos objetivos específicos será também apresentada num dos artigos que compõem o capítulo dos estudos empíricos sobre SSR.

2.4.1. Percurso metodológico - Grupos de discussão focalizada

A investigação qualitativa tem como objetivo traduzir e expressar o sentido dos fenómenos do mundo social. E segue uma intencionalidade que procura compreender os fenómenos intensos e complexos de natureza mais subjetiva e problemática. As metodologias qualitativas enfatizam a descrição, as perceções dos sujeitos, os processos e as intenções, contribuindo para um melhor entendimento da mudança e interação humana. Neste âmbito, a recolha de informação foi feita através da técnica de grupos de discussão focalizada que se baseiam na dinâmica de interação que se gera entre as pessoas num debate focalizado num tema específico. Este procedimento metodológico tem vindo a ser cada vez mais utilizado na investigação em ciências sociais, ciências da saúde e de enfermagem. A utilização desta técnica reveste-se de particular importância na discussão e análise de temas complexos, multifacetados (Jayasekara, 2012) e de natureza íntima (Oliveira, 2011), incluindo a investigação de questões relacionadas com a SSR (Lobdell, Gilboa, Mendola & Hesse, 2005; Oliveira 2011; Peterson-Sweeney, 2005). Alguns estudos têm demonstrado a utilidade deste procedimento na obtenção de informação, não só sobre comportamentos individuais, mas também sobre fenómenos coletivos, dado que a análise é focalizada nos dados obtidos no grupo, e não em cada um dos participantes em particular (Flick, 2005). Das discussões focalizadas emergem aspetos que não seriam acessíveis sem a interação grupal e que o processo de compartilhar e comparar oferece oportunidade, ao investigador, de compreender o modo como os participantes entendem as suas similaridades e diferenças (Morgan, 2000).

A utilização de grupos focais no presente estudo permitiu recolher informação para complementar e clarificar alguns dos resultados obtidos na primeira fase do estudo, dado que a análise qualitativa nem sempre permite compreender as perceções e cognições que lhe estão associadas. Este tipo de abordagem permite perceber e explorar os significados atribuídos pelos participantes a alguns dos temas em estudo, uma vez que os comportamentos sexuais devem ser entendidos numa dinâmica, onde a interação humana assume um papel fundamental (Hollander, 2004; Oliveira, 2011). Ainda de acordo com Dias e Rocha (2009), esta técnica de recolha de dados tem subjacente a necessidade de se compreender as múltiplas variáveis que contribuem para os complexos, e muitas vezes contraditórios, significados de crenças, atitudes e comportamentos de risco e de proteção, relacionados com a SSR, construídos num contexto de elementos sociais, económicos e culturais que caracterizam a realidade específica em que os indivíduos estão inseridos.

Segundo Morgan (2000), a metodologia dos grupos focais pode ser definida como uma discussão cuidadosamente preparada, que tem como objetivo obter dados sobre as perceções dos participantes acerca de determinada área de interesse, denominada de

“foco”. O objetivo é perceber as opiniões, crenças, atitudes, valores, representações sociais, conceitos vigentes no grupo e sentimentos subjacentes a um determinado comportamento (Gill, Stewart, Treasure & Chadwick, 2008; Hollander, 2004). Desta forma, os grupos focais permitem, num espaço de tempo relativamente curto, otimizado pela reunião de um número significativo de participantes e pelo confronto de ideias que se estabelece, ouvir a perspetiva dos principais interessados e envolvê-los no processo de tomada de decisão (Jayasekara, 2012).

Para salvaguardar e maximizar a qualidade dos dados recolhidos, tivemos em consideração alguns dos aspetos considerados como relevantes, seguindo as linhas orientadoras propostas por autores de referência no planeamento e condução dos grupos focais (Gill et al., 2008; Hyde, Howlett, Brady & Drennan, 2005; Krueger & Casey, 2009; Morgan, 2000):

i) o número de participantes dos grupos focais é variável, sendo consensual que deve variar geralmente entre seis e 12 participantes, constituído por pessoas com características homogéneas (cultura, idade, gênero, estatuto social, de entre outros aspetos), que permitam que a discussão seja centrada numa temática (Morgan, 2000). Argumenta-se que grupos menores podem ser mais eficazes para tópicos complexos (Krueger & Casey, 2009). Na discussão de temáticas consideradas sensíveis, como aquela que é abordada neste estudo, a constituição dos grupos deve ser criteriosa, de forma a facilitar a comunicação, mantendo a heterogeneidade de atitudes, crenças e comportamentos.

ii) o papel do moderador é considerado como um elemento fundamental desta metodologia, pois é o responsável por promover e facilitar a discussão. A atitude do moderador pode influenciar a qualidade da informação obtida, pelo que deve ser capaz de cobrir a máxima variedade de tópicos sobre o assunto e promover uma discussão produtiva (Morgan, 2000). Dada a natureza e os objetivos em estudo, é comummente aceite a existência de dois moderadores para fazer face à minúcia das intervenções. As questões deverão ser colocadas de forma aberta no sentido de evitar limitar as respostas dos participantes (Krueger & Casey, 2009). Neste sentido, tivemos dois moderadores nos grupos focais do presente estudo que tentaram reduzir a sua influência durante a discussão, criando condições de empatia e confiança, para que cada participante pudesse manifestar e partilhar o seu ponto de vista sobre a temática em estudo. A discussão decorreu sem uma estrutura rígida mas seguindo um conjunto de questões de orientação, procurando respeitar-se a dinâmica e os interesses do grupo, uma vez que não havia a necessidade de se estabelecer um ponto de consensualidade entre os diversos participantes. O interesse deste estudo não foi o de procurar generalizações,

mas explorar os pontos de vista dos participantes acerca dos seus conhecimentos, atitudes e práticas, na área da SSR.

iii) a escolha de um ambiente privado, acolhedor, confortável e convidativo à participação ativa é fundamental para o sucesso da técnica. Os grupos focais decorreram num local com condições acústicas adequadas e com a possibilidade de os participantes se sentarem em círculo, de forma a favorecer a dinâmica de grupo.

2.4.2. Planeamento e procedimentos de recolha de dados

No processo de planeamento foram tidos em consideração os objetivos do estudo, os critérios de seleção dos participantes, o instrumento de recolha de dados, o local de realização das sessões, o processo de recrutamento dos estudantes e o incentivo à participação.

Os convites com os objetivos e informação sobre as condições de participação foram enviados por correio electrónico para os endereços cedidos pelos estudantes na primeira fase da investigação e que mostraram disponibilidade para participar na segunda fase. Responderam à nossa solicitação 23 estudantes, a quem foi enviado toda a informação sobre os grupos focais (procedimentos e condições de recolha de dados) *(Apêndice C).* Os estudantes foram informados, oralmente e por escrito, sobre os objetivos do estudo, o carácter confidencial da informação recolhida, os procedimentos e a modalidade dos grupos focais, assim como o carácter voluntário da participação. Dos estudantes que responderam à solicitação para participar nos grupos focais, procedeu-se à sua alocação num dos grupos, de forma a equilibrar o número de participantes em cada grupo focal, assegurando-se a representatividade do género e da área científica de formação. Para garantir a comparência no dia fixado, foram realizados contactos telefónicos com os estudantes, sendo o último contacto realizado no dia anterior para confirmar hora e local de realização dos grupos focais. Dois dos estudantes não compareceram no dia das entrevistas.

Com os 21 estudantes que compareceram no dia das entrevistas, foram constituídos dois grupos focais, com 10 e 11 elementos em cada grupo, que decorreram em abril de 2015, numa sala de reuniões da universidade. Procuramos que o local fosse confortável, privado e neutro para os estudantes e que permitisse que estivessem sentados em círculo, de forma a favorecer a dinâmica de grupo. Antes da entrevista foi realizado um pequeno lanche de convívio, com o objetivo de permitir alguma interação prévia entre os participantes. Previamente, foi preparado todo o material de registo de som e imagem. A dinâmica decorreu na presença e na interação desenvolvida entre todos – os dois moderadores e os participantes – e cada sessão demorou em média cerca de duas horas. Alguns jovens

conheciam-se previamente e outros não, os que se conheciam frequentavam o mesmo curso, embora em anos diferentes.

Os grupos focais foram iniciados com o acolhimento dos participantes, foi realizado um agradecimento pelo facto de terem aceitado participar no estudo, foi feita a apresentação da equipa de investigação, relembrados os objetivos do estudo e enfatizadas algumas regras de participação: a importância da participação de todos, a possibilidade de todos intervirem de forma ordenada sem sobreposição para facilitar o processo de transcrição das entrevistas e não existir respostas certas ou erradas visto que todas as opiniões são válidas.

Foram ainda realizados alguns esclarecimentos adicionais sobre algumas considerações éticas inerentes à metodologia utilizada, nomeadamente:

i) a necessidade das sessões serem gravadas em áudio e vídeo;

ii) reiterada a garantia de anonimato e confidencialidade de toda a informação individual recolhida, com destruição do material após o fim do estudo;

iii) esclarecimento dos participantes de que a sua participação era absolutamente voluntária e que, não se sentindo confortáveis na discussão, poderiam abandonar a sala sem prestarem qualquer explicação;

iv) esclarecimento dos papéis dos moderadores, que são fundamentalmente de escuta ativa e de menor intervenção na discussão;

v) possibilidade das moderadoras intervirem para recentrar o assunto em discussão ou para permitir que alguns dos participantes menos interventivos pudessem expressar a sua opinião;

vi) importância de todos participarem na discussão, sem conversas paralelas e que todos teriam a oportunidade de expressar a sua opinião sobre a temática em discussão.

No início do grupo focal foi realizada uma atividade de apresentação, com os objetivos de “quebra-gelo” e facilitar a apresentação interpessoal, que incluía a seleção de algumas palavras para alocar ao conceito de SSR, e explicação sumária por parte de cada um dos participantes da sua escolha. Foi realizado o preenchimento do consentimento informado.

Todos os participantes tiveram oportunidade de manifestar a sua opinião sobre os vários temas abordados, de forma mais ou menos aprofundada, de acordo com as características pessoais, mais inibidos ou mais expansivos, mas foram sempre convidados a participar na discussão. Nesta investigação, o objetivo principal do grupo focal foi gerar diferentes pontos de vista e perspetivas em torno das questões da sexualidade e da saúde reprodutiva no que diz respeito a comportamentos sexuais de risco e de proteção. Neste sentido, o debate foi-se desenvolvendo seguindo um conjunto de questões orientadoras sobre a temática

(Apêndice D), mas de forma pouco rígida. No final das sessões foi aplicado o questionário de caracterização dos participantes.

Cada sessão foi registada em vídeo e áudio, após o consentimento prévio dos participantes, de forma a maximizar a recolha de dados e permitir a transcrição integral das discussões realizadas, para posterior análise. Finalizados os grupos de discussão focalizada, procedeu-se ao registo das observações sobre a dinâmica e interação dos participantes nos dois grupos e transcrição integral da informação recolhida.

2.4.3. Instrumento de recolha de dados

Para a recolha de dados elaborou-se previamente um conjunto de questões norteadoras do tema em estudo, e que favorecessem a condução dos grupos focais, o que acabou por se constituir num dispositivo essencial de orientação do foco da discussão (*Apêndice D – questões orientadoras)*. Utilizou-se também um questionário individual *(Apêndice E)*, para caracterização dos participantes aos níveis: sociodemográfico (género, idade, proveniência), académico (tipo de curso e ano de curso) e dos comportamentos sexuais e reprodutivos (têm relações sexuais, utilização de contraceção, utilização do preservativo na última relação sexual, realização de vigilância de saúde). As questões orientadoras utilizadas nos grupos focais tiveram por base a revisão da literatura e os dados da primeira fase do estudo, incluindo as áreas temáticas onde se pretendia aprofundar a compreensão dos resultados da análise quantitativa, garantindo ainda que a dinâmica de cada um dos grupos se desenvolvia numa base comum. Em síntese, a sua utilização permitiu sistematizar as áreas que pretendíamos focar, dar um quadro referencial à discussão e organizar a recolha da informação, garantindo que todas as questões relevantes da temática seriam abordadas, sem, no entanto, conferir uma estrutura rígida à discussão.

2.4.4. Participantes no estudo

A amostra representada no estudo foi uma amostra constituída por 21 estudantes do ensino superior, 16 do género feminino e cinco do masculino, com idades compreendidas entre os 19 e os 27 anos, e uma média de idades de 21 anos (Tabela 5).

Tabela 5.
Distribuição das características sociodemográficas dos participantes

Variáveis	Grupo Focal 1	Grupo Focal 2	Total
	**N=11**	**N=10**	**N=21**
Género			
Feminino	8	8	16
Masculino	3	2	5
Classe etária			
Menor ou igual a 19	1	1	2
20- 24anos	9	9	18
Igual ou superior 25		1	1
Proveniência			
Vila Real	7	8	15
Outra (Norte de Portugal)	4	2	6
Ano de curso			
2º Ano	4	2	6
3ª Ano	7	7	14
4ª Ano		1	1
Área científica do curso			
Psicologia	3	4	7
Educação básica	1	2	3
Ciências da Comunicação	-	1	1
Ciências do Desporto	2	-	2
Engenharia	2	1	3
Enfermagem	2	2	4
Enologia	1	-	1

Quinze participantes eram do distrito onde se insere a universidade onde decorreu o estudo e os restantes de outros distritos da região norte do país. Os participantes frequentavam diversas áreas científicas dentro dos cursos disponibilizados pela universidade e a maioria frequentava o 2.º e 3º ano do curso. Relativamente aos comportamentos de SSR (Tabela 6), a maioria dos estudantes (16) referiu ter relações sexuais e todos afirmaram usar contraceção.

Dos métodos de contraceção utilizados, 8 estudantes usavam o preservativo, 4 a pílula e 9 a dupla contraceção. Cerca de metade dos estudantes (12) refere ter utilizado o preservativo na última relação sexual. Quanto à vigilância de SSR, 14 realizam esta vigilância, e dos 7 que não realizam, 4 são do género masculino, como se pode verificar na Tabela 6. As características individuais estão de acordo com os estudos na área e o grupo de jovens acaba por espelhar os comportamentos na sua faixa etária, da população estudantil.

Tabela 6.
Comportamentos sexuais e reprodutivos dos participantes

Variáveis	Grupo Focal 1	Grupo Focal 2	Total
	**N=11**	**N=10**	**N=21**
Atualmente relações sexuais			
Sim	7	9	**16**
Não	4	1	**5**
Contraceção			
Sim	11	10	**21**
Não	-	-	**-**
Método contracetivo utilizado			
Preservativo	5	3	**8**
Pílula	2	3	**5**
Preservativo e Pílula	4	4	**8**
Utilizou preservativo na última relação sexual			
Sim	5	7	**12**
Não	6	3	**9**
Realiza vigilância de SSR			
Sim	7	7	**14**
Não	4	3	**7**

2.4.5. Análise interpretativa de dados

O processo de análise dos dados pressupôs uma abordagem qualitativa (Bardin, 2004; Krueger & Casey, 2009), que procura articular com rigor a sistematização e a flexibilidade exigida pela natureza dos dados recolhidos. O *corpo* de análise que resultou da transcrição integral da informação foi, então, sujeito a uma análise exploratória do material, de acordo com os objetivos do trabalho. Mais especificamente, após as transcrições obtivemos um conjunto de características, relatos de comportamentos ilustrativos do conhecimento que os jovens interiorizaram sobre a problemática, que submetemos a uma leitura "flutuante" e a uma análise exploratória indutiva que nos permitiu sinalizar um conjunto de unidades de registo, que nos possibilitou interpretar os dados à luz do quadro de referência teórico proposto pelo HPM de Pender (Pender et al., 2011). Com este modelo de análise, procuramos avaliar os comportamentos relacionados com a promoção da SSR, pelo estudo das interrelações de vários fatores relacionados com o risco e a proteção que os jovens adultos estão expostos no âmbito da atividade sexual, designadamente:

i) *características individuais e experiências*, que compreendem os fatores individuais (biológicos, psicológicos e socioculturais) e o comportamento sexual e reprodutivo anterior;

ii) *conhecimento e sentimentos sobre o comportamento* que se pretende alcançar (comportamento sexual seguro, nomeadamente uso consistente do preservativo o acesso aos serviços de SSR), avaliado pelas variáveis: perceção de benefícios e barreiras para a ação, perceção de autoeficácia, sentimentos em relação ao comportamento e influências interpessoais (família, pares e profissionais de saúde) e situacionais (uso de álcool e

drogas associado às relações sexuais, acessibilidade aos serviços de SSR) e o compromisso com o plano de ação, que são as ações que possibilitam o indivíduo manter-se no comportamento de promoção da saúde desejado, e no qual a intervenção de enfermagem é fundamental;

iii) *comportamento promotor de saúde*, que no presente trabalho foi avaliado pela perceção dos estudantes sobre as estratégias mais importantes a integrar num plano de ação para promoção da SSR em estudantes do ensino superior.

No sentido de agilizar o processo de análise, foi elaborada uma tabela, integrando cada uma das componentes de análise (Tabela 7), em que as transcrições relevantes foram numeradas e identificadas (cada estudante é referenciado pela letra E, seguida numeração do grupo focal que integrou e respetivo número de identificação individual, e.g. E1.1). À medida que os discursos foram transcritos, houve a preocupação de agrupar a informação segundo ideias/significados idênticos, que foram sendo ajustadas à medida que os dados foram organizados nas diferentes componentes de análise. Em cada componente foram incluídos excertos dos discursos, considerados como ilustrativos dos significados atribuídos pelos estudantes. As discussões foram, ainda, comparadas dentro de cada grupo e no global.

Tabela 7.
Componentes do Modelo de Promoção da Saúde de Pender, adaptado de *Health Promotion in Nursing Pratice* (Pender et al., 2011), utilizados na análise dos grupos focais

Comportamentos individuais e experiências	Conhecimento e sentimentos sobre o comportamento específico	Resultados dos comportamentos de saúde
Nesta dimensão mobilizaram-se os discursos sobre os comportamentos anteriores e os fatores pessoais, biológicos, socioculturais que são suscetíveis de influenciar o comportamento.	Nesta dimensão mobilizam-se os dados sobre sentimentos experienciados pelos jovens, perceção de benefícios e as barreiras para o comportamento, autoeficácia, influências interpessoais e situacionais.	Nesta dimensão mobilizam-se os discursos sobre as propostas de estratégias úteis no desenho de programas intervenção, para promover comportamentos sexuais seguros.

Estes constructos integradores do modelo HPM de Pender sugerem-nos possibilidades interpretativas e decerto permitem-nos identificar aspetos e variáveis a considerar para o delineamento de um projeto de intervenção da promoção da SSR para estudantes do ensino superior.

2.5. Considerações éticas

A investigação foi desenvolvida em conformidade com as recomendações éticas inerentes a todos os processos de investigação científica, onde o respeito pela qualidade ética dos procedimentos representou uma preocupação adicional, dada a natureza particularmente

sensível da temática em estudo. O protocolo de investigação foi submetido e aprovado pela Comissão Nacional de Proteção de Dados (Autorização nº 7409/2013 - *Apêndice F),* assim como aprovado pela Comissão de Ética da universidade onde decorreu o estudo (Parecer nº 2/2012 - *Apêndice G)*. A investigação foi desenvolvida em conformidade com as recomendações da Comissão Nacional de Proteção de Dados relativas às questões consideradas sensíveis e o documento de consentimento informado respeitou as regras de conduta propostas pela *Declaração de Helsínquia*. No estudo quantitativo, o questionário foi de preenchimento voluntário, sem identificação nominal, sendo aposto apenas um código numérico, pelo que em nenhum momento foi possível a identificação dos participantes. No estudo qualitativo, todos os participantes assinaram o consentimento informado *(Apêndice H)*, expressando a sua disponibilidade em participar no estudo, depois de informados sobre os objetivos do mesmo. O questionário de caracterização foi de preenchimento voluntário e anónimo. Aos participantes foi garantida a confidencialidade e utilização exclusiva dos dados apenas no âmbito da presente investigação.

CAPÍTULO 3

ESTUDOS EMPÍRICOS METODOLÓGICOS

CAPÍTULO 3

ESTUDOS EMPÍRICOS METODOLÓGICOS

3.1. Artigo 1 - Adaptação portuguesa e validação da versão reduzida da Condom Use Self-efficacy Scale

Santos, M.J., Ferreira, E., Duarte, J., & Ferreira, M. (2017).
Revista Internacional de Andrologia, 15 (1), 23-30. doi:10.1016./j.androl.2016.06.002

ADAPTAÇÃO PORTUGUESA E VALIDAÇÃO DA VERSÃO REDUZIDA DA *CONDOM USE SELF-EFFICACY SCALE*

PORTUGUESE ADAPTATION AND VALIDATION OF THE BRIEF VERSION OF CONDOM USE SELF-EFFICACY SCALE

Maria José Santos[1]; Elisabete Ferreira[2]; João Duarte[3]; Manuela Ferreira[3]

[1] Escola Superior de Enfermagem de Vila Real, Universidade de Trás-os-Montes e Alto Douro, Vila Real, Portugal. mjsantos@utad.pt

[2] Faculdade de Psicologia e de Ciências da Educação, Universidade do Porto, Porto, Portugal

[3] Escola Superior de Saúde de Viseu, Instituto Politécnico de Viseu, Viseu, Portugal

Resumo

Introdução: O preservativo é o método mais eficaz na prevenção de infeções sexualmente transmissíveis. É reconhecido o papel da autoeficácia na predição de comportamentos de saúde e a autoeficácia para usar preservativo tem-se mostrado um constructo chave relacionado com o uso efetivo do preservativo.

Objetivos: Proceder à adaptação e validação da versão reduzida da *Condom Use Self-efficacy Scale* (CUSES) para estudantes do ensino superior português.

Material e métodos: Foi realizado um estudo quantitativo, descritivo-correlacional numa amostra de conveniência de 1946 estudantes do ensino superior, 64% raparigas e 36% rapazes, com idade média de 21 anos (20,74±2,32).

Resultados: A análise das características psicométricas da escala foi avaliada com recurso à análise fatorial exploratória (AFE) e confirmatória (AFC), realizada em dois subgrupos aleatorizados da amostra inicial. Os resultados da AFE revelaram uma estrutura com quatro fatores, semelhante à escala original, que explicam 70,6% da variância e uma boa consistência interna (α=0,86). A AFC revelou a necessidade de ajustamento do modelo aos dados, apresentando o modelo modificado índices aceitáveis de ajustamento. A versão modificada revelou valores adequados de fiabilidade, validade fatorial e validade concorrente e discriminante.

Conclusões: As propriedades psicométricas avaliadas permitem considerar a utilização deste instrumento no desenvolvimento de programas de saúde sexual e reprodutiva para estudantes do ensino superior português, pois permite determinar os domínios relevantes da perceção da autoeficácia para usar o preservativo.

Palavras-chave: Autoeficácia, preservativo, estudantes do ensino superior, saúde sexual e reprodutiva.

Abstract

Introduction: The condom is most effective method to prevent sexually transmitted infections. It is recognized the role of self-efficacy in predicting health behaviours and self-efficacy to use condoms has been shown to be a key related construct to the effective use of condoms.

Objectives: Adaptation and validation of the reduced version of Condom Use Self-efficacy Scale (CUSES) for Portuguese college students.

Material and methods: We conducted a quantitative, descriptive and correlational in a convenience sample of 1946 university students study, 64% girls and 36% boys, mean age 21 years (20.74 ± 2.32).

Results: The analysis of the psychometric characteristics of the scale was carried using exploratory (EFA) and confirmatory factor analysis (EFC), held in two randomized subgroups of the original sample. The results of EFA revealed a structure with four factors, which explains 70.6% of variance and have a good values of internal consistency (α=0.86). The AFC revealed the need to adjust the model to the data, presenting acceptable levels of adjustment. The modified version had good reliability, factorial validity and concurrent and discriminant validity.

Conclusions: The psychometric properties assessed allow considering the use of this instrument in the development of sexual and reproductive health programs for students of Portuguese higher education as it allows determining the relevant fields of self-efficacy perception to use condom.

Keywords: Self-efficacy, condoms, college students, sexual and reproductive health.

Introdução

O início da idade adulta, com acesso ao ensino superior, é uma fase de particular vulnerabilidade face aos riscos envolvidos nos comportamentos sexuais, resultado não só do desenvolvimento psicossexual (Cavanhaug, 2005), mas também do contexto académico onde os comportamentos sexuais podem envolver risco para a saúde, nomeadamente relações sexuais sem preservativo, com parceiros ocasionais e associadas ao consumo de álcool e drogas (Kann et al., 2014; Logan, Koo, Kilmer, Blayney & Lewis, 2015; Reis, Ramiro, Matos & Diniz, 2011; Shepherd et al., 2010; Singh, Darroch & Ashford, 2014). A vulnerabilidade desta fase de experimentação reflete-se nas taxas prevalência das principais infeções sexualmente transmissíveis (IST) e gravidez indesejada nesta faixa etária. Em Portugal, as taxas mais elevadas de VIH/SIDA situam-se nos jovens adultos (11,2% - 20 a 24 anos e 18,8% - 25 a 29 anos) (Martins & Shivaj, 2014) e a prevalência da infeção por HPV é de 19,4%, com o valor máximo de 28,8% nas jovens entre os 18-24 anos (Pista et al., 2011). Relativamente às restantes IST, os dados nacionais não se encontram sistematizados, mas provavelmente Portugal segue a tendência dos países da União Europeia, onde a infeção por *Chlamydia* é das mais frequentes, com cerca de 350 mil casos, dos quais cerca de 75% é diagnosticada em jovens com menos de 25 anos. Considerando que estas infeções têm custos significativos a nível individual e para os serviços de saúde, o incentivo ao uso consistente do preservativo deve ser considerado uma estratégia fundamental dos programas de saúde sexual e reprodutiva (SSR) (Walsh, Fielder & Carey, 2013). O uso do preservativo de forma correta e consistente é altamente eficaz não só na prevenção da gravidez indesejada mas também da transmissão sexual do HIV/SIDA (Lopez, Otterness, Chen, Steiner & Gallo, 2013) e outras IST (Centers for Disease Control and Prevention [CDC], s.d.; Holmes, Levine & Weaver, 2004). Contudo, vários estudos observaram uma baixa utilização do preservativo por jovens, quer norte-americanos (Kann et al., 2014) quer Europeus (Reis et al., 2011), cuja taxa de utilização não ultrapassava os 60%. O último estudo de âmbito nacional com estudantes universitários (Reis et al., 2011) revelou que a situação em Portugal é mais grave, dado que apenas 32,8% utilizou o preservativo de forma consistente nos últimos 12 meses. A preocupação com as elevadas taxas de prevalência das IST e, em particular, a infeção pelo VIH/SIDA, tem permitido o aparecimento de modelos teóricos que procuram abordar os determinantes da mudança de comportamentos de risco sexual. Conforme documentado numa revisão sistemática (Shepherd et al., 2010), a medida de autoeficácia mais comummente utilizada para avaliar alterações comportamentais na prevenção das IST em jovens foi a autoeficácia para usar o preservativo. O conceito de autoeficácia de Bandura (1977), é definido como a crença ou expectativa de que é possível, através do esforço individual, realizar com sucesso tarefas

específicas que possam exigir esforço e perseverança face a situações adversas (Bandura, 2006). Este conceito tem sido identificado em diversos estudos como um importante preditor da utilização do preservativo e capacidade de negociação de sexo seguro (Artistico, Oliver, Dowd, Rothenberg & Khalil, 2014; Asante & Doku, 2010; Casey, Timmermann, Allen, Krahn & Turkiewicz, 2009; French & Holland, 2013). A *Condom Used Self-efficacy Scale* (CUSES) (Brafford & Beck, 1991)*,* foi desenvolvida para avaliar a confiança de estudantes universitários norte americanos para usar preservativo. A escala é composta por 28 itens que pretendem avaliar a autoeficácia dos estudantes para comprar, aplicar, remover e negociar o uso do preservativo com o parceiro sexual. Os autores observaram que os indivíduos com maior experiência sexual, que usavam preservativo e sem história clínica de IST apresentavam pontuações mais elevadas. A análise das características psicométricas da CUSES revelou bons valores de fiabilidade (alfa de *Cronbach* =0,91; fiabilidade teste-reteste =0,81, após 2 semanas) (Brafford & Beck, 1991). Uma análise posterior realizada por Brien, Thombs, Mahoney e Wallnau (1994), numa amostra de 339 estudantes universitários, permitiu obter uma versão reduzida com 15 itens, organizados em quatro dimensões (mecanismos, desaprovação do parceiro, assertividade e intoxicantes), que revelaram uma boa consistência interna para as quatro dimensões observadas (valores de alfa de *Cronbach*= 0,78, 0,81, 0,80 e 0,82, respetivamente), assim como para escala global (α=8,85). Existe ampla evidência da utilização da CUSES em diversos contextos culturais em populações universitárias. Brien e colaboradores (1994) avaliaram a capacidade discriminante da CUSES para diferentes utilizadores do preservativo em estudantes norte americanos e Barkley e Burns (2000) discutem a validação da CUSES numa amostra multicultural de estudantes. Foram, ainda, realizados estudos da sua validade para estudantes universitários do Gana (Asante & Doku, 2010) e da Etiópia (Shaweno & Tekletsadik, 2013), entre outros. alpha

Sabe-se que um bom preditor do uso do preservativo só poderá ser usado no planeamento de intervenções em saúde se for fiável, válido e adaptado à realidade cultural do país. Com o presente estudo pretende-se realizar a adaptação e validação da versão reduzida da CUSES (Brien et al., 1994) para estudantes do ensino superior, dado que desconhecemos a existência no contexto português de um instrumento que permita avaliar este constructo.

Material e métodos

Participantes

Foi realizado um estudo do tipo descritivo-correlacional, transversal, utilizando uma amostra de conveniência de 1 946 estudantes (64% raparigas e 36% rapazes), com idade média de 21 anos (20,74±2,32), de uma universidade do norte de Portugal. A amostragem foi feita por

grupos *Cluster sampling,* onde a unidade de amostra foi a turma. Da amostra inicial, foram considerados os 1936 questionários que se encontravam preenchidos corretamente. A maioria dos participantes era solteira (97,6%), proveniente da cidade (40%), de famílias com baixo rendimento (57%, ≤ 2 salários mínimos) e com baixo nível de escolaridade (54,5% das mães e 61,6% dos pais têm apenas o ensino básico). Cerca de dois terços dos estudantes (77,2%) referem ter tido relações sexuais no último ano e utilizar contraceção de forma regular (♀=97,1%; ♂=94,4%), mas apenas 39,6% dos estudantes menciona que usou o preservativo de forma consistente nas relações sexuais nos últimos 12 meses.

Instrumentos

Na recolha de dados foi utilizado um questionário de autopreenchimento para registar aspetos sociodemográficos e comportamentos sexuais e reprodutivos (previamente definidos na caracterização da amostra) e a versão reduzida da CUSES (Brien et al., 1994) (CUSES-R). A CUSES-R é composta por 15 itens, avaliados numa escala tipo *Likert* com cinco opções (0 – "*discordo totalmente"* a 4 – "*concordo totalmente"*), com uma pontuação total que pode variar entre 0 e 60 pontos; sendo que às pontuações mais elevadas (após inversão dos itens redigidos na negativa: itens - 5, 6, 8, 9 e 10), corresponde uma maior autoeficácia para usar o preservativo. A CUSES-R tem demonstrado boas características psicométricas de validade e fiabilidade nos diversos estudos.

Procedimentos

Para aplicação dos instrumentos de colheita de dados foi solicitada autorização à direção das escolas universitárias onde foi realizado o estudo e enviado o protocolo de investigação. O questionário foi aplicado em sala de aula, no final da componente letiva, a todos os estudantes que voluntariamente anuíram a participar, depois de informados sobre os objetivos e asseguradas as questões de anonimato e confidencialidade. O estudo foi autorizado pela Comissão Nacional de Proteção de Dados (Autorização nº 7409/2012) e pela Comissão de Ética da Universidade (Parecer nº 2/2012).

Antes de se iniciar o processo de tradução e validação do instrumento, foi solicitada autorização aos autores. No processo de tradução e adaptação do instrumento de inglês para português, foram adotadas as orientações de autores de referência (Beaton, Bombardier, Guillemin & Ferraz, 2000; WHO, 2011), nomeadamente a tradução preliminar, revisão por painel de especialistas, retroversão e pré-teste do instrumento na população alvo. O pré-teste foi realizado numa amostra de 173 estudantes, tendo sido introduzidas pequenas alterações de sintaxe. A versão portuguesa final da versão reduzida da *Condom Use Self-efficacy Scale* passou a ser denominada de CUSES-RP.

A análise estatística foi realizada no programa IBM SPSS *Statistics* (v. 21) e IBM AMOS (v. 22). No tratamento estatístico consideramos os procedimentos e critérios apontados por diversos autores (Kline, 2004; Marôco, 2010; 2011; Worthington & Whittaker, 2006), nomeadamente a análise da validade pela AFE, com rotação ortogonal dos fatores pelo método *varimax*, o estudo da fiabilidade pela homogeneidade dos itens e alfa de *Cronbach* ($\alpha \geq 0{,}70$) e correlações de *Pearson.* Na análise fatorial confirmatória (AFC), o ajustamento do modelo foi estimado pelo do método da máxima verosimilhança e testado pela razão entre o qui-quadrado e os graus de liberdade (Χ2/gl), considerando os seguintes índices de ajustamento e critérios de corte: *Comparative Fit Index* (CFI>0,90), *Goodness-of-fit Index* (GFI>0,90), *Standardized Root Mean Square Residual* (SRMSR<0,08) e *Root Mean Square Error of Approximation* (RMSEA<0,06). Foi avaliada a validade convergente pela variância extraída média (VEM), e a discriminante comparando a VEM com a correlação de *Pearson* ao quadrado e a fiabilidade do constructo avaliada pela fiabilidade compósita (FC). Consideramos como valores de referência (Marôco, 2010): VEM>0,5, FC≥0,7 e existência de validade discriminante, quando a correlação ao quadrado entre os fatores é menor do que a VEM para cada fator. Na análise estatística a amostra total (n=1936) foi dividida aleatoriamente em duas metades, tendo sido realizada a AFE com a primeira metade (n=952) e a AFC com a segunda metade (n=984).

Resultados

Os resultados da avaliação da estrutura fatorial da escala com recurso à AFE encontram-se sumariados na Tabela 1.

Os valores de *Kaiser-Meyer-Olkin* (KMO=0,867) e do teste de esfericidade de *Bartlett* (χ^2=7381,2 p<0,001) calculados indicam a adequação da amostra para análise fatorial (Marôco, 2011). O critério do valor próprio (*eigenvalue),* apontou para uma solução com quatro fatores, os mesmos que a escala original, que no seu conjunto explicam 70,6% da variância total.

Tabela 1.
Estrutura fatorial da versão portuguesa da versão reduzida da *Condom Use Self-efficacy Scale* (CUSES-RP)

Nº Item	Itens	Carga Fatorial				H^2
		Fator 1	Fator 2	Fator 3	Fator 4	
9	Não me sinto confiante para sugerir o uso de preservativo a um novo parceiro(a) pois temo que ele/ela pense que tenho uma infeção sexualmente transmissível	0,861				0,794
10	Não me sinto confiante para sugerir o uso de preservativo a um novo parceiro pois temo que ele/ela pense que eu possa achar que ele/ela tem uma infeção sexualmente transmissível	0,851				0,756
8	Não me sinto confiante para sugerir o uso de preservativo a um novo parceiro (a) pois temo que ele/ela pense que tive uma experiência homossexual	0,845				0,762
6	Caso não tivesse certeza da opinião do meu parceiro (a) em relação ao uso do preservativo não seria capaz de sugerir o seu uso	0,738				0,574
5	Se tivesse de sugerir o uso de um preservativo a um parceiro teria medo que ele/ela me rejeitasse	0,687				0,500
11	Estou confiante na minha capacidade de colocar um preservativo em mim ou no meu parceiro rapidamente		0,846			0,745
1	Estou confiante na minha capacidade de colocar um preservativo em mim ou no meu parceiro		0,785			0,737
7	Estou confiante que seria capaz de retirar corretamente o preservativo depois de termos relações sexuais		0,752			0,579
14	Estou confiante na minha capacidade para usar um preservativo corretamente		0,668			0,645
15	Estou confiante que seria capaz de colocar um preservativo em mim ou no meu parceiro mesmo no "calor da paixão"		0,638			0,581
4	Estou confiante que seria capaz de sugerir o uso de um preservativo sem que o meu parceiro se sentisse doente			0,791		0,715
2	Estou confiante na minha capacidade de discutir o uso do preservativo com qualquer parceiro que possa ter			0,781		0,752
3	Estou confiante que seria capaz de sugerir o uso do preservativo a um novo parceiro			0,777		0,782
13	Estou confiante que seria capaz de me lembrar de usar um preservativo mesmo depois de ter consumido drogas				0,900	0,837
12	Estou confiante que seria capaz de me lembrar de usar um preservativo mesmo depois de ter bebido álcool				0,863	0,830
	Medida de adequação da amostra Kaiser-Meyer-Olkin	0,867				
	Teste de esfericidade de Bartlett	$\chi 2$ =7381,2 p<0,001				
	Valores próprios iniciais	5,469	2,747	1,311	1,070	
	% Variância explicada	23,1	20,5	14,9	12,0	
	Coeficiente alfa Cronbach total	0,861				

H^2- Comunalidades

O fator 1 - "desaprovação do parceiro" (itens 5, 6, 8, 9, 10), avalia o medo do que um novo parceiro possa pensar sobre a utilização do preservativo; o fator 2 - "mecanismos" (itens 1, 7, 11, 14, 15), avalia a confiança para utilizar o preservativo num encontro sexual; o fator 3 - "assertividade" (itens, 2, 3, 4), avalia a confiança para discutir a utilização do preservativo com um novo parceiro sexual; e o fator 4 - "intoxicantes" (itens 12, 13), avalia a confiança

para utilizar o preservativo sob o efeito de álcool ou drogas. Os valores das comunalidades são na sua maioria elevados (Marôco, 2011), variando entre 0,501 para o item 5 e 0,837 para o item 13, indicando que uma boa parte da variância dos resultados de cada item é explicada pela solução fatorial. Os PF dos itens são também significativos, situando-se entre um mínimo de 0,638 para o item 15 e um máximo de 0,900 para o item 13. Não foram excluídos itens dado que todos obtiveram PF adequados, todavia observou-se uma alteração na alocação do item 15 "*Estou confiante que seria capaz de colocar um preservativo em mim ou no meu parceiro mesmo no "calor da paixão*", que abandona a dimensão teórica "intoxicantes", e passa a integrar a dimensão teórica "mecanismos", onde apresenta PF claramente superior (0,638).

A CUSES-RP apresenta uma elevada fiabilidade (Tabela 2), quer para o total da escala (α=0,86), quer para as subescalas (F1 - "desaprovação do parceiro", α=0,87; F2 - "mecanismos", α=0,85; F3 - "assertividade", α=0,82 e F4 - "intoxicantes" α=0,82).

Tabela 2.
Análise da fiabilidade (valores de alfa de *Cronbach*, médias das correlações inter-itens e amplitude das correlações item-total corrigidas) para a versão reduzida portuguesa da *Condom Use Self-efficacy Scale* (CUSES-RP)

Fatores	Alfa de *Cronbach*	Média das correlações inter-item	Amplitude das Correlações item-total corrigidas
**Desaprovação do parceiro**	0,87	0,567	0,578-0,793
**Mecanismos**	0,85	0,522	0,581-0,704
**Assertividade**	0,82	0,606	0,630-0,742
**Intoxicantes**	0,82	0,694	0,696-0,700
**CUSES-RP Total**	0,86	0,292	0,338-0,665

Verificou-se, ainda, que nenhum dos itens aumentava a consistência interna do respetivo fator se este fosse eliminado. Os valores da média de correlação inter-itens para as subescalas são moderados a elevados, situam-se entre 0,522 do F2 - "mecanismos" e 0,694 do F3 - "assertividade", evidenciando a homogeneidade dos itens que constituem a escala. A análise da amplitude das correlações item-total corrigidas revelou valores a variar entre 0,578 (item 5) e 0,793 (item 9), ambos referentes à dimensão "desaprovação do parceiro". Da análise das correlações *Pearson* entre os diversos fatores e a nota global da escala (Tabela 3), observaram-se valores de correlação fracos a moderados entre os quatro fatores (variando entre 0,096 e 0,507) e correlações fortes dos quatro fatores com o total da escala (F1 - "desaprovação do parceiro" com r=0,691, F2 - "mecanismos" com r=0,792, F2 - "assertividade" com r=0,748), excetuando o F4 - "intoxicantes" que apresenta uma correlação moderada (r=0,557), com diferenças estatisticamente significativas (p<0,01).

Tabela 3.
Correlação de *Pearson* entre os diferentes fatores que compõem a versão reduzida portuguesa da *Condom Use Self-efficacy Scale* (CUSES-RP)

Fatores	Desaprovação do parceiro	Mecanismos	Assertividade	Intoxicantes	CUSES-RP Total
**Desaprovação do parceiro**	1	0,227**	0,467**	0,096**	0,691**
**Mecanismos**		1	0,507**	0,418**	0,792**
**Assertividade**			1	0,250**	0,748**
**Intoxicantes**				1	0,557**

**p<0,01

A AFC da estrutura de quatro fatores da CUSES-RP, na amostra global, revelou que os valores de ajustamento do índice $x^{2/}$gl =6,029, pode ser considerado sofrível, embora possa existir algum enviesamento relacionado com a elevada sensibilidade destes índices ao tamanho amostral (Marôco, 2010), mas os restantes índices podem ser considerados bons, de acordo com os critérios adotados (CFI=0,945; GFI=0,935; RMSEA=0,072; SRMR=0,050). O resultado do modelo com os índices de mordicação propostos (Figura 1a), revela associação dos erros de medida 1 vs. 2 e 9 vs. 10, o que pode ser sugestivo de problemas de colinearidade entre os itens que lhes corresponde. Contudo, com a associação dos mesmos observou-se uma melhor adequação nos índices de ajustamento global (x2/gl=5,379; GFI=0,943; CFI=0,953; RMSEA=0,067; SRMR=0,051; IC 90%). O modelo de segunda ordem (Figura 1b) apresenta pior qualidade dos índices de ajustamento mas, mesmo assim, com valores aceitáveis, com exceção para os índices x2/gl e SRMR (x2/gl=6,765; GFI=0,929; CFI=0,937; RMSEA=0,077; SRMR=0,086; IC 90%), mostrando a invariância configuracional do modelo de segunda ordem. O cálculo da VEM para cada um dos fatores revela que a validade convergente é adequada para todos os fatores (VEM_{F1}=0,589, VEM_{F2}=0,524, VEM_{F3}=0,669, VEM_{F4}=0,731). A comparação dos valores de VEM com o quadrado da correlação de *Pearson* entre fatores indica que todos os fatores apresentam validade discriminante (Tabela 4). A fiabilidade do constructo avaliada pela FC indica também uma fiabilidade adequada para todos os fatores (FCF1=0,873, FCF2=0,845, FCF3=0,858, FCf4=0,844). A autoeficácia para usar o preservativo manifesta-se com maior intensidade na “assertividade” (β=0,98) e menor intensidade nos “intoxicantes” (β=0,21).

Discussão

Na presente investigação, a análise da fiabilidade da CUSES-RP, para estudantes do ensino superior portugueses, revelou valores de alfa de *Cronbach* considerados bons, quer para a escala total (α=0,86), quer para as diferentes subescalas, o que evidência a sua fiabilidade.

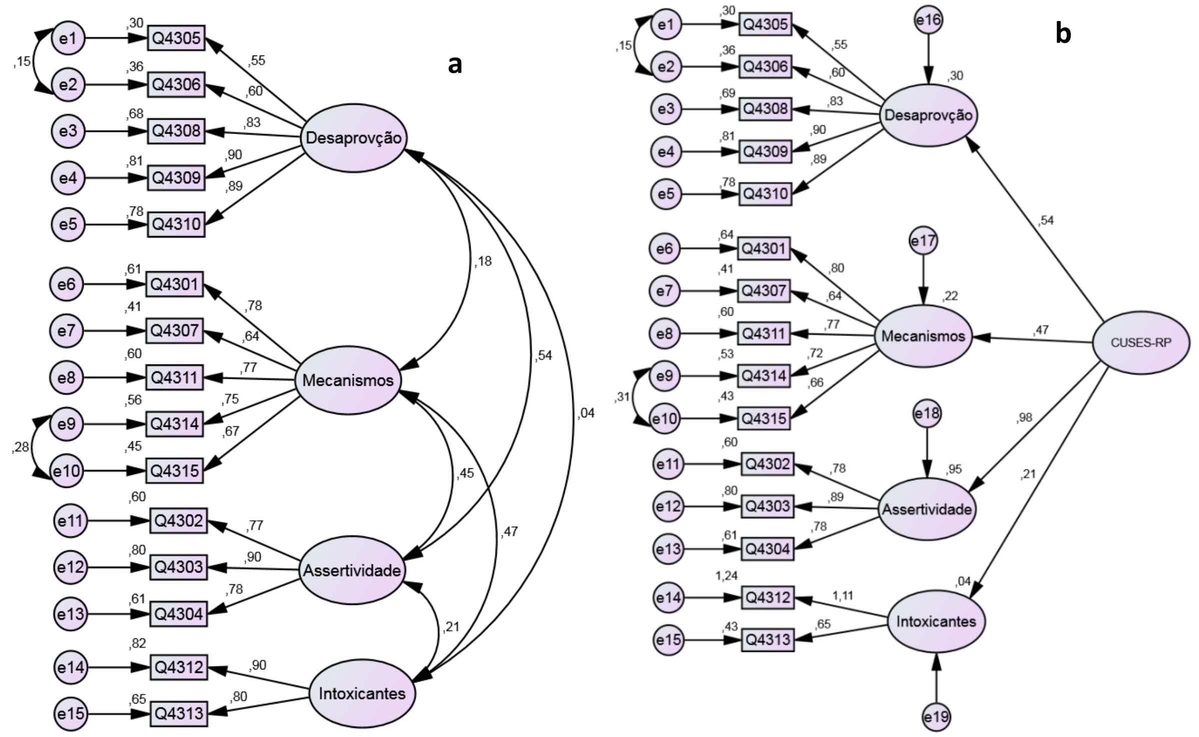

Figura 1. Pesos fatoriais, fiabilidade individual e correlações entre fatores da CUSES-RP do modelo com índices de modificação (a) e modelo de segunda ordem da CUSES-RP (b)

Tabela 4.
Fiabilidade compósita, variância extraída média e validade discriminante da versão reduzida portuguesa da *Condom Use Self-efficacy Scale* (CUSES-RP).

Fatores	*FC*	*VEM*	Validade discriminante		
			F2	F3	F4
**F1- Desaprovação**	0.873	0.589	0.032	0.291	0.001
**F2 - Mecanismos**	0.845	0.524	-	0.202	0.220
**F3 - Assertividade**	0.858	0.669		-	0.044
**F4 - Intoxicantes**	0.844	0.731			-

A análise da estrutura fatorial da CUSES-RP aponta para uma estrutura de quatro fatores, concordante com a estrutura da CUSES-R (Brien et al., 1994), onde todos os itens apresentam PF>0,50. Na presente validação o item 15, que integrava na escala original a dimensão “intoxicantes”, passou a integrar dimensão “mecanismos”, indicando que os estudantes não colocam ao mesmo nível o efeito de substâncias psicoativas e o efeito da excitação sexual. Essa mudança configura-se como coerente, pois não é expectável que a excitação sexual tenha um efeito sobre a cognição e perceção dos riscos tão extenso como o álcool ou as drogas. Decorrente dessa mudança do item 15, o F4 - “intoxicantes” ficou com apenas dois itens, contudo, os seus PF são elevados (item 12=0,900 e item 13=0,863), indicativo de uma estrutura bem definida (Worthington & Whittaker, 2006), pelo que optamos por manter esta dimensão da escala. Em termos conceptuais, esta opção é apoiada pelo

facto do consumo de substâncias psicoativas estar relacionado com o uso inconsistente do preservativo em estudantes universitários (Logan et al., 2015; Reis et al., 2011), reforçando mais uma vez a importância desta dimensão no constructo geral da autoeficácia para usar o preservativo. Adicionalmente, as correlações positivas existentes entre o total da escala e os respetivos fatores foram estatisticamente significativas, o que suporta a conclusão de que os fatores avaliam o mesmo constructo. A AFC revela a necessidade de ajustamento do modelo aos dados, apresentando o modelo modificado índices mais aceitáveis de ajustamento, considerando os valores de corte adotados. Foi encontrada validade convergente e discriminante para todos os factores e confirmada a validade de constructo pelos valores adequados da AFC. A análise do modelo de segunda ordem indica que a assertividade é o aspecto mais importante na confiança para usar o preservativo, e o uso de drogas ou álcool o menos importante. Consideramos manter a denominação original dos quatro fatores, uma vez que os 15 itens da escala se agrupam em fatores teoricamente significativos e representativos do constructo em análise.

As diferenças encontradas na estrutura da escala após o processo de validação poderão ser resultado de diferenças culturais existentes entre os jovens norte americanos e portugueses, mais do que resultado de eventuais distorções no processo de tradução. O estudo das características psicométricas da escala revelou tratar-se de um instrumento com bons níveis de fidelidade e validade, que permitem considerar a sua utilização na investigação com estudantes do ensino superior portugueses. As limitações do estudo estão relacionadas com o facto do processo de amostragem não ter sido aleatório, limitando a generalização dos resultados e as medidas de intenção comportamental serem auto-referidas, podendo não ser uma medida precisa do comportamento real. A validade externa também pode ter sido comprometida por utilizarmos apenas estudantes de uma universidade, não representando a população de estudantes do ensino superior a nível nacional. Contudo, esta limitação pode ter sido minimizada pelo facto dos estudantes serem provenientes de diversas regiões do país. É importante continuar o processo de avaliação das qualidades psicométricas da escala, nomeadamente a avaliação da estabilidade temporal e testar a versão modificada numa amostra independente e mais representativa do grupo em estudo.

Conclusões

O estudo inicial das características psicométricas da versão CUSES-RP revelou valores de validade e fiabilidade promissores. A realização da AFC permitiu sustentar as dimensões fatoriais do instrumento e obter dados relativamente à validade de constructo. A estrutura fatorial final adequa-se razoavelmente aos dados e assemelha-se à da escala original. A adaptação cultural e validação da CUSES-RP para estudantes do ensino superior

portugueses, pode ser um contributo para disponibilizar um instrumento preciso e de fácil aplicação, que pode ser útil no planeamento de intervenções de saúde, onde seja importante avaliar a confiança dos jovens adultos para utilizar o preservativo.

Responsabilidades éticas

Proteção de pessoas e animais. Os autores declaram que para esta investigação não se realizaram experiências em seres humanos e/ou animais.

Confidencialidade dos dados. Os autores declaram que não aparecem dados de pacientes neste artigo.

Direito à privacidade e consentimento escrito. Os autores declaram que não aparecem dados de pacientes neste artigo.

Conflito de interesses

Os autores declaram não haver conflito de interesses.

Referências bibliográficas

Artístico, D., Oliver, L. S., Dowd, S., Rothenberg, A., & Khalil, M. (2014). The predictive role of self-efficacy, outcome expectancies, past behavior and attitudes on condom use in a sample of female college students. *Journal of European Psychology Students, 5*(3), 100-107. doi:http://doi.org/10.5334/jeps.cl

Asante, K. O., & Doku, P. (2010). Cultural adaptation of the Condom Use Self Efficacy Scale (CUSES) in Ghana. *BMC Public Health, 10*, 1-7. doi:10.1186/1471-2458-10-227

Bandura, A. (1977). *Self-efficacy: The exercise of control.* New York: Freeman.

Bandura, A. (2006). Guide for constructing self-efficacy scales. In F. Pajares & T. Urdan (Eds.), *Self-efficacy beliefs of adolescents* (pp. 307-337). Charlotte: Information Age Publishing.

Barkley, T., & Burns, J. (2000). Factor analysis of the condom use self-efficacy scale among multicultural college students. *Health Educucation Research, 15*(4), 485-489. doi: 10.1093/her/15.4.485

Beaton, D. E., Bombardier, C., Guillemin, F., & Ferraz, M. B. (2000). Guidelines for the process of cross-cultural adaptation of self-report measures. *Spine, 25*(24), 3186-3191. doi: 10.1097/00007632-200012150-00014

Brafford, L. J., & Beck, K. H. (1991). Development and validation of a condom self-efficacy scale for college students. *Journal of American College Health, 39*(5), 219-225. doi: 10.1080/07448481.1991.9936238

Brien, T. M., Thombs, D. L., Mahoney, C. A., & Wallnau, L. (1994). Dimensions of self-efficacy among three distinct groups of condom users. *Journal of American College Health*, 42(4), 167-174. doi:10.1080/07448481.1994.9939665

Casey, M., Timmermann, L., Allen, M., Krahn, S., & Turkiewicz, K. (2009). Response and self-efficacy of condom use: A meta-analysis of this important element of AIDS education and prevention. *Southern Communication Journal, 74*(1), 57-78. doi:10.1080/10417940802335953

Cavanhaug, J. (2005). *Adult development and aging*. California: Cole Publishing Company.

Centers for Disease Control and Prevention. (s.d.). C*ondoms and STDs: Fact sheet for public health personnel. Retirado de* http://www.cdc.gov/condomeffectiveness/docs/condoms_and_stds.pdf

French, S., & Holland, K. (2013). Condom negotiation strategies as a mediator of the relationship between self-efficacy and condom use. *Journal of Sex Research, 50*(1), 48-59. doi: 10.1080/00224499.2011.626907

Holmes, K. K., Levine, R., & Weaver, M. (2004). Effectiveness of condoms in preventing sexually transmitted infections. *Bulletin of the World Health Organization, 82*(6), 454-461. Retirado de https://www.ncbi.nlm.nih.gov/pmc/articles/PMC2622864/pdf/15356939.pdf

Kann, L., Kinchen, S., Shanklin, S. L., Flint, K. H., Kawkins, J., Harris, W. A.., … Centers for Disease Control and Prevention. (2014). Youth risk behavior surveillance - United States, 2013. *MMWR, 63*(4), 1-168.

Kline, R. B. (2004). Principles and practice of structural equation modeling (2nd ed.). New York: The Guilford Press.

Logan, D. E., Koo, K. H., Kilmer, J. R., Blayney, J. A., & Lewis, M. A. (2015). Use of drinking protective behavioral strategies and sexual perceptions and behaviors in U.S. College Students. *Journal of Sex Research, 52*(5), 558-569. doi:10.1080/00224499.2014.964167

Lopez, L. M., Otterness, C., Chen, M., Steiner, M., & Gallo, M. F. (2013). Behavioral interventions for improving condom use for dual protection. *Cochrane Database System Review, 10*, CD010662. doi:10.1002/14651858.CD010662.pub2

Marôco J. (2011). *Análise estatística com o SPSS Statistics* (5a ed.). Lisboa: Report Number.

Marôco, J. (2010). *Análise de equações estruturais*. Lisboa: Report Number.

Martins, H. C., & Shivaj, T. (2014). *Infeção VIH/SIDA: Situação em Portugal a 31 de dezembro de 2013*. Lisboa: Instituto Nacional de Saúde Doutor Ricardo Jorge.

Pista, A., Oliveira, C. F., Cunha, M. J., Paixão, M. T., Real, O., & CLEOPATRE Portugal Study Group. (2011). Prevalence of human papillomavirus infection in women in Portugal: The CLEOPATRE Portugal study. *International Journal of Gynecological Cancer, 21*(6), 1150-1158. doi:10.1097/IGC.0b013e31821dd3b2

Reis, M., Ramiro, L., Matos, M. G., & Diniz, J. A. (2011). Saúde sexual e reprodutiva dos estudantes do ensino superior: Dados nacionais 2010. Problemas emergentes e modelo compreensivo. Aventura Social. Lisboa: Edições FMH, 2011. http://aventurasocial.com/arquivo/1303147187_2b-HBSC%20Universitarios%202010_11.pdf

Shaweno, D., & Tekletsadik, E. (2013). Validation of the condom use self-efficacy scale in Ethiopia. *BMC International Health and Human Rights, 13*(22), 1-8. doi:10.1186/1472-698X-13-22

Shepherd, J., Kavanagh, J., Picot, J., Cooper, K., Harden, A., Barnett-Page, E., … Price, A. (2010). The effectiveness and cost-effectiveness of behavioural interventions for the prevention of sexually transmitted infections in young people aged 13-19: a systematic review and economic evaluation. *Health Technology Assessment, 14*(7), 1-206. doi:10.3310/hta14070

Singh, S., Darroch, J. E., & Ashford, L. S. (2014). *The costs and benefits of investing in sexual and reproductive health*. New York: Guttmacher Institute and UNFPA.

Walsh, J., Fielder, R., & Carey, M. (2013). Changes in women's condom use over the first year of college. *Journal of Sex Research, 50*(2), 128-138. doi:10.1080/00224499.2011.642024

World Health Organization. (2011). *Process of translation and adaptation of instruments*, Retirado de http://www.who.int/substance_abuse/research_tools/translation/en/

Worthington, R., & Whittaker, T. (2006). Scale development research. A content analysis and recommendations for best practices. *The Counseling Psychologist, 34*(6), 806-838. doi:10.1177/0011000006288127

CAPÍTULO 3

ESTUDOS EMPÍRICOS METODOLÓGICOS

3.2. Artigo 2 - Adaptação portuguesa e validação da *Sexual Sensation Seeking Scale* para estudantes do ensino superior

Santos, M.J., Duarte, J., Ferreira, E., & Ferreira, M.

(Submetido à *Revista de Enfermagem Referência)*

ADAPTAÇÃO PORTUGUESA E VALIDAÇÃO DA *SEXUAL SENSATION SEEKING SCALE* (SSSS) PARA ESTUDANTES DO ENSINO SUPERIOR

PORTUGUESE ADAPTATION AND VALIDATION OF THE SEXUAL SENSATION SEEKING SCALE (SSSS) FOR COLLEGE STUDENTS

ADAPTACIÓN PORTUGUESA Y VALIDACIÓN DE LA *SEXUAL SENSATION SEEKING SCALE* (SSSS) PARA LOS ESTUDIANTES DE EDUCACIÓN SUPERIOR

Maria José Santos[1]; João Duarte[2]; Elisabete Ferreira[3]; Manuela Ferreira[4]

[1] Escola Superior de Enfermagem de Vila Real, Universidade de Trás-os-Montes e Alto Douro, Vila Real, Portugal. mjsantos@utad.pt

[2,4] Escola Superior de Saúde de Viseu, Instituto Politécnico de Viseu, Viseu, Portugal

[3] Faculdade de Psicologia e de Ciências da Educação, Universidade do Porto, Porto, Portugal

Resumo

Introdução: A utilização de instrumentos de avaliação de traços de personalidade é importante, não só na compreensão dos comportamentos sexuais, mas também no desenvolvimento de intervenções de enfermagem que visem a prevenção de comportamentos sexuais de risco.

Objetivos: Proceder à adaptação e validação da *Sexual Sensation Seeking Scale* (SSSS) para estudantes do ensino superior portugueses.

Metodologia: As características psicométricas da SSSS foram avaliadas utilizando a análise fatorial exploratória (AFE) e confirmatória (AFC), em dois subgrupos aleatorizados da amostra inicial. Da amostra global (n=1946), consideramos os 1500 estudantes que já tinham iniciado relações sexuais.

Resultados: A AFE revelou uma estrutura fatorial composta por nove itens distribuídos por dois fatores, que explicam 54,80% da variância dos resultados e uma boa consistência interna (α=0,789). Os resultados da AFC revelam que o modelo modificado, após a eliminação de dois itens, apresenta índices de qualidade de ajustamento globais e locais adequados.

Conclusão: Este instrumento possui bons níveis de validade e de fidelidade, que permitem considerar a sua utilização para avaliar a busca de sensações sexuais em jovens adultos.

Palavras-chave: Busca sensações sexuais, estudantes do ensino superior, risco sexual, estudos de validação, intervenção de enfermagem.

Abstract

Introduction: The use of instruments for the evaluation of personality traits is important not only in the understanding of sexual behaviors but also in the development of nursing interventions viewing the prevention of sexual risk behaviors.

Objectives: Adaptation and validation of the Sexual Sensation Seeking Scale (SSSS) for Portuguese college students.

Methods: The psychometric characteristics of SSSS were avaluete using exploratory factorial analysis (EFA) and confirmatory factorial analysis (CFA), in two randomized subgroups. From the global sample (n=1946) we considered 1500 students who had already started having sex.

Results: The EFA revealed a factorial structure composed of nine items, distributed by two factors, which explains 54.80% of results' variance and a good internal consistency (α=0.789). The CFA results show that the modified model, after elimination of two items has adequate global and local adjustment quality indices.

Conclusion: The instrument has good validity and fidelity parameters, which allow us to consider its use to evaluate the search for sexual sensation seeking in young adults.

Keywords: Sexual sensation seeking, college students, risk behaviours, nursing care, validation studies.

Resumen

Introducción: El uso de instrumentos de evaluación de los riesgos de la personalidad es importante no sólo en la comprensión de la conducta sexual, sino también en el desarrollo de las intervenciones de enfermería para la prevención de la conducta sexual de riesgo.

Objetivos: Proceder a la adaptación y validación de la Sexual Sensation Seeking Scale (SSSS) para los estudiantes de educación superior portugueses.

Métodos: Las características psicométricas de la SSSS se evaluaron utilizando un análisis fatorial exploratorio (AFE) y confirmatorio (AFC), en dos subgrupos aleatorizados de la muestra inicial. De la muestra total (n=1946), consideramos solo los 1500 estudiantes que ya habían iniciado relaciones sexuales.

Resultados: La AFE reveló una estructura que consta de nueve ítems, distribuidos en dos factores que explican 54,80% de la varianza de los resultados y una buena consistencia interna (α=0,789). Los resultados de la AFC muestran que el modelo modificado después de

la eliminación de los dos ítems presenta un ajuste de los índices de calidad global y local apropiado.

Conclusión: El instrumento tiene un buen nivel de validez y fiabilidad, para considerar su uso para la evaluación de la búsqueda de sensaciones sexuales en los adultos jóvenes.

Palabras clave: Búsqueda de sensaciones sexuales, estudiantes de educación superior, comportamientos sexuales de riesgo, estudios de validación; intervención de enfermería.

Introdução

Embora o procurar riscos e experimentar atividades novas seja considerado normal na juventude, os estudantes do ensino superior apresentam maior propensão para procurar novas experiências sociais, nomeadamente em contexto de festas académicas, envolvendo-se em relacionamentos sexuais com parceiros ocasionais, sexo sem preservativo e práticas sexuais associadas ao consumo de álcool e drogas (Burnett et al., 2014; Logan, Koo, Kilmer, Blayney & Lewis, 2015). Também no contexto académico português, têm vindo a ser identificados comportamentos sexuais de risco em estudantes, designadamente o uso inconsistente do preservativo nas relações amorosas de curta duração, relações sexuais desprotegidas com parceiros ocasionais e a associação de álcool e/ou drogas com as relações sexuais (Cunha-Oliveira, Cunha-Oliveira, Pita & Massano-Cardoso, 2009; Reis, Ramiro, Matos & Diniz, 2013).

A procura de sensações em contexto de atividades sexuais tem sido alvo de diversos estudos, dado que esta predisposição para a procura de sensações variadas, complexas e intensas no âmbito da sexualidade apresenta algum risco. Na população universitária, tem sido estudada, fundamentalmente, a relação da busca de sensações sexuais com a exposição às infeções sexualmente transmissíveis (IST), tendo sido observadas pontuações mais elevadas na procura de sensações sexuais em estudantes com comportamentos sexuais de risco, nomeadamente não utilizar preservativo ou com parceiros ocasionais (Gaither & Sellbom, 2003; Gullette & Lyons, 2006). Alguns trabalhos têm destacado a importância de traços de personalidade, não só na compreensão do comportamento sexual, mas também no desenvolvimento de intervenções de prevenção do HIV/SIDA e outras IST (Hendershot, Stoner, George & Norris, 2007). Esta dimensão da personalidade permite compreender os comportamentos de jovens, em particular os que representam transgressão das normas sociais, como os que estão associados ao risco, a partir da valorização que o jovem faz da procura de novas experiências e emoções intensas (Arnett, 1994). Desta forma, o constructo de busca de sensações não é considerado apenas como a necessidade individual dos jovens experimentarem situações de risco, mas também uma forma de

socialização. Com o presente estudo pretende-se realizar a adaptação cultural e validação da *Sexual Sensation Seeking Scale* (SSSS) para estudantes do ensino superior português.

Enquadramento

Cada indivíduo possui características ou traços de personalidade que o definem e que o diferenciam das outras pessoas. São essencialmente as características internas, relacionais ou sociais que permitem aos indivíduos agir de determinada forma perante uma situação (McMartin, 1995). Embora a maioria da investigação sobre comportamentos sexuais de risco tenha sido orientada pelas teorias cognitivas e comportamentais que procuram explicar a mudança de comportamentos de saúde, nas últimas décadas verifica-se um interesse crescente no papel das disposições da personalidade de busca de sensações sexuais enquanto mediador dos comportamentos de risco sexual, incluído um maior risco para a aquisição de IST (Hendershot et al., 2007; Teva, Bermúdez & Buela-Casal, 2010; Voisin King, Schneider, Diclemente & Tan, 2012). O conceito de busca de sensações foi inicialmente proposto por Zuckerman (1971), referindo-se à necessidade dos indivíduos viverem experiências variadas, novas, complexas e intensas, apenas pelo desejo de assumir riscos físicos e sociais, com o intuito de satisfazer as suas necessidades pessoais. A investigação tem demonstrado que as pessoas com maior predisposição para a busca de sensações preferem contextos sociais que envolvam atividades de prazer e recreação e que tenham associada uma componente de risco social, físico, legal e até financeiro (Pechorro et al., 2016).

A busca de sensações sexuais é uma dimensão específica da busca de sensações, e foi adaptada por Kalichman e Rompa (1995), a partir da *Sensation Seeking Scale* (Zuckerman, 1971). A SSSS é uma escala que procura avaliar a propensão do indivíduo para o envolvimento em experiências e sensações sexuais novas e variadas. A escala tem sido testada em diferentes países e populações, incluindo estudantes universitários (Gaither & Sellbom, 2003), jovens adultos (Hendershot et al., 2007) e adolescentes (DiClemente et al., 2010; Teva et al., 2010). Recentemente, a SSSS foi validada para Portugal por Pechorro *e* colaboradores (2016), numa amostra de adultos. Os diversos estudos têm demonstrado que os indivíduos com níveis superiores de busca de sensações sexuais apresentam uma atitude de maior permissividade sexual (Gaither & Sellbom, 2003; Hendershot et al., 2007), comportamentos de infidelidade, consumo de pornografia e procura de sexo na internet (Perry, Accordino & Hewes, 2007), consumo de álcool ou drogas associado a relações sexuais e maior risco de transmissão de IST (Gullette & Lyons, 2006). Foi também encontrada uma forte associação entre uma maior busca de sensações e a persecução de um sistema de valores hedonista. Nesses indivíduos, verifica-se uma procura constante de

estimulação e uma forte motivação para o uso de álcool e drogas (Voisin et al., 2012). É neste contexto que se justifica a utilização de instrumentos de medida validados para a população portuguesa, que podem representar uma mais-valia no planeamento de intervenções de promoção da saúde sexual e reprodutiva (SSR), específicos para a população em estudo.

Questão de investigação

O presente estudo tem como questão de investigação: A adaptação portuguesa da Escala de Busca de Sensações Sexuais (SSSS) para estudantes do ensino superior apresenta propriedades psicométricas adequadas?

Metodologia

Foi realizado um estudo transversal e analítico numa amostra composta por 1946 estudantes a frequentar uma universidade do norte do país, no ano letivo de 2013/2014. A amostragem foi realizada por grupos *Cluster sampling,* onde o grupo ou a unidade de amostra foi a turma. A recolha de dados foi realizada após o envio do protocolo de investigação e a aprovação do estudo pelos órgãos da direção da universidade onde foi realizado o estudo.

O questionário foi aplicado em sala de aula, no final da componente letiva, a todos os estudantes que voluntariamente mostraram disponibilidade para participar, depois de informados sobre os objetivos do estudo e depois de estarem asseguradas as questões de anonimato e confidencialidade. Os pormenores de preenchimento constantes da folha de instruções do protocolo de investigação foram transmitidos a todos os estudantes, de forma a evitar a rejeição de questionários em virtude do seu incorreto preenchimento.

A realização do estudo salvaguardou, nos diferentes momentos do processo, os princípios ético-deontológicos consignados na Declaração de Helsínquia e na legislação em vigor, que rege a pesquisa realizada com seres humanos. O estudo foi autorizado pela Comissão Nacional de Proteção de Dados (Autorização nº 7409/2013) e pela Comissão de Ética da universidade onde foi realizado (Parecer nº 2/2012).

Foi utilizado um questionário de autopreenchimento que permitiu recolher dados sobre os aspetos sociodemográficos (género, idade, nacionalidade, proveniência, escolaridade e rendimento dos pais), académicos (área científica de estudo) e comportamentos sexuais e reprodutivos (vida sexual ativa, utilização de contraceção, método contracetivo utilizado, consistência de utilização do preservativo, atividade sexual sobre efeito de álcool e/ou drogas ou com parceiros ocasionais, realização do teste de VIH e de IST), e a *Sexual*

Sensation Seeking Scale (SSSS) de Kalichman e Rompa (1995). A SSSS é uma escala de autopreenchimento unidimensional, em que os 11 itens convergem para avaliar o constructo geral de busca de sensações sexuais. O comportamento de busca de sensações sexuais é avaliado numa escala com quatro opções de resposta (1- *Nunca se aplica a mim* até 4- *Muitas vezes aplica-se a mim*), permitindo obter uma pontuação entre 11 e 44 pontos. Todas as questões são formuladas no sentido da adesão ao comportamento de risco, pelo que uma pontuação mais elevada indica uma maior propensão para busca de sensações sexuais. O estudo psicométrico realizado pelos autores da escala revelou uma boa consistência interna da escala (alfa de *Cronbach* de 0,79), assim como o estudo de validação da SSSS em estudantes universitários norte americanos (Gaither & Sellbom, 2003), onde também revelou características psicométricas adequadas (alfa de *Cronbach* foi de 0,83 para os rapazes e 0,81 para as raparigas).

Antes de iniciar o processo de adaptação cultural linguístico do instrumento, foi confirmada a autorização dos autores, que mencionaram que a escala é de utilização livre. O processo de tradução e adaptação do instrumento seguiu as orientações de vários autores (WHO, 2011), que recomendam a tradução preliminar de inglês para português, revisão por painel de especialistas, retroversão e pré-teste do instrumento. As diferentes versões (original, tradução e retroversão) foram comparadas e a versão pré-final foi avaliada por dois especialistas na área da SSR e um em psicologia, relativamente à equivalência semântica, idiomática e conceptual, permitindo obter uma versão de consenso. Esta versão de consenso foi submetida a pré-teste numa amostra de 173 estudantes para avaliar dificuldades de compreensão e interpretação do conteúdo dos seus itens, tendo sido introduzidas pequenas alterações de sintaxe. A versão final da *SSSS* adaptada para a população portuguesa passou a ser denominada por SSSS-P.

A análise estatística foi realizada no programa IBM SPSS *Statistics* (v.21), e IBM SPSS AMOS (v.22). A validade de constructo foi analisada por análise fatorial exploratória (AFE), pelo método *varimax*. Na escolha da estrutura fatorial foram consideradas as orientações definidas por Marôco (2014): o critério de *Keiser* (valores próprios ≥1,0), o peso fatorial dos itens ser igual ou superior a 0,4, a percentagem da variância total explicada pelos fatores ser no mínimo de 40% e retenção de, pelo menos, três itens por fator. No estudo da fiabilidade, a consistência interna foi avaliada pelo alfa de *Cronbach* ($\alpha \geq 0,70$) e pelo estudo da homogeneidade dos itens com determinação do coeficiente de correlação de *Pearson.* Na análise fatorial confirmatória (AFC), o modelo foi estimado pelo método da máxima verosimilhança e testado pela razão entre o qui-quadrado e os graus de liberdade (χ^2/gl), considerando os seguintes índices de ajustamento e critérios de corte (Marôco, 2010): *Comparative Fit Index* (CFI>0,90), *Goodness-of-fit Index* (GFI>0,90), *Standardized Root*

Mean Square Residual (SRMSR<0,08), *Root Mean Square Residual* (RMR - tanto melhor quanto mais próximo de zero) e *Root Mean Square Error of Approximation* (RMSEA<0,06). A validade convergente foi avaliada pela variância extraída média (VEM), e a discriminante comparando a VEM com o quadrado do coeficiente de correlação de *Pearson* e a fiabilidade do constructo pela fiabilidade compósita (FC). Foram considerados como valores de referência a VEM>0,5, FC≥0,7 e existência de validade discriminante quando o quadrado do coeficiente de correlação entre os fatores foi menor do que a VEM para cada fator. No estudo das características psicométricas da escala, do total dos participantes (n=1946), foram considerados apenas os estudantes que já tinham iniciado relações sexuais coitais (n=1500), uma vez que algumas das situações descritas exigiam que os estudantes tivessem experiência sexual. Por sua vez, esta subamostra foi dividida aleatoriamente, tendo sido realizada a AFE com a primeira metade (n=747) e a AFC com a segunda metade da amostra (n=753).

Resultados

Características da amostra

A amostra deste estudo foi constituída por 1946 estudantes, de diversas áreas científicas de uma universidade do norte do país. Dos participantes, 64% era do género feminino e 36% do género masculino, com média de idades de 21 anos (20,74±2,32). A maioria era de nacionalidade portuguesa (97,3%), solteira (97,6%), proveniente da cidade (40%), de famílias com baixo rendimento (57%, ≤ 2 salários mínimos), baixo nível de escolaridade (54,5% das mães e 61,6% dos pais têm apenas o ensino básico) e com profissões pouco diferenciadas. A maioria dos estudantes referiram ter tido relações sexuais no último ano (76,9%) e utilizar contraceção de forma regular (96,7%). Os rapazes utilizaram preferencialmente o preservativo (32,1%) e as raparigas a contraceção hormonal (52,9%). A percentagem de estudantes que referiu já ter realizado o teste de VIH foi reduzida (14,5%), assim como a daqueles que referiu já ter tido alguma IST (1,3%). Uma percentagem elevada de estudantes reportou envolvimento em comportamentos de risco sexual, nomeadamente sexo sobre a influência de álcool (33,0%), drogas (9,7%), com parceiros ocasionais (32, 0%) e utilização inconsistente do preservativo (60,5%), nos últimos 12 meses.

Análise fatorial exploratória

A análise das características psicométricas da SSSS-P foi realizada na primeira metade aleatória da amostra (n=747), com recurso à análise fatorial de componentes principais com rotação ortogonal de *varimax* (Tabela 1). No estudo da adequação da amostra à AFE, o valor do índice de *Kaiser-Meyer-Olkin* (*KMO=0,825*) e o teste de esfericidade de *Bartlett*

(χ^2=2056,442, p<0,000), indicam correlações significativas entre as variáveis que permitem prosseguir com a análise fatorial (Marôco, 2014). Inicialmente, uma vez que a escala proposta pelos autores apresenta uma estrutura unifatorial, forçou-se a extração de um único componente, mas a variância explicada (38,43%) não cumpriu os critérios estabelecidos. Optamos, então, por realizar uma nova análise, considerando os valores próprios (*eigenvalue*) e a sua distribuição pelas componentes observadas no gráfico de autovalores (*Scree Plot*), que apontou para uma solução de dois fatores que explicam, no seu conjunto, 47,62% da variância total. Face aos resultados, optamos por dar continuidade à validação da SSSS-P como uma medida bifatorial.

Tabela 1.
Constituição dos fatores, níveis de saturação e comunalidades da *Sexual Sensation Seeking Scale* (SSSS-P)

Nº Itens	Itens	Carga Fatorial		Comunalidades
		Fator 1	Fator 2	
11	Gosto de experiências e sensações sexuais novas e excitantes	0,838		0,715
10	Apetece-me explorar a minha sexualidade	0,838		0,702
9	Estou interessado em experimentar novas sensações sexuais	0,813		0,689
1	Gosto de encontros sexuais desinibidos	0,483		0,381
8	Já disse coisas que não eram necessariamente verdade, para levar as pessoas a terem relações sexuais comigo		0,774	0,603
5	No que diz respeito ao sexo, a atração física é mais importante para mim do que conhecer bem a pessoa		0,744	0,557
7	Gosto de ver vídeos pornográficos		0,628	0,471
4	Os meus parceiros sexuais, vêem-me, provavelmente, como alguém que gosta de "correr riscos"		0,559	0,333
6	Aprecio a companhia de pessoas "sensuais"		0,558	0,482
Medida de adequação da amostra *Kaiser-Meyer-Olkin*		0,818		
Teste de esfericidade de *Bartlett*		χ^2=1793,780; p <0,001		
Valor próprio		3,364	1,568	
% de variância explicada		28,58	26,21	54,80

Os valores das comunalidades, de forma genérica, são bons, com exceção dos itens 2 e 3 que apresentam valores de 0,261 e 0,149, respetivamente, apontando para a sua eliminação. A análise dos pesos fatoriais confirmou a eliminação dos itens 2 e 3, por serem inferiores a 0,4. A estrutura fatorial da escala ficou composta por nove itens, distribuídos por dois fatores com uma variância total explicada de 54,80% (F1=28,59% e F2=26,21%). O primeiro fator, constituído por quatro itens (1, 9, 10, 11), engloba aspetos relacionados com a procura de novas sensações sexuais, pelo que foi denominado de "busca de novas sensações sexuais" e o segundo fator, constituído por cinco itens (4, 5, 6, 7, 8), agrega aspetos relacionados com a procura de situações de excitação sexual, foi denominado de "busca de novas experiências sexuais". Na designação dos fatores procuramos refletir o conteúdo conceptual global dos itens que compõem cada um deles. Os valores das correlações inter-itens para as subescalas são moderados, situam-se entre 0,275 e 0,613 para a subescala F1 - "busca de novas sensações sexuais" e 0,195 e 0,434 do F2 - "busca

de novas experiências sexuais". A análise da amplitude das correlações item-total corrigidas revelou valores que variam entre 0,372 (item 4) e 0,668 (item 11). Da análise da fiabilidade da SSSS-P na presente amostra, composta pelos nove itens (Tabela 2), foi possível observar valores de consistência interna moderados para as duas subescalas, assim como para a escala global (α=0,789).

Tabela 2.
Resultados descritivos dos itens e correlação item x total da *Sexual Sensation Seeking Scale* (SSSS-P)

Fator Nº Item	Itens	Média	DP	Correlação item total corrigida	Correlação múltipla ao quadrado	*Alfa* de *Cronbach* se item eliminado
**Fator 1** - **Busca de novas sensações sexuais**						
**1**	Gosto de encontros sexuais desinibidos	2,23	1,086	0,388	0,167	0,818
**9**	Estou interessado em experimentar novas sensações sexuais	2,53	0,992	0,656	0,473	0,670
**10**	Apetece-me explorar a minha sexualidade	2,56	0,980	0,612	0,452	0,695
**11**	Gosto de experiências e sensações sexuais novas e excitantes	2,95	0,923	0,668	0,465	0,669
**Fator 2** - **Busca de novas experiências sexuais**						
**4**	Os meus parceiros sexuais, vêem-me, provavelmente, como alguém que gosta de "correr riscos"	1,54	0,821	0,372	0,159	0,686
**5**	No que diz respeito ao sexo, a atração física é mais importante para mim do que conhecer bem a pessoa	1,56	0,809	0,509	0,278	0,633
**6**	Aprecio a companhia de pessoas "sensuais"	2,48	1,008	0,471	0,235	0,650
**7**	Gosto de ver filmes pornográficos	1,88	0,979	0,480	0,265	0,645
**8**	Já disse coisas que não eram necessariamente verdade, para levar as pessoas a terem relações sexuais comigo	1,31	0,682	0,492	0,276	0,648
**Coeficiente *alfa Cronbach***		Fator 1		0,778		
		Fator 2		0,710		
		Total		0,789		

A análise das correlações *Pearson* entre os dois fatores e, destes, com a classificação global da escala (Tabela 3), revelou valores de correlação moderados (r=0,520) entre os fatores e correlações elevadas dos dois fatores com a classificação global da escala (F1 - "busca de novas sensações sexuais" com r=0,885, F2 - "busca de novas experiências sexuais" com r=0,857), com diferenças estatisticamente significativas (p<0,01).

Tabela 3.
Correlação de *Pearson* entre os diferentes fatores que compõem a versão portuguesa da *Sexual Sensation Seeking Scale* (SSSS-P)

Fatores	Busca de novas sensações sexuais	Busca de novas experiências sexuais	SSSS-P Total
**F1 - Busca de novas sensações sexuais**	1	0,520**	0,885**
**F2 – Busca de novas experiências sexuais**		1	0,857**

**p<0,01

Análise fatorial confirmatória

Visando confirmar se a estrutura fatorial obtida na AFE se adequava aos dados, foi realizada a AFC da SSSS-P com a segunda metade da amostra (n=747). A análise descritiva com os nove itens revelou que os valores de assimetria e curtose apresentam índices em termos absolutos inferiores a 3 (variando entre 0,009 e 1,994) e 7 (variando entre 0,067 e 2,912), respetivamente, pelo que assumimos os pressupostos de normalidade da amostra. Também o coeficiente multivariado de *Mardia* é normal, sendo inferior a 5 (1,025). As trajetórias e os rácios críticos são todos superiores a 1,96 e altamente significativos, pelo que não foi eliminado nenhum item.

No modelo inicial (Figura 1a), de acordo com as suturações dos itens nos fatores, seria de eliminar o item 1 (λ=0,48) do primeiro fator e o item 4 (λ=0,49) do segundo fator, por apresentarem saturações inferiores a 0,5. Contudo, dado que se trata de um estudo preliminar da escala e uma vez que os valores estão no limite do critério de exclusão, decidimos manter ambos os itens na análise. Neste modelo, a maioria dos índices de qualidade de ajustamento global revela-se adequada (GFI=0,941; CFI=0,912; RMR=0,070; SRMR=0,075), com exceção dos índices $x^{2/}$gl=8,600 e RMSEA=0,104. O elevado valor de ajustamento do índice $x^{2/}$gl pode ser explicado pela grande sensibilidade deste índice ao tamanho amostral (Marôco, 2010). Posteriormente, foi realizada a análise do modelo com índices de modificação propostos pelo AMOS (Figura 1b). O modelo revelou problemas de colinearidade entre os erros de medida 5 e 9, observando-se, no entanto, uma melhoria, ainda que ligeira, nos índices de qualidade de ajustamento do modelo, com os índices de ajustamento global a manterem-se bons para GFI=0,952; CFI=0,927; RMR=0,071; SRMR=0,075 e sofríveis para os restantes (x^2/gl=7,534; RMSEA=0,093). Os itens 1 e 4 continuam a apresentar pesos fatoriais inferiores a 0,5, mas os restantes apresentam índices significativos, com coeficientes superiores a 0,62 entre os fatores e as suas manifestações observáveis. A correlação entre os dois fatores é moderada (r=0,49).

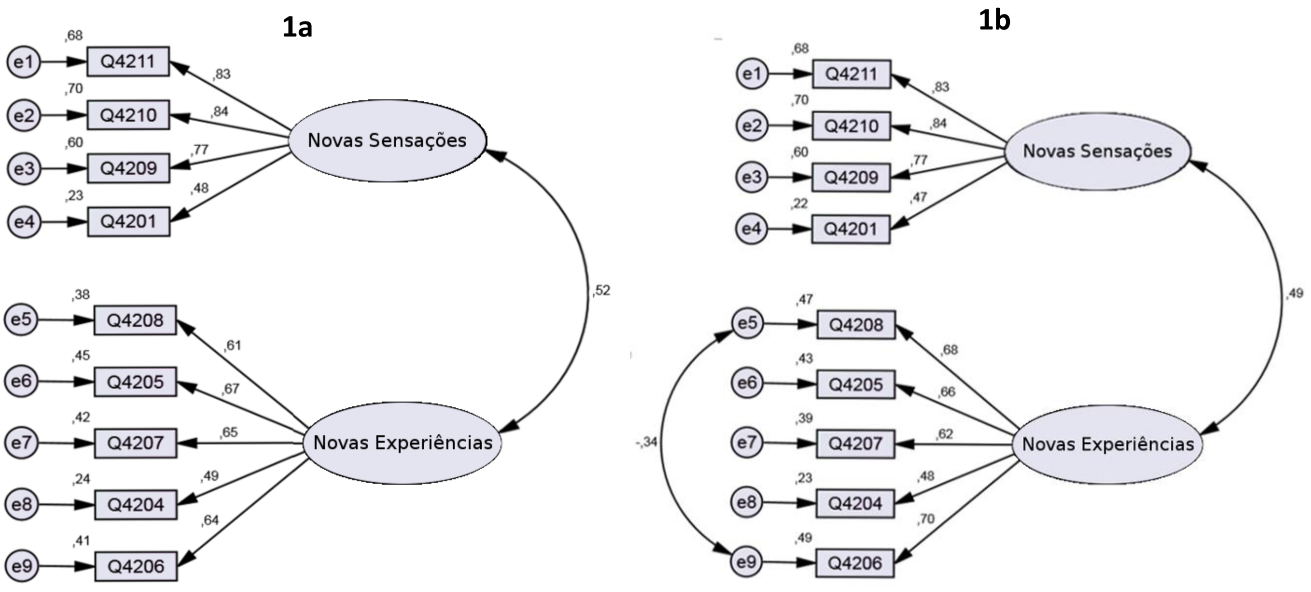

Figura 1. Pesos fatoriais, fiabilidade individual e correlações entre fatores da SSSS-P do modelo inicial (1a) e modelo final (1b)

A validade convergente (Tabela 4), determinada pelo cálculo da VEM para cada um dos fatores, revelou-se adequada para o primeiro fator (VEM F1=0,533) mas não para o segundo (VEM F2=0,402). A fiabilidade do constructo avaliada pela FC, ao apresentar valores acima de 0,7 em ambos os fatores (FC_{F1}=0,826; FC_{F2}=0,768), indicia também uma boa consistência interna dos itens em cada fator. A validade discriminante entre os dois fatores também se revelou adequada (0,240), uma vez que é inferior à VEM para cada um dos fatores. O alfa estratificado é de 0,885 para uma VEM de 0,469, esta última ligeiramente abaixo do valor de referência.

O género e a idade determinaram diferenças significativas na procura de sensações sexuais, uma vez que são os rapazes (♂ 21,88±5,07; ♀ 16,61±4,61; p<0,001) mais velhos (≤19 anos=17,97±5,46; 20 a 24 anos=18,68±5,29; ≥24 anos=19,36±5,87; p=0,005), que revelam *scores* mais elevados de busca de sensações sexuais com diferenças estatisticamente significativas.

Tabela 4.
Fiabilidade compósita, variância extraída média e validade discriminante da versão portuguesa da *Sexual Sensation Seeking Scale* (SSSS-P)

Fatores	*FC*	*VEM*	Validade discriminante
**F1 - Busca de novas sensações sexuais**	0,826	0,533	0,240
**F2 - Busca de novas experiências sexuais**	0,768	0,402	

Discussão

Alguns investigadores têm enfatizado a importância das variáveis de personalidade nos comportamentos de risco, bem como a necessidade da sua introdução nos modelos teóricos que visam compreender o envolvimento em riscos sexuais (Hendershot et al., 2007). Nesta perspetiva, a utilização de instrumentos de medida, como a SSSS, validados e adaptadas à realidade portuguesa, podem ser importantes no delineamento de programas de promoção da SSR.

Na análise psicométrica da versão portuguesa da SSSS-P pela AFE, a solução fatorial encontrada difere da versão proposta por Kalichman e Rompa (1995), uma vez que esta análise aponta para uma solução bifatorial, com eliminação de dois itens (itens 2 e 3) e os autores propõem uma estrutura unidimensional. A análise revelou valores promissores ao nível da variância explicada para o global da escala e dos pesos fatoriais, que permitiram considerar a validação desta solução fatorial. Adicionalmente, as correlações moderadas e positivas existentes entre o total da escala e os respetivos fatores foram estatisticamente significativas, o que suporta a conclusão de que os fatores avaliam o mesmo constructo. As correlações item-total corrigidas são adequadas, dado que são superiores a 0,20, que é o valor mínimo considerado aceitável pela literatura (Marôco, 2014), embora menores que as reportadas por Gaither e Sellbom (2003), numa amostra de 546 estudantes universitários. Na validação deste instrumento em populações similares, nomeadamente em adolescentes espanhóis (Teva et al., 2010) e estudantes universitários norte-americanos (Gaither & Sellbom, 2003), os autores mantiveram a proposta da estrutura unifatorial, não obstante realizarem apenas a AFE. As diferenças encontradas na estrutura fatorial da SSSS-P refletem provavelmente o tamanho amostral e as diferenças culturais que devem ser sempre consideradas na adaptação de instrumentos psicométricos para diferentes países.

No presente estudo, quer a AFE quer a AFC apontaram para a exclusão dos itens 2 “as sensações físicas são a coisa mais importante do sexo” e item 3 “gosto da sensação de ter relações sexuais sem preservativo”. A exclusão destes itens pode estar associada às questões de género, uma vez que as raparigas, género predominante na amostra, dão maior importância à componente afetiva das relações e valorizam mais o uso do preservativo como medida de proteção contra a gravidez indesejada e IST. Por outro lado, persistem, ainda, mitos associados à perda de prazer sexual pela utilização do preservativo, com desvalorização da dimensão erótico-hedonista que o preservativo pode ter (Cunha-Oliveira et al., 2009). Os resultados do presente estudo confirmam que o género e a idade são variáveis a considerar quando se estuda a procura de sensações sexuais, uma vez que são os rapazes, e mais velhos (≥24 anos), aqueles que apresentam valores mais elevados

de busca de sensações sexuais, resultados que estão em consonância com os obtidos por outros investigadores (Teva et al., 2010; Zuckerman, 2006).

Os resultados AFC indicam que existe consistência interna adequada para os dois fatores, confirmando a validade do constructo, mas apenas para no primeiro fator a validade convergente é adequada. A solução fatorial final confirmou uma estrutura de dois fatores, sendo que o primeiro fator F1- “busca de novas sensações sexuais” é o mais importante na determinação do constructo em estudo. De uma forma geral, a versão portuguesa da SSSS possui bons níveis de validade e fidelidade, que permitem considerar a sua utilização na investigação com estudantes do ensino superior.

As limitações do presente estudo estão relacionadas com o facto de a técnica de amostragem ser não-probabilística, limitando a generalização dos resultados. Esta limitação pode ter sido minimizada pelo facto de se ter realizado a aleatorização das turmas para a recolha de dados. As medidas de intenção comportamental, sendo auto-referidas, podem também não ser uma medida precisa do comportamento real. Uma outra limitação decorre do uso de uma escala de quatro pontos, em que sem a opção de uma resposta neutral pode ter reduzido a capacidade informativa da análise e ter limitado a qualidade da estimação. Decorrente do exposto, consideramos recomendável continuar o processo de avaliação das qualidades psicométricas da SSSS-P, realizando a avaliação da sua estabilidade temporal e adequação da estrutura bifatorial numa amostra independente daquela onde o modelo foi ajustado e mais representativa da população em estudo.

Conclusão

O estudo inicial das características psicométricas da SSSS-P revelou valores de validade e fiabilidade promissores. A realização da AFC determinou uma estrutura bifatorial, diferente da proposta pelos autores, mas suficientemente robusta para considerar a sua utilização. A estrutura fatorial final, embora diferindo da escala original, adequa-se razoavelmente aos dados. A adaptação cultural e validação da SSSS-P para estudantes do ensino superior portugueses representa um contributo na disponibilização de um instrumento preciso e de fácil aplicação, que pode ser útil no planeamento de intervenções de saúde, onde seja importante avaliar a propensão do indivíduo para procurar riscos sexuais.

Referências bibliográficas

Arnett, J. (1994). Sensation seeking: A new conceptualization and a new scale. *Personality and Individual Differences, 16*(2), 289-296. doi:10.1016/0191-8869(94)90165-1

Burnett, A. J., Sabato, T. M., Walter, K. O., Kerr, D. L., Wagner, L., & Smith, A. (2014). The Influence of attributional style on substance use and risky sexual behaviour among college students. *College Student Journal, 48* (21), 325-336.

Cunha-Oliveira, A., Cunha-Oliveira, J., Pita, J. R., & Massano-Cardoso, S. (2009). A aquisição do preservativo e o seu (não) uso pelos estudantes universitários. *Revista Referência*, *11* (Série 2), 7-22.

DiClemente, R., Milhausen, R. R., Salazar, L. F., Spitalnick, J., Sales, J. M., & Crosby, R. A., … Wingood, G. M. (2010). Development of the sexual sensation-seeking for african american adolescent women. *Internacional Journal of Sexual Health, 22*(4), 248-261. doi:10.1080/19317611.2010.491388

Gaither, G. A., & Sellbom, M. (2003). The sexual sensation seeking scale: Reliablity and validity within a heterosexual college student sample. *Journal of Personality Assessment, 81*(2), 157-167. doi:10.1207/S15327752JPA8102_07

Gullette, D. L., & Lyons, M. A. (2006). Sensation seeking, self-esteem, and unprotected sex in college students. *Journal of Association of Nursing in AIDS Care, 17*(5), 23-31. doi:10.1016/j.jana.2006.07.001

Hendershot, C. S., Stoner, S. A., George, W. H., & Norris, J. (2007). Alcohol use, expectancies and sexual sensation seeking as correlates of HIV risk behaviour in heterosexual young adults. *Psychology of Addictive Behaviors*, *21*(3), 365-372. doi: 10.1037/0893-164X.21.3.365

Kalichman, S. C., & Rompa, D. (1995). Sexual sensation seeking and sexual compulsivity scales: Reliability, validity, and predicting HIV risk behavior. *Journal of Personality Assessment, 65* (3), 586-601. doi:10.1207/s15327752jpa6503_16

Logan, D. E., Koo, K. H., Kilmer, J. R., Blayney, J. A., & Lewis, M. A. (2015). Use of drinking protective behavioral strategies and sexual perceptions and behaviors in U.S. college students. *Journal of Sexual Research, 52*(5), 558-569. doi:10.1080/00224499.2014.964167.

Marôco, J. (2010). *Análise de equações estruturais: Fundamentos teóricos, software & aplicações*. Pêro Pinheiro: Report Number.

Marôco, J. (2014). *Análise estatística com o SPSS statistics*. Pero Pinheiro: Report Number.

McMartin, J. (1995). *Personality psychology: A student-cantered approach*. London: Sage Publications.

Pechorro, P., Pascoal, P., Figueiredo, C., almeida, A. I., Vieira, R., & Jesus, S. (2016). Validação portuguesa da Escala de Busca de Sensações Sexuais. *Revista Internacional de Andrologia*, *13*(4), 1-6. doi:10.1016/j.androl.2014.11.003

Perry, M., Accordino, M. P., & Hewes, R. L. (2007). An investigation of internet use, sexual and nonsexual sensation seeking, and sexual compulsivity among college students. *Sexual Addiction & Compulsivity, 14*(4), 321-335.

Reis, M., Ramiro, L., Matos, M. G., & Diniz, J. A. (2013). Determinants influencing male condom use among university students in Portugal. *International Journal of Sexual Health, 25*(2), 115-127. doi:10.1080/19317611.2012.728554

Teva, I., Bermúdez, M. P., & Buela-Casal, B. (2010). Sexual sensation seeking, social stress, and coping styles as predictors of HIV/STD risk behaviors in adolescents. *Youth & Society*, *42*(2), 255-277. doi:10.1177/0044118X09353572

Voisin, D., King, K., Schneider, J., Diclemente, R., & Tan, K. (2012). Sexual sensation seeking, drug use and risky sex among detained youth. *Journal of AIDS & Clinical Research*, S1, 1-5. doi:10.4172/2155-6113.S1-017

World Health Organization. (2011). *Process of translation and adaptation of instruments*. Retirado de http://www.who.int/substance_abuse/research_tools/translation/en/

Zuckerman, M. (1971). Dimension sensation of seeking. *Journal of Consulting and Clinical Psychology*, *36*(1), 45-52.

Zuckerman, M. (2006). *Sensation seeking and risky behavior*. New York: American Psychological Association.

CAPÍTULO 3

ESTUDOS EMPÍRICOS METODOLÓGICOS

3.3. Artigo 3 - Construção e validação da Escala de Atitudes de Saúde Sexual e Reprodutiva (EASSR) para estudantes do ensino superior

CONSTRUÇÃO E VALIDAÇÃO DE UMA ESCALA DE ATITUDES DE SAÚDE SEXUAL E REPRODUTIVA (EASSR) PARA ESTUDANTES DO ENSINO SUPERIOR

CONSTRUCTION AND VALIDATION OF A SEXUAL AND REPRODUCTIVE HEALTH ATTITUDES SCALE (SRHS) FOR COLLEGE STUDENTS

Maria José Santos[1]; João Duarte[2]; Elisabete Ferreira[3]; Manuela Ferreira[4]

[1] Escola Superior de Enfermagem de Vila Real, Universidade de Trás-os-Montes e Alto Douro, Vila Real, Portugal. mjsantos@utad.pt

[2,4] Escola Superior de Saúde de Viseu, Instituto Politécnico de Viseu, Viseu, Portugal

[3] Faculdade de Psicologia e de Ciências da Educação, Universidade do Porto, Porto, Portugal

Resumo

Introdução: As atitudes face à sexualidade têm-se revelado preditoras consistentes dos comportamentos de proteção face ao risco sexual, sendo, portanto, de primordial importância o estudo dos fatores que as influenciam. A validação de um instrumento de medida neste âmbito permite o planeamento de intervenções que facilitem a mudança de comportamentos sexuais de risco.

Objetivos: Proceder à construção e validação da Escala de Atitudes de Saúde Sexual e Reprodutiva (EASSR), num grupo de estudantes do ensino superior em Portugal.

Metodologia: As qualidades psicométricas da escala foram avaliadas numa amostra não probabilística de 1 946 estudantes do ensino superior, através da análise fatorial exploratória e confirmatória.

Resultados: A análise fatorial exploratória revelou uma estrutura fatorial de 20 itens divididos em cinco fatores, que designamos por: Atitude de responsabilidade, Atitude face ao preservativo, Atitude preventiva, Atitude hedonista, Atitude face às IST. No seu conjunto, os cinco fatores explicam 46,72% da variância total e a sua organização parece representar o constructo em estudo. A escala demonstrou uma boa consistência interna global ($\alpha=0,820$). Pela análise fatorial confirmatória não foi confirmada a validade de constructo e discriminante.

Conclusões: A versão final da EASSR revela propriedades psicométricas adequadas na amostra utilizada, permitindo considerar a sua utilização no desenvolvimento de programas de saúde sexual e reprodutiva em contexto universitário. Contudo, sendo esta a sua primeira validação, devem desenvolver-se novos estudos para validação da estrutura fatorial.

Palavras-chave: Atitude, estudantes ensino superior, estudos de validação, saúde sexual e reprodutiva.

Abstract

Introduction: Attitudes toward sexuality are consistent predictors of sexual risk protective behaviours. Therefore, it is of primary importance the study of the factors that influence these attitudes. The validation of assessment instrument on attitudes toward sexuality allows the planning of interventions to facilitate the change of sexual risk behaviours.

Objectives: To proceed the construction and validation of the Sexual and Reproductive Health Scale (SRHS) in a group of Portuguese college students.

Methodology: The psychometric qualities of the scale were evaluated in a non-probabilistic sample of 1946 college students through exploratory and confirmatory factorial analysis.

Results: The exploratory factorial analysis revealed a factorial structure of 20 items divided into five factors, which we designate: Attitude of responsibility, Attitude towards the condom, Preventive attitude, Hedonistic attitude, Attitude towards STI. Overall, the five factors explain 46.72% of the total variance and their organization seems to represent the construct under study. The scale showed good overall internal consistency (α=0.820). Confirmatory factor analysis did not confirm construct and discriminant validity

Conclusions: The final version of the SRHS reveals adequate psychometric properties in the sample used, allowing its use in the development of sexual and reproductive health programs in a university context. However, once this is its first validation, more studies should be developed to validate the factorial structure of construct.

Keywords: Attitude, college students, validation studies, sexual and reproductive health.

Introdução

A saúde sexual e reprodutiva (SSR) representa, atualmente, uma das principais preocupações da saúde pública, apontando para a necessidade de se intervir nesta área, sobretudo junto das populações mais jovens, com o objetivo de diminuir a gravidez indesejada e as infeções sexualmente transmissíveis (IST) (Khan, Berger, Wells & Cleland, 2012; Kann et al., 2016). O estudo das atitudes face à sexualidade é particularmente importante, uma vez que, de acordo com López e Fuertes (2001), conhecendo-se a estrutura de determinada atitude, torna-se mais fácil compreender os comportamentos sexuais e levar a cabo intervenções que facilitem a mudança dos comportamentos de risco. Conceptualmente, as atitudes podem ser definidas como uma predisposição para emitir uma

opinião, sentir e atuar face a objetos sexuais, situações, pessoas, normas ou costumes sociais e mesmo condutas sexuais (Diéguez Ruibal, López Castedo, Sueiro Domínguez & López Sánchez, 2005; López, 2009; López & Fuertes, 2001). As atitudes não são mais do que avaliações positivas ou negativas que as pessoas têm para com outras pessoas, situações, conceitos ou mesmo fenómenos (Ajzen & Fishbein, 2005). No estudo das atitudes, alguns autores consideram-nas um conceito multidimensional constituído por três componentes: uma componente cognitiva, que inclui as crenças ou ideias, uma componente afetiva, que inclui sentimentos e emoções, e uma componente comportamental, que traduz a predisposições ou intenção de agir, que determinam a forma como o indivíduo atua diante do objeto da atitude (Breckler & Wiggins, 1989; Eagly & Chaiken, 1993).

As atitudes não são inatas, vão sendo adquiridas no processo de socialização em situações de convívio em comunidade e família. Inicialmente, são moldadas pelas influências familiares que vão diminuindo no decorrer da evolução intelectual do indivíduo, durante a adolescência sofrem influência particular do grupo de pares e, na idade adulta, da comunicação social e da comunidade (Kirby, Coyle, Alton, Rolleri & Robin, 2011). A distinção entre atitudes e outros conceitos psicológicos, como crenças e valores, nem sempre é clara, porque muitas vezes estes conceitos sobrepõem-se. De uma forma geral, a atitude diferencia-se das crenças porque inclui sempre a componente afetiva. Daqui resulta que as atitudes face à sexualidade não sejam inteiramente determinadas pelo pensamento racional, sendo afetadas por sentimentos como o amor, a atração sexual, o medo, a insegurança e até mesmo pela perceção de invulnerabilidade (Asare, 2015).

As atitudes podem explicar e prever comportamentos em todos os domínios e são uma componente importante que determina os comportamentos individuais de risco relativamente à saúde, pelo que, na educação para a saúde, tem sido dada particular atenção à manutenção de atitudes favoráveis à saúde (Straub, 2005). Em geral, as atitudes tendem a prever melhor comportamento quando são fortes e consistentes, têm uma relação específica com o comportamento previsto, têm por base as experiências pessoais e o indivíduo tem consciência das suas atitudes (Fishbein & Ajzen, 2010). Contudo, devemos salientar que as crenças e atitudes sobre a sexualidade são distintas do comportamento sexual. Esta aparente incongruência acontece porque, na realidade, o comportamento nem sempre corresponde ou segue logicamente as crenças, valores e atitudes individuais (acreditamos que é possível contrair uma IST, e mesmo assim não adotamos um comportamento preventivo).

As atitudes traduzem a posição do indivíduo face a uma experiência subjetiva, influenciando de forma decisiva o seu comportamento, que resulta da integração das normas sociais onde o indivíduo está inserido (Ajzen & Fishbein, 2005; Fishbein & Ajzen, 2010). No campo da

sexualidade, a atitude é um fator importante para aceitação de determinadas interações sexuais, determinando diferenças significativas entre os diferentes tipos de amor e as vivências da sexualidade (Hendrick & Hendrick, 1987; Juhasz, Kaufman & Meyer, 1986). As atitudes têm vindo a revelar-se como um dos preditores mais consistentes dos comportamentos de proteção (Ajzen & Fishbein, 2005; Albarracín, Johnson, Fishbein & Muellerleile, 2001), incluindo da utilização do preservativo (Asare, 2015; Kang & Moneyham, 2007; Kirby et al., 2011; Reis, Ramiro, Matos & Diniz, 2013). Embora o uso do preservativo não seja apenas determinado pelas atitudes, uma atitude mais positiva contribui para reduzir o risco de IST e envolve preocupações físicas e emocionais. O medo de implicações negativas pela sua utilização como, por exemplo, o sexo parecer ter sido planeado, transmitir a ideia de que existem outros parceiros sexuais, bem como os ideais românticos associados à espontaneidade e ao envolvimento cego na relação amorosa, revelam-se elementos importantes das atitudes face ao preservativo e preditores do seu uso (Alvarez & Garcia-Marques, 2008; Bermúdez et al., 2012; Cunha-Oliveira, Cunha-Oliveira, Pita & Masano-Cardoso, 2013; Ganczak et al., 2007).

Esta presunção da capacidade da atitude poder influenciar a resposta dos indivíduos a um determinado estímulo do meio social e de poder predizer o comportamento tem determinado o interesse de diversos investigadores na procura de técnicas de mensuração e promoção de mudanças atitudinais na área da sexualidade. As atitudes são constructos hipotéticos impossíveis de serem observados de forma direta, por isso, na sua avaliação, são usados instrumentos indiretos que avaliam o comportamento ou a resposta ou posicionamento do indivíduo a um determinado estímulo, sendo possível inferir a atitude subjacente.

Verificamos que existem várias escalas orientadas para avaliação das atitudes sexuais, constructo relacionado, mas distinto do que pretendíamos estudar - saúde sexual e reprodutiva (SSR). Perspetivando as atitudes sexuais como um conceito multidimensional, Hendrick e Hendrick (1987) desenvolveram a Escala de Atitudes Sexuais, instrumento já validado para estudantes universitários portugueses (Alferes, 1999). Mais recentemente, Nelas (2010) construiu e validou também uma escala de atitudes sexuais, também orientada para adolescentes. Por sua vez, Nemèiæ *e* colaboradores (2008) desenvolveram o *Questionnaire Measuring Attitudes Towards Sexual Health*, um instrumento de medida mais próximo do constructo em análise. Este questionário foi desenvolvido para estudantes universitários croatas e é constituído por 50 itens organizados em três domínios: “cuidados pessoais de saúde”, “princípios de comportamento sexual” e “comportamento sexual seguro”. Contudo, não foi considerada a sua adaptação, uma vez que o instrumento não correspondia à estrutura conceptual que pretendíamos avaliar. Neste sentido, a avaliação da atitude de SSR pode ser um alvo privilegiado de estudo, não só por permitir um maior

conhecimento acerca dos processos cognitivos, afetivos e comportamentais envolvidos na atitude face à sexualidade e saúde reprodutiva, mas também porque pode contribuir para o desenvolvimento de estratégias de intervenção, com o objetivo de promover nos jovens adultos atitudes mais positivas face à SSR. Face ao exposto, o objetivo deste trabalho é apresentar os resultados do processo de construção e validação da escala de atitudes de SSR em estudantes do ensino superior.

Metodologia

Foi realizado um estudo descritivo-correlacional, transversal, numa amostra de 1946 estudantes, a frequentar uma universidade do norte do país, no ano letivo de 2013/2014. A amostragem foi realizada por grupos *cluster sampling,* onde o grupo ou a unidade de amostra foi a turma. A recolha de dados foi realizada após envio do protocolo de investigação e aprovação do estudo pelos órgãos da direção da universidade onde foi realizado o estudo. O questionário foi aplicado em sala de aula, a participação no estudo foi de carácter voluntário e foram asseguradas as recomendações éticas, nomeadamente a confidencialidade dos resultados e o anonimato dos participantes. Os pormenores de preenchimento constantes da folha de instruções do protocolo de investigação foram transmitidos a todos os estudantes, de forma a evitar a rejeição de questionários por incorreto preenchimento. Da amostra inicial foram considerados os 1946 questionários que se encontravam preenchidos corretamente, e o tempo médio de preenchimento foi de 45 minutos.

O estudo foi autorizado pela Comissão Nacional de Proteção de Dados (Autorização nº 7409/2013) e pela Comissão de Ética da universidade onde foi realizado (Parecer nº 2/2012). A realização do estudo salvaguardou, nos diferentes momentos do processo, os princípios ético-deontológicos consignados na Declaração de Helsínquia e na legislação em vigor que rege a investigação com seres humanos.

Instrumentos

Considerando a necessidade de dispormos de um instrumento que permitisse avaliar a atitude face à SSR em estudantes do ensino superior, foi construída a Escala de Atitudes de Saúde Sexual e Reprodutiva (EASSR), cujo processo de construção e validação preliminar passamos a descrever.

Na fundamentação e definição do referencial teórico-prático do instrumento, foi realizada pesquisa bibliográfica, privilegiando-se as publicações científicas de autores de referência na área (Abreu, 2010; Matos, Reis, Ramiro & Equipa Aventura Social, 2011; Nemèiæ et al., 2008; Reis & Matos 2007), orientações da World Healh Organization (WHO, 2011), DGS

(2008) e Associação para o Planeamento da Família (APF, 2010) e do Centers of Diseases Control and Prevention (CDC, 2009a, 2009b). Foram também revistos alguns instrumentos de avaliação da atitude face à contraceção e IST e realizadas duas entrevistas com os peritos na área da SSR. Na conceção do instrumento foi realizada a definição das suas dimensões teóricas, redação dos itens e definida a escala de resposta. A versão provisória do instrumento, constituída por 47 itens, foi submetida a apreciação por um painel de cinco peritos com experiência na área para validação do conteúdo através da análise semântica dos itens propostos. Dada a complexidade do tema e a sua transdisciplinaridade, o grupo de peritos foi constituído por dois enfermeiros da prática clínica, dois docentes investigadores em enfermagem e um psicólogo. Após a análise e das sugestões dos peritos, foram eliminados nove itens que, de alguma forma, tinham informação redundante ou marginal ao constructo em estudo. A versão de consenso ficou constituída por 38 itens, 20 com direção positiva e 18 com direção negativa (que assumem a inversão de valores na cotação), para evitar a tendência de resposta. As questões foram operacionalizadas numa escala de *Likert* de 7 pontos (1 – "discordo totalmente" a 7 – "concordo totalmente"), que pretendiam avaliar as dimensões teóricas da atitude de SSR. A versão de consenso foi submetida a pré-teste numa amostra de 173 estudantes de duas instituições do ensino superior, para avaliar dificuldades de compreensão e interpretação do conteúdo dos seus itens, tendo sido introduzidas pequenas alterações de sintaxe, das quais resultou uma boa compreensão do instrumento por todos. A versão final foi denominada de Escala de Atitudes de SSR (EASSR).

Tratamento estatístico

A análise foi realizada no programa IBM SPSS *Statistics* (v. 22), e IBM SPSS AMOS (v.22). A validade de constructo foi analisada por análise fatorial exploratória (AFE), pelo método *Varimax* (Tabachnick & Fidell, 2007). Na escolha da estrutura fatorial foram consideradas as orientações definidas por Worthington e Whittaker (2006) e Marôco (2014): o critério de *Keiser* (valores próprios ≥1,0), o peso fatorial dos itens ser igual ou superior a 0,4, a percentagem da variância total explicada pelos fatores ser no mínimo de 40%, com retenção de pelo menos três itens por fator e a consistência interna dos fatores, determinada pelo alfa de *Cronbach*, deve ser igual ou superior a 0,7.

Na análise fatorial confirmatória (AFC), o modelo foi estimado pelo método da máxima verosimilhança, que assume que os dados têm uma distribuição normal e multivariada, e testado pela razão entre o qui-quadrado e os graus de liberdade (χ^2/gl), considerando os índices de ajustamento e critérios de corte, propostos por Worthington e Whittaker (2006) e Marôco (2014): x2/gl<5; *Comparative Fit Index* (CFI>0,90), *Goodness-of-fit Index*

(GFI>0,90), *Standardized Root Mean Square Residual* (SRMSR<0,08), *Root Mean Square Residual* (RMR<0,08) e *Root Mean Square Error of Approximation* (RMSEA<0,06). No ajustamento do modelo consignaram-se os índices de modificação superiores a 11 propostos pelo AMOS. A validade convergente foi avaliada pela variância extraída média (VEM), a discriminante comparando a VEM com o quadrado da correlação de *Pearson* e a fiabilidade do constructo pela fiabilidade compósita (FC). Foram considerados como valores de referência a VEM>0,5, FC≥0,7 e existência de validade discriminante quando a correlação ao quadrado entre os fatores é menor do que a VEM para cada fator (Marôco, 2011). No estudo das características psicométricas da escala, a amostra total (n=1946) foi dividida aleatoriamente, tendo sido realizada a AFE com a primeira metade (n=940) e a AFC com a segunda metade da amostra (n=998). O número de participantes respeita o rácio de 10:1 (número de sujeito por cada item do questionário), valor recomendado para a obtenção de soluções fatoriais estáveis (Worthington & Whittaker, 2006).

Resultados

Características da amostra

A amostra foi constituída por 1946 estudantes, com predomínio do género feminino (64% vs 36%), com média de idades de 21 anos (20,74±2,32), que frequentavam diversas áreas científicas de uma universidade do norte do país. A maioria era de nacionalidade portuguesa (97,3%), solteira (97,6%), proveniente da cidade (40%), de famílias com baixo rendimento (57%, ≤ 2 salários mínimos), baixo nível de escolaridade (54,5% das mães e 61,6% dos pais têm apenas o ensino básico) e com profissões pouco diferenciadas. Relativamente aos comportamentos sexuais e reprodutivos, a maioria dos estudantes refere ter tido relações sexuais coitais no último ano (76,9%) e utilizar contraceção (96,7%), sendo o método mais utilizado o hormonal (43,7%) e o preservativo (21,8%). A percentagem de estudantes que refere já ter realizado o teste de VIH é reduzida (14,5%), assim como a daqueles que referem já ter tido alguma IST (1,3%). Uma percentagem significativa de estudantes reporta o envolvimento em comportamentos de risco sexual nos últimos 12 meses, nomeadamente sexo sobre a influência de álcool (33,0%), drogas (9,7%), parceiros ocasionais (32,0%) e utilização inconsistente do preservativo (60,5%).

Análise das Propriedades Psicométricas da Escala de Atitude de SSR

Para validar a qualidade psicométrica da EASSR, foram efetuados os estudos de validade e fiabilidade, dividindo a amostra em duas metades aleatórias. A AFE foi realizada numa amostra de 940 estudantes. Numa fase inicial, foi calculado o valor de confiabilidade para o total dos 38 itens que compõem a escala. No que se refere aos coeficientes de correlação

item-total, a maioria dos itens revela bons índices de correlação, com exceção dos itens 4, 7, 27, 36 e 38, que estão aquém do valor crítico de 0,20 definido como o limite mínimo e aceitável do índice de associação. Estes itens apresentam ainda uma correlação corrigida *item-total* que apoia a exclusão do respetivo item, por forma a aumentar a consistência interna da escala, pelo que foram retirados da análise. A escala ficou, assim, composta por 33 itens, com correlação *item-total* superior a 0,20 e cujo valor do alfa de *Cronbach's* diminui se forem excluídos. Assim, a AFE prosseguiu com base nesta estrutura de itens que cumprem os critérios iniciais para permanecerem no procedimento estatístico.

Estudo da validade

A estrutura fatorial da EASSR foi avaliada através da AFE, com extração de fatores pelo método de componentes principais (ACP), com rotação ortogonal de *varimax* (Tabela 1). O teste de adequação da amostra de *Kaiser-Meyer-Olkin* (*KMO*) apresentou um valor de 0,921 e o teste de *Bartlett* estatisticamente significativo ($\chi 2$=8834,291 p<0,000), que permitem realizar a análise fatorial. Os valores das comunalidades encontram-se dentro dos valores de referência, com valores que oscilam entre 0,331 (item 16) e 0,597 (item 1). Assim, podemos assumir que a totalidade dos 35 itens apresenta valores aceitáveis de extração, pelo que procedemos à ACP. Considerando o critério dos valores próprios superiores a 1, a solução fatorial permitiu extrair inicialmente oito fatores que, no seu conjunto, explicam 47,15 % da variância total. Contudo, tendo em consideração a análise do ponto de inflexão do gráfico de autovalores (*Scree Plot*) e do critério dos valores próprios (*eigenvalue*), procedemos à análise fatorial forçada a cinco fatores, de modo a tentar estabelecer um método mais consistente de análise da escala.

Verificou-se que a solução fatorial forçada a cinco fatores explica somente 38,427% de variabilidade total, o que não cumpre os critérios definidos inicialmente. Em adição, observou-se que existem alguns itens cuja aplicação dos critérios iniciais também sugerem a sua eliminação, mais concretamente os itens 13, 14, 16, 25 e 35, por apresentarem pesos fatoriais inferiores a 0,4 e, posteriormente, os itens 6, 9, e 18 pelo mesmo critério.

Deste modo, foi realizada nova AFE com os 25 itens restantes (Tabela 2), alcançando valores de *KMO* elevados (0,895) e de *Bartlett's* que permitem continuar com o procedimento estatístico (p>0,0001), e em cuja análise novamente forçada a cinco fatores se obteve uma percentagem de 46,72% de variância total.

Tabela 1.
Constituição dos fatores/dimensões, níveis de saturação e consistência interna das dimensões e total da Escala de Atitudes SSR (EASSR)

Nº Item	Fatores/Dimensão					
	Atitude Responsabilidade	Atitude Preservativo	Atitude Preventiva	Atitude Hedonismo	Atitude IST	H^2
**1**. A afetividade é muito importante num relacionamento sexual	0,805					0,669
**2**. É importante que os parceiros sexuais falem sobre os métodos de contraceção	0,726					0,612
**5**. Devemos iniciar relações sexuais com um novo parceiro quando temos a certeza que estamos preparados para assumir essa responsabilidade	0,665					0,493
**11**. Considero importante saber o passado sexual do meu parceiro	0,545					0,394
**26**. É divertido ter experiências sexuais ocasionais	0,535					0,421
**3**. Se o meu parceiro sexual tivesse relações sexuais com outra pessoa gostaria de ter conhecimento	0,513					0,360
**17**. O uso do preservativo mostra falta de confiança no nosso parceiro sexual		0,666				0,517
**20**. Se o meu parceiro insistisse para usarmos preservativo ficaria incomodado		0,664				0,505
**19**. Quando conhecemos o nosso parceiro sexual não precisamos de usar o preservativo		0,649				0,428
**24**. É um insulto sugerir ao meu parceiro(a) o uso do preservativo mesmo que para prevenir uma IST		0,618				0,510
**31**. Se o meu parceiro não gosta de utilizar o preservativo devo respeitar a sua vontade e ter relações sexuais sem preservativo		0,608				0,457
**22**. Considero que quando se toma a pílula não é necessário utilizar o preservativo		0,582				0,362
**32**. A possibilidade de vir a ter cancro do útero é motivo suficiente para modificar o meu comportamento sexual			0,620			0,419
**29**. É importante saber se o nosso parceiro sexual realizou o teste do VIH/ SIDA			0,596			0,385
**33**. Se tivesse mais de um parceiro sexual faria testes de saúde com regularidade			0,553			0,465
**23**. Para mim a elevada taxa de IST é uma preocupação			0,537			0,352
**30**. É mais importante a nossa saúde que ter uma breve experiência sexual			0,534			0,345
**34**. Considero importante convencer o meu parceiro a praticar apenas sexo seguro			0,522			0,456
**12**. A masturbação é uma prática sexual saudável				0,720		0,552
**10**. A colocação do preservativo pode ser um jogo erótico				0,689		0,496
**15**. A masturbação é tão normal nas mulheres como nos homens				0,642		0,416
**8**. A satisfação sexual é muito importante num relacionamento amoroso				0,573		0,466
**21**. A maioria das infeções de transmissão sexual não interfere com a relação amorosa					0,769	0,624
**28**. Considero que a maioria das IST não interfere com a vida quotidiana					0,763	0,618
**37**. As IST têm pouco impacto no bem-estar físico e psicológico dos jovens					0,541	0,356
Kaiser-Meyer-Olkin	KMO = 0,892					
Teste de *Bartlett*	χ^2=688,417; p <0,0001					
Valores próprios iniciais	5,221	2,050	1,762	1,401	1,244	
% de variância explicada	11,54	11,04	8,89	8,47	6,78	46,72
Alfa de *Cronbach*	0,747	0,750	0,662	0,640	0,608	
Coeficiente alfa de *Cronbach* Total=0,820						

H^2 - Comunalidades

O agrupamento dos itens indica uma estrutura de cinco fatores, sendo que o primeiro fator designado de “Atitude de responsabilidade”, ficou constituído por seis itens (1, 2, 3, 5, 11 e 26), o segundo fator denominado de “Atitude face ao preservativo”, ficou composto por seis itens (17, 19, 20, 22, 24, 31), o terceiro fator ficou constituído por seis itens (23, 29, 30, 32, 33 e 34), que teoricamente representam a “Atitude preventiva”, o quarto fator denominado de Atitude hedonista, ficou constituído por quatro itens (8, 10, 12 e 15) e, por fim, o quinto fator designado “Atitude face às IST”, ficou composto por três itens (21, 28 e 37). Na designação dos fatores procuramos refletir o conteúdo conceptual global dos itens que compõem cada um deles.

Os valores das comunalidades são bons (Marôco, 2011), situando-se entre 0,345 para o item 30 e o 0,669 para o item 1, indicando que uma boa parte da variância dos resultados de cada item é explicada pela solução fatorial. Os pesos fatoriais dos itens nos fatores cumprem o critério estabelecido, uma vez que são todos superiores a 0,5. Na análise da fiabilidade, verificamos que o valor de consistência interna é adequado (α=0,820).

Procurando saber se a estrutura fatorial da EASSR se adequava aos dados, procedemos à AFC pela estimação da máxima verosimilhança, utilizando a segunda metade da amostra (n=998). Esta análise testa o ajuste relativo a modelos fatoriais concorrentes, sendo por isso de grande valor no processo de revisão e refinamento das suas estruturas fatoriais. A análise descritiva com os 25 itens revelou valores de assimetria (SK) e de achatamento (KU) normais (SK-variando entre 0,372 e 2,764; KU-variando entre 0,090 e 5,139), com exceção do valor de curtose nos itens 2 e 3 (Item 2=7,324; item 3=7,891) que, sendo superiores a 7, deveriam ser eliminados. Contudo, dado que se trata de um estudo preliminar da escala e que os valores estão no limite do critério de exclusão, decidimos manter ambos os itens na análise, e assumir os pressupostos de normalidade.

A análise dos rácios críticos das trajetórias entre os 25 itens e os factores que lhe correspondem revelou que são todos significativos mas, de acordo com as opções metodológicas, aponta para a eliminação dos itens com saturações inferiores a 0,40 (itens 26, 15, 30 e 32). Embora a eliminação dos itens deva ocorrer com valores inferiores a 0,50, dado que se trata de um estudo preliminar da escala, os itens foram mantidos na análise.

No modelo inicial (Figura 1a), a maioria dos índices de qualidade de ajustamento revelaram-se adequados (Tabela 2), com exceção dos índices CFI= 0,887 que revelou um ajustamento sofrível e RMR=0,116 com um ajustamento inaceitável. A análise das saturações dos itens nos fatores confirma a eliminação do item 26 “*É divertido ter experiências sexuais ocasionais*”, do primeiro fator, dos itens 30 “*É mais importante a nossa saúde que ter uma breve experiência sexual*” e 32 “*A possibilidade de vir a ter cancro do útero é motivo*

suficiente para modificar o meu comportamento sexual", ambos do terceiro fator, e do item 15 "*A masturbação é tão normal nas mulheres como nos homens*", que faz parte do quarto fator, por apresentarem saturações inferiores a 0,4. Após a eliminação dos quatro itens, a qualidade de ajustamento do modelo melhora ligeiramente para o CFI=0,909 e mantém-se sofrível para o RMR=0,097.

Efetuado o refinamento do modelo com índices de modificação propostos pelo AMOS (Figura 1b), o modelo revelou problemas de colinearidade entre os erros de medida 19 vs 20 e 19 vs 22, pelo que o item 19 foi eliminado. Após a eliminação deste item, o modelo sem os índices de colinearidade revelou uma melhoria, ainda que ligeira, nos índices de qualidade de ajustamento global (χ^2/gl=2,632; CFI=0,938; RMSEA=0,040; RMR=0,085).

Tabela 2.
Comparação dos resultados dos índices de qualidade de ajustamento dos cinco modelos analisados

Modelo	χ^2/gl	GFI	CFI	RMSEA	RMR	SRMR
**Modelo inicial**	3,147	0,937	0,887	0,046	0,116	0,048
**Modelo com itens eliminados**	3,260	0,947	0,909	0,048	0,097	0,046
**Modelo com índices de modificação**	2,752	0,956	0,931	0,042	0,089	0,043
**Modelo final sem itens com colinearidade**	2,632	0,961	0,938	0,040	0,085	0,043
**Modelo final 2ª ordem**	2,975	0,955	0,922	0,045	0,105	0,050

Quanto à qualidade do ajustamento local, o peso fatorial dos itens apresentados pelos coeficientes lambda indicam índices superiores a 0,48 e a fiabilidade individual dos itens revela valores correlacionais inferiores a 0,25.

O modelo de 2.ª ordem (Figura 1b), mantém valores aceitáveis de qualidade de ajustamento global, com exceção do índice RMR=0,105, mostrando a invariância configuracional do modelo de 2.ª ordem. A dimensão atitude preventiva é a que está mais correlacionado (β=0,80) com a atitude SSR, e a dimensão atitude hedonismo a que está menos correlacionada (β=0,42).

A fiabilidade compósita apresenta índices adequados (acima de 0,70) para os dois primeiros fatores, sofrível para o quarto fator, e inadequada para os restantes, sendo indicador de uma fiabilidade de constructo inapropriada (Tabela 3). A validade convergente, obtida através da VEM, não foi confirmada, uma vez que todos os fatores apresentarem índices inferiores a 0,50.

Tabela 3.
Fiabilidade compósita, variância extraída média e validade discriminante da Escala de Atitudes de SSR (EASSR)

Fatores	FC	VEM	Validade Discriminante									
			1 vs 2	1 vs 3	1vs 4	1vs 5	2 vs 3	2 vs 4	2 vs 5	3 vs 4	3 vs 5	4 vs 5
**Atitude Responsabilidade**	0,721	0,344	0,324	0,476	0,160	0,152						
**Atitude Preservativo**	0,747	0,375					0,348	0,084	0,476			
**Atitude Prevenção**	0,663	0,336								0,129	0,184	
**Atitude Hedonismo**	0,597	0,337										0,032
**Atitude IST**	0,542	0,255										

Quanto à validade discriminante, não se regista entre os fatores $F2_{Preservativo}$ vs $F5_{IST}$ e $F1_{Responsabilidade}$ vs $F3_{Prevenção}$, pois a correlação ao quadrado entre os fatores é maior do que a VEM para cada fator (Marôco, 2014).

De acordo com os resultados obtidos, que apresentamos na Tabela 4, verificamos que as correlações de *Pearson*, entre os cinco fatores e o valor global da escala, são positivas, fortes e significativamente correlacionadas, com valores que oscilam entre r=0,789 ($F2_{Preservativo}$ *vs* atitude SSR total) e r=0,482 ($F4_{Hedonismo}$ *vs* atitude SSR total).

Tabela 4.
Correlação de *Pearson* entre os diferentes fatores que compõem a Escala de Atitude de SSR (EASSR)

Fatores	Atitude Responsabilidade	Atitude Preservativo	Atitude Preventiva	Atitude Hedonismo	Atitude IST	Atitude SSR Total
**Atitude Responsabilidade**	1	0,388**	0,472**	0,278**	0,237**	0,713**
**Atitude Preservativo**		1	0,417**	0,205**	0,445**	0,789**
**Atitude Preventiva**			1	0,277*	0,276**	0,718**
**Atitude Hedonismo**				1	0,119**	0,482**
**Atitude IST**					1	0,616**
**Atitude SSR Total**						1

Nota: **p<0,01

Observaram-se, ainda, correlações positivas, fracas a moderadas entre os cinco fatores, com valores que oscilam ente r=0,472 ($F1_{Responsabilidade}$ vs $F3_{Prevenção}$) e r=0,119 ($F4_{Hedonismo}$ vs $F5_{IST}$).

Procurando analisar as diferenças de género relativas à atitude de SSR, realizamos um teste de comparação de médias (*T-student*), observando-se que, de uma forma geral, a atitude de SSR é elevada, sendo mais positiva para as raparigas, que para os rapazes, com diferenças estatisticamente significativas (♀=121,94±11,16 vs ♂=112,00±16,06, *score* total

EASSR=140 pontos; T-teste=81,594; p<0,001). As dimensões atitude de responsabilidade e atitude face ao preservativo foram as que apresentaram pontuações médias mais elevadas e as dimensões atitude face às IST e hedonismo pontuações médias mais baixas. As raparigas revelam pontuações médias significativamente mais elevadas (p<0,001) em todas as subescalas, com exceção da subescala atitude hedónica onde os rapazes apresentam pontuações superiores mas sem diferenças significativas (♂=17,65±3,01 vs ♀=17,51±3,154; T-teste= 3,091; p=0,079).

Discussão

As atitudes são um componente importante que determina os comportamentos individuais de risco relativamente à saúde, pelo que, na educação para a saúde, tem sido dada particular atenção à melhoria ou manutenção de atitudes favoráveis à saúde (Asare, 2015; Straub, 2005). As atitudes são medidas através das opiniões, por isso torna-se importante encontrar indicadores adequados e relevantes da atitude que vai ser medida. Nesta perspetiva, a construção de um instrumento de medida que permita avaliar as componentes da atitude sexual e reprodutiva, pode ser importante não só na compreensão dos comportamentos sexuais e reprodutivos, mas também no desenvolvimento de intervenções que visem a prevenção de comportamentos sexuais de risco.

Um conjunto de alargado de questões (47 questões) foi gerado a partir da literatura e da contribuição de peritos e foi submetido a um processo de validação, ficando a versão de consenso constituída por 38 questões.

A AFE, realizada com a 1.ª metade aleatória da amostra global, apontou para uma solução fatorial composta por 25 itens, com pesos fatoriais superiores a 0,5 que se agruparam em cinco fatores ($F1_{Responsabilidade}$, $F2_{Preservativo}$, $F3_{Prevenção}$; $F4_{Hedonismo}$ e $F5_{IST}$), que explicam 46,72% de variância total dos dados. As subescalas apresentam uma organização de itens que reflete o conteúdo conceptual em análise, assim o conceito de SSR na sua complexidade pode ser representado pelas cinco dimensões, que incluem questões relacionadas com a assertividade nas relações, comunicação com o companheiro, sexo seguro e IST, comportamentos sexuais de risco, mas também de prevenção e vigilância de saúde e, por último, as questões do erotismo e prazer que indiscutivelmente aparecem sempre associadas à sexualidade e que assumem especial relevância nas faixas etárias mais jovens. A consistência interna da escala global também se revelou adequada (α=0,832), contudo 3 das subescalas ($F3_{Prevenção}$; $F4_{Hedonismo}$ e $F5_{IST}$), apresentavam valores baixos de alfa de *Cronbach,* justificado em parte pelo menor número de itens que as constituem (Marôco, 2011).

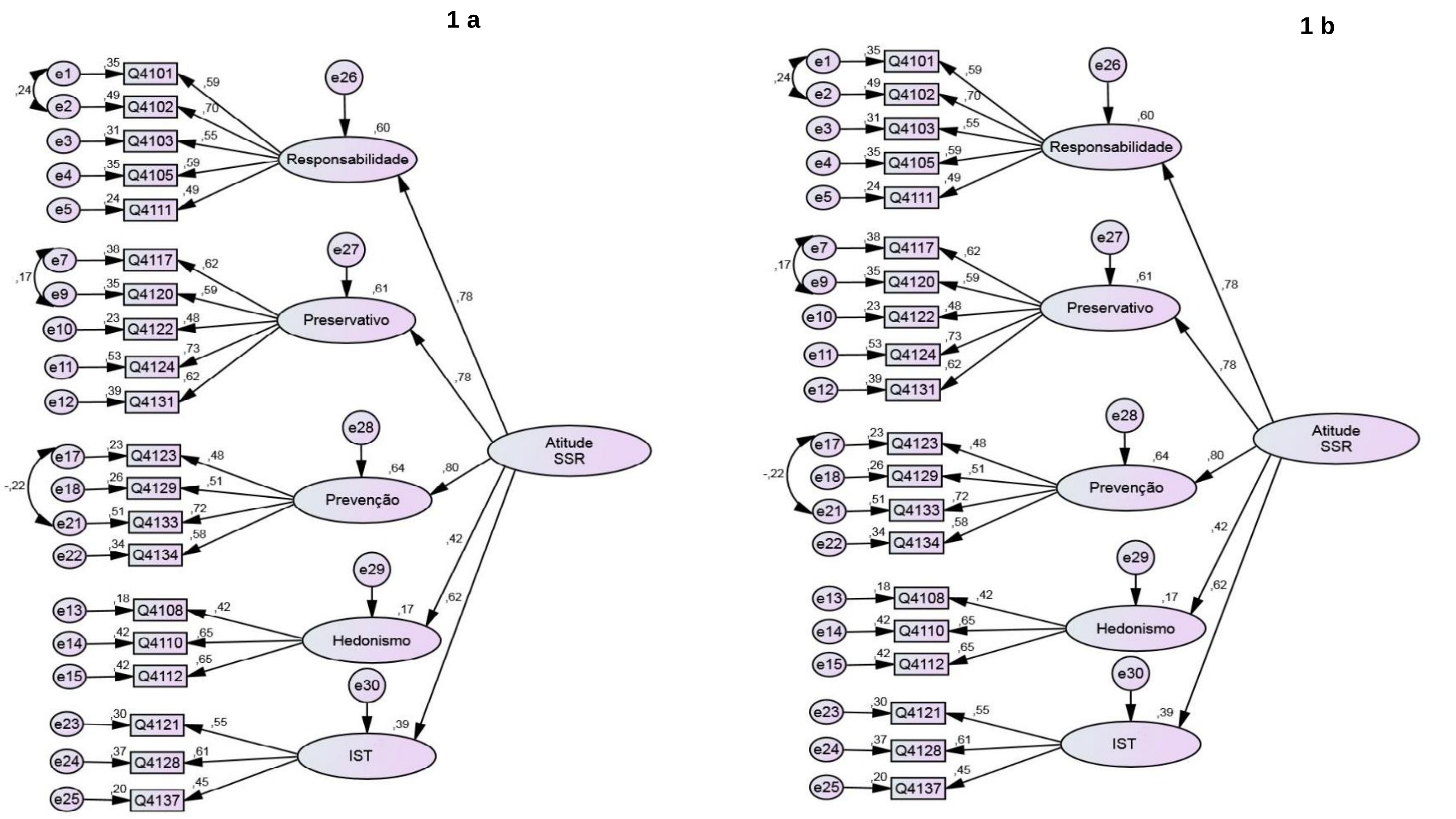

Figura 1. Pesos fatoriais, fiabilidade individual e correlações entre os fatores da EASSR do modelo inicial (1a) e modelo de 2.ª ordem final (1b)

A validação da estrutura fatorial da EASSR foi realizada pela AFC, utilizando a segunda metade aleatória da amostra. Dos 25 itens iniciais, cinco foram excluídos por não cumprirem os critérios de análise recomendados, ficando a solução fatorial final composta por 20 itens organizados nos cinco factores já definidos na AFE. Os índices de qualidade de ajustamento do modelo aos dados, na sua maioria, revelaram-se bons, contudo, não foi possível confirmar a validade de constructo, uma vez que a FC apresenta índices adequados apenas para dois dos fatores da EASSR ($F1_{Responsabilidade}$, $F2_{Preservativo}$). A validade convergente e discriminante também não foram confirmadas. No entanto, as correlações moderadas a elevadas entre o total da escala e os respetivos fatores foram positivas e estatisticamente significativas, o que suporta a conclusão de que os fatores avaliam o mesmo constructo.

Em termos globais, as atitudes de SSR são positivas (independentemente dos componentes avaliados), o que confirma os dados existentes na literatura, no sentido em que os jovens adultos apresentam atitudes positivas face às questões relacionadas com a sexualidade (Abreu, 2010; Antunes, 2007; Matos et al., 2011). Na análise comparativa dos géneros observaram-se diferenças significativas, com as raparigas a revelar uma atitude SSR mais adequada que os rapazes. Esta tendência mantém-se para todas as subescalas, com exceção da atitude hedónica, em que as pontuações médias são superior nos rapazes, resultados que são também concordantes com os de vários estudos, onde os rapazes revelam uma maior valorização da componente erótica – hedonista da sexualidade (Cunha-Oliveira et al., 2013; Janeiro et al., 2013). A atitude de responsabilidade e a atitude face ao preservativo são as dimensões que revelam valores mais elevados em ambos os géneros e a subescala relacionada com a atitude hedónica e atitude face às IST a que apresenta valores mais baixos. O estudo realizado por Matos e colaboradores (2011), também revelou que, de uma forma geral, as raparigas apresentam uma atitude mais positiva em relação aos contracetivos e ao preservativo, e os rapazes uma atitude mais positiva face à sexualidade com maior aceitação do risco sexual.

As limitações do estudo estão relacionadas com o facto de a técnica de amostragem ser não probabilística de conveniência, limitando a generalização dos resultados. Procuramos minimizar esta limitação realizando a aleatorização das turmas e, posteriormente, a aleatorização da amostra para efeitos de análise estatística. Na amostra existe um claro predomínio de participantes do sexo feminino, pelo que futuras investigações adicionais devem usar amostras mais diversificadas. O facto das medidas de intenção comportamental serem auto-referidas, pode não representar uma medida precisa do comportamento real e implica que se considerem aspetos como a desejabilidade social ou o estilo de reposta. Importa salientar que a EASSSR, na forma como foi construída, aplicada e validada,

destina-se a jovens adultos com formação superior, representando uma amostra homogénea, o que pode configurar-se também como uma limitação do estudo.

Conclusões

A análise inicial da EASSR, revelou na AFE características psicométricas bastante promissoras, com bons níveis de fiabilidade (α=0,820), no entanto, a AFC aponta para algumas fragilidades na confirmação da estrutura fatorial, uma vez que foi confirmada a validade de constructo apenas para dois fatores (atitude responsabilidade e atitude face ao preservativo). A validade convergente e discriminante também não foram confirmadas. Tendo em linha de conta que o objetivo do presente estudo era a construção e validação da EASSR para estudantes do ensino superior, os resultados sustentam a necessidade de mais estudos, testando a adequação da estrutura fatorial e a validade de constructo numa amostra independente daquela onde o modelo foi ajustado.

Referências bibliográficas

Abreu, J. F. (2010). *O conhecimento e a atitude face à saúde sexual e reprodutiva. Um estudo correlacional em estudantes universitários.* Dissertação de mestrado não publicada, Faculdade de Ciências da Universidade de Lisboa.

Ajzen, I., & Fishbein, M. (2005). The influence of attitudes on behavior. In D. Ibarracín, B. T. Johnson & M. P. Zanna (Eds.), *The handbook of attitudes* (pp. 173-221). Mahwah, NJ: Erlbaum.

Albarracín, D., Johnson, B. T., Fishbein, M., & Muellerleile, P. A. (2001). Theories of reasoned action and planned behavior as models of condom use: A meta-analysis. *Psychological Bulletin*, *127*(1), 142-161. doi:10.1037/0033-2909.127.1.142

Alferes, V. R. (1999). *Escala de atitudes sexuais*. In M. R. Simões, M.M. Gonçalves & L. Almeida (Eds.), *Testes e provas psicológicas em Portugal* (Vol. 2, pp. 131-148). Braga: SHO/APPORT.

Alvarez, M. J., & Garcia-Marques, L. (2008). Condom inclusion in cognitive representations of sexual encounters. *Journal of Sexual Research, 45*(4), 358-370. doi:10.1080/00224490802398415.

Antunes, M. T. (2007). *Atitudes e comportamentos sexuais de estudantes do ensino superior*. Coimbra: Formasau.

Asare, M. (2015). Using the theory of planned behavior to determine the condom use behavior among college students. *American Journal of Health Studies, 30*(1), 43-50. Retirado de https://www.ncbi.nlm.nih.gov/pmc/articles/PMC4621079/

Associação para o Planeamento da Família. (2010). *Direitos e saúde sexual e reprodutiva de jovens na Europa: Um guia para o desenvolvimento de políticas sobre direitos e saúde sexual e reprodutiva de jovens na Europa*. Lisboa: Federação Internacional de Planeamento Familiar.

Bermúdez, M. P., Teva, I., Ramiro, M. T., Uribe-Rodríguez, A. F., Carlos, J., & Buela-Casal, G. (2012). Knowledge, misconceptions, self-efficacy and attitudes regarding HIV: Cross-sultural assessmeent and analysis in adolescents. *International Journal of Clinical and Health Psychology, 12*, 235-249.

Breckler, S. J., & Wiggins, E. C. (1989). Affect versus evaluation in the structure of attitudes. *Journal of Experimental Social Psychology, 25*(3), 253-271.

Centers for Disease Control and Prevention. (2009a). Sexual and reproductive health of persons aged 10-24 years - United States, 2002-2007. *Morbidity and Mortality Weekly Report, 58* (SS-6), 1-60.

Centers for Diseases Control and Prevention. (2009b). *Sexually transmitted disease surveillance, 2008.* Atlanta, GA: U.S. Department of Health and Human Services. Retirado de http://www.cdc.gov/std/stats08/surv2008

Cunha-Oliveira, A., Cunha-Oliveira, J., Pita, J. R., & Massano-Cardoso, S. (2013). A aquisição do preservativo e o seu (não) uso pelos estudantes universitários. *Revista Referência*, *11* (Série 2), 7-22.

Diéguez Ruibal, J. L., López Castedo, A., Sueiro Domínguez, E., & López Sánchez, F. (2005). Propiedades psicométricas de la escala de actitudes hacia la sexualidad (ATSS) ampliada. *Cuadernos de Medicina psicosomatica y Psiquiatria de enlace (74),* 46-56.

Direção-Geral da Saúde. (2008). *Saúde reprodutiva. Planeamento familiar. Programa nacional de saúde reprodutiva*. Lisboa: Autor.

Eagly, A. H., & Chaiken, S. (1993). *The psychology of attitudes*. Orlando, FL: Harcourt Brace Jovanovich.

Fisheben, M., & Ajzen, I. (2010). *Predicting and changing behavior: The reasoned action aproched*. New York: Taylor & Francis Group.

Ganczak, M., Barss, P., Alfaresi, F., Almazrouei, S., Muraddad, A., & Al-Maskari, F., (2007). Break the silence: HIV/AIDS knowledge, attitudes, and educational needs among Arab university students in United Arab Emirates. *Journal of Adolescent Health, 40*, 572. e1-572.e8. doi: 10.1016/j.adohealth.2007.01.011.

Hendrick, S., & Hendrick, C. (1987). Multidimensionality of Sexual Attitudes. *The Journal of Sex Research, 23*, 502-526.

Janeiro, J.V., Oliveira, I.S., Rodrigues, I.S., Macieiras, M.J., & Rocha, G. M. (2013). As atitudes sexuais, contraceptivas, o lócus de controle da saúde e a autoestima em estudantes do ensino superior. *Revista Brasileira de Promoção da Saúde* 26(4), 505 512. doi:10.5020/18061230.2013.p505

Juhasz, A. M., Kaufman, B., & Meyer, H. (1986). Adolescent attitudes and beliefs about sexual behavior. *Child and Adolescent Social Work, 3*, 177-193. doi:10.1007/BF00779246

Kang, H. E., & Moneyham, L. (2007). Use of emergency contraceptive pills and condoms by college students: A survey. *International Journal of Nursing Studies, 45* (5), 775 -783. doi:10.1016/j.ijnurstu.2007.01.008

Kann, L., McManus,T., Harris, W., Shanklin, S.L., Flint, K., Hawkins, J., … Zaza, S. (2016). Youth Risk Behavior Surveillance - United States, 2015. *Morbidity and Mortality Weekly Report, 65*(6), 1-174.

Khan, M. R., Berger, A. T., Wells, B. E., & Cleland, C. M. (2012). Longitudinal associations between adolescent alcohol use and adulthood sexual risk behavior and sexually transmitted infection in the United States: Assessment of differences by race. *American Journal of Public Health, 102(*5), 867-876. doi: 10.2105/AJPH.2011.300373

Kirby, D., Coyle, K., Alton, F., Rolleri, L., & Robin, L., (2011). *Reducing adolescent sexual risk. Theoretical guide for developing and adapting curriculum-based programs.* California: ETR Associates.

López, F. (2009). *La educación sexual*. Madrid: Biblioteca Nueva.

López, F., & Fuertes, A. (2001). *Para compreender a sexualidade.* Lisboa: Associação para o Planeamento da Família.

Marôco J. (2014). *Análise estatística com o SPSS Statistics*. Pero Pinheiro: Report Number.

Marôco, J. (2011). Análise de equações estruturais: Fundamentos teóricos, software & aplicações. Pêro Pinheiro: Report Number.

Matos, M. G. Reis, M. Ramiro, L., & Equipa Aventura Social. (2011). *Saúde sexual e reprodutiva dos estudantes do ensino superior. Relatório do estudo: Dados nacionais 2010*. Lisboa: Aventura Social.

Nelas, P. (2010). Educação sexual em contexto escolar. Tese de doutoramento não publicada, Universidade de Aveiro.

Nemèiæ, N., Novak, S., Mariæ, L., Novosel, I., Kronja, O., Hren, D., Marušiæ, A., & Marušiæ, M. (2005). Development and validation of questionnaire measuring attitudes towards sexual health among university students. *Croat Medicine*, *46* (1), 52-57.

Reis, M., & Matos, M. G. (2007). Conhecimentos e atitudes face ao uso de métodos contraceptivos e à prevenção das ISTs em jovens. *Revista Lusófona de Ciências e Tecnologias da Saúde, 4*(1), 23-35. Retirado e http://recil.grupolusofona.pt/bitstream/handle/10437/2029/679-2436-1-PB.pdf?sequence=1

Reis, M., Ramiro, L., Matos, M. G., & Diniz, J. A. (2013). Determinants influencing male condom use among university students in Portugal. *International Journal of Sexual Health, 25*(2), 115-127. doi: 10.1080/19317611.2012.728554

Straub, R. O. (2005). *Psicologia da saúde*. Porto Alegre: Artmed.

Tabachnick, B. G., & Fidell, L. S. (2007). *Using multivariate statistics*. Boston: Pearson/Allyn & Bacon.

World Health Organization. (2011). *The sexual and reproductive health of younger adolescents: Research issues in developing countries*. Geneva: Switzerland. Retirado de http://apps.who.int/iris/bitstream/10665/44590/1/9789241501552 _eng.pdf

Worthington, R. W., & Whittaker, T. A. (2006). Scale development research: A content analysis and recommendations for best practices. *Counseling Psychologist, 34*(6), 806-838. doi:10.1177/0011000006288127

CAPÍTULO IV

ESTUDOS EMPÍRICOS EM SAÚDE SEXUAL E REPRODUTIVA

.

CAPÍTULO 4

ESTUDOS EMPÍRICOS EM SAÚDE SEXUAL E REPRODUTIVA

4.1. Artigo 1 - Knowledge of and attitudes on sexual and reproductive health among college students

Santos, M.J., Ferreira, E., & Ferreira, M. (2016).

Atención Primaria, 48 (Esp Cong 1), 188-194.

KNOWLEDGE OF AND ATTITUDES TOWARD SEXUAL AND REPRODUCTIVE HEALTH AMONG COLLEGE STUDENTS

Maria José Santos[1]; Elisabete Ferreira[2]; Manuela Ferreira[3]

[1] Escola Superior de Enfermagem de Vila Real, Universidade de Trás-os-Montes e Alto Douro, Vila Real, Portugal. mjsantos@utad.pt

[2] Faculdade de Psicologia e Ciências da Educação, Universidade do Porto, Porto, Portugal.

[3] Escola Superior de Saúde de Viseu, Instituto Politécnico de Viseu, Viseu, Portugal

Abstract

Introduction: Knowledge provides the foundation for values, attitudes and behavior. Knowledge about sexual and reproductive health (SRH) and positive attitudes are essential for implementing protective behaviors. Objectives: The aim of this study was to evaluate SRH knowledge and attitudes in college students and their association with sexual and reproductive behaviors.

Methods: A cross-sectional study was conducted in a sample of 1946 college students. The data were collected using a self-report questionnaire on the sociodemographics characteristics of the sample, an inventory on SRH knowledge and an attitude scale, and were analyzed with descriptive and inferential statistics (ANOVA and Pearson's correlation).

Results: The sample was 64% female and 36% male, with a mean age of 21 years. The majority were sexually active and used contraception. The SRH knowledge was moderate (22.27±5.79; maximum score=44), while the average SRH attitude score was more favorable (118.29±13.92; maximum score=140). Female and younger students studying life and health sciences had higher ($p<0.05$) SRH knowledge and attitude scores. The consistent use of condom and health care surveillance were highly dependent on the students' SRH knowledge and attitudes. Engagement in sexual risk behaviors was associated with lower scores for these variables.

Conclusions: Strategies to increase SRH knowledge and attitudes are important tools for improving protective behaviors, especially with respect to contraception, health care surveillance and exposure to sexual risk. Older males studying topics other than life sciences should be a priority target for interventions due to their higher sexual risk.

Keywords: Attitude, knowledge, sexual and reproductive behavior, college students.

Introduction

Sexual and reproductive health (SRH) is an essential part of public health and can considerably influence general well-being and quality of life (Hartney et al., 2015). The promotion of SRH is of particular importance in college students because this population is at high risk for sexually transmitted infections (STI) and unintended pregnancies (Kann et al., 2014; Wilton, Palmer & Maramba, 2014). International surveillance reports demonstrate trends of a high prevalence of STIs among young adults, namely, HIV, Chlamydia, trichomoniasis and HPV (European Centre for Disease Prevention and Control / WHO Regional Office for Europe, 2014; Kann et al., 2014). Portugal is among the countries with the highest prevalence of HIV, with people aged 20 to 24 years accounting for 11.2% of reported cases (European Centre for Disease Prevention and Control / WHO Regional Office for Europe, 2014), and the prevalence of HPV infection is 28.8% for females aged 18 to 24 years (Pista et al., 2011). In Portugal, in 2015, 2296 teenagers (12-19 years) were mothers (Instituto Nacional de Estatística, 2016), and higher rates of abortion were observed in young adult women (22.4% among women 20 to 24 years old and 20.8% among women 25 to 29 years old) (Direção-Geral da Saúde, 2015). In the 2014/15 academic year, 44 659 students were enrolled in universities and colleges in Portugal (Direção-Geral do Ensino Superior, 2016). A significant percentage of students engage in risky sexual behaviors such as using drugs or alcohol during sexual activity (Burnett et al., 2014; Ghandour, Mouhanna, Yasmine & El Kak, 2014; Wicki, Kuntsche & Gmel, 2010), having sex with multiple and casual partners (Garcia, Reiber, Massey & Merriwether, 2012; Grello, Welsh & Harper, 2006), and inconsistently using condoms (Reis, Ramiro, Matos & Diniz, 2013; Wilton et al., 2014).

Understanding the factors (e.g., knowledge, attitudes and behavioral skills) that influence the SRH behaviors of college students is important because they have a significant impact on sexual decision making, and behavior and educational programs can significantly improve these factors (Kirby, Coyle, Alton, Rolleri & Robin, 2011; Reis et al., 2013). Knowledge provides a foundation for human action; this is a central concept in many psychological theories and is commonly used as the theoretical basis for effective sex and STD/HIV education programs. Knowledge may also transform behavior indirectly by affecting values, attitudes, perceptions of norms and even perceptions of self-efficacy. However, even though knowledge may provide a foundation, greater knowledge may not necessarily assure responsible behavior, because knowledge alone is not sufficient (Kirby et al., 2011). According to several models that aim to explain changes in health behavior, attitudes are among the most important theoretical constructs affecting behavior (Albarracín, Johnson & Zanna, 2014; Pickens, 2011). The attitude toward a behavior is the extent to which a person has a favorable or unfavorable appraisal of that behavior (Asare, 2015). Attitudes are formed

through life experiences and are learned behaviors from others. In contemporary language, attitudes are defined as complex, multidimensional constructs comprising cognitive, affective, and behavior components. The three components reinforce each other to form a joint structure that tends to remain stable (Albarracín et al., 2014). The relationship between knowledge, attitudes and behavior is complex. However, several studies suggested that information and positive attitudes towards condom use and other forms of contraception has a strong association with consistent condom use (Albarracín, Johnson, Fishbein & Muellerleile, 2001; Asare, 2015), and with condom negotiation skills (Gakumo, Moneyham, Enah & Childs, 2002. College students who are well informed and have more positive attitudes and skills tend not to accept sexual risks (Gakumo, 2001; Reis et al., 2013).

Understanding college students' SRH knowledge and attitudes is important for planning effective educational programs in universities. The aim of this study was to evaluate SRH knowledge and attitudes in college students and their association with sexual and reproductive behaviors.

Materials and methods

Design and sampling

A cross-sectional study was conducted in a sample of 1946 college students (64% female and 36% male) with a mean age of 21 years attending a university in Portugal in the 2012/2013 academic year. Students were recruited by direct solicitation in the classroom, and participation was voluntary. Classes were selected for their representation of the scientific areas of study and for the different years of study of the students. The data were collected with an anonymous self-administered survey.

Measures

We collected information on demographic characteristics: gender, age (≤19, 20-24, and ≥25 years), nationality, place of childhood (urban, rural), area of studies, parental education level (9 years, 10 to 12 years, and university) and monthly family income (<1000 US$; 1000-2000 US$; and >2000 US$). Information on previous sexual behavior was collected: had sex in the last twelve months, use of sexual health care services, use of a contraceptive method, having sexual intercourse consistently using a condom in the last 12 months, sources of information on SRH, having sex while under the influence of alcohol or drugs and having sex with casual partners, having had an HIV test and having had an STI.

Knowledge about SRH was measured using an inventory-based self-report questionnaire made up of 44 items developed by the authors for the present study. The SRH knowledge

inventory was structured in four dimensions based on knowledge of the students' social environments and on a literature review of SRH: (1) information related to sexual physiology and conception, (2) methods of contraception, (3) STIs and STI prevention, and (4) SRH surveillance. Items were rated using three response options: true, false and don't know. Correct answers were coded as one, and incorrect or uncertain responses were coded as zero. The total score ranged from 0 to 44 points. The reliability of the knowledge inventory was assessed using Kuder-Richardson's formula 20 coefficient (KR20), an analogue to Cronbach's alpha and is used as a measure of internal consistency in studies with dichotomous scales (Hair, Black, Babin & Anderson, 2014). The result was KR20=0.78, confirming the adequate internal consistency of the instrument.

Attitudes towards SRH were measured using a scale composed of 20 items designed specifically for the study. The SRH attitude scale had seven response options (1 - *strongly disagree* to 7 - *strongly agree*), which assessed five theoretical dimensions of SRH attitudes (responsible sexuality, preventive attitudes, attitudes towards STIs, attitudes towards condoms, and hedonism). The results vary between 20 and 140 points, with the highest score indicating more positive SRH attitudes. The scale revealed good internal consistency (Cronbach's alpha =0.820).

Data analysis

The data were analyzed using SPSS software (version 22.0, 2012; SPSS, Inc., Chicago, IL, USA). The associations between the SRH knowledge scores and SRH attitudes and sociodemographic and academic characteristics and sexual behaviors were analyzed using one-way ANOVA. The relationships between knowledge and SRH attitudes were established using Pearson's correlations. Missing cases were omitted from the analysis. Statistical significance was set at a p value of less than 0.05.

Ethical considerations: The study was authorized by the Comissão de Proteção de Dados (National Data Protection Committee) (Autorização Nº 7409/2013) and was sanctioned by the Ethics Committee (Opinião Nº 2/2012) of the University where the study was conducted.

Results

The average age of the students surveyed was 20.74±2.32 years, and 64% were female. Approximately two-thirds (63.4%) were students in life sciences courses. Their childhood residence was either rural (60%) or urban (40%). Their household income was generally low (57%<1000 US$/month). Their parents' levels of education were also low: 54.5% of the mothers and 61.6% of the fathers only had nine years of education.

The majority of students (76.9%) was sexually active and report having used contraception (96.7%). The most commonly used contraceptive methods were hormonal contraception (43.7%) and condoms (21.8%). Only 39.5% of the participants reported using condoms consistently. The rate of students who have previously used an SRH service was 22.2%, and women used these services more than men (♀=30.4% vs ♂=7.6%, $p<0.0001$). Only 14.5% of students had been tested for HIV. STIs were reported by only 1.3% of the students. The most common sources of SRH information were friends (46.1%), the internet (45.3%), and teachers (42.7%). Nursing professionals have a secondary role in transmitting information (24.2%). A considerable proportion of students reported engagement in risk behaviors, such as having intercourse under the influence of alcohol (33.0%) or drugs (9.7%) and having intercourse with occasional partners (32.0%).

The mean total SRH knowledge score was 22.76±5.79 (the score ranges from 0 to 44) (Table 1). The contraception knowledge score was slightly higher than the middle of the scale (8.42±2.44, maximum of the score was 16), and the STI knowledge score was slightly lower than the middle of the scale (6.05±2.73, maximum score 16). The worst knowledge scores were observed for SRH surveillance. This dimension of knowledge was composed of four questions related to HPV vaccination and vaginal cytology. Even considering that these aspects are more related to female health surveillance, the mean score was below the middle of the scale.

Female students had higher levels of knowledge ($p<0.001$). Significant differences were also observed in knowledge among students belonging to different age groups. Younger students had higher scores ($p<0.05$) for the general aspects of sexual physiology, contraception and, consequently, had higher total SRH knowledge scores. The main difference observed among students of different ages was that older students (≥25 years) were less informed than students aged 20-24 years or ≤19 years. The scientific area of study also influenced the knowledge of these students. Those majoring in life and health sciences presented with higher ($p<0.001$) knowledge scores than those studying other areas.

Generally, students held positive attitudes (Table 1) clearly above the middle of the scale (118.28±13.92). As observed for the knowledge scores, all dimensions had a similar trend. The effects of gender, age and area of study observed for the knowledge was generally also observed for the attitude. Only the attitude towards hedonism had a different pattern, showing that gender, older age and studying non-life sciences had a more significant influence on hedonic perspectives of sex.

Table 1.
The influence of demographics and academic factors on sexual and reproductive health knowledge and attitudes. The results are presented as the mean (standard deviation)

	**SRH Knowledge**					**SRH Attitude**					
**Variables**	**General (0 – 8)**[Ψ]	**Contraception (0 – 16)**[Ψ]	**STI´s**[1] **(0 – 16)**[Ψ]	**Surveillance (0 – 4)**[Ψ]	**Total (0 – 44)**[Ψ]	**Re.**[2] **sexuality (5 – 35)**[Ψ]	**Preventive (4 – 28)**[Ψ]	**STI`s (3 – 21)**[Ψ]	**Condom (5 – 35)**[Ψ]	**Hedonism (3 – 21)**[Ψ]	**Total (20 – 140)**[Ψ]
**All students**	3.86±1.67	8.42±2.44	6.05±2.73	1.43±0.95	22.76±5.79	31.17±4.31	22.97±4.09	15.94±3.50	30.11±5.34	17.62±3.17	118.29±13.92
**Gender**											
Male (n=690)	3.21±1.65	7.37±2.62	8.50±2.95	1.03±0.90	20.11±6.07	29.09±5.13	21.66±4.22	15.25±3.52	28.00±6.18	17.54±3.07	111.85±15.87
Female (n=1246)	4.22±1.56	9.01±2.12	9.36±2.55	1.65±0.91	24.24±5.05	32.34±3.24	23.70±3.82	16.33±3.44	31.29±4.38	17.66±3.14	121.89±11.19
	p < 0.001	p < 0.001	p < 0.001	p < 0.001	p < 0.001	p < 0.001	p < 0.001	p < 0.001	p < 0.001	p = 0.417	p < 0.001
**Age**											
≤ 19 (n=619)	3.94±1.59b[4]	8.63±2.39b	9.10±2.72	1.50±0.94	23.17±5.60b	31.36±4.36b	23.03±4.13	15.98±3.34	30.74±5.19b	17.31±3.15a	118.99±13.95b
20-24 (n=1187)	3.86±1.69b	8.40±2.43b	9.05±2.70	1.41±0.95	22.72±5.83b	31.21±4.21b	22.99±4.02	15.97±3.54	29.94±5.31b	17.74±3.11ab	118.27±13.67b
≥ 25 (n=139)	3.53±1.70a	7.69±2.59a	8.85±3.05	1.32±0.95	21.39±6.08a	30.12±4.80a	22.49±4.42	15.56±3.85	28.81±5.75a	17.90±2.91b	115.41±15.43a
	p = 0.038	p < 0.001	p = 0.624	p = 0.061	p = 0.005	p = 0.008	p = 0.353	p = 0.412	p < 0.001	p = 0.011	p = 0.023
**Area of studies**											
Life & H[3] (n=1233)	4.10±1.63	8.80±2.30	9.60±2.52	1.52±0.96	24.02±5.33	31.45±3.93	23.38±3.93	16.26±3.49	30.69±4.92	16.86±2.94	120.19±12.44
Other (n=713)	3.44±1.65	7.75±2.53	8.10±2.82	1.27±0.92	20.57±5.89	30.69±4.86	22.25±4.25	15.40±3.46	29.10±5.86	17.18±3.35	114.97±15.63
	p < 0.001	p < 0.001	p < 0.001	p < 0.001	p < 0.001	p < 0.001	p < 0.001	p < 0.001	p < 0.001	p < 0.001	p < 0.001

SRH - Sexual and Reproductive Health; **Ψ -** Minimum and maximum scores of the scale. [1] **STI´s** - Sexually Transmitted Infections; [2] **Re** – Responsible; [3] **H** - Health; [4] **abc** - Means in the same column for variable age followed by different letters are different (p<0.05).

The relationship between the self-reported sexual behaviors and the global SRH knowledge and attitude scores of the students was established comparing the mean scores of the students and their sexual behaviors (Table 2). Having an active sexual life was found to be independent ($p>0.05$) of SRH knowledge or attitudes. The utilization of the health care services and the use of contraception were dependent on the students' SRH knowledge and attitude scores. Having always used a condom when having sex in the last twelve months had no relationship ($p>0.05$) with knowledge, but it was associated with higher attitude scores. The three sexual risk behaviors self-reported by the students were also highly dependent ($p<0.001$) on their SRH knowledge and attitudes, with students with lower knowledge and attitude scores reporting more risky behaviors. Having done the HIV test was dependent on the knowledge ($p=0.031$) but not the attitude ($p=0.510$).

The relationship between the knowledge and attitude scores was established by Pearson's correlations for the entire sample and separately for women and men (data not presented). The correlation between the global SRH knowledge and attitude scores for all students was 0.369 ($p<0.001$), indicating that the theoretically expected association between attitude and knowledge exists. The relationship between the contraception and STI knowledge and attitude dimensions was interesting. When the correlations are established separately for women and for men, for all possible associations, the value of the correlation coefficient is always higher among males (contraception knowledge, and SRH attitude, $r=0.306$ $p<0.001$; IST´s knowledge and SRH attitude, $r=0.395$ ($p<0.001$) than among females (contraception knowledge and SRH attitude, $r=0.222$, $p<0.001$; IST´s knowledge and SRH attitude, $r=0.213$ ($p<0.001$) indicating that men and women use knowledge differently in the development of their attitudes.

Discussion

College students have moderate knowledge levels and positive attitudes towards SRH, as consistently reported in the literature (Reis et al., 2013; Sohbet & Geçici, 2014).

The higher knowledge and more favorable attitudes of women have been systematically found in surveys about psycho-cognitive aspects of sexuality among young adults (Reis et al., 2013; Sohbet & Geçici, 2014; Yazic, Dolgun, Zengin & Bayram, 2012). This trend might be justified mainly by higher investment of healthcare services has in the needs of women because, due to multiple biological and social determinants, they are more exposed and vulnerable to sexual and reproductive risks (WHO, 2009). Better knowledge among women might also be attributed to the sources of information they prefer, which are more formal (health professionals and teachers) than the sources used by men (friends and the internet).

Table 2.
The relationship between sexual and reproductive health behaviors and knowledge and attitudes. The results are presented as the mean (standard deviation)

	SRH Knowledge		SRH Attitude	
	Mean±SD	p	Mean±SD	p
**Active sexual life**				
Yes (n=1496)	22.77±5.78	0.930	118.20±14.18	0.665
No (n=449)	22.74±5.86		118.53±13.04	
**Contraception use**				
Yes (n=1447)	22.86±5.76	0.003	118.43±14.06	0.010
No (n=56)	20.44±6.02		113.48±15.26	
**Consistent condom use**				
Yes (n=591)	22.69±5.81	0.625	119.68±14.51	0.001
No (n=906)	22.84±5.76		117.29±13.85	
**Sex with alcohol**				
No (n=1011)	23.06±5.77	0.001	120.18±13.25	<0.001
Yes (n=511)	22.00±5.82		114.14±15.01	
**Sex with drugs**				
Yes (n=147)	20.41±5.98	<0.001	110.99±15.27	<0.001
No (n=1361)	22.96±5.74		118.97±13.80	
**Sex with casual partners**				
Yes (n=482)	21.81±5.68	<0.001	113.77±15.21	<0.001
No (n=1023)	23.14±5.78		120.27±13.13	
**SRH health care use**				
Yes (n=432)	24.78±4.95	<0.001	122.18±12.24	<0.001
No (n=1449)	22.18±5.88		117.17±14.17	
**VIH test**				
Yes (n=283)	23.37±5.89	<0.031	117.85±14.83	<0.501
No (n=1663)	22.66±5.76		118.35±13.76	

SRH - Sexual and Reproductive Health; SD- standard deviation.

In the present study, older students had lower levels of knowledge and less favorable attitudes than younger students. This result might be attributed to the more hedonic behaviors of older students or to education (Gómez-Camargo et al., 2014; Reis et al., 2011). Younger students attended high school more recently, at a time when sex education was a mandatory course. This curriculum was introduced in Portugal after 2009, resulting in younger students having a different background of sexual knowledge in relation to older college students. The importance of formal sexual education in high school has been studied in literature reviews (Gómez-Camargo et al., 2014; Kirby et al., 2011), which demonstrated that formal sexual education could effectively modify sexual risk behavior in college students (Reis et al., 2011).

The life and health sciences area of study had a clear influence on the students' SRH knowledge and attitude scores. This finding was expected because sexuality issues are a

part of the syllabus of several bachelor degree programs (nursing, veterinary, and biology, among others).

It is accepted by several theoretical frameworks that knowledge is the foundation of individuals' attitudes and behaviors (Kirby et al., 2011). Thus, modifying knowledge is expected to affect attitudes and, subsequently, behaviors (Albarracín et al., 2014; Kirby et al., 2011). Considering this theoretical framework, the results of the present work are concerning because the students had only moderate levels of knowledge. As an example of the gaps in knowledge that should be addressed with the students, there are a considerable number of students that do not know the difference between being HIV positive and having AIDS or do not understand that STIs are more likely to be transmitted through unprotected anal sex than vaginal sex. In the present work, there is an apparent inconsistency between knowledge and attitudes: students with moderate levels of knowledge have very positive attitudes. This apparent discrepancy might be due to the more formal format of the knowledge inventory, which captured very detailed knowledge levels among the surveyed students. On the other hand, the favorable attitudes might have been formed by the foundation of knowledge, because attitude is formed over a lifetime through an individual's socialization process. Attitude formation is a result of learning, modeling others, and our experiences with people and situations (Albarracín et al., 2014; Pickens, 2011). In that sense, one of the basic behaviors that should be clearly recommended among young adults, consistent condom use, was not associated with knowledge, but it was associated with attitudes, suggesting that students form adequate attitudes independent of their knowledge.

The results suggest that knowledge is more important among males, who may have a more cognitive approach to attitude formation, while females' attitude formation is more determined by emotive aspects, with previous emotional experiences playing an important role.

Limitations

The survey results may not be generalizable because the participants are from one university. However, the students came from various regions of the country, and we randomly selected classrooms to minimize this limitation. The behavioral intention measures were self-reported and may not accurately measure actual behaviors. Social desirability may have been an issue because of the sensitive nature of the survey items.

Conclusions

In the Portuguese academic context, students have good attitudes towards sexual and reproductive issues, albeit their knowledge was only moderate. Both knowledge and attitudes were associated with the sexual behaviors of contraception use and the use of SRH

surveillance services. Students reporting frequent engagement in risky sexual behaviors had lower knowledge and attitude scores. The association between knowledge and attitudes was positive. That relationship was stronger in men than in women, indicating that knowledge is more important for attitude formation in men than in women. This finding is of greatest importance, as men are also more exposed to risks and require particular attention in the development of nursing interventions to improve the sexual health of college students.

References

Albarracín, D., Johnson, B. T., & Zanna, M. P. (2014). *The handbooh of attitudes*. New York: Psycology Press.

Albarracín, D., Johnson, B. T., Fishbein, M., & Muellerleile, P. A. (2001). Theories of reasoned action and planned behavior as models of condom use: A meta-analysis. *Psychological Bulletin, 127*(1), 142-161. doi:10.1037//0033-2909.127.1.142

Asare, M. (2015). Using the theory of planned behavior to determine the condom use behavior among college students. *American Journal of Health Studies, 30*(1), 43-50. Retrieved from https://www.ncbi.nlm.nih.gov/pmc/articles/PMC4621079/

Burnett, A. J., Sabato, T. M., Walter, K. O., Kerr, D. L., Wagner, L., & Smith, A. (2014). The Influence of attributional style on substance use and risky sexual behaviour among college students. *College Student Journal, 48*(21), 325-336.

Direção-Geral da Saúde. (2015). *Relatório dos registos de interrupção da gravidez: Dados 2014*. Lisboa: Divisão de Saúde Sexual, Reprodutiva, Infantil e Juvenil.

Direção-Geral do Ensino Superior. (2016). *Alunos matriculados no ensino superior*. Retrieved from: http://www.dges.mctes.pt/estatisticasacesso/2015/

European Centre for Disease Prevention and Control/WHO Regional Office for Europe. (2014). *HIV/AIDS surveillance in europe 2013*. Stockholm: ECDC. Retrieved from http://ecdc.europa.eu/en/publications/Publications/hiv-aids-surveillance-report-Europe-2013.pdf

Gakumo, C. A., Moneyham, L. D., Enah, C. C., & Childs, G. D. (2012). The moderating effect of sexual pressure on young urban women's condom use. *Research Nursing Health, 35*(1), 4-14. doi:10.1002/nur.20465

Garcia, J. R., Reiber, C., Massey, S. G., & Merriwether, A. M. (2012). Sexual hookup culture: A review. *Review of General Psychology, 16*(2), 161-176. doi:10.1037/a0027911.

Ghandour, L., Mouhanna, F., Yasmine, R., & El Kak, F. (2014). Factors associated with alcohol and/or drug use at sexual debut among sexually active university students: Cross-sectional findings from Lebanon. *BMC Public Health, 14*, 671-681. doi:10.1186/1471-2458-14-671

Gómez-Camargo, D. E., Ochoa-Diaz, M. M., Canchila-Barrios, C. A., Ramos-Clason, E. C., Salguedo-Madrid, G. I., & Malambo-García, D. I. (2014). Salud sexual y reproductiva en estudiantes universitarios de una institución de educación superior en Colombia. *Revista de Salud Pública,16*(5). 660-672. Retrieved from http://www.scielosp.org/pdf/rsap/v16n5/ v16n5a02.pdf

Grello, C. M., Welsh, D. P., & Harper, M.S. (2006). No strings attached: The nature of casual sex in college students. *Journal of Sexual Research, 43*(3), 255-267. doi:10.1080/00224490609552324

Hair, J. F., Black, W. C., Babin, J. B., & Anderson, R. E. (2014). *Multivariate data analysis* (7th ed.). Edinburgh: Pearson Educational.

Hartney, T., Westrop, S. J., Anderson, J., Brigstock-Barron, O., Hadley, A., Guthrieet, L., … Connor, N. (2015). *Health promotion for sexual and reproductive health and HIV: Strategic action plan, 2016 to 2019*. London: Public Health England. Retrieved from https://www.gov.uk/government/uploads/system/uploads/attachment_data/file/488090/SRHandHIVStrategicPlan_211215.pdf

Instituto Nacional de Estatística. (2016). *Dados estatísticos: Taxas de natalidade refentes ao ano de 2015*. Retrieved from https://www.ine.pt/xportal/xmain?xpgid=ine_main&xpid=INE&xlang=pt

Kann, L., Kinchen, S., Shanklin, S. L., Flint, K. H., Kawkins, J., Harris, W. A.., … Centers for Disease Control and Prevention. (2014). Youth risk behavior surveillance - United States, 2013. *MMWR, 63*(4), 1-168.

Kirby, D., Coyle, K., Alton, F., Rolleri, L., & Robin, L. (2011). *Reducing adolescent sexual risk. Theoretical guide for developing and adapting curriculum-based programs*. California: ETR Associates.

Pickens, J. (2011). Attitudes and perceptions. In N. Borkowski, *Organizational behavior in health care* (2nd ed., chap. 3, pp. 43-76). Florida: Jones and Bartlett Publishers. Retrieved from http://healthadmin.jbpub.com/Borkowski/chapter3.pdf

Pista, A., Oliveira, C. F., Cunha, M. J., Paixão, M. T., Real, O., & CLEOPATRE Portugal Study Group. (2011). Prevalence of human papillomavirus infection in women in Portugal: The CLEOPATRE Portugal study. *International Journal of Gynecological Cancer, 21*(6), 1150-1158. doi:10.1097/IGC.0b013e31821dd3b2

Reis, M., Ramiro, L., Matos, M. G., & Diniz, J. A. (2011). The effects of sex education in promoting sexual and reproductive health in portuguese university students. *Procedia - Social and Behavioral Science, 29*, 477-485. doi:10.1016/j.sbspro.2011.11.266

Reis, M., Ramiro, L., Matos, M. G., & Diniz, J. A. (2013). Determinants influencing male condom use among university students in Portugal. *International Journal of Sexual Health, 25*(2), 115-127.

Sohbet, R., & Geçici, F. (2014). Examining the Level of knowledge on sexuality and reproductive health of students of gaziantep university. *Sexuality & Disability, 32*(1), 75-84. doi: 10.1007/s11195-013-9332-6

Wicki, M., Kuntsche, E., & Gmel, G. (2010). Drinking at european universities? A review of students' alcohol use. *Addictive Behaviors, 35*(11), 913-924. doi:10.1016/j.addbeh.2010.06.015

Wilton, L., Palmer, R. T., & Maramba, L. C. (2014). *Understanding HIV and STIs prevention for college students*. London: Routledge Research in Higher Education.

World Health Organization. (2009). *Women and health. Today´s evidence tomorrow´s agenda*. Geneva: WHO Press.

Yazici, S., Dolgun, G., Zengin, N., & Bayram, G. (2012). The determination of university students' knowledge, attitudes and behaviors on the matter of sexual health. *Sexuality & Disability, 30*(1), 67-75. doi:10.1007/s11195-011-9246-0

What we know about the theme

- Theoretically, there is an association between knowledge, attitudes and behavioral skills concerning SRH.

- College students have low knowledge and positive attitudes concerning SRH, especially with respect to contraceptive methods and STIs/VIH. However this population remains sexual and reproductive risk behaviors.

What we learn from the study

- Knowledge and attitudes were positively associated with sexual and reproductive behaviors, including the use of contraception and health care services, and negatively associated risky sexual behaviors.

- The association between SRH knowledge and attitudes was positive, although this relationship was different in men and women. Attitudes are more strongly dependent on knowledge among men than among women.

CAPÍTULO 4

ESTUDOS EMPÍRICOS EM SAÚDE SEXUAL E REPRODUTIVA

4.2. Artigo 2 - Comportamentos contracetivos de estudantes do ensino superior

Santos, M.J., Ferreira, E., & Ferreira, M.

(A submeter à Revista Portuguesa de Saúde Pública)

COMPORTAMENTOS CONTRACETIVOS DE ESTUDANTES DO ENSINO SUPERIOR

CONTRACEPTIVE BEHAVIOR OF STUDENTS IN COLLEGE STUDENTS

Maria José Santos[1]; Elisabete Ferreira[2]; Manuela Ferreira[3]

[1] Escola Superior de Enfermagem de Vila Real, Universidade de Trás-os-Montes e Alto Douro, Vila Real, Portugal. mjsantos@utad.pt

[2] Faculdade de Psicologia e Ciências da Educação, Universidade do Porto, Porto, Portugal.

[3] Escola Superior de Saúde de Viseu, Instituto Politécnico de Viseu, Viseu, Portugal.

Resumo

Introdução: O acesso universal dos jovens aos serviços de saúde e a métodos contracetivos constitui uma forma privilegiada de diminuir a gravidez indesejada e as infeções sexualmente transmissíveis (IST). A utilização de métodos contracetivos de forma eficaz depende de uma grande variedade de fatores, inerentes à complexidade do comportamento sexual e que devem ser considerados na criação das políticas e programas de promoção da saúde sexual e reprodutiva na juventude.

Objetivos: O presente estudo teve como objetivo caracterizar as práticas contracetivas dos estudantes do ensino superior, atendendo às questões de género e identificar fatores que contribuem para a utilização dos diferentes métodos contracetivos.

Metodologia: Foi realizado um estudo transversal, descritivo-correlacional, numa amostra se 1946 estudantes do ensino superior (63% raparigas e 37% rapazes), com idades compreendidas entre os 18-29 anos (20,74±2,32).

Resultados: A maioria dos estudantes é sexualmente ativa e utiliza contraceção, as raparigas usam preferencialmente os métodos hormonais e os rapazes o preservativo. Dos fatores estudados, o género feminino, as idades mais novas, a religião, o comportamento anterior de usar o preservativo, o conhecimento sobre contraceção, a atitude e autoeficácia para usar o preservativo, estiveram associados a escolhas contracetivas mais seguras.

Conclusões: Investir na educação dos jovens adultos é uma importante estratégia de saúde pública, dado que pode potenciar escolhas mais adequadas e melhorar adesão aos métodos contracetivos, diminuindo o risco de gravidez indesejada e de IST.

Palavras-chave: Contraceção, enfermagem, dupla proteção, estudantes ensino superior, preservativo, saúde reprodutiva.

Abstract

Introduction: Universal access of young people to health services and contraceptive methods is a key way to reduce unwanted pregnancies and sexually transmitted infections (STI). The use of contraceptive methods effectively depends on a wide range of factors, inherent of the complexity of sexual behaviour, and should be considered in the development of policies and programs to promote sexual and reproductive health in youth.

Objectives: This study aimed to characterize the contraceptive practices of college students, considering gender particularities and to identify factors that contribute to the use of different contraceptive methods.

Methodology: A cross-sectional, descriptive-correlational study was carried out in a sample of 1946 higher education students (63% girls and 37% boys), aged 18-29 years (20.74±2.32).

Results: Most students are sexually active and use contraception. Girls preferentially use hormonal methods and boys the condom. Of the factors studied, the female gender, the younger ages, the religion, the previous behavior of using the condom, the knowledge about contraception, the attitude and self-efficacy to use the condom, were associated with safer contraceptive choices.

Conclusions: Investing in the education of young adults is an important public health strategy, as it can potentiate better choices and improve adherence to the contraceptive method, reducing the risk of unwanted pregnancies and STI.

Keywords: Condom, contraception, college students, dual protection, nursing, reproductive health.

Introdução

Os jovens são considerados um grupo de intervenção prioritária no âmbito da saúde sexual e reprodutiva (SSR), centrando-se as estratégias na prevenção das infeções sexualmente transmissíveis (IST) e gravidez indesejada (Kann et al., 2016; Lazarus, Sihvonen-Riemenschneider, Laukamm-Josten, Wong & Liljestrand, 2010; Neto, Bombas, Arriaga, Almeida & Moleiro, 2012). Disponibilizar aconselhamento contracetivo e métodos contracetivos que respondam às necessidades de cada pessoa na sua diversidade e de acordo com as suas características individuais constitui um importante mecanismo de melhoria dos cuidados de SSR prestados (Direção-Geral da Saúde [DGS], 2015). Os profissionais de saúde, e em particular os enfermeiros, desempenham um papel crucial na educação sexual dos jovens, não só no sentido de potenciar a sua capacidade de escolha e

qualidade do uso dos contracetivos, mas também alertando para a importância da proteção adequada, particularmente de métodos de barreira que evitam as IST, da vacinação pré-exposição no caso do vírus do papiloma humano (HPV), e da importância da vigilância regular da SSR (Barros & Neves, 2013; Lopez, Otterness, Chen, Steiner & Gallo, 2013). Atendendo às características dos jovens adultos, idealmente, o método contracetivo deverá ser seguro, eficaz, reversível, pouco dispendioso, discreto e com poucos efeitos colaterais (Barros & Neves, 2013).

A utilização de métodos contracetivos de forma regular e eficaz depende de uma grande panóplia de fatores, que devem ser considerados na criação das políticas e programas de promoção da SSR da juventude. Neste sentido, alguns investigadores enfatizam a importância dos estudos que procuram compreender a influência dos fatores psicossociais e comportamentais implicados nas práticas contracetivas dos jovens adultos, salientando a importância da perceção de (in) vulnerabilidade face às IST (Kirby, Coyle, Alton, Rolleri & Robin, 2011; Wilton, Palmer & Maramba, 2014), as expectativas associadas à utilização de contraceção (dificuldade em obter contracetivos), a (in) capacidade de planear a atividade sexual dado o carácter espontâneo das relações nesta faixa etária (Reis & Matos, 2008), a autoeficácia, a (des) confiança nas capacidades para utilização de métodos contracetivos de forma consistente (Gomes & Nunes, 2011; Hickey & Cleveland, 2013; Kann et al., 2016), a dificuldade em comunicar com o parceiro sexual, com os pais e com os profissionais de saúde sobre contraceção (Associação para o Planeamento da Família [APF], 2010; Gomes & Nunes, 2011) e o tipo de relacionamento mais estável ou ocasional (Chamratrithirong & Kaiser, 2012; O'Sullivan, Udell, Montrose, Antoniello & Hoffman, 2010; Ssewanyana et al., 2015). Acresce que, no contexto universitário, têm vindo a ser identificados outros comportamentos sexuais, que configuram um risco acrescido, designadamente a prática de relações sexuais desprotegidas com parceiros ocasionais e com outras pessoas, para além do parceiro habitual (Kuperberg & Padgett, 2015; Pacheco, 2012; Reis, Ramiro, Matos & Diniz, 2013; Wilton et al., 2014) e a utilização de substâncias psicoativas (álcool e drogas) associadas às relações sexuais, considerado quase normativo no contexto da noite e das festas académicas (Burnett et al., 2014; Pollock, 2016; Rodrigues, Salvador, Lourenço & Santos, 2014; Wicki, Kuntsche & Gmel, 2010). Esses consumos podem levar a uma menor eficácia da contraceção, quer pela sua utilização incorreta, quer pela incapacidade de negociar a sua utilização com a pessoa com está em vias de fazer sexo (Certain, Harahan, Saewyc & Fleming, 2009; Uecker, 2015; Wilton et al., 2014). Segundo Reis e Matos (2008), uma percentagem significativa de jovens sexualmente ativos está envolvida numa combinação perigosa de consumo de álcool e drogas, associadas a comportamentos de risco sexual. A elevada prevalência de IST e gravidez indesejada nas faixas etárias mais

jovens (Kann et al., 2016; WHO, 2011), traduzem a necessidade de melhorar a representação que os e as jovens possuem do que significa "sexo seguro", bem como as suas capacidades para se auto protegerem e protegerem os seus parceiros sexuais (APF, 2010).

O acesso universal a consultas e métodos contracetivos constitui uma forma privilegiada de diminuir a gravidez indesejada e as IST (DGS, 2015). Contudo, continuam a existir barreias e assimetrias no acesso aos serviços e cuidados de saúde reprodutiva sobretudo pelos jovens, conforme documentado no último estudo nacional sobre as práticas contracetivas (Sociedade Portuguesa de Ginecologia [SPG] & Sociedade Portuguesa de Contraceção [SPC], 2015), onde se constatou que 50% das jovens, entre os 20 e 29 anos, com vida sexual ativa e a usar contraceção, não frequentou no último ano a consulta de planeamento familiar. A falta de informação, os receios sobre a confidencialidade, a timidez e o medo de potenciais efeitos colaterais, associados a alguns métodos contracetivos ou mesmo desconhecimento sobre os métodos contracetivos disponíveis, são alguns dos fatores que contribuem para o adiamento da procura de informação sobre contraceção (APF, 2010; Ott, Sucato & Committee on Adolescence, 2014). A informação e educação sexual podem não ser eficazes se não forem acompanhadas pelo acesso a métodos contracetivos tendencialmente gratuitos e adequados às necessidades de cada jovem, incluindo a contraceção de emergência (CE) e os preservativos feminino e masculino (DGS, 2015). Os serviços e profissionais de saúde devem assegurar a educação contracetiva dos jovens, uma vez que diversos estudos comprovam o aconselhamento contracetivo melhora a adesão e diminui a taxa de descontinuação (Alli, Maharaj & Vawda, 2013; Neto et al., 2012).

As consequências dos comportamentos sexuais de risco nos jovens, nomeadamente a gravidez indesejada, a interrupção voluntária da gravidez e as IST, constituem um problema de saúde pública (Barros & Neves, 2013; Kirby et al., 2011), pelo que entender os fatores que podem influenciar o uso de métodos contracetivos, é essencial no delineamento de programas e planeamento de estratégias para promover o uso de métodos contracetivos de uma forma mais adequada e segura. O presente estudo teve como objetivo caracterizar as práticas contracetivas dos estudantes do ensino superior, atendendo às questões de género, assim como identificar fatores que contribuem para a utilização dos diferentes métodos contracetivos.

Metodologia

Foi realizado um estudo transversal, descritivo-correlacional numa amostra de 1946 estudantes (64% raparigas e 36% rapazes), com idade média de 21 anos (20,74±2,32) de uma Universidade do norte de Portugal. A maioria é de nacionalidade portuguesa (97,3%),

solteira (97,6%), proveniente da cidade (40%), de famílias com baixo rendimento (57%, ≤ 2 salários mínimos), baixo nível de escolaridade (54,5% das mães e 61,6% dos pais têm apenas o ensino básico) e com profissões pouco diferenciadas.

A recolha de dados foi realizada após envio do protocolo de investigação e aprovação do estudo pelos órgãos da direção da universidade onde foi realizado o estudo. A amostragem foi realizada por grupos *Cluster sampling,* onde o grupo ou a unidade de amostra foi a turma. O questionário de autopreenchimento foi aplicado em sala de aula, no final da componente letiva, a todos os estudantes que se disponibilizaram para participar, depois de informados sobre os objetivos do estudo e asseguradas as questões de anonimato e confidencialidade. Os detalhes de preenchimento foram comunicados a todos os estudantes, visando ter o mínimo número possível de questionários incorretamente preenchidos.

Na recolha de informação foram salvaguardados os princípios ético-deontológicos consignados na Declaração de Helsínquia e na lei. O estudo foi autorizado pela Comissão Nacional de Proteção de Dados (Autorização nº 7409/2013) e pela Comissão de Ética da universidade onde foi realizado (Parecer nº 2/2012).

Instrumentos

Na recolha de dados foi utilizado um questionário de autopreenchimento para registar aspetos sociodemográficos, comportamentos sexuais e de contraceção. Características sociodemográficas: sexo, idade (≤19, 20-24 anos e ≥25 anos), nacionalidade, local onde cresceu (urbano, rural), área científica de estudos (ciências da vida e saúde, outras ciências), nível de escolaridade dos pais (9 anos, 10 a 12 anos, ensino superior), rendimento familiar mensal (menos 2 salários mínimos; entre 2 e 4 salários mínimos, mais 4 salários mínimos), importância atribuída à religião (reduzida/nula, moderada, elevada). Comportamento sexual e contracetivo: utilização de preservativo na iniciação sexual, se teve sexo nos últimos 12 meses, uso de um método contracetivo, qual o contracetivo usado (nenhum, preservativo, hormonal, dupla – preservativo e hormonal), fontes de informação sobre contraceção (profissionais de saúde, de ensino, família, amigos, internet), local de aquisição, utilização de CE (frequência, motivo, aconselhamento por profissional de saúde). Foi introduzida uma questão de controlo numa no questionário para saber se os estudantes utilizavam o preservativo de forma consistente (utiliza sempre, frequentemente, raramente e nunca). Foram ainda colhidos dados sobre os fatores cognitivos e psicossociais que de acordo com a literatura podem influenciar as práticas contracetivas individuais, nomeadamente:

Conhecimento sobre contraceção: Para avaliar o conhecimento, utilizou-se o Inventário de Conhecimento sobre SSR, composto por 44 questões, construído especificamente para o estudo. Este instrumento é constituído por quatro dimensões que procuram avaliar o conhecimento sobre SSR: (1) informação relacionada à fisiologia e reprodução (2) métodos de contraceção, (3) IST e prevenção de riscos associados às IST (4) vigilância e cuidados de saúde. Os itens foram classificados usando três opções de resposta: "verdadeiro", "falso" e "não sei". As respostas corretas foram codificadas como um e as respostas incorretas ou " não sei", foram codificadas como zero, o que permite obter uma pontuação total entre 0 a 44 pontos, sendo que quanto maior a pontuação maior é o conhecimento. A confiabilidade do inventário de conhecimento foi avaliada utilizando-se o coeficiente *Kuder-Richardson* (KR20), um análogo ao alfa de *Cronbach* e que permite avaliar a consistência interna em estudos com escalas dicotômicas. O resultado foi KR20=0,78, confirmando a consistência interna adequada do instrumento. No presente estudo, usou-se somente a dimensão do conhecimento relacionada com a contraceção, constituída por 16 questões, com pontuação a variar entre 0-16 pontos.

Atitude SSR: A atitude de SSR foi medida usando uma escala de 20 itens, construída e validada no presente estudo. A escala é constituída por cinco dimensões (sexualidade responsável, atitudes preventivas, atitudes em relação às IST, atitudes em relação ao preservativo e hedonismo). As questões foram avaliadas numa escala tipo *Likert* com sete opções de resposta (1 – "discordo totalmente" e 7 – "totalmente de acordo"), e a pontuação total da escala pode variar entre 20 e 140 pontos, com a maior pontuação a indicar atitudes mais positivas. A escala revelou boa consistência interna (alfa de *Cronbach*=0,82).

Autoeficácia para usar o preservativo: Para avaliar a autoeficácia para usar o preservativo foi utilizada a versão reduzida da Escala de Autoeficácia para Usar o Preservativo (CUSES – Brien, Thombs, Mahoney & Wallnau, 1994), é composta por 15 itens, avaliados numa escala tipo *Likert* com cinco opções de resposta (0 – "discordo totalmente" a 4 – "concordo totalmente"), permite obter uma pontuação total entre 0 e 60 pontos; sendo que às pontuações mais elevadas corresponde uma maior autoeficácia para usar o preservativo. A escala tem uma estrutura multidimensional composta por quatro fatores (mecanismos, desaprovação do parceiro, assertividade e intoxicantes), e têm demonstrado boas características psicométricas de validade e fiabilidade nos diversos estudos onde foi utilizada, incluindo o estudo de validação em estudantes universitários. No presente estudo, utilizamos a versão traduzida e validada no presente estudo (CUSES-R, Santos, Ferreira & Ferreira, no prelo), que manteve uma estrutura fatorial semelhante à da escala original. A CUSES-R revelou um bom valor de alfa de *Cronbach* de (α=0,86), semelhante à consistência interna da escala original (α=0,85).

Suporte social: A satisfação com o suporte social foi avaliada pela Escala de Suporte Social (ESSS) proposta por Ribeiro (1999), que pretende avaliar o suporte social percebido e apoio que os respondentes recebem dos amigos, família e comunidade. A escala é composta por 15 itens, distribuídos por quatro dimensões (*satisfação com os amigos*, intimidade, satisfação com a família, atividades sociais). As questões são avaliadas numa escala tipo *Likert* com cinco opções de resposta (1- “concordo totalmente” a 5 – “discordo totalmente”), com exceção dos itens invertidos que são cotados de forma inversa. A pontuação total da escala varia entre 15 e 75 pontos e à pontuação mais elevada corresponde uma maior satisfação com o suporte social. Após o processo de validação da ESSS na nossa amostra, a escala ficou constituída por 12 itens distribuídos por quatro dimensões, as mesmas propostas pelo autor. Os valores de consistência interna revelaram-se adequados (α=0,814) e semelhante aos encontrados pelos autores quer no estudo de validação inicial (Escala: α=0,85; Fatores: α=0,64-0,83), quer no estudo mais recente realizado pelo autor de adaptação transcultural da ESSS com estudantes do ensino superior portugueses e brasileiros (Marôco, Campos, Vinagre & Pais-Ribeiro, 2014), onde a análise da consistência interna dos fatores revelou valores no limite da confiabilidade adequada (αSA=0,656; αIN=0,673; αSF=0,649; αAS=0,696).

Análise de dados

O tratamento estatístico dos dados foi efetuado com o programa *Statistical Package for the Social Sciences* (SPSS, Inc, Chicago, IL, USA), na versão 22.0. Na análise de dados foi utilizada a estatística descritiva: frequência ou medidas de tendência central e de dispersão, conforme a natureza dos dados. A associação entre as características sociodemográficas e as escolhas de contraceção dos estudantes foi estabelecida usando o teste de Qui-quadrado. A Relação entre os fatores cognitivos e psicossociais as escolhas de contracetivas foi estabelecida por análise de variância simples. A localização de diferenças entre médias foi feita com o teste *Tukey*. Em todas as estatísticas inferenciais tomou-se como limite de significância o valor de $p<0,05$.

Antes de proceder ao tratamento estatístico, as variáveis relacionadas com o uso de preservativo foram ajustadas com a questão de controlo que serviu para saber se o estudante utilizava o preservativo de forma consistente. Considerou-se que o estudante utiliza preservativo (isoladamente ou na dupla contraceção) somente quando na pergunta de controlo indicou usá-lo sempre. A utilização frequente ou esporádica foi considerada uma utilização não consistente de preservativo, e considerada não utilização. As proporções apresentadas relativas à utilização de métodos contracetivos são calculadas em relação aos estudantes que tiveram relações sexuais nos últimos 12 meses.

Resultados

As principais características das práticas contracetivas dos estudantes de ensino superior são apresentadas na Tabela 1. A população alvo do presente estudo foi constituída por 1946 estudantes, dos quais 76,9% indicou ter tido relações sexuais nos últimos 12 meses; essa proporção foi significativamente maior (p<0,001) no sexo masculino que no feminino (♂=81,9% vs ♀=74,1%). A esmagadora maioria dos respondentes apontaram uma orientação heterossexual. A maior preocupação das raparigas com a contraceção é muito clara quando se observa a proporção daquelas que referem não usar nenhum método contracetivo (7,2%), que é muito inferior à proporção de rapazes (32,6%). Quando se cruzou a informação sobre a utilização de métodos contracetivos com a pergunta de controlo destinada a avaliar a utilização consistente do preservativo, observou-se que cerca de metade dos estudantes (49,8%) que indicou usar preservativo, isoladamente ou em combinação com a pílula, não o usava consistentemente, pelo que foram considerados não utilizadores. O método contracetivo de eleição de raparigas e rapazes é diferente (p<0,001) e está muito associado à responsabilidade direta de quem o utiliza.

As raparigas apontam como principal escolha (52,9%) o método hormonal e os rapazes o preservativo (32,1%). A quase totalidade dos estudantes que referem usar o método hormonal utilizava a pílula. Os métodos contracetivos menos dependentes da utilizadora, designadamente o anel vaginal e o adesivo ou injetável, foram utilizados por um número muito reduzido de estudantes, cerca de 1%.

Ainda que num primeiro olhar para os resultados possa parecer que as raparigas aderem menos ao preservativo, pois somente 15,5% indica ser o seu método, quando observamos conjuntamente a proporção de utilização de preservativo e da dupla contraceção (definida como a combinação do método hormonal com o preservativo), observa-se que a utilização do preservativo vem a ser semelhante em ambos os géneros (♀=39,9% vs ♂=38,6%).

O esquecimento da toma da pílula uma vez foi referido por 45,1% das estudantes e duas vezes ou mais por 27,8% (dados não apresentados). No caso de esquecimento, 83,4% das estudantes tomou-a nas 24 horas seguintes, 14,2% usou preservativo e 2,3% recorreu à CE.

As fontes de informação sobre contraceção são diversas, havendo uma diferença muito clara entre raparigas e rapazes. Elas privilegiam mais os contextos formais, tais como os profissionais de saúde, e os rapazes os contextos informais, nomeadamente a escola, os amigos e a internet. Em termos familiares, a mãe é igualmente referida por ambos os géneros, mas a referência ao pai como fonte de informação foi quase exclusivamente mencionada pelo género masculino.

Tabela 1.
Caracterização das práticas contracetivas de estudantes do ensino superior

	Género			Total
	Feminino	Masculino	F; p	
**Total n**	1246	700		1946
**Teve relações sexuais nos últimos 12 meses**	923 (74,1)	573 (81,9)	14,827; p <0,001	1496 (76,9)
**Método contracetivo usado**				
Nenhum	67 (7,2)	186 (32,6)	290,540; p <0,001	253 (16,9)
Preservativo	143 (15,5)	183 (32,1)		326 (21,8)
Hormonal	489 (52,9)	164 (28,8)		653 (43,7)
Dupla contraceção	226 (24,4)	37 (6,5)		263 (17,6)
**Fonte de informação sobre contraceção** Ψ				
Enfermeiro	330 (35,8)	90 (15,7)	69,365; p <0,001	420 (28,11)
Médico	596 (64,6)	157 (27,4)	139,925; p <0,001	753 (50,3)
Farmácia	74 (8,0)	101 (17,6)	31,602; p <0,001	175 (11,7)
Mãe	267 (28,9)	163 (28,4)	0,40; p=0,842	430 (28,7)
Pai	44 (4,8)	154 (26,9)	148,564; p <0,001	198 (13,2
Amigos	174 (18,9)	230 (40,1)	81,275; p <0,001	404 (27,0)
Professores	163 (17,7)	227 (39,6)	88,427; p <0,001	390 (26,1)
Internet	182 (19,7)	257 (44,9)	107,708; p <0,001	439 (29,3)
**Onde adquire os contracetivos** Ψ				
Farmácia	556 (60,2)	356 (62,1)	0,455; p=0,500	912 (61,0)
Centro de Saúde	392 (42,5)	101 (17,6)	97,637; p <0,001	493 (33,0)
Supermercado; comércio em geral	130 (14,1)	257 (44,9)	174,510; p <0,001	387 (25,9)
**Usou CE no passado**	306 (24,6)	157 (22,5)	1,106; p=0,293	463 (23,8)
**Quantas vezes**				
Uma	214 (69,7)	98 (62,8)	9,468; p=0,009	312 (67,4)
Duas	73 (23,8)	34 (21,8)		107 (23,1)
Três ou mais	20 (6,5)	24 (15,4)		44 (9,5)
**Motivo para CE**				
Falha na contraceção regular	270 (87,9)	119 (76,3)	9,632; p=0,002	389 (84,0)
Ausência de contraceção	37 (12,1)	37 (23,7)		74 (16,0)
**Houve informação quando adquiriu a CE**	229 (74,6)	121 (77,6)	0,495; p=0,482	350 (75,6)
**Teve uma gravidez não desejada (ou a sua companheira)**	26 (2,8)	20 (3,5)	0,541; p= 0,462	46 (3,1)
**Utilizou os serviços de SSR**	379 (30,4)	53 (7,6)	160,029; p <0,001	432 (22,2)

Ψ - Questões que permitiam mais do que uma opção de resposta

No que respeita ao local e aquisição dos contracetivos, o padrão é similar ao das fontes de informação, já que as raparigas preferem obter os contracetivos em farmácias (60,2%) e no centro de saúde (42,5%), enquanto os rapazes, embora também prefiram a farmácia, usam muito (44,9%) o supermercado. Ainda que o local de aquisição dos contracetivos apresente diferenças significativas (p<0,001) entre os dois géneros, essas diferenças poderão não estar necessariamente associadas a escolhas ponderadas, mas sim pragmaticamente à

disponibilidade comercial do tipo de contracetivo. A utilização dos serviços de SSR pelos estudantes é reduzida (22,2%), e as raparigas a revelarem uma maior preocupação com a vigilância de saúde que os rapazes (♀=30,4% vs ♂=3,7%, p<0,001).

A utilização de CE é indicada por 23,8% dos estudantes, sendo similar (p=0,293) em ambos géneros. Não obstante, a reincidência na utilização da CE é diferente entre os géneros (p=0,009), com maior proporção de rapazes (15,9%) a indicarem a sua utilização repetida, três ou mais vezes, pela companheira. Observaram-se, também, diferenças entre os géneros (p=0.002), no que respeita à argumentação apresentada para a utilização deste método, uma vez que as raparigas apontam o recurso à CE por falha no método habitual (87,9%), mais frequentemente que os rapazes (73,1%), e uma percentagem significativa dos rapazes refere, ainda, a utilização de CE por relações sexuais sem proteção (23,7%). Destaca-se o facto de cerca de um quarto dos estudantes, de ambos os géneros, referir não ter recebido qualquer tipo de informação específica sobre a utilização deste método aquando da sua aquisição. A percentagem de estudantes que refere ter estado grávida sem o desejar (ou a companheira) é muito baixa (3,1%), em 27 dos casos o bebé nasceu, em 13 dos casos foi realizada interrupção voluntária da gravidez, em três casos ocorreu aborto espontâneo e em três casos a gravidez estava a decorrer.

A influência que os fatores individuais e ambientais estudados têm na escolha do tipo de contraceção entre os géneros é apresentada na Tabela 2. Os estudantes mais velhos tendem a valorizar mais a contraceção hormonal, aparentemente em substituição do preservativo, já que a adesão a este método apresenta um decréscimo com a idade, particularmente entre os rapazes mais novos (≤19 anos) e os de idades compreendidas entre os 20 e 24 anos. Para as raparigas, a contraceção hormonal é sempre o método de eleição, em todos os escalões etários. O meio onde os estudantes cresceram (urbano ou rural) e o rendimento da família não influenciam (p≥0,05) as escolhas contracetivas. A importância dada à religião não influencia (p≥0,05) a escolha das práticas contracetivas quando se faz a comparação por género. Porém, no total dos estudantes essa associação é significativa (p=0,004), e determinada pelos extremos – a maior proporção de estudantes que não dão importância à religião e não usam contraceção (27,3%), muito superior aos 15,9% e 15,6% dos que dão uma importância moderada ou elevada à religião, respetivamente, e aqueles que usam dupla contraceção, que tem uma tendência oposta, com a maior adesão por aqueles que são mais crentes (19,9%). Ter iniciado a atividade sexual usando preservativo reflete-se no seu uso atual. Em termos médios, para o total dos estudantes aquele acontecimento passado determina que 26,7% use preservativo, contra os 6,4% que usa preservativo atualmente, apesar de terem começado a ter relações sexuais sem o usar, com diferenças estatisticamente significativas (p<0,001) em ambos os géneros.

Tabela 2.
Influência dos fatores ambientais e individuais nas escolhas contracetivas dos estudantes do ensino superior

**Fatores Individuais e Ambientais**	**Feminino**					**Masculino**					**Total**				
	**Nen.**	**Pres.**	**Hor.**	**Dup.**	***Qui*** ^{2} ***p***	**Nen.**	**Pres.**	**Hor.**	**Dup.**	***Qui*** ^{2} ***p***	**Nen.**	**Pres.**	**Hor.**	**Dup.**	***Qui*** ^{2} ***p***
**Idade**															
≤ 19	**7,2**	**24,0**	42,3	26,5	*30,303*	30,9	**41,7**	**18,7**	8,6	*17,740*	15,1	**29,9**	**34,4**	20,6	*47,409*
20-24	**7,2**	**11,8**	56,7	24,2	*<0,001*	31,7	**28,09**	**32,8**	6,7	*0,007*	16,5	**18,2**	**47,7**	17,6	*<0,001*
≥ 25	7,7	11,5	65,4	15,4		40,8	29,6	28,2	1,4		**26,8**	22,0	43,9	**7,3**	
**Local onde cresceu**															
Urbano	6,4	15,3	56,1	22,3	*2,826*	33,6	31,0	29,9	5,5	*3,013*	18,0	21,7	42,5	17,7	*2,714*
Rural	7,8	15,6	50,9	25,8	*0,419*	30,7	34,4	26,5	8,5	*0,390*	15,0	22,1	45,6	17,4	*0,438*
**Rendimento familiar**															
<2 SM	7,6	16,1	52,0	24,3	*1,655*	34,2	33,8	25,2	6,8	*4,803*	16,6	22,1	42,9	18,3	*1,651*
2 - 4 SM	6,7	14,5	55,4	23,4	*0,949*	33,5	30,2	30,7	5,6	*0,569*	17,4	20,8	45,5	16,3	*0,949*
> 4 SM	6,9	147,7	50,9	27,6		27,4	31,0	34,5	7,1		17.0	22,7	42,8	17,5	
**Importância da religião**															
Reduzida/Nula	9,1	11,7	58,4	20,8	*7,811*	45,5	22,1	27,3	5,2	*11,924*	**27,3**	16,9	42,9	13,0	*18,819*
Moderada	6,2	13,4	56,6	23,9	*0,252*	30,6	35,1	29,8	4,4	*0,064*	15,9	22,1	45,9	16,1	*0,004*
Elevada	7,8	17,7	49,1	25,5		30,6	32,2	28,2	9,0		15,6	22,6	41,9	**19,9**	
**Utilizou preservativo na 1ª relação sexual**															
Não	7,1	**2,6**	56,4	**33,8**	*54,135*	29,5	**16,8**	**46,3**	7,4	*2,.951*	**13,0**	**6,4**	**53,7**	**26,9**	*90,596*
Sim	7,3	**20,6**	51,4	**20.6**	*<0,001*	33,3	**35,2**	**25,3**	6,3	*<0,001*	**18,2**	**26,7**	**40,5**	**14,6**	*<0,001*
**Rlações sexuais numa relação de namoro**															
Não	12,5	12,5	**33,3**	**41,7**	*12,026*	40,2	**50,9**	**6,3**	2,7	*45,453*	31,9	39,4	14,4	14,4	*85,312*
Sim	7,0	15,6	**53,9**	**23,5**	*0,007*	30,8	**27,5**	**34,3**	7,4	*<0,001*	15,1	19,7	47,2	18,0	*<0,001*
**Área científica de estudos**															
Ciências da Vida e Saúde	6,2	16,1	51,6	26,1	*5,563*	**28,9**	31,2	**32,7**	7,2	*9,326*	**14,5**	21,6	44,7	**19,3**	*14,483*
Ciências Humanas, Sociais e Tecnologias	9,2	14,2	55,4	21,2	*0,135*	**38,4**	33,5	**22,8**	5,4	*0,025*	**21,3**	22,2	41,9	**14,6**	*0,002*

Nen. Nenhum; **Pres.** Preservativo; **Hor.** Hormonal**; Dup.** Dupla; As frequências destacadas a negrito correspondem a resíduos padronizados ≥1.96, em valor absoluto.

Quando os estudantes têm sexo no contexto de uma relação de namoro têm uma maior adesão à contraceção hormonal e um concomitante abandono da utilização do preservativo, principalmente pelos rapazes. Como o envolvimento dos estudantes numa relação estável e a idade mais avançada são fatores associados ao abandono do preservativo, equacionou-se que se tratasse dos mesmos indivíduos – os mais velhos serem os que estão envolvidos numa relação. Porém, quando se testou essa associação, observou-se que não era significativa (χ^2=0,009; p=0,995).

A área científica de estudos dos participantes só teve reflexos nas escolhas contracetivas dos rapazes, observando-se que, dentro da elevada percentagem de rapazes que referem não usar contraceção (32,6%), a proporção dos que estudam em cursos da área de ciência da vida ou da saúde é significativamente menor (p=0,025), comparativamente aos que estudam noutras áreas, que ascendem a 38,4%. Entre as raparigas, a área de estudos não foi importante (p=0,135).

No sentido de avaliar a influência de aspetos cognitivos e psicossociais nas escolhas de contraceção feitas pelos estudantes, aplicaram-se vários instrumentos para recolher informação relativa ao conhecimento sobre contraceção, a atitude face à SSR, autoeficácia para o uso de preservativo e a satisfação com o suporte social (Tabela 3). O conhecimento sobre contraceção influenciou as escolhas dos estudantes. Entre as raparigas, as que não usam contraceção são as que têm um conhecimento menor (8,49±2,53) e significativamente diferente ($p<0,05$), daquelas que usam a dupla contraceção pílula/preservativo (9,54±2,09). No grupo dos rapazes, curiosamente os que têm menos conhecimento não são os que não usam contraceção, mas sim os que usam somente preservativo, distinguindo-se daqueles que fazem sexo com dupla proteção.

A atitude SSR segue um padrão semelhante ao observado para o conhecimento, sendo a atitude de SSR mais desfavorável observada nos estudantes que não usam contraceção e a mais favorável nos que usam dupla contraceção. Nas raparigas, a diferença entre os extremos é de cerca de cinco pontos e nos rapazes cerca de oito.

Tabela 3.
Influência dos fatores cognitivos e psicossociais nas escolhas contracetivas dos estudantes do ensino superior

Fatores cognitivos e psicossociais	Género	Nenhum	Preservativo	Hormonal	Dupla	F (p)
**Conhecimento sobre contraceção**	Raparigas	8,49±2,53a	8,66±2,20a	9,05±1,98ab	9,54±2,09b	7,257 (<0,001)
(0-16) Ψ	Rapazes	7,37±2,81ab	7,01±2,56a	7,67±2,32ab	8,14±2,43b	3,020 (0,029)
	Total	7,67±2,78a	7,74±2,54a	8,71±2,15b	9,34±2,19c	33,453 (<0,001)
**Atitude SSR**	Raparigas	119,09±12,17a	122,36±12,69ab	121,24±11,06ab	124,28±10,88b	2,274 (0,001)
(20-140) Ψ	Rapazes	110,38±16,22a	112,44±16,60a	112,63±14,68ab	118,51±14,74b	2,838 (0,037)
	Total	112,68±15,71a	116,79±15,78b	119,07±12,62b	123,47±11,64c	20,372 (<0,001)
**Autoeficácia para usar o preservativo**	Raparigas	44,24±9,43a	50,31±7,61b	49,57±8,27b	50,03±7,54b	10,213 (<0,001)
(0-60) Ψ	Rapazes	47,40±9,59	47,81±9,42	48,38±9,70	50,46±9,21	14,177 (0,318)
	Total	46,56±9,63a	48,90±8,75b	49,27±8,66b	50,09±7,78b	8,109 (<0,001)
**Perceção de suporte social**	Raparigas	44,15±7,22	45,90±7,21	44,67±7,43	45,50±6,99	1,722 (0,161)
(15-75) Ψ	Rapazes	45,13±7,28	44,97±7,62	46,09±7,01	47,25±6,78	1,531 (0,205)
	Total	44,87±7,26	45,38±7,45	45,02±7,35	45,74±6,98	0,844 (0,470)

Ψ Scores mínimos e máximos das escalas, **a, b, c** - médias seguidas de letras diferentes na mesma linha apresentam diferenças significativas (p<0,05).

A escala de autoeficácia para o uso do preservativo confirma a tendência observada com os dois constructos precedentes. A autoeficácia dos estudantes para usar o preservativo é elevada, e são os estudantes que não usam contraceção os que têm uma menor autoeficácia para usar o preservativo. Essas diferenças não têm significado estatístico no grupo dos rapazes, pois mesmo os que não usam contraceção têm pontuações elevadas e semelhantes aos que usam outros métodos contracetivos. Entre as raparigas, as que não usam contraceção têm uma autoeficácia para o uso do preservativo significativamente menor que as que usam contraceção, independentemente do método escolhido. A satisfação com o suporte social não mostrou qualquer influência nas escolhas contracetivas feitas pelos estudantes.

Discussão

A utilização de métodos contracetivos de forma regular e eficaz depende de uma grande variedade de fatores, que devem ser considerados na criação das políticas e programas de promoção de saúde para a juventude. Os estudantes universitários com quem este trabalho foi conduzido têm comportamentos de sexualidade e contraceção dentro do habitualmente observado nesse grupo populacional (Oliveira, 2014; Pacheco, 2012; Reis, Matos, Simões &

Diniz, 2011; Trieu, Bratton & Marshak, 2011). A maioria é sexualmente ativa, frequentemente numa relação de namoro e utiliza como método contracetivo, a pílula ou preservativo, preferido por raparigas e rapazes, respetivamente. A tendência das raparigas utilizarem maioritariamente contraceção hormonal prende-se com a sua elevada eficácia e facilidade de utilização, uma vez que são elas que, na maior parte das situações, assumem a responsabilidade pela escolha do método contracetivo. Por sua vez, o método contracetivo mais utilizado pelos rapazes é o preservativo, que é muito recomendado, dado carácter espontâneo e muitas vezes não planeado das relações sexuais nesta faixa etária (Tyler et al., 2014). A adesão aos métodos de contraceção menos dependente das utilizadoras (anel vaginal e o adesivo contracetivo), e de longa duração (implante contracetivo e a contraceção intrauterina), foram pouco utilizados pelas participantes, embora o seu uso esteja associado a um aumento da eficácia e continuidade na utilização (Barros & Neves, 2013). Dada a sua elevada eficácia, são atualmente recomendados a mulheres de todas as idades, inclusive adolescentes e jovens (DGS, 2015). No último estudo sobre práticas contracetivas das mulheres portuguesas (SPG & SPC, 2015) observou-se também uma fraca utilização de métodos não dependentes da utilizadora, embora com uma tendência de aumento face aos anos anteriores, tais como o anel vaginal (2,7%), implante subcutâneo (5,4%), adesivo (3%) e DIU (11,8%). Em 34% dos casos, a má adesão à toma regular da pílula foi o motivo que determinou a mudança para outro método, sobretudo nas faixas etárias mais jovens, uma vez que 29,4% das mulheres entre os 20 e os 29 anos refere que se esquece de tomar a pílula pelo menos uma vez em todos os ciclos.

A dupla contraceção tem sido reconhecida como uma estratégia muito segura não só do ponto de vista de prevenção da gravidez, mas também da prevenção de IST, pelo que é recomendável a sua utilização, em particular por jovens (López, Otterness, Chen, Steiner & Gallo, 2013; SPG & SPC, 2011; Tyler et al., 2014). A amostra alvo do presente estudo não reflete essa tendência comportamental promotora de saúde, pois há dois fatores que determinam a substituição do preservativo – a idade mais avançada e o sexo no contexto de namoro. Os estudantes mais velhos são os que apresentam um comportamento contracetivo mais inconsistente. O abandono do uso do preservativo pode resultar não só da alegada redução no prazer (Gomes & Munes, 2011), mas também da personalidade com forte tendência para procura de novas sensações sexuais, mais marcada em rapazes mais velhos (Gullette & Lyons, 2006; Voisin, King, Schneider, Diclemente & Tan, 2012) e, por outro, à baixa perceção de risco de transmissão de IST (O'Sullivan et al., 2010; Wilton et al., 2014). A implicação que a relação de compromisso tem nas escolhas contracetivas com redução na utilização consistente do preservativo tem sido amplamente identificada em jovens (Chamratrithirong & Kaiser, 2012; Goldstein, Upadhyay & Raine, 2013; Hickey &

Cleveland, 2013; Janeiro, Oliveira, Rodrigues, Macieiras & Rocha, 2013). Se, do ponto de vista da prevenção da gravidez, é uma decisão sensata, do ponto de vista da prevenção das IST, essa decisão assenta numa forte confiança sobre o passado sexual do companheiro e na monogamia (Certain et al., 2009). No entanto, muitos parceiros sexuais mentem quanto ao seu passado sexual, e há taxas elevadas de envolvimento sexual não assumido fora das relações de compromisso (Grello, Welsh & Harper, 2006). Este impacto negativo que as relações mais estáveis têm na utilização do preservativo merece atenção, devendo, por isso, ser estudado de forma mais aprofundada, uma vez que pode aumentar a vulnerabilidade às IST (APF, 2010).

Num estudo relativamente recente sobre práticas contracetivas das mulheres portuguesas (SPG & SPC, 2015) detetou-se que, para a população em geral, a utilização da CE está a aumentar (17%), o que tem alimentado um vasto debate sobre a utilização da CE como método contracetivo de uso regular, mais do que para atender a situações excecionais, para as quais foi concebida. No presente trabalho, a utilização da CE é da mesma ordem de grandeza da média nacional acima referida, mas cerca de dez vezes superior à observada por Reis et al. (2011), também com estudantes universitários. Esta disparidade entre os resultados destes últimos autores e os que observamos no presente trabalho aponta para um maior uso da CE. Amengual, Canto, Berenguer e Pol (2016) verificaram que a maioria das utilizadoras da CE em Espanha, são jovens dos 21 aos 24 anos, na sua maioria estudantes universitárias, que apontam como motivo para o recurso a este método a rotura do preservativo.

No contexto do aconselhamento contracetivo está recomendado a orientação dos jovens sobre a utilização da CE, dado que é a ultima oportunidade para evitar uma gravidez, após uma relação sexual não protegida ou mesmo falha no método habitual. No entanto, o aconselhamento deve ser sempre acompanhado da mensagem de que a CE é segura, não tem contraindicações, embora com uma eficácia contracetiva inferior à de qualquer um dos métodos de contraceção regular (Barros & Neves, 2013; International Consortium for Emergency Contraception [ICEC], 2015). Neste sentido, a procura da CE deve constituir uma oportunidade para os profissionais de saúde promoverem o uso de métodos de contraceção regular e adequados às necessidades de cada jovem (DGS, 2015; ICEC, 2015). A formação dos profissionais de saúde para as necessidades específicas dos estudantes universitários é necessária para garantir que os estudantes não encontram barreiras nos próprios serviços que os deviam apoiar, nomeadamente na indicação de contracetivos adequados à sua vivência da sexualidade ou apoio à utilização de CE (Moscou, 2016).

O princípio de que comportamento anterior é um bom preditor do comportamento atual (Asare, 2015; Protogerou, Fisher, Wild & Aaro, 2013), aplica-se aos estudantes que participaram no presente estudo, já que os rapazes que usam preservativo e que usaram o preservativo na primeira relação sexual são o dobro dos que não o usaram, sendo essa relação de grandeza cerca de dez vezes no caso das raparigas.

Embora fosse expectável que os estudantes do ensino superior detivessem elevado conhecimento e competências no domínio da contraceção, os resultados do presente trabalho não confirmam essa expectativa, já que cerca de 7% das raparigas e 33% dos rapazes indicaram ter feito sexo sem utilizar qualquer tipo de contraceção. Esta proporção desviada para o lado dos rapazes é comummente observada em jovens universitários (Reis et al., 2013), sendo justificada pela maior valorização da vertente hedonista do sexo, que procuram uma oportunidade de ter sexo, mesmo que não estejam reunidas as condições de proteção (Gaither & Sellbom, 2003; Hendershot, Stoner, George & Norris, 2007). Por outro lado, pelas consequências da gravidez afetarem mais diretamente as raparigas, estas são inerentemente mais prudentes. Nesse sentido, elas utilizam fontes de informação sobre contraceção mais formais que os rapazes, assim como recorrem mais frequentemente aos serviços de SSR, quer para aconselhamento, quer para aquisição do método contracetivo. A evidência sugere que os jovens, e em particular os rapazes, continuam a não privilegiar como fontes de informação os profissionais de saúde (Ribeiro & Fernandes, 2009; Sant'Anna et al., 2008), obtendo informação sobre contraceção, sobretudo nas aulas de educação sexual, em conversas com os amigos e na internet. No contexto familiar, a mãe é o elemento mais ativo na transmissão de conhecimento sobre contraceção, estando o papel do pai reservado essencialmente para a educação dos rapazes. Esta tendência é frequentemente observada na educação sexual prestada pelas famílias aos filhos (Harris, 2016). A utilização dos serviços e cuidados de SSR pelos participantes no presente estudo é reduzida, assim como o é na generalidade da população. Informação sistematizada pela SPG e SPC (2015) aponta para 40% das mulheres com vida sexual ativa e a usar contraceção que não frequentaram, no último ano, a consulta de SSR, situação que é mais grave entre os grupos mais jovens - 90% adolescentes e 50% entre os 20 e 29 anos.

Os resultados observados apontam para uma associação positiva entre um maior conhecimento de SSR, atitudes SSR mais favoráveis e comportamentos mais protetores. Um dos achados do presente estudo e que merece alguma reflexão é o facto dos estudantes que não usam de todo contraceção serem os que têm um conhecimento mais limitado e uma atitude mais desfavorável. O conhecimento tem sido apontado como uma estratégia importante para melhorar as práticas de contraceção (Kirby et al., 2011; Reis et al., 2013), o que tem alicerçado as políticas de contraceção de vários países, onde a escola

tem sido o canal privilegiado para a implementação dessas políticas (Rocha & Duarte, 2016). Considerando o adiamento da maturidade dos jovens para aquilo que se convencionou chamar a adultez emergente (Arnett, 2004) e as oportunidades de melhoria identificadas no grupo de estudantes alvo do presente trabalho, é inevitável equacionar a hipótese de considerar o contexto universitário um ambiente favorável à aprendizagem sobre contraceção. A forma de transmitir esse conhecimento e competências não poderia ter o carácter formal que tem no ensino secundário, mas podem ser realizadas atividades formativas sobre métodos de contraceção, à semelhança das que são promovidas por diversas entidades de saúde pública ligadas à prevenção do VIH/SIDA para promover o uso do preservativo. Os serviços de saúde devem disponibilizar aconselhamento sobre contraceção, distribuição gratuita de contracetivos, consultas de vigilância de saúde que, no caso dos jovens sexualmente ativos, devem incluir o rastreio de IST, estando recomendado a realização de testes anuais de pesquisa de *Chlamydia* e *Neisseria Gonorrhoeae* em jovens até aos 25 anos (Mansouri & Santos, 2014).

A eficácia do uso do preservativo e de outros métodos contracetivos, para além do conhecimento, pode estar dependente de outros fatores, tais como a autoeficácia, a influência dos pares e dos contextos sociais. Os resultados revelam que os níveis de autoeficácia são superiores nos estudantes que utilizam contraceção, o que reflete a importância deste constructo como determinante do comportamento de usar contraceção. Diferentes estudos têm demonstrado esta associação (Asare, 2015; Kirby et al., 2011; Reis et al., 2013), constatando que níveis mais elevados de autoeficácia estão associados não só a uma maior utilização do preservativo, mas também à capacidade de ultrapassar barreiras na sua aquisição e uma utilização mais consistente. A perceção do suporte social é considerado um fator protetor de comportamentos de risco entre os jovens (Pollock, 2016), contudo, no presente estudo não se revelou importante nas escolhas contracetivas dos estudantes.

Como limitações do estudo mencionamos o método de amostragem e o desenho transversal do estudo. O método de amostragem não probabilístico pode condicionar de alguma forma a generalização dos resultados. Esta limitação pode ter sido minimizada pelo facto de se ter realizado a aleatorização das turmas. O desenho transversal que não permite estabelecer relações de causalidade e perceber as mudanças comportamentais ao longo do tempo, pelo que seria interessante recorrer a estudos longitudinais, com o objectivo de avaliar a evolução comportamental. O facto das medidas de intenção comportamental serem auto-referidas, o pode não ser uma medida precisa do comportamento real e implica que se considerem aspetos como a desejabilidade social ou o estilo de reposta.

Conclusões

Os resultados do presente estudo refletem a necessidade de se investir na promoção da SSR dos estudantes do ensino superior, numa perspetiva de género, melhorando o conhecimento, a atitude face aos métodos contracetivos e autoeficácia para usar o preservativo, fatores que estiveram associados a escolhas contracetivas mais seguras. A utilização dos métodos de contraceção é elevada, contudo, as práticas contracetivas revelam sobretudo uma preocupação com a gravidez indesejada, com o uso inconsistente ou mesmo abandono do preservativo, em particular pelos estudantes mais velhos e que estão envolvidos numa relação de namoro, o que deixa os estudantes mais vulneráveis às IST. Cerca de metade dos estudantes que refere usar preservativo, não o usou forma consistente.

Insistir na aprendizagem sobre contraceção e prevenção de IST, durante a vida universitária não é extemporâneo, pelo contrário, centrar estratégias de aprendizagem no momento em que se adquirem ou consolidam rotinas de contraceção, reforçando o comportamento contracetivo é essencial para potenciar escolhas adequadas, melhorar a adesão e diminuir a taxa de descontinuação dos métodos contracetivos. As estratégias de educação para a saúde devem ser complementadas com a divulgação e disponibilização de serviços de SSR adequados às necessidades dos jovens, atendendo também às necessidades de SSR dos rapazes, assim como da implementação de campanhas de prevenção universal, visando o esclarecimento e treino de competências relacionadas com a sexualidade, contraceção, práticas de sexo seguro, e sensibilização dos jovens para a importância da vigilância regular de saúde com rastreio das IST mais prevalentes nesta faixa etária.

Referências bibliográficas

Alli, F., Maharaj, P., & Vawda, M. (2013). Interpersonal relations between health care workers and young clients: Barriers to accessing sexual and reproductive health care. *Journal of Community Health, 38*(1), 150-155. doi:10.1007/s10900-012-9595-3

Amengual, M. L. B., Canto, M. E., Berenguer, I. P., & Pol, M. I., (2016). Revisão sistemática do perfil de usuárias de contracepção de emergência. *Revista Latino-Americana de Enfermagem***,** *24*, e2733.

Arnett, J. J. (2004). *Emerging adulthood: The winding road from the late teens through the twenties*. New York, NY: Oxford University Press.

Asare, M. (2015). Using the theory of planned behavior to determine the condom use behavior among college students. *American Journal of Health Studies, 30*(1), 43–50.

Associação para o Planeamento da Família. (2010). *Direitos e saúde sexual e reprodutiva de jovens na Europa. Um guia para o desenvolvimento de políticas sobre direitos e saúde sexual e reprodutiva de jovens na Europa*. Lisboa: Autor.

Barros, J., & Neves. J. (2013). Contraceção na adolescência. In J. Neves, *Contraceção* (pp. 121-134). Lisboa: Lidel.

Brien, T. M., Thombs, D. L., Mahoney, C. A., & Wallnau, L. (1994). Dimensions of self-efficacy among three distinct groups of condom users. *Journal American College Health*, *42* (4), 167-174. doi:10.1080/07448481.1994.9939665

Burnett, A. J., Sbato, T. M., Ott walter, K., Kerr, D. L., Wagner, I., & Smith, A. (2014). The influence of attributional style on substance use and risky sexual behavior among college students. *College Student Journal*, *47*(1), 122-136.

Certain, H., Harahan, B., Saewyc, E., & Fleming, M. (2009). Condom use in heavy drinking college students: The importance of always using condoms. *Journal of American College Health*, *58*(3), 187-194. doi:10.1080/07448480903295284

Chamratrithirong, A., & Kaiser, P. (2012). The dynamics of condom use with regular and casual partners: Analysis of the 2006 national sexual behavior survey of Thailand. *PLoS ONE*, *7* (7), e42009, 1-11. doi:10.1371/journal.pone.0042009

Direção-Geral da Saúde. (2015). *Orientação nº 10/2015: Disponibilidade de métodos contracetivos*. Lisboa: Autor.

Gaither, G. A., & Sellbom, M. (2003). The sexual sensation seeking scale: Reliablity and validity within a heterosexual college student sample. *Journal of Personality Assessment, 81*(2), 157-167. doi:10.1207/S15327752JPA8102_07

Goldstein, R. L., Upadhyay, U. D., & Raine, T. R. (2013). With pills, patches, rings, and shots: Who still uses condoms? A longitudinal cohort study. *The Journal of Adolescent Health*, *52*(1), 77-82. doi:10.1016/j.jadohealth.2012.08.001

Gomes, A., & Nunes, C. (2011). Caracterização do uso do preservativo em jovens adultos portugueses. *Análise Psicológica*, *4*(29), 489-503.

Grello, C. M., Welsh, D. P., & Harper, M. S. (2006). No strings attached: The nature of casual sex in college students. *The Journal of Sex Research*, *43*(3), 255-267. doi: 10.1080/00224490609552324

Gullette, D. L., & Lyons, M. A. (2006). Sensation seeking, self-esteem, and unprotected sex in college students. *Journal of Association of Nursing in AIDS Care*, *17*(5), 23-31. doi: 10.1016/j.jana.2006.07.001

Harris, A. L. (2016). African American parent–son sexual communication among a college sample. *Journal of Pediatric Nursing,* *31*(3), 199-206. doi:10.1016/j.pedn.2015.12.006.

Hendershot, C. S., Stoner, S. A., George, W. H., & Norris, J. (2007). Alcohol use, expectancies and sexual sensation seeking as correlates of HIV risk behaviour in heterosexual young adults. *Psychology of Addictive Behaviors*, *21*(3), 365-372. doi:10.1037/0893-164X.21.3.365

Hickey, M. T., & Cleland, C. (2013). Sexually transmitted infection risk perception among female college students. *Journal American Association of Nurse Practitioners*, *25*(7), 377-384. doi:10.1111/j.1745-7599.2012.00791.x

International Consortium for Emergency Contraception. (2015). Repeated use of emergency contraceptive pills: The facts. New York. Retirado de http://www.cecinfo.org/custom-content/uploads/2015/10/ICEC_Repeat-Use_Oct-2015.pdf

Janeiro, J.V., Oliveira, I.S., Rodrigues, I.S., Macieiras, M.J., & Rocha, G. M. (2013). As atitudes sexuais, contraceptivas, o lócus de controle da saúde e a autoestima em estudantes do ensino superior. *Revista Brasileira de Promoção da Saúde* 26(4), 505 512. doi:10.5020/18061230.2013.p505

Kann, L., McManus, T., Harris, W., Shanklin, S. L., Flint, K., Hawkins, J., … Zaza, S. (2016). *Youth Risk Behavior Surveillance - United States, 2015*. Centers for Disease Control and Prevention, *65*(6), 1-171. doi:10.15585/mmwr.ss6506a1

Kirby, D., Coyle, K., Alton, F., Rolleri, L., & Robin, L (2011). *Reducing adolescent sexual risk. Theoretical guide for developing and adapting curriculum-based programs*. California: ETR Associates.

Kuperberg, A., & Padgett, J. E. (2015). Dating and hooking up in college: Meeting contexts, sex, and variation by gender, partner's gender, and class standing. *The Journal of Sex Research*, *52*(5), 517-531. doi:10.1080/00224499.2014.901284

Lazarus, J. V., Sihvonen-Riemenschneider, H., Laukamm-Josten, U., Wong, F., & Liljestrand, J. (2010). Systematic Review of Interventions to prevent spread of sexually transmitted infections, including hiv, among young people in Europe. *Croatian Medical Journal*, *51* (1), 74-84. doi: 10.3325/cmj.2010.51.74

Lopez, L. M., Otterness, C., Chen, M., Steiner, M., & Gallo, M. F. (2013). Behavioral interventions for improving condom use for dual protection. *Cochrane Database System Review, 10*, CD010662. doi:10.1002/14651858.CD010662.pub2

Mansouri, R., & Santos, X. M. (2014). Doenças sexualmente transmissíveis em adolescentes. *Patient Care. 19*(199), 23-31.

Marôco, J. P., Campos, J. A., Vinagre, M. G., & Pais-Ribeiro, J. L. (2014). Adaptação transcultural Brasil-Portugal da Escala de Satisfação com o Suporte Social para estudantes do ensino superior. *Psicologia Reflexão e Crítica*, *27*(2), 247-256.

Moscou, S. (2016). Meeting contraceptive needs of college students: Bringing evidence into practice. *The Journal for Nurse Practitioners*, *12*(1), e11-e15. doi.org/10.1016/j.nurpra.2015.09.013

Neto, S., Bombas, T., Arriaga, C., Almeida, M.C., & Moleiro, P. (2012). *Contraceção na adolescência: Recomendações para o aconselhamento contracetivo*. Lisboa: Sociedade Portuguesa de Medicina do Adolescente da Sociedade Portuguesa de Pediatria.

O'Sullivan, L. F., Udell, W., Montrose, V. A., Antoniello, P., & Hoffman, S. (2010). A cognitive analysis of college students' explanations for engaging in unprotected sexual intercourse. *Archives of Sexual Behavior*, 39(5), 1121–1131. doi:org/10.1007/s10508-009-9493-7

Oliveira, A. C. (2014). *VIH/SIDA e comportamentos de risco: Monitorizar a evolução*. Tese de Doutoramento não publicada, Faculdade de Medicina da Universidade de Coimbra.

Ott, M. A., Sucato, G. S., & Committee on Adolescence. (2014). *Contraception for adolescents. Pediatrics*, *134*(4), 1257-1281. doi:10.1542/peds.20142300

Pacheco, N. S. (2012). *A sexualidade dos jovens estudantes universitários portugueses*. Tese de Doutoramento não publicada, Universidade da Beira Interior, Covilhã.

Pollock, T. (2016). *The effects of alcohol consumption and perception of normative behaviors on risky sexual behavior among college students*. Thesis submitted in partial fulfillment of the requirements for Honors Studies in Social Work. The University of Arkansas.

Protogerou, C., Flisher, A. J., Wild, L., & Aaro, L. E. (2013). Predictors of condom use in South African university students: A prospective application of the theory of planned behavior. *Journal of Applied Social Psychology, 43*(S1), 23-36. doi:10.1111/jasp.12039

Reis, M., & Matos, M. G. (2008). Contraceção em jovens universitários portugueses. *Análise Psicológica*, *26*(1), 71-79.

Reis, M., Matos, M., Simões, C., & Diniz, J. (2011). *A saúde sexual e reprodutiva dos estudantes do ensino superior: Dados nacionais 2010. Problemas emergentes e modelo compreensivo. Aventura Social*. Lisboa. Lisboa: Edições FMH.

Reis, M., Ramiro L., Matos M. G., & Diniz, J. A. (2013). Determinants influencing male condom use among university students in Portugal. *International Journal of Sexual Health, 25*(2), 115-27. Doi: org/10.1080/19317611.2012.728554

Ribeiro, J. L. P. (1999). Escala de satisfação com o suporte social. *Análise Psicológica, 17*(3), 547-558.

Ribeiro, M. I., & Fernandes, J. (2009). Comportamentos sexuais de risco em estudantes do ensino superior. Público da cidade de Bragança. *Psicologia, Saúde e Doenças*, *10*(1), 99-113.

Rocha, A. C., & Duarte, C. (2016). Impacto das políticas públicas na promoção da educação sexual: o caso português. *Global Journal of Community Psychology Practice*, *7*(1s), 1-23.

Rodrigues, P., Salvador, A., Lourenço, I., & Santos, L. (2014). Padrões de consumo de álcool em estudantes da Universidade de Aveiro: Relação com comportamentos de risco e stresse. *Análise Psicológica, 4*(32), 453-466. doi:10.14417/ap.789

Sant'Anna, M. J. Carvalho, K. A.; Bastos, M. B., & Coates, P. V. (2008). Sexual behavior of young university students. *Adolescência & Saúde*, *5*(2), 52-56.

Santos, M. J., Ferreira, E., & Ferreira, M. (in press). Portuguese adaptation and validation of the brief version of Condom Use Self-efficacy Scale in college students. *Revista Internacional de Andrologia.* Under review.

Sociedade Portuguesa de Ginecologia & Sociedade Portuguesa da Contraceção. (2015). *Estudo das práticas contracetivas das mulheres portuguesas*. Lisboa: Autor.

Ssewanyana, D., Sebena, S., Petkeviciene, J., Lukács, A., Miovsky, M., & Stock, C. (2015). Condom use in the context of romantic relationships: A study among university students from 12 universities in 4 Central and Eastern European countries. *Europen*

Journal Contraception and Reproductive Health Care, 9, 1-11. doi: 10.3109/13625187.2014.1001024

Trieu, S. L., Bratton, S., & Marshak, H. (2011). Sexual and reproductive health behaviors of california community college students. *Journal of American College Health*, *59*(8), 744-750. doi:10.1080/07448481.2010.540764

Tyler, C. P., Whiteman, M. K, Kraft, J., Zapata, L., Hillis, S., Curtis, K., … Marchbanks, P.A. (2014). Dual use of condoms with other contraceptive methods among adolescents and young women in the United States. *Journal of Adolescent Health*, *54*(2), 169-175. doi: 10.1016/j.jadohealth.2013.07.042

Voisin, D., King, K., Schneider, J., Diclemente, R. & Tan, K. (2012). Sexual sensation seeking, drug use and risky sex among detained youth. *AIDS & Clinical Research*, *S1*, 1-5. doi: 10.4172/2155-6113.S1-017

Wicki, M., Kuntsche, E., & Gmel, G. (2010). Drinking at European universities? A review of students' alcohol use. *Addictive Behaviors*, *35*, 913-924. doi:10.1016/j.addbeh.2010.06.015

Wilton, L., Palmer, R. T., & Maramba, L. C. (2014). *Understanding HIV and STIs prevention for college students*. London: Routledge Research in Higher Education.

World Health Organization. (2011*). The sexual and reproductive health of younger adolescents: Research issues in developing countries*. Retirado de http://apps.who.int/iris/bitstream/ 10665/44590/1/9789241501552_eng.pdf

CAPÍTULO 4

ESTUDOS EMPÍRICOS EM SAÚDE SEXUAL E REPRODUTIVA

4.3. Artigo 3 - Risk and protective factors for college students, sexual and reproductive health

Santos, M.J., Ferreira, E., Duarte, J., & Ferreira, M.

(Artigo submetido à *International Nursing Review*, INR-2016-0221)

RISK AND PROTECTIVE FACTORS FOR PORTUGUESE COLLEGE STUDENTS` SEXUAL AND REPRODUCTIVE HEALTH

M.J. Santos[1]; E. Ferreira[2]; J. Duarte[3]; M. Ferreira[3]

[1] Escola Superior de Enfermagem de Vila Real, Universidade de Trás-os-Montes e Alto Douro, Vila Real, Portugal. mjsantos@utad.pt

[2] Faculdade de Psicologia e Ciências da Educação, Universidade do Porto, Porto, Portugal

[3] Escola Superior de Saúde de Viseu, Instituto Politécnico de Viseu, Viseu, Portugal

Abstract

Background: College students have unprotected sex and associate alcohol and/or drugs with sexual practices. These behaviours are risk factors for sexual and reproductive health that might be targeted by nursing interventions.

Aim: To determine the risk and protective factors influencing sexual and reproductive risk-taking behaviour in Portuguese college students.

Methods: A cross-sectional study was conducted in a sample of 1946 students. Data were collected using a self-report questionnaire on the individual, environmental, cognitive and psychosocial factors, namely Knowledge of STIs, Attitude for sexual and reproductive health, Subjective norms, Condom Use Self-Efficacy and Sexual Sensation Seeking. Four sexual and reproductive risk behaviours were considered: having sex (1) without a condom (2) while under the influence of alcohol or (3) drugs and (4) with casual partners.

Results: Older male students were at higher risk for all risk behaviours studied. Students involved in a committed relationship were at risk because abandon the use of condom. The high importance given to sexual sensation seeking was highly associated to sexual risk. The higher the importance students placed on peers' opinion, the more protective were their behaviours.

Conclusions: Nursing interventions should include strategies addressing the high importance given to the hedonic perspective of sex, as well as the opportunities to use peers' based interventions to achieve the goal of reducing the exposure to STI´s.

Implications for nursing and health policy: The results of this paper showed that college students are exposed to sexual risks. The effort made with adolescents on sexual education and sexual risk prevention should be continued at the transition to adulthood.

Keywords: College Students, condom, nursing, sexual and reproductive health, sexual risk behaviour.

Introduction

Sexually transmitted infections (STIs) continue to be a public health concern of the actuality, with high prevalence rates in the young people across Europe. Surveillance reports both from the US and the EU demonstrate a trend of a high prevalence of *Chlamydia,* gonorrhea, syphilis, HPV, genital herpes and HIV, particularly in the 15–24 age groups (CDC, 2014; ECDC & WHO 2014). Portugal follows the trend of EU countries for most of the STI`s, with highest prevalence of HIV (11.2%) and HPV (28.8%) in young people (ECDC & WHO 2014; Pista et al., 2011). The STI´s can lead to serious long term sexual and reproductive health (SRH) consequences, especially for young women, such as the pelvic inflammatory disease, infertility and cervical cancer (Singh, Darroch & Ashford, 2014).

Young adults are at risk of STIs due to the combination of behavioural, biological and cultural factors. College provides a new and important context in which emerging adults learn to manage their intimate relationships and sexuality (Claxton & van Dulman, 2015). It is also in that period that students having unprotected sex, associate alcohol or drugs with sex, or have sex with casual partners. Because of the social nature of college life, alcohol and drug abuse is common (Burnett Sabato, Wagner & Smith, 2014; Wicki, Kuntsche & Gmel, 2010). Impaired judgment, typical of alcohol and drug (ab)use has direct consequences on the ability and likelihood of using a condom or negotiating it with a partner (Uecker, 2015; Wilton, Palmer & Maramba, 2014).

College is also a period of enjoying new experiences, which frequently leads to sex with casual sex partners. Alcohol may facilitate casual sex by reducing inhibitions, increasing confidence to approach new partners, or increasing susceptibility to social pressures (Fielder & Carey, 2010). These behaviours have been identified as risk factors for SRH with negative outcomes, such as STI´s and unintended pregnancy (Asare, 2015; Kann et al., 2014).

The consistent use of condoms, during sexual intercourse provides substantial protection against STI´s (CDC, 2014). However many students do not use condoms consistently and correctly, especially those who drink heavily and take drugs (Adefuye et al., 2009; Gwon & Lee, 2016; Uecker, 2015). The failure of many students to recognize the degree of their risk for STI´s is also a significant obstacle for condom use and intervention strategies (Hickey & Cleland, 2013; Wilton et al., 2014).

The importance of risk and protective factors for youth SRH behaviour has been reviewed in the scientific literature. Some of most important group of risk and protective factors, because

they are modifiable, are of cognitive, psychosocial and personality nature, and includes knowledge, attitudes, self-efficacy, subjective norms and sensations seeking (IPPF, 2007; Kirby, Coyle, Alton, Rolleri & Robin, 2011; Lazarus, Sihvonen-Riemenschneider, Laukamm-Josten, Wong & Liljestrand, 2010; Zuckerman, 2007).

Knowledge provides the foundation for attitudes and is essential to reducing the sexual risks behaviours. Attitude, self-efficacy and subjective norms influence behavioural intention and has a strong association with protective behaviour, namely condom use (Albarracín, Johnson, Fishbein & Muellerleile, 2001; Asare, 2015, Guo, Lia, Owena, Wangb & Duncan, 2015) and condom negotiation skills (Gakumo, Moneyham, Enah & Childs, 2012). The sexual sensation seeking has been associated with higher number of sexual partners and unprotected sex (Gullette & Lyons 2006; Sales et al., 2013).

The assumption that knowledge of STIs and avoidance of risk is positively correlated with educational attainment is often inaccurate (Lewis, Miguez-Burbano & Malow, 2009). Some studies have shown the sexual risk behaviours remains in transition from adolescence to adulthood (Reis, Ramiro, Matos & Diniz, 2013). Identifying the risk and protective factors for youth's SRH behaviours and outcomes is important for develop and implement STI´s prevention, treatment and care policies, and programs for college students. Those interventions might be implemented through the universities health services, with the specific contribution of nursing care. The aim of this study was to identify the major risk and protective factors that influence sexual risk-taking behaviour in college students, to be considered in the design of preventive interventions for this population.

Methods

Design and sample

A cross-sectional study was conducted in a sample of 1946 students (64% female and 36% male), between the ages of 18 and 29 years, attending university in Portugal in the 2012/2013 academic year. The technique used for sample selection was a random sampling of cluster groups, in which the sample unit was the class. Classes were selected for their representation of the scientific areas of studies (Life sciences, health and human and social science) and for the different years of study. Students were recruited by direct solicitation in the classroom, and participation was voluntary. Data were collected in the classroom at the end of class through a completely anonymous self-administered survey. Information on anonymity and confidentiality was provided to the participants. After completing the survey, the students delivered the survey in a closed unidentified envelope.

The demographic questionnaire collected information on gender, age (≤19, 20-24, ≥25 years), nationality, importance of religion (high, moderate, low or null), area of studies, parental education level (9 years, 10 to 12 years, university) and monthly family income (<1000 US$; 1000-2000 US$; >2000 US$).

Four SRH risky behaviours were considered in the present study: (1) having sexual intercourse without using a condom in the last 12 months, (2) having sex while under the influence of alcohol or (3) drugs and (4) having sex with casual partners. Additionally, information on previous sexual behaviour was collected: age at first sexual intercourse, condom use in first sexual relationship, whether they had had sex in the context of a committed relationship in the last twelve months and if they had ever had a STI.

Measures

Knowledge about IST`s was measured using an inventory-based self-report questionnaire made up of 16 items, developed for the present study, to determine the knowledge about diseases and risks associated to STI´s. Items were rated using three options for an answer: true, false and don’t know. Correct answers were coded as one, and incorrect or uncertain responses were coded as zero. Knowledge’s reliability was assessed using Kuder-Richardson’s formula 20 (KR20) used as a measure of internal consistency in the studies with dichotomous scales. The result was KR20=0.78, confirming the internal consistency of the instrument as adequate.

Attitudes towards SRH were measured using a scale composed of 20 items with, designed specifically for the study. The scale had seven even response options (1 - *strongly disagree* to 7 - *strongly agree*), which assessed five theoretical dimensions of SRH attitude (responsible sexuality, preventive attitude, attitude towards STI´s, attitude towards condoms and hedonism). The scale revealed good internal consistency (α=0.82).

Subjective norms were measured with three questions to assess peer`s perceptions about condom use, sex with alcohol and drugs, and sex with occasional partners. The items were scored using a seven-point scale ranging from 1- *rarely* to 7-f*requently*. These three items were combined to provide a direct assessment of subjective norms. In this sample the Cronbach’s alpha was 0.79.

Self-efficacy for condom use was measured with a reduced version of the Condom Use Self-Efficacy Scale (CUSES – Brien, Thombs, Mahoney & Wallnau, 1994). The Portuguese college students’ version of the reduced version of CUSES (CUSES-RP) comprises 15 items measured on a five-point scale (0 - *strongly disagree to* 4 - *completely agree*). This scale has a multidimensional structure with four factors (mechanisms, partner’s disapproval,

assertiveness and intoxicants). In this sample, the CUSES-RP had a good Cronbach's alpha (α=0.86), similar to the internal consistency of the original scale (α=0.85).

The *Sexual Sensation Seeking* Scale (SSSS – Kalichman & Rompa, 1995) has been used to measure the propensity to pursue high levels of sexual arousal and to engage in novel sexual risky experiences. The SSSS includes 11 items with a rating scale from 1 (*never applies to me*) to 4 (*often applies to me*). The Portuguese college students' version of SSSS-P comprises 9 items (unpublished results). The scale's internal consistency was good (α=0.83) in comparison to that reported by authors (α=0.83 for men, α=0.81 for women).

Interpretation of the scores of STI knowledge, SRH attitudes, subjective norms and self-efficacy: the higher is the score, the higher is the factor, and is conceptually associated to a health-promotion behavior. In the SSSS, high scores correspond to a health-compromising behaviour. Score ranges are indicated in Table 2.

Ethical considerations

The study was approved by the National Data Protection Committee (Nº 7409/2013) and was sanctioned by the Ethics Committee (Nº 2/2012) of the University where the study was conducted.

Data analysis

Data were analyzed using SPSS software (version 22.0, 2012; SPSS, Inc, Chicago, IL, USA). The associations between sexual risk behaviour and contributing individual and environmental factors were studied using Chi-square test. The associations between the cognitive and psychosocial variables and the four sexual risk behaviours were analyzed using analyze of variance (ANOVA). Logistic regression was used to identify risk behaviour predictors, from the cognitive and psychosocial factors studied. Models were adjusted for gender and age. Statistical significance was set at a p value of less than 0.05.

Results

Demographic characteristics and sexual behaviours

Of the students surveyed, 64% were female, the average age was 20.7±2.3 years and the majority was Portuguese (97.3%). About two-thirds (63.4%) were students in life sciences courses. Their household income was generally low (57% < 1000 US$/month). Their parents' level of education was also low, as 54.5% of the mothers and 61.6% of the fathers only had nine years of education. Approximately half of the students (50.8%) consider religion important. The average age at first sexual intercourse was 17±1.8 years, and 90.8% of the

students used a condom the first time they had intercourse. Eighty-nine percent of the students who had sex in the last 12 months were engaged in committed relationships. The number of students who reported having had an STI was low (1.3% had had an infection, and 3.8% were not sure). Only 14.5% of students were tested for HIV/AIDS.

Individual and environmental factors affecting sexual risk behaviour

Having intercourse without a condom, under the influence of alcohol, drugs or with casual partners was reported by 60.5%, 33.0%, 9.7 % and 32.0%, respectively. The relationship between the individual and environmental factors and the frequency of the SRH behaviours is presented in Table 1. The education of the parents was omitted, as they showed generally the same trend as that observed for family income. No association was observed between the area of studies (life sciences-related or other areas) and the risky behaviours studied. No differences were observed in condom use between both genders. More than half (51.8%) of the men reported having sex under the influence of alcohol, while the corresponding prevalence in women was only 21.4%. The frequency of having sex under the influence of drugs was lower than that observed for the use of alcohol. Having sex with casual partners was clearly more frequent ($p<0.001$) among men (53.8%) than women (18.5%).Younger students were less engaged ($p<0.001$) to all the risky sexual behaviours surveyed. Students from families with higher income were more exposed to the risk of having sex after alcohol ($p<0.001$) or drugs ($p=0.005$) consumption and with casual partners ($p=0.001$).

Considering religion to be important was associated with more protective behaviours. The students who used a condom the first time they had sex were also those who reported using it more consistently. When sexual activity occurred within a committed relationship, the frequency of condom use, having sex after consuming alcohol or having sex while taking drugs were all less frequent. Among students involved in a relationship, 26.1% indicated that they had also had sex with casual partners.

Cognitive and psychosocial factors predicting sexual risk behaviour

The influence of cognitive and psychosocial factors on the risk-taking behaviours are presented in Table 2. Significant associations were further used to predict risk behaviour through logistic regression (Table 3). Students surveyed in this study had an average knowledge on STI´s, with a mean score of 9.05±2.73. The only risk behaviour associated to the students' knowledge was the combination of sex with drugs ($p=0.010$). Students had a positive attitude towards SRH (118.28±13.92). All four risk behaviours studied were associated ($p<0.001$) with students who had a lower score on SRH attitude. The increase on SRH attitude reduced the odds (Table 3) of engagement in sex with casual partners ($OR=0.989, p=0.032$).

Table 1.
The influence of individual and environmental factors on the risk-taking behaviours of having sex without using a condom, having sex under the influence of alcohol or drugs and having had sex with casual partners over the last 12 months. Results are presented in percentage in rows pertaining to each of the risk behaviours

	Unprotected sex, no-condom			Sex with alcohol			Sex with drugs			Sex with casual partners		
Variables	**No**	**Yes**	**Chi p-value**	**No**	**Yes**	**Chi ^{2} p-value**	**No**	**Yes**	**Chi ^{2} p-value**	**No**	**Yes**	**Chi ^{2} p-value**
n (min-max)	590-591	906		998-1011	495-498		1347-1361	146-147		1011-1023	480-482	
**Gender**												
Female	39.9	60.1	0.173	78.6	21.4	148.243	95.2	4.8	63.346	81.5	18.5	201.955
Male	38.8	61.2	(0.359)	48.2	**51.8**	(<0.001)	82.3	**17.7**	(<0.001)	46.2	**53.8**	(<0.001)
**Age**												
≤ 19	**50.6**	**49.4**	32.116	**75.2**	**24.8**	18.534	91.9	8.1	2.096	75.5	24.5	34.136
20-24	35.9	64.1	(<0.001)	64.2	35.8	(<0.001)	89.8	10.2	(0.351)	67.4	32.6	(<0.001)
≥ 25	29.3	70.7		60.6	39.4		88.2	11.8		**48.0**	**52.0**	
**Family income**												
< 1000 US$	40.5	59.5	1.246	**73.0**	**27.0**	40.170	91.7	8.3	6.0089	71.5	28.5	13.852
100-2000 US$	37.3	62.7	(0.536)	64.0	36.07	(<0.001)	89.4	10.6	(0.050)	66.0	34.0	(0.001)
> 2000 US$	40.2	59.8		*51.5*	*48.5*		86.6	13.4		59.1	**40.9**	
**Importance of religion**												
Low/Null	**29.9**	70.1	9.026	54.9	**45.1**	29.910	88.2	11.8	13.621	57.9	**42.1**	24.537
Moderate	38.4	61.6	(0.011)	62.4	**37.6**	(<0.001)	87.4	**12.6**	0.001	63.5	**36.5**	(<0.001)
High	42.5	57.5		**73.5**	**26.5**		93.2	**6.8**		73.9	**26.1**	
**Condom usage in first sexual relation**												
No	**33.2**	66.8	7.748	74.9	25.1	13.369	89.8	10.2	0.061	77.3	22.7	19.189
Yes	41.5	58.5	(0.003)	64.3	35.7	(<0.001)	68.6	7.3	(0.804)	64.8	35.2	(<0.001)
**Sex in committed relationship**												
No	**54.0**	46.0	16.099	33.1	66.9	90.246	81.3	18.8	15.228	17.5	82.5	205.222
Yes	37.7	62.3	(<0.001)	70.9	29.1	(<0.001)	91.3	8.7	(<0.001)	73.9	26.1	(<0.001)

n= Number; Chi ^{2}= Chi-square test
Bold frequencies correspond to a standardized residual ≥1.96 absolute value

Attitude is a poor predictor for having sex without a condom (OR=0.991, p=0.086) and for combining sex with alcohol (OR=0.993, p=0.240) or drugs (OR=0.998, p=0.801). Students considered others' opinions about sexual risk behaviour, as revealed by the high scores on subjective norms (16.58±3.37). This construct is one of the best predictors of protective behaviour concerning condom use (OR=0.954, p=0.012) and of having sex after consuming alcohol (OR=0.993, p=0.001) or drugs (OR=0.888, p<0.001). The self-efficacy for condom use was generally high (47.75±8.97), independently of the risk behaviour of the students. The sexual sensation seeking is an excellent predictor of sexual risk behaviour.

Discussion

The prevalence of condom use was similar for both men and women. Men were more likely to combine sex with alcohol or with drugs and were much more involved in sex with casual partners. These behaviours are intrinsically related to the nature of the male gender, in which hedonism and sensation seeking play a stronger role (Gullette & Lyons, 2006), but also to the social pressure exerted by the peers (Ghandour, Mouhanna, Yasmine & El Kak, 2014). On the contrary, young women generally receive more messages promoting abstinence, traditional sex roles, and sex within a relationship context (Tung, Hu, Efird, Su & Yu, 2013).

The older students were less likely to report condom use, and more likely to have sex with casual partner than the younger students. This trend might be associated to a less integration on social life and to the eventual sexual and psychological immaturity of some of the younger students (Uecker, 2015). Another hypothesis that could be considered to explain the differences observed is that only younger students received sex education in high school. A law modification was introduced in Portugal after 2009 that led to mandatory sex education, resulting in younger students having a different sexual education background than older students. The higher the family's income, the higher were the odds that the student reported having risky behaviours, probably due to the higher purchasing power, which allows these students to have a social life with more opportunities for consuming alcohol and/or drugs (Berhan & Berhan, 2015). The religion is commonly regarded as a protective factor against sexual risk-taking behaviours (Ghandour et al., 2014). Accordingly, students who valued religion were less exposed to sexual risk. Previous behaviours are considered excellent predictors of actual behaviours (Albarracín et al., 2001). In the present study, using a condom at the first sexual intercourse was observed to predict the actual use of condoms. For sex behaviours in the context of a committed relationship this study's findings are worrying because condom use was considerably lower for those involved in a relationship, ranging from 54.0% for those not committed to 37.7% for those who are.

Table 2.
The influence of cognitive and psychosocial factors on the risk-taking behaviours of having unprotected sex without a condom, having sex under the influence of alcohol or drugs, having had sex with casual partners over the last 12 months. Results are presented in mean (standard deviation)

Variables	Unprotected sex, no-condom			Sex with alcohol			Sex with drugs			Sex with casual partners		
	No	Yes	F p-value	No	Yes	F p- value	No	Yes	F p-value	No	Yes	F p-value
n (min-max)	571-591	888-906		978-1011	489-498		1325-1361	142-147		993-1023	472-482	
**STIs' Knowledge** (0-16)$^{\Psi}$	9.00 (2.75)	9.09 (2.77)	0.334 (0.563)	9.03 (2.77)	9.03 (2.76)	0.010 (0.971)	9.09 (2.75)	8.47 (2.92)	6.647 (0.010)	9.08 (2.76)	8.91 (2.77)	1.295 (0.255)
**SRH Attitude** (20-140)$^{\Psi}$	119.68 (14.51)	117.29 (13.85)	10.288 (0.001)	120.19 (13.25)	114.14 (15.01)	63.652 (<0.001)	118.87 (13.80)	110.99 (15.27)	43.445 (<0.001)	120.27 (13.13)	113.77 (15.21)	72.428 (<0.001)
**Subjective Norms** (3-21)$^{\Psi}$	17.05 (3.08)	16.26 (3.51)	19.463 (<0.001)	17.00 (3.23)	15.66 (3.46)	53.955 (<0.001)	16.77 (3.31)	14.56 (3.25)	55.297 (<0.001)	16.87 (3.31)	15.92 (3.42)	25.670 (<0.001)
**Condom Use Self-Efficacy** (0-60)$^{\Psi}$	49.38 (8.18)	48.51 (9.02)	3.483 (0.052)	49.19 (8.41)	48.06 (9.52)	5.507 (0.019)	49.06 (8.47)	46.57 (11.30)	10.679 (0.001)	49.13 (8.51)	40.13 (9.41)	4.292 (0.038)
**Sexual Sensations Seeking** (9-36)$^{\Psi}$	18.67 (5.27)	19.46 (5.23)	8.080 (0.005)	17.73 (4.85)	22.05 (4.89)	261.783 (<0.001)	18.67 (5.04)	23.55 (5.31)	122.848 (<0.001)	17.74 (4.84)	22.15 (4.89)	268.565 (<0.001)

n= Number; min= Minimum; max= maximum; $^{\Psi}$ Minimum and maximum scores of the scale

Table 3.
Logistic regression models obtained for cognitive and psychosocial factors predict risk-taking behaviours of having sex without using a condom, having sex under the influence of alcohol or drugs and having had sex with casual partners over the last 12 months

Predictors	Unprotected sex, no-condom			Sex with alcohol			Sex with drugs			Sex with casual partners		
	OR	95% CI	p-value	OR	95% CI	p-value	OR	95% CI	p-value	OR	95% CI	p-value
**Knowledge STIs** (0-16)$^{\Psi}$							0.990	0.922-1.036	0.789			
**SSR Attitude** (20-140)$^{\Psi}$	0.991	0.981-1.001	0.086	0.993	0.983-1.004	0.240	0.998	0.982-1,014	0.804	0.988	0.977-0.999	0.032
**Subjective Norms** (3-21)$^{\Psi}$	0.954	0.920-0.990	0.012	0.933	0.896-0.971	0.001	0.888	0.837-0.941	<0.001	0.992	0.952-1.023	0.688
**Condom Use Self-Efficacy** (0-60)$^{\Psi}$	0.998	0.984-1.013	0.832	1.008	0.992-1.024	0.354	1.002	0.977-1.027	0.905	1.007	0.990-1.023	0.440
**Sexual Sensation Seeking** (9-36)$^{\Psi}$	1.028	1.005-1.052	0.025	1.146	1.114-1.176	<0.001	1.141	1.015-1.189	<0.001	1.144	1.113-1.176	<0.001
**Ommibus Test** Chi^2 (p-value)	59.7 (<0.001)			293.3 (<0.001)			139.8 (<0.001)			320.2 (<0.001)		
**Hosmer and Lemeshow Test** Chi^2 (p-value)	6.05 (0.641)			9.46 (0.305)			3.24 (0.918)			12.28 (0.139)		

$^{\Psi}$ Minimum and maximum score of scale; OR= Odds ratio; CI= Confidence interval; Chi^2=Chi-square; Models were adjusted for gender and age

This indicates that condoms are used mainly to prevent pregnancy and that they are replaced by oral contraception and concerns about STI are reduced. Goldstein, Upadhyay and Raine (2013) observed that after initiating a contraceptive pill, condom use in young women (15-24 years old) decreased by 9%. The main barrier to using condoms in committed relationships is that there is a great trust not only in the partner, namely in his/her sexual behaviour in the past, but also in the faithfulness associated with a seriousness relationship (Tung et al., 2010). Consistent with previous studies (Gullette & Lyons, 2006; Sabone et al., 2007), the results indicate that individuals involved in a committed relationship also have sex with other partners and condom use is more common in casual sexual encounters. Of those in a stable relationship, 26.1% indicated that they had had sex with a casual partner while committed. Encouraging individuals to use double contraception methods (e.g., pill plus condom) is one of the main aspects that should be considered in nursing interventions to help students protect themselves from STI (IPPF, 2007).

Cognitive and psychosocial factors associated with risk behaviour

The decision to have sex involves a balance between the expected pleasure, arousal and eventually emotional involvement and the assessment of the risk of an STI or pregnancy. Students are more worried about contraception than STI, as most of them abandoned the use of condoms when they began oral contraception, apparently based in a strong confidence in the partners' sexual history and monogamy. Given that the risk assessment involved in deciding whether to have sex and the protective measures to take are based on previous knowledge, it would be expected that those students with higher STI knowledge would make safer choices, more specifically through having a more positive attitude towards safer sex (Kirby et al., 2011). In the present study, attitude was significantly correlated with knowledge, but knowledge was unrelated to the risk-taking behaviours. Knowledge of health risks alone does not predict safe sex practices (Kabwama & Berg-Beckhoff, 2015). Biases in judgment such as excessive optimism, psychological distancing and overconfidence also explain how knowledge may fail to predict risk perception (Adefuye et al., 2009).

Attitude is one of the most important theoretical constructs affecting health behaviour (Asare, 2015). Reis et al. (2013) observed that college students who had more positive attitudes tended not to accept sexual risks. Despite the differences observed in the attitude scores (Table 2), with higher scores corresponding to a more protective behaviour, when attitude was considered together with the other predictors, no significant predictive ability was found for the behaviours of having sex without a condom, with alcohol or with drugs (Table 3).

The perceptions young people have about their peers' rules regarding sexual activity, condom or contraception use, and alcohol and drug consumption affect their own behaviour

(Adefuye et al., 2009; Kirby et al., 2011). All the risk behaviours reported in this study were considerably influenced by the peer norms, which reflect the interpretations one makes based on others' judgement. The immediate social environment of the college campus and the larger cultural context, transmitted through the media, contribute to shape student's sexual behaviour (Fielder & Carey, 2010). Though self-efficacy for condom use is currently noted as a good predictor of sexual risk (Guo et al., 2015), our results of the logistic model indicate no predictive ability. That can arise from the high scores observed in this scale for most of the students, with little differences among groups. Moreover, most students had sex in the context of a committed relationship and abandoned the condom use.

Several studies indicate SSSS to be an excellent predictor of engaging in risk behaviours, specifically those increasing the risk of HIV/AIDS like unprotected sex, casual sex and having multiple sexual partners (Gullette & Lyons, 2006). In the present study, the students' SSSS-P score was found to be the best predictor for engagement in sexual risky behaviours. The results reflect the changes that have existed in recent decades in intimate relationships. Young people manifest more permissive attitudes toward casual sex without commitment, accepting diverse partners and value the erotic and hedonistic dimension of sexuality (Sales et al., 2013).

Conclusions

The results of the present study allow us to identify two groups of students at a higher risk of having sexual practices that increases their exposure to STI and pregnancy: older male students and students involved in a commitment relationship that replaced the condom use by contraceptive pills. The cognitive and psychosocial constructs studied showed different abilities to predict sexual risky behaviour. We observed that knowledge, attitude and self-efficacy have a limited ability to predict SRH behaviours. Hedonism is the main determinant for engaging in risky practices, as inferred by the high predictive ability of SSSS- P as well as by the higher risk exposure observed of older male students.

Implications for nursing and health policies

The results of the present study confirm that university students are at risk of infection with STIs, as they frequently engaged in risky practices and have a reduced awareness about the risk they face. There are several factors that increase the risk exposure that should be incorporated in SRH promoting programs by adapting the health services to the specific needs of this population and strengthening education in this area. Both, general health care services and university administrations should adopt measures to improve the reorganization of these services to young adults and to increase the awareness about STI and SRH regular check-ups.

Promotion of SRH care is included in nursing care competencies namely through the evaluation of the sexual history, elucidation students' doubts and encouragement to the adoption of health-promoting behaviours viewing the reduction of the odds of negative health outcomes. This education must be provided within the scope of primary health services, in family planning, and reinforced by the nurses in the health care services provided by most universities. These university nursing services are very important to provide to students because of their proximity, open-minded and non-moralistic environment. Nurses that are requested to clarify sexual issues or identify high risk students should help students evaluate the potential sexual risks they face while considering that students who are male, older, and involved in committed relationships are all at higher risk. Students must be clearly educated on the serious health complications that result from STIs, particularly those related to women's reproductive health. Suspicious cases of higher risk students should be tested for most prevalent STIs in this age group. Significant effort should be made to increase students' awareness to avoid infecting other people.

Concerning the availability of health services, we believe that there is a gap between the perceived availability and reality of services. It is necessary to improve the divulgation of existing services in the university, that are unknown by most of the students and have a reduced utilisation. While men are more exposed to risk, most health services are organized for women and associated with gynaecological care or family planning services. As such, these services are frequently named "women's health services" that may not be considered appropriate for men's needs. Health policies should consider the (re)organization of these services, creating "sexual and reproductive health services", that may be more appealing and "friendly" while offering more flexible service schedules that also adapt nursing care to the student's gender.

Educational strategies that incorporate the findings of this study into behaviour change interventions (i.e., through sexual health peer education programs) should be developed by nursing schools in conjunction with SRH services at universities. Due to the strong influence of peers, interventions targeting student communities may shape social pressures that promote more protective health behaviours. To increase self-perceptions of sexual risk workshops for freshmen during the orientation week within the university are recommend. Attempts to change the perception that young adults have regarding condom use may also be useful, namely, promoting the hedonic potential of its use. Considering the universal use of mobile phones among youth, using existing university applications to disseminate short messages about sexual protection should be implemented, particularly in periods of academic parties. University students should be involved in the development STI prevention policies and programmes to promote effective results that meet their needs. Nurses can lead

SRH promotion programs at universities, promoting the use of condoms as a matter of social responsibility and citizenship, and raising awareness of the risks associated with STI.

Limitations

This study was conducted in one university with a convenience sample, limiting the generalization of the results. However, the students came from various regions of the country and the classes were selected randomly. The sexual behavior measures were self-reported and may not have been an accurate measure of actual behaviour. Social desirability may have been an issue because of the sensitive nature of the survey items.

References

Adefuye, A. S., Abiona, T. C., Balogun, J. A., & Lukobo-Durrell, M. (2009). HIV sexual risk behaviors and perception of risk among college students: Implications for planning interventions. *BMC Public Health*, *9*, 281. doi:10.1186/1471-2458-9-281

Albarracín, D., Johnson, B. T., Fishbein, M., & Muellerleile, P. A. (2001). Theories of reasoned action and planned behavior as models of condom use: A meta-analysis. *Psychological Bulletin, 127*(1), 142-161.

Asare, M. (2015). Using the theory of planned behavior to determine the condom use behavior among college students. *American Journal of Health Studies*, *30*(1), 43-50.

Authors omitted for anonymity. (in press). Adaptação portuguesa e validação da versão reduzida da Escala de Autoeficácia para Usar o Preservativo (CUSES). *Revista Internacional de Andrologia.* doi:10.1016/j-Androl.2016.06.002. (accepted for publication)

Berhan, Y., & Berhan, A. (2015). A meta-analysis of risky sexual behaviour among male youth in developing countries. *AIDS Research and Treatment*, 1-9. doi.org/10.1155/2015/580961

Brien, T. M., Thombs, D. L., Mahoney, C. A., & Wallnau, L., (1994). Dimensions of self-efficacy among three distinct groups of condom users. *Journal American College Health, 42*(4), 167-174. doi:10.1080/07448481.1994.9939665

Burnett, A. J., Sabato, T. M., Wagner, L., & Smith, A. (2014). The influence of attributional style on substance use and risky sexual behaviour among college students. *College Student Journal, 48*(2), 325-336.

Centers for Disease Control and Prevention. (2014). *Sexually transmitted disease surveillance 2013*. Atlanta, Georgia: Division of STD Prevention.

Claxton, S. E., & van Dulman, M. H. M. (2015). *The oxford handbook of emerging adulthood.* New York: Oxford University Press.

European Centre for Disease Prevention and Control, & World Health Organization Regional Office for Europe. (2014). *HIV/AIDS surveillance in Europe 2013*. Stockholm. Retrieved from http://ecdc.europa.eu/en/publications/publications/hiv-aids-surveillance-report-europe-2013.pdf

Fielder, R. L., & Carey, M. P. (2010). Predictors and consequences of sexual “hookups” among college students: A short-term prospective study. *Archives of Sexual Behavior*, *39*(5), 1105-1119. doi.org/10.1007/s10508-008-9448-4

Gakumo, C. A., Moneyham, L. D., Enah, C. C., & Childs, G. D. (2012). The moderating effect of sexual pressure on young urban women's condom use. *Research Nursing Health, 35*(1), 4-14. doi:10.1002/nur.20465

Ghandour, L. A., Mouhanna, L., Yasmine, R., & El Kak, F. (2014). Factors associated with alcohol and/or drug use at sexual debut among sexually active university students: Cross-sectional findings from Lebanon. *BMC Public Health, 14*, 671-681. doi:10.1186/1471-2458-14-671

Goldstein, R. L., Upadhyay, U. D., & Raine, T. R. (2013). With Pills, Patches, Rings, and Shots: who still uses condoms? A longitudinal cohort study. *The Journal of Adolescent Health : Official Publication of the Society for Adolescent Medicine*, *52*(1), 77-82. doi:10.1016/j.jadohealth.2012.08.001

Gullette, D. L., & Lyons, M. A. (2006). Sensation seeking, self-esteem, and unprotected sex in college students. *Journal of Association of Nursing in AIDS Care, 17*(5), 23-31. doi: 10.1016/j.jana.2006.07.001

Guo, G., Lia, Y., Owena, C., Wangb, H., & Duncan, G. J. (2015). A natural experiment of peer influences on youth alcohol use. *Social Science Research, 52*, 193-207. doi: 10.1016/j.ssresearch.2015.01.002

Gwon, S.H., &. Lee, C. Y. (2016). Factors influencing sexually transmitted infections among adolescents in South Korea. *Internacional Nursing Review*, *63*(1), 68-77. doi: 10.1111/inr.12206

Hickey, M. T., & Cleland, C. (2013). Sexually transmitted infection risk perception among female college students. *Journal American Association of Nurse Practitioners*, *25*(7), 377-384. doi:10.1111/j.1745-7599.2012.00791.x

IPPF European Network. (2007). Sexual and reproductive health & rights of young people in europe. The SAFE Project, Brussels: IPPF EN.

Kabwama, S. N., & Berg-Beckhoff, G. (2015). The association between HIV/AIDS related knowledge and perception of risk for infection: A systematic review. *Perspectives in Public Health, 135*(6), 299-308. doi:10.1177/1757913915595831

Kalichman, S. C., & Rompa, D. (1995). Sexual sensation seeking and sexual compulsivity scales: Reliability, validity, and predicting HIV risk behavior. *Journal of Personality Assessment, 65*(3), 586-601. doi:10.1207/s15327752jpa6503_16

Kann, L., Kinchen, S., Shanklin, S. L., Flint, K. H., Kawkins, J., Harris, W. A.., … Centers for Disease Control and Prevention. (2014). Youth risk behavior surveillance - United States, 2013. *MMWR, 63*(4), 1-168.

Kirby, D., Coyle, K., Alton, F., Rolleri, L., & Robin, L. (2011). *Reducing adolescent sexual risk. Theoretical guide for developing and adapting curriculum-based programs*. California: ETR Associates.

Lazarus, J. V., Sihvonen-Riemenschneider, H., Laukamm-Josten, U., Wong, F., & Liljestrand, J. (2010). Systematic review of interventions to prevent spread of sexually

transmitted infections, including HIV, among young people in europe. *Croatian Medical Journal*, *51*(1), 74-84. doi:10.3325/cmj.2010.51.74

Lewis, J., Miguez-Burbano, M., & Malow, R. (2009). HIV risk behavior among college students in the United States. *College Student Journal*, *43*(2), 475-491.

Pista, A., Oliveira, C. F., Cunha, M. J., Paixão, M. T., Real, O., & CLEOPATRE Portugal Study Group. (2011). Prevalence of human papillomavirus infection in women in Portugal: The CLEOPATRE Portugal study. *International Journal of Gynecological Cancer, 21*(6), 1150-1158. doi:10.1097/IGC.0b013e31821dd3b2

Reis, M., Ramiro, L., Matos, M. G., & Diniz, J. A. (2013). Determinants influencing male condom use among university students in Portugal. *International Journal of Sexual Health, 25*(2), 115-27. doi.org/10.1080/19317611.2012.728554

Sabone, M., Ntsayagae, E., Brown, M. S., Seboni, N. M., Mogobe, K. D., & Sebego, M. (2007). Perceptions of undergraduate students not participating in HIV/AIDS prevention activities in Botswana. *International Nursing Review, 54*(4), 332-338. doi:10.1111/j.1466-7657.2007.00544.x

Sales, J. M., Smearman, E. L., Brody, G. H., Milhausen, R., Philibert, R. A., & Diclemente, R. J. (2013). Factors associated with sexual arousal, sexual sensation seeking and sexual satisfaction among female. *African American adolescents. Sexual Health, 10*(6), 512-521. doi:10.1071/SH13005

Singh, S., Darroch, J. E., & Ashford, L. S. (2014). *The costs and benefits of investing in sexual and reproductive health 2014*. Guttmacher Institute and UNFPA.

Tung W-C., Hu, J., Efird, J. T., Su, W., & Yu, L. (2013). HIV knowledge and condom intention among sexually abstinent Chinese students. *International Nursing Review, 60*(3), 366-373. doi:10.1111/inr.12039

Uecker, J. E. (2015). Social context and sexual intercourse among first-year students at selective colleges and universities in the United States. *Social Science Research, 52*, 59-71. doi:10.1016/j.ssresearch.2015.01.005

Wicki, M., Kuntsche, E., & Gmel, G. (2010). Drinking at european universities? A review of students' alcohol use. *Addictive Behaviors, 35*(11), 913-924. doi:10.1016/j.addbeh.2010.06.015

Wilton, L., Palmer, R. T., & Maramba, L. C. (2014). *Understanding HIV and STIs prevention for college students*. London: Routledge Research in Higher Education.

Zuckerman, M. (2007). *Sensation seeking and risky behavior*. Washington, DC: American Psychological Association.

CAPITULO 4

ESTUDOS EMPÍRICOS EM SAÚDE SEXUAL E REPRODUTIVA

4.4. Artigo 4 - Barreiras no acesso dos jovens adultos aos cuidados de saúde sexual e reprodutiva

Santos, M.J., Ferreira, E., & Ferreira, M. (2015)

Revista da Associação Portuguesa de Enfermeiros Obstetras, *15* (6), 4-8.

BARREIRAS NO ACESSO DOS JOVENS ADULTOS AOS CUIDADOS DE SAÚDE SEXUAL E REPRODUTIVA

BARRIERS IN ACCESSING SEXUAL AND REPRODUCTIVE HEALTH CARE OF YOUNG PEOPLE

BERRERAS EN EL ACCESO DE LOS ADULTOS JÓVENES A LA ATENCIÓN DE LA SALUD SEXUAL Y REPRODUCTIVA

Maria José Santos[1]; Elisabete Ferreira[2]; Manuela Ferreira[3]

[1] Escola Superior de Enfermagem de Vila Real, Universidade de Trás-os-Montes e Alto Douro, Vila Real, Portugal. mjsantos@utad.pt

[2] Faculdade de Psicologia e Ciências da Educação, Universidade do Porto, Porto, Portugal.

[3] Escola Superior de Saúde de Viseu, Instituto Politécnico de Viseu, Viseu, Portugal.

Resumo

Introdução: Continuam a existir barreiras no acesso aos serviços e cuidados de saúde sexual e reprodutiva (SSR) por parte dos jovens, relacionadas com a inexistência de serviços específicos, rigidez de horários, localização inadequada, aspetos administrativos, sociais, culturais e religiosos.

Objetivos: O presente estudo pretende caracterizar os comportamentos de procura dos serviços de SSR por parte dos estudantes do ensino superior e identificar barreiras no acesso a esses serviços.

Metodologia: Foi realizado um estudo descritivo, correlacional de natureza quantitativa, numa amostra não aleatória de 1946 estudantes universitários. Na recolha de dados foi utilizado um questionário de autopreenchimento aplicado em sala de aula e foram asseguradas as questões de anonimato e confidencialidade. Os dados foram analisados por estatística descritiva e inferencial (qui-quadrado).

Resultados: Dos 1964 participantes, uma elevada percentagem (69,9%) nunca recorreu aos serviços de SSR. As dificuldades no acesso estão relacionadas com os horários (10,8%) e a demora na marcação das consultas (8,7%). Para aumentar a utilização dos serviços de SSR, os estudantes propõem como estratégias uma maior divulgação destes serviços (70,6%) e a garantia da confidencialidade (23,4%).

Conclusões: É necessário investir na divulgação dos serviços existentes no contexto universitário e na sensibilização dos estudantes, em particular dos rapazes, para a

importância da vigilância da SSR. Para diminuir as barreiras no acesso, os serviços devem estar localizados no campus universitário e os profissionais de saúde devem ser capazes de transmitir informação com recurso a estratégias mais inovadoras.

Palavras-chave: Acessibilidade, cuidados de saúde, enfermagem, saúde sexual e reprodutiva.

Abstract

Introduction: There are still barriers in the access to services and sexual and reproductive health (SRH) of young people, related to the lack of specific services, rigidity of schedules, inadequate location, and administrative, social, cultural and religious barriers.

Objectives: This study aims to characterize the behavior of demand for SRH services by university students and identify barriers to access.

Methodology: A descriptive correlational and quantitative study was conducted, not a random sample of 1946 college students. In the data collection we used a self-report questionnaire in the classroom and ensured the anonymity and confidentiality. Data were analyzed using descriptive and inferential statistics (chi-square).

Results: A high percentage (69.9%) of the 1946 participants never used the SRH services. Difficulties in access are related to schedules (10.8%) and delay in the appointments (8.7%). To increase the use of SRH services as students propose strategies, wider dissemination of these services (70.6%) and the guarantee of confidentiality (23.4%).

Conclusions: It is necessary to improve the dissemination of the services in the university context, but also the awareness of students, particularly boys, to the importance of surveillance by SRH. In order to reduce barriers to access, services must be located on university campus and health professionals should be able to provide information using the most innovative strategies

Keywords: Accessibility, health care, nursing, sexual and reproductive health.

Resumen

Introducción: Siguen existiendo barreras en el acceso a los servicios y atención de salud sexual y reproductiva (SSR) de los jóvenes, relacionados a la ausencia de servicios específicos, rigidez de horarios, localización inadecuada, barreras administrativas, sociales, culturales y religiosas.

Objetivos: Este estudio pretende caracterizar os comportamientos de procura de los servicios de SSR de los estudiantes de enseñanza superior e identificación de barreras en el acceso.

Metodología: Se realizó un estudio descriptivo, correlacional, de naturaleza cuantitativa, con una muestra no aleatoria de 1946 estudiantes universitarios. En la recogida de datos se ha utilizado un cuestionario de autocompletar, aplicado en clases y aseguradas las cuestiones de anonimato y la confidencialidad de los datos. Los dados fueron analizados por estadística descriptiva y inferencial (chi-cuadrado).

Resultados: De los participantes, 82,2%, ha tenido sexo en el último año y utilizó métodos anticonceptivos (96,1%). Una gran proporción (69,9%), nunca ha utilizado los servicios de SSR. Las dificultades para el acceso están relacionadas a los tiempos (10,8%) Y demora de la cita con un médico (8,7%). Para aumentar el uso de los servicios de SSR os estudiantes proponen como estrategias una mayor difusión del servicios (70,6%) y garantía de confidencialidad (23,4%).

Conclusiones: Es necesario invertir en la difusión de los servicios en el contexto de la universidad, pero también la sensibilización de estudiantes, los hombres en especial, sobre la importancia de la vigilancia de la SSR. Para reducir las barreras de acceso, los servicios deberán estar localizados en el campus de la universidad y los profesionales de la salud deben ser capaces de dar información, utilizando estrategias más innovadoras.

Palabras-clave: Accesibilidad, atención sanitaria, enfermería, salud sexual y reproductiva.

Introdução

É atualmente reconhecido que “a forma como os jovens vivenciam a sua sexualidade e os relacionamentos, bem como, as escolhas que fazem em termos de saúde sexual e reprodutiva (SSR), têm um impacto considerável no futuro e nas suas vidas” (Associação para o Planeamento da Família [APF], 2010, p.10). O aumento das infeções sexualmente transmissíveis (IST), da gravidez indesejada e de outros riscos ligados à atividade sexual, fazem com que os jovens sejam considerados um grupo de intervenção prioritário em termos de SSR. O início da idade adulta tem sido descrito como uma fase de particular vulnerabilidade face aos riscos envolvidos nos comportamentos sexuais, resultado não só do desenvolvimento psicossexual, mas também do contexto académico, com oportunidades para atividades sexuais que envolvem potencial risco para a saúde, associadas ao consumo de álcool, drogas e a relações sexuais com parceiros ocasionais (Burnett et al., 2014; Cunha-Oliveira, Cunha-Oliveira, Pita & Massano-Cardoso, 2013; Eaton et al., 2012; Kuperberg & Padgett, 2015). É cada vez mais consensual que as instituições do ensino

superior podem assumir um papel importante na educação para a saúde e a cidadania, tendo em vista informar e capacitar os jovens para opções de vida promotoras de saúde (Reis, Matos, Simões & Diniz, 2011; Rocha, 2008).

Os cuidados a prestar em saúde reprodutiva devem ser constituídos por um conjunto diversificado de serviços e atividades que contribuam para a saúde e o bem-estar reprodutivos através da prevenção e da resolução de problemas, dando resposta adequada às necessidades específicas dos indivíduos, ao longo do ciclo de vida (Direção-Geral da saúde [DGS], 2008). Uma estratégia fundamental na promoção da saúde global dos jovens é a existência de serviços apropriados, economicamente acessíveis e integrados (Silva & Meneses, 2010), pelo que a disponibilização destes serviços dentro das universidades pode ser uma mais-valia Contudo, continuam a existir barreiras no acesso aos serviços e a cuidados de saúde reprodutiva, relacionados não só com a insuficiência de serviços específicos, rigidez de horários, localização inadequada mas também com aspetos administrativos, financeiros, sociais, culturais e mesmo religiosos (Vilar, 2012).

Atendendo a que o conhecimento e o acesso a serviços de saúde é um dos aspetos fundamentais para a adoção de comportamentos preventivos, com o presente estudo pretende-se caracterizar os comportamentos de procura dos serviços de SSR por parte dos estudantes do ensino superior e identificar potenciais barreiras no seu acesso.

Contextualização

A SSR nas suas diferentes dimensões emergiu como uma área prioritária a considerar nas políticas e programas de saúde que visam promover a saúde juvenil. Para uma vivência positiva da sexualidade dos jovens, é importante que se aposte na promoção da SSR, através da educação para a saúde para formar e desenvolver atitudes e competências nos jovens, mas também na existência de serviços apropriados, acessíveis e integrados numa resposta de saúde e educação abrangentes e de acordo com as suas necessidades (APF, 2010). No limiar da idade adulta, muitos jovens recebem mensagens contraditórias e confusas sobre sexualidade e género e continuam a demonstrar uma preparação pouco adequada para sua vida sexual, o que os deixa potencialmente vulneráveis a gravidez indesejada, IST, inclusive, o VIH e situações de coação e violência (UNESCO, 2010). As conclusões de um estudo recente de âmbito nacional sobre a SSR dos estudantes do ensino superior (Reis et al., 2011) evidenciam que os jovens universitários, em particular nas faixas etárias mais jovens (18-21 anos), revelem mais competências e comportamentos preventivos, mas existe ainda uma percentagem significativa de jovens em risco a frequentar as universidades.

Segundo Carvacho, Mello, Morais e Silva (2008), o conceito de acesso aos serviços de saúde pode ser sistematizado em quatro dimensões: acesso geográfico (quantidade, tipo e localização dos serviços); acesso económico (custos para obtenção dos serviços, transporte, farmácia ou laboratórios); acesso administrativo (normas e procedimentos burocráticos, horário de atendimento, tempo de espera); acesso a informação (conhecimento da oferta e a necessidade de os utilizar). Por sua vez, Bertrand e colaboradores (1995), apontam uma quinta dimensão: as barreiras de natureza psicossocial, onde podem ser incluídas as barreiras psicológicas e/ou culturais que levam os potenciais utilizadores a não procurar o serviço de saúde por medo, crenças religiosas, questões de género e problemas determinados pela "distância social", tais como as diferenças de idade, classe social e etnia entre prestadores e utilizadores.

O acesso dos jovens aos serviços e cuidados de SSR é amplamente discutido no âmbito do Projeto SAFE (*Sexual Awareness Europe*), onde se fez uma reflexão sobre os desafios e barreiras que os jovens têm de enfrentar no acesso a estes serviços. Considera-se que a idade aumenta a existência de barreiras no acesso aos serviços de saúde, como a falta de serviços específicos a esta faixa etária, a localização, os horários de funcionamento e a confidencialidade (Vilar, 2012). Das recomendações apresentadas para a minimização das barreiras identificadas destacam-se a disponibilização dos serviços tendencialmente gratuitos que garantam as questões de anonimato e confidencialidade, a necessidade de assegurar serviços inclusivos e em locais de fácil acessibilidade, a participação dos jovens na implementação e no desenvolvimento de espaços de atendimento específicos, bem como a formação adequada e regular da equipa de saúde multidisciplinar (APF, 2008). É ainda fundamental que os profissionais de saúde procurem excluir da prestação de cuidados em SSR as suas convicções religiosas e culturais, uma vez que a sua atitude mais conservadora pode tendencialmente desencorajar os jovens a procurar aconselhamento ou tratamento relacionados com a SSR (APF, 2008). Neste sentido, considera-se que o contributo do enfermeiro especialista em saúde materna e obstétrica é indispensável. No domínio das suas competências profissionais (Regulamento n.º 127/2011, de 18 de fevereiro) está previsto o cuidado às mulheres, inseridas na família e na comunidade, no âmbito da SSR. Porém, as recomendações atuais apontam para uma atenção especial sobre as necessidades dos jovens adultos, nomeadamente em contextos de proximidade, como os gabinetes de atendimento em contexto académico constituídos por equipas multidisciplinares. Esta atenção por parte dos profissionais de saúde justifica-se ainda porque se reconhece que o ambiente académico propicia, muitas vezes comportamentos de risco sexual (López Navarrete, 2008; Reis et al., 2011; Ribeiro & Fernandes, 2009), mas também porque se trata de um período de preparação para o papel social do adulto do

ponto de vista da família, da procriação, da profissão, com plenos direitos e responsabilidade (Sant'Anna, Carvalho, Bastos & Coates, 2008).

Para promover o processo de aprendizagem e de responsabilização na tomada de decisão e a adoção de comportamentos sexuais seguros, será determinante assegurar a existência de serviços apropriados, economicamente acessíveis e integrados, considerados como uma ferramenta fundamental na promoção da saúde global dos jovens (APF, 2010; Matos, Reis & Ramiro, 2009; Silva & Meneses, 2010).

Metodologia

Foi realizado um estudo transversal, descritivo-correlacional, de natureza quantitativa, numa amostra não aleatória de 1946 estudantes do ensino superior (64% raparigas e 36% rapazes), de diversas áreas de formação de uma universidade do norte do país. Na recolha de dados foi utilizado um questionário de autopreenchimento e que permitiu a caracterização sociodemográfica, académica, de comportamentos de SSR, práticas de vigilância de saúde e tipo de utilização dos serviços de SSR. O questionário foi aplicado em sala de aula a todos os estudantes que aceitaram participar no estudo, depois de terem conhecimento dos seus objetivos e depois de asseguradas as questões de anonimato e confidencialidade. O estudo foi autorizado pela Comissão Nacional de Proteção de Dados (Autorização nº 7409/2013) e obteve parecer favorável pela Comissão de Ética (Parecer nº 2/2012) da universidade onde foi realizado o estudo.

O tratamento estatístico dos dados foi efetuado através do programa *Statistical Package for the Social Sciences* (SPSS) na versão 22.0. Na análise de dados foi utilizada a estatística descritiva (medidas de tendência central e de dispersão) e inferencial (Qui-quadrado), para analisar as diferenças relativamente às variáveis em estudo. O valor de $p<0{,}05$ foi considerado como indicando significado estatístico.

Resultados[5]

Os participantes têm uma idade média de 21 anos (M=20,74; DP=2,32), a maioria é de nacionalidade portuguesa (97,3%), solteira (97,6%), com um rendimento médio familiar baixo (57% dos estudantes refere um rendimento familiar inferior ou igual a dois salários mínimos). No que respeita aos comportamentos sexuais, cerca de dois terços (77,2%) teve

[5] As proporções apresentadas para os aspetos relativos à contraceção e aos comportamentos de risco no presente artigo são diferentes das que constam dos artigos 4.2 "Comportamentos contracetivos de estudantes do ensino superior" e 4.3 "Risk and protective factors for portuguese college students' sexual and reproductive health". Esse facto deve-se a dois aspetos: (1) No presente artigo todas as proporções foram calculadas em relação ao total de participantes no estudo, enquanto nos dois suprarreferidos a maioria as proporções foram calculadas tendo por base os estudantes que tinha tido relações sexuais no último ano; (2) No presente artigo utilizaram-se diretamente as respostas dos estudantes, enquanto nos dois artigos supramencionados as variáveis relacionadas com o uso de preservativo foram ajustadas com uma questão de controlo que serviu para saber se o estudante utilizava o preservativo de forma consistente (sempre, frequentemente, raramente, nunca).

relações sexuais no último ano. As raparigas mencionaram mais frequentemente do que os rapazes que as relações sexuais aconteceram no contexto de uma relação amorosa (♀=94,7% vs ♂=80,2%), sendo a diferença significativa (p≤ 0,0001). Os estudantes utilizam contraceção de forma regular (♀=97,1% vs ♂=94,4%). Os rapazes privilegiam mais o uso do preservativo (62%) e as raparigas a dupla contraceção pílula/preservativo (46%). Os métodos contracetivos mais inovadores, como o anel vaginal ou o implante, são pouco utilizados. Mais de metade dos estudantes (57,3%), adquiriu o método contracetivo numa farmácia, embora as raparigas também o obtenham frequentemente no centro de saúde (♀=40,3% vs ♂=16,3%) e os rapazes em lojas e supermercados (♂=41,4% vs ♀=13,4), sendo estas diferenças estatisticamente significativas entre géneros (p≤0,0001). Cerca de um terço das estudantes (31,3 %) ou as companheiras, no caso dos rapazes (25,1%), refere já ter utilizado contraceção de emergência.

Os comportamentos de riscos avaliados pelas relações sexuais associados a substâncias psicoativas e sexo ocasional, são mais prevalentes nos rapazes que nas raparigas, sendo esta diferença significativa (p≤0,001). Os rapazes mencionam mais frequentemente relações sexuais associadas ao álcool (♂=24,1% vs ♀=6%), drogas (♂=7,3% vs ♀=1,1%) e parceiros ocasionais (♂=27,5% vs ♀=3%), do que as raparigas.

No que respeita à vigilância de saúde, a maioria (85,5%) nunca realizou o teste do VIH e apenas 66,9% das raparigas foram vacinadas para o vírus do papiloma humano (HPV - *Human Papiloma Vírus*), sendo que 6,4% desconhece mesmo essa necessidade. Uma grande maioria das raparigas (65,1%) nunca realizou citologia vaginal e das que realizam, 63,7% fazem o teste anualmente. Mais de metade das raparigas (56,6%) não faz autoexame da mama e cerca de metade dos rapazes (47,9%) não realiza o e o autoexame do testículo, e 32,2% desconhece a sua importância.

Uma elevada percentagem de estudantes (69,9%) nunca recorreu aos serviços de SSR, ou desconhece mesmo a sua existência (7,9%) e as raparigas procuram mais a esses serviços que os rapazes (♀=30,4% vs ♂=7,6%; p≤0,001). Apenas uma pequena percentagem de estudantes (26,4%) conhece os serviços de SSR disponíveis na universidade, observando-se também diferenças significativas entre rapazes e raparigas (♀=31,2% vs ♂=17,7%; p≤ 0,0001). Dos diferentes tipos de serviços de SSR existentes, ambos os géneros procuram preferencialmente o centro de saúde (70,6%), para aquisição de métodos contracetivos (56,5%), aconselhamento sobre contraceção (48,6%) e realização de citologia (27,1%). Os horários (10,8%) e localização pouco convenientes (4,0%) e a demora na marcação da consulta (8,7%), foram referidas como dificuldades no acesso aos serviços de SSR. Quando lhes foi solicitado que mencionassem medidas para promover a utilização dos serviços de SSR disponíveis, os estudantes apontaram como estratégias uma maior divulgação dos

serviços (70,6%), a garantia da confidencialidade e anonimato (23,4%) e a transmissão de informação de forma mais inovadora e com recurso às novas tecnologias (21,3%).

Discussão

Os resultados obtidos permitem afirmar que maioria dos estudantes são sexualmente ativos, mais de metade dos participantes tem um relacionamento amoroso atual e mantém relações sexuais com o companheiro, revelando na sua maioria preocupação com utilização de um método contracetivo nas relações sexuais com penetração. Na comparação de géneros verifica-se que há uma tendência para as raparigas iniciarem relações sexuais mais tarde e utilizarem mais a dupla contraceção (pílula e preservativo) comparativamente com os rapazes. Também constatamos que existe mudança no padrão de contraceção nas relações amorosas de longa duração, verificando-se que, ao aumentar a confiança no companheiro, deixa de existir preocupação com as IST e os estudantes passam a utilizar menos o preservativo como método de proteção. Estes resultados confirmam as tendências do último estudo publicado em Portugal sobre os comportamentos sexuais dos estudantes do ensino superior (Reis et al., 2011), no qual se observou que maioria dos jovens era sexualmente ativa (83,3%) e utilizava como método contracetivos preferencialmente a pílula (70,4%) e o preservativo (69%).

Na presente investigação também se observou que os métodos contracetivos como o anel vaginal, o adesivo contracetivo, o implante e o preservativo feminino são pouco utilizados pelos estudantes, embora a sua divulgação esteja recomendada, dado que permitem uma contraceção mais eficaz, colmatando os riscos associados ao uso inadequado das opções contracetivas mais tradicionais (Vilar & Ferreira, 2009).

Constatou-se ainda que os estudantes procuram pouco os serviços de SSR e que os rapazes recorrem menos do que as raparigas. Estas recorrem preferencialmente ao centro de saúde para obter métodos contracetivos. Também Martins Nunes, Muñoz-Silva e Sánchez-García (2008), observaram, numa amostra de jovens universitários (portugueses e espanhóis), que os profissionais de saúde são procurados essencialmente por raparigas e sobretudo pelas jovens de idade superior a 23 anos, pressupondo que o fazem por serem sexualmente mais ativas e com necessidade de métodos contracetivos de prescrição médica. No que se refere ao exercício da sexualidade, a evidência demonstra que os jovens continuam a privilegiar fontes de informações pouco fiáveis (Martins et al., 2008; Ribeiro & Fernandes, 2009; Sant'Anna et al., 2008), remetendo para frequências ainda reduzidas os serviços de SSR ou profissionais de saúde.

Granja (2009), afirma que para muitos jovens, os horários, localização ou os custos dos serviços tornam-nos inacessíveis, além de que as atitudes dos profissionais de saúde são desencorajadoras, dificultando o aceso dos jovens aos serviços para obterem informação, aconselhamento ou tratamento. No presente estudo foram igualmente estes os aspetos apontados como condicionantes no acesso. A garantia do anonimato e confidencialidade foi também uma das preocupações demonstradas pelos jovens e um fator que condicionou a procura dos serviços de SSR. O respeito pelo anonimato e a confidencialidade necessitam de ser assegurados pelos profissionais de saúde, uma vez que o seu incumprimento tem sido associado a uma menor utilização dos serviços de saúde por parte dos jovens e a maus resultados clínicos (Ott, Sucato & Committee on Adolescence, 2014).

As questões relacionadas com a vigilância de saúde também parecem preocupantes se considerarmos que os participantes são estudantes do ensino superior e alguns de áreas da saúde e afins, onde estas questões são abordadas nos planos curriculares dos seus cursos. Os dados revelam que pouco mais de metade das raparigas foi vacinada contra o HPV, apesar de a vacina estar integrada no plano nacional de vacinação desde 2008. Adicionalmente, uma grande maioria das estudantes nunca realizou citologia vaginal, apesar de ter vida sexual ativa há mais de três anos e realiza citologia anualmente. A Sociedade Portuguesa de Ginecologia (SPG, 2014) menciona que em programas de rastreio organizado as recomendações vão no sentido de se realizar citologia vaginal de três em três anos, entre os 25 e os 65 anos. Em rastreio oportunístico aconselha-se a realização de uma citologia de três em três anos, a partir dos 21 anos e/ou pelo menos três anos após início da atividade sexual.

Torna-se fundamental motivar os jovens para a prevenção e vigilância regular de saúde, nomeadamente a vacinação contra o HPV, que está indicada em raparigas e rapazes entre os 9-26 anos, mesmo após o início da atividade sexual, para prevenção de lesões genitais pré-malignas e cancro do colo do útero (SPG, 2014). Os estudantes apontam como estratégias para melhorar a adesão aos serviços de SSR uma maior divulgação e a utilização das novas tecnologias. Neste sentido, Reis e colaboradores (2011) referem que é essencial apostar em serviços de saúde nas Universidades, constituídos por equipas multidisciplinares com formação específica, com recurso à internet e inovação como as mHealth (SMS nos telemóveis).

Em síntese, salienta-se que aceder a informação de qualidade, educação e procura de aconselhamento e cuidados nos serviços de SSR disponíveis, pode conduzir a um aumento da prática de sexo seguro, a práticas contracetivas mais adequadas e a uma melhor perceção sobre a importância de uma vigilância regular da SSR

Conclusões

Os resultados evidenciam que existe uma subutilização dos serviços de SSR e pouca adesão a comportamentos de autovigilância de saúde por parte dos estudantes. A procura dos serviços de SSR é ainda limitada, pelo que é essencial a divulgação dos serviços existentes no contexto universitário, mas também a sensibilização dos estudantes, em particular dos rapazes, para a importância da vigilância da SSR e de adotar comportamentos de saúde mais responsáveis. Na opinião dos estudantes, as maiores dificuldades no acesso aos serviços e SSR estão relacionadas com a localização e horários de funcionamento, pelo que seria conveniente os serviços estarem localizados no campus universitário, com horários mais alargados e os profissionais de saúde terem particular atenção em assegurar as questões de anonimato e confidencialidade, bem como transmitirem informação com recurso a estratégias mais interativas, tais como o cinema, teatro, debate e novas tecnologias. A utilização de tecnologias de informação e comunicação podem assumir um papel privilegiado na implementação de abordagens inovadoras e apelativas na educação para a saúde.

Referências bibliográficas

Associação de Planeamento Familiar. (2008). *Manual de educação inter-pares em direitos & saúde sexual e reprodutiva.* Lisboa: Alfaprint.

Associação para o Planeamento da Família. (2010). *Direitos e saúde sexual e reprodutiva de jovens na Europa: Um guia para o desenvolvimento de políticas sobre direitos e saúde sexual e reprodutiva de jovens na Europa*. Lisboa: Federação Internacional de Planeamento Familiar.

Bertrand, J.T., Hardee, K., Magnani, R.J., Angle, M.A. (1995). Access, quality of care and medical barriers in family planning programs. *Internacional Family Planning Perspective*, *21*(2), 64-69. doi:10.2307/2133525

Burnett, A. J., Sabato, T. M., Walter, K. O., Kerr, D. L., Wagner, L., & Smith, A. (2014). The influence of attributional style on substance use and risky sexual behaviour among college students. *College Student Journal, 48*(2), 325-336.

Carvacho, I. E., Mello, M. B., Morais, S. S., & Silva, J. L. P. (2008). Factores associados ao acesso anterior à gestação a serviços de saúde para adolescentes gestantes. *Revista de Saúde Pública, 42*(5), 886-894. Retirado de http://www.scielo.br/pdf/rsp/v42n5/6062.pdf

Cunha-Oliveira, A., Cunha-Oliveira, J., Pita, J. R., & Massano-Cardoso, S. (2009). A aquisição do preservativo e o seu (não) uso pelos estudantes universitários. *Revista Referência*, *11* (Série 2), 7-22.

Direção-Geral da Saúde. (2008). *Saúde reprodutiva. Planeamento familiar. Programa nacional de saúde reprodutiva*. Lisboa: Autor.

Eaton, D., Kann, L., Kinchen, S., Ross, J., Hawkins, J., Harris, …Wechsler, H. (2012). *Youth risk behavior surveillance: United States, 2011*. Atlanta: Centers for Disease Control and Prevention.

Granja, P. M. (2009). Caracterização dos comportamentos sexuais dos adolescentes que frequentam o Olá Jovem. *Sexualidade & Planeamento Familiar, 52/53*, 48-54.

Kuperberg, A., & Padgett, J. E. (2015). Dating and hooking up in college: Meeting contexts, sex, and variation by gender, partner's gender, and class standing. *The Journal of Sex Research, 52*(5), 517-531. doi:10.1080/00224499.2014.901284

López Navarrete, G. E. (2008). Infección por vírus de papiloma humano. *Revista de la Facultad de Medicina, UNAM, 51*(6), 243-244.

Martins, M. T., Nunes, C., Muñoz-Silva, A., & Sánchez-García, M. (2008). Fontes de informação, conhecimentos e uso do preservativo em estudantes universitários do Algarve e de Huelva. *PSICO, 39*(1), 7-13. file:///C:/Users/User/Downloads/2820-11311-1-PB%20(2).pdf

Matos, M. G., Reis, M., & Ramiro. L. (2009). Educação sexual em Portugal e em vários países da América Latina. *Psicologia, Saúde & Doenças, 10*(1), 149-158.

Organização das Nações Unidas para a Educação, a Ciência e a Cultura. (2010). *Orientação técnica internacional sobre educação em sexualidade. Uma abordagem baseada nas evidências para escolas, professores e educadores em saúde*. Brasília: Divisão da Coordenação das prioridades da ONU em Educação, secção VIH/SIDA. Retirado de http://unesdoc.unesco.org/images/0018/001832/183281por.pdf

Ott, M. A., Sucato, G. S., & Committee on Adolescence. (2014). Contraception for adolescents. *Pediatrics, 134*(4), 1257-1281. doi:10.1542/peds.20142300

Regulamento n.º 127/2011, de 18 de fevereiro. Define o perfil das competências específicas do enfermeiro especialista em enfermagem de saúde materna, obstétrica e ginecológica. Diário da República, 35. Série II.

Reis, M., Matos, M. G., Simões, C., & Diniz, J. (2011). Saúde sexual e reprodutiva em estudantes do ensino superior. Dados nacionais 2010. Problemas emergentes e modelo compreensivo. Aventura Social.

Ribeiro, M. I., & Fernandes, J. (2009). Comportamentos sexuais de risco em estudantes do ensino superior. Público da cidade de Bragança. *Psicologia, Saúde & Doenças, 10*(1), 99-113.

Rocha, E. (2008). Universidades promotoras de saúde. *Revista Portuguesa de Cardiologia, 27*(1), 29-35. Retirado de http://www.spc.pt/DL/RPC/artigos/886.pdf

Sant'Anna, M. J., Carvalho, K. A., Bastos, M. B., & Coates, P.V. (2008). Sexual behavior of young university students. *Adolescência & Saúde, 5*(2), 52-56.

Silva, M., & Meneses, R. F. (2010). Educação para a saúde e atitudes sexuais de estudantes universitários. In *Actas do VII Simpósio Nacional de Investigação em Psicologia Universidade do Minho*, Braga.

Sociedade Portuguesa de Ginecologia. (2014). *Consensos sobre infeção por HPV e neoplasia intraepitelial do colo vulva e vagina*. Lisboa: Secção Portuguesa de Colposcopia e Patologia Cervico-Vulvovaginal.

Vilar, D. (2012). *Projeto Sexual Awareness for Europe (SAFE II)*. Lisboa: Associação para o Planeamento da Família.

Vilar, D., & Ferreira, P. M. (2009). Educação sexual dos jovens portugueses: Conhecimentos e fontes. *Educação Sexual em Rede, 5*, 2-53.

CAPÍTULO 4

ESTUDOS EMPÍRICOS EM SAÚDE SEXUAL E REPRODUTIVA

4.5. Artigo 5 - Uso do preservativo em estudantes do ensino superior: Aplicação do Modelo de Promoção da Saúde de Pender

Santos, M.J., Duarte, J., Ferreira, E., & Ferreira, M.

(Artigo a submeter à Revista Latino-Americana de Enfermagem)

USO DO PRESERVATIVO EM ESTUDANTES DO ENSINO SUPERIOR: APLICAÇÃO MODELO DE PROMOÇÃO DA SAÚDE DE NOLA PENDER

CONDOM USE IN COLLEGE STUDENTS: APPLICATION OF NOLA PENDER HEALTH PROMOTION MODEL

Maria José Santos[1]; João Duarte[3]; Elisabete Ferreira[2]; Manuela Ferreira[3]

[1] Escola Superior de Enfermagem de Vila Real, Universidade de Trás-os-Montes e Alto Douro, Vila Real, Portugal. mjsantos@utad.pt

[2] Faculdade de Psicologia e Ciências da Educação, Universidade do Porto, Porto, Portugal

[3] Escola Superior de Saúde de Viseu, Instituto Politécnico de Viseu, Viseu, Portugal

Resumo

Introdução: O uso consistente do preservativo é uma das estratégias mais eficazes para evitar não só a gravidez indesejada, mas também as infeções sexualmente transmissíveis. Apesar da sua eficácia, o preservativo tem uma utilização modesta pelos jovens, sendo desejável inverter essa tendência. A compreensão dos fenómenos que determinam a adesão a este comportamento é importante para que se possa atuar na sua modificação.

Objetivo: Pretende-se com este estudo identificar fatores preditores de uso do preservativo em estudantes do ensino superior.

Material e métodos: Foi realizado um estudo transversal, correlacional-preditivo numa amostra de conveniência de 1946 estudantes do ensino superior, 64% raparigas e 36% rapazes, com idade média de 21 anos (20,74±2,32). O Modelo de Promoção da Saúde de Pender (HPM) foi utilizado como referencial conceptual e metodológico para compreender a relação entre os determinantes para o uso do preservativo.

Resultados: A utilização do preservativo é baixa, apenas cerca de 40% referem o seu uso consistente. Os benefícios percebidos, os sentimentos positivos e as influências interpessoais foram as variáveis que tiveram uma influência positiva mais clara sobre o compromisso para utilizar o preservativo, com tendência semelhante em ambos os géneros. Por sua vez o compromisso é o melhor preditor do comportamento de usar o preservativo (β=0,580, $p<0,001$). A perceção de barreiras e sentimentos negativos influenciaram negativamente a utilização do preservativo e a autoeficácia para usar o preservativo não revelou capacidade preditiva.

Conclusões: O HPM de Pender mostrou-se adequado para explicar as relações entre as variáveis que determinam a utilização do preservativo. Os resultados identificam como

principais domínios de intervenção os benefícios percebidos, sentimentos positivos e as influências interpessoais, domínios que se devem trabalhar com os estudantes para os ajudar a comprometer-se com, e adotar o comportamento promotor de saúde - a utilização consistente do preservativo.

Palavras-chave: Comportamento sexual de risco, estudantes do ensino superior, modelo de promoção de saúde, preservativo.

Abstract

Introduction: Consistent condom use is one of the most effective strategies to prevent, not only unwanted pregnancies, but also sexually transmitted infections. Given the protective health effect that the condom represents, its use is a health promoting behaviour. Despite its effectiveness, condoms have a modest use by young people, and it is desirable to reverse this trend.

Objective: This study aims to identify predictive factors of condom use in college students.

Material and methods: A cross-sectional, predictive-correlational study was conducted in a convenience sample of 1946 higher education students, 64% girls and 36% boys, with a mean age of 21 years (20.74 ± 2.32).The Pender' Health Promotion Model was used as a conceptual and methodological reference to understand the relationship between the determinants for condom use.

Results: Condom use is low, only about 40% refer to its consistent use. Perceived benefits, positive affects, and interpersonal influences were the variables that had a clearer positive influence on condom use, with a similar trend in both genders. The perception of negative barriers and feelings negatively influences the use of the condom and the self-efficacy to use the condom did not reveal predictive capacity.

Conclusions: The Nola Pender' health promotion model was adequate to explain the relationships between the phenomena that determine the use of condoms. The results identify as main domains of intervention the perceived benefits, positive feelings and interpersonal influences, which it is necessary to work with the college students to increase the commitment and adoption of the health promoting behavior that represents the consistent condom use.

Keywords: Sexual risk behaviours, college students, Health Promotion Model, condom.

Introdução

O sexo desprotegido é um importante fator de morbilidade, e mesmo de mortalidade em algumas zonas do mundo, decorrente das infeções sexualmente transmissíveis (IST), incluindo o VIH/SIDA. Dos diversos métodos contracetivos, o preservativo masculino é o método mais recomendado na faixa etária jovem, em virtude da sua elevada eficácia, facilidade de utilização, disponibilidade e baixo custo (Centers of Diseases Control and Prevention [CDC], 2014). Promover o seu uso de forma correta e consistente é uma importante medida de promoção da saúde sexual e reprodutiva (SSR) dos jovens.

O uso consistente do preservativo pode diminuir 80% a 90% o risco de transmissão do HIV/SIDA de um parceiro infetado (Joint United Nations Programme on HIV/AIDS [UNAIDS], 2013). O preservativo feminino também protege de forma eficaz contra a transmissão de IST e gravidez indesejada, mas não está quantificado o seu grau de eficácia, dada a sua baixa utilização (Gallo, Kilbourne-Brook & Coffey, 2012). Diversos estudos revelam que a utilização do preservativo por estudantes universitários é inconsistente (Kann et al., 2014; Kritsotakis, Psarrou, Vassilaki, Androulaki & Philalithis, 2016; Matos, Reis, Ramiro & Aventura Social, 2011; Wilton, Palmer & Maramba, 2014). Num estudo realizado em Portugal sobre os comportamentos SSR de estudantes universitários (Matos et al., 2011), mais de metade dos participantes referiu usar como método contracetivo o preservativo (69%), sendo assumido por 31% dos estudantes que não o utilizam de forma consistente. Para além da inconsistência, a sua eficácia pode estar diminuída em resultado de falhas na correta utilização, estando descrito entre homens heterossexuais, que cerca de um terço apresenta falhas de utilização (31,3%), relacionadas com situações de rompimento precoce, aplicação tardia, remoção antes de tempo e outros (Crosby, Charnigo, Weathers, Caliendo & Shrier, 2007).

Alguns investigadores têm procurado um modelo explicativo das motivações que estão na origem da não utilização consistente do preservativo. Essas razões são diversificadas e estão relacionados com as características individuais, cognitivas, psicossociais e culturais. Estão descritos fatores como o género, a personalidade mais hedonista e impulsiva, onde os mitos associados à diminuição do prazer sexual podem representar uma barreira ao seu uso efetivo (Maia, 2010; Oliveira, 2015). Ao nível das variáveis cognitivas e psicossociais, o conhecimento, a atitude mais positiva, a autoeficácia e o compromisso individual para o usar (Asare, 2015; Kirby, Coyle, Alton, Rolleri & Robin, 2011; Reis, Ramiro, Matos & Diniz, 2013) o tipo de parceiro sexual, o estatuto da relação e a capacidade de negociação do seu uso com o parceiro sexual (Chamratrithirong & Kaiser, 2012; Manlove, kramullah & Terry-Humen, 2008; Oliveira, 2015) estão habitualmente relacionados com a adoção do comportamento promotor de saúde. As influências interpessoais refletem a importância que

é dada pelo indivíduo à opinião daqueles que lhe são próximos e cujo ponto de vista respeita, nomeadamente os amigos, namorados, familiares, profissionais de saúde, entre outros (Kirby et al., 2011). As influências situacionais decorrem da ocorrência de oportunidades que criam um ambiente favorável ao comportamento, promotor ou comprometedor de saúde. No contexto académico, em qua há uma forte componente de atividades sociais, muitas vezes associadas ao consumo de álcool ou drogas, reúnem-se condições para a ocorrência de comportamentos sexuais de risco (Caldeira et al., 2009; Logan, Koo, Kilmer, Blayney & Lewis, 2015; Lorant, Nicaise, Soto & d`Hoore, 2013; Riordan, Scarf & Conner, 2015). Nessas situações, as substâncias psicotrópicas podem afetar negativamente a capacidade para usar e negociar a utilização do preservativo com o parceiro sexual, ou mesmo de fazer uma avaliação racional sobre o nível de risco que um parceiro sexual possa representar (Uecker, 2015; Wicki, Kuntsche & Gmel, 2010; Wilton et al., 2014). Esse risco pode ser agravado pelos fatores culturais, tais como a cultura do "engate" (Kuperberg & Padgett, 2015), que encoraja os encontros sexuais casuais, normalmente de uma noite, como uma forma de experimentar intimidade sexual sem se investir emocionalmente num relacionamento. Esse comportamento foi aproveitado pela indústria de aplicações para telemóveis, nomeadamente o "Tinder", que tem cerca de 10 milhões de utilizadores por dia. Sumter, Sumter e Ligtenberg (2017) estudaram a adesão a esta aplicação entre jovens adultos (18-30 anos), tendo observado que há uma proporção assinalável que a usa para sexo casual. Dados de diversos estudos sugerem que entre 60% e 80% dos estudantes universitários relatam pelo menos uma experiência de sexo casual no decurso da sua vida académica, sem utilizarem o preservativo (Garcia, Reiber, Massey & Merriwether, 2012; Grello, Welsh, & Harper, 2006; Zeng, Luo & Zhou, 2015). Se o contexto académico é uma influência situacional que cria oportunidades para o envolvimento em comportamentos de risco, o acesso aos serviços de saúde, que permite aceder ao aconselhamento contracetivo e adquirir o preservativo de uma forma fácil e gratuita, é uma influência com o efeito previsivelmente positivo, estimulando a adesão ao comportamento promotor de saúde (UNAIDS, 2004).

O comportamento sexual, no qual se inclui o comportamento de proteção com o uso consistente do preservativo, resulta da interação entre indivíduos diferentes, e destes com o meio social e cultural, pelo que estudar os fatores que determinam o seu uso consistente reveste-se de particular complexidade. Nesse sentido é importante estudar fatores potencialmente modificáveis que possam contribuir para uma melhor compreensão das motivações que levam os indivíduos a não utilizarem de forma correta e consistente o preservativo, colocando em risco a sua saúde e, eventualmente, a dos seus parceiros sexuais. Face à contextualização, pretende-se com este estudo identificar fatores preditores

de uso do preservativo em estudantes do ensino superior, utilizando o modelo HPM como referencial conceptual.

Referencial teórico

O Modelo de Promoção da Saúde (HPM - Health Promotion Model, de Pender, Murdaugh & Parsons, 2011) foi utilizado como referencial teórico e metodológico, para identificar fatores preditores do uso do preservativo. Os autores propõem uma estrutura guia para explorar os complexos processos biológicos e psicológicos que motivam as pessoas a adotarem comportamentos salutogénicos.

O modelo contempla a natureza multidimensional das interações que os indivíduos têm ao realizar ações para promover e manter sua saúde, direcionando a ação dos enfermeiros para desenvolver intervenções de enfermagem promotoras de saúde no âmbito da promoção da saúde ao longo do ciclo de vida. As componentes do modelo (Pender et al., 2011; Vítor, Lopes & Ximenes, 2005) estão descritas na Figura 1 e incluem:

1) Características individuais e experiências, que compreende o comportamento comprometedor de saúde que carece ser mudado, assim como os fatores pessoais que podem influenciar a adesão a comportamentos salutogénicos (biológicos, psicológicos e socioculturais);

2) *Conhecimentos e sentimentos sobre o comportamento específico* que se pretende alcançar, e que envolve: i) a perceção de benefícios e barreiras para a ação, que são representações mentais, positivas ou negativas, que fortalecem ou dificultam a decisão de adotar o comportamento; ii) a autoeficácia percebida, que traduz a capacidade do individuo para executar o comportamento de saúde; iii) os sentimentos positivos ou negativos subjetivos relacionados com o comportamento de saúde; iv) as influências interpessoais, que incluem normas e apoio social e as influências situacionais relacionadas com as influências ambientais que podem facilitar ou impedir determinados comportamentos de saúde;

3) *Os resultados do comportamento*, que ao equacionar o compromisso com um plano de ação, possibilita a pessoa manter-se no comportamento desejado, reforçado pelas intervenções de enfermagem e pelas exigências imediatas e preferências, permite concretizar o comportamento de promoção de saúde que o indivíduo deve integrar no seu estilo de vida, resultando numa melhoria da sua saúde.

Material e métodos

Foi realizado um estudo transversal, descritivo-correlacional numa amostra de 1946 estudantes de uma universidade do norte de Portugal no ano letivo de 2012/2013. A técnica de amostragem foi o *cluster sampling,* onde o grupo ou a unidade de amostra foi a turma.

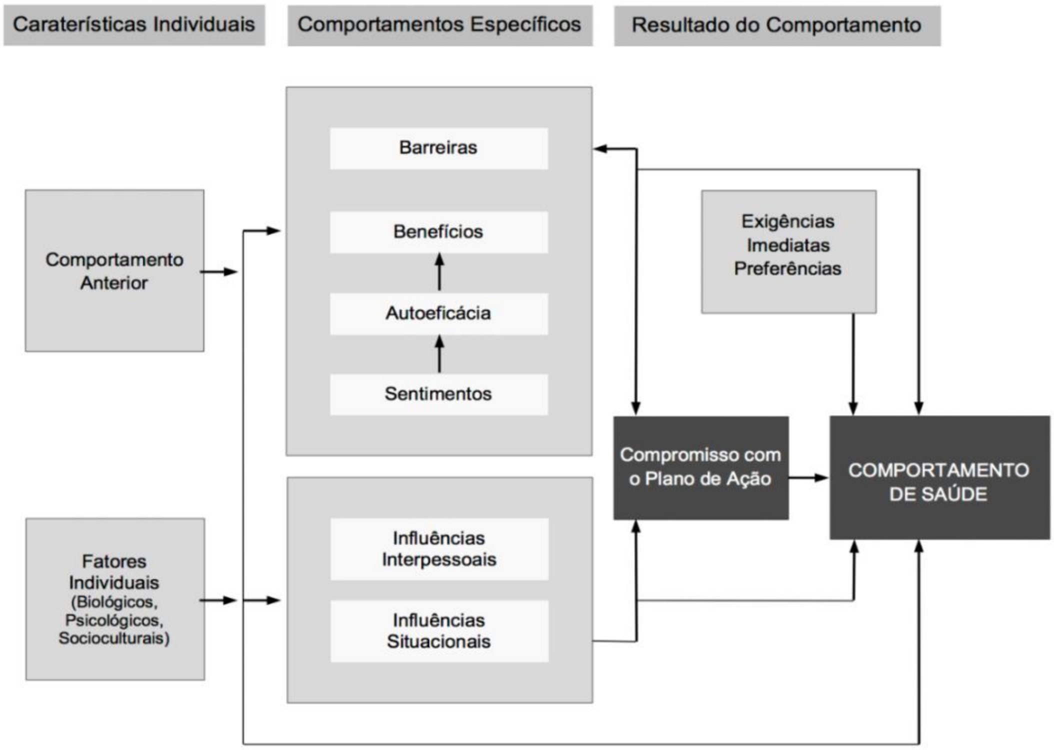

Figura 1. Modelo de Promoção da Saúde (HPM) (adaptado de Pender et al., 2011)

A recolha de dados foi realizada após aprovação do protocolo de investigação pelos órgãos da direção da universidade onde foi realizado o estudo. O questionário foi aplicado em sala de aula, no final da componente letiva, a todos os estudantes que se disponibilizaram para participar, depois de informados sobre os objetivos mesmo e do seu carácter voluntário e anónimo. Os pormenores de preenchimento constantes da folha de instruções do protocolo de investigação foram transmitidos a todos os estudantes, e o tempo médio de preenchimento foi de 50 minutos.

Instrumentos

Utilizou-se um questionário de autopreenchimento que permitiu recolher dados sobre: 1) *Características individuais e experiências*: incluiu os fatores biológicos e socioculturais (género, idade, nacionalidade, proveniência, escolaridade e rendimento dos pais e área científica de estudo, importância da religião), experiências relacionadas com o comportamento sexual e reprodutivo (já teve relações sexuais, idade da primeira relação

sexual; método contracetivo utilizado, orientação sexual, no último ano teve relações sexuais, as relações sexuais aconteceram no contexto de uma relação amorosa, utiliza contraceção, qual o método contracetivo) e comportamentos sexuais de risco (no último ano utilizou sempre o preservativo nas relações sexuais, teve parceiros ocasionais e consumo de álcool, drogas associadas às relações sexuais); 2) *Conhecimento e sentimentos sobre o comportamento* que se pretende alcançar e que foi avaliado pelas variáveis propostas pelo modelo HPM de Pender (Tabela 1); 3) *Comportamento promotor de saúde a ser implementado*, que no presente estudo foi conceptualizado como o comportamento de usar de forma consistente o preservativo.

Tabela 1.
Variáveis preditoras do uso do preservativo, de acordo com o HPM de Pender

Variáveis	Questões	Consistência interna Variação dos scores
**Benefícios percebidos**	"Ao adotar comportamentos preventivos, estou a prevenir futuras complicações de saúde reprodutiva".<br>"Para mim é fácil usar preservativo no meu dia-a-dia".	Alfa *Cronbach* =0,442<br>Scores entre 2-14
**Barreiras percebidas** Ψ	"Ir buscar preservativos ao centro de saúde é uma situação embaraçosa<br>"Falar com os profissionais de saúde sobre assuntos relacionados com o uso de contracetivos pode ser embaraçoso".<br>"Comprar preservativos é uma situação embaraçosa, porque expõe a minha intimidade".	Alfa *Cronbach* =0,720<br>Scores entre 3-21
**Sentimentos positivos** Ψ	"Usar e dialogar sobre os métodos contracetivos faz parte de uma sexualidade responsável".<br>"Sinto-me melhor comigo próprio quando uso métodos contracetivos".	Alfa *Cronbach* =0,420<br>Scores entre 2-14
**Sentimentos negativos** Ψ	" Usar o preservativo intefere com o prazer sexual".<br>"Ficaria incomodado se o meu parceiro me pedisse para usar preservativo".	Alfa=0,546<br>Scores entre 2-14
**Autoeficácia para o uso do preservativo** Φ	Versão Portuguesa da Escala de Autoeficácia do Uso do Preservativo (CUSES – Brien, Thombs, Mahoney & Wallnau, 1994), composta por 15 questões, organizadas numa estrutura de quatro fatores (mecanismos, desaprovação do parceiro, assertividade e intoxicantes).	Alfa *Cronbach* =0,820<br>Scores entre 0 -60
**Influências interpessoais** Ψ	"As pessoas que são importantes para mim aconselham-me a ter e a usar sempre o preservativo".<br>"É importante que os parceiros sexuais falem sobre o uso do preservativo".	Alfa *Cronbach* =0,594<br>Scores entre 2-14
**Influências Situacionais** Ψ	"É divertido ter experiências sexuais com parceiros ocasionais".<br>"Uma boa maneira de obter prazer sexual é tendo relações sexuais sob o efeito de álcool ou drogas".	Alfa *Cronbach* =0,421<br>Scores entre 2-14
**Compromisso com o plano de ação** Ψ	"Há uma grande probabilidade de eu usar o preservativo no próximo mês".<br>"Se tiver relações sexuais no próximo mês tenciono usar sempre o preservativo".	Alfa *Cronbach* =0,876<br>Scores entre 2-14

Ψ- As questões foram avaliadas numa escala tipo Likert com sete opções de resposta (1 – "*discordo completamente* " a 7 – "*concordo completamente*"; **Φ** – As questões foram avaliadas numa escala tipo Likert com cinco opções de resposta (0-"*discordo totalmente*" a 4-"*concorto totalmente*")

Caracterização da amostra

A amostra foi constituída por 1 946 estudantes (64% raparigas e 36% rapazes), com idade média de 21 anos (20,74±2,32) que frequentavam diversas áreas científicas de uma universidade do norte de Portugal. A maioria dos participantes era de nacionalidade portuguesa (97,3%), solteiros (97,6%), de famílias com baixo rendimento (57%, ≤ 2 salários mínimos), baixo nível de escolaridade (54,5% das mães e 61,6% dos pais têm apenas o

ensino básico) e com profissões pouco diferenciadas. O número de estudantes com orientação homossexual foi residual.

A maioria dos estudantes referiu já ter tido relações sexuais (82,4%), e a iniciação sexual ocorreu em média aos 17 anos (17±1,81). Apenas 17% dos estudantes refere que a primeira experiência sexual ocorreu antes dos 15 anos, predominando neste grupo os rapazes. Dois terços dos estudantes tiveram relações sexuais no último ano (76,9%) e, destes, 83,1% menciona que utilizou contraceção, sendo o método mais utilizado pelas raparigas o hormonal (52,9%) e pelos rapazes o preservativo (31,1%). A percentagem de estudantes que refere já ter tido uma IST é reduzida (1,3%), assim como a gravidez indesejada (3,1%). Uma percentagem significativa de estudantes reporta o envolvimento em comportamentos de risco sexual nos últimos doze meses, nomeadamente a utilização inconsistente do preservativo (60,5%), parceiros ocasionais (32,0%), e relações sexuais associadas ao consumo de álcool (33,0%) ou drogas (9,7%).

Considerações éticas

A realização do estudo salvaguardou os princípios éticos inerentes aos estudos de investigação, respeitando as questões de anonimato e confidencialidade e foi autorizado pela Comissão Nacional de Proteção de Dados (Autorização nº 7409/2013) e pela Comissão de Ética da universidade onde foi realizado (Parecer nº 2/2012).

Análise de dados

O tratamento estatístico dos dados foi efetuado com recurso ao programa SPSS 22.0 (SPSS, Inc, Chicago, IL, USA). A associação entre as características individuais e a utilização do preservativo foi estabelecida usando o teste de Qui-quadrado. A relação entre os constructos do HPM e o comportamento promotor de saúde usar o preservativo foi estabelecida pelo teste *T-student*. Antes de proceder ao tratamento estatístico, as variáveis relacionadas com o uso de preservativo foram ajustadas com a questão de controlo que serviu para saber se o estudante utilizava o preservativo de forma consistente. Considerou-se que o estudante utiliza preservativo somente quando na pergunta de controlo indicou usá-lo sempre. A utilização frequente ou esporádica foi considerada uma utilização não consistente de preservativo, e considerada não utilização.

Efetuaram-se modelos de mediação através de equações estruturais para compreender as relações e interações entre as variáveis pertencentes ao modelo teórico hipotético e o comportamento promotor da saúde mediada pelo compromisso com a ação. Foi ainda utilizada a *Path analysis (através do programa AMOS, versão 23) para testar* o modelo teórico explicativo do comportamento de utilização do preservativo. Esta técnica estatística

permitiu estudar as relações e interações entre as variáveis pertencentes ao HPM e incluir todas as variáveis dentro de um mesmo modelo de regressão (Marôco, 2011). A colinearidade entre as variáveis independentes inseridas no modelo foi avaliada pelos valores de inflação da variância (VIF), aceitando-se a ausência de colinearidade quando os valores foram inferiores a 5. Em todas as estatísticas inferenciais tomou-se como limite de significância o valor de $p<0,05$.

Resultados

A influência que algumas características individuais tiveram na adoção do comportamento promotor de saúde considerando apresenta-se na Tabela 2, separadamente para os estudantes do género feminino, masculino e para o total dos participantes. A taxa de não utilização consistente do preservativo ronda os 60% para ambos os géneros. A idade dos estudantes tem uma influência estatisticamente significativa na utilização regular do preservativo. Independentemente do género, apurou-se que os estudantes mais velhos são os que utilizam menos o preservativo. Nos estudantes mais jovens (≤ 19) a utilização é feita por cerca de metade dos estudantes. Essa proporção decresce até somente menos de um terço entre os estudantes com 25 anos ou mais velhos. A área de estudos em ciências da vida e saúde revelou-se uma influência positiva na adoção do comportamento promotor de saúde entre as raparigas, pois a proporção das que estudam nessa área e usam preservativo é maior ($p=0,027$) do que a das que estudam noutras áreas. Na população masculina não se detetou essa influência. O rendimento familiar não mostrou ter influência ($p>0,05$) na adoção do comportamento. A escolaridade dos pais foi redundante com o rendimento familiar, sendo que uma maior escolaridade determina maior rendimento, pelo que não se apresentam esses resultados.

A importância que os estudantes atribuem à religião determinou diferenças na adoção no comportamento, pois verificou-se que nos que professam religião é maior a proporção dos que usa preservativo consistentemente ($p=0,011$). O comportamento prévio “usou preservativo na primeira relação sexual”, que pretendia avaliar a preocupação em ter um comportamento sexual seguro logo no início da atividade sexual, influenciou significativamente ($p=0,001$) o comportamento atual dos rapazes. Entre as raparigas, ainda que o sentido da associação seja o mesmo, não foi significativa ($p=0,176$). A atividade sexual, se ocorrer no contexto de uma relação de namoro, resulta numa menor utilização do preservativo, quer por raparigas ($p=0,038$) quer por rapazes ($p<0,001$). Uma vez que a idade mais avançada e o envolvimento numa relação de namoro são ambos fatores que determinam o abandono do preservativo, equacionou-se que se tratasse do mesmo fenómeno – serem os estudantes mais velhos os que estão envolvidos em relações

estáveis. Observou-se porém que não há relação significativa entre a idade ($\chi 2$=0,009; p=0,995) e a atividade sexual no contexto do namoro.

No sentido de avaliar a relação entre as variáveis da componente do conhecimento e sentimentos relacionados com o comportamento específico, na utilização do preservativo, comparou-se o valor médio de cada variável separadamente por géneros e para a globalidade dos participantes (Tabela 3).

Tabela 2.
Influência de características individuais e experiências no comportamento promotor da saúde - uso do preservativo

Características individuais e experiências	Raparigas		Rapazes		Total	
	**Usa**	**Não usa**	**Usa**	**Não usa**	**Usa**	**Não usa**
n (min-max) (%)	369 (39,9%)	556 (60,1)	221 (38,7)	350 (61,3)	590 (39,4)	906 (60,6)
**Idade**						
≤ 19	**50,5**	**49,5**	**50,7**	**49,3**	**50,6**	**49,4**
20-24	**36,0**	**64,0**	**35,7**	**64,3**	35,9	64,1
≥ 25	**26,9**	**73,1**	32,0	69,0	29,3	70,7
χ^2 (p)	*20,535 (<0,001)*		*11,623 (0,003)*		*32,116 (<0,001)*	
**Área de estudos**						
Vida e Saúde	**42,32**	**57,8**	38,6	61,4	40,9	59,1
Humanas, Sociais e Tecnologias	**35,4**	**64,6**	39,1	60,9	37,0	63,0
χ^2 (p)	*3,962*	*(0,027)*	*0,014*	*(0,906)*	*2,234*	*(0,135)*
**Rendimento familiar**						
< 2 salários mínimos	40,4	59,6	40,6	59,4	40,5	59,5
2-4 salários mínimos	37,9	62,1	36,5	63,5	37,3	62,7
> 4 salários mínimos	42,2	57,38	38,1	61,9	40,2	59,8
χ^2 (p)	*0,756*	*(0,685)*	*0,842*	*(0,656)*	*1,246*	*(0,536)*
**Importância da religião**						
Reduzida/Nula	32,5	67,5	27,3	72,7	**29,9**	70,1
Moderada	37, 3	62,7	40,0	60,0	38,4	61,6
Elevada	43,2	56,8	41,3	58,7	42,5	57,5
χ^2 (p)	*4,956*	*(0,084)*	*5,066*	*(0,079)*	*9,026*	*(0,011)*
**Uso de preservativo na 1ª relação**						
Sim	41,3	58,7	**41,7**	**58,3**	41,5	58,5
Não	36,5	63,5	**24,2**	**75,8**	**33,2**	66,8
χ^2 (p)	*1,827*	*(0,176)*	*10,226*	*(0,001)*	*7,748*	*(0,003)*
**Relações sexuais no contexto de namoro**						
Sim	**39,1**	**60,9**	**34,9**	**65,1**	37,7	62,3
Não	**54,2**	**45,8**	**54,0**	**46,0**	**54,0**	46,0
χ^2 (p)	*4,302*	*(0,038)*	*13,861*	*(<0,001)*	*16,099*	*(<0,001)*

Observou-se que a maioria das variáveis estudadas teve uma associação significativa com a adoção do comportamento promotor de saúde. Os benefícios percebidos, os sentimentos positivos e as influências interpessoais foram as variáveis que tiveram uma influência

positiva mais clara sobre o comportamento, com tendência semelhante em ambos os géneros. A perceção do benefício e os sentimentos positivos face ao comportamento promotor de saúde apresentaram pontuações elevadas na escala em ambos os casos, nos que usam e nos que não usam preservativo, indicando que esses aspetos já estão razoavelmente interiorizados pelos estudantes. Porém, ainda que a pontuação seja elevada, há diferenças significativas entre os estudantes que têm o comportamento promotor de saúde e os que não o têm. As influências interpessoais apresentam pontuações próximas do centro da escala, sugerindo ser um aspeto que não é tão valorizado pelos estudantes. A autoeficácia para o uso do preservativo e as influências situacionais também contribuíram positivamente para o comportamento promotor de saúde, porém com um padrão ligeiramente diferente entre raparigas e rapazes. A autoeficácia para o uso do preservativo apenas se revelou significativa no grupo das raparigas, e as influências situacionais no grupo dos rapazes. As barreiras percebidas e os sentimentos negativos foram as duas variáveis que, como previsto na conceptualização teórica do modelo HPM, estiveram associadas ao comportamento promotor de saúde, mas no sentido negativo. Merece destaque, o facto da perceção de barreiras apresentar um valor elevado, pois em termos médios a pontuação dessa escala situou-se entre os 15 e os 16 pontos, num total de 21.

Na conceptualização dos determinantes da adoção do comportamento promotor de saúde do HPM, há relações e interrelações entre as variáveis dos domínios do conhecimento e sentimentos e das influências interpessoais e situacionais entre si e o compromisso para com o comportamento (designado no HPM por compromisso com o plano de ação) e com o comportamento propriamente dito. Ainda que através da análise univariada se tenha compreendido a maioria das relações entre os constructos do HPM e o uso do preservativo, perde-se a perceção do efeito que exercem em conjunto.

A modelização de fenómenos complexos através de equações estruturais é uma técnica baseada em regressões múltiplas que se adequa à quantificação da importância relativa dessas relações, principalmente porque admite variáveis mediadoras, que são simultaneamente dependentes e independentes. No HPM, este papel é claramente atribuído à variável compromisso com a ação, ou no contexto do presente trabalho, compromisso com o uso do preservativo.

Considerando as diferenças observadas na análise univariada entre os géneros, esta análise foi também realizada para raparigas (Tabela 4, Figura 2), rapazes (Tabela 4, Figura 3) e para o total da amostra estudada (Tabela 4, Figura 4). Observou-se que os benefícios e barreiras percebidos e os sentimentos têm uma forte influência na variável mediadora em ambos os géneros e, por inerência, na amostra total estudada, ainda que o valor dos coeficientes da trajetória (β) sejam ligeiramente diferentes.

Tabela 3.
Influência dos constructos do HPM relacionados com o compromisso com o plano de ação e o comportamento promotor de saúde- uso do preservativo

Constructos do HPM *(intervalo da escala)*	**Raparigas**			**Rapazes**			**Total**		
	**Usa**	**Não usa**	*t-Student (p)*	**Usa**	**Não usa**	*t - Student (p)*	**Usa**	**Não usa**	*t- Student (p)*
**Beneficios percebidos** (2-14)	12,56±2,27	11,46±2,50	*-6,861 (< 0,001)*	11,99±2,36	11,09±26	*-4,147 (< 0,001)*	12,35±2,32	11,32±2,53	*-8,008 (< 0,001)*
**Barreiras percebidas** (3-21)	15,51±4,62	16,33±4,51	*2,675 (0,008)*	15,01±4,40	15,78±4,44	*2,023 (0,044)*	15,32±4,54	16,11±4,48	*3,327 (0,001)*
**Autoeficácia para o uso do preservativo** (0-60)	50,14±7,56	48,93±8,59	*-2,248 (0,025)*	48,13±9,47	47,86±9,65	*-0,329 (0,742)*	49,38±8,38	48,52±9,02	*-1,895 (p=0,058*
**Sentimentos positivos** (2-14)	13,26±1,59	12,65±1,85	*-5,370 (< 0,001)*	12,09±2,05	11,03±2,24	*-5,699 (< 0,001)*	12,82±1,86	12,03±2,15	*36,501 (<0,001)*
**Sentimentos negativos** (2-14)	2,90±1,88	3,44±2,09	*4,023 (< 0,001)*	3,52±2,27	4,16±2,53	*3,090 (0,002)*	3,13±2,06	3,72±2,29	*5,088 (<0,001)*
**Influências interpessoais** (2-14)	8,84±3,42	7,46±3,57	*-5,789 (< 0,001)*	10,91±2,64	9,49±3,21	*-5,669 (< 0,001)*	9,61±3,31	8,25±3,57	*-7,460 (<0,001)*
**Influências situacionais** (2-14)	11,95±2,43	11,84±2,64	*-0,595 (0,552)*	9,09±2,90	8,70±3,06	*-1,528 0,0127*	10,87±2,96	10,63±3,20	*-1,517 (0,130)*

Tabela 4.
Coeficientes do modelo final refinado para predizer a adoção do comportamento promotor de saúde de usar preservativo (variável dependente), do compromisso com o plano de ação (variável mediadora) e os constructos do HPM (variáveis independentes)

Compromisso com o comportamento desejado		Constructos HPM	C.R.	P	β
**Raparigas**					
**Compromisso com o comportamento de usar o preservativo**	←	Beneficios percebidos	8,218	***	0,239
	←	Barreiras percebidas	-4,497	***	-0,126
	←	Sentimentos positivos	3,518	***	0,100
	←	Sentimentos negativos	-5,201	***	-0,151
	←	Influências interpessoais	12,458	***	0,354
	←	Influências situacionais	-2,159	0,031	-0,060
Comportamento promotor de saúde	←	Influências interpessoais	-2,705	0,007	-0,078
	←	Compromisso com o comportamento	22,433	***	0,643
**Rapazes**					
**Compromisso com o comportamento de usar o preservativo**	←	Beneficios percebidos	3,317	***	0,129
	←	Barreiras percebidas	-3,175	0,001	-,120
	←	Sentimentos positivos	5,568	***	0,215
	←	Sentimentos negativos	-1,971	0,049	-0,078
	←	Influências interpessoais	9,321	***	0,350
Comportamento promotor de saúde	←	Compromisso com o comportamento	14,280	***	0,516
**Total da população**					
**Compromisso com o comportamento de usar o preservativo**	←	Beneficios percebidos	8,575	***	0,200
	←	Barreiras percebidas	-5,719	***	-0,130
	←	Sentimentos positivos	5,825	***	0,136
	←	Sentimentos negativos	-5,269	***	-0,126
	←	Influências interpessoais	16,263	; p<0,001	0,370
	←	Influências situacionais	-2,238	025	-0,051
**Comportamento promotor de saúde – usar preservativo**	←	Compromisso com o comportamento	24,031	***	0,580
	←	Barreiras percebidas	-2,065	0,039	-0,045
	←	Sentimentos positivos	3,047	0,002	0,065
	←	Influências interpessoais	-2,271	0,023	-0,054

*** p<0,001; Valores de VIF são <5 (variando entre 1,035 e 1,186), indicam que não existem problemas de colinearidade entre as variáveis.

Para as raparigas, os benefícios percebidos são mais importantes (β=0,239; p<0,001) do que para os rapazes (β=0,129; p<0,001), assim como os sentimentos negativos. Pelo contrário, o compromisso com o comportamento promotor de saúde nos rapazes é mais influenciado pelos sentimentos positivos (β=0,215; p<0,001) do que nas raparigas (β=0,100; p<0,001).

Para ambos, as influências interpessoais é o constructo do HPM que se mostra melhor preditor do compromisso com o comportamento, com coeficiente de trajetória da ordem dos 0,35, altamente significativo. As influências situacionais não se mostraram um bom preditor do compromisso. Nos rapazes, esta variável não foi significativa e foi excluída do modelo, e nas raparigas e na amostra total, ainda que significativos, apresentaram valores absolutos muito reduzidos (β= -0,060, vs β= -0,051, respetivamente). Em concordância com o HPM, os coeficientes de trajetória entre o compromisso com o comportamento e o comportamento promotor de saúde são muito elevados e altamente significativos. Essas associações tiveram o seu valor máximo no grupo das raparigas (β= 0,643; p<0,001). Nos rapazes, esse coeficiente de trajetória ficou-se pelos 0,516 (p<0,001), revelando que elas são provavelmente mais eficazes em materializar o compromisso num comportamento efetivo. Por outro lado, o modelo encontrado para os rapazes (Figura 4) revela que a única variável que determina a adoção do comportamento é estar comprometido com ele, pois não se observaram trajetórias diretas com significado estatístico entre os constructos do HPM e o comportamento promotor de saúde. Nas raparigas acresce ao compromisso um pequeno contributo negativo (β= -0,078; P=0,007) das influências interpessoais.

Possivelmente devido ao maior número de observações, e por ter assimilado as tendências ligeiramente diferentes de ambos os géneros, quando se estudou a população completa, a variável compromisso foi a que revelou melhor capacidade preditiva, explicando 58% da variabilidade do comportamento de usar o preservativo (β=0,580; p<0,001). Observaram-se ainda pequenos contributos diretos das barreiras percebidas (β= -0,045; p=0,035), dos sentimentos positivos (β=0,065; p=0,002) e das influências interpessoais (β= -0,054; p=0,023).

Discussão

A vida académica corresponde a uma fase de desenvolvimento psicológico e biológico do indivíduo que, combinada com o contexto social muito apelativo, pode resultar numa grande procura e envolvimento em relações sexuais, que podem ou não acontecer numa relação de namoro (Kritsotakis et al., 2016). As relações sexuais em que esses estudantes se envolvem, se não forem devidamente protegidas, podem representar um risco acrescido para a aquisição de IST (Cragg, Steenbeek, Asbridge, Andreou & Langille, 2016).

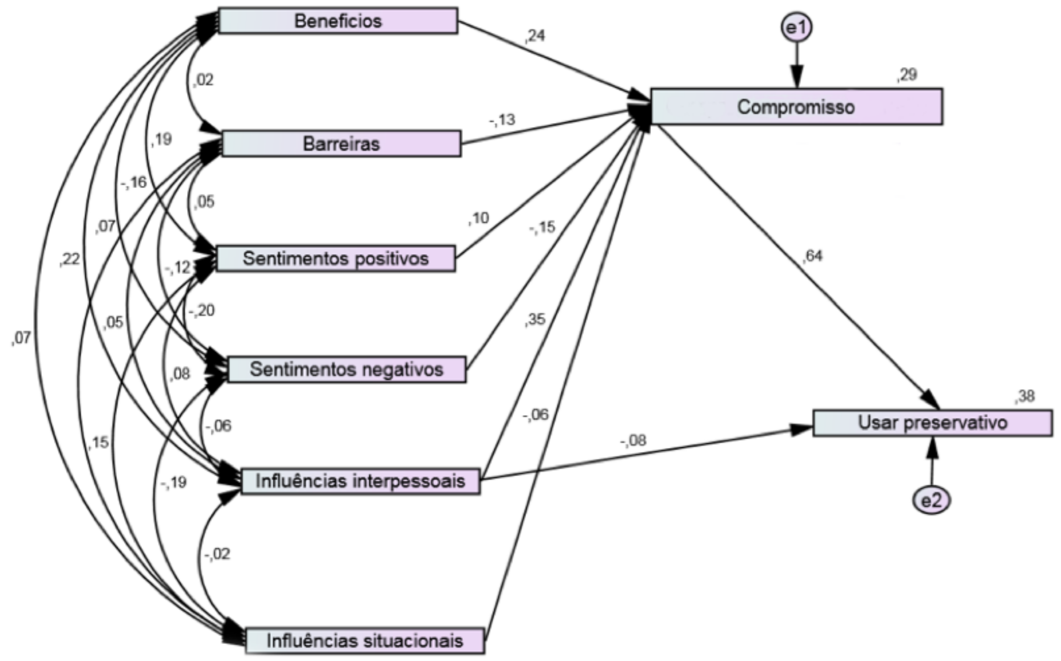

Figura 2. Modelo final refinado da associação entre adoção do comportamento promotor de saúde de usar preservativo, do compromisso com o plano de ação e os constructos do HPM, para o género feminino

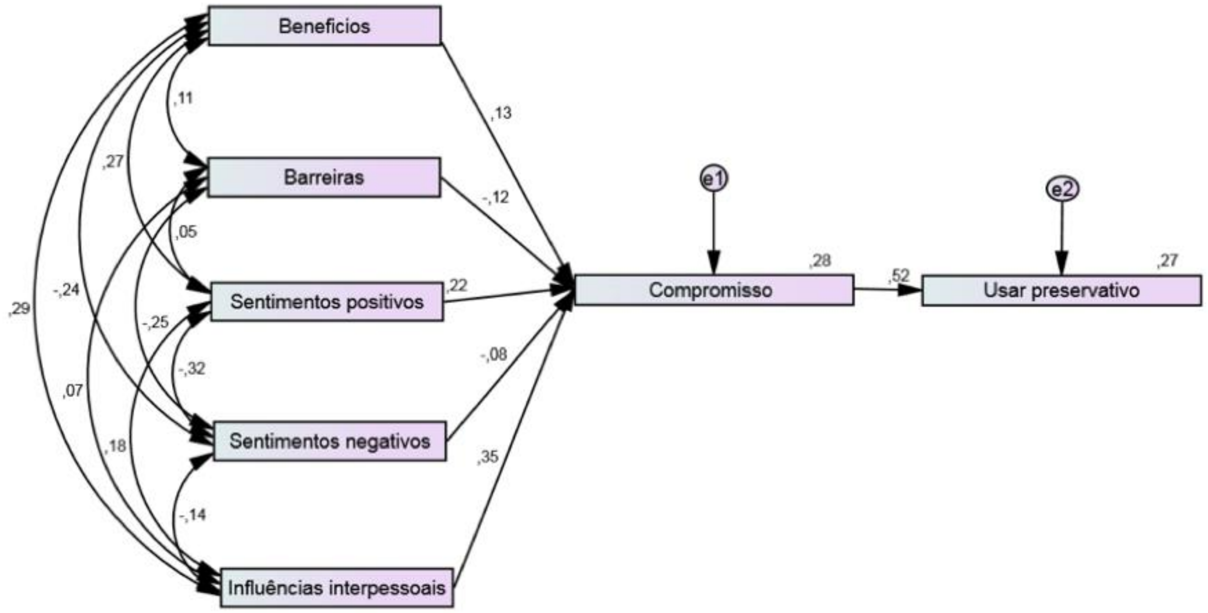

Figura 3. Modelo final refinado da associação entre adoção do comportamento promotor de saúde de usar preservativo, do compromisso com o plano de ação e os constructos do HPM, para o género masculino

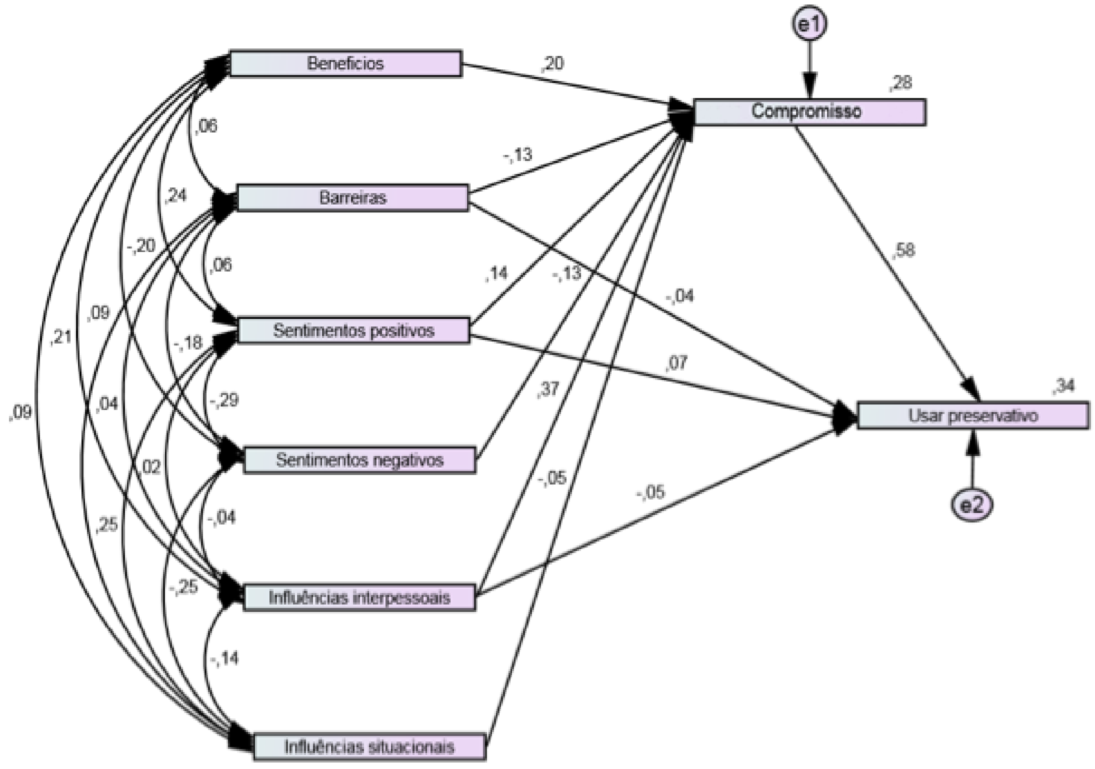

Figura 4. Modelo final refinado da associação entre adoção do comportamento promotor de saúde de usar preservativo, do compromisso com o plano de ação e os constructos do HPM, para o total da população estudada

À luz do conhecimento atual, em alternativa à pouca atrativa abstinência sexual (Underhill, Montgomery & Operario, 2008), a utilização consistente e correta do preservativo é a forma mais eficaz de evitar a transmissão de IST, tendo a vantagem adicional de ser um método de elevada eficácia na prevenção da gravidez, ser prático de usar, não necessitar de um planeamento muito complexo, e ser razoavelmente económico. Pelo papel que o seu uso consistente tem na prevenção de IST, deve ser considerado um comportamento promotor de saúde (Sagherian, Huedo-medina, Pellowski, Eaton & Johnson, 2016). Não obstante todas estas vantagens, no presente trabalho observou-se uma baixa adesão a este método, não chegando aos 40% a percentagem de estudantes que o usa de forma consistente. Vários estudos, quer europeus quer norte-americanos, observaram também esta tendência, constatando que a percentagem de utilização do preservativo por jovens não ultrapassava os 60% (CDC, 2014; Kann et al., 2013). Num estudo de âmbito nacional com estudantes universitários, os resultados revelaram que a situação em Portugal é mais grave, dado que apenas 32,8% utilizou o preservativo de forma consistente (Matos et al., 2011). A elevada proporção de não utilizadores do preservativo é preocupante do ponto de vista da prevenção de IST, pois mesmo havendo uma baixa taxa de infeção autorreferida pelos estudantes,

desconhecem-se as consequências reais que esse problema possa ter, pois muitas das IST podem ser assintomáticas, algumas durante vários anos, como a infeção por HIV e por HPV (Kann et al., 2013; Patel et al., 2012).

Os estudantes que usam menos o preservativo são os mais velhos, que dão pouca importância à religião, que têm relações sexuais no contexto da relação de namoro, as raparigas que estudam em áreas de ciências humanas e sociais e os rapazes que iniciaram a sua vida sexual utilizando o preservativo. Estas tendências são basicamente as encontradas por outros autores (Parks et al., 2017, Reis, Ramiro, Gaspar Matos, Alves & Diniz, 2012). A não utilização ou utilização inconsistente do preservativo é particularmente preocupante, não só pelos efeitos diretos que pode ter a curto e longo prazo na saúde reprodutiva, mas também porque é nesta altura de ganho de autonomia em relação à família que o jovem vai consolidar muitos dos comportamentos relacionados com a saúde que vai ter ao longo da sua vida (Kritsotakis et al., 2016). Face à necessidade de mudar esse comportamento comprometedor da saúde para um comportamento promotor, estudaram-se alguns constructos que, de acordo com o HPM, contribuem para a mudança. Os resultados do presente trabalho demonstram a coerência e adequação do HPM a esta problemática, pois as associações e interrelações previstas pelos autores encontram-se, na sua maioria, demonstradas na população alvo do presente estudo. Ainda que as tendências principais tenham sido muito semelhantes, observou-se que o padrão das raparigas e dos rapazes foi ligeiramente diferente. Os estudantes têm perceção dos benefícios que decorrem da utilização do preservativo, sendo esse um dos constructos que, mediado pelo compromisso com a ação, se mostrou um dos melhores preditores para o seu uso efetivo. A perceção dos benefícios do uso do preservativo na prevenção de IST é habitualmente tida pelos estudantes, ainda que a sua utilização efetiva passe aparentemente pela perceção de que não estão expostos ao risco (Cragg et al., 2016). Os sentimentos positivos, relativos à gratificação associada à opção mais responsável, são também apontados como um dos melhores preditores para o compromisso com o comportamento de usar o preservativo, particularmente pelos rapazes participantes no presente estudo, o que é contraditório com as elevadas taxas de não utilização observadas. Provavelmente, a discrepância é fomentada pelas barreiras percebidas, pois esse constructo contribui significativamente para a não adoção do compromisso com o uso do preservativo. As situações de embaraço relacionadas com a aquisição e uso do preservativo, que são exploradas nas barreiras percebidas, continuam a ser um dos aspetos que mais dificulta a utilização sistemática do preservativo (Kirby et al., 2011; Mmari & Sabherwal, 2013). As influências interpessoais são a variável que teve um contributo maior para o compromisso com o comportamento, revelando que a opinião das pessoas que lhes são importantes e do parceiro sexual

contribuem para o desenvolvimento do processo intelectual que determina a assunção do compromisso de usar o preservativo (Asare, 2015; Black, Schmiege & Bull, 2013). Essas são tão importantes nas raparigas que, para além da relação com o comportamento através da variável mediadora compromisso, mostrou também capacidade preditiva direta. A perspetiva hedonista e de risco associada ao envolvimento em relações sexuais com parceiros ocasionais ou o sob o efeito de álcool ou drogas que foi considerada nas influências situacionais, ainda que previsivelmente fosse um determinante importante na não adoção do compromisso (Sales et al., 2013), mostrou alguma influência, mas com coeficientes de trajetória modestos, indicadores do seu reduzido contributo. Esta tendência poderá ser ligeiramente maior na realidade do que o que os resultados do presente estudo demonstram, pois essas influências situacionais ocorrem muitas vezes associadas a momentos de impulsividade (Stevens, Littlefield, Talley & Brown, 2017) que poderão não ser facilmente reconhecidos pelos estudantes.

Na sua concretização, o estudo apresenta algumas limitações, nomeadamente o facto de ter sido realizado apenas numa universidade utilizando uma amostra não probabilística de conveniência., o que pode limitar a generalização dos resultados, contudo, esta limitação pode ter sido minimizada pelo facto dos estudantes serem provenientes de diversas zonas do país e das turmas onde foram aplicados os instrumentos de recolha de dados terem sido selecionadas de forma acidental. As medidas de avaliação do comportamento sexual foram auto-referidas, o que pode não representar uma medida precisa do comportamento real e estar associada a um viés relacionado com a desejabilidade social devido à natureza sensível da área em estudo. Adicionalmente, pode ter existido ainda um viés de memória, associado ao distanciamento temporal.

Conclusões

A SSR dos jovens universitários é influenciada por múltiplos fatores, individuais, sociais, cognitivos, psicossociais e relacionados com a sua própria personalidade, pelo que se reveste de alguma complexidade. Porém, há uma prática simples e económica que pode reduzir substancialmente o risco de gravidez indesejada e de IST – o uso consistente do preservativo. Essa utilização consistente do preservativo é um comportamento promotor de saúde que se pretende incentivar nos estudantes. Assim, utilizando o referencial teórico do HPM de Pender, procurou-se perceber quais os determinantes que tinham um maior impacto no compromisso para utilizar o preservativo e na sua utilização propriamente dita. Observou-se que a maioria das variáveis estudadas teve uma associação significativa com o compromisso para adotar o comportamento promotor de saúde. Os benefícios percebidos, os sentimentos positivos e as influências interpessoais foram as variáveis que tiveram uma

influência positiva mais clara sobre o compromisso para utilizar o preservativo, com tendência semelhante em ambos os géneros. A perceção de barreiras e sentimentos negativos influenciaram negativamente a adoção do comportamento de usar o preservativo. A autoeficácia para usar o preservativo, ao contrário do que seria de esperar, mostrou-se um fraco preditor, quer para o compromisso, quer do comportamento propriamente dito. A estrutura multivariada da associação dos constructos psicossociais e o comportamento promotor de saúde demonstra que o modelo teórico, que refere que o acontecimento que marca a adoção do comportamento é, sobretudo, o compromisso com a ação. Os resultados demostram essa forte associação, pois o compromisso tem uma elevada associação com o uso do preservativo. Estes resultados identificam quais são os principais domínios (benefícios percebidos, sentimentos positivos e influências interpessoais) em que se deve trabalhar com os estudantes do ensino superior para os ajudar a comprometer-se com, e adotar o comportamento promotor de saúde - a utilização consistente do preservativo.

Referências bibliográficas

Asare, M. (2015). Using the theory of planned behavior to determine the condom use behavior among college students. *American Journal of Health Studies, 30* (1), 43-50. Retirado de https://www.ncbi.nlm.nih.gov/pmc/articles/PMC4621079/

Black, S. R., Schmiege, S., & Bull, S. (2013). Actual versus perceived peer sexual risk behavior in online youth social networks. *Translational Behavioral Medicine*, 3(3), 312-319. doi:10.1007/s13142-013-0227-y

Caldeira, K. M., Arria, A. M., O'Grady, K. E., Zarate, E. M., Vicent, K. B, & Wish, E. D. (2009). Prospective associations between alcohol and drug consumption and risky sex among female college students. *Journal Alcohol Drug Education*, *53* (2), 1-13.

Centers for Disease Control and Prevention. (2014). *Sexually transmitted disease surveillance 2013.* Atlanta, Georgia: Division of STD Prevention.

Chamratrithirong, A., & Kaiser, P. (2012). The dynamics of condom use with regular and casual partners: Analysis of the 2006 National Sexual Behavior Survey of Thailand. *PLoS ONE 7* (7), e42009. doi:10.1371/journal.pone.0042009

Cragg A., Steenbeek, A., Asbridge,M., Andreou, P., Langille, D. (2016).Sexually transmitted infection testing among heterosexual Maritime Canadian university students engaging in different levels of sexual risk taking. *Canadian Journal Public Health*, *107*(2), e149-54. doi:10.17269/cjph.107.5036

Crosby, R. A., Charnigo, R. A., Weathers, C., Caliendo, A. M., & Shrier, L. A. (2012). Condom effectiveness against non-viral sexually transmitted infections: A prospective study using electronic daily diaries. *Sexually Transmitted Infections, 88*(7), 484-489. doi:10.1136/sextrans-2012-050618

Gallo, M. F., Kilbourne-Brook, M., & Coffey, P. S. (2012). A review of the effectiveness and acceptability of the female condom for dual protection. *Sexual Health*, *9*(1), 18-26.

Garcia, J. R., Reiber, C., Massey, S. G., & Merriwether, A. M. (2012) .Sexual hookup culture: A review. *Review of General Psychology, 16,* 161-176.

Grello, C. M., Welsh, D. P., & Harper, M. S. (2006). No strings attached: The nature of casual sex in college students. *The Journal of Sex Research*, *43* (3), 255-267.

Guedes, N. G., Morreira, R. P., Cavalcante, T. F., Araújo, T. L., & Ximenes, L. B. (2009). Atividade física de escolares: Análise segundo o modelo teórico de promoção da saúde de Pender. *Revista da Escola de Enfermagem da USP*, *43* (4), 774-780.

Joint United Nations Programme on HIV/AIDS. (2013). *Global report: UNAIDS report on the global AIDS epidemic 2013*. Geneva: UNAIDS.

Kann, L., Kinchen, S., Shanklin, S. L., Flint, K. H., Hawkins, J., Harris, W. A.,… Centers of Diseases Control and Prevention. (2014). Youth risk behavior surveillance-United States, 2013. *Morbidity and Mortality Weekly Report, 63*, 1-168.

Kirby, D., Coyle, K., Alton, F., Rolleri, L., & Robin, L. (2011). *Reducing adolescent sexual risk. Theoretical guide for developing and adapting curriculum-based programs*. California: ETR Associates.

Kritsotakis, G., Psarrou, M., Vassilaki, M., Androulaki, Z., & Philalithis, A. E. (2016). Gender differences in the prevalence and clustering of multiple health risk behaviours in young adults. *Journal of Advanced Nursing*, *72*(9), 2098-2113. doi:10.1111/jan.12981

Kuperberg, A., & Padgett, J. E. (2015). Dating and hooking up in college: Meeting contexts, sex, and variation by gender, partner's gender, and class standing. *The Journal of Sex Research*, *52*(5), 517-531. doi:10.1080/00224499.2014.901284

Logan, D. E., Koo, K. H., Kilmer, J. R., Blayney, J. A., & Lewis, M. A. (2015). Use of drinking protective behavioral strategies and sexual perceptions and behaviors in U.S. college students. *Journal of Sex Research*, *52*(5), 558-569. doi:10.1080/00224499.2014.964167

Lorant, V., Nicaise, P., Soto, V. E., & d`Hoore, W. (2013). Alcohol drinking among college students: College responsibility for personal troubles. *BMC Public Health*, *13*, 615-619. doi:10.1186/1471-2458-13-615.

Maia, M. (2010). Práticas de risco no contexto das relações homossexuais. In P. M. Ferreira & M. V. Cabral, *Sexualidades em Portugal: Comportamentos de risco* (Cap. 9, pp. 324-387). Lisboa: Editorial Bizâncio,

Manlove, J., Ikramullah, E., & Terry-Humen, E. (2008). Condom use and consistency among male adolescents in the United States. *Journal Adolescent Health, 43,* 325-333. doi: 10.1016/ j.jadohealth.2008.03.008.

Matos, M. G., Reis, M. Ramiro, L., & Equipa Aventura Social. (2011). *Saúde sexual e reprodutiva dos estudantes do ensino superior. Relatório do estudo: Dados nacionais 2010*. Lisboa: Aventura Social.

Mccullagh, M. (2009). Health promotion. In S. Peterson & T. Bredow (Eds.), *Middle Range theories: Application to nursing research* (pp. 3-45), Philadelphia: Wolters kluwer/Lippincott Williams & Wilkins.

Mmari, K., & Sabherwal, S. (2013). A review of risk and protective factors for adolescent sexual and reproductive health in developing countries: An update. *Journal Adolescent Health*, *53*(5), 562-572. doi:10.1016/j.jadohealth.2013.07.018

Oliveira, J. G. (2015). A confiança nas relações homoeróticas femininas. *LES Online, 7*(1), 17-25.

Parks, K.A., Frone, M.R., Muraven, M., & Boyd, C. (2017). Nonmedical use of prescription drugs and related negative sexual events: Prevalence estimates and correlates in college students. *Addictive Behaviors,* 65, 258-263. doi.org/10.1016/j.addbeh.2016.08.018

Patel, D. A., Zochowski, M., Peterman, S., Dempsey, A. F., Ernst, S., & Dalton, V. K. (2012). Human Papillomavirus Vaccine Intent and uptake among female college students. *Journal of American College Health, 60*(2), 151-161. doi: 10.1080/07448481.2011.580028

Pender, N., Murdaugh, C., & Parsons, M. A. (2011). Individual models to promote health behavior. In M. Connor, D. MacKnight, K. Mortimer & S. Wrocklage (Eds.), *Health promotion in nursing practice* (pp. 35-66). New York: Pearson.

Reis, M., Ramiro, L., Gaspar Matos, M., Alves, & Diniz, J. (2012). Nationwide survey of contraceptive and sexually transmitted infection knowledge, attitudes and skills of university students in Portugal. *International Journal of Clinical and Health Psychology*, *13*(2), 127-137.

Reis, M., Ramiro, L., Matos, M. G., & Diniz, J. A. (2013). Determinants influencing male condom use among university students in Portugal. *International Journal of Sexual Health, 25(2), 115-127.*

Riordan, B. C., Scarf, D., & Conner, T. S. (2015). Is orientation week a gateway to persistent drinking in university students: A preliminary investigation. *Journal of Studies on Alcohol and Drugs*, *76*, 204–211.

Sagherian, M. J., Huedo-Medina, T. B., Pellowski, J. A., Eaton, L. A., & Johnson, B.T. (2016). *Single-session behavioral interventions for sexual risk reduction: a meta-analysis .The Society of Behavioral Medicine, 50*, 920–934. doi10.1007/s12160-016-9818-4

Sales, J. M., Smearman, E. L., Brody, G. H., Milhausen, R., Philibert, R. A., & Diclemente, R. J. (2013). Factors associated with sexual arousal, sexual sensation seeking and sexual satisfaction among female African American adolescents. *Sexual Health, 10*, 512-521.

Stevens, A. K., Littlefield, A. K., Talley, A. E., & Brown, J. L (2017). Do individuals higher in impulsivity drink more impulsively? A pilot study within a high risk sample of young adults, United States. *Addictive Behaviors 65,* 147-153.

Sumter, S. R., Vandenbosch, L., & Ligtenberg, L. (2017). Love me Tinder: Untangling emerging adults' motivations for using the dating application Tinder. *Telematics and Informatics, 34*, 67-78.

Uecker, J. E. (2015). Social Context and Sexual Intercourse among First-Year Students at Selective Colleges and Universities in the United States. *Social Science Research*, *52,* 59-71.

Underhill, K., Montgomery, P., & Operario, D. (2008). Abstinence-plus programs for HIV infection prevention in high income countries. Cochrane Database Systematic Reviews, (1). doi: 10.1002/14651858.CD007006.

Vítor, J. F., Lopes, M. V., & Ximenes, L. B. (2005). Análise do diagrama do modelo de promoção da saúde de Nola J. Pender. *Acta Paulista de Enfermagem, 18*(3), 235-240.

Wicki, M., Kuntsche, E., & Gmel, G. (2010). Drinking at European universities? A review of students' alcohol use. *Addictive Behaviors*, *35,* 913-924. doi: 10.1016/j.addbeh.2010.06.015

Wilton, L., Palmer, R. T., & Maramba, L. C. (2014). *Understanding HIV and STIs prevention for college students*. London: Routledge Research in Higher Education.

Zeng, Y., Luo, T., & Zhou, Y. (2015). Relationships between attitudes toward sexuality, sexual behaviors, and contraceptive practices among Chinese medical and nursing undergraduates, China. *Nursing and Health* Sciences, *17,* 287-292 doi: 10.1111/nhs.12185

CAPÍTULO 4

ESTUDOS EMPÍRICOS EM SAÚDE SEXUAL E REPRODUTIVA

4.6. Artigo 6 - *“Usar o preservativo não é questão de confiança é uma questão de saúde*”**. Discussão focalizada com estudantes de ensino superior sobre comportamentos de saúde sexual e reprodutiva**

"USAR O PRESERVATIVO NÃO É QUESTÃO DE CONFIANÇA É UMA QUESTÃO DE SAÚDE". DISCUSSÃO FOCALIZADA COM ESTUDANTES DE ENSINO SUPERIOR SOBRE COMPORTAMENTOS DE SAÚDE SEXUAL E REPRODUTIVA

"USING CONDOMS IS NOT A MATTER OF TRUST IS A MATTER OF HEALTH"

FOCUSED DISCUSSION WITH COLLEGE STUDENTS ON SEXUAL AND REPRODUCTIVE HEALTH BEHAVIOURS

Maria José Santos[1]; Manuela Ferreira[2]; Elisabete Ferreira[3]

[1] Escola Superior de Enfermagem de Vila Real, Universidade de Trás-os-Montes e Alto Douro, Vila Real, Portugal. mjsantos@utad.pt

[2] Escola Superior de Saúde de Viseu, Instituto Politécnico de Viseu, Viseu, Portugal.

[3] Faculdade de Psicologia e Ciências da Educação, Universidade do Porto, Porto, Portugal.

Resumo

Introdução: A promoção da saúde sexual e reprodutiva (SSR) adquire particular importância nas faixas etárias mais jovens, as idades em que se conquista uma maior autonomia e se estabelecem as primeiras relações de intimidade. A entrada no ensino superior pode comportar riscos adicionais, decorrente do estilo de vida académico, onde estão descritos diversos comportamentos sexuais com reflexos negativos na SSR.

Objetivos: Identificar as características individuais e os comportamentos de SSR atuais; Conhecer as perceções dos estudantes sobre benefícios, barreiras, influências situacionais e contextuais, sentimentos e autoeficácia, com implicações no comportamento sexual seguro; identificar estratégias que os estudantes consideram relevantes para a construção de programas de promoção da SSR dirigidos a estudantes do ensino superior.

Metodologia: Foi realizado um estudo qualitativo, com recurso à técnica dos grupos de discussão focalizada, participando 21 estudantes do ensino superior, divididos em dois grupos mistos. O Modelo de Promoção da Saúde (HPM) de Pender foi utilizado como referencial teórico e metodológico na análise interpretativa dos dados, considerando as suas três componentes principais: características individuais e experiências, comportamento específico e resultado do comportamento.

Resultados: No que respeita à adoção de comportamentos sexuais seguros, ao nível das características individuais, os estudantes referem o tipo de personalidade forte, consciente e responsável; percebem como benefícios uma atitude positiva face ao preservativo, a comunicação e a capacidade de negociação do uso do preservativo com o parceiro e como

barreiras as crenças negativas sobre o preservativo, o embaraço na sua aquisição, a estabilidade da relação de namoro, mas sobretudo a confiança no parceiro. No acesso aos serviços de SSR, são apontadas como barreiras, a falta de garantia do anonimato e confidencialidade e a demora na marcação de consultas. Ao nível das influências interpessoais, os amigos têm um papel duplo, podendo ter um efeito protetor mas também potenciar o risco, e revelam-se importantes na tomada de decisão, os profissionais de saúde assumem um papel relevante, em particular para as raparigas. Ao nível das influências situacionais relacionadas com o contexto académico, apontam como influências negativas os consumos excessivos de álcool e drogas, associados a práticas sexuais inseguras, bem como a parceiros ocasionais. Nas propostas para melhorar a adesão a comportamentos sexuais seguros, que se poderiam constituir como linhas orientadoras da intervenção de enfermagem, os estudantes demonstraram dificuldades em encontrar estratégias específicas e bem definidas, apontando sobretudo para as estratégias educacionais em que focam a importância de ações mais intimistas que privilegiem a participação dos jovens numa relação de proximidade e cuidado com os técnicos de saúde e que, essencialmente, permita melhorar as respostas e a otimização dos serviços de saúde.

Conclusões: Os dados obtidos com os grupos focais permitem uma melhor compreensão da forma como os estudantes do ensino superior percecionam e vivenciam as questões relacionadas com os comportamentos sexuais (in)seguros. Os constructos que integram o HPM de Pender permitiram, entre outros, identificar variáveis importantes a considerar pelos enfermeiros no delineamento de estratégias de promoção da SSR para estudantes do ensino superior.

Palavras-chave: Comportamento sexual, estudantes ensino superior, modelo de promoção da saúde, enfermagem.

Abstract

Introduction: The promotion of sexual and reproductive health assumes particular importance in the younger age groups, in which greater autonomy is achieved and the first intimacy relationships are established. Admission to higher education may entail additional risks due to the academic lifestyle, where several sexual behaviors with negative outcomes on sexual and reproductive health (SRH) are described.

Objectives: To identify individual characteristics and current SSR behaviors; To know students' perceptions of benefits, barriers, situational and contextual influences, affects and self-efficacy, with implications for safe sexual behavior; Identify strategies that students consider relevant for the construction of SRH promotion programs for higher education students.

Methodology: A qualitative study was carried out using the focus group technique, involving 21 students from higher education, divided into 2 mixed groups. The Pender Health Promotion Model (HPM) was used as a theoretical and methodological reference in the interpretative analysis of the data, considering its three main components: individual characteristics and experiences, specific behavior, and behavioural outcome.

Results: Regarding the adoption of safe sexual behaviors, at the level of the individual characteristics the students refer to the strong, conscious and responsible personality type; they have a positive attitude towards the condom, communication and negotiation ability of condom use with the partner, and identify as barriers the negative beliefs about condoms, embarrassment in acquisition, commitment in the relationship and confidence in the partner. In the access to SRH services, they identified as barriers the lack of guarantee of anonymity and confidentiality and delay in the appointments. In terms of interpersonal influences, friends perform a dual role, which can have a protective effect but also increase risk, and are important in decision making. Health professionals play a relevant role, particularly for girls. At the level of the situational influences related to the academic context, they indicate as negative influences the excessive consumption of alcohol and drugs, associated to risky sexual practices, as well as the occasional partners. In the proposals to improve adherence to safe sexual behaviors, which could be used as guidelines for nursing intervention, students demonstrated difficulties in finding specific and well-defined strategies, pointing above all to educational strategies that focus on the importance of more intimate actions, that privilege a relationship of proximity and care with the health technicians and that essentially allows better answers and the optimization of the health services.

Conclusions: The data obtained from the focus groups provide a better understanding of how higher education students perceive and experience issues related to (un)safe sexual behavior. The constructs that integrate Pender's HPM allowed, among others, to identify important variables, to be considered by nurses, in the design of strategies to promote SRH for higher education students.

Keywords: Sexual and reproductive behaviour, college students, health promotion model, nursing.

Introdução

A promoção da saúde dos jovens deve ter como pressuposto básico dar resposta às suas necessidades específicas, particularmente no que diz respeito a questões fundamentais, como a saúde sexual e reprodutiva (SSR), procurando construir sociedades mais saudáveis e melhorando a sua qualidade de vida. Atualmente, reconhece-se que as universidades

podem ter um papel fundamental na promoção da saúde dos seus estudantes, criando aprendizagens, vivências mais saudáveis e promovendo o bem-estar e a saúde (Silva & Brito, 2014). No contexto do desenvolvimento sustentável e do aumento significativo dos comportamentos de risco para a saúde dos estudantes do ensino superior, que tem vindo a ser reportado em alguns estudos (Ghandour, Mouhanna, Yasmine & El Kak, 2014; Logan, Koo, Kilmer, Blayney & Lewis, 2015; Matos, Reis, Ramiro & Equipa Aventura Social, 2011; Wilton, Palma & Maramba, 2014), reafirma-se a importância de envolver as Universidades em projetos que visem a integração da saúde na sua missão, cultura, estruturas e modos de funcionamento, que se configuram como Universidades Promotoras de Saúde (Lara Flores, Saldaña Balmori, Férnandez Vera & Delgadillo Gutiérrez, 2015; Rocha, 2008).

Na sociedade ocidental, a juventude é marcada por acontecimentos normativos, tais como a transição para o ensino superior, que pressupõe a realização de duas tarefas nucleares deste período desenvolvimental, a construção da autonomia e da intimidade (Dias & Sá, 2013). A decisão de iniciar relações sexuais acontece paralelamente a várias modificações na vida dos jovens universitários, sendo que o exercício da sexualidade traz implicações no processo reprodutivo e na saúde biológica e psicossocial (Claxton & van Dulman, 2015). Alguns dos estudos sobre os comportamentos sexuais e reprodutivos em jovens universitários portugueses referem que um número significativo mantém comportamentos sexuais de risco (Matos et al., 2011; Oliveira, 2014; Pacheco, 2012), resultado da não utilização ou da utilização inconsistente do preservativo e da perceção de (in) vulnerabilidade face às IST (O'Sullivan, Udell, Montrose, Antoniello & Hoffman, 2010; Trieu, Bralton & Marshak, 2011; Wilton et al., 2014). Dentro dos fatores referenciados como potenciais obstáculos ao comportamento sexual seguro, a evidência sugere a eventualidade de o preservativo poder transmitir falta de confiança ao parceiro sexual, em particular no contexto de uma relação monogâmica e de maior duração (Manlove, Ikramullah & Terry-Humen, 2008), a perda de prazer sexual e da espontaneidade da relação (Gomes & Nunes, 2011), o embaraço na compra de preservativo (Cunha-Oliveira, Cunha-Oliveira, Pita & Massano-Cardoso, 2009) e o facto de se estar a usar outro método contracetivo.

Nesta perspetiva, a compreensão dos comportamentos sexuais dos jovens adultos constitui uma necessidade, do ponto de vista epidemiológico, à qual se associa a necessidade de conhecer as perceções, a racionalidade das escolhas e os fatores potenciadores do risco sexual (Ferreira & Cabral, 2010), visando definir estratégias de intervenção que promovam a adoção de comportamentos sexuais seguros, por esta população de jovens adultos.

Os comportamentos de risco e o estilo de vida são, atualmente, dois determinantes importantes da saúde dos jovens, pelo que, na promoção da saúde, para além da educação, devem considerar-se o desenvolvimento de competências pessoais e sociais e serviços de

saúde acessíveis, adequados e integrados numa resposta abrangente e adequada às suas necessidades (Direção-Geral da Saúde [DGS], 2008). Face ao exposto, no presente estudo procurou-se aprofundar o conhecimento e a compreensão sobre os comportamentos sexuais (in)seguros dos estudantes do ensino superior, propondo-nos: identificar as características dos comportamentos sexuais individuais e experiências; conhecer os sentimentos e perceções sobre os fatores que podem beneficiar ou dificultar o comportamento sexual seguro e identificar estratégias que os estudantes consideram relevantes na construção de programas de promoção da SSR para estudantes do ensino superior.

Modelo conceptual de análise

A utilização de modelos e teorias, no campo da promoção da saúde, pode facilitar a compreensão dos determinantes dos problemas de saúde, permitindo orientar as intervenções dos profissionais de enfermagem que visam a sua resolução tendo por base as necessidades identificadas. No presente estudo, foi preconizada a utilização do Modelo de Promoção da Saúde de Pender (Health Promotion Model – HPM*,* Pender, Murdaugh & Parsons, 2011), por se encontrar dentro dos modelos de promoção da saúde que centra o seu foco de atenção na intervenção de enfermagem, através de uma aproximação aos modelos comportamentais que procuram perceber a mudança de comportamento associada à saúde. Este modelo contempla ainda a natureza multidimensional dos indivíduos, na sua interação com os outros e com o ambiente na procura da saúde e bem-estar (Vítor, Lopes & Ximenes, 2005).

De acordo com Pender et al. (2011), o HPM pode ser usado para planear, implementar e avaliar ações de promoção da saúde, pelo estudo das interrelações de três componentes principais: (1) A primeira componente “Características individuais e experiências”, compreende o comportamento anterior, que carece ser mudado e os fatores pessoais (biológicos, psicológicos e socioculturais); (2) A segunda componente “Conhecimentos e sentimentos sobre o comportamento" que se pretende alcançar, compreende a perceção de benefícios e barreiras para a ação, que são representações mentais positivas ou negativas que fortalecem ou dificultam a decisão de adotar um comportamento de saúde, a autoeficácia percebida, que se relaciona com as capacidades pessoais de organizar e executar ações, os sentimentos positivos ou negativos subjetivos relacionados com o comportamento de saúde, as influências interpessoais, que incluem normas e apoio social e as influências situacionais, relacionadas com o ambiente onde a pessoa está inserida e que podem facilitar, ou impedir, determinados comportamentos de saúde;

(3) A terceira componente "Resultado do comportamento", que equaciona o compromisso com um plano de ação, as exigências imediatas e preferências, que são os comportamentos alternativos sobre o qual as pessoas têm um controlo reduzido, ou um grande controlo, e que permitem concretizar o comportamento de promoção da saúde (Pender et al., 2011; Sakraida, 2004; Vítor et al., 2005).

O HPM foi utlizado como referencial teórico na análise interpretativa dos dados, uma vez que permite compreender a complexidade dos comportamentos de saúde sexual, no sentido de delinear estratégias de intervenção em enfermagem, para promover a SSR dos estudantes e a mudança comportamental.

Metodologia

Foi realizado um estudo de natureza qualitativa, com recurso à técnica de grupos focais (*Focus Group)*. Este procedimento metodológico dos grupos de discussão focalizada caracteriza-se por permitir uma forte interação dos grupos na problematização de temáticas sensíveis. Este tipo de abordagem permitiu perceber e explorar os significados atribuídos pelos participantes ao tema em estudo, uma vez que os comportamentos sexuais, que entram no domínio do íntimo e do privado, devem ser entendidos numa dinâmica, onde a interação humana assume um papel fundamental (Oliveira, 2011). Esta metodologia tem vindo, então, a ser utilizada com sucesso no contexto das ciências sociais, das ciências da saúde e da enfermagem, para estudar questões relacionadas a temas complexos, multifacetados (Jayasekara, 2012) e de natureza íntima (Oliveira, 2011), incluindo a investigação de questões relacionadas com os comportamentos sexuais seguros (Lobdell, Gilboa, Mendola & Hesse, 2005; Oliveira, 2011; Peterson-Sweeney, 2005).

A pertinência desta metodologia para estudar os comportamentos de saúde sexual e reprodutiva é confirmada por Dias e Rocha (2009), quando afirma que esta técnica de recolha de dados tem subjacente a necessidade de se compreender as múltiplas variáveis que contribuem para os complexos, e muitas vezes contraditórios, significados, crenças, atitudes e comportamentos de risco e de proteção, relacionados com a SSR, construídos num contexto de elementos sociais, económicos e culturais da realidade específica em que cada indivíduo está inserido.

Planeamento e instrumento de recolha de dados

No processo de planeamento foram tidos em consideração os objetivos de estudo, bem como algumas das orientações de autores de referência (Billups, 2012; Hyde, Howlett, Brady & Drennan, 2005; Krueger & Casey, 2009; Morgan & Krueger, 1998), no que respeita aos critérios de seleção dos participantes, o instrumento de recolha de dados, o local de

realização das sessões, o processo de recrutamento dos estudantes e o incentivo à participação. Dos 21 estudantes que acederam voluntariamente a participar no estudo, foram constituídos dois grupos mistos, o primeiro com 10 estudantes (7 raparigas e 3 rapazes) e o segundo com 11 estudantes (9 raparigas e 2 rapazes), procurando assegurar não só a representatividade do género mas também a área científica de estudos. Os grupos focais decorreram numa sala de reuniões da universidade, e na preparação do espaço procuramos que o local fosse confortável, privado, que permitisse uma organização circular, de forma a favorecer a dinâmica do grupo. A dinâmica decorreu na presença e na interação desenvolvida entre todos – moderadoras e participantes – e cada sessão demorou, em média, duas horas.

Os grupos focais tiveram início com o acolhimento dos participantes, a apresentação da equipa de investigação e dos objetivos do estudo e foram enfatizadas algumas “regras” de participação. A realização dos grupos focais foi precedida de uma apresentação individual e de uma atividade “quebra-gelo”, solicitando-se ao grupo que identificasse, numa folha de papel, as palavras que na sua opinião se enquadravam no conceito de saúde reprodutiva e no conceito de saúde sexual, explicando sucintamente o motivo da sua escolha. Esta atividade foi muito participada e, na discussão, os estudantes apresentaram algumas reflexões sobre os conceitos em análise. Para a recolha de dados elaborou-se previamente um conjunto de questões norteadoras do tema em estudo, e que favorecessem a condução dos grupos focais, o que acabou por se constituir num instrumento essencial de orientação do foco da discussão. No final das sessões foi ainda aplicado o questionário elaborado para a caracterização dos participantes aos níveis: sociodemográfico (género, idade, proveniência), académico (tipo de curso e ano de curso) e dos comportamentos sexuais e reprodutivos (têm relações sexuais, utilização de contraceção, utilização do preservativo na última relação sexual, realização de vigilância de saúde). Cada sessão foi registada em vídeo e áudio, após o consentimento prévio dos participantes, de forma a maximizar a recolha de dados e a permitir a transcrição integral das discussões realizadas, para análise posterior. Finalizados os grupos de discussão focalizada, procedeu-se ao registo das observações sobre a dinâmica e interação dos participantes nos dois grupos e transcrição integral da informação recolhida.

Participantes no estudo

O grupo que participou no estudo foi constituído de forma voluntária, resultando em 21 estudantes, 16 do género feminino e 5 do género masculino, com idades compreendidas entre os 19 e os 27 anos, e uma média de idades de 21 anos. Os participantes frequentavam diversas áreas científicas dentro dos cursos disponibilizados pela

universidade e a maioria frequentava o 2.º e o 3º ano da licenciatura. Relativamente aos comportamentos de SSR, a maioria (16 estudantes) referiu ter relações sexuais e todos afirmavam usar contraceção. Dos métodos de contraceção utilizados, 8 estudantes usavam o preservativo, 4 a pílula e 9 a dupla contraceção. Metade dos estudantes (12) refere ter utilizado o preservativo na última relação sexual. Quanto à vigilância de SSR, 14 estudantes realizam vigilância, e dos 7 que não realizam, 4 são do género masculino.

Procedimentos da análise interpretativa dos dados

O processo de análise dos dados pressupôs uma abordagem qualitativa (Bardin, 2004; Krueger & Casey, 2009) com o intuito de dar conta de dimensões mais subjetivas, nomeadamente as perceções e os sentimentos dos estudantes. O *corpo* de análise resultou da transcrição integral da informação e o texto foi sujeito a uma leitura “flutuante” e de análise exploratória e indutiva do material, de acordo com os objetivos específicos. Após as transcrições, obtivemos um conjunto de características, relatos de comportamentos ilustrativos do conhecimento que os jovens interiorizaram sobre a problemática, o que nos permitiu sinalizar um conjunto de unidades de registo, passíveis de serem interpretadas à luz do quadro teórico de referência do HPM de Pender (Pender et al., 2011). Com este modelo de análise, procuramos compreender os comportamentos relacionados com a promoção da SSR, pelo estudo das interrelações de vários fatores relacionados com o risco e a proteção a que os jovens adultos estão expostos no âmbito da atividade sexual, designadamente:

i) *Características individuais e experiências*, que compreendem os fatores individuais (biológicos, psicológicos e socioculturais) e o comportamento sexual e reprodutivo anterior.

ii) *Conhecimento e sentimentos sobre o comportamento,* que se pretende alcançar e que no presente estudo foi conceptualizado como sendo o comportamento sexual seguro e avaliado pelas seguintes variáveis: perceção de benefícios e barreiras para a ação, perceção de autoeficácia, sentimentos, influências interpessoais (família, pares e profissionais de saúde) e situacionais (uso de álcool e drogas associado às relações sexuais e tipo de acesso aos serviços de SSR).

iii) *Comportamento promotor de saúde*, que no presente trabalho foi avaliado pela perceção dos estudantes sobre as estratégias mais importantes a integrar num plano de ação para promoção de comportamentos sexuais seguros em estudantes do ensino superior.

No sentido de agilizar o processo de análise, foi elaborada uma tabela, integrando cada uma das componentes de análise, onde foram incluídos excertos dos discursos, considerados como ilustrativos dos significados atribuídos pelos estudantes, depois de numerados e

identificados (cada estudante é referenciado pela letra E, seguida da numeração do grupo focal que integrou e respéctivo número de identificação individual, e.g. E1.1).

Considerações éticas

O documento de consentimento informado respeitou as regras de conduta propostas pela *Declaração de Helsínquia*, e depois de devidamente informados sobre os objetivos do estudo, todos os participantes assinaram o consentimento livre e esclarecido, expressando a sua disponibilidade em participar no estudo. O estudo foi aprovado pela Comissão de Ética da Universidade onde decorreu a investigação (Parecer nº 2/2012).

Análise interpretativa dos dados

Características individuais e experiências

Atendendo aos pressupostos teóricos do HPM, esta componente integra duas variáveis, o comportamento anterior e os fatores pessoais, que permitem ao enfermeiro planear cuidados de promoção de comportamentos sexuais seguros.

Considerando o comportamento sexual anterior, todos os estudantes são sexualmente ativos e tiveram a sua primeira relação sexual, em média, cerca dos 17 anos. *V*ários estudos têm revelado que a idade de iniciação sexual tem vindo a aumentar na última década, com o género feminino a registar limites etários superiores (Matos et al., 2011; Oliveira, 2014; Pacheco, 2012). As raparigas continuam a demonstrar uma orientação mais responsável e idealista da sexualidade, retardando o início da vida sexual (Antunes, 2007).

Os motivos apontados para iniciarem as relações sexuais em determinado momento das suas vidas são a maior interação entre jovens de diversas idades, e sugerem a procura de afirmação no grupo de pares ou mesmo a pressão social para se tornarem sexualmente ativos.

> *“Há mais grupos mistos, digamos, não no sentido de serem rapazes e raparigas mas de diferentes idades […]”* E1.2
>
> *“Eu acho que há cada vez mais isso, e há necessidade de afirmação no grupo.* E1.3
>
> *“Não é só a pressão dos pares, é a pressão no geral e da sociedade…”* E2.1

No que respeita às práticas contracetivas, todos os participantes referem usar algum método de contraceção, em particular o preservativo ou a dupla contraceção (pílula/preservativo). A aquisição destes métodos é realizada, preferencialmente, no centro de saúde e IPJ, como referem:

"A mim fornecem-me no centro de saúde, dão-me um envelope lacrado com 30 preservativos de 3 em 3 meses." E 2.2

"No meu caso, vou buscar a pílula ao instituto de que falei, o IPJ." E1.2

Conforme documentado em diversos estudos com estudantes universitários (Borges, Fujimori, Hoga & Contin, 2010; Gomes & Nunes, 2011; Matos et al., 2011), o preservativo e a pílula continuam a ser os métodos de contraceção mais utilizados, mas existe um número significativo de estudantes que começa a utilizar a dupla proteção, conforme preconizado por alguns organismos internacionais de referência na área, como o método mais efetivo de prevenção de IST e da gravidez indesejada nesta faixa etária (Lopez, Otterness, Chen, Steiner & Gallo, 2013).

Na escolha do método contracetivo prevalece uma visão tradicional, entendendo os estudantes que a *"A contraceção é responsabilidade das raparigas".* E2.6, ainda que algumas estudantes reafirmem a importância desta decisão ser partilhada pelo casal e, nesse sentido, se pronunciaram:

"No meu caso, decidimos os dois em conjunto. Tem de ser, a responsabilidade é dos dois se não houver um preservativo". E1.3

"Considero que o homem tem um papel tão importante como a mulher nesse aspeto do controlo e prevenção da sua saúde, incluindo a contraceção". E2.4

No que diz respeito à contraceção de emergência (CE), a perceção do grupo é que este método é muito usado, e muitas vezes de forma recorrente ou mesmo "*irresponsável*". No seu discurso, algumas estudantes demonstram, inclusivamente, alguma preocupação com os potenciais efeitos nocivos na saúde, decorrente desta utilização recorrente, e mencionam que:

"Sim está a usar-se mais" (referem vários estudantes em coro) e "Cada vez se está a usar mais…e em idades mais precoces". E2.1

"Eu continuo a achar que os jovens pensam que uma vez não faz mal […] para eles, está tudo bem…." E2.6; e uma estudante afirma mesmo que *"Deviam proibir a venda."* E1.7

O último estudo de âmbito nacional da SPG e SPC (2015) revelou que é na faixa etária dos jovens adultos (20-24 anos) que esta percentagem de utilização de CE é mais elevada, situando-se nos 90%. No mesmo sentido, Borges e colaboradores (2010) afirmam que um maior nível de escolaridade não implica, necessariamente, um comportamento contracetivo mais responsável, uma vez que, no seu estudo, cerca de 50% dos estudantes universitários já tinha utilizado a CE. Utilização que estava relacionada sobretudo com a inconsistência no uso de métodos contracetivo regular e as motivações para não engravidar. A este respeito

devemos salientar que, embora a CE não substitua os métodos de contraceção regular, até porque tem menor eficácia contracetiva, a evidência mais recente sugere que não existem contraindicações à sua utilização (Barros & Neves, 2013; International Consortium for Emergency Contraception [ICEC], 2015).

Tradicionalmente, a juventude é percecionada como uma etapa de desafios e experimentação, mas o exercício da sexualidade, além de proporcionar novas emoções e sentimentos, pode comportar riscos para a saúde. Neste sentido, os estudantes apontam para características individuais que podem estar relacionadas com a adoção de comportamentos sexuais seguros, tais como *"A personalidade forte é muito importante mesmo." E2.3.,* assim como *"A consciência, a responsabilidade".* E1.6

Alguns estudos nesta área sustentam que os tipos de personalidade podem estar associados a diferentes atitudes e comportamentos sexuais. Os jovens com maiores níveis de extroversão revelaram-se galanteadores e isentos de recato afetivo, enquanto os jovens com valores altos de neuroticismo revelam mais nervosismo, culpa, inibição e insatisfação, manifestam-se por isso, mais inibidos em relação a comportamentos sexuais socialmente interditos (Antunes, 2007). Nas últimas décadas, tem existido um interesse crescente no papel das disposições da personalidade para a busca de sensações sexuais enquanto mediador dos comportamentos de risco sexual (Voisin, King, Schneider, Diclemente & Tan, 2012).

No presente estudo, os comportamentos sexuais de risco em estudantes universitários são percecionados como frequentes e associados maioritariamente ao género masculino. Os estudantes apontam como comportamentos sexuais não seguros o sexo sem preservativo e os parceiros ocasionais, mencionando:

> *"Acho que os principais são mesmo o sexo desprotegido, com vários parceiros, e que aumenta muito o risco."* E1.5
>
> *"Começam muito cedo e chega a uma altura em que querem arriscar mais, não chega um só companheiro, e mais do que um, dois ou três…"* E2.4
>
> *"Porque sei que há muitos amigos meus aqui na universidade, que tipo são capazes de conhecer a rapariga hoje e bebem uns copos e depois de manhã acordam com ela…que cena…e depois tinham preservativo?"* E1.4
>
> *"E mesmo nas relações de longa duração não são bem assim […], pelo meio existem sempre alguns parceiros ocasionais."* E2.1

Na juventude, o sexo ocasional, fora das relações românticas tradicionais, é cada vez mais comum e socialmente aceite, sendo entendido como uma forma de se explorar a sexualidade e intimidade sem se investir numa relação amorosa (Kuperberg & Padgett,

2015). Nesta perspetiva, alguns autores têm vindo a assinalar o surgimento de um fenómeno cada vez mais normativo entre estudantes universitários, o da cultura do "*hook-hoop*", que em português pode ser entendida como "engate". Esta prática, caracterizada por breves encontros sexuais entre indivíduos que não têm uma relação de namoro, representa uma mudança social significativa na aceitação do sexo sem compromisso (Claxton & van Dulman, 2015; Kuperberg & Padgett, 2015). Uma revisão da literatura sugere que os encontros sexuais deste tipo são frequentes, com 60% a 80% dos estudantes universitários norte americanos a referir algum tipo de experiência com práticas sexuais deste tipo (Garcia, Reiber, Massey & Merriwether, 2012). Esta prática sexual pode comportar um risco acrescido, uma vez que, em muitas situações, não é utilizado o preservativo e os indivíduos são mais propensos a ter múltiplos parceiros sexuais (Lewis, Granato, Blayney, Lostutter & Kilmer, 2012). No estudo realizado por Lewis et al. (2011), estes verificaram que dos 1468 estudantes universitários participantes, 29,2% refere ter tido sexo com parceiros ocasionais e, destes, apenas 46,6% usou preservativo.

Refletindo sobre a normalização deste fenómeno, um dos estudantes afirma *"Inclusive agora vemos nas gerações mais velhas aquelas trocas de casais [...], na nossa geração também acontece, mas às claras e de forma consentida, por aventura. Os jovens já consideram isto como um comportamento normal…".* E2.4, ou mesmo banalizado, nas suas palavras:

> *"Ter relações sexuais também dá estilo. Quantas é que foram esta noite? Ouvimos muitas vezes, estás maldisposta, isso é falta de sexo".* E2.1

O abandono do preservativo está associado ao facto das raparigas realizarem contraceção hormonal oral, apontando os resultados para uma preocupação muito clara com a gravidez indesejada, argumentando que:

> *"Com o meu namorado a minha preocupação não é se tem uma doença ou não, é mesmo a gravidez".* E1.1

> *"Acho que é mesmo a questão de prevenir a gravidez".* E1.3

> *"Eu acho que a maior parte dos jovens pensa que os métodos contracetivos só servem para não se engravidar".* E2.1

Estes resultados corroboram os encontrados por diversos estudos, onde se verifica que o receio de engravidar continua a ser, entre os estudantes universitários, o principal fator motivacional para usar o preservativo (Goldstein, Upadhyay & Raine, 2013). A pouca importância atribuída pelos jovens ao preservativo na prevenção de IST, permite que se perpetuem as elevadas taxas de infeção, em particular entre os jovens adultos (Kann et al., 2016).

Na generalidade, os estudantes recorrem pouco aos serviços de SSR e utilizam preferencialmente ao centro de saúde e IPJ *"Médica de família, qualquer dúvida, médico de família."* E1.1*,* e o IPJ essencialmente para adquirir métodos contracetivos *"Eu continuo a ir ao IPJ porque sinto que tenho um bom acompanhamento. Eu vou apenas buscar a pílula e assim."* E1.2

Conhecimento e sentimentos em relação aos comportamentos sexuais seguros

Esta segunda componente do HPM integra os sentimentos subjetivos, a autoeficácia, a perceção de benefícios e barreiras e as influências interpessoais e situacionais relacionadas com o ambiente onde a pessoa está inserida e que podem ter influência nos comportamentos de saúde.

No que respeita aos sentimentos, os estudantes fazem referência a alguns medos e preocupações que condicionam a vivência da sexualidade em "plena liberdade", salientando em particular o medo da gravidez indesejada "*Ficar grávida na nossa idade, acho que é uma das maiores preocupações*". E1.7, e a aquisição de doenças. "*A mim o que me assusta são as doenças, sempre que ouvimos esses números dos 70% de pessoas infetadas com HPV*." E1.1

Paradoxalmente, alguns estudantes têm a perceção que os jovens adotam uma postura de despreocupação, comodismo e desinteresse, não só no que diz respeito à prevenção de comportamentos de risco sexual, mas também nas questões de vigilância de saúde:

> *"É o deixa-andar, afinal nunca tive nada…"* E1.4
>
> *"Eu acho que é complicado chegar à cabeça dos jovens hoje em dia. Acho que é muito comodismo."* E1.3

A literatura sustenta que elevado nível de autoeficácia potencia o uso consistente do preservativo e a capacidade de negociação do sexo seguro (Artístico, Oliver, Dowd, Rothenberg & Khalil, 2014; French & Holland, 2013). No entanto, apesar do discurso sobre o preservativo ser positivo, alguns estudantes revelam uma baixa perceção da autoeficácia para o usar, referindo que, *"Apesar de termos aquela convicção, preservativo sempre. Nós temos essa noção só que depois as coisas na prática são diferentes [...], "* E2.1*,* transparecendo nos seus discursos que a intenção de usar o preservativo nem sempre é concretizada e que pode ter consequências: *"Sim, só na hora conseguimos decidir se usamos ou não, e nem sempre usamos".* E2.8*. "Mesmo sabendo que é falta de responsabilidade […] mas no calor do momento, às vezes não tomamos a decisão certa".* E2.1

Da análise do discurso dos estudantes surgem como benefícios percebidos para a adoção de comportamentos sexuais seguros as atitudes positivas face ao preservativo, a

comunicação, a capacidade de negociação do uso do preservativo com o parceiro sexual e o conhecimento sobre o passado sexual do parceiro.

A evidência sugere que uma atitude mais favorável ao preservativo é preditora do seu efetivo uso (Asare, 2015). Em ambos os grupos, os participantes revelaram uma atitude favorável à utilização do preservativo, associando o seu uso a uma prática protetora da gravidez, como já mencionado anteriormente, mas também de IST, opinião que é clara nos seus discursos.

> *"Temos de usar, no meu caso, conheço pessoas que já apanharam doenças, já aqui na universidade […], eu tomo a pílula, mas mesmo assim eu prefiro usar preservativo, mais vale segurança a dobrar que nenhuma."* E1.1

> *"[…] Nem o preservativo por si só é seguro a 100%, por isso mais vale usar os dois e ver se dá maior proteção. O preservativo, para além de evitar as doenças, evita a gravidez, eu acho que a partir do momento em que temos uma relação temos de o usar sempre."* E1.3

> *"[…] eu uso sempre o preservativo e considero que tem de estar sempre próximo do rapaz. Devemos usar sempre para proteção."* E2.4

> *"Podemos não ficar grávidas mas as doenças existem…"* E2.9

Do mesmo modo, percecionam como importante na adoção de comportamentos sexuais seguros a comunicação com os parceiros sexuais sobre o uso do preservativo e o acordo quanto à sua utilização:

> *"Eu acho que a comunicação deve estar sempre presente e a prevenção também."* E2.4

> *"Eu sim, eu converso, eu partilho, tem de ser, senão não me sentiria à vontade na relação."* E2.3

> *"Sim, porque se ele não tem preservativo ela não tem de aceitar fazer sem preservativo… No meu caso, ou usa o preservativo ou não acontece nada."* E1.5

Num estudo com estudantes universitários, Martins, Nunes, Muñoz-Silva e Sanchez-Garcia (2008) observaram as dificuldades dos estudantes em conversarem com os seus parceiros sobre a utilização do preservativo. As dificuldades de comunicação estavam relacionadas com a baixa percepção de risco, a espontaneidade do sexo, a escolha deliberada de não usar preservativo e a baixa autoeficácia para negociar a sua utilização. A este propósito, O'Sullivan e colaboradores (2010) afirmam que ideais românticos associados à espontaneidade da relação, bem como a dificuldade em transmitir a ideia de que existem outros parceiros, revelam-se elementos importantes das atitudes face ao preservativo e preditores do seu uso.

O conhecimento do passado sexual do parceiro, foi também percebido como um benefício em ambos os grupos,

> *"Eu coloco tudo em pratos limpos o que é que eu fiz, o que não fiz, com à vontade, […] e não vou já entrar em maluquices […] e depois tento saber sempre o que ela já fez ou não fez."* E1.4

> *"[…] eu fui a primeira a pôr todas as minhas dúvidas em cima da mesa, já que ele já tinha tido outras relações, e esclareci tudo, considero isso fundamental."* E2.3

No entanto, de acordo Manlove et al. (2008), a discussão da história sexual do parceiro não resulta necessariamente na negociação de práticas sexuais mais seguras, criando mesmo, muitas vezes, uma segurança ilusória e substituindo os comportamentos de proteção, pois muitas vezes os parceiros sexuais ocultam a verdade e subestimam o risco. Horan (2016), realizou um estudo com estudantes universitários Norte Americanos onde observou que cerca de 60% dos participantes, mentiram aos seus parceiros atuais sobre os comportamentos sexuais anteriores, e destes, quase 20% ocultaram o número de parceiros sexuais anteriores.

No presente estudo, a maioria dos estudantes entendeu ser legítimo o questionamento do parceiro sexual sobre a realização do teste VIH, incluindo o conhecimento sobre o estado serológico, mencionando que não se importariam de realizar o teste.

> *"Já perguntei…sim, é bom deixar tudo em aberto."* E1.4

> *"Eu acho que isso é cada vez mais importante questionar sobre isso, e realizarmos o teste os dois."* E1.3

Os resultados do presente estudo divergem dos observados por Matos e colaboradores (2011), onde uma elevada percentagem de estudantes universitários referiu que não tinha intenção de realizar o teste para o VIH (53,1%) nem tencionava pedir ao parceiro para o realizar (50,7%). O teste de despistagem do VIH pode ser entendido como um sinal de falta de confiança, parecendo sugerir que o parceiro teve no passado comportamentos sexuais de risco (Alvarez & Oliveira, 2007; Cunha-Oliveira et al., 2009).

Foram percecionadas como barreiras para adoção do comportamento sexual seguro as crenças negativas sobre o preservativo, o embaraço na aquisição do preservativo e os custos associados aos mesmos, o estatuto da relação amorosa, fatores condicionantes o acesso aos serviços de saúde e o conhecimento que detêm sobre sexo seguro.

No que respeita à utilização consistente do preservativo, persistem ainda crenças negativas, em particular por parte dos rapazes. Todos os rapazes argumentam que *"Dá mais prazer sem preservativo. Qualquer preservativo retira algum prazer à relação sexual"* E1.5, que o

preservativo diminui a sensibilidade, corta a espontaneidade do momento, e que pode mesmo constituir *"[…] uma barreira entre o casal."* E2.4

Nas últimas décadas, na área da sexualidade e em particular na sexualidade juvenil, valoriza-se cada vez mais o prazer, a experimentação e até alguma transgressão. Admite-se uma grande diversidade de comportamentos sexuais, com tendência para uma maior valorização da dimensão erótico-hedonista da sexualidade (Antunes, 2007). A este propósito, Maia (2010) argumenta que a valorização do prazer em detrimento da prevenção tem influência sobre a perceção de risco, uma vez que o preservativo está frequentemente associado à diminuição do prazer sexual e raramente associado ao imaginário erótico, comprometendo a utilização do mesmo. No entanto, a perceção do risco individual para a possibilidade de contrair uma IST é fundamental para compreendermos o que motiva os jovens a envolver-se em comportamentos sexuais de risco (Cunha-Oliveira, 2008).

Uma outra barreira importante na adoção de comportamentos sexuais seguros é o estatuto do relacionamento amoroso, justificando os participantes o abandono do preservativo pela estabilidade da relação e confiança no parceiro, afirmando os estudantes que:

> *"Os casos que eu conheço, que dispensam a utilização do preservativo, são os que já têm uma relação de muito tempo."* E1.2

> *"Ela pedir para não usar o preservativo? Já aconteceu… mas comigo é diferente… já há uma base de confiança."* E1.4

> *"Eu já ouvi muitos colegas a dizer que após um ano de namoro, usar preservativo é falta de confiança no parceiro."* E2.8

> *"[…] se fosse com um parceiro de há muito tempo, eu não usaria o preservativo, se calhar não usaria.* E2.3

São diversos os estudos que encontram uma estreita relação entre as relações com um parceiro estável e a não utilização do preservativo e a sua maior utilização com um parceiro casual. A denegação do risco é acompanhada pela crença no afeto, romance, fidelidade e confiança mútua como garantia de proteção, como se existisse uma incompatibilidade entre o sentimento amoroso e o risco (Maia, 2010).

No presente estudo, dois estudantes revelam um discurso crítico face a esta lógica da proteção, baseada na confiança no parceiro, alegando que "*Eu não penso deste modo de forma nenhuma* […], *usar o preservativo não é uma questão de confiança é uma questão de saúde. Sem dúvida de saúde*" E2.6, posição que é reafirmada por outro participante quando alega "*Eu nunca tive nenhuma relação sexual sem ser com preservativo, nem naquelas relações mais longas […], mesmo quando sentia confiança, não era a confiança que me fazia deixar de usar.*" E2.4

A relevância do conceito de confiança no universo das relações afetivas e sexuais aparece pioneiramente nos trabalhos de Giddens desde a década de 1980, tendo sido usada para analisar os processos histórico-culturais na constituição das relações íntimas contemporâneas. Segundo este autor (Giddens, 2002), a confiança é necessária à sociedade moderna, uma vez que esta resulta das mudanças nas relações sociais e que só pode existir em uma relação que proporcione risco. A confiança contribui para desenvolver um sentido de invulnerabilidade, uma vez que cada parceiro é "único" e o relacionamento entre ambos é acompanhado de emoções, e preocupação mútua, as quais ultrapassam as questões de proteção individual (Alvarez & Oliveira, 2007). Esta perceção também transparece no discurso dos estudantes:

> *"[…] eles não tinham essa preocupação (com as doenças) porque diziam, eu já tive várias raparigas mas foi sempre a primeira vez para elas, por isso elas não podiam ter nenhuma doença".* E2.1

Refletindo sobre o lugar da confiança no universo das relações amorosas, Oliveira (2015) afirma que a confiança aparece como um discurso elaborado que pretende justificar a ausência de métodos de proteção nas práticas sexuais, relacionada com a expectativa de fidelidade conjugal. A manutenção de uma relação exclusiva justifica um modelo de fidelidade valorizado pela sociedade, que, por sua vez, sugere uma confiança recíproca. Deste modo, a negação do risco e o não uso do preservativo significam uma demonstração de confiança no parceiro sexual.

De acordo com a maioria dos participantes, também o embaraço na aquisição do preservativo pode representar uma barreira à adoção de comportamentos sexuais seguros. Uma das estudantes alega que *"Mesmo tendo esse acesso e mesmo tendo à vontade para falar sobre isso eu não seria capaz de pedir ou comprar preservativos"* E2.3

Esta dificuldade é apontada tanto por rapazes como raparigas, pronunciando-se a maioria neste sentido:

> *"[…] as pessoas ainda olham de lado, eu já fui comprar e a mulher ficou a olhar para mim, do género: a rapariga a comprar?"* E1.2
>
> *"Eu acho que ainda há aquela vergonha… principalmente da parte masculina, embora nas raparigas também."* E2.6
>
> *"No meu caso também acho uma situação um bocado constrangedora."* E2.5

Contudo, duas das estudantes reconhecem que a vergonha e o embaraço não podem ser condicionantes para a aquisição do preservativo, dado que estes podem ser adquiridos de forma fácil e anónima em diferentes locais, referindo uma das participantes que *"Agora*

consegue-se adquirir preservativos em qualquer sítio, até mesmo na semana da queima, e esses são grátis.” E2.10

O acesso aos serviços e cuidados de saúde revela-se de extrema importância para diminuir as vulnerabilidades e promover a SSR nas comunidades. Contudo, os estudantes reconhecem que “*Não, no geral os jovens não recorrem, tem um bocado de vergonha, sentem-se intimidados.”.E1.3* e reportam algumas barreiras no acesso aos serviços e cuidados de SSR, nomeadamente as questões de garantia do anonimato e confidencialidade da informação, relacionadas com o facto de viverem numa cidade pequena, onde muitas pessoas se conhecem “ Em meios pequenos como o nosso há muitos boatos”. E1.9, “[…] *lá na minha zona todos os enfermeiros e médicos conhecem a minha mãe, é um bocado complicado.“* E1.3, não sendo possível ocultar o motivo da consulta “*Nós vamos lá e toda a gente sabe que fomos lá por causa disto, por causa daquilo.”* E1.10, no mesmo sentido, outra estudante acrescenta, *“Sim e até a maneira como nos olham…”. E1.5.*

Relativamente às influências interpessoais, considerando os diferentes agentes de socialização (grupos de pares/amigos, família e profissionais de saúde), compreendemos níveis de valorização diferenciados entre rapazes e raparigas.

Os jovens são particularmente sensíveis às influências dos pares, às normas partilhadas pelo grupo e pela cultura que está subjacente, aspectos que moldam de forma significativa as respostas individuais, em termos de sexualidade. Os amigos e grupo de pares também se revelaram uma influência importante para os jovens, em particular na partilha de conhecimento na área da sexualidade, tal como ilustram as seguintes narrativas:

> *“A minha primeira fonte de informação foi o grupo de pares, o meu grupo de colegas”. Podia não ser a informação mais fidedigna, mas partia sempre dai. Depois, claro, tentava pesquisar onde podia.”* E2.4
>
> *“A maneira mais fácil é com aquela amiga. Ok, tudo bem que ela não é um profissional de saúde mas normalmente vai ajudar de alguma forma.”* E2.5
>
> *“com as minhas amigas não falo da parte da saúde”. E.1.1*

Estes dados são concordantes com os de diversos estudos nacionais, onde os amigos constituem a principal fonte de informação sobre sexualidade, em cerca de 60% dos casos (Cunha-Oliveira et al., 2009; Matos et al., 2011).

No entanto, alguns estudos também têm documentado que beber excessivamente pode estar associado à influência dos pares, e que a associação do álcool às relações sexuais pode potenciar comportamentos sexuais de risco (Guo, Li, Owen, Wang & Duncan, 2015; Wechsler, Xuan, Lee, Weitzman & Wechsler, 2009). No presente estudo, os participantes reconhecem esta dupla influência do grupo, mencionando que *“O grupo, se for um bom grupo*

também pode ajudar, um grupo que aceite todas as diferenças dos pares." E2.8, mas também potenciar o risco, referindo os estudantes que *"A pressão dos pares associa-se muito também ao consumo excessivo de álcool que é cada vez mais frequente"* E2.4

Embora sendo consensual que a família é o espaço emocional privilegiado para o desenvolvimento de atitudes e comportamentos saudáveis na área da sexualidade, pelo apoio na construção da sua autonomia e na tomada de decisões, a maioria dos participantes refere que não conversa com os pais sobre questões de sexualidade, pronunciando-se a maioria nesse sentido:

> *"Eu não tenho intimidade para falar com os meus pais, para falar com eles diretamente."* E2.7
>
> *"Eu não falo com os meus pais ..., os meus pais não estão preparados para abordar estes assuntos."* E1.9

As estudantes reconhecem nos profissionais de saúde, uma fonte de informação credível e de confiança sobre as questões da sexualidade, em particular as raparigas, valorizando a possibilidade de poderem falar abertamente e partilhar dúvidas com estes profissionais, a título ilustrativo:

> *"Dúvidas mesmo e coisas importantes é mesmo com os profissionais de saúde [...] quando tenho dúvidas sobre alguma coisa têm de ser mesmo com o enfermeiro, com quem posso esclarecer dúvidas e falar sobre tudo."* E2.2

Contudo, o recurso a profissionais e serviços de saúde por parte dos estudantes é reduzido. Em algumas situações justificado pelo facto de *"Se calhar esta não é a forma mais correta, achei sempre que as minhas amigas e a internet, era suficiente".* E2.5. Neste sentido, Vilar e Ferreira (2009), afirmam que os jovens podem apresentar alguma relutância na utilização de serviços de saúde com os quais não têm uma relação habitual, o que dificulta o estabelecimento de contactos de proximidade.

A maioria dos estudantes considerou como muito importante a informação transmitida sobretudo pelos profissionais de saúde, afirmando que *"[...] esclareci muitas dúvidas que tinha e com frequência quando as tenho ou me acontece alguma coisa é lá que vou (centro de saúde)."* E2.3, *uma das estudantes acrescenta " Acho também importante referir que nós temos os serviços de ação social aqui da universidade [...]. Eu já tive acesso e eles são sem dúvida uma ajuda" E2.1,* mas também pelos pais, em particular pela mãe, mencionando que *"[...] deu-me estabilidade, não só a nível emocional mas uma maior segurança para poder agir e tomar decisões."* E2.6

Todavia, alguns estudantes reconhecem que têm lacunas de conhecimento, sobretudo em relação às IST, como revelam os seus discursos:

"Fala-se de SIDA e papiloma humano, mas mais nada." E1.3

"E o HPV, é o papiloma qualquer coisa…" E1.4

"*Sim mas existe a Hepatite, SIDA, HPV…e aquelas mais raras…a gonorreia e a sífilis, essas coisas assim*." E2.1

Tais resultados são coincidentes com os de outros estudos de nível nacional, onde cerca de um terço (31%) dos jovens do ensino superior afirmou não se sentir informado, sobre temas como as IST (60%) e contraceção (48,5%) (Reis, Ramiro, Matos & Diniz, 2013).

Ao nível das influências situacionais, os estudantes apontam como influências negativas e que podem impedir a adoção de comportamentos sexuais seguros, o crescente consumo de substâncias psicoativas (álcool e drogas) associado às relações sexuais, e os parceiros ocasionais em particular durante as festas académicas e noite em geral *" Sim a noite, festas académicas, discotecas, bares…"* E2.6, referindo:

"Consome-se muito álcool e por vezes outras coisas…para além do álcool. Mistura-se muita coisa sim, sim…" E1.5

"Mesmo assim, eu ainda acho que é o álcool que se utiliza mais, apesar de estar a aumentar o tráfico de drogas seja cada vez mais fácil aceder a drogas, ainda assim acho que o álcool é mais problemático." E2.2

"Nesses momentos uma pessoa alcoolizada não vai pensar em responsabilidades […], e acontecem relações sexuais em que por vezes não há nada, nem pílula, nem preservativo".E2.4

" Na queima, sim sexo ocasional nas casas de banho… em público… forte". E1.3

" É aquela ideia, uma vez não faz mal, com ela não faz mal, com ela também não, para eles está tudo bem…". E2.6

Embora seja difícil estabelecer uma relação entre drogas ilícitas e o comportamento sexual dos jovens adultos, vários autores vêm documentando que o sexo casual é um comportamento cada vez mais frequente entre os estudantes universitários, em particular no contexto das festas académicas e está muitas vezes associado ao consumo excessivo de álcool e drogas (Pollock, 2016; Uecker, 2015; Wilton et al., 2014). Alguns estudos nacionais têm revelado esta associação, uma vez que que cerca de um terço dos estudantes universitários consumiu álcool na última relação sexual, sendo este comportamento mais frequente nos rapazes (Pacheco, 2012; Reis et al., 2011). O álcool é usado como estimulador emocional, reduzindo as inibições e aumentando a confiança para novos contactos sociais, o que pode facilitar o sexo com parceiros ocasionais (Fielder & Carey,

2010). Contudo, o consumo de substâncias psicoativas está associado alterações de pensamento, com consequências diretas na capacidade de praticar sexo seguro e recusar ter relações sexuais não desejadas (Uecker, 2015; Wilton et al., 2014).

Dentro das influências situacionais foi considerada a disponibilidade e o tipo de acesso aos serviços de saúde. De uma forma geral os estudantes recorrem pouco aos serviços de saúde, conforme mencionado por dois dos estudantes *"Mesmo os estudantes da área saúde não se preocupam minimamente com isso. Aconselham e dão a informação mas não fazem". E2.4, " Noto que há muito desinteresse da parte delas. Muito desinteresse mesmo…". E2.6*

Observaram-se também diferenças no padrão de utilização dos serviços entre os géneros, sendo que a quase totalidade dos rapazes confirmam que não recorrem aos serviços de SSR, um dos estudantes afirma que *"Nunca fui a um serviço de saúde, porque se calhar nunca tive nenhuma dúvida, que não conseguisse esclarecer na internet ou com pessoas que tinham a certeza."* E2.5, e outro acrescenta *"[…] eu nunca fui ao médico de família sem a minha mãe, nunca tive disponibilidade para ir, nunca senti essa necessidade de ir, porque achava que supostamente já sabia tudo" E1.10,* até porque tem a perceção que estes estão essencialmente vocacionados para o género feminino, não atendendo às suas necessidades específicas:

> *"[…] o homem tem um papel tão importante como a mulher nesse aspeto do controle e prevenção da saúde sexual e também na contraceção […], mas nas consultas de planeamento familiar, em 80% dos casos só lá vão mulheres ou as jovens."* E2.4

A este propósito, afirma-se há mais de uma década que "em matérias ligadas à saúde reprodutiva, não tem sido atribuído o relevo necessário aos rapazes, enquanto grupo particular com necessidades específicas e com responsabilidades partilhadas com as raparigas" (Prazeres, 2003, p.11).

Estas assimetrias no acesso aos serviços de SSR têm vindo a ser documentadas (Vilar, 2012) e prendem-se com algumas das barreiras já identificadas no presente estudo, tais como *"O tempo de espera pela consulta ". No meu caso nunca frequentei o médico, porque se tiver de ir ao médico tenho de esperar para aí 3 meses".* E1.4. Outra das dificuldades está relacionada com a falta de divulgação dos serviços de saúde existentes, como é o caso dos da universidade, afirmando os estudantes que:

> *"Seria importante divulgar mais que temos cá esse serviço de saúde na universidade, porque eu não sabia. Acho que está pouco divulgado."* E1.3

Embora a maior parte dos estudantes não tenha recorrido aos serviços da universidade, as duas estudantes que utilizam estes serviços, realçam a grande disponibilidade dos profissionais de saúde e a rápida resposta às suas necessidades por parte destes serviços, referindo que *"Sim, os serviços são bons e mesmo a enfermeira é muito disponível e não precisamos de esperar pela consulta."* E2.8

Plano de intervenção para promoção de comportamentos sexuais seguros: um processo em construção

Realçando a importância de os indivíduos serem capazes de decidir sobre a sua saúde e serem envolvidos no delineamento das políticas de promoção da saúde, os estudantes foram incentivados a discutir e apresentar estratégias que considerassem relevantes para promover comportamentos sexuais seguros. Nesta perspetiva, os participantes revelaram alguma dificuldade em apontar estratégias, e as que foram apontando foram diversas, pouco consensuais, mas curiosamente estruturadas de um modo mais intimista e relacional, o que permitiu o debate entre vários intervenientes. Sugerem em particular a realização de palestras, reflexões em grupo, tertúlias, discussões focalizadas, debates e testemunhos de vida.

"Sim, … este formato de discussão em grupo, acho que poderia funcionar." E1.3

"Acho que poderia ajudar termos reuniões mais focadas em alguns temas, por exemplo sobre as IST. E se alguém pudesse estar presente e dar o seu testemunho." E2.4

"*Talvez o que possa afetar mais os jovens sejam os casos reais, onde se vê o sofrimento que eles passaram.*" E2.2

"Exatamente, histórias, narrativas. Acho que é isso que nos cativa mais e nos chama mais atenção. Acho que ficamos aterrorizados e que isso nos faz pensar, pode mesmo acontecer isto". Podemos ou não mudar o nosso comportamento, muitas vezes não vai acontecer, mas ficamos a pensar naquilo." E2

Não obstante mencionam *"Muita gente tem vergonha, toda agente se conhece, e tem receio de falar destes assuntos."* E1.4*; "Não com toda a gente […]. Acho que isso depende da personalidade, do à vontade para falar, porque as pessoas podem sentir-se acanhadas, porque têm uma opinião diferente e não a expressam. Acho que com pessoas mais tímidas esse método pode não ser eficaz."* E2.2

Surpreendentemente, a maioria dos estudantes dos dois grupos refere a possibilidade de serem utilizadas também estratégias de "confrontação pelo medo", com imagens reais e chocantes.

"Acho que a informação deveria ser acompanhada por esses tais casos chocantes, ou de coisas que chamem a atenção, riscos que as pessoas correm. Por exemplo, dizer isto pode acontecer-te, ou serem coisas que chamem a atenção." E1.2

"Se as pessoas fossem chocadas, como houve o choque da SIDA. Aí acho que as pessoas procurariam informação. Como os números referidos há bocado que 70% dos jovens são portadores do HPV, se houvesse um cartaz com esses valores não sei se ninguém ligava. Eu se calhar ligaria." E1.5

"Eu acho que é mesmo preciso assustar um bocadinho." E2.1

"Sim, se as imagens fossem chocantes, nós olhávamos para lá." E2.8

Embora a generalidade do grupo assuma que a confrontação com imagens chocantes pode ser uma estratégia a considerar, um dos estudantes faz alusão ao insucesso das campanhas de prevenção do uso do tabaco mencionando: *"Mas isso só não chega e até já existe por exemplo com o tabaco"* e sugere outras estratégias, tais como *"Acho que é mais pelo vídeo, pelo teatro, pelo cinema. Aquilo que vemos fica*". E2.4

Alguns estudantes justificam o discurso juvenil de desvalorização dos riscos sexuais, pela forma como a informação é transmitida nas campanhas de prevenção, com uma abordagem habitualmente centrada no modelo biomédico e racionalista. Tentando avançar uma explicação para a ineficácia das campanhas de prevenção de riscos sexuais em jovens, alguns estudos sugerem que este facto resulta de alguma saturação das campanhas de informação sobre VIH/SIDA, colocando em questão a eficácia persuasiva destas campanhas e a precisão da informação e das mensagens transmitidas, tal como verificado no discurso dos estudantes (Cunha-Oliveira, 2008).

"O "bicho papão" é a SIDA, mas existem outros vírus que se transmitem sexualmente que as pessoas não têm conhecimento, e eu próprio também não, a maior parte delas não revela logo sintomas […] e pode continuar a transmiti-los." E1.4

"O VIH/SIDA é o mais grave, mas se calhar não é a doença que existe mais, na nossa faixa etária." E1.1

Os estudantes consideram ainda que *"A informação é sempre muito teórica, ouvimos dizer: Já ouvi várias palestras sobre isso".* E2.8, e pouco esclarecedora: *"É preciso abordar as questões como elas são, não ter medo de assustar, não andar com rodeios."* E2.1, e que os educadores *"[…] ainda estão muito presos aos métodos expositivos, tipo os diapositivos…."* E2.2

Em síntese, ao mobilizarmos todas as afirmações dos estudantes sobre a possibilidade de melhoria de um plano de ação, compreende-se a crítica global às dinâmicas de grande grupo promovidas e um claro apelo a uma atitude de proximidade e de cuidado, desenvolvida pelos serviços de saúde.

Para além da perspetiva educacional, os jovens fizeram também referência à utilidade de programa de vigilância de saúde para jovens adultos, assim como a notificação obrigatória de IST, incluindo o VIH/SIDA.

"Como há consultas quando somos mais novos, também deveria haver quando somos mais velhos, quer iniciássemos relações sexuais quer não se iniciasse." E.1.3

"Eu também acho que sim, e por exemplo fazer um teste do VIH/SIDA deveria ser obrigatório. Em termos de saúde pública, deveria ser. Eu sei que mexe com questões éticas, com questões de liberdade, mas é uma questão que diz respeito a todos." E2.4

Considerações finais

Os dados obtidos nos grupos focais permitem em geral uma melhor compreensão da forma como os estudantes do ensino superior percecionam e vivenciam as questões relacionadas com a sexualidade e saúde reprodutiva, num olhar particular sobre os fatores que podem influenciar os comportamentos sexuais seguros

Ao nível dos comportamentos individuais e experiências, salientamos dos resultados obtidos neste grupo que todos os estudantes são sexualmente ativos, apontando como motivos para a iniciação sexual a maior interação entre jovens de diversas idades, a procura de afirmação no grupo de pares e, sobretudo, a pressão social para se tornarem sexualmente ativos. No que respeita às práticas contracetivas, todos os participantes referem usar contraceção, sendo os métodos mais utilizados o preservativo e dupla contraceção pílula-preservativo. A aquisição destes métodos é realizada preferencialmente no centro de saúde e IPJ. Na escolha do método contracetivo prevalece uma visão tradicional de género, onde as raparigas assumem a grande responsabilidade pela escolha do método utilizado. O acesso e a utilização dos serviços de SSR pelos estudantes são reduzidos e o padrão de utilização é dependente do género, com as raparigas a utilizarem mais estes serviços e a revelarem uma atitude mais preventiva, eventualmente influenciada pelo risco de gravidez indesejada.

No que respeita aos sentimentos, embora os estudantes revelem algum medo e preocupação com gravidez indesejada e com a aquisição de doenças, persiste aparentemente uma postura de despreocupação, comodismo e desinteresse, não só sobre o que diz respeito à prevenção de comportamentos de risco sexual, mas também nas questões de vigilância de saúde.

Embora percebam a importância da utilização consistente do preservativo, algumas estudantes admitem uma baixa perceção da autoeficácia para o usar. Nos seus discursos transparece o facto de a intenção de usar o preservativo nem sempre ser concretizada no momento em que se estabelece um contacto sexual, em particular nas relações de longa

duração, onde, mais uma vez, a questão da confiança é o argumento central para justificar o abandono desta medida de proteção.

Na promoção de comportamentos sexuais seguros, quer os benefícios, quer as barreiras desempenham um papel relevante. Como benefícios percebidos foram identificadas as características da personalidade, em particular a “personalidade forte”, consciente e responsável, uma atitude positiva face ao preservativo, assim como a capacidade de comunicação e de negociação do uso do preservativo com o parceiro. Alguns estudantes reconhecem, ainda, como importante o conhecimento da biografia sexual do companheiro, sobressaindo a convicção de que é legítimo o questionamento sobre o seu passado sexual como medida protetora, embora reconhecendo que esta abordagem pode ser difícil e levanta questões relacionadas com a confiança e o direito à intimidade.

No que refere às barreiras percebidas, para adotarem comportamentos sexuais seguros os estudantes centram as suas narrativas nas barreiras para uma utilização inconsistente do preservativo e nas relacionadas com o acesso aos serviços de SSR. Configuram-se como barreiras à utilização consistente do preservativo a estabilidade da relação com a utilização da contraceção hormonal oral, a confiança no parceiro, a crença de que o preservativo interfere no prazer sexual, em particular por parte dos rapazes, argumentando estes que o preservativo diminui o prazer e reduz a espontaneidade da relação sexual, bem como o embaraço na aquisição do preservativo. Apesar de os estudantes estarem informados dos riscos sexuais, existe uma desvalorização do risco e uma falta de consciencialização do comportamento sexual como um risco em si mesmo, decorrente da perceção do sentimento de invulnerabilidade. Nos dois grupos, existe algum consenso de que nas relações onde prevalecem os afetos, o compromisso e de confiança, não existe necessidade de uso do preservativo.

No que se refere às barreiras relacionadas com o acesso aos serviços de SSR, para além das questões de género, dado que os rapazes referem que os serviços de SSR são essencialmente vocacionados para o género feminino, os jovens mencionam ainda a falta de anonimato, a falta de confidencialidade da informação e a demora na marcação de consultas.

Em relação às influências interpessoais, destacam a possibilidade de o grupo de amigos ter um efeito protetor, mas também poder potenciar o risco, nomeadamente em relação ao consumo de substâncias psicoativas como o álcool, e a sua associação com práticas sexuais de risco. Os profissionais de saúde assumem um papel de destaque na divulgação e partilha de informação sobre comportamentos sexuais seguros, papel que é reconhecido sobretudo pelas raparigas. Os amigos ou grupos de pares são referidos por ambos os

géneros, mas em particular pelos rapazes. Embora as famílias tenham dificuldades em abordar as questões relacionadas com a sexualidade, as raparigas reconhecem a importância da família, em particular das mães, no desenvolvimento de uma atitude mais positiva para adoção de comportamentos sexuais seguros. A maioria dos estudantes considerou como importante o conhecimento e a informação sobre SSR que recebeu de diversas influências interpessoais, mas em particular dos profissionais de saúde, não só pela sensação de proteção e segurança que lhes transmite, mas também pela maior capacidade para tomar decisões sobre a sua vida sexual e possibilitar escolhas mais informadas e responsáveis.

Relativamente às influências situacionais, nos seus discursos os estudantes apontam as festas académicas, as discotecas e bares como contextos onde se estabelecem contactos com parceiros ocasionais e prevalecem consumos excessivos de álcool e drogas, associados a práticas sexuais de risco. Os consumos excessivos de substâncias psicoativas estão associados sobretudo à desinibição, o ato de beber social e a pressão dos pares. Os estudantes recorrem pouco aos serviços de SSR, mas alguns percebem benefícios na organização dos serviços, na disponibilidade dos profissionais de saúde e na facilidade na marcação das consultas, em serviços de saúde da comunidade (centros de saúde e IPJ), assim como nos serviços de saúde disponíveis na universidade

Nas propostas para melhorar a adesão a comportamentos sexuais seguros, observou-se algumas dificuldades nos grupos em refletirem e apontarem estratégias de visão futura que possam ser qualificadas de objetivas, assim como a falta de consenso sobre as estratégias que seriam mais eficazes. Não obstante, os jovens referem várias estratégias de proximidade, menos usuais ou contempladas nas dinâmicas dos profissionais. Essencialmente, referem práticas que intensifiquem a discussão e a partilha de vivências entre os jovens e que abandonem os métodos mais expositivos e de grande grupo, permitindo que surjam outras dinâmicas criativas. Propõem, sobretudo, abordagens mais interativas com enfoque cultural, grupos de discussão, reflexão em grupo, debate, teatro, cinema e exposições e por último o recurso às estratégias de confrontação pelo medo, onde incluem as “mensagens/imagens chocantes”.

Os jovens participantes atribuem aos profissionais de saúde e em especial aos enfermeiros um papel essencial, na promoção da saúde, valorizando fundamentalmente um contacto de proximidade, uma atenção cuidada e específica, bem como todas as atividades realizadas em pequenos grupos de pertença. Dos seus discursos, percebe-se a importância que atribuem a estes profissionais, quando estes reconhecem e respeitam as idiossincrasias dos jovens. Quando este nível de discrição, de comunicação e de respeito acontece, encontra-

se nos jovens uma outra implicação, *quiçá* um compromisso gerador de mudança e adesão a comportamentos sexuais seguros que se impõem por questões de saúde.

Referências bibliográficas

Alvarez, M. J., & Oliveira, M. (2007). Programa de prevenção do VIH/SIDA para estudantes universitários: Um estudo. *Revista Portuguesa de Educação, 21*(1),183-211.

Antunes, M. T. (2007). *Atitudes e comportamentos sexuais de estudantes do ensino superior*. Coimbra: Formasau.

Artistico, D., Oliver, L. S., Dowd, S., Rothenberg., A., & Khalil, M. (2014).The predictive role of self-efficacy, outcome expectancies, past behavior and attitudes on condom use in a sample of female college students. *Journal of European Psychology Students*, *5*(3), 100-107. doi: http://dx.doi.org/10.5334/jeps.cl

Asare M. (2015). Using the theory of planned behavior to determine the condom use behavior among college students. *American Journal of Health Study, 30*(1), 43-50.

Bardin, L (2004). *Análise de conteúdo*. Lisboa: Edições 70; 197.

Barros, J., & Neves. J. (2013). Contraceção na adolescência. In J. Neves*, Contraceção* (pp. 121-134). Lisboa: Lidel.

Billups, F. D. (2012). Conducting focus groups with college students: Strategies to ensure success. *Research Methodology. Paper 2.*

Borges, A. L., Fujimori, E., Hoga, L. A., & Contin, M. V. (2010). Práticas contraceptivas entre jovens universitários: O uso da anticoncepção de emergência. *Cadernos Saúde Pública, 26*(4), 816-826, doi:org/10.1590/S0102-311X2010000400023

Claxton, S. E., & van Dulman, M. H. M. (2015). Casual sexual relationships and experiences in emerging adulthood. In *The Oxford handbook of emerging adulthood* (Cap. 16, pp. 245-261). New York: Oxford University Press.

Cunha-Oliveira (2008). *Preservativo, sida e saúde pública*. Coimbra: Imprensa da Universidade de Coimbra.

Cunha-Oliveira, A., Cunha-Oliveira, J., Pita, J., & Massana-Cardoso, S. (2009). A aquisição do preservativo e o seu (não) uso pelos estudantes universitários. *Revista Referência, 11* (Série 2), 7-22.

Dias, D. & Sá, M. J. (2013). Tornar-se jovem ou estudante: Um desafio desenvolvimental. *Revista Portuguesa de Pedagogia, 47*(2), 65-84.

Dias, S. F., & Rocha, C. F. (2009). Saúde sexual e reprodutiva de mulheres imigrantes africanas e brasileiras: Um estudo qualitativo. Lisboa: Alto-comissariado para a Imigração e Diálogo Intercultural.

Direção-Geral da Saúde (2008). *Saúde reprodutiva. Planeamento familiar. Programa nacional de saúde reprodutiva*. Lisboa: Autor.

Ferreira, P. M., & Cabral, M. V. (2010). *Sexualidades em Portugal: Comportamentos de risco*. Lisboa: Editorial Bizâncio.

Fielder, R. L., & Carey, M. P. (2010). Predictors and consequences of sexual “hookups” among college students: A short-term prospective study. *Archives of Sexual Behavior, 39*(5), 1105-1119.

French, S., & Holland, K. (2013). Condom negotiation strategies as a mediator of the relationship between self-efficacy and condom use. *Journal of Sex Research, 50*(1), 48-59. doi:10.1080/00224499.2011.626907

Garcia, J. R., Reiber, C., Massey, S. G., & Merriwether, A. M. (2012). Sexual hookup culture: A review. *Journal of Division 1, of the American Psychological Association, 16*(2), 161-176. doi:org/10.1037/a0027911

Ghandour, L. A., Mouhanna, L., Yasmine, R., & El Kak, F. (2014). Factors associated with alcohol and/or drug use at sexual debut among sexually active university students: Cross-sectional findings from Lebanon. *BMC Public Health, 14*, 671-681.

Giddens, A. (2002). *A transformação da intimidade: Sexualidade, amor e erotismo nas sociedades modernas*. São Paulo: Editora da Unesp.

Goldstein, R. L., Upadhyay, U. D., & Raine, T. R. (2013). With pills, patches, rings, and shots: Who still uses condoms? A longitudinal cohort study. *The Journal of Adolescent Health, 52*(1), 77-82. doi:10.1016/j.jadohealth.2012.08.001

Gomes A., & Nunes C. (2011). Caracterização do uso do preservativo em jovens adultos portugueses. *Análise Psicológica*, *4*(29), 489-503.

Guo, G., Li, Y., Owen, C., Wang, H., & Duncan, G. J. (2015). A natural experiment of peer influences on youth alcohol use. *Social Science Research, 52*, 193-207.

Horan, S.M. (2016). Further understanding sexual communication: Honesty, deception, safety, and risk. *Journal of Social and Personal Relationships*. 33(4), 449–468. doi: 10.1177/0265407515578821

Hyde, A., Howlett, E., Brady, D., & Drennan, J. (2005). The focus group method: Insights from focus group interviews on sexual health with adolescents. *Social Science and Medicine, 61*, 2588-2599.

International Consortium for Emergency Contraception. (2015). Repeated use of emergency contraceptive pills: The facts. New York. Retirado de http://www.cecinfo.org/custom-content/uploads/2015/10/ICEC_Repeat-Use_Oct-2015.pdf

Jayasekara, R. S. (2012). Focus groups in nursing research: Methodological perspectives. *Nursing Outlook, 60*(6), 411-416. doi:org/10.1016/j.outlook.2012.02.001

Kann, L., McManus,T., Harris, W., Shanklin, S.L., Flint, K.,… Zaza, S. (2016). Youth risk behavior surveillance - United States, 2015. *Centers for Disease Control and Prevention, 65*(6), 1-171.

Krueger, R. A., & Casey, M. A. (2009). *Focus groups: A practical guide for applied research* (4th ed.). San Francisco: Sage.

Kuperberg, A., & Padgett, J. E. (2015). Dating and hooking up in college: Meeting contexts, sex, and variation by gender, partner's gender, and class standing. *The Journal of Sex Research, 52*(5), 517-531. doi:10.1080/00224499.2014.901284

Lara Flores, N., Saldaña Balmori , Y., Férnandez Vera, N., & Delgadillo Gutiérrez, H. J. (2015). Salud, calidad de vida y entorno universitario en estudiantes mexicanos de

una universidad pública. *Hacia la Promoción de la Salud*, *20*(2), 102-117. doi:10.17151/hpsal.2015.20.2.8

Lewis, M. A., Granato, H., Blayney, J. A., Lostutter, T. W., & Kilmer, J. R. (2012). Predictors of hooking up sexual behaviors and emotional reactions among U.S. *College Students .Archives of Sexual Behavior, 41*(5), 1219-1229. Doi:org/10.1007/s10508-011-9817-2

Lobdell, D., Gilboa, S., Mendola, P., & Hesse, B. (2005). Use of focus groups for the environmental health researcher. *Journal of Environmental Health*, *67*(9), 36-42.

Logan, D. E., Koo, K. H., Kilmer, J. R., Blayney, J. A., & Lewis, M. A. (2015). Use of drinking protective behavioral strategies and sexual perceptions and behaviors in U.S. college students. *Journal of Sex Research, 52*(5), 558-569. doi:10.1080/00224499.2014.964167.

Lopez, L. M., Otterness, C., Chen, M., Steiner, M., & Gallo, M. F. (2013). Behavioral interventions for improving condom use for dual protection (Review). *Cochrane Database Systematic Reviews. 10*. doi:10.1002/14651858.CD10662.pub2

Maia, M. (2010). Práticas de risco no contexto das relações homossexuais. In P. M. Ferreira & M. V. Cabral, *Sexualidades em Portugal: Comportamentos de risco* (Cap. 9, pp. 324-387). Lisboa: Editorial Bizâncio,

Manlove, J., Ikramullah, E., & Terry-Humen, E. (2008). Condom use and consistency among male adolescents in the United States. *Journal Adolescent Health, 43*, 325-333. doi: 10.1016/ j.jadohealth.2008.03.008.

Martins, A. T., Nunes, C., Muñoz-Silva, A., & Sánchez-García, M. (2008). Fontes de informação, conhecimentos e uso do preservativo em estudantes universitários do Algarve e de Huelva. *Psico, 39*(1), 7-13.

Matos, M. G., Reis, M., Ramiro, L., & Equipa Aventura Social. (2011). Saúde sexual e reprodutiva dos estudantes do ensino superior. Relatório do estudo: Dados nacionais 2010. Lisboa: Aventura Social.

Morgan, D. L., & Krueger, R. A. (Eds.). (1998). *The focus group kit*. Thousand Oaks, CA: Sage.

O'Sullivan, L. F., Udell, W., Montrose, V. A., Antoniello, P., & Hoffman, S. (2010). A cognitive analysis of college students' explanations for engaging in unprotected sexual intercourse. *Archives of Sexual Behavior, 39*(5), 1121-1131. doi:org/10.1007/s10508-009-9493-7

Oliveira, A.C. (2014). *VIH/SIDA e comportamentos de risco: Monitorizar a evolução*. Tese de doutoramento não publicada, Faculdade de Medicina da Universidade de Coimbra.

Oliveira, D. L. (2011). The use of focus groups to investigate sensitive topics: An example taken from research on adolescent girls' perceptions about sexual risks. *Ciência & Saúde Coletiva, 16*(7), 3093-3102. doi:org/10.1590/S1413-81232011000800009

Oliveira, J. G. (2015). A confiança nas relações homoeróticas femininas. *LES Online, 7*(1), 17-25.

Pacheco, N. S. (2012). *A sexualidade dos jovens estudantes universitários portugueses.* Tese de doutoramento não publicada, Universidade da Beira Interior, Covilhã.

Pender, N., Murdaugh, C., & Parsons, M. A. (2011). Individual models to promote health behavior. In M. Connor, D. MacKnight, K. Mortimer & S. Wrocklage (Eds.), Health Promotion in Nursing Practice (pp. 35-66). New York: Pearson.

Peterson-Sweeney, K. (2005). The use of focus groups in pediatric and adolescent research. *Journal of Pediatric Health Care*, *19*(2), 104-110.

Pollock, T. (2016). *The effects of alcohol consumption and perception of normative behaviors on risky sexual behavior among college students.* Thesis submitted in partial fulfillment of the requirements for Honors Studies in Social Work. The University of Arkansas.

Prazeres, V. (2003). *Saúde juvenil no masculino: Género e saúde sexual e reprodutiva.* Lisboa: Direção-Geral da Saúde.

Reis, M., Ramiro, L., Matos, M.G., & Diniz, J.A. (2013). Determinants influencing male condom use among university students in Portugal. *International Journal of Sexual Health, 25*(2), 115-27.

Rocha, E. (2008). Universidades promotoras de saúde. *Revista Portuguesa de Cardiologia*, *27*(1), 29-35. Retirado de http://www.spc.pt/DL/RPC/artigos/886.pdf

Sakraida, T. (2004). Modelo de promoção da saúde. In A. Marriner Tomey & M. Alligood (Eds.), *Teóricas de enfermagem e a sua obra: Modelos e teorias de enfermagem* (pp. 699-715). Loures: Lusociência.

Silva, A., & Brito, I. (2014). Instituições de ensino superior promotoras de saúde. In R. Pedroso & I. Brito (Eds.), *Saúde dos estudantes do ensino superior de enfermagem: Estudo de contexto na Escola Superior de Enfermagem de Coimbra*. Coimbra: Unidade de Investigação em Ciências da Saúde, Escola Superior de Enfermagem de Coimbra.

Trieu, S. L., Bratton, S., & Marshak, H. (2011). Sexual and reproductive health behaviors of California Community College Students. *Journal of American College Health*, *59*(8), 744-750. doi:10.1080/07448481.2010.540764

Uecker, J. E. (2015). Social context and sexual intercourse among first-year students at selective colleges and universities in the United States. *Social Science Research, 52*, 59-71.

Vilar, D. (2012). *Projeto Sexual Awareness for Europe (SAFE II)*. Lisboa: Associação para o Planeamento da Família.

Vilar, D., & Ferreira, P. M. (2009). Educação sexual dos jovens portugueses: Conhecimentos e fontes. *Educação Sexual em Rede*, *5,* 2-53.

Vítor, J. F., Lopes, M. V., & Ximenes, L. B. (2005). Análise do diagrama do modelo de promoção da saúde de Nola J. Pender. *Acta Paulista de Enfermagem, 18*(3), 235-240.

Voisin, D., King, K., Schneider, J., Diclemente, R. & Tan, K. (2012). Sexual sensation seeking, drug use and risky sex among detained youth. *AIDS & Clinical Research*, S1, 1-5.

Wechsler, H., Xuan, Z., Lee, H., Weitzman, E. R., & Wechsler, H. (2009). Persistence of heavy drinking and ensuing consequences at heavy drinking colleges. *Journal of Studies Alcohol Drugs*, *70*(5),726-734.

Wilton, L., Palmer, R. T., & Maramba, L. C. (2014). Understanding HIV and STIs prevention for college students. London: Routledge Research in Higher Education.

CAPÍTULO 5

SÍNTESE INTEGRATIVA E PRINCIPAIS CONCLUSÕES

O presente trabalho teve como objetivo geral contribuir para a compreensão dos comportamentos de SSR de estudantes do ensino superior, no sentido de dispor de informação atualizada e específica para este grupo populacional, visando delinear intervenções para melhorar as falhas detetadas. Para cumprir esse objetivo, estudaram-se os comportamentos de contraceção desses estudantes, as suas práticas sexuais de risco e a sua vigilância da saúde. Foram identificados os fatores individuais e socioculturais da população em estudo e utilizados vários constructos para avaliar aspetos cognitivos, psicossociais e de personalidade, no sentido de identificar quais os determinantes dos comportamentos sexuais de risco e de proteção mais relevantes, visando estabelecer estratégias de promoção da saúde. Dado o estereótipo tradicional do papel dos géneros na sexualidade, atendeu-se a essa questão na análise de resultados. Acessoriamente, procedeu-se à construção, adaptação cultural e validação de instrumentos psicométricos com relevância para o estudo da área da sexualidade e saúde reprodutiva nesta população.

A amostra do presente estudo foi constituída por 1946 estudantes, com idade média de 21 anos, de uma universidade do norte de Portugal no ano letivo de 2012/2013. A maioria era de nacionalidade portuguesa, solteira, proveniente da cidade, de famílias de baixo rendimento e baixo nível de escolaridade. A idade média de início de atividade sexual situava-se nos 17 anos. A maioria indicou ter tido relações sexuais nos últimos 12 meses.

Conhecimento SSR. Os estudantes do ensino superior têm um conhecimento mediano sobre SSR (22,76±5,79, pontuação entre 0 e 44). Dos quatro domínios que compuseram o inventário de conhecimento, observou-se que os estudantes têm melhor conhecimento sobre contraceção (8,42±2,44, em 16) e pior sobre comportamentos sexuais de risco (6,05±2,73 em 16). As raparigas apresentaram um conhecimento superior, em particular sobre contraceção. Esta diferença vai ao encontro do papel dos géneros na sexualidade, que atribui essencialmente à rapariga a responsabilidade de não engravidar, pelo que há um muito maior investimento das próprias, da família e do sistema de saúde em informá-las adequadamente. Os estudantes mais novos, provavelmente devido às mudanças nos currículos do ensino básico e secundário que aconteceram durante a sua escolaridade – com a obrigatoriedade de ensino de educação sexual - apresentam conhecimentos superiores. Como seria de esperar, os estudantes da área científica de ciências da vida e da saúde – agrárias, da vida e do ambiente e enfermagem – têm melhores conhecimentos sobre SSR do que os estudantes de tecnologias e ciências humanas e sociais. Este facto dever-se-á ao percurso em ciências desde o ensino secundário e à presença de disciplinas de anatomia, fisiologia, biologia de mamíferos, reprodução, de entre outras, nos currículos dos cursos dessas áreas (cursos de Enfermagem, Medicina Veterinária, Biologia, Desporto, Engenharia Zootécnica).

Atitude SSR. Os estudantes revelaram ter uma atitude muito favorável face à SSR, claramente acima da média (118,28±13,92, intervalo da escala 20-140) em todas as dimensões da escala. Como há uma forte associação entre o conhecimento e a atitude, as tendências observadas para o género, a idade e a área científica de estudos foram basicamente as mesmas. Porém, a associação entre atitude e conhecimento foi mais forte nos rapazes do que nas raparigas, indicando que para eles o conhecimento é mais importante para a formação de atitude do que para elas.

As *práticas contracetivas* dos estudantes do ensino superior, ainda que adequadas, revelam falhas e várias oportunidades de melhoria. Alguns estudantes têm relações sexuais e indicam não usar contraceção (raparigas 2,9%; rapazes 5,6%). Este cenário é seriamente agravado quando se considera a utilização consistente do preservativo – assumida no presente estudo como sendo a utilização em todas as relações sexuais. Cerca de metade (49,8%) daqueles que referem usar preservativo, isoladamente ou em combinação com a pílula, indicam também que não o usam em todas as relações sexuais, pelo que foram considerados não utilizadores. Assim, as proporções de estudantes que não usam contraceção aumentaram para 7,2% nas raparigas e para uns preocupantes 32,6% nos rapazes. As escolhas dos métodos contracetivos estão muito dependentes de quem os utiliza de forma mais direta. As raparigas utilizam mais o método hormonal (52,9%) e os rapazes o preservativo (32,1%). A quase totalidade das estudantes que refere usar o método hormonal utiliza a pílula, remetendo os métodos menos dependentes da utilizadora – anel vaginal, adesivo, implante – para uma proporção residual. A dupla contraceção, que tem vindo a ser preconizada pela sua elevada eficácia na prevenção simultânea da gravidez e IST, era usada somente por 17,6% dos estudantes. A utilização de CE é apontada por cerca de um quarto dos estudantes, sendo justificada maioritariamente pela falha no método contracetivo habitual. Em linha com os resultados observados na avaliação do conhecimento sobre SSR, as raparigas procuram fontes de informação mais formais, essencialmente profissionais de saúde, enquanto os rapazes recorrem a fontes mais informais. Os estudantes mais velhos têm piores práticas contracetivas, o que poderá decorrer do seu menor conhecimento ou de uma maior valorização erótico-hedonista, particularmente por parte dos rapazes, devido à alegada redução de prazer que associam ao preservativo. Um dos achados mais preocupantes do presente estudo foi o abandono da utilização do preservativo por estudantes que namoram, e sua substituição pela contração hormonal. Esta opção resulta da alegada confiança no passado sexual e na monogamia do parceiro. Porém, resultados publicados por vários investigadores e os resultados do presente estudo são consensuais quanto à ocorrência de práticas sexuais fora da relação de namoro, que no presente trabalho foi identificada por 26,1% dos participantes, e na omissão

do passado sexual. A maioria das variáveis cognitivas e psicossociais contribuíram para as escolhas contracetivas. Observou-se que a não utilização de contraceção está muito associada a um menor conhecimento e uma atitude menos favorável. A autoeficácia para a utilização do preservativo mostrou-se mais importante em raparigas do que em rapazes, particularmente porque as raparigas que não usam contraceção têm uma autoeficácia menor.

Os estudantes do ensino superior estão expostos a riscos sexuais e reprodutivos. Esses decorrem da não utilização consistente do preservativo (60,5%), da prática de sexo com parceiros ocasionais (32,0%), dos quais se desconhece o passado sexual e o nível de risco que representam. Por sua vez, a combinação de sexo com substâncias psicoativas que reduzem a capacidade cognitiva, nomeadamente álcool (33,0%) ou drogas (9,7%), podem também ter consequências negativas pela redução da capacidade para negociar a utilização de preservativo, para o utilizar de forma correta e para avaliar o risco que pode representar envolver-se com determinados parceiros dos quis desconhecem o estado serológico e/ou em determinadas práticas sexuais. As tendências observadas neste trabalho, genericamente, vão de encontro às detetadas quando se estudou a questão da contraceção. Os rapazes e os estudantes mais velhos são os que estão mais expostos ao risco. Os estudantes provenientes de famílias com maiores rendimentos estão mais expostos ao risco de combinar sexo com álcool e/ou drogas e de terem parceiros sexuais ocasionais, provavelmente devido ao poder de compra e à inerente vida social noturna mais intensa. A crença religiosa e a utilização do preservativo na primeira relação sexual são dois fatores que se mostraram protetores na adoção de comportamentos de risco. Tal como suprarreferido para as escolhas contracetivas, quando as relações sexuais acontecem no contexto do namoro verifica-se o abandono do preservativo. Porém, em relação às outras práticas consideradas de risco, o namoro tem um efeito protetor. As variáveis cognitivas e psicossociais estudadas apresentaram uma tendência de, a uma maior exposição ao risco, corresponder uma atitude SSR mais desfavorável, uma menor importância dada aos pares, uma menor autoeficácia para o uso do preservativo e uma maior procura de sensações sexuais. Ao contrário do observado para as escolhas contracetivas, o conhecimento sobre IST não influenciou a exposição ao risco, o que se poderá dever ao facto de este segmento do conhecimento dos estudantes ser aquele onde se detetaram maiores fragilidades. Apesar das tendências observadas, quando estas variáveis cognitivas e psicossociais foram estudadas em conjunto através de regressão logística, as normas subjetivas foram as que mostraram um efeito protetor mais relevante e a procura de sensações sexuais um efeito potenciador do risco. Os resultados deste trabalho revelam que os estudantes têm alguma exposição ao risco. Transparece dos resultados alguma perceção de invulnerabilidade, que

é característica desta faixa etária, que não valoriza eventuais repercussões negativas destes comportamentos de risco, confirmada pela elevada percentagem de estudantes que desconhece o seu estado serológico para a infeção pelo VIH/SIDA (72,6%) e que não realizaram a vacinação para o HPV (33,1% das raparigas). O carácter assintomático das IST contribui para essa perceção, uma vez que muitas das complicações de saúde decorrentes destas infeções só se manifestam a longo prazo. Pelos resultados da avaliação do conhecimento, das práticas contracetivas e da exposição ao risco de infeção por IST, inferiu-se da existência de falhas na promoção de SSR no âmbito deste grupo etário. Nesse sentido, estudaram-se as barreiras no acesso aos cuidados de saúde. Existe uma subutilização dos serviços de SSR e pouca adesão a comportamentos de vigilância de saúde, particularmente por parte dos rapazes. Muitos desconhecem mesmo a existência de serviços de saúde enquadrados nos serviços de ação social da universidade. Na perspetiva dos estudantes, as maiores dificuldades no acesso aos serviços de SSR estão relacionadas com a localização e com os horários de funcionamento.

A SSR dos jovens universitários é influenciada por múltiplos fatores, individuais, sociais, cognitivos, psicossociais e relacionados com a sua própria personalidade, pelo que se reveste de alguma complexidade. Porém, há uma prática simples e económica que pode reduzir substancialmente o risco de gravidez indesejada e de IST – a utilização consistente do preservativo – que, infere-se dos estudos previamente abordados, não é utilizada por um número significativo dos estudantes que têm relações sexuais. Essa utilização consistente do preservativo é um comportamento promotor de saúde que se pretende incentivar nos estudantes. Assim, utilizando o referencial teórico do HPM de Pender, procurou-se perceber quais os determinantes que tinham um maior impacto no compromisso para utilizar o preservativo e na sua utilização propriamente dita. Observou-se que a maioria das variáveis estudadas teve uma associação significativa com o compromisso para adotar o comportamento promotor de saúde. Os benefícios percebidos, os sentimentos positivos e as influências interpessoais foram as variáveis que tiveram uma influência positiva mais clara sobre o compromisso para utilizar o preservativo, com tendência semelhante em ambos os géneros. A perceção de barreiras e sentimentos negativos influenciaram negativamente a adoção do comportamento. A autoeficácia para o uso do preservativo, ao contrário do que seria de esperar, mostrou-se um fraco preditor, quer para o compromisso, quer para o comportamento propriamente dito. A estrutura multivariada da associação dos constructos psicossociais e o comportamento promotor de saúde demonstra que o modelo teórico, que refere que o acontecimento que marca a adoção do comportamento é sobretudo o compromisso com a ação. Os resultados demostram essa forte associação, pois o compromisso com a utilização do preservativo tem uma elevada associação com o uso do

preservativo. Embora o HPM assuma que há constructos que têm influência direta no comportamento promotor de saúde, no presente trabalho observou-se que esses constructos atuam essencialmente através da variável mediadora compromisso. Estes resultados identificam quais são os principais domínios em que se deve trabalhar com os estudantes do ensino superior para os ajudar a comprometer-se com, e adotar o comportamento promotor de saúde - a utilização consistente do preservativo.

A investigação quantitativa, baseada em questionários, é muito útil para conhecer as grandes tendências da população em estudo. Porém, pela sua natureza, perdem-se detalhes que podem ser importantes para compreender alguns fenómenos e, consequentemente, melhorar o planeamento de estratégias para resolver os constrangimentos identificados. Através da utilização de grupos focais com estudantes do ensino superior, foi possível compreender algumas questões para as quais não tínhamos explicação clara, assim como identificar as motivações subjacentes a algumas opções assumidas pelos estudantes. Da análise dos discursos gerados durante os grupos focais, confirmaram-se as grandes tendências observadas no estudo quantitativo. Uma maior preocupação e responsabilidade por parte das raparigas, uma grande preocupação com a gravidez e reduzida perceção de vulnerabilidade face às IST. Os estudantes têm um discurso muito favorável à utilização do preservativo, mas a utilização inconsistente que foi discutida ao longo de todo o estudo quantitativo, aparece também no discurso dos estudantes dos grupos focais, pois estes indicam usá-lo, mas admitem muitas exceções. O motivo para essas exceções confirma algumas das hipóteses que foram colocadas para justificar a sua não utilização – a forte confiança no parceiro e a alegada redução de prazer associada à utilização do preservativo. Também no sentido confirmatório, o “calor do momento”, não poucas vezes alimentado pelo álcool ou por drogas, é justificação para não usar preservativo. As competências de comunicação e negociação foram frequentemente abordadas no discurso, no que se refere à negociação para a utilização do preservativo. As barreiras no acesso aos serviços de saúde confirmam-se, mencionando os estudantes que os horários são desadequados e há falta de confiança na confidencialidade e anonimato. Os rapazes desconhecem, não usam, sentem que não precisam e não se revêm nos serviços atualmente disponíveis, que na sua opinião são muito vocacionados para a saúde da mulher. No que respeita aos serviços de saúde da universidade, há um desconhecimento generalizado da sua existência, localização e horários que praticam. As raparigas são as principais utilizadoras destes serviços, mas principalmente para obter contraceção, dado que raramente os usam para fazer vigilância de saúde.

Quando os estudantes foram desafiados a propor estratégias para melhorar a situação, o discurso foi algo incoerente e discordante entre os participantes. Numa perspetiva foram

unânimes: a participação naquele grupo focal melhorou a sua forma de olhar para o problema, pelo que lhes agradou a ideia de iniciativas dessa natureza, em grupos pequenos e com ambiente intimista para se discutirem estas questões da sexualidade e saúde reprodutiva. Foram apontadas várias pistas mais ou menos previsíveis, como a utilização de estratégias de sensibilização através reflexões em grupo, discussões focalizadas, debates e testemunhos de vida ou mesmo a utilização do teatro e artes performativas. Um dos aspetos que consideramos mais inesperado, e que reuniu algum consenso entre os participantes, foi a utilização de estratégias de “confrontação pelo medo”, com fotografias e dados sobre IST. Alguns estudantes justificam o discurso juvenil de desvalorização dos riscos sexuais, pela forma como a informação é transmitida nas campanhas de prevenção, nomeadamente nas campanhas de informação sobre o VIH/SIDA, com uma abordagem habitualmente centrada no modelo biomédico e racionalista, onde a promoção do preservativo é sempre realizada numa perspetiva preventiva e sanitarista, e não na perspetiva erótico-hedonista.

Com base nos resultados do presente estudo, podemos inferir que os estudantes universitários têm práticas contracetivas suscitáveis de serem melhoradas e são um grupo com alguma exposição ao risco para IST. A utilização consistente e correta do preservativo é o comportamento promotor de saúde que importa consolidar, associado ao evitamento de situações de maior exposição ao risco e a uma vigilância de SSR mais regular. Os resultados do presente estudo sugerem que na promoção da SSR dos estudantes podem ser considerados dois níveis de intervenção: (re)organização e adequação dos serviços e cuidados de saúde às necessidades específicas dos estudantes e o delineamento de programas educacionais direcionados no sentido de colmatar as lacunas identificadas pelos estudantes. Ao nivel educacional algumas das estratégias a considerar podem incluir desenvolvimento de competências pessoais, cognitivas e sociais, com participação dos jovens no desenvolvimento das intervenções educativas. Ao nível organizacional deve ser priorizada a adequação dos serviços de saúde (facilitando o acesso administrativo, o acesso à informação e diminuindo o distanciamento social) e a divulgação dos serviços de saúde existentes, nomeadamente dos serviços e SSR da universidade.

Limitações do estudo

Todo o processo de investigação científica tem associado um conjunto de limitações, nomeadamente de carácter metodológico, e a presente investigação não é exceção, nesse sentido as principais limitações metodológicas foram mencionadas nos diversos estudos apresentados.

CAPÍTULO 6

LINHAS ORIENTADORAS DE PROMOÇÃO DA SAÚDE SEXUAL E REPRODUTIVA PARA ESTUDANTES DO ENSINO SUPERIOR

Na promoção da saúde podem ser usadas diferentes estratégias e abordagens para desenvolver as capacidades individuais, alterar os contextos sociais e os comportamentos de saúde das pessoas. Estratégias que na sua interrelação visam estabelecer políticas públicas saudáveis, criar ambientes favoráveis à saúde, envolver a comunidade, reorganizar os serviços de saúde e desenvolver competências pessoais (OE, 2000). Nesta perpectiva, reconhecendo a importância e a diversidade das questões relacionadas com a SSR, é importante intervir na promoção de comportamentos sexuais saudáveis junto dos estudantes do ensino superior. Os enfermeiros na complementaridade com outros profissionais de saúde podem assumir o papel de agentes facilitadores e dinamizadores na implementação de programas de promoção da SSR nas instituições de ensino superior.

Em função dos resultados do presente estudo apresentam-se algumas linhas orientadoras para o desenvolvimento de um programa de promoção da SSR para estudantes do ensino superior, sendo que foi possível identificar pelo menos dois eixos de intervenção - educacional e organizacional - que serviram de linhas orientadoras para a proposta de intervenção. De salientar que, embora o modelo de intervenção tenha sido conceptualizado para a universidade onde decorreu o estudo, esta proposta poderá ser inspiradora de outras práticas, noutras instituições, que se podem assumir como pequenos contributos para a concretização a longo prazo da rede de instituições de ensino superior mais saudáveis.

A adoção de comportamentos salutogénicos é um desafio que se tem colocado sistematicamente aos profissionais de saúde, pois muitas vezes são identificados os determinantes desses comportamentos, mas a fraca compreensão das suas relações e interações compromete o resultado final pretendido, que é a adoção do comportamento promotor de saúde. Neste contexto, o HPM de Pender, ajuda a compreender essa complexidade, pelo que foi usado como referencial organizativo de medidas que consideramos pertinentes para promover a mudança de comportamentos sexuais de risco.

Na Figura 1 é apresentada esquematicamente a compreensão das tendências identificadas nos estudos, quantitativo e qualitativo, realizados com esta população e agrupa os constructos identificados no HPM de Pender. Ao nível da primeira componente – características - apresentam-se os comportamentos comprometedores de saúde identificados e as características individuais avaliadas. Na segunda componente que representa o núcleo central do modelo – conhecimento e sentimentos sobre o comportamento específico- apresentam-se os resultados mais importantes do estudo (caixa a branco) de acordo com as variáveis propostas no HPM e apontam-se as medidas educacionais ou organizacionais mais relevantes para melhorar o comportamento promotor de saúde (caixa a preto). A terceira componente - resultado do comportamento - não foi avaliada dado que não foi realizada a implementação do programa. Na faixa lateral foi

incluída uma nota relativa aos instrumentos utilizados. A apresentação da proposta do programa de intervenção seguirá esta orientação esquemática, realizando-se numa primeira fase a apresentação dos resultados mais relevantes do estudo, seguindo-se uma síntese das medidas propostas para melhorar o comportamento promotor de saúde dos estudantes, que serão destacadas em itálico no texto.

No presente estudo, foram identificados três comportamentos comprometedores de saúde: (1) aquele que se configura como o mais importante é a utilização não consistente do preservativo; (2) a associação de relações sexuais com álcool ou drogas, e as relações sexuais com parceiros ocasionais e (3) a insuficiente vigilância de saúde SSR entre os estudantes, uma vez que os rapazes não a fazem de todo e as raparigas, embora usem os serviços de saúde, fazem-no maioritariamente para obter contracetivos. Ainda que, ao longo do estudo, estes comportamentos tenham sido analisados *per si*, estão profundamente interrelacionados, sendo previsível que as estratégias conducentes à mudança de um deles, melhore também os outros.

Dos fatores individuais que estão associados ao comportamento comprometedor de saúde, no presente estudo concluiu-se que os que têm maior influência são: ser do género masculino, ser mais velho, namorar, ser frequentador de atividades sociais noturnas, ser de famílias com maior poder de compra, e ter uma personalidade que valorize o hedonismo e o risco. Estes aspetos não são passíveis de ser modificados, mas a sua identificação é determinante para o sucesso de qualquer intervenção, pois é importante incorporar essas características individuais nas estratégias utilizadas, tanto ao nível dos cuidados de saúde, como de educação. Os estudantes que não valorizam a religião foram os que estavam mais expostos ao risco. Porém, dado tratar-se de um tema sensível, consideramos que esta variável não deve ser considerada em qualquer estratégia promotora da mudança de comportamento.

O núcleo central do HPM identifica o conhecimento e os sentimentos sobre o comportamento específico, e é considerada a parte mais importante do modelo, pois sistematiza as variáveis que podem ser modificadas no sentido de promover a adoção de comportamentos sexuais seguros. Essas incluem a perceção de benefícios e barreiras para a ação, a autoeficácia, os sentimentos positivos e negativos, as influências interpessoais e as situacionais, o compromisso com o plano de ação e os fatores competidores.

No presente estudo foi possível determinar que os estudantes têm uma fraca perceção dos benefícios que podem obter do comportamento promotor da saúde.

.

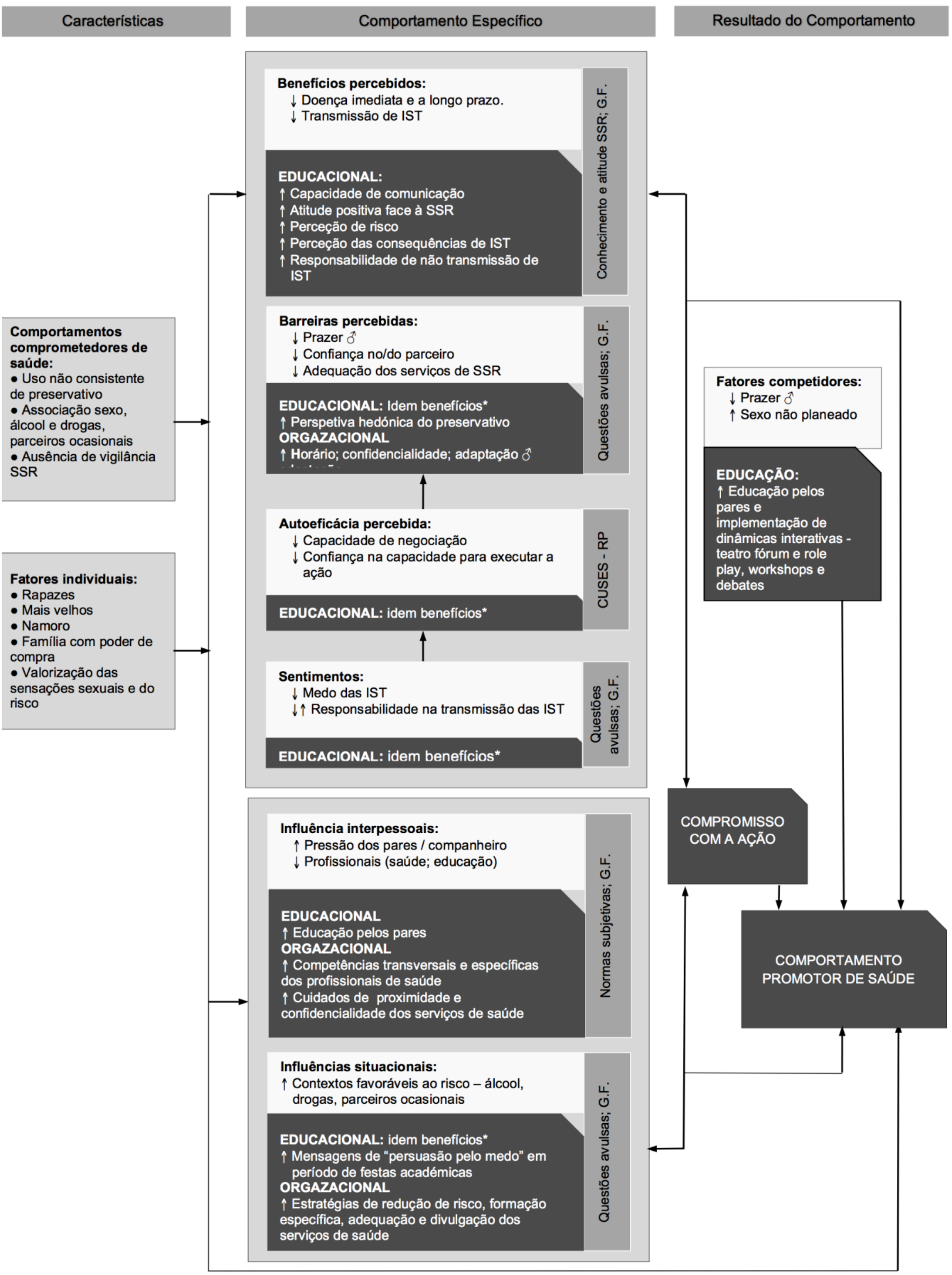

Figura 1. Síntese dos resultados do presente estudo relativos aos constructos do HPM de Pender, instrumentos utilizados para os avaliar e medidas propostas para melhorar o comportamento promotor de saúde dos estudantes; **GF**. Grupos Focais; * A nível educacional as estratégias são similares.

Os estudantes não se consideram expostos ao risco e entendem que esse não é um problema que os possa afetar. Verificou-se, também, que têm uma fraca perceção do risco que podem representar para outrem enquanto fonte de transmissão de IST, particularmente porque desconhecem se são eventuais portadores assintomáticos da infeção, uma vez que a grande maioria desconhece o seu estado serológico.

Melhorar a perceção de benefício pode passar por estratégias de educação com o intuito de reforçar a perceção de risco e das consequências das IST, garantindo que os estudantes se possam rever em si mesmos como potencialmente responsáveis por transmissão de IST a outros parceiros sexuais. As estratégias educativas devem fugir dos modelos expositivos tradicionais tais como palestras e conferências, que são pouco apreciados pelos estudantes, e ser baseada em estratégias mais apelativas para transferência de informação, e que impliquem mais os estudantes, nomeadamente através do recurso a artes performativas e dinâmicas de interação grupal. Neste âmbito, as estratégias de discussão focalizada e em grupo, que foram muito apontadas pelos participantes dos grupos focais, devem ser consideradas no conjunto de medidas de intervenção. Adicionalmente, pode ser importante trabalhar as capacidades de comunicação e negociação para garantir que num contexto de relação sexual uma parte (habitualmente género feminino) adquira competências para negociar com sucesso a utilização do preservativo. A coexistência, nesta universidade, de um curso de enfermagem, e um em artes performativas pode permitir a construção de parcerias em que se podem explorar novas estratégias de comunicação como, por exemplo, seguir um caminho apontado pelos estudantes nos grupos focais, com recuso à dramatização, utilizando técnicas como o teatro fórum e o role play. Para acolher os novos alunos na universidade, é realizada habitualmente uma semana de receção ao caloiro, em que ocorrem várias atividades de desenvolvimento de competências transversais, ou soft skills. Seria importante integrar nessa semana atividades educacionais relacionadas com a redução de risco sexual (sexo seguro, utilização consistente do preservativo, redução do número de parceiros, identificação de situações de alto risco sexual) e o treino de competências (compra do preservativo, utilização correta e negociação de comportamentos de proteção, nomeadamente o uso do preservativo e testes de rastreio do VIH/SIDA e outras IST). Realçando a importância das intervenções de curta duração como as apontadas anteriormente, uma meta-análise recente (n= 20,039) que estudou a eficácia de uma única sessão de intervenção na redução de comportamentos de risco sexual (redução de sexo não protegido ou maior utilização do preservativo), demonstrou uma redução significativa nos comportamentos sexuais de risco. Os resultados do estudo indiciam que este tipo de intervenções de curta duração deve ser priorizado, até porque têm sido identificados

problemas com a adesão dos jovens a programas mais estruturados e de longa duração (Sagherian et al., 2016).

Considerando ainda o uso universal do telemóvel entre os jovens, a aplicação móvel existente na universidade pode também ser usada para divulgar mensagens curtas sobre proteção sexual, enviadas de forma periódica, mas com maior frequência do decurso das festas académicas. Das ações a desenvolver prevê-se que os estudantes passem a vir a desenvolver uma atitude mais favorável face ao sexo seguro.

A perceção de barreiras é um dos principais problemas que se pode colocar aos estudantes para adotarem o comportamento promotor de saúde e é muito determinada pela perceção de autoeficácia para realizar esse comportamento. A ideia generalizada de que o preservativo reduz o prazer está ainda muito presente na população masculina, e aparece tendencialmente nestes estudantes que tentam encontrar oportunidades para ter relações sexuais sem o usar. No contexto da negociação, principalmente em relações de namoro, acontece frequentemente a exigência de utilização de preservativo ser constrangedora para quem o pretende utilizar, uma vez que pode levantar questões sobre a fidelidade e confiança. Este aspeto, ainda que pareça de simples resolução, é de alguma complexidade nas relações que os estudantes estabelecem, pois envolve aspetos de intimidade, sensibilidade, de sentimentos profundos em relação ao companheiro e de frustração pela quebra de confiança que pode desencadear. Outra barreira percebida pelos estudantes, de natureza mais pragmática, é a adequação dos serviços de SSR. Detetamos uma lacuna entre a realidade e a resposta dos serviços de saúde. Apesar dos rapazes estarem mais expostos ao risco, a maior parte dos serviços são concebidos e organizados para mulheres, associados ao planeamento familiar, por vezes designados de "serviços de saúde da mulher", que os rapazes no estudo consideram não serem adequados às suas necessidades. O horário de funcionamento, que é frequentemente incompatível com o horário das atividades académicas, e a confidencialidade e discrição foram também apontados como aspetos que poderiam ser melhorados.

As duas primeiras barreiras apontadas exigem estratégias educativas para as ultrapassar. As que são apontadas para a variável benefícios percebidos também terão impacto na perceção de (des)confiança no parceiro, pois se a prática de utilizar o preservativo for uma rotina na relação, a questão da confiança nunca precisa de ser equacionada – mesmo quando essa confiança é traída. A alegada redução de prazer masculino quando se utiliza o preservativo é um problema que não se configura fácil de ultrapassar. Parece-nos que usar estratégias assertivas em que se incentiva o seu uso pela positiva, nos permite explorar o seu potencial hedónico e não apenas preventivo, e pode resultar numa utilização mais consistente. Uma das possibilidades a considerar, na exploração da perspetiva hedonista

que o preservativo pode ter, é a utilização de mensagens que apelem ao prazer e excitação sexual – retardar o orgasmo masculino, utilização de preservativos divertidos, com sabores ou concebidos para aumentar o prazer feminino. Contudo os custos associados à aquisição destes preservativos podem ser uma barreira que também deve ser equacionada.

Para ultrapassar as barreiras detetadas ao nível dos serviços de SSR devem ser consideradas medidas organizativas dos serviços do SNS, que estão fora do âmbito do presente trabalho. Existem, porém, pequenos investimentos, que podem ter bons resultados, nomeadamente proporcionar serviços de proximidade, com garantia da confidencialidade e com horários mais ajustados às necessidades dos estudantes. As competências de comunicação são fundamentais em todas as áreas de intervenção em enfermagem, mas assumem especial relevância quando se abordam tópicos sensíveis, como as questões da intimidade sexual, pelo que os profissionais de saúde devem desenvolver competências pessoais nesta área, que lhes permitam ser mais empáticos e assertivos nas abordagens, evitando juízos de valor e descriminação, e criar uma perpectiva de serviços de saúde " amigos dos jovens" (Kuzma & Peters, 2015). Os resultados do presente estudo, devem também ser considerados pelos profissionais de saúde no plano de intervenção para melhorar os comportamentos protetores de saúde, e que deverá passar pela adequação dos cuidados às questões de género, à idade dos estudantes e ao tipo de relação.

Os estudantes têm uma elevada autoeficácia para utilizar o preservativo, pelo menos assim o indicam os resultados da aplicação da escala de autoeficácia para usar o preservativo. Porém, como se detetou ao longo do estudo, essa autoeficácia nem sempre se reflete na sua utilização consistente. Essa inconsistência decorre essencialmente da incapacidade de negociação para a sua utilização, assim como da confiança que têm neles próprios para o fazer. Adicionalmente as barreiras percecionadas tais como o embaraço na aquisição do preservativo podem também interferir com o desempenho comportamental.

Melhorar a autoeficácia para o uso do preservativo passa por estratégias educativas com promoção social do uso do preservativo (Marketing social), que permitam desenvolver a confiança na capacidade de o adquirir e negociar o seu uso com o parceiro sexual e na sua impossibilidade, a capacidade de recusar ter relações sexuais, assim como do reforço de competências para uma correta utilização. Apontam-se como estratégias que podem ser úteis para reforçar a capacidade de negociação do preservativo a técnica de gestão de conflitos e as discussões focalizadas. Para diminuir o embaraço relacionado com a aquisição de contracetivos/preservativo, podem ser usadas estratégias mais interativas de reforço da confiança e da comunicação como o teatro fórum e role play, que podem ser dinamizadas pelos grupos de pares.

A autoeficácia assenta nos sentimentos que o estudante tem em relação à ação. Identificou-se no presente trabalho que os estudantes não têm medo das IST, porque se sentem invulneráveis, e por inerência nem consideram a hipótese de serem responsáveis pelo contágio de outrem.

As mudanças ao nível destes sentimentos assentam, inicialmente, na melhoria do conhecimento sobre as temáticas que suportam as decisões relacionadas com o comportamento sexual e, que como se observou no estudo é somente mediano, e particularmente mais fraco no que se refere às IST. As estratégias educacionais, com uma forte componente cultural, já mencionadas (teatro fórum, visionamento de filmes, debates, tertúlias, histórias de vida) e as técnicas de "confrontação pelo medo", utilizando dados dados de prevalência e incidência das principais IST na população juvenil e imagem "chocantes", podem contribuir para criar essas mudanças. A adoção de estratégias de autoafirmação, gestão stresse e de coping podem também ser úteis, ajudando os estudantes a desenvolver uma perspetiva mais otimista do problema, fazendo recurso às suas competências individuais para concretizar mudanças comportamentais.

O comportamento de saúde é passível de ser alvo de influências interpessoais, principalmente com os pares, em particular os amigos, parceiro sexual, família e profissionais de saúde. No presente estudo, as influências dos pares na utilização do preservativo foi muito positiva, para ambos os géneros. A relação com os profissionais de saúde revela fragilidades em algumas situações, nomeadamente pela falta de confiança na confidencialidade já abordada na perceção de barreiras.

As estratégias educacionais devem incorporar os resultados do estudo, propondo-se intervenções vocacionadas para estimular a mudança comportamental, nomeadamente através de programas de educação sobre saúde sexual entre pares, que podem ser desenvolvidos pela escola de enfermagem em articulação com os serviços de saúde da universidade. A forte influência dos pares para a adoção de comportamentos promotores da saúde, sugere que trabalhar estes problemas através de grupos de pares pode ser uma estratégia facilitadora, o que reflete a tendência atual, de valorização desta estratégia na promoção da saúde, na medida em que promove o "duplo empowerment", pelo desenvolvimento de competências do educando e do educador (Brito, 2009). No sentido de aumentar a perceção do risco e aumentar a pressão social dos pares, são propostas várias intervenções já mencionadas (discussões focalizada, autorreflexão, histórias de vida seguidas de debate, técnicas de role play), visando elucidar claramente os estudantes sobre medidas de prevenção, sintomas e tratamento das IST, e das sérias complicações de saúde que podem resultar destas infeções a curto e a longo prazo, particularmente para o sistema reprodutor feminino.

A nivel organizacional, devem ser consideradas estratégias de reforço das competências transversais e específicas dos profissionais de saúde, não só porque podem beneficiar a relação entre os profissionais de saúde e os estudantes, mas também permitir decisões mais informadas. Sugere-se como estratégias o desenvolvimento das competências relacionais que facilitem a comunicação/interação com os jovens, técnicas de aconselhamento sobre saúde sexual, assim como a atualização do conhecimento específico na área da sexualidade e reprodução.

A atualização do conhecimento específico revela-se particularmente importante, não só porque muitos dos conteúdos específicos relacionados com a SSR não são abordados de nos cursos de licenciatura em enfermagem, mas também porque exigem uma constante atualização, nomeadamente no que diz respeito ao aconselhamento contracetivo, pela constante evolução da informação nesta área. Neste sentido consideramos que a formação continua sobre sexualidade deveria ser assegurada pela DGS, a todos os profissionais de saúde que exercem funções nesta área, à semelhança do que acontece noutras áreas de cuidados de enfermagem onde se entende urgente a atualização. Tal como afirmam Carteiro e Nené (2015), a formação e o desenvolvimento de competências na área da sexualidade são essenciais para que os enfermeiros possam prestar cuidados holísticos, abordando a sexualidade com segurança, não como a um assunto interdito, silenciado ou invisível. Muitos profissionais demonstram ainda atitudes negativas face à sexualidade, adotando uma postura defensiva e algo conservadora, especialmente quando confrontados com situações de sexualidade não normativa. De acordo com Rowniak e Selix (2016), as escolas de enfermagem podem assumir um papel importante na construção de atitudes mais positiva fase à sexualidade, integrando programas nos programas dos cursos de licenciatura em enfermagem, de uma forma mais efetiva, conteúdos relacionados com a sexualidade ao longo do ciclo de vida, sem esquecer as questões da identidade de género.

Revela-se também importante uma atitude de autoformação, de acordo com o previsto no Artigo 88º do Estatuto da Ordem dos Enfermeiros (Decreto-Lei n.º 104/98 de 21 de abril), onde se afirma que procurando a excelência do exercício profissional, o enfermeiro assume o dever de "manter a atualização contínua dos seus conhecimentos … ". Nesta perspetiva, a formação contínua dos profissionais de enfermagem constitui não só como uma forma de desenvolvimento profissional e pessoal mas também um dever com a profissão (Rodrigues, 2011).

Ainda no núcleo central do HPM, interessa referir as influências situacionais, que foram identificadas como os contextos que são favoráveis aos comportamentos de risco sexual, resultado da associação de relações sexuais ao consumo de álcool e drogas que reduzem as capacidades cognitivas e do envolvimento com parceiros ocasionais, em particular no

decurso de festas académicas. A disponibilidade dos serviços de saúde deve também ser considerada como uma influência situacional. No presente estudo, a maioria dos estudantes refere que desconhece os serviços de saúde da universidade e a taxa de utilização dos serviços é muito baixa e exclusiva do género feminino. No entanto, verificou-se uma apreciação claramente positiva dos mesmos por parte dos estudantes, salientando a celeridade na marcação de consultas e a grande disponibilidade dos profissionais de saúde que os integram.

Para melhorar as influências situacionais, propõe-se utilizar estratégias baseadas em mensagens curtas e chocantes em períodos de festas académicas, em que os consumos de substâncias psicotrópicas aumentam exponencialmente, e ao nível organizacional, reforçar estratégias que já existem de mitigação de risco implementadas pelos serviços de saúde, como a distribuição de preservativos e aconselhamento sobre medidas de sexo seguro. No âmbito programa de Soft Skills disponibilizado pela universidade aos novos estudantes na academia no início do ano létivo, poderiam ser incluídos workshops sobre questões relacionadas com sexo seguro, prevenção de IST e a importância da vigilância regular de saúde nesta faixa etária.

No que respeita aos serviços de saúde da universidade, devem ser equacionadas estratégias de divulgação mais eficazes, dado que existe um desconhecimento significativo da sua existência e tipo de cuidados disponibilizados. A disponibilização dos serviços de saúde na universidade é de extrema importância, não só pelos cuidados de proximidade que pode proporcionar, mas também devido ao seu ambiente de expectável mentalidade aberta e não moralista. Os enfermeiros que trabalham nos serviços de SSR e que são solicitados a esclarecer questões relacionadas com sexualidade e/ou que identificam estudantes de maior risco devem aproveitar a oportunidade para informar sobre comportamentos sexuais protetores, medidas de redução de risco e referenciar para o médico, os estudantes sexualmente ativos para realizarem o rastreio de IST mais prevalentes nesta faixa etária. Ainda no que respeita aos serviços de SSR, recomenda-se a realização de ações de formação para os profissionais de enfermagem deste serviço de saúde, com o objetivo de reforçar as suas competências no domínio da educação sexual e das estratégias para comprometer os estudantes a aderir a comportamentos promotores de saúde.

Existem, porém, aspetos que prejudicam a relação entre o compromisso com a ação e o comportamento promotor de saúde propriamente dito. Os fatores competidores com a ação são aqueles que desviam o estudante da adoção do comportamento, por haver um comportamento alternativo que se configura preferencial. Aqui identificamos dois aspetos essenciais: um recupera um aspeto que também consideramos como uma barreira, que é a preferência dos rapazes por sexo sem preservativo e a outro é o sexo não planeado, que

acontece num momento de excitação sexual, em que muitas vezes o preservativo não está disponível. Consideramos que as estratégias apontadas podem contribuir para melhorar o compromisso com o comportamento promotor da saúde, que é assumido no referencial teórico do HPM de Pender, e que foi demostrado num dos artigos que compõem o presente estudo.

Em síntese, neste período de transição da vida dos jovens adultos, a autonomia e o interesse constituem-se como domínios mais (in)tensos e são pontos da maior convergência nas descobertas e no desenvolvimento da sexualidade na juventude. Confirma-se que várias são as contradições e os paradoxos inerentes à descoberta da intimidade e da sexualidade nos jovens. Trata-se de consolidar o projeto identitário do jovem e aceder à partilha da sensibilidade e da intimidade com outro, trazendo novos sentidos para a vida. Tal compromisso para consigo e para com o outro acarreta novas responsabilidades, novos conhecimentos que implica a adoção de comportamentos promotores de SSR, da sua própria saúde e a dos outros, que também se expressam numa ética quotidiana de vivência ativa da cidadania.

As instituições de ensino superior enquanto agentes de conhecimento, divulgação de saberes, desenvolvimento de competências, investigação científica e inovação, podem contribuir também para a promoção da saúde, influenciando de forma positiva a saúde e qualidade de vida seus estudantes. Assim, considerando não só a relevância dos resultados obtidos, mas também um compromisso assumido implicitamente com os estudantes, em que a voz dos estudantes e as suas preocupações nesta área influenciem as decisões, torna-se pertinente a implementação de um programa de promoção da SSR dirigido a esta população. Dado que a Universidade vem assumindo uma cultura favorecedora de saúde e bem-estar, este programa poderia ser integrado numa estratégia institucional e implementado pela parceria e partilha de sinergias entre estudantes, Escola Superior de Enfermagem e os serviços de saúde da Universidade.

REFERÊNCIAS BIBLIOGRÁFICAS

Referências bibliográficas da introdução e enquadramento conceptual

Abreu, J. F. (2010). *O conhecimento e a atitude face à saúde sexual e reprodutiva. Um estudo correlacional em estudantes universitários*. Dissertação de mestrado não publicada, Faculdade de Ciências da Universidade de Lisboa. Retirado de http://www.uwm.edu/Dept/CUTS/focus.htm

Adefuy, A.S., Abiona, T., Balogun, J. A., & Lukobo-Durrell, M. (2009). HIV sexual risk behaviors and perception of risk among college students: Implications for planning interventions. *BMC Public Health, 9(281)*. doi:10.1186/1471-2458-9-281

Ajzen, I. (1991). The theory of planned behavior. *Organizational Behavior and Human Decision Processes, 50*(2), 179-211. doi:10.1016/0749-5978(91)90020-T

Ajzen, I., & Fishbein, M. (1985). From intentions to actions: A theory of planned behavior. In J. Kuhl & J. Beckman (Eds.), *Action control: From cognition to behavior* (pp. 11-39). New York: Springer-Verlag.

Albarracín, D., Johnson, B. T., & Zanna, M. P. (2014). *The handbooh of attitudes*. New York: Psycology Press.

Albarracín, D., Johnson, B. T., Fishbein, M., & Muellerleile, P. A. (2001). Theories of reasoned action and planned behavior as models of condom use: A meta-analysis. Psychological Bulletin, 127(1), 142-161. doi:10.1037//0033-2909.127.1.142

Alli, F., Maharaj, P. & Vawda, M. Y. (2013). Interpersonal relations between health care workers and young clients: Barriers to accessing sexual and reproductive health care. *Journal Community Health*, *38*(1), 150-155. doi:10.1007/s10900-012-9595-3

Alvarez, M. J. (2005). Representações cognitivas e comportamentos sexuais de risco: O guião e as teorias implícitas da personalidade nos comportamentos de protecção sexual.

Antunes, M. T. (2010). *Atitudes e comportamentos sexuais de estudantes do ensino superior*. 777-792.

Arnett, J. J. (1998). Learning to stand alone: The contemporary American transition to adulthood in cultural and historical context. *Human Development, 41*(5/6), 295-315.

Arnett, J. J. (2004). *Emerging adulthood: The winding road from the late teens through the twenties*. New York, NY: Oxford University Press.

Artistico, D., Oliver, L. S., Dowd, S., Rothenberg, A., & Khalil, M. (2014). The predictive role of self-efficacy, outcome expectancies, past behavior and attitudes on condom use in a sample of female college students. *Journal of European Psychology Students, 5*(3), 100-107. doi:http://doi.org/10.5334/jeps.cl

Asante, K. O., & Doku, P. (2010). Cultural adaptation of the Condom Use Self Efficacy Scale (CUSES) in Ghana. *BMC Public Health, 10*, 1-7. doi:10.1186/1471-2458-10-227

Asare, M. (2015). Using the theory of planned behavior to determine the condom use behavior among college students. *American Journal of Health Studies, 30*(1), 43-50. Retrieved from https://www.ncbi.nlm.nih.gov/pmc/articles/PMC4621079/

Associação para o Planeamento da Família. (2010). *Direitos e saúde sexual e reprodutiva de jovens na Europa: Um guia para o desenvolvimento de políticas sobre direitos e*

saúde sexual e reprodutiva de jovens na Europa. Lisboa: Federação Internacional de Planeamento Familiar.

Associação para o Planeamento da Família. (2015). *Para um programa nacional de saúde sexual e reprodutiva*. Lisboa: Autor. Retirado de http://www.apf.pt/sites/default/files/media/2016/encontro_para_um_pnssr_recomendacoes_2015_05_16.pdf

Bandura, A. (1977). Self-efficacy: Toward a unifying theory of behavioral change. *Psychological Review, 84*(2), 191-215. Retirado de https://www.uky.edu/~eushe2/Bandura/Bandura1977PR.pdf

Bandura, A. (1986). *Social foundations of thought and action*. Englewood Cliffs, NJ: Prentice Hall.

Bandura, A. (1992). A social cognitive approach to the exercise of control of AIDS infection. In R. J., & DiClemente (Ed.), *Adolescent and AIDS: A generation in Jeopardy* (pp. 89-116). Newbury Park, CA: SAGE.

Bandura, A. (2006). Guide for constructing self-efficacy scales. In F. Pajares & T. Urdan (Eds.), Self-efficacy beliefs of adolescents (pp. 307-337). Charlotte: Information Age Publishing.

Barros, J., & Neves. J. (2013). Contraceção na adolescência. In J. Neves, *Contraceção* (pp. 121-134). Lisboa: Lidel.

Bermúdez, M. P., Teva, I., Ramiro, M. T., Uribe-Rodríguez, A. F., Carlos, J., & Buela-Casal, G. (2012). Knowledge, misconceptions, self-efficacy and attitudes regarding HIV: Cross-sultural assessmeent and analysis in adolescents. *International Journal of Clinical and Health Psychology, 12*, 235-249.

Black, S. R., Schmiege, S., & Bull, S. (2013). Actual versus perceived peer sexual risk behavior in online youth social networks. *Translational Behavioral Medicine, 3*(3), 312-319. doi: 10.1007/s13142-013-0227-y

Brafford, L. J., & Beck, K. H. (1991). Development and validation of a condom self-efficacy scale for college students. *Journal of American College Health, 39*(5), 219-225. doi: 10.1080/07448481.1991.9936238

Breakwell, G. M., Millward, L. J., & Fife-Schaw, C. (1994). Commitment to safer sex as a predictor of condom use among 16-20-year-olds. *Journal of Applied Social Psychology, 24*, 189-217. doi:10.1111/j.1559-1816.1994.tb00579.x

Bruni, L., Barrionuevo-Rosas, L., Albero, G., Aldea, M., Serrano, B., Valencia, S., … Castellsagué X. (2015). *ICO Information Centre on HPV and Cancer (HPV Information Centre). Human Papillomavirus and Related Diseases in the World. Summary Report 2015- 04-08*. [Data Accessed].

Burnett, A. J., Sabato, T. M., Walter, K. O., Kerr, D. L., Wagner, L. & Smith, A. (2014).The Influence of attributional style on substance use and risky sexual behaviour among college students. *College Student Journal, 48* (1), 325-336.

Carmack, C. C., & Lewis-Moss, R. K. (2009). Examining the theory of planned behaviour applied to condom use: The effect-indicator vs. Causal-Indicator models. *The Journal of Primary Prevention, 30(6), 659-676. doi: 10.1007/s10935-009-0199-3*

Carteiro, D., & Nené, M. (2015). A importância da formação na área da sexualidade em enfermagem. Revista da Associação Portuguesa dos Enfermeiros Obstetras, 16, 4-8.

Carvalho, M., & Baptista, A. (2006). O modelo informação-motivação: Aptidões comportamentais para a compreensão do uso de preservativos. In *6º Congresso Nacional de Psicologia da Saúde.* Universidade do Algarve.

Casey, M., Timmermann, L., Allen, M., Krahn, S., & Turkiewicz, K. (2009). Response and self-efficacy of condom use: A meta-analysis of this important element of AIDS education and prevention. *Southern Communication Journal, 74*(1), 57-78. doi:10.1080/10417940802335953

Cassidy, C., Curran, J., Steenbeek, A., & Langille, D. (2015). University students' sexual health knowledge: A scoping literature review. *CJNR, 47*(3), 18-38. Retirado de http://cjnr.archive.mcgill.ca/article/viewFile/2490/2484

Castro, A. (2016). Sexual Behavior and Sexual Risks Among Spanish University Students: a Descriptive Study of Gender and Sexual Orientation. Sexuality *Research and Social Policy*, 13, 84-94. doi:10.1007/s13178-015-0210-0

Catania, J., Kegeles, S., & Coates, T. J. (1990). Towards an understanding of risk behavior: An AIDS risk reduction model (ARRM). *Health Education Quarterly, 17*, 381-399.

Cavanhaug, J. (2005). *Adult development and aging*. California: Cole Publishing Company.

Centers for Disease Control and Prevention. (2014). *Sexually transmitted disease surveillance 2013*. Atlanta, Georgia: Division of STD Prevention.

Certain, H., Harahan, B., Saewyc, E., & Fleming, M. (2009). Condom use in heavy drinking college students: The importance of always using condoms. *Journal of American College Health*, *58*(3), 187-194. doi:10.1080/07448480903295284

Claxton, S. E., & van Dulman, M. H. M. (2015). Casual sexual relationships and experiences in emerging adulthood. In *The Oxford handbook of emerging adulthood* (Chap. 16, pp. 245-261). New York: Oxford University Press.

Cohen, M.S. (2004). HIV and sexually transmitted diseases: lethal synergy. *Top HIV Med*, 12, 104–7.

Conner, M., & Armitage, C. J. (1998). Extending the theory of planned behavior: A review and avenues for future research. *Journal of Applied Social Psychology, 28,* (15), 1429-1464.

Conselho de Enfermagem. (2009). *Parecer nº 109/2009: educação sexual nas escolas*. Lisboa: Ordem dos Enfermeiros. Retirado de http://www.ordemenfermeiros.pt/documentos/documents/parecer_ce-109-2009.pdf

Corrêa, S., Alves, J. E. D., & Jannuzzi, P. M. (2006). Direitos e saúde sexual e reprodutiva: Marco teórico conceitual e sistema de indicadores. In S. Cavenaghi (Org.), *Indicadores municipais de saúde sexual e reprodutiva* (Cap. 1, pp. 27-62). Rio de Janeiro: ABEP. Retirado de http://unfpa.org.br/Arquivos/indicadores.pdf

Cragg, A. et al. (2016). Sexually transmitted infection testing among heterosexual Maritime Canadian university students engaging in different levels of sexual risk taking.*Ca nadian Journal Public Health*, *107*(2), e149-54. doi:10.17269/cjph.107.5036

Cunha-Oliveira, A., Cunha-Oliveira, J., Pita, J., Massaana-Cardoso, S. (2009). A aquisição do preservativo e o seu (não) uso pelos estudantes universitários. *Referência, 11* (Série 2), 7-22.

Currie, C., Zanotti, C., Morgan, A., Currie, D., Looze, M., Roberts, C., … Barnekow, V. (Eds.). *Social determinants of health and well-being among young people: Health Behaviour in School-Aged Children (HBSC) study: International report from the 2009/2010 survey*. Copenhagen: World Health Organization.

Decreto-Lei nº 104/98, de 21 de abril. Cria a Ordem dos Enfermeiros e aprova o respectivo Estatuto. Diário da República, 93. Série I-A.

Decreto-Lei nº 259/2000, de 17 de outubro. Regulamenta a Lei n.º 120/99, de 11 de Agosto (reforça as garantias do direito à saúde reprodutiva), fixando condições de promoção da educação sexual e de acesso dos jovens a cuidados de saúde no âmbito da sexualidade e do planeamento familiar. Diário da República, 240. Série I-A.

Dehdari, T., Rahimi, T., Aryaeian, N., & Gohari, M.R. (2014). Effect of nutrition education intervention based on Pender's Health Promotion Model in improving the frequency and nutrient intake of breakfast consumption among female Iranian students. *Public Health Nutrition, 17*(3), 657-666. doi:https://doi.org/10.1017/S1368980013000049

Dellar, R. C., Dlamini, S., & Karim, Q. A. (2015). Adolescent girls and young women: key populations for HIV epidemic control. *Journal of the International AIDS Society*, 18(2Suppl 1), 19408. http://doi.org/10.7448/IAS.18.2.19408

Dembo, R., Wareham, J., Krupa, J., & Winters, K. C. (2015). Sexual risk behavior among male and female truant youths: Exploratory, multi-group latent class analysis. *Journal of Alcoholism and Drug Dependence, 3*(6), 226. doi:org/10.4172/2329-6488.1000226

Despacho nº 19737/2005, de 13 de setembro. Cria um grupo de trabalho no âmbito do Ministério da Educação com o objetivo de estudar e propor os parâmetros gerais dos programas de educação sexual em meio escolar, na perspetiva da promoção da saúde escolar. Diário da República, 176. Série 2.

Dias, C. (2007). *Utilização dos métodos contraceptivos pela população portuguesa: 4º inquérito nacional de saúde*. Comunicação apresentada no Congresso da Associação para o Planeamento da Família, 9 de setembro.

Dias, S. M. F. (2009). Comportamentos sexuais nos adolescentes: Promoção da saúde sexual e prevenção do VIH/SIDA. Lisboa: Fundação Calouste Gulbenkian e Fundação para a Ciência e a Tecnologia.

Direção-Geral da Saúde. (2006). *Programa nacional de saúde dos jovens 2006-2010.* Lisboa: Autor.

Direção-Geral da Saúde. (2008). *Saúde reprodutiva. Planeamento familiar* (ed. rev. e atual.). Lisboa: Autor. Retirado de file:///C:/Users/User/Downloads/i009646.pdf

Direção-Geral da Saúde. (2010). *Compromisso com os princípios enunciados nos objectivos para o desenvolvimento do milénio relativos à saúde materno-infantil*. Lisboa: Divisão de Saúde Reprodutiva.

Direção-Geral da Saúde. (2013). *Programa Nacional de Saúde Infantil e Juvenil*. Lisboa: Autor.

Direção-Geral da Saúde. (2015). *Orientação nº 10/2015: Disponibilidade de métodos contracetivos*. Lisboa: Autor.

Eaton, D. K., Kann, L., Kinchen, S., Shanklin, S., Ross, J., & Hawkins, J., … Wechsler, H. (2010). Youth risk behavior surveillance - United States, 2009. *Morbidity and Mortality Weely Report, 59*(SS05), 1-142.

European Center of Disease Control and Prevention. (2012). *Introduction of HPV vaccines in European Union countries: An update*. Retirado de http://ecdc.europa.eu/en/ publications/_layouts/forms/Publication_DispForm.aspx?List=4f55ad51-4aed-4d32-b960af70113dbb90&ID=677#sthash.guw T6xd5.dpuf

European Centre for Disease Prevention and Control, & WHO Regional Office for Europe. (2014). *HIV/AIDS surveillance in Europe 2013*. Stockholm: Autor.

Eurostat. (2014). *Live births by mother's age at last birthday and legal marital status*. Bruxelas: Autor.

Falcão Júnior, J. S. P., Freitas, L. V., Lopes, E., Rabelo, S. T., Pinheiro, A. B., & Ximenes, L. B. (2009). Conhecimentos de universitários da área da saúde sobre contracepção e prevenção de doenças sexualmente transmissíveis. *Enfermaria Global, 8*(15), 1-12. Retirado de http://scielo.isciii.es/pdf/eg/n15/pt_ docencia4.pdf

Faria, C. M. (2008). *Vinculação e desenvolvimento epistemológico de jovens adultos*. Tese de doutoramento não publicada, Universidade do Minho, Braga.

Feather, N. T. (1988). Values, valences, and course enrollment: Testing the role of personal values within an expectancy-value framework. *Journal of Educational Psychology, 80*, 381-391.

Ferreira, M., Ferreira, S., Ferreira, N., Andrade, J. & Duarte, J. (2014). Adesão ao Rastreio do Cancro do Colo do Útero e da Mama da Mulher Portuguesa. Millenium, 47, 83-96.

Ferreira, P. M., & Cabral, M. V. (2010). *Sexualidades em Portugal: Comportamentos de risco*. Lisboa: Editorial Bizâncio.

Fielder, R. L., & Carey, M. P. (2010). Predictors and consequences of sexual “hookups” among college students: A short-term prospective study. *Archives of Sexual Behavior, 39*(5), 1105-1119. doi:10.1007/s10508-008-9448-4

Fisher, J. D., & Fisher, W. A. (1992). Changing AIDS-risk behavior. *Psychological Bulletin, 111*(3), 455-474.

Fisher, J. D., Fisher, W. A., & Shuper, P. A. (2009). The information-motivation-behavioral skills model of HIV preventive behavior. In R. J. DiClemente, R. A. Crosby & M. C. Kegler (Eds.), Emerging theories in health promotion practice and research (pp. 21-63). San Francisco, CA: Jossey-Bass.

Flores, N. L., Balmori, Y., Vera, N. F., & Gutiérrez, H. J. (2015). Salud, calidad de vida y entorno universitario en estudiantes mexicanos de una universidad pública. *Hacia la Promoción de la Salud*, *20*(2), 102-117. doi:10.17151/hpsal.2015.20.2.8

Freitas, A., Martins, J., & Vasconcelos, R. (2003). Integração do(a)s aluno(a)s do 1º ano na Universidade do Minho. *Revista Galego-Portuguesa de Psicoloxia e Educación*, 138-1663.

Freitas, V., & Mota, C. P. (2015). Implicações da vinculação amorosa e suporte social na autoestima em jovens universitários. *Análise Psicológica*, *33*(3), 303-315. doi: 10.14417/ap.863

French, S., & Holland, K. (2013). Condom negotiation strategies as a mediator of the relationship between self-efficacy and condom use. *Journal of Sex Research, 50*(1), 48-59. doi:10.1080/00224499.2011.626907

French, S., & Holland, K. (2013). Condom negotiation strategies as a mediator of the relationship between self-efficacy and condom use. *Journal of Sex Research, 50*(1), 48-59. doi:10.1080/00224499.2011.626907

Fundo das Nações Unidas Para a População. (2005). *Situação da população mundial 2005: A promessa de igualdade: Equidade em matéria de género, saúde reprodutiva e objectivos de desenvolvimento do milénio*. Nova Iorque: Autor.

Fundo das Nações Unidas Para a População. (FNUAP) (2005). *Situação da população mundial 2005: A promessa de igualdade: equidade em matéria de género, saúde reprodutiva e objectivos de desenvolvimento do milénio*. Retirado de http://www.unric.org/pt/actualidade/6259

Geary, R. S., Gómez-Olivé, F. X., Kahn, K., Tollman, S., & Norris, S. A. (2014). Barriers to and facilitators of the provision of a youth-friendly health services programme in rural South Africa. *BMC Health Services Research*, *14*, 259. doi:10.1186/1472-6963-14-259

Gerend, M. A., & Shepherd, J. E. (2011). Correlates of HPV knowledge in the Era of HPV Vaccination: A study of unvaccinated young adult women. *Women & Health*, *51*(1), 25-40. doi:10.1080/03630242.2011.540744

Glanz, K., & Bishop, D. B., 2010. The Role of Behavioral Science Theory in Development and Implementation of Public Health Interventions. *Annual Review Public Health, 31,* 399-418. doi:10.1146/annurev.publhealth.012809.103604

Gomes, A., & Nunes, C. (2011). Caracterização do uso do preservativo em jovens adultos portugueses. *Análise Psicológica, 4*(29), 489-503.

Gomes, B. S. (1988). Para a história do planeamento familiar. Revista *da Associação para o Planeamento Familiar, 37*, 2-5.

Graça, L. (2015). Promoção da saúde: Uma abordagem positiva da saúde. In L. Santos, C. Parente, J. Ribeiro & A. Pontes (Coords.), *Promoção da saúde: Da investigação à prática* (pp. 8-14). Lisboa: SPPS, Editora.

Granja, P.M. (2009). Caracterização dos comportamentos sexuais dos adolescentes que frequentam o Olá Jovem. *Sexualidade & Planeamneto Familiar, 52/53.*

Guerreiro, M. D., & Abrantes, P. (2004). Moving into adulthood in a Southern European country: Transitions in Portugal. *Revista Portuguesa de Ciências Sociais, 3*(3), 191-209.

Guerreiro, M. D., & Abrantes, P. (2007). *Transições incertas. Os jovens perante o trabalho e a família*. Lisboa: CITE.

Haggerty, C. L., Gottlie, S. L., Taylor, B. D., Low, N., Xu, F., & Ness, R. B. (2010). Risk of sequelae after Chlamydia trachomatis genital infection in women. *Journal of Infectious Diseases, 201* (Suppl 2), S134-155.

Hickey, M. T., & Cleland, C. (2013) Sexually transmitted infection risk perception among female college students. *Journal American Association of Nurse Practitioners*, *25*(7), 377-384. doi:10.1111/j.1745-7599.2012.00791.x

Höglund, A. T., Tydén, T., Hannerfors, A. K., & Larsson, M. (2009). Knowledge of human papillomavirus and attitudes to vaccination among Swedish high school students. *International Journal of STD & AIDS, 20*, 102-107. doi:10.1258/ijsa.2008.008200

International Consortium for Emergency Contraception. (2013). *The unfnished agenda: Next steps to increase access to emergency contraception*. New York: Autor. Retirado de http://www.cecinfo.org/custom-content/uploads/2014/01/ICEC_Next-Steps-WEB_2014.pdf

Joint United Nations Programme on HIV/AIDS. (2013). *Global Report UNAIDS: Report on the global AIDS epidemic 2013*. Retirado de http://www.unaids.org/sites/default/files/media_asset/UNAIDS_Global_Report_2013_en_1.pdf

Kalina, O., Lukacs, A., Kriaucioniene, V., & Orosova (2014). Different Types of Perceived Social Support and its Role on Sexual Risk Behaviour Among Young Adults From Hungary, Lithuania and Slovakia. *Bulletin of the European Health Psychology Society, 16*.

Kann, L., Kinchen, S., Shanklin, S. L., Flint, K. H., Hawkins, J., Harris, W. A.,… Zaza, S. (2014). Youth risk behavior surveillance-United States, 2013. *Morbidity and Mortality Weekly Report, 63*(SS04), 1-168.

Kigozi, G., Wawer M., Ssettuba, A., Kagaayi, J., Nalugoda, F., Watya, S….. (2009). Foreskin surface area and HIV acquisition in Rakai, Uganda (size matters), *AIDS, 23,* 2209-22013.

Kirby, D., & Lepore, G. (2007). *Factors Affecting Teen Sexual Behavior, Pregnancy, Childbearing, And Sexually Transmitted Disease: Which Are Important? Which Can You Change*? ETR Associates.

Kirby, D., Coyle, K., Alton, F., Rolleri, L., & Robin, L., (2011). *Reducing adolescent sexual risk. Theoretical guide for developing and adapting curriculum-based programs.* California: ETR Associates.

Koshiol, J., Lindsay, L., Pimenta, J. M., Poole, C., Jenkins, D., & Smith, J. S. (2008). Persistent human papillomavirus infection and cervical neoplasia: A systematic review and meta-analysis. *American Journal of Epidemiology, 168*(2), 123-137. doi: 10.1093/aje/kwn036

Kritsotakis, G., Psarrou, M., Vassilaki, M., Androulaki, Z., & Philalithis, A. E. (2016). Gender differences in the prevalence and clustering of multiple health risk behaviours in young adults. *Journal of Advanced Nursing*, *72*(9), 2098-2113. doi:10.1111/jan.12981

Kuperberg, A., & Padgett, J. E. (2015). Dating and hooking up in college: Meeting contexts, sex, and variation by gender, partner's gender, and class standing. *The Journal of Sex Research, 52*(5), 517-531. doi:10.1080/00224499.2014.901284

Kurtinaitiene, R., Drasutiene, G., Apeikiene, L., & Ragauskyte, A. (2007). Vilnius high school student´s knowledge of cervical cancer risk factors. *Acts Medical Lithuania, 14*(4), 291-296.

Lange, I., & Vio, F. (2006). *Guía para universidades saludables y otras instituciones de educación superior.* Santiago: Productora Gráfica Andros. Retirado de http://www.eligevivirsano.cl/wp-content/uploads/2012/01/Guia-Universidades-Saludables_INTAOPS.pdf

Lazarus, J. V., Sihvonen-Riemenschneider, H., Laukamm-Josten, U., Wong, F., & Liljestrand, J. (2010). Systematic review of interventions to prevent spread of sexually transmitted infections, including HIV, among young people in Europe. *Croatian Medical Journal, 51*(1), 74-84. doi:10.3325/cmj.2010.51.74

Lei n.º 3/84, de 24 de março. Estabelece o direito à educação sexual e define as formas de acesso ao planeamento familiar. Diário da República, 71. Série I.

Lei nº 6/84, de 11 de maio. Exclusão de ilicitude em alguns casos de interrupção voluntária da gravidez. Diário da República, 109. Série I.

Lei nº 12/2001, de 29 de maio. Contraceção de emergência. Diário da República, 124. Série I-A.

Lei nº 16/2007, de 17 de abril. Exclusão da ilicitude nos casos de interrupção voluntária da gravidez. Diário da República, 75. Série I.

Lei nº 4/84, de 5 de abril. Proteção da maternidade e paternidade. Diário da República, 81. Série I.

Lei nº 60/2009, de 6 de agosto. Estabelece o regime da aplicação da educação sexual em meio escolar. Diário da República, 151. Série I.

Lei nº 90/2001, de 20 de agosto. Define as medidas de apoio às mães e aos pais estudantes. Diário da República, 192. Série I-A.

Lisboa. Fundação Calouste Gulbenkian. Fundação para a Ciência e Tecnologia. Ministério da Ciência e Ensino Superior.

Logan, D. E., Koo, K. H., Kilmer, J. R., Blayney, J. A., & Lewis, M. A. (2015). Use of drinking protective behavioral strategies and sexual perceptions and behaviors in U.S. College Students. *Journal of Sex Research, 52*(5), 558-569. doi:10.1080/00224499.2014.964167

Lopez, L. M., Otterness, C., Chen, M., Steiner, M., & Gallo, M. F. (2013). Behavioral interventions for improving condom use for dual protection (Review). *Cochrane Database Systematic Reviews, 10*. doi:10.1002/14651858.CD10662.pub2

Loureiro, I., & Miranda, N. (2010). *Promover a saúde. Dos fundamentos à acção*. Coimbra: Edições Almedina.

Mansouri, R., & Santos, X. M. (2014). Doenças sexualmente transmissíveis em adolescentes. *Patient Care, 19*(199), 23-31.

Manuel, H. I. (2007). *Conhecimentos, atitudes e práticas sobre planeamento familiar de mulheres timorenses residentes em Portugal*. Lisboa: Alto-Comissariado para a Imigração e Minorias Étnicas.

Marôco, J. P., Campos, J. A., Vinagre, M. G., & Pais-Ribeiro, J. L. (2014). Adaptação transcultural Brasil-Portugal da Escala de Satisfação com o Suporte Social para estudantes do ensino superior. *Psicologia Reflexão e Crítica*, *27*(2), 247-256.

Martins, A. T., Nunes, C., Muñoz-Silva, A., & Sánchez-García, M. (2008). Fontes de informação, conhecimentos e uso do preservativo em estudantes universitários do Algarve e de Huelva. *Psico, 39*(1), 7-13.

Martins, H. C., & Shivaji, T. (2014*). Infeção VIH/SIDA: A situação em Portugal a 31 de dezembro de 2013*. Lisboa: Instituto Nacional de Saúde Doutor Ricardo Jorge.

Marziale, M. H. P., Zapparoli, A., Felli, V. E. A., & Anabuki, M. H. (2010). Rede de prevenção de acidentes de trabalho: Uma estratégia de ensino à distância. *Revista Brasileira de Enfermagem, 63*, 250-256.

Matos, M. G., Diniz, J. A., & Simões, C. (2011). Health behaviors school: Aged children. Dados Nacionais 2010. Problemas emergentes e contextos sociais. Lisboa: Projeto Aventura Social.

Matos, M. G., Reis, M. Ramiro, L., & Equipa Aventura Social. (2011). *Saúde sexual e reprodutiva dos estudantes do ensino superior. Relatório do estudo: Dados nacionais 2010*. Lisboa: Aventura Social.

Matos, M. G., Reis, M., Ramiro, L., Pais-Ribeiro, J. L., & Leal, I. (2014). Educação sexual em Portugal: Legislação e avaliação da implementação nas escolas. *Psicologia, Saúde & Doenças*, 15(2), 335-355.

Matos, M. G., Simões, C., Camacho, I., Reis, M., & Equipa Aventura Social (2015). *A saúde dos adolescentes portugueses em tempos de recessão, dados nacionais 2014*. Lisboa: Centro de Malária e Outras Doenças Tropicais /IHMT/UNL, FMH/ Universidade de Lisboa.

Mccullagh, M. (2009). Health promotion. In S. Peterson & T. Bredow (Eds.), Middle range theories: Application to nursing research (pp. 3-45), Philadelphia: Wolters kluwer/Lippincott Williams & Wilkins.

Mello, A. L., Moysés, S. T., & Moysés, S. J. (2010). A universidade promotora de saúde e as mudanças na formação profissional. *Comunicação Saúde Educação, 14*(34), 683-692.

Melo, G.C., Trezza, M.C., Reis, R.K., Santos, D., Riscado, J.L., & Leite, J.L. (2016). Comportamentos relacionados à saúde sexual de pessoas vivendo com o Vírus da Imunodeficiência Humana. *Escola Anna Nery, 20*(1), 167-175. doi10.5935/1414-8145.20160022

Mendonça, M., Andrade, C., & Fontaine, A. M. (2009). Transição para a idade adulta e adultez emergente: Adaptação do Questionário de Marcadores da Adultez junto de jovens portugueses. *Psychologica, 51*, 147-168.

Mmari, K., & Sabherwal, S. (2013). A review of risk and protective factors for adolescent sexual and reproductive health in developing countries: An update. *Journal Adolescent Health, 53*(5), 562-572. doi:10.1016/j.jadohealth.2013.07.018

Mortensen, GL. (2010). Drivers and barriers to acceptance of human-papillomavirus vaccination among young women: a qualitative and quantitative study. *BMC Public Health, 14,* 10-68.

Neto, S., Bombas, T., Arriaga, C., Almeida, M. C., & Moleiro, P. (2014). Contraception in adolescence. Recommendations for couselling: Guidelines. *Acta Pediátrica Portuguesa, 45*, 51-63.

Neves, J. (2013). *Contraceção*. Lisboa: Lidel.

Noar, S. M. (2008). Behavioral interventions to reduce HIV-related sexual risk behavior: Review and synthesis of meta-analytic evidence. *AIDS Behavior, 12*(3), 335-353.

Nodin, N. (2001). *Os jovens portugueses e a sexualidade em finais do século XX*. Lisboa: Associação para o Planeamento da Família.

Oh, J. K., Lim, M. K., Yun, E. H., Lee, E.-H., & Shin, H.-R. (2010). Awareness of and attitude towards human papillomavírus infection and vaccination for cervical cancer prevention among adult males and females in Korea: A nationwide interview survey. *Vaccine, 28*(7), 1854-1860. doi:10.1016/j.vaccine.2009.11.079

Oliveira, A. C. (2014). *VIH/SIDA e Comportamentos de risco: Monitorizar a evolução*. Tese de doutoramento não publicada, Faculdade de Medicina da Universidade de Coimbra.

Ordem dos Enfermeiros. (1996). *Regulamento do exercício profissional do enfermeiro (REPE). Decreto-Lei n.º 161/96, de 4 de setembro (com as alterações introduzidas pelo Decreto-lei, nº 104/98 de 21 de abril).* Lisboa: Autor.

Ordem dos Enfermeiros. (2000). O ICEe a mobilização dos enfermeiros para a promoção da saúde. Lisboa. Autor.

Organização das Nações Unidas para a Educação, a Ciência e a Cultura. (2010). *Orientação técnica internacional sobre educação em sexualidade. Uma abordagem baseada nas evidências para escolas, professores e educadores em saúde.* Brasília: Divisão da Coordenação das prioridades da ONU em Educação.

Organização das Nações Unidas. (1995). *IV Conferência mundial sobre a mulher. Platforma de Ação*. Pequim.

Organização Mundial da Saúde. (1978). *Alma Ata*. Genebra: Autor.

Organização Mundial de Saúde. (1986). *Carta de Ottawa para a Promoção da Saúde*. Primeira Conferência Internacional sobre Promoção da Saúde. Ottawa. Canadá.

Organização Mundial de Saúde. (2005). *Carta de Bangkok para la promoción de la salud en un mundo globalizado*. Retirado de http://www.who.int/healthpromotion/conferences/6gchp/BCHP_es.pdf?ua=1

Ott, M. A., Sucato, G. S., & Committee on Adolescence. (2014). Contraception for adolescents. *Pediatrics, 134*(4), 1257-1281. doi:10.1542/peds.20142300.

Pacheco, A., Machado, A., Costa, A., Lanhoso, A., Cruz, E., Palma, … Gomes, V. (2011). *Consensos sobre contraceção*. Lisboa: Sociedade Portuguesa de Ginecologia. Sociedade Portuguesa de Contraceção.

Pacheco, N. S. (2012). *A sexualidade dos jovens estudantes universitários portugueses*. Tese de doutoramento não publicada, Universidade da Beira Interior, Covilhã.

Pechorro, P., Pascoal, P., Figueiredo, C., almeida, A. I., Vieira, R., & Jesus, S. (2016). Validação portuguesa da Escala de Busca de Sensações Sexuais. Revista Internacional de Andrologia, *13*(4), 1-6. doi:10.1016/j.androl.2014.11.003

Pedrosa, A., Camacho, L., passos, S., & Oliveira, R. (2011). Consumo de álcool entre estudantes universitários. *Cadernos Saúde Pública, 27*(8), 1611-1621. Retirado de http://www.scielo.br/pdf/csp/v27n8/16.pdf

Pedroso, R., & Brito, I. (2014). *Saúde dos estudantes do ensino superior enfermagem: Estudo de contexto na Escola Superior de Enfermagem de Coimbra*. Coimbra: Unidade de Investigação em Ciências da Saúde: Escola Superior de Enfermagem de Coimbra.

Pender, N. J. (1996). *Health promotion in nursing practice* (3rd ed.). Stamford, CT: Appleton & Lange.

Pender, N. J. (2011). *Heath promotion model manual*. University of Michigan. Retirado de http://hdl.handle.net/2027.42/85350

Pender, N. J., & Yang, K. P. (2002). Promotion phisical activity. *Journal of Nursing Research, 10*(1), 57-64.

Pender, N., Murdaugh, C., & Parsons, M. A. (2011). Individual models to promote health behavior. In M. Connor, D. MacKnight, K. Mortimer & S. Wrocklage (Eds.), *Health promotion in nursing practice* (pp. 35-66). New York: Pearson.

Pickens, J. (2011). Attitudes and perceptions. In N. Borkowski, *Organizational behavior in health care* (2nd ed., chap. 3, pp. 43-76). Florida: Jones and Bartlett Publishers. Retrieved from http://healthadmin.jbpub.com/Borkowski/chapter3.pdf

Pinheiro, M., & Ferreira, J. (2005). A percepção de suporte social da família e dos amigos como elementos facilitadores da transição para o ensino superior. In *Actas do VIII Congresso Galaico Português de Psicopedagogia* (pp. 467-485). Braga: Instituto de Educação e Psicologia/Centro de Investigação em Educação.

Pista, A., Oliveira, C. F., Cunha, M. J., Paixão, M. T., Real, O., & The CLEOPATRE Portugal Study. (2011). Prevalence of human papillomavirus infection in women in Portugal: The CLEOPATRE Portugal study. *International Journal of Gynecological Cancer. 2011; 21*(6), 1150-1158. doi:10.1097/IGC.0b013e31821dd3b2

Pordata, Base de Dados Portugal Contemporâneo. (2016). *Estatísticas: Educação, escolariedade da população*.

Portaria nº 196-A/2010, de 4 de abril. Regulamenta a Lei n.º 60/2009, de 6 de agosto, que estabelece o regime de aplicação da educação sexual em meio escolar.

Portaria nº 52/85, de 26 de janeiro. Regulamento das consultas de planeamento familiar e centros de atendimento para jovens. Diário da República, 22. Série I.

Porter, C. P. (2002). Female “tweens” and sexual development. *Journal of Pediatric Nursing, 17*(6), 402-406. doi:10.1053/jpdn.2002.129791

Prazeres, V. (2003). *Saúde juvenil no masculino: Género e saúde sexual e reprodutiva*. Lisboa: Direção-Geral da Saúde.

Prazeres, V., Laranjeira, A. R., Marques, A. M., Calado, B., von Ámann, G., Sassetti, L., & Cepeda, T. (2006). *Programa nacional de saúde dos jovens 2006/2010*. Lisboa: Direção-Geral da Saúde.

Precioso, J. (2004). Educação para a saúde na universidade: Um estudo realizado em alunos da Universidade do Minho. *Revista Electrónica de Ensenanza de las Ciências, 3*(2).

Prochaska, J. O., DiClemente, C. C., & Norcross, J. C. (1992). In search of how people change. Applications to addictive behaviors. *American Psychologist, 47*(9), 1102-1114.

Protogerou, C., Flisher, A. J., Wild, L., & Aarø, L. E. (2013). Predictors of condom use in South African university students: A prospective application of the theory of planned behavior. *Journal of Applied Social Psychology*, *43*(1), 23-36. doi:10.1111/jasp.12039

Ramiro, L., Reis, M., Matos, M. G., Diniz, J. A., & Simões, C. (2011). *Educação sexual, conhecimentos, crenças, atitudes e comportamentos nos adolescentes. Revista Portuguesa de Saúde Pública, 29*(1), 11-21.

Ratelle, F. C., Simard, K., & Guay, F. (2013). University students' subjective well-being: The role of autonomy support from parents, friends, and the romantic partner. *Journal of Happiness Studies, 14*, 893-910.

Regulamento n.º 127/2011, de 18 de fevereiro. Define o perfil das competências específicas do enfermeiro especialista em enfermagem de saúde materna, obstétrica e ginecológica. Diário da República, 35. Série II.

Reid, A. E., & Aiken, L. S. (2011). Integration of five health behaviour models: Common strengths and unique contributions to understanding condom use. *Psychology & Health, 26*(11), 1499-1520. doi:10.1080/08870446.2011.572259

Reis, M. (2012). *Promoção da saúde sexual em jovens universitários portugueses: Conhecimentos e atitudes face à contraceção e prevenção das IST*. Tese de doutoramento não publicada.

Reis, M., Matos, M. G., Simões, C., & Diniz, J. (2011). *Saúde sexual e reprodutiva em estudantes do ensino superior: Dados nacionais 2010: Problemas emergentes e modelo compreensivo*. Aventura social. Faculdade de Motricidade Humana/Universidade Técnica de Lisboa.

Reis, M., Ramiro, L., Matos, M. G., & Diniz, J. A. (2013). Determinants influencing male condom use among university students in Portugal. *International Journal of Sexual Health, 25*(2), 115-127. doi:10.1080/19317611.2012.728554

Remoaldo, P. C. (2001). *The past, present and future of family planning in Portugal*. Retirado de http://www.iussp.org/Brazil2001/s20/s20_PO2_Remoaldo.pdf

Resolução da Assembleia da República nº 51/98, de 2 de fevereiro. Educação sexual e planeamento familiar. Diário da República, 253. Série I-A.

Ribeiro, M. I., & Fernandes, J. (2009). Comportamentos sexuais de risco em estudantes do ensino superior público da cidade de Bragança. *Psicologia, Saúde & Doenças, 10*(1), 99-113.

Rocha, E. (2008). Universidades promotoras de saúde. *Revista Portuguesa de Cardiologia, 27*(1), 29-35.

Rodrigues, M., Pereira, A., & Barroso, T. (2005*). Educação para a saúde*. Coimbra: Formasau.

Rodrigues, P., Salvador, A., Lourenço, I., & Santos, L. (2014). Padrões de consumo de álcool em estudantes da Universidade de Aveiro: Relação com comportamentos de risco e stress. *Análise Psicológica, 32*(4), 453-466. doi:10.14417/ap.789

Royal College of Nursing. (2001). *Sexual health strategy*. Guidance for nursing staff.

Royal College of Nursing. (2014). *Defining nursing*. Retirado de https://www2.rcn.org.uk/__ data/assets/pdf_file/0003/604038/Defining_Nursing_Web.pdf

Sakraida, T. (2004). Modelo de promoção da saúde. In A. Marriner Tomey & M. Alligood (Eds.), *Teóricas de enfermagem e a sua obra: Modelos e teorias de enfermagem* (pp. 699-715). Loures: Lusociência.

Sampaio, D. (2006). *Lavrar o mar. Um novo olhar sobre o relacionamento entre pais e filhos*. Lisboa: Editora Caminhos.

Sant'Anna, M. J., Carvalho, K. A., Bastos, M. B., & Coates, P. V. (2008). Sexual behavior of young university students. *Adolescência & Saúde, 5*(2), 52-56.

Santos, A. E., Leão, F. M., Araújo, L. S., & Ferreira, L.T. (2011). *Saúde sexual e reprodutiva: Direitos e desafios em um mundo multicultural.* Retirado de http://www.sinus.org.br/2011/press/downloads/oms.pdf

Santos, I., Soares, C. S., & Berardinelli, L. M. M. (2013). Promovendo autocuidado de clientes com obesidade e coronariopatia: Aplicação do diagrama de Pender. *Revista de Enfermagem da UERJ, 21*(3), 301-306.

Santos, M. J., & Figueiredo, A. (2015). Contraceção na adolescência: aconselhamento e linhas de orientação. *Revista Electrónica de Educação e Psicologia, 2*(3), 62-75.

Santos, S. S. C., Barlem, E. L., Silva, B. T., Cestari, M. E., & Lunardi, V. L. (2008) Promoção da saúde da pessoa idosa: Compromisso da enfermagem geronto-geritrica. *Acta Paulista Enfermagem, 21*(4), 649-653.

Secginli, S., & Nahcivan, N. O. (2006). Breast self examination remains an important component of breast health: A response to Tarrant. International *Journal of Nursing Studies, 43*(4), 521-523. doi:10.1016/j.ijnurstu. 2006.02.002

Shepherd, J., Kavanagh, J., Picot, J., Cooper, K., Harden, A., Barnett-Page, E., … Price, A. (2010). The effectiveness and cost-effectiveness of behavioral interventions for the prevention of sexually transmitted infections in young people aged 13-19: A systematic review and economic evaluation. *Health Technology Assessment, 14*(7), 1-206. doi:10.3310/hta14070

Shneyderman, Y., & Schwartz, S. J. (2012). Contextual and Intrapersonal Predictors of Adolescent Risky Sexual Behavior and Outcomes. *Health Eduction and Behavior*.

Silva, A., & Brito, I. (2014). Instituições de ensino superior promotoras de saúde. In R. Pedroso & I. Brito (Eds.), *Saúde dos estudantes do ensino superior de enfermagem: Estudo de contexto na Escola Superior de Enfermagem de Coimbra* (pp.17-31).

Coimbra: Unidade de Investigação em Ciências da Saúde: Escola Superior de Enfermagem de Coimbra.

Silva, M., & Meneses, R. F. (2010). Educação para a saúde e atitudes sexuais de estudantes universitários. In *Actas do VII Simpósio Nacional de Investigação em Psicologia Universidade do Minho*, Braga.

Silva, M. O. (2013). Breves subsídios para a história da contarceção moderna em Portugal. In J. Neves. *Contraceção* (pp. 8-11). Lisboa: Lidel.

Silva, R. S., & Silva, V. R. (2011). Política nacional de juventude: Trajetória e desafios. *Caderno CRH, 24*(63), 663-678.

Simões, C., Nogueira, C., Lopes, D., Santos, N., & Peres, S. (2012). *Enfermeiros educação para a saúde, um aliado para a mudança de comportamentos*, Ordem dos Enfermeiros. Secção Regional dos Açores.

Singh, S., Darroch, J. E., & Ashford, L. S. (2014). *The costs and benefits of investing in sexual and reproductive health*. New York: Guttmacher Institute and UNFPA.

Sociedade Portuguesa de Ginecologia, & Sociedade Portuguesa da Contraceção. (2015). *Avaliação das práticas contracetivas das mulheres em Portugal, 2015*. Lisboa: Autor. Retirado de http://www.spginecologia.pt/uploads/Praticas-contracetivas-2015.pdf

Sprecher, S. (2011). The influence of social networks on romantic relationships: Through the lens of the social network. *Personal Relationships, 18*, 630-644.

Stanley, M. (2009). Early age of sexual debut: a risky experience. *Journal Family Planning Reproductive Health Care, 35,*118-120.

Steinberg, L. (2008). A social neuroscience perspective on adolescent risk-taking. *Developmental Review, 28*(1), 78-106. doi:10.1016/j.dr.2007.08.002

Straub, R. O. (2005). *Psicologia da saúde*. Porto Alegre: Artmed.

Teva, I., Bermúdez, M. P., & Buela-Casal, B. (2010). Sexual sensation seeking, social stress, and coping styles as predictors of HIV/STD risk behaviors in adolescents. *Youth & Society*, *42*(2), 255-277. doi:10.1177/0044118X09353572

Tones, K., & Tilford, S. (1994). *Health education. Efectiveness, efficiency and equity*. London: Chapman & Hall.

Trieu, S. L., Bratton, S., & Marshak, H. H. (2011). Sexual and reproductive health beahaviors of California Community College Students. *Journal of American College Health*, *59(*8), 744-750. doi:10.1080/07448481.2010.540764

Tsouros, A. D., Dowding, G., Thompson, J., & Dooris, M. (Eds.). (1998). *Health promoting universities: Concept, experience and framework for action*. Copenhagen: WHO Regional Office for Europe.

Uecker, J. E. (2015). Social context and sexual intercourse among first-year students at selective colleges and universities in the United States. *Social Science Research, 52*, 59-71. doi:10.1016/j.ssresearch.2015.01.005

Vilar, D. (1986). O estado das coisas. *Planeamento Familiar, 36*, 8-16.

Vilar, D. (2003). *Falar disso*. Porto: Afrontamento.

Vilar, D. (2008). Contributos para a história das políticas de saúde sexual reprodutiva em Portugal. *E-cadernos*, 1-28.

Vilar, D. (2009). Contributos para a história das políticas de saúde sexual reprodutiva em Portugal. *E-cadernos ces, 04*, 1-28. doi:10.4000/eces.203

Vilar, D., & Ferreira, P. M. (2010). Educação sexual dos jovens portugueses: Conhecimentos e fontes. *Educação Sexual em Rede, 5,* 2-53.

Vítor, J. F., Lopes, M. V., & Ximenes, L. B. (2005). Análise do diagrama do modelo de promoção da saúde de Nola J. Pender. *Acta Paulista de Enfermagem, 18*(3), 235-240.

Voisin, D., King, K., Schneider, J., Diclemente, R., & Tan, K. (2012). Sexual sensation seeking, drug use and risky sex among detained youth. *Journal of AIDS & Clinical Research, S1*, 1-5. doi:10.4172/2155-6113.S1-017

Walsh, J. L., Senn, T. E., Scott-Sheldon, L. A. J., Vanable, P. A., & Carey, M. P. (2011). Predicting Condom Use Using the Information-Motivation-Behavioral Skills (IMB) Model: A multivariate latent growth curve analysis. *Annals of Behavioral Medicine, 42*(2), 235-244. doi:org/10.1007/s12160-011-9284-y

Wechsler, H., Kuh, G., & Davenport, A. E. (2009). Fraternities, sororities and binge drinking: Results from a national study of American colleges. *NASPA Journal, 46*(3), 395-416. doi:10.2202/1949-6605.5017

Wicki, M., Kuntsche, E., & Gmel, G. (2010). Drinking at european universities? A review of students' alcohol use. *Addictive Behaviors, 35*(11), 913-924. doi: 10.1016/j.addbeh.2010.06.015

Wilton, L., Palmer, R. T., & Maramba, L. C. (2014). *Understanding HIV and STIs prevention for college students*. London: Routledge Research in Higher Education.

Workowski, K. A., & Bolan, G. A. (2015). Centers for Disease Control and Prevention. Sexually transmitted diseases treatment guidelines, 2015. *MMWR Recomm Rep, 64,* 1-137

World Health Organization. (1948). *Constituição da Organização Mundial da Saúde (OMS/WHO) – 1946*. Retirado de http://www.direitoshumanos.usp.br/index.php/ OMS-Organiza%C3%A7%C3%A3o-Mundial-da-Sa%C3%BAde/constituicao-da-organizacao-mundial-da-saude-omswho.html

World Health Organization. (2002). *Definition of youth*. Retirado de http://www.un.org/esa/ socdev/documents/youth/fact-sheets/youth-definition.pdf

World Health Organization. (2004). *Contraception in adolescence*. Geneve: Autor.

World Health Organization. (2010). *Social determinants of sexual and reproductivehealth: Informing future research and programme implementation*. Geneva: Autor.

World Health Organization. (2011). *The sexual and reproductive health of younger adolescents: Research issues in developing countries*. Geneva: Autor. Retirado de http://apps.who.int/iris/bitstream/10665/44590/1/9789241501552_eng.pdf

World Health Organization. (2015). *Adolescent health. World Health Statistics 2015.* Retirado de http://www.who.int/mediacentre/factsheets/fs364/en/

Yi, T.J., Shannon, B., Prodger, J., McKinnon, L., & Kaul, R. (2013). Genital immunology and HIV susceptibility in young women. *American Journal Reproductive Immunology*; *69*(1), 74-79.

Referências bibliográficas da metodologia geral

Arafat, S. M. A., Chowdhury, H. R., Qusar, M. M. S., & Hafez, A. (2016). Cross Cultural adaptation & psychometric validation of research instruments: A methodological review. *Journal Behaviour Health, 5*(3), 129-136. doi:10.5455/jbh.20160615121755

Bardin, L. (2004). *Análise de conteúdo*. Lisboa: Edições 70.

Beaton, D. E., Bombardier, C., Guillemin, F., & Ferraz, M. B. (2000). Guidelines for the process of cross-cultural adaptation of self-report measures. *Spine, 25*(24), 3186-3191.

Borsa, J. C., Damásio, B. F., & Bandeira, D. R. (2012). Adaptação e validação de instrumentos psicológicos entre culturas: Algumas considerações. *Paidéia, 22*(53), 423-432. doi:org/10.1590/1982-43272253201314

Brafford, L. J., & Beck, K. H. (1991). Development and validation of a condom self-efficacy scale for college students. *Journal American College Health*, *39,* 219-225. doi: 10.1080/07448481.1991.9936238

Brien, T. M., Thombs, D. L., Mahoney, C. A., & Wallnau, L., (1994). Dimensions of self-efficacy among three distinct groups of condom users. *Journal American College Health, 42*(4), 167-174.

Brown, T. A. (2016). *Confirmatory factor analysis for applied research*. Boston: Todd D. Little.

Davis, M., & Yarber, W. L., Bauseserman, R., Schreer, G., & Davis, S. L. (1998). *Handbook of sexuality-related measures.* London: Sage.

Dias, S. F., & Rocha, C. F. (2009). *Saúde sexual e reprodutiva de mulheres imigrantes africanas e brasileiras: Um estudo qualitativo*. Lisboa: Alto-comissariado para a imigração e Diálogo Intercultural.

Ferreira, T., Ribeiro, J. L. P., & Guerreiro, M. (2004). Estudo de adaptação da escala da satisfação com o suporte social em doentes submetidos a revascularização do miocárdio. *Revista Referência, 11,* 12.

Flick, U. (2005). *Métodos qualitativos na investigação científica*. Coimbra: Edição Monitor.

Fortin, M.F., Côté, J., & Filion, F. (2009). *Fundamentos e etapas do processo de Investigação*. Loures: Lusodidacta.

Gil, A. C. (1999). *Métodos e técnicas de pesquisa social* (5a ed.). São Paulo: Atlas.

Gill. P., K. Stewart, K., Treasure, E., & Chadwick, B. (2008). Methods of data collection in qualitative research: Interviews and focus groups. *British Dental Journal, 204,* 291-295. doi:10.1038/bdj.2008.192

Hair, J. F. Jr., Black, W. C., Babin, B. J., & Anderson, R. E. (2006). *Multivariate data analysis* (7th ed.). London: Pearson New International Edition.

Hambleton, R. K. (2005). Issues, designs, and technical guidelines for adapting tests into multiple languages and cultures. In R. K. Hambleton, P. F. Merenda & C. D.

Spielberger (Eds.), *Adapting educational and psychological tests for cross-cultural assessment* (pp. 3-38). Mahwah, NJ: Lawrence Erlbaum.

Hills, M. M., & Hill, A. (2012). *Investigação por questionário* (3a ed.). Lisboa: Edições Silabo.

Hollander, J. (2004). The social context of focus groups. *Journal of Contemporary Ethnography, 33*(5), 602-637.

Hyde, A., Howlett, E., Brady, D., & Drennan, J. (2005). The focus group method: Insights from focus group interviews on sexual health with adolescents. *Social Science and Medicine, 61*, 2588-2599.

Jayasekara, R. S. (2012). focus groups in nursing research: methodological perspectives. *Nursing Outlook, 60*(6), 411-416. doi:org/10.1016/j.outlook.2012.02.001

Kalichman, S. C., & Rompa, D. (1995). Sexual sensation seeking and sexual compulsivity scales: Reliability, validity, and predicting HIV risk behavior. *Journal of Personality Assessment, 65*(3), 586-601. doi:10.1207/s15327752jpa6503_16

Kline, R. B. (2005). *Principles and practice of structural equation modeling* (2nd ed.). New York: Guilford.

Krueger, R. A., & Casey, M. A. (2009). *Focus groups: A practical guide for applied research* (4th ed.). San Francisco: Sage.

Lobdell, D., Gilboa, S., Mendola, P., & Hesse, B. (2005). Use of focus groups for the environmental health researcher. *Journal of Environmental Health*, *67*(9), 36-42.

Marôco, J. (2011). *Análise de equações estruturais: Fundamentos teóricos, software & aplicações*. Pêro Pinheiro: Report Number.

Marôco, J. (2014). *Análise estatística com o SPSS Statistics*. Pero Pinheiro: Report Number.

Marôco, J. P., Campos, J. A., Vinagre, M. G., & Pais-Ribeiro, J. L. (2014). Adaptação transcultural Brasil-Portugal da Escala de Satisfação com o Suporte Social para Estudantes do Ensino Superior. *Psicologia Reflexão e Crítica, 27*(2), 247-256.

Marôco, J., & Garcia-Marques, T. (2006). Qual a fiabilidade do alfa de Cronbach? Questões antigas e soluções modernas?. *Laboratório de Psicologia, 4*(1), 65-90.

Morgan, D. L. (2000). *Focus groups as qualitative research* (2nd ed., vol. 16). London: Sage.

Oliveira, D. L. (2011). The use of focus groups to investigate sensitive topics: An example taken from research on adolescent girls' perceptions about sexual risks. *Ciência & Saúde Coletiva, 16*(7), 3093-3102. doi:org/10.1590/S1413-81232011000800009

Pestana, M. H., & Gageiro, J. N. (2014). *Análise de dados para ciências sociais: A Complementaridade do SPSS* (6a ed.). Lisboa: Edições Sílabo.

Peterson-Sweeney, K. (2005). The use of focus groups in pediatric and adolescent research. *Journal of Pediatric Health Care*, *19*(2), 104-110.

Porta, M. (2008). *A dictionary of epidemiology* (5th ed.). New York: Oxford University Press.

Ribeiro, J. L. (2010). *Investigação e avaliação em psicologia da saúde* (2a ed.). Lisboa: Placebo Editora.

Ribeiro, J. L. P. (1995). Adaptação de uma escala de avaliação da autoeficácia geral. *Avaliação Psicológica: Formas e Contextos, 3*,163-176.

Ribeiro, J. L. P. (2007). *Metodologia de investigação em psicologia e saúde*. Porto: Legis Editora.

Richardson, R. J. (1999). *Pesquisa social: Métodos e técnicas* (3a ed.). São Paulo: Atlas.

Santos, A. E., Leão, F. M., Araújo, L. S., & Ferreira, L. T. (2011). *Saúde sexual e reprodutiva: Direitos e desafios em um mundo multicultural*. Retirado de http://www.sinus.org.br/2011/press/downloads/oms.pdf

Santos, C., Pais Ribeiro, J., & Lopes, C. (2003). Estudo de adaptação da Escala de Satisfação com o Suporte Social (ESSS) a pessoas com diagnóstico de doença oncológica. *Psicologia, Saúde & Doenças, 4*(2), 185-204.

World Health Organization. (2011). *The sexual and reproductive health of younger adolescents: Research issues in developing countries*. Geneva: Autor. Retirado de http://apps.who.int/iris/bitstream/10665/44590/1/9789241501552_eng.pdf

Worthington, R. W., & Whittaker, T. A. (2006). Using exploratory and confirmatory fator analysis in scale development research: A content analysis and recommendations for best practices. *Counseling Psychologist, 34*(6), 806-838.

Referências das linhas orientadoras de promoção da saúde sexual e reprodutiva para estudantes do ensino superior

Brito, I. (2009). Promoção da saúde nos jovens utilizando a educação pelos pares: Intervenção com estudantes de enfermagem e jovens enfermeiros. *Enfermagem e o Cidadão*, *19*, 6-9.

Carteiro, D., & Nené, M. (2015). A importância da formação na área da sexualidade em enfermagem. *Revista da Associação Portuguesa dos Enfermeiros Obstetras, 16,* 4-8.

Decreto-Lei nº 104/98, de 21 de abril. Cria a Ordem dos Enfermeiros e aprova o respectivo Estatuto. Diário da República, 93. Série I-A.

Kuzma, E. K., & Peters, R. M. (2015). Adolescent Vulnerability, sexual health, and the NP´s role in health advocacy. *Journal of the American Association of Nurse Practioners*, *28*(7), 353-361. doi:10.1002/2327-6924.12331

Ordem dos Enfermeiros. (2000). *O ICE e a mobilização dos enfermeiros para a promoção da saúde*. Lisboa. Autor.

Rodrigues, S. V. (2011). *Aprendizagem dos enfermeiros ao longo da vida: Adaptação e validação da escala de Jefferson*. Dissertação de mestrado não publicada, Instituto de Ciências da Saúde da Universidade Católica Portuguesa.

Rowniak, S., & Selix, N. (2016). Preparing nurse practitioners for competence in providing sexual health care. *Journal of Association Nurses AIDS Care*, *27*(3), 355-361.doi: 10.1016/j.jana.2015.11.010

Sagherian, M. J., Huedo-Medina, T. B., Pellowski, J. A., Eaton, L. A., & Johnson, B.T. (2016). *Single-session behavioral interventions for sexual risk reduction: A meta-analysis*. *The Society of Behavioral Medicine, 50,* 920-934. doi:10.1007/s12160-016-9818-4.

APÊNDICES

APÊNDICE A

Protocolo de investigação

SAÚDE SEXUAL E REPRODUTIVA DE ESTUDANTES DO ENSINO SUPERIOR

Número do Questionário_____________

Este questionário faz parte de um projeto de investigação do doutoramento em Ciências de Enfermagem do ICBAS da Universidade do Porto, e têm como finalidade conhecer os determinantes de saúde sexual e reprodutiva de estudantes do ensino superior, para que, com base nos resultados se possam delinear estratégias de intervenção para a construção de programas de promoção da saúde sexual e reprodutiva em estudantes do ensino superior.

A sua concretização só será possível com a sua colaboração, para tal, solicitamos a sua participação, respondendo às questões que lhe são colocadas. O questionário **é anónimo e confidencial.**

Pedimos-lhe que responda a todas as perguntas de uma forma espontânea e sincera, de acordo com aquilo que faz, sente ou pensa. Não existem respostas corretas ou incorretas, **o que nos interessa é a sua opinião.**

Nas afirmações onde existir uma quadrícula (☐), deve assinalar com uma cruz (X) a(s) alínea(s) que está(ão) de acordo com o seu caso.

Nas questões com um espaço em branco (_____), deve responder claramente e de forma legível.

Para que seja salvaguardada a validade do questionário, pedimos que não deixe nenhuma questão por responder, desde que esteja de acordo com a sua situação.

Desde já agradecemos a sua colaboração e disponibilidade

Nota: Ao longo do questionário não utilizamos a duplicidade de género (o/a) (os/as), porque consideramos a pessoa na sua diversidade.

1. Género: 1 ☐ Masculino 2 ☐ Feminino **2. Idade**: _____ anos **3. Nacionalidade**_____________________________

4**. Curso que frequenta** _________________________________**5. Ano do curso** 1 º☐ 2º☐ 3 º☐ 4º☐ 5º ☐

6. Zona habitacional onde cresceu: 1 ☐ Aldeia 2 ☐ Vila 3 ☐ Cidade

7. Estado civil: 1 ☐ Solteiro 2 ☐ Casado 3 ☐ União de facto 4 ☐ Divorciado 5 ☐ Viúvo

8. Qual a importância da religião para si?
1 ☐ Muito Importante 2 ☐ Importante 3 ☐ Pouco Importante 4 ☐ Nada Importante 5 ☐ Não tenho Religião

9. Qual a profissão dos seus pais? (seja especifico e se forem reformados ou estiverem desempregados mencione a profissão anterior) **9.1 Mãe** __________________________**9.2. Pai**____________________

10. Qual a escolaridade dos seus pais?
10.1. Mãe: 1 ☐ 1º Ciclo 2 ☐ 2º Ciclo 3 ☐ 3º Ciclo 4 ☐ Secundário 5 ☐ Licenciatura 6 ☐ Mestrado/Doutoramento
10.2. Pai : 1 ☐ 1º Ciclo 2 ☐ 2º Ciclo 3 ☐ 3º Ciclo 4 ☐ Secundário 5 ☐ Licenciatura 6 ☐ Mestrado/Doutoramento

11. Qual o rendimento médio do seu agregado familiar? (sabendo que o salário mínimo em Portugal é de **485 Euros**)
1 ☐ Menos de 2 salários mínimos 2 ☐ 2 Salários mínimos 3 ☐ 4 Salários mínimos 4 ☐ Mais de 4 salários mínimos

12. Para si, qual foi a fonte de informação mais importante sobre sexualidade e saúde reprodutiva? (pode responder mais de uma opção)
1 ☐ Médicos
2 ☐ Enfermeiros
3 ☐ Professores
4 ☐ Amigos
5 ☐ Pai
6 ☐ Mãe
7 ☐Outro familiar
8 ☐ Centro de Atendimento a Jovens (CAJ)
9 ☐ Internet

10 ☐ Meios de comunicação social
11 ☐ Livros e revistas científicas
12 ☐ Outra, Qual?___________________

13. Já teve relações sexuais? 1 ☐ Sim 2 ☐ Não

(Se **NÃO** teve relações sexuais passe para a questão **14**)

13. 1. Se sim, idade da 1ª relação sexual: _____________ anos

13.2. Qual o método contracetivo que utilizou na sua 1ª relação sexual?

1 ☐ Nenhum
2 ☐ Preservativo
3 ☐ Pílula
4 ☐ Pílula e preservativo
5 ☐ Coito interrompido
6 ☐ Métodos naturais (calendário, temperatura, muco cervical)
7 ☐ Outro, Qual?_________________________

14. Como se considera relativamente à orientação sexual?

1 ☐ Heterossexual 2 ☐ Bissexual 3 ☐ Homossexual 4 ☐ Não sei

15. No último ano teve relações sexuais? 1 ☐ Sim 2 ☐ Não

(Se **NÃO** teve relações sexuais passe para a questão **31 se é Rapariga e 34 se é Rapaz)**

16. As relações sexuais aconteceram no contexto de uma relação amorosa? 1 ☐ Sim 2 ☐ Não

16.1. Se sim, há quanto tempo mantinha essa relação amorosa quando teve relações sexuais?

1 ☐ Menos de 1 mês 2 ☐ 3 meses 3 ☐ 6 meses 4 ☐ 1 ano 5 ☐ Mais de 1 ano

17. Utiliza contraceção? 1 ☐ Sim 2 ☐ Não

17.1. Se sim, qual o método contracetivo?

1 ☐ Preservativo
2 ☐ Pílula
3 ☐ Pílula e Preservativo
4 ☐ Coito interrompido
5 ☐ Anel vaginal
6 ☐ Adesivo contracetivo
7 ☐ Implante
8 ☐ Métodos naturais (calendário, temperatura, muco cervical)
9 ☐ Outro, Qual? _________

Se é Rapariga

18. No último ano se utilizou a pílula, alguma vez se esqueceu de a tomar?

1 ☐ Nunca 2 ☐ Pelo menos uma vez 3 ☐ Várias vezes

18.1. Se já esqueceu alguma vez a toma da pílula, o que fez?

1 ☐ Tomei o comprimido esquecido quando me lembrei
2 ☐ Tomei o comprimido no dia seguinte de acordo com o esquema normal
3 ☐ Usei o preservativo
4 ☐ Tomei a contraceção de emergência
5 ☐ Outra situação?____

19. Onde obteve informação sobre o método contracetivo que costuma utilizar? (pode responder mais de uma opção)

1 ☐ Médicos
2 ☐ Enfermeiros
3 ☐ Professores
4 ☐ Amigos
5 ☐ Pai
6 ☐ Mãe
7 ☐ Outro familiar
8 ☐ Centro de Atendimento a Jovens (CAJ)
9 ☐ Farmácia
10 ☐ Internet
11 ☐ Meios de comunicação social
12 ☐ Livros e Revistas científicas
13 ☐ Outro, Qual? ____________________________

20. Onde adquiriu o método contracetivo que costuma utilizar? (pode responder mais de uma opção)

1 ☐ Farmácia 2 ☐ Centro de Saúde 3 ☐ Supermercado/Loja 4 ☐ Outro Local, Qual? _______________________

21. Já utilizou contraceção de emergência / pílula do dia seguinte (no caso dos rapazes a companheira)**?**

1 ☐ Sim 2 ☐ Não

(Se **NÃO** utilizou contraceção de emergência passe para a questão **23)**

22.1. Se sim, quantas vezes ______________

22.2. Onde adquiriu a contraceção de emergência?

1 ☐ Farmácia (sem receita médica)
2 ☐ Farmácia (com receita médica)
3 ☐ Centro de Saúde
4 ☐ Hospital
5 ☐ Centro de Atendimento a Jovens
6 ☐ Outro Local, Qual? ____________________________

22.3. Qual o motivo para ter usado contraceção de emergência?

1 ☐ Falha no método contracetivo habitual (rotura do preservativo, esquecimento pontual da pilula,…)
2 ☐ Não usar contraceção regular
3 ☐ Outro motivo, Qual?_________________________

22.4. Foi-lhe dada alguma informação sobre a contraceção de emergência? 1 ☐ Sim 2 ☐ Não

22.5. Se sim, qual?

1 ☐ Modo de utilização 2 ☐ Efeitos adversos 3 ☐ Contraindicações 4 ☐ Todas as informações anteriores

23. No último ano quando teve relações sexuais, utilizou sempre o preservativo?

1 ☐ Sim, sempre 2 ☐ Frequentemente 3 ☐ Raramente 4 ☐ Nunca 5 ☐ Não se aplica (não tive relações sexuais)

23.1. Qual o motivo para não ter utilizado sempre o preservativo?

1 ☐ Não tinha o preservativo disponível no momento
2 ☐ Confio no meu parceiro
3 ☐ Tenho uma relação de longa duração apenas com este parceiro sexual
4 ☐ Uso outro método contracetivo
5 ☐ Não gosto de usar, porque diminui o prazer sexual
6 ☐ Não sei usar corretamente
7 ☐ Opção própria

24. Já teve relações sexuais associadas ao consumo de álcool?

1 ☐ Nunca 2 ☐ Pelo menos uma vez 3 ☐ Várias vezes **24.1. Quantas vezes no último ano?** _______

25. Já teve relações sexuais associadas ao consumo de drogas?

1 ☐ Nunca 2 ☐ Pelo menos uma vez 3 ☐ Várias vezes **25.1. Quantas vezes no último ano?** _______

26. Já teve relações sexuais com parceiros ocasionais?

1 ☐ Nunca 2 ☐ Pelo menos uma vez 3 ☐ Várias vezes **26.1. Quantas vezes no último ano**? _______

28. Já teve alguma Infeção Sexualmente Transmissível (IST)?

1 ☐ Sim 2 ☐ Não 3 ☐ Não sei

(Se **NÃO** teve nenhuma **IST** passe para a questão **29)**

28.1. Se sim, qual foi a IST? ___________________________

28.2. Se sim, onde recorreu para obter o tratamento?
1 ☐ Farmácia 2 ☐ Médico de Família 3 ☐ Hospital 4 ☐Serviços Médicos Privados 5 ☐ Não fiz tratamento

28.3. Se sim, informou o seu parceiro sexual que estava com uma IST?
1 ☐ Sim 2 ☐ Não

28.4. Se não, Porquê? ____

29.1. Ao adotar comportamentos preventivos (usar o preservativo) estou a prevenir, complicações de saúde reprodutiva
Discordo completamente 1 ☐ 2 ☐ 3 ☐ 4 ☐ 5 ☐ 6 ☐ 7 ☐ Concordo completamente

29.2. Para mim é fácil usar o preservativo no meu dia-a-dia
Discordo completamente 1 ☐ 2 ☐ 3 ☐ 4 ☐ 5 ☐ 6 ☐ 7 ☐ Concordo completamente

29.3. Ir buscar preservativos ao centro de saúde é uma situação embaraçosa
Discordo completamente 1 ☐ 2 ☐ 3 ☐ 4 ☐ 5 ☐ 6 ☐ 7 ☐ Concordo completamente

29.4. Falar com os profissionais de saúde sobre assuntos relacionados com o uso de contracetivos pode ser embaraçoso
Discordo completamente 1 ☐ 2 ☐ 3 ☐ 4 ☐ 5 ☐ 6 ☐ 7 ☐ Concordo completamente

29.5. Comprar preservativos é uma situação embaraçosa, porque expõe a minha intimidade
Discordo completamente 1 ☐ 2 ☐ 3 ☐ 4 ☐ 5 ☐ 6 ☐ 7 ☐ Concordo completamente

29.6. Usar e dialogar sobre os métodos contracetivos (preservativo) faz parte de uma sexualidade responsável
Discordo completamente 1 ☐ 2 ☐ 3 ☐ 4 ☐ 5 ☐ 6 ☐ 7 ☐ Concordo completamente

29.7. Sinto-me melhor comigo próprio quando uso o preservativo
Discordo completamente 1 ☐ 2 ☐ 3 ☐ 4 ☐ 5 ☐ 6 ☐ 7 ☐ Concordo completamente

29.8. Usar o preservativo interfere com o prazer sexual
Discordo completamente 1 ☐ 2 ☐ 3 ☐ 4 ☐ 5 ☐ 6 ☐ 7 ☐ Concordo completamente

29.9. Ficaria incomodado se o meu parceiro insistisse para usarmos o preservativo
Discordo completamente 1 ☐ 2 ☐ 3 ☐ 4 ☐ 5 ☐ 6 ☐ 7 ☐ Concordo completamente

29.10. As pessoas que são importantes para mim, aconselham-me a ter sempre comigo um preservativo?
Discordo completamente 1 ☐ 2 ☐ 3 ☐ 4 ☐ 5 ☐ 6 ☐ 7 ☐ Concordo completamente

29.11. É importante que os parceiros sexuais falem sobre o uso do preservativo
Discordo completamente 1 ☐ 2 ☐ 3 ☐ 4 ☐ 5 ☐ 6 ☐ 7 ☐ Concordo completamente

29.12. É divertido ter experiências sexuais ocasionais
Discordo completamente 1 ☐ 2 ☐ 3 ☐ 4 ☐ 5 ☐ 6 ☐ 7 ☐ Concordo completamente

29.13. Uma boa maneira de obter prazer sexual é tendo relações sexuais sob o efeito de álcool ou drogas
Discordo completamente 1 ☐ 2 ☐ 3 ☐ 4 ☐ 5 ☐ 6 ☐ 7 ☐ Concordo completamente

29.14. Há uma grande probabilidade de eu usar o preservativo no próximo mês
Discordo completamente 1 ☐ 2 ☐ 3 ☐ 4 ☐ 5 ☐ 6 ☐ 7 ☐ Concordo completamente

29.15. Se tiver relações sexuais no próximo mês tenciono usar sempre o preservativo
Discordo completamente 1 ☐ 2 ☐ 3 ☐ 4 ☐ 5 ☐ 6 ☐ 7 ☐ Concordo completamente

30. Já teve alguma gravidez indesejada ou engravidou a sua companheira?
1 ☐ Sim 2 ☐ Não 3 ☐ Não tenho a certeza **Se sim, quantas vezes?** ______________

(Se **NÃO** houve gravidez passe para a questão **32 se é rapariga ou 35 se é rapaz)**

31. O que aconteceu com a gravidez?
1 ☐ A gravidez está a decorrer 2 ☐ Optamos pela interrupção da gravidez (IVG)
3 ☐ Ocorreu aborto espontâneo 4 ☐ O bebé nasceu

Se é Rapariga

32. Foi vacinada contra o Vírus do Papiloma Humano (HPV)?
1 ☐ Sim 2 ☐ Não 3 ☐ Desconheço essa necessidade

33. Faz citologia vaginal (Papanicolau)?
1 ☐ Sim 2 ☐ Não 3 ☐ Desconheço essa necessidade

33.1. Se faz citologia vaginal, quando a realiza?
1 ☐ Uma vez por ano 2 ☐ De 2/2 anos 3 ☐ De 5/5 anos

34. Faz o autoexame da mama regularmente?
1 ☐ Sim 2 ☐ Não 3 ☐ Desconheço essa necessidade

Se é Rapaz

35. Faz o autoexame dos testículos? 1 ☐ Sim 2 ☐ Não 3 ☐ Desconheço essa necessidade

36. Já fez o teste do VIH/SIDA?
1 ☐ Sim 2 ☐ Não 3 ☐ Considero que nunca estive exposto à infeção

37. Já recorreu aos serviços de saúde reprodutiva?
1 ☐ Sim 2 ☐ Não 3 ☐ Desconheço a sua existência

37.1. Se sim, onde recorreu?
1 ☐ Centro de Saúde
2 ☐ Hospital
3 ☐ Centro de Atendimento a Jovens (CAJ)
4 ☐ Consultório Privado
5 ☐ Serviços de Saúde da Universidade/ Faculdade/ Escola

37.2. Qual o motivo que o levou a recorrer aos serviços de saúde sexual e reprodutiva?
1 ☐ Aconselhamento sobre contraceção
2 ☐ Pedir um método contracetivo
3 ☐ Realizar vigilância médica de rotina
4 ☐ Diagnóstico ou tratamento de infeções sexualmente transmissíveis
5 ☐ Realizar citologia vaginal (papanicolau)
6 ☐ Realizar teste de gravidez
7 ☐ Interrupção da gravidez
8 ☐ Outro motivo, **Qual ?**_____________________

37.3. Teve dificuldades no acesso aos serviços de saúde sexual e reprodutiva?
1 ☐ Horários pouco convenientes
2 ☐ Localização pouco conveniente
3 ☐ Demora na marcação da consulta
4 ☐ Pouca disponibilidade dos profissionais de saúde
5 ☐ Falta de confidencialidade
6 ☐ Medo de ser reconhecido
7 ☐ Receio que os pais tivessem conhecimento
8 ☐ Não tive dificuldades no acesso

38. Tem conhecimento da existência de serviços de saúde sexual e reprodutiva na sua Universidade/ Escola?
1 ☐ Sim 2 ☐ Não têm disponível este serviço 3 ☐ Desconheço a existência deste serviço

39. Na sua opinião o que se poderia mudar para que os serviços de saúde e sexual e reprodutiva fossem de encontro às necessidades dos jovens?
1 ☐ Maior divulgação
2 ☐ Horários de atendimento mais alargados
3 ☐ Garantir o anonimato e confidencialidade
4 ☐ Maior abertura e disponibilidade dos profissionais de saúde
5 ☐ Disponibilidade destes serviços na Universidade / Escola
6 ☐ Transmitir informação de formas inovadoras (palestras, sessões informativas, exposições de arte e atividades artísticas)

40. Indicam-se abaixo algumas afirmações relativas ao **Conhecimento Sobre Saúde Sexual e Reprodutiva.** Diga qual o seu grau de concordância com cada uma delas, assinalando a opção que considera correta.

**Conhecimento Sobre Saúde Sexuel e Reprodutiva**	**Verdadeira**	**Falsa**	**Não Sei**
1. Os espermatozoides têm um tempo médio de vida no interior do aparelho reprodutor feminino de cerca de 72 horas			
2. O período de fertilidade do óvulo varia entre 4 a 5 dias após ovulação			
3. A pílula é um método abortivo porque impede a implantação no útero de um ovo já fecundado			
4. O 1º dia de um ciclo menstrual corresponde ao 1º dia da menstruação			
5. Os espermicidas são compostos por substâncias que eliminam a mobilidade dos espermatozoides			
6. Algumas infeções sexualmente transmissíveis podem ser transmitidas pela mãe ao bebé durante a gravidez e parto			
7. A mulher pode engravidar com maior facilidade entre o 11º dia e o 16º dia, a contar do primeiro dia da menstruação			
8. Na suspeita de uma gravidez, o teste de gravidez para ser fiável deve ser realizado 3 dias após a data prevista para a menstruação			
9. A pílula evita a gravidez, porque impede a ovulação através da ação hormonal			
10. A pílula pode piorar a tensão pré-menstrual e a dor relacionada com o período menstrual			
11. O diagnóstico das infeções sexualmente transmissíveis é fácil porque tem sintomas específicos e visíveis			
12. Quando o esquecimento da toma da pílula é inferior 24 horas, devemos continuar a tomar, incluindo o último comprimido esquecido, sem necessidade de um método adicional			
13. O prurido, ardor e dor ao urinar podem ser sintomas de uma infeção de transmissão sexual			
14. Logo que a rapariga começa a tomar a pílula fica imediatamente protegida contra uma gravidez não desejada			
15. A pilula reduz o risco de cancro do ovário e do endométrio			
16. Os vírus que infetam a área anogenital podem ser transmitidos durante o sexo vaginal, oral ou anal, ou durante o contacto íntimo de pele com pele			
17. Os antibióticos diminuem o efeito da pílula			
18. Uma pessoa que tem um teste de VIH positivo tem SIDA			
19. A utilização de um lubrificante adequado com o preservativo masculino reduz a probabilidade de rutura durante o sexo anal			
20. O anel vaginal pode ser retirado durante a relação sexual e colocado até 6 horas depois, sem existirem problemas de contraceção			
21. O método do coito interrompido é eficaz na prevenção da gravidez			
22. As infeções de transmissão sexual estão relacionadas com situações de infertilidade			
23. O tratamento das infeções sexualmente transmissíveis, deve ser feito sempre aos dois parceiros sexuais mesmo quando não existem sintomas			

Conhecimento Sobre Saúde Sexuel e Reprodutiva	Verdadeira	Falsa	Não Sei
24. O preservativo feminino tem a mesma eficácia que o masculino na prevenção da gravidez e infeções sexualmente transmissíveis			
25. O risco de ter uma infeção sexual aumenta sempre que temos um novo parceiro sexual			
26. As infeções de transmissão sexual transmitem-se apenas numa relação sexual desprotegida com penetração			
27. O dispositivo intrauterino (DIU) é um método contracetivo recomendado para jovens			
28. O anel vaginal é um método contracetivo que ao ser colocado na vagina inibe a entrada dos espermatozoides			
29. Um único contato sexual é suficiente para uma pessoa adquirir uma infeção sexualmente transmissível			
30. O implante hormonal é um método contracetivo de longa duração e pode ser usado em qualquer idade			
31. A contraceção de emergência (pílula do dia seguinte) deve ser utilizada quando existe uma suspeita de gravidez por atraso na menstruação			
32. A contraceção de emergência (pílula do dia seguinte) pode ser usada com segurança no máximo até 2 dias após relação sexual não protegida			
33. Uma das complicações das infeções sexualmente transmissíveis na mulher é a doença inflamatória pélvica			
34. É impossível adquirir uma infeção de transmissão sexual em casas de banho públicas ou ginásios			
35. A citologia vaginal (papanicolau) deve ser realizada anualmente por todas as mulheres com ou sem atividade sexual, a partir dos 18 anos			
36. Depois de uma relação sexual sem preservativo, deve ser realizado o teste para despiste de infeções sexualmente transmissíveis imediatamente			
37. O adesivo contracetivo e o anel vaginal podem não ser eficazes em caso de vómitos e diarreia			
38. A vacinação contra o vírus do papiloma humano (HPV) é importante para prevenir o cancro do colo do útero			
39. Os rapazes são biologicamente mais suscetíveis às infeções sexualmente transmissíveis do que as raparigas			
40. A vacinação contra o HPV está recomendada para mulheres a partir dos 18 anos			
41. A mulher pode engravidar se tiver relações sexuais 4 a 5 dias depois da ovulação			
42. O sexo oral desprotegido é menos arriscado do que o sexo vaginal desprotegido, relativamente à transmissão do VIH/SIDA			
43. A citologia vaginal (papanicolau), permite diagnosticar precocemente as infeções de transmissão sexual			
44. As infeções de transmissão sexual são fáceis de tratar e normalmente os sintomas não voltam a aparecer			

41. Indicam-se abaixo algumas afirmações relativas a **Atitudes Face à Saúde Sexual e Reprodutiva em Jovens Adultos.** Diga qual o seu grau de concordância com cada uma delas, mesmo que não tenha tido relações sexuais, assinalando a opção que melhor correspondente à sua opinião, considerando o esquema de resposta indicado.

**Atitudes Face à Saúde Sexual e Reprodutiva em Jovens Adultos**	**Discordo Totalmente**						**Concordo Totalmente**
	**1**	**2**	**3**	**4**	**5**	**6**	**7**
1. A afetividade é muito importante num relacionamento sexual							
2. É importante que os parceiros sexuais falem sobre os métodos de contraceção							
3. Se o meu parceiro sexual tivesse relações sexuais com outra pessoa gostaria de ter conhecimento							
4. Usar métodos contracetivos faz a relação amorosa parecer mais permanente							
5. Devemos iniciar relações sexuais com um novo parceiro quando temos a certeza que estamos preparados para assumir essa responsabilidade							
6. O uso de contracetivos é apenas da responsabilidade da mulher pois é ela que engravida							
7. Para as mulheres a sexualidade é tão importante como para os homens							
8. A satisfação sexual é muito importante num relacionamento amoroso							
9. Conversar sobre as infeções de transmissão sexual podem comprometer o relacionamento amoroso							
10. A colocação do preservativo pode ser um jogo erótico							
11. Considero importante saber o passado sexual do meu parceiro							
12. A masturbação é uma prática sexual saudável							
13. A escolha do método de contracetivo deve ser realizada após o aconselhamento por um profissional de saúde							
14. Falar com os profissionais de saúde sobre o uso de contracetivos é embaraçoso							
15. A masturbação é tão normal nas mulheres como nos homens							
16. Devemos ser capazes de recusar ter relações sexuais sem preservativo							
17. O uso do preservativo mostra falta de confiança no nosso parceiro sexual							
18. Se me fosse diagnosticada uma infeção sexualmente transmissível conversaria com o meu parceiro sobre o assunto							
19. Quando conhecemos o nosso parceiro sexual não precisamos de usar o preservativo							
20. Se o meu parceiro insistisse para usarmos preservativo ficaria incomodado							
21. A maioria das infeções de transmissão sexual não interfere com a relação amorosa							
22. Quando se toma a pílula não é necessário utilizar o preservativo							
23. Para mim a elevada taxa de infeção de transmissão sexual é uma preocupação							
24. É um insulto sugerir ao meu parceiro o uso do preservativo mesmo que para prevenir uma infeção de transmissão sexual							
25. Não me sinto à vontade para falar sobre as infeções de transmissão sexual com os meus amigos							
26. É divertido ter experiências sexuais ocasionais							

Atitudes Face à Saúde Sexual e Reprodutiva em Jovens Adultos	Discordo Totalmente						Concordo Totalmente
	1	2	3	4	5	6	7
27. Fico orgulhoso quando consigo convencer o meu parceiro a usar o preservativo							
28. Considero que a maioria das infeções de transmissão sexual não interfere com a vida quotidiana							
29. É importante saber se o nosso parceiro sexual realizou o teste do VIH/ SIDA							
30. É mais importante a nossa saúde que ter uma breve experiência sexual							
31. Se o meu parceiro não gosta de utilizar o preservativo devo respeitar a sua vontade e ter relações sexuais sem preservativo							
32. A possibilidade de vir a ter cancro do útero ou infertilidade é motivo suficiente para modificar o comportamento sexual							
33. Se tivesse mais de um parceiro sexual faria testes de saúde com regularidade							
34. Considero importante convencer o meu parceiro a praticar apenas sexo seguro							
35. As pessoas que não têm relações sexuais não necessitam de ir regularmente ao Médico (Ex. ginecologista)							
36. Usar o preservativo interfere com o prazer sexual							
37. As infeções sexualmente transmissíveis têm pouco impacto no bem-estar físico e psicológico dos jovens							
38. Quando tenho relações sexuais o meu maior receio é a possibilidade de engravidar							

42. Indicam-se abaixo algumas afirmações relativas à **Procura de Sensações Sexuais.**
Assinale a opção que melhor traduz a sua opinião, considerando o esquema de resposta indicado.

**Procura de Sensações Sexuais** (Adaptada da Sexual Sensation Seeking Scale (Kalichman & Rompa, 1995)	Nunca se aplica a mim	Por vezes aplica-se a mim	Algumas vezes aplica-se a mim	Muitas vezes Aplica-se a mim
1. Gosto de encontros sexuais desinibidos				
2. As sensações físicas são a coisa mais importante do sexo				
3. Gosto da sensação de ter relações sexuais sem preservativo				
4. Os meus parceiros sexuais, veem-me, provavelmente, como alguém que gosta de "correr riscos"				
5. No que diz respeito ao sexo, a atracão física é mais importante para mim do que conhecer bem a pessoa				
6. Aprecio a companhia de pessoas "sensuais"				
7. Gosto de ver vídeos pornográficos				
8. Já disse coisas que não eram necessariamente verdade, para levar as pessoas a terem relações sexuais comigo				
9. Estou interessado em experimentar novas sensações sexuais				
10. Apetece-me explorar a minha sexualidade				
11. Gosto de experiências e sensações sexuais novas e excitantes				

**43**. Indicam-se abaixo algumas afirmações relativas ao **uso do preservativo masculino,** qual o grau de concordância com cada uma delas, considerando o esquema de resposta indicado.

**Autoeficácia para Usar o Preservativo**<br>(Adaptada da Condon Use Self Efficacy Scale, versão reduzida de Brien et al., 1994)	**Discordo Totalmente**				**Concordo Totalmente**
	**0**	**1**	**2**	**3**	**4**
1. Estou confiante na minha capacidade de colocar um preservativo em mim ou no meu parceiro					
2. Estou confiante na minha capacidade de discutir o uso do preservativo com qualquer parceiro que possa ter					
3. Estou confiante que seria capaz de sugerir o uso do preservativo a um novo parceiro					
4. Estou confiante que seria capaz de sugerir o uso de um preservativo sem que o meu parceiro se sentisse doente					
5. Se tivesse de sugerir o uso de um preservativo a um parceiro teria medo que ele/ela me rejeitasse					
6. Caso não tivesse certeza da opinião do meu parceiro(a) em relação ao uso do preservativo não seria capaz de sugerir o seu uso					
7. Estou confiante que seria capaz de retirar corretamente o preservativo depois de termos relações sexuais					
8. Não me sinto confiante para sugerir o uso de preservativo a um novo parceiro(a) pois temo que ele/ela pense que tive uma experiência homossexual					
9. Não me sinto confiante para sugerir o uso de preservativo a um novo parceiro(a) pois temo que ele/ela pense que tenho uma infeção sexualmente transmissível					
10. Não me sinto confiante para sugerir o uso de preservativo a um novo parceiro pois temo que ele/ela pense que eu possa estar a achar que ele/ela tem uma infeção sexualmente transmissível.					
11. Estou confiante na minha capacidade de colocar um preservativo em mim ou no meu parceiro rapidamente					
12. Tenho a certeza que seria capaz de me lembrar de usar um preservativo mesmo depois de ter bebido álcool					
13. Estou confiante que seria capaz de me lembrar de usar um preservativo mesmo depois de ter consumido drogas					
14. Estou confiante na minha capacidade para usar um preservativo corretamente					
15. Estou confiante que seria capaz de colocar um preservativo em mim ou no meu parceiro mesmo no “calor da paixão”					

44. Os itens que se seguem procuram medir qual o **grau de satisfação que manifesta perante o apoio social que habitualmente recebe**. Leia cuidadosamente cada afirmação e assinale a opção que melhor traduz a sua forma de pensar, considerando o esquema de resposta indicado.

**Escala de Satisfação Com o Suporte Social** (Versão Portuguesa de Ribeiro, J.L.P., 1999)	**Discordo Totalmente**	**Discordo**	**Não Concordo Nem Discordo**	**Concordo**	**Concordo Totalmente**
1. Por vezes sinto-me só no mundo e sem apoio					
2. Não saio com amigos tantas vezes quantas eu gostaria					
3. Os amigos não me procuram tantas vezes quantas eu gostaria					
4. Quando preciso de desabafar com alguém encontro facilmente amigos com quem o fazer.					
5. Mesmo nas situações mais embaraçosas, se precisar de apoio de emergência tenho várias pessoas a quem posso recorrer					
6. Às vezes sinto falta de alguém verdadeiramente íntimo que me compreenda e com quem possa desabafar sobre coisas íntimas					
7. Sinto falta de atividades sociais que me satisfaçam					
8. Gostava de participar mais em atividades de organizações (ex. clubes desportivos, partidos políticos, etc.)					
9. Estou satisfeito com a forma como me relaciono com a minha família					
10. Estou satisfeito com a quantidade de tempo que passo com a minha família					
11. Estou satisfeito com o que faço em conjunto com a minha família					
12. Estou satisfeito com a quantidade de amigos que tenho					
13. Estou satisfeito com a quantidade de tempo que passo com os meus amigos					
14. Estou satisfeito com as atividades e coisas que faço com o meu grupo de amigos					
15. Estou satisfeito com o tipo de amigos que tenho					

Obrigada pela sua colaboração

APÊNDICE B

Autorização dos autores para utilização das escalas

Maria José De Oliveira Santos

**De:**	Thombs, Dennis <Dennis.Thombs@unthsc.edu>
**Enviado:**	terça-feira, 22 de Janeiro de 2013 20:02
**Para:**	Maria José De Oliveira Santos
**Assunto:**	RE: Scale use permission request

Maria: you have my permission to use the brief version of the CUSES. The scale is not a copyrighted instrument. Good luck with your research!

Dennis L. Thombs, PhD, FAAHB
Professor and Chair
Department of Behavioral and Community Health - EAD 711 School of Public Health
3500 Camp Bowie Blvd.
UNT Health Science Center
Fort Worth, TX 76107-2699
(817) 735-5439

-----Original Message-----
From: Maria José De Oliveira Santos [mailto:mjsantos@utad.pt]
Sent: Tuesday, January 22, 2013 1:39 PM
To: Thombs, Dennis
Subject: Scale use permission request

Dear Professor Dennis L. Thombs

I am professor/researcher at School of Nursing - University of Trás-os-Montes e Alto Douro (UTAD), Portugal. I am preparing a PhD in the subject of reproductive and sexual health in young adults (university students) in Portugal.

The approach we intend to use involves the evaluation of knowledge/attitude on reproductive and sexual issues of the target population through the use of instruments to assess different parameters of the population. I am contacting you in order to request authorization to use (and validate) the reduced version of CUSES (Tina M. Brien MSEda, Dennis L. Thombs PhDb, Colleen A. Mahoney PhDb & Larry Wallnau PhD, 1994) in the portuguese context.

Thanks in advance for your valuable collaboration, Waiting for your feed-back, Best regards,

Maria José Santos

ESEVR-UTAD Lugar do Tojal,

Lordelo

5000-232 Vila Real, Portugal

Maria José De Oliveira Santos

**De:**	Seth Kalichman <sethckal@gmail.com>
**Enviado:**	segunda-feira, 29 de Outubro de 2012 19:29
**Para:**	Maria José De Oliveira Santos
**Assunto:**	Re: Scale use permission request

Hi
Yes you have permission to use the scale. Good luck.

Seth Kalichman
Sent from my remote office

On Oct 29, 2012, at 8:34 AM, Maria José De Oliveira Santos <mjsantos@utad.pt> wrote:

> Dear Professor Seth Kalichman,
>
> I has been trying to contact you by email, but I am not sure if I have the correct email address. Can you please confirm if you received this email.
> Thanks for your attentions,
> Best regards,
>
> Maria José Santos
>
>
> ESEVR-UTAD
> Lugar do Tojal, Lordelo
> 5000-232 Vila Real, Portugal
> Email- mjsantos@utad.pt
>
> -----Mensagem original-----
> De: Maria José De Oliveira Santos
> Enviada: domingo, 21 de Outubro de 2012 16:57
> Para: seth.k@uconn.edu
> Assunto: Scale use permission request
>
> Dear Professor Seth Kalichman,
>
> I am professor/researcher at School of Nursing - University of Trás-os-Montes e Alto Douro (UTAD), Portugal. I am preparing a PhD thesis in the subject of reproductive and sexual health in young adults (university students) in Portugal.
> The approach we intend to use involves the evaluation of knowledge/attitude on reproductive and sexual issues of the target population through the use of instruments to assess different parameters of the population. Among these, we would like to assess the Sexual Sensation Seeking Scale (SSSS, Kalichman and Rompa, 1995).
> I am contacting you in order to request authorization to use the SSSS in our work.
>
> If you consider pertinent, fell free to ask for detsils of the research project.
>
> Thanks in advance for your valuable cooperation, Waiting for your
> feed-back, Best regards, Maria José Santos, ESEVR-UTAD Lugar do Tojal,
> Lordelo
> 5000-232 Vila Real, Portugal

APÊNDICE C

Informação transmitida aos participantes convidados a integrar os grupos focais sobre os procedimentos e condições de recolha de dados

Caro Estudante

No último ano letivo participou num estudo sobre **Saúde Sexual e Reprodutiva em estudantes do Ensino Superior**, com o preenchimento de um questionário. Mais uma vez agradeço a sua valiosa colaboração.

No sentido de dar continuidade ao estudo complementando a informação já recolhida torna-se necessário realizar alguns grupos focais, pelo que vimos por este meio solicitar a sua colaboração para participar. A sua participação é de extrema importância pois visamos desenvolver um programa de promoção da saúde sexual e reprodutiva, específico par a os estudantes do Ensino Superior, tendo por base os resultados obtidos.

Informamos que:

- Os grupos focais serão realizados no **dia 10 de abril a partir das 16 horas** e terão em média uma duração de 2 horas (1º grupo das 16 às 18h e 2ª grupo das 18 às 2oh). A atividade será composta por uma dinâmica de "quebra-gelo" seguida da discussão focalizada;

- Cada grupo deverá integrar cerca de **12 estudantes duma amostra representativa de estudantes dos diferentes cursos que integram as cinco Escolas da UTAD,** selecionados aleatoriamente dentro do grupo de estudantes que mostrem disponibilidade para participar no estudo;

- A atividade será realizada numa das **salas da biblioteca da UTAD,** evitando desta forma que os estudantes tenham de sair do campus universitário;

- Se o desejar, poderá desistir em qualquer momento, não existindo qualquer implicação na sua desistência.

- Asseguramos mais uma vez que todos os dados serão colhidos de forma anónima e confidencial e que o estudo está validado pela Comissão de Ética da UTAD;

- Todos os detalhes sobre a atividade, **nomeadamente a confirmação da data e hora** da mesma serão enviados para o seu e-mail ou caso o deseje para o seu contato telefónico que poderá enviar.

Agradeço desde já sua valiosa participação, certa de que entende do seu interesse para todos os estudantes.

A**gradeço que confirme via correio eletrónico se está ou não interessado em participar nesta fase do estudo**.

Ao seu dispor para qualquer esclarecimento adicional,

Com os melhores cumprimentos, Maria José Santos

Maria José Santos
Departamento de Enfermagem de Saúde Materna e Infantil
Escola Superior de Enfermagem de Vila Real – UTAD
Email: mjsantos@utad.pt
TM: 919136696

APÊNDICE D

Questões orientadoras dos grupos de discussão focalizada

SAÚDE SEXUAL E REPRODUTIVA EM ESTUDANTES DO ENSINO SUPERIOR

GRUPOS DE DISCUSSÃO FOCALIZADA SOBRE SAÚDE SEXUAL E REPRODUTIVA - QUESTÕES ORIENTADORAS

Componentes Modelo HPM	Eixos Interpretativos	Questões Orientadoras	Unidades de Registo Exemplificativas
**CARACTERÍSTICAS**	**Comportamentos Individuais e Experiências**	Na vossa opinião quais os motivos que levam os jovens a iniciarem relações sexuais?	*"Eu acho que há cada vez mais isso, e há necessidade de afirmação no grupo. E1.3.*<br>*"Não é só a pressão dos pares é a pressão no geral, do mundo em geral*
		As relações sexuais acontecem em relações ocasionais ou numa relação amorosa?	*"Começam cedo e chega a uma altura em que quer arriscar mais, não chega um só companheiro, e mais do que um, dois ou três…" E2.4.*<br>*"E mesmo nas relações de longa duração não são bem assim […], pelo meio existem sempre alguns parceiros ocasionais." E2.1.*
		Utilizam métodos contracetivos? Quais?	*"A mim fornecem-me no centro de saúde, dão-me um envelope lacrado com 30 preservativos de 3 em 3 meses." E 2.2.*<br>*"No meu caso, vou buscar a pílula ao instituto de que falei, o IPJ." E1.2.*
		De quem é a decisão sobre o método contracetivo a dotar?	*"A contraceção é responsabilidade das raparigas". E2.6.*<br>*" […] o homem tem um papel tão importante como a mulher nesse aspeto do controle e prevenção da saúde sexual e também na contraceção […], mas nas consultas de planeamento familiar, em 80% dos casos só lá vão mulheres" E2.4.*
		Quando utilizam o preservativo qual é a vossa maior preocupação?	*"Com o meu namorado a minha preocupação não é se tem uma doença ou não, é mesmo a gravidez". E1.1.*<br>*"Acho que é mesmo a questão de prevenir a gravidez". E1.3.*
		Os jovens conhecem e utilizam a contraceção de emergência?	*" Sim, e eu conheço casos de pessoas que tomaram a pílula do dia seguinte N vezes".E1.1.*
		Quando iniciam uma relação amorosa tem preocupação em saber o passado sexual do vosso companheiro?	*"Eu sim, eu converso, eu partilho, tem de ser, senão não me sentiria à vontade na relação." E2.3.*<br>*"Eu coloco tudo em pratos limpos o que é que eu fiz, o que não fiz, com à vontade, […] e depois tento saber sempre o que ela já fez ou não fez." E1.4.*
		Na vossa opinião os jovens correm mais riscos sexuais? Quais são os riscos?	*"Acho que os principais são mesmo o sexo desprotegido, com vários parceiros, aumenta muito o risco.". E1.5.*<br>*"Começam muito cedo e chega a uma altura em que quer arriscar mais, não chega um só companheiro, e mais do que um, dois ou três… E2.4.*

Componentes Modelo HPM	Eixos Interpretativos	Questões Orientadoras	Unidades de Registo Exemplificativas
CONHECIMENTO E SENTIMENTOS SOBRE COMPORTAMENTO SEXUAL SEGURO	Benefícios	Consideram importante a utilização do preservativo?	*"Temos de usar, no meu caso, conheço pessoas que já apanharam doenças, já aqui na universidade". E1.1.*<br>*"Podemos não ficar grávidas mas as doenças existem…". E2.9.*
		Consideraram importante falar com o parceiro sexual sobre contraceção ou sobre comportamentos de risco sexual?	*"Eu acho que a comunicação deve estar sempre presente e a prevenção também". E2.4*<br>*"Eu coloco tudo em pratos limpos o que é que eu fiz, o que não fiz, com à vontade, […] e depois tento saber sempre o que ela já fez ou não fez". E1.4.*
		Consideram importante recorrer aos serviços e fazer vigilância de SSR?	*"Eu continuo a ir ao IPJ porque sinto que tenho um bom acompanhamento." E1.2.*<br>*" Acabei por ir (centro de saúde) e acho que me ajudou muito, esclareci muitas dúvidas que tinha e com frequência quando as tenho ou me acontece alguma coisa é lá que vou". E2.3*<br>*" Acho também importante referir que nós temos os serviços de ação social aqui da UTAD, que são gratuitos, pelo menos a partir do ano passado. Eu já tive acesso e eles são sem dúvida uma ajuda". E2.1*
	Barreiras	Estão descritas baixas taxas de utilização do preservativo pelos estudantes universitários. Na vossa opinião porque será que isto acontece?<br>Quais são os principais motivos para jovens não utilizarem o preservativo?	*"Dá mais prazer sem preservativo. Qualquer preservativo retira algum prazer à relação sexual" E1.5.*<br>*"Ela pedir-me a mim para não usar o preservativo? Já aconteceu… mas comigo é diferente… já há uma base de confiança." E1.4.*<br>*"Os casos que eu conheço, que dispensam a utilização do preservativo, são os que já tem uma relação de muito tempo". E1.2.*<br>*" Eu acho que ainda há aquela vergonha… principalmente da parte masculina, embora nas raparigas também". E2.6.*<br>*"Mas são caros, não são grátis". E1.5*
		Na vossa opinião o que pode limitar o acesso aos serviços de SSR?	*"O tempo de espera pela consulta".E1.4*<br>*"Eu acho que não é vergonha, deve-se ter mais atitude, é hoje vou…. Acho que*

Componentes Modelo HPM	Eixos Interpretativos	Questões Orientadoras	Unidades de Registo Exemplificativas
CONHECIMENTO E SENTIMENTOS SOBRE COMPORTAMENTOS SEXUAIS SEGUROS			*é isso, deixam passar". E1.8*<br>*" Em meios pequenos como o nosso há muitos boatos". E1.9*<br>*"Toda a gente fica a saber o que vamos fazer lá basicamente". E1.1*<br>*"É a maneira como nos olham…". E1.5*
	**Sentimentos**	Existe da vossa parte medos relacionados com a sexualidade? Quais são?	"*Ficar grávida na nossa idade, acho que é uma das maiores preocupações*". E1.7.<br>"*A mim o que me assusta são as doenças, sempre que ouvimos esses números dos 70% (pessoas infetadas com HPV)* ". E1.1.
	**Autoeficácia**	Os jovens sabem que o preservativo é uma forma eficaz de proteção, mas não o utilizam de forma consistente. Porquê?	*"Apesar de termos aquela convicção, preservativo sempre. Nós temos essa noção só que depois as coisas na prática são diferentes [...].E2.1.*<br>*"Sim, só na hora conseguimos decidir se usamos ou não, e nem sempre usamos". E2.8*
		Seriam capazes de recusar ter relações sexuais se não tivessem preservativo (num momento de paixão)?	*"Sim, porque se ele não tem preservativo ela não tem de aceitar fazer sem… No meu caso, ou usa o preservativo ou não acontece nada." E1.5*<br>*"Quem normalmente recusa usar é o homem, sempre". E2.6*
	**Influências Interpessoais**	Quais foram as fontes de informação ou pessoas mais importantes para obterem conhecimento sobre sexualidade, contraceção, IST…?<br>Com quem se sentem mais à vontade para falarem (no sentido de obter informação) sobre estas questões?	*"[…] foi o grupo de pares, o meu grupo de colegas. Podia não ser a informação mais figdigna, mas partia sempre dai. Depois, claro, tentava pesquisar onde podia." E2.4.*<br>*"Mas com as minhas amigas não falo da parte da saúde". E.1.1*<br>*"Dúvidas mesmo e coisas importantes é mesmo com os profissionais de saúde […] quando tenho dúvidas sobre alguma coisa têm de ser mesmo com o enfermeiro, com quem posso esclarecer dúvidas e falar sobre tudo." E2.2.*<br>*" A maneira mais fácil é com aquela amiga." […] achei sempre que as minhas amigas e a internet, era suficiente". E2.5*<br>*"Eu não tenho intimidade para falar com os meus pais, para falar com eles diretamente". E2.7*

Componentes Modelo HPM	Eixos Interpretativos	Questões Orientadoras	Unidades de Registo Exemplificativas
**CONHECIMENTO E SENTIMENTOS SOBRE COMPORTAMENTOS SEXUAIS SEGUROS**		Consideram importante falara com o vosso companheiro sobre as IST e estado serológico para o VIH/ SIDA?	*"Eu acho que isso é cada vez mais importante questionar sobre isso, e realizarmos o teste, os dois." E1.3.*<br>*"Eu também acho que sim, e por exemplo fazer um teste do VIH/SIDA deveria ser obrigatório" E2.4.*
	**Influências Situacionais**	Consideram que há momentos ou contextos que acarretam um maior risco sexual? (Semana académica, festas, saídas à noite, viagens de curso)?	*" Consome-se muito álcool e por vezes outras coisas…para além do álcool. Mistura-se muita coisa sim, sim …mas são principalmente as misturas" E1.5.*<br>*" Nesses momentos uma pessoa alcoolizada não vai pensar em responsabilidades […] e acontecem relações sexuais em que por vezes não há nada, nem pílula, nem preservativo".E2.4*<br>*" Na queima, sim sexo nas casas de banho… em público… forte".E1.3*<br>*" Não sei se é o ambiente académico só por si, são as festas, o álcool, a desinibição das pessoas…". E2.6*
		Na vossa opinião as relações com parceiros ocasionais acontecem frequentemente? Porquê?	*" Sim muitas vezes associadas ao álcool, o objectivo é desinibir" E1.4*<br>*"É aquela ideia, uma vez não faz mal, com ela não faz mal, com ela também não, para eles está tudo bem…E2.6*
		Vocês costumam recorrer aos SSR? Sabem onde podem recorrer?	*"Não, no geral os jovens não recorrem, tem um bocado de vergonha, sentem-se intimidados.".E1.3*<br>*"É verdade, eu não vou aos serviços, eu já fiz algumas perguntas a farmacêuticos e enfermeiros, mas nunca para mim, eu pergunto para saber em relação à minha parceira". E1.4*<br>*"Médica de família, qualquer dúvida, médico de família." E1.1.*<br>*"[…] eu nunca fui ao médico de família sem a minha mãe, nunca senti essa necessidade de ir, porque achava que supostamente já sabia tudo" E1.10*<br>*" Eu utilizo os serviços de saúde da Universidade". E2.1*
		Quais os motivos que levam os jovens a procurarem estes serviços?	*"No meu caso vou buscar a pílula ao instituto que falei o IPJ." E1.2.*<br>*"Nós sabemos que a partir do momento em que iniciamos relações sexuais, nós raparigas, temos de fazer o 1º papanicolau" E1.3*
		Consideram que estes serviços devem estar disponíveis na Universidade?	*"Seria importante divulgar mais que temos cá esse serviço de saúde na universidade, porque eu não sabia. Acho que está pouco divulgado." E1.3*

Componentes Modelo HPM	Eixos Interpretativos	Questões Orientadoras	Unidades de Registo Exemplificativas
**RESULTADO DO COMPORTAMENTO**	**Plano de Intervenção para Promoção de Comportamentos Sexuais Seguros**	Na vossa opinião o que poderia mudar para que a educação sexual e os serviços de SSR, fossem de encontro às necessidades específicas dos jovens adultos nesta área?	*"Acho que poderia ajudar termos reuniões mais focadas em alguns temas, por exemplo sobre as IST. E se alguém pudesse estar presente e dar o seu testemunho." E2.4.*<br>*"Exatamente, histórias, narrativas. Acho que é isso que nos cativa mais e nos chama mais atenção.E2.*<br>*"Se fossem pessoas do meu curso não apareciam nas palestras, não apareciam em nada, isso eu garanto, pessoas do meu curso não dá" E1.1*<br>*"Acho que a informação deveria ser acompanhada por esses tais casos de chocantes, ou de coisas que chamem a atenção dos riscos que as pessoas correm". E1.2.*<br>*"Sim, se as imagens fossem chocantes, nós olhávamos para lá". E2.8.*<br>*" Acho que é mais pelo vídeo, pelo teatro, pelo cinema. Aquilo que vemos fica". E2.4.*<br>*"Como há consultas quando somos mais novos, também deveria haver quando somos mais velhos, quer iniciássemos relações sexuais quer não se iniciasse". E1.3*<br>*"Eu também acho que sim, e por exemplo fazer um teste do VIH/SIDA deveria ser obrigatório. Em termos de saúde pública deveria ser". E2.4*
Alguma coisa que queiram acrescentar…			

APÊNDICE E

Questionário de caracterização dos participantes dos grupos focais

Universidade do Porto
Instituto de Ciências Biomédicas
Abel Salazar

SAÚDE SEXUAL E REPRODUTIVA EM ESTUDANTES DO ENSINO SUPERIOR

1 – CARACTERIZAÇÂO

1. Género: ☐ Masculino ☐ Feminino

2. Idade: ______

3. Naturalidade____________________

4.Local de residência

5. Curso que frequenta ___________________________

5. Ano do Curso __________

2 – COMPORTAMENTOS DE SAÚDE SEXUAL E REPRODUTIVA

1. Atualmente têm relações sexuais? ☐ Sim ☐ Não

2. Utiliza contraceção? ☐ Sim ☐ Não

3. Qual o método contracetivo?____________________________

4. Na última relação sexual utilizou o preservativo? ? ☐ Sim ☐ Não

5. Faz vigilância de saúde sexual e reprodutiva regularmente? ☐ Sim ☐ Não

6. Se não porquê?____

Muito obrigada pela colaboração

APÊNDICE F

Autorização da Comissão Nacional de Proteção de dados **(Nº 7409/2013)**

COMISSÃO NACIONAL
DE PROTECÇÃO DE DADOS

AUTORIZAÇÃO Nº 7409 /2013

I. Do Pedido

Maria José de Oliveira Santos, no âmbito da sua Tese de Doutoramento, notificou à CNPD um tratamento de dados pessoais com a finalidade de realização de um estudo observacional e intervencional sobre a saúde sexual e reprodutiva em estudantes do ensino superior e a eficácia de um programa de intervenção.

O estudo está divido em duas fases, sendo a primeira caracterizada por uma vertente quantitativa, através da aplicação de questionários anónimos a estudantes das escolas da Universidade de Trás-os-Montes e Alto Douro, e por uma vertente qualitativa, pela constituição de três *focus groups* de doze estudantes universitários que voluntariamente se ofereçam para colaborar nesta investigação. Já a segunda fase do estudo visa testar a eficácia de uma intervenção formativa e implica a aplicação de questionários sobre os conhecimentos sobre a saúde sexual e reprodutiva e comportamentos adotados a um grupo experimental e a um grupo de controlo, antes e após a implementação do programa de intervenção.

Os questionários recolhem informação sociodemográfica, escala de avaliação do nível de conhecimentos em saúde sexual e reprodutiva, escala de atitudes face à saúde sexual e reprodutiva, escala de autoeficácia geral, escala de autoeficácia no uso do preservativo, escala de procura de sensações sexuais e escala de satisfação com o suporte sexual.

A investigadora solicitará o consentimento informado e escrito aos participantes no estudo, cuja declaração conservará em local de acesso reservado. Contudo, na vertente qualitativa da primeira fase do estudo, o consentimento será implícito ao preenchimento dos questionários, de modo a não afetar o seu caráter anónimo.

Os dados são recolhidos de forma direta, junto dos titulares dos dados, mediante questionários de autopreenchimento e discussões em *focus groups*.

COMISSÃO NACIONAL
DE PROTECÇÃO DE DADOS

No "caderno de recolha de dados" não há identificação nominal do titular, sendo aposto um código de participante. A chave desta codificação só será conhecida da investigadora.

Aos titulares dos dados é assegurado o direito de conhecer e corrigir os dados que lhes respeitem.

A segurança das informações é garantida pela manutenção dos dados em local de acesso reservado.

Os destinatários serão ainda informados sobre a natureza facultativa da sua participação e será garantida confidencialidade no tratamento.

II. Da Análise

Porque em grande parte referentes à saúde e à vida privada, os dados recolhidos pela requerente têm a natureza de sensíveis, nos termos do disposto no n.º 2 do artigo 7.º da LPD.

Em regra, o tratamento de dados sensíveis é proibido, de acordo com o disposto no n.º 1 do artigo 7.º da LPD.

Todavia, o tratamento de dados de saúde é permitido, quando haja uma disposição legal que consagre esse tratamento de dados, quando por motivos de interesse público importante o tratamento for indispensável ao exercício das atribuições legais ou estatutárias do seu responsável ou quando o titular dos dados tiver prestado o seu consentimento.

Não estando preenchidas as duas primeiras condições de legitimidade, para a realização deste tratamento de dados é necessário o «consentimento expresso do titular», entendendo-se por consentimento qualquer manifestação de vontade, livre, específica e informada, nos termos da qual o titular aceita que os seus dados sejam

COMISSÃO NACIONAL
DE PROTECÇÃO DE DADOS

objeto de tratamento, o qual deve ser obtido através de uma "declaração de consentimento informado " onde seja utilizada uma linguagem clara e acessível.

Nos termos do artigo 10.º da LPD, a declaração de consentimento tem de conter a identificação do responsável pelo tratamento e a finalidade do tratamento, devendo ainda conter informação sobre a existência e as condições do direito de acesso e de retificação por parte do respetivo titular.

Os titulares dos dados, de acordo com a declaração de consentimento informado junta aos autos, apõem as suas assinaturas na mesma, deste modo satisfazendo as exigências legais.

O fundamento de legitimidade é o consentimento dos titulares dos dados.

A informação tratada é recolhida de forma lícita (cfr. alínea a) do n.º 1 do artigo 5.º da LPD), para finalidades determinadas, explícitas e legítimas (cfr. alínea b) do mesmo artigo).

Alerta-se a responsável pelo tratamento para a necessidade de incluir na declaração de consentimento informado um contacto para o exercício do direito de acesso e de oposição dos participantes no estudo, bem como a consagração expressa da possibilidade de desistência da colaboração no estudo, nos termos dos artigos 10.º, 11.º e 12.º da LPD.

III. Da Conclusão

Em face do exposto, a Comissão Nacional de Protecção de Dados (CNPD) autoriza o tratamento de dados pessoais *supra* apreciado, nos termos do n.º2 do artigo 7.º, da alínea a) do n.º1 do artigo 28.º e do n.º 1 do artigo 30.º da LPD, consignando-se o seguinte:

Responsável pelo tratamento: Maria José de Oliveira Santos

COMISSÃO NACIONAL
DE PROTECÇÃO DE DADOS

Finalidade: Estudo observacional e intervencional sobre a saúde sexual e reprodutiva em estudantes do ensino superior e a eficácia de um programa de intervenção.

Categoria de Dados pessoais tratados: código de participante, dados sociodemográficos (género, idade, nacionalidade, curso que frequenta, zona habitacional – aldeia/vila/cidade – estado civil, importância da religião, profissão dos pais, escolaridade dos pais, rendimento médio do agregado familiar por escalões), fontes de informação sobre a sexualidade e saúde reprodutiva, comportamentos sexuais e métodos contracetivos, conhecimentos e atitudes sobre saúde sexual e reprodutiva, procura de sensações sexuais, escala de autoeficácia geral, escala de autoeficácia no uso do preservativo e escala de satisfação com o suporte social.

Entidades a quem podem ser comunicados: Não há.

Formas de exercício do direito de acesso e retificação: Junto da investigadora principal.

Interconexões de tratamentos: Não há.

Transferências de dados para países terceiros: Não há.

Prazo de conservação dos dados: A chave de codificação e os questionários devem ser destruídos um mês após a defesa da Tese.

Lisboa, 22 de outubro de 2013

Ana Roque (Relatora), Luís Barroso, Carlos Campos Lobo, Helena Delgado António, Vasco Almeida, Luís Paiva de Andrade

Filipa Calvão (Presidente)

APÊNDICE G

Parecer da Comissão de Ética da universidade **(Paracer nº 2/2012)**

env. prot. SRH
27.11.2012

UNIVERSIDADE DE TRÁS-OS-MONTES E ALTO DOURO
COMISSÃO DE ÉTICA DA UTAD

Comissão de Ética da UTAD

**Parecer**	**Despacho**

**Parecer da Comissão de Ética Nº.**	2/2012
**Data:**	27-11-2012
**Assunto:**	**Doc1/CE/2012**<br>Questionário de caraterização e escalas - Doutoramento em Ciências de Enfermagem
**Requerente:**	Maria José de Oliveira Santos

A Comissão de Ética dá parecer favorável.

À superior consideração de V. Exa.

Pela Comissão de Ética
A Presidente da Comissão,

Maria da Conceição Azevedo

Maria da Conceição Azevedo

APÊNDICE H

Consentimento informado dos participantes dos grupos focais

CONSENTIMENTO INFORMADO

Caro Estudante

No âmbito do Curso de Doutoramento em Ciências de Enfermagem do Instituto de Ciências Biomédicas de Abel Salazar da Universidade do Porto, estamos a realizar um estudo/investigação subordinado ao tema "*Saúde Sexual e Reprodutiva de Estudantes do Ensino Superior: programa de intervenção*", com o objetivo de avaliar os conhecimentos, atitudes e comportamentos face à saúde sexual e reprodutiva em estudantes do ensino superior e com base nos resultados construir, uma proposta de um programa de intervenção de promoção da saúde sexual e reprodutiva. Nesta segunda fase o estudo desenvolve-se através de entrevistas focalizadas (*Focus Groups*).

A sua participação é muito importante, pois procuramos ter um novo olhar sobre as questões da saúde sexual e reprodutiva em estudantes do ensino superior, pelo que a partilha das suas vivências, do que sente e pensa sobre esta problemática é fundamental.

As suas informações são estritamente confidenciais e a análise garante o anonimato e a utilização dos dados na área da temática de pesquisa.

Esta participação será voluntária, pelo que poderá interrompe-la a qualquer momento bem como, responder ou partilhar somente o que entender.

Assim, depois de devidamente informado (a) **autorizo a participação** neste estudo:

____(localidade), __/__/____

Assinatura do Participante

____

Assinatura das Investigadoras

____.

____.
